# Magnetic topological quantum chemistry

**DOI:** 10.1038/s41467-021-26241-8

**Published:** 2021-10-13

**Authors:** Luis Elcoro, Benjamin J. Wieder, Zhida Song, Yuanfeng Xu, Barry Bradlyn, B. Andrei Bernevig

**Affiliations:** 1 Department of Condensed Matter Physics, University of the Basque Country UPV/EHU, Bilbao, Spain; 2 Department of Physics, Massachusetts Institute of Technology, Cambridge, MA USA; 3 Department of Physics, Northeastern University, Boston, MA USA; 4 Department of Physics, Princeton University, Princeton, NJ USA; 5 Max Planck Institute of Microstructure Physics, Halle, Germany; 6 Department of Physics and Institute for Condensed Matter Theory, University of Illinois at Urbana-Champaign, Urbana, IL USA; 7 Donostia International Physics Center, Donostia-San Sebastian, Spain; 8 IKERBASQUE, Basque Foundation for Science, Bilbao, Spain

**Keywords:** Computational methods, Electronic structure, Magnetic properties and materials, Topological insulators

## Abstract

For over 100 years, the group-theoretic characterization of crystalline solids has provided the foundational language for diverse problems in physics and chemistry. However, the group theory of crystals with commensurate magnetic order has remained incomplete for the past 70 years, due to the complicated symmetries of magnetic crystals. In this work, we complete the 100-year-old problem of crystalline group theory by deriving the small corepresentations, momentum stars, compatibility relations, and magnetic elementary band corepresentations of the 1,421 magnetic space groups (MSGs), which we have made freely accessible through tools on the Bilbao Crystallographic Server. We extend Topological Quantum Chemistry to the MSGs to form a complete, real-space theory of band topology in magnetic and nonmagnetic crystalline solids – Magnetic Topological Quantum Chemistry (MTQC). Using MTQC, we derive the complete set of symmetry-based indicators of electronic band topology, for which we identify symmetry-respecting bulk and anomalous surface and hinge states.

## Abstract (web summary)

The band topology of nonmagnetic crystals can be characterized by Topological Quantum Chemistry (TQC), whereas the band topology of magnetic crystals remains unexplored. Here, the authors extend TQC to the magnetic space groups to form a complete, real-space theory of band topology in magnetic and nonmagnetic crystalline solids.

## Introduction

A crystal is defined by its discrete translation symmetry. Over the past 140 years^1,2^, a tremendous number of physical phenomena have been shown to arise from the complicated mathematical structures implied by this otherwise simple definition of a crystal. For example, the symmetry and group theory of crystalline solids have been used to characterize phase transitions^3^, identify biological structures like the DNA double helix^4^, and, most recently, to elucidate the position-space origin of topological bands through the theories of Topological Quantum Chemistry (TQC)^5,6^ and equivalent works^7–9^.

**Fig. 1:**
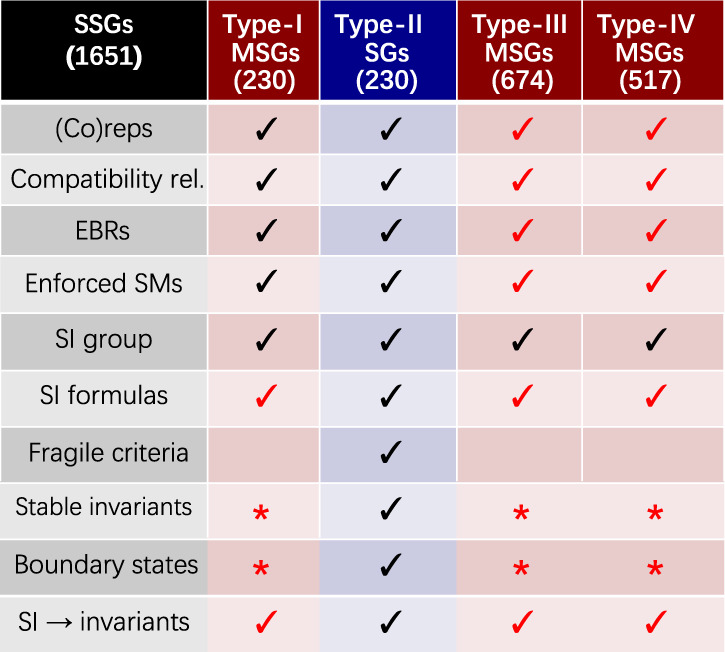
Summary of results. In this work, we have derived the complete sets of trivial bands [elementary band (co)representations (EBRs), see SN 17] and symmetry-indicated, spinful, stable topological bands in the 1,651 Shubnikov space groups [SSGs]. The EBRs subdivide into the physical EBRs of the 230 Type-II nonmagnetic space groups [SGs] and the magnetic EBRs [MEBRs] of the 1,421 Type-I, III, and IV magnetic SGs [MSGs, see SN 2]^10–12^. We have additionally performed the first complete calculation of the small (co)representations [(co)reps] and compatibility relations [see SN 11] for all 1,651 single and double SSGs, which we have made accessible through the tools listed in Table 1. These results comprise the theories of Magnetic Topological Quantum Chemistry (MTQC) and fermionic symmetry-based indicators (double SIs)^7,30–32,51^, which apply to all possible 3D magnetic and nonmagnetic crystals with mean-field Hamiltonians. We have also determined the physical bases of all double (spinful) symmetry-based indicators (SIs), and symmetry-indicated topological bulk and anomalous boundary states for all 1,651 double SSGs (SN 26). Lastly, the MEBRs of the Type-III and Type-IV MSGs computed in this work also facilitates the complete enumeration of symmetry-enforced magnetic topological semimetals (SMs) – examples are provided in Fig. 4c and in SN 15. In this figure, we have used red checks to indicate areas of magnetic topological band theory completed in this work, and we have used red stars to indicate areas in which we have solved complete subareas (such as the double SIs of the 1,651 double SSGs), but in which there remain topological features outside of the scope of this work, such as non-symmetry-indicated stable topological bands^25,26,34,55^ and bosonic (spinless) topological crystalline insulators (TCIs).

In time-reversal- (${{{{{{{\mathcal{T}}}}}}}}$-) symmetric, periodic systems – which most familiarly include nonmagnetic crystalline solids – the energy (Bloch) eigenstates respect the symmetries of the nonmagnetic (Type-II) Shubnikov space group (SSGs)^10–12^ [see Fig. 1 and Supplementary Note (SN) 2]. Though there are 230 Type-II SSGs, including SSGs with complicated combinations of glide and screw symmetries, the group theory of nonmagnetic crystalline solids has been largely solved for over 40 years^11^. In particular, the enumeration of the irreducible momentum-space [small] corepresentations [coreps, see SN 13], and a partial enumeration of the space group (elementary band) coreps [EBRs, see SN 17] of the Type-II SSGs were completed prior to the advent of personal and distributed computing^11,13–16^. In recent years, the group theory of Type-II SSGs has facilitated a revolution in the search for topological insulators (TIs)^17–22^ and topological crystalline insulators (TCIs)^23–26^, including the recent discovery of higher-order TCIs (HOTIs)^27–29^ through TQC and related methods^30–34^.

However, the 230 Type-II SSGs represent only a fraction of the 1,651 (magnetic and nonmagnetic) SSGs (MSGs and SGs, respectively, see Fig. 1 and SN 2). Specifically, while Type-II SGs contain unitary symmetries and ${{{{{{{\mathcal{T}}}}}}}}$ about any point ($\{{{{{{{{\mathcal{T}}}}}}}}| {{{{{{{\bf{0}}}}}}}}\}$), there are also Type-I MSGs with only unitary symmetries, Type-III MSGs that contain combinations of ${{{{{{{\mathcal{T}}}}}}}}$ and rotation or reflection (e.g. $\{{C}_{2z}\times {{{{{{{\mathcal{T}}}}}}}}| {{{{{{{\bf{0}}}}}}}}\}$, in which *C*_*ni*_ is a rotation by 2*π*/*n* about the *i* axis), and Type-IV MSGs that contain the combination of ${{{{{{{\mathcal{T}}}}}}}}$ and fractional lattice translation ($\{{{{{{{{\mathcal{T}}}}}}}}| {{{{{{{\bf{a}}}}}}}}/2\}$, in which **a** is an odd-integer linear combination of lattice vectors). The small (co)reps and magnetic EBRs [MEBRs] of the MSGs are necessary for a wide range of physical applications, including characterizing magnetic topological semimetals (SMs)^35–38^, TIs^39,40^, and TCIs^41,42^. Beyond topological materials, the magnetic small (co)reps are also required to construct theories of magnetic phase transitions with nonzero **q** vectors from magnetic structure data obtained through neutron diffraction experiments^43,44^, and to characterize ${{{{{{{\mathcal{T}}}}}}}}$-breaking superconducting phases^45^ with nonzero Cooper-pair momenta, such as Fulde-Ferrell-Larkin-Ovchinnikov states^46–49^. Nevertheless, due to the relative complexity of the MSGs, and despite a number of significant partial tabulations^50,51^, progress towards completing the group theory of magnetic crystals has largely stalled for the past 70 years^10,11^.

**Fig. 2:**
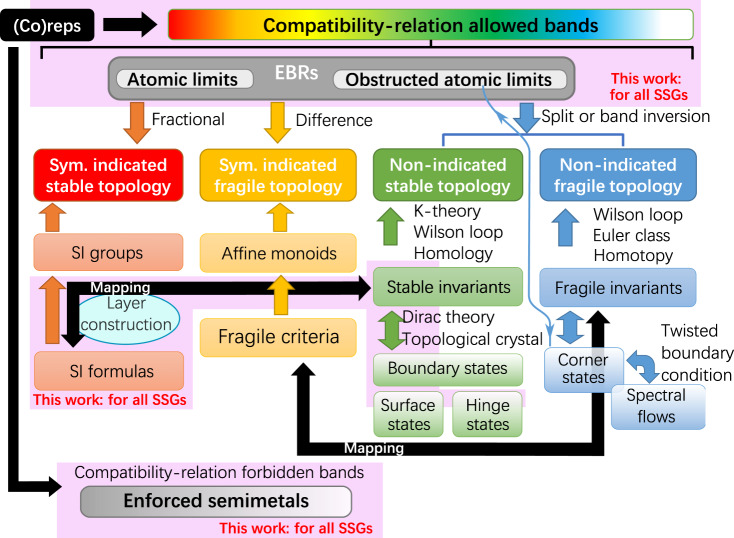
Magnetic Topological Quantum Chemistry in the scheme of topological band theory. The complete scheme of topological band theory for 3D crystals, following the framework and notation established in refs. ^5,6,31,66,67^. Through crystal symmetry eigenvalues [small (co)reps] in momentum space (SN 13), the compatibility relations (SN 16) indicate whether a set of bands is allowed by symmetry to be energetically isolated from other bands in the energy spectrum. If the bands are energetically isolated, then there exists a wide range of methods for diagnosing whether the bands exhibit the stable topology of topological insulators (TIs) and TCIs^17–20,23–34,54^, fragile topology^52,54,55,64–67^, or the polarization-nontrivial topology of obstructed atomic limits^5,54,84^. For example, as detailed in refs. ^7,30–32,51,66,67^, the small (co)reps of a set of isolated bands comprise momentum-space symmetry data that can be mapped to position-space topology and boundary states through stable and fragile SIs and real-space invariants. If the bands are instead required by symmetry to cross, then the bands characterize a topological SM, which may exhibit surface^38^ or hinge^34,54^ states. In this figure, the pink boxes indicate areas of topological band theory completed in this work.

In this work, we use a combination of computational and analytic methods to derive the small (co)reps and MEBRs of the MSGs, completing the 100-year-old problem of crystalline group theory. Using the small (co)reps and MEBRs, we construct a complete position-space theory of mean-field band topology in the 1,651 single (spinless) and double (spinful) SSGs – Magnetic Topological Quantum Chemistry (MTQC) – that subsumes the earlier theory of TQC^5,6^ [see Fig. 2]. The completeness of MTQC stems from the completeness of our tabulation of the MEBRs. Specifically, even in MSGs in which trivial and topological states cannot be distinguished by symmetry eigenvalue labels, the MEBRs provide a complete basis for constructing and analyzing all possible lattice models of trivial, gapless, and stable and fragile topological insulating phases (for specific examples of non-symmetry-indicated topological phases analyzed using EBRs, see refs. ^34,52–55^). To access the data generated for this work, we have implemented several programs on the Bilbao Crystallographic Server (BCS)^56,57^, which are listed in Table 1. Each of the programs listed in Table 1 contains data for both the magnetic and nonmagnetic SSGs, and therefore replaces an existing tool on the BCS. In the Results section below, we will first describe the underlying machinery of MTQC through which band (co)reps in momentum space are induced from magnetic atomic (Wannier) orbitals in position space. Next, we will detail the topological information that can be inferred from the MEBRs, which include lattice models for magnetic exceptions to fermion doubling theorems^26,58^, and symmetry-based indicators (SIs)^7,30–32,51^ for magnetic SMs, TIs, and TCIs (see SN 26). In particular, in this work, going beyond the earlier tabulation of the magnetic SI groups in ref. ^51^, we have for the first time generated the complete double SI formulas, as well as symmetry-respecting topological bulk and boundary states for all 1,651 double SSGs, which characterize spinful electronic states in solid-state materials. Through this calculation, we have obtained the complete set of symmetry-indicated 3D spinful (fermionic) topological phases.

**Table 1:** Applications on the Bilbao Crystallographic Server implemented for MTQC.

BCS Applications Implemented for MTQC		
Application	Contents	Description
MKVEC	Momentum stars of the MSGs	SN 12
Corepresentations	Small and full magnetic (co)reps	SN 13
MCOMPREL	Compatibility relations in the MSGs	SN 16
CorepresentationsPG	Magnetic site-symmetry group (co)reps	SN 18
MSITESYM	Magnetic small (co)reps at one **k** point induced from a site **q**	SN 22
MBANDREP	MEBRs of the MSGs	SN 23

For this work, we have implemented the Bilbao Crystallographic Server (BCS) programs listed in this table to access group-theoretic properties of the MSGs that we have computed to complete the theory of MTQC. In order, this table contains the name of the program, the data accessible through the program, and the Supplementary Note in which the program is detailed. In addition to the properties of the MSGs listed in this table, each tool contains the analogous properties of the 230 Type-II (nonmagnetic) SGs. Therefore, as respectively detailed in each listed Supplementary Note, each program in this table subsumes the content of an existing program on the BCS.

We find that many of the symmetry-indicated spinful magnetic topological phases consist of familiar Weyl SMs with surface Fermi arcs^59–61^, 3D quantum anomalous Hall (QAH) phases constructed from layered integer quantum Hall states (2D Chern insulators)^39,62^, and axion insulators (AXIs), which are equivalent to 3D TIs with magnetically gapped surface states on particular crystal facets^21,55,63^. However, we also in this work discover the existence of previously unidentified non-axionic magnetic HOTIs with mirror-protected helical hinge states (see SN 33). We conclude by briefly discussing future directions in magnetic group theory, including the prediction of spinless (bosonic) TCIs, and applications of magnetic crystal symmetry beyond mean-field theory. We have also included extensive Supplementary Notes containing additional details of our methodology, historical commentary, references, documentation for the BCS programs introduced in this work, and data for the EBRs and double SIs (see SN 1 and 36).

## Results

### MEBRs from magnetic atomic orbitals

**Fig. 3:**
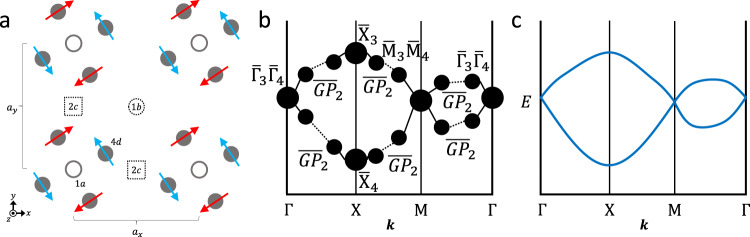
Magnetic band (co)reps from magnetic atomic orbitals. **a** A crystal with lattice-commensurate magnetic order. In the mean-field, the basis states of the electronic Hamiltonian of the crystal in (**a**) are magnetic atomic orbitals (SN 18). When weakly coupled, the magnetic atomic orbitals in (**a**) continue to form a set of exponentially localized, symmetric Wannier orbitals^5,15,16,53^ that transform in the (co)reps of magnetic site-symmetry groups [SN 7]. **b** The magnetic site-symmetry (co)reps in (**a**) induce a band (co)rep in momentum [**k**] space. **c** Correspondingly, the Bloch eigenstates of the Fourier-transformed electronic Hamiltonian of the magnetic crystal in (**a**) transform in the band (co)rep in (**b**) [see SN 22].

To construct the theory of MTQC, we first tabulate the EBRs of the 1,651 SSGs, which include the MEBRs of the MSGs [Fig. 3b and SN 17]. In each SSG, the EBRs correspond to the independent topologically trivial bands. Specifically, each EBR corresponds to a (set of) band(s) that can be inverse-Fourier-transformed into exponentially localized, symmetric Wannier orbitals, and the set of EBRs in each SSG forms the basis for all energetically isolated sets of trivial bands (i.e. bands without stable or fragile topology)^5–9,15,16,30–34,52–55,64–67^.

We begin by considering a nonmagnetic crystal that is furnished with atomic orbitals that are sufficiently weakly coupled as to not invert bands at any **k** point in the Brillouin zone (BZ). Each atomic orbital occupies a site in a Wyckoff position of a Type-II SG. Crucially, the atomic orbitals on each site transform in direct sums of the irreducible coreps of the site-symmetry group (SN 7 and 18), which is necessarily isomorphic to one of the 32 nonmagnetic point groups (PGs, see SN 8).

We next consider the case in which the crystal undergoes a transition into a phase with lattice-commensurate magnetic order [Fig. 3a]. The onset of magnetism lowers the crystal symmetry from a Type-II SG into either a Type-I, III, or IV MSG (see refs. ^10–12^ and SN 3, 5, and 6, respectively). Specifically, in the limit in which the magnetic moments are taken to be decoupled from the underlying lattice, the crystal of moments may appear to exhibit additional symmetries, such as global and local spin rotation. However, when the coupling between the spins and the underlying lattice is not ignored, the magnetic phase transition strictly lowers the system symmetry to that of a magnetic Shubnikov subgroup *M* of the Type-II SG *G* of the parent nonmagnetic crystal^11^.

Hence, the magnetic order also lowers the symmetry at each site in the crystal. This can be seen by recognizing that $\{{{{{{{{\mathcal{T}}}}}}}}| {{{{{{{\bf{0}}}}}}}}\}$ is an element of every site-symmetry group in a nonmagnetic crystal, but cannot be an element of any site-symmetry group in a magnetic crystal (SN 9). For example, in a solid-state material with magnetic atoms, the orbitals of nonmagnetic atoms elsewhere in the unit cell are necessarily subject to a background magnetic potential (see SN 10). While the energy scale of the magnetic potential is detail-dependent, the magnetic potential on the atoms considered to be nonmagnetic is only exactly zero in a fine-tuned limit. This statement remains valid whether individual atoms in the magnetic crystal are taken to host localized magnetic dipole moments, or whether the magnetic crystal is taken to consist of multi-atom clusters with higher magnetic multipole moments^68,69^. Consequently, independent of the phenomenological microscopic treatment of the magnetic order, each site-symmetry group in the magnetic crystal is isomorphic to one of the 90 crystallographic magnetic point groups (MPGs, see SN 8). In a solid-state material in which the effects of magnetism can be approximated through mean-field theory, the atomic orbitals of the original crystal [e.g., *s* and *p*_*x*,*y*_] split into magnetic atomic orbitals [e.g., *s* and *p*_*x*_ ± *ip*_*y*_] that transform in (co)reps of the MPGs [see SN 19, 20, and 21]. For this work, we have implemented the CorepresentationsPG tool (http://www.cryst.ehu.es/cryst/corepresentationsPG, detailed in SN 18), through which users can access the (co)reps of all 122 single and double PGs and MPGs.

Next, the magnetic site-symmetry (co)reps in each Wyckoff position in the crystal induce a band (co)rep into *M* [Fig. 3(b)]. The set of all possible band (co)reps in each MSG is spanned by the MEBRs of *M*. In this work, we have for the first time computed the 22,611 MEBRs of all 1,191 single and double Type-III and Type-IV MSGs, which – along with the 5,641 MEBRs of the 230 Type-I MSGs and the 4,757 EBRs of the 230 Type-II SGs previously calculated for TQC^5,15,16,53^ [Fig. 1] – can be accessed through the MBANDREP tool on the BCS (http://www.cryst.ehu.es/cryst/mbandrep, further detailed in SN 23). To enumerate the MEBRs of each MSG *M*, we begin by inducing band (co)reps from each irreducible (co)rep of one site-symmetry group within each of the highest-symmetry [*i.e*. maximal, see SN 9] Wyckoff positions in *M*. We next exclude the exceptional cases in which the induced band (co)reps are equivalent to direct sums of other bands (co)reps [SN 24 and 37]. The remaining band (co)reps are defined as elementary [*i.e*. MEBRs]; statistics and further details for the MEBRs are provided in SN 25 and 38.

Importantly, just as each MEBR is the Fourier-transformed description of a crystal of site-symmetry (co)reps, the Wannierizable bands that transform in each MEBR are the Bloch eigenstates of the Fourier-transformed electronic Hamiltonian of weakly coupled magnetic atomic orbitals [Fig. 3c and SN 22]. Consequently, in each momentum star of each MSG – which are accessible through the MKVEC tool (http://www.cryst.ehu.es/cryst/mkvec, see SN 12) – each MEBR contains a set of full (co)reps that is specified by the Wyckoff position from which the MEBR is induced. Each full (co)rep can be reduced through subduction to a set of irreducible small (co)reps at each k point that are known as the symmetry data [Fig. 3b]. The complete set of small and full (co)reps of each MSG and direct dependencies between the site-symmetry (co)reps at **q** and the induced symmetry data at **k** are respectively accessible through the Corepresentations (http://www.cryst.ehu.es/cryst/corepresentations, detailed in SN 13) and MSITESYM (http://www.cryst.ehu.es/cryst/msitesym, detailed in SN 22) tools. Lastly, to determine whether the bands that transform in the induced symmetry data are required by symmetry to be degenerate or cross along high-symmetry paths in the BZ, we have computed the magnetic small (co)rep compatibility relations, which are accessible through the MCOMPREL tool introduced in this work (https://www.cryst.ehu.es/cryst/mcomprel, detailed in SN 16).

Before discussing topological applications of the MEBRs and the small and full (co)reps of each MSG, we will first briefly discuss the advances made in this work in the context of previous studies of magnetic symmetry and group theory. First, in the 1960’s, Miller and Love in ref. ^50^ performed the largest tabulation of magnetic small (co)reps prior to this work. Specifically, in ref. ^50^, Miller and Love computed the single- and double-valued irreducible small (co)reps of the little groups of each MSG at high-symmetry points and along high-symmetry lines, but not along high-symmetry planes or in the BZ interior, which are required to complete the insulating compatibility relations for each MSG (SN 16) and to compute the MEBRs (SN 17). Additionally, the magnetic small (co)reps computed in ref. ^50^ are displayed in difficult-to-read tables outputted directly from computer code, and are hence difficult to verify. For this work, we have implemented the Corepresentations tool on the BCS [SN 13], which represents the first complete and publicly available online tabulation of the magnetic small (co)reps. Through Corepresentations, users may obtain the matrix representatives in each magnetic small (co)rep of the generating symmetries of the magnetic little group at each **k** point in each MSG in an accessible format readily suited towards analyzing the output of tight-binding and first-principles calculations [see SN 14 and 15 for representative examples of the output of Corepresentations]. We additionally note that prior to this work, Evarestov Smirnov, and Egorov in ref. ^16^ introduced a method for obtaining the MEBRs of the MSGs and computed representative examples, but did not perform a large-scale tabulation of MEBRs or establish a connection to magnetic band topology. In this work, we have employed a method equivalent to the procedure in ref. ^16^ to perform the first complete tabulation of the single- and double-valued MEBRs of the 1,421 MSGs (see SN 23), which we have additionally made publicly accessible through the MBANDREP tool on the BCS.

Having computed the MEBRs of the single and double MSGs and established the theory of MTQC, we will next describe two applications of the MEBRs and MTQC to the discovery and characterization of novel topological phases of matter: elucidating the relationship between topological SMs and TCIs through symmetry-enhanced fermion doubling theorems, and extending the SIs of stable band topology^7,30–32,34^ to the MSGs.

### Symmetry-enhanced fermion doubling theorems

The surface states of each *d*-dimensional [*d*-D] TI and TCI are termed anomalous because the surface states cannot be stabilized in a (*d* − 1)-D lattice model with the symmetries of the TI or TCI surface. In 3D TIs, AXIs, and Chern (QAH) insulators, the boundary anomaly and bulk response can be understood from the perspective of well-known high-energy field theories^21,62,63^. For example, the bulk of a 3D TI is characterized by a quantized axionic magnetoelectric response governed by a Lagrangian density ${{{{{{{{\mathcal{L}}}}}}}}}_{EM}\propto \theta {{{{{{{\bf{E}}}}}}}}\cdot {{{{{{{\bf{B}}}}}}}}$ in which the axion angle *θ* is pinned to the nontrivial value *θ* mod 2*π* = *π* by $\{{{{{{{{\mathcal{T}}}}}}}}| {{{{{{{\bf{0}}}}}}}}\}$ symmetry^21,63^. As a consequence of the bulk axionic topology, each surface of a 3D TI exhibits an odd number of twofold-degenerate Dirac cones, representing an exception to the 2D parity anomaly – a fermion doubling theorem that mandates the existence of an even number of symmetry-stabilized twofold Dirac cones in any 2D system with a lattice (-regularized) description^19–21,26,63^. However, in other gapped topological phases, such as 3D helical TCIs and HOTIs, the boundary anomalies and bulk response theories have not yet been elucidated in the language of high-energy field theory^26,28,29,32,34,55^. Nevertheless, as shown in refs. ^26,29,32^, the anomalous surface states of *d*-D TIs and TCIs may be classified through a comparison to the complete set of (*d* − 1)-D lattice models of symmetry-stabilized topological SMs.

It is possible to evade a fermion doubling theorem by either stabilizing the anomalous nodal point[s] on the (*d* − 1)-D boundary of a *d*-D topological [crystalline] insulator [*i.e*. through spectral flow], or by modifying one of the system symmetries so that the symmetry is represented differently at low and high energies. For example, the matrix representatives of $\{{{{{{{{\mathcal{T}}}}}}}}| {{{{{{{\bf{0}}}}}}}}\}$ and $\{{{{{{{{\mathcal{T}}}}}}}}| {{{{{{{\bf{a}}}}}}}}/2\}$ are the same near **k** = **0**, but differ at larger **k** (see SN 15). In effect, systems with $\{{{{{{{{\mathcal{T}}}}}}}}| {{{{{{{\bf{0}}}}}}}}\}$ symmetry and integer lattice translations are nonmagnetic (see SN 4) and constrained by fermion doubling theorems that derive from $\{{{{{{{{\mathcal{T}}}}}}}}| {{{{{{{\bf{0}}}}}}}}\}$ symmetry^26^, whereas systems generated by $\{{{{{{{{\mathcal{T}}}}}}}}| {{{{{{{\bf{a}}}}}}}}/2\}$ and integer lattice translations are antiferromagnetic (see SN 6), and are not constrained by the same doubling theorems^58^. As discussed in ref. ^70^, it is desirable to identify lattice-regularizable systems that circumvent fermion doubling theorems, because correlation effects in these systems can be modeled without also incorporating complicated and numerically intensive bulk degrees of freedom. Many of the symmetry-enhanced fermion doubling theorems exceptions discovered to date rely on emergent unitary particle-hole symmetries that act nonlocally^70,71^, and relate to the anomalous surface states of particle-hole-symmetric TCIs in Class AIII in the nomenclature of ref. ^72^. However, emergent unitary particle-hole is typically only a valid symmetry in a handful of solid-state materials, and only then at low energies. As we will discuss below, by considering nodal degeneracies stabilized by MSG symmetries – which are conversely valid in solid-state magnetic materials at all energies without fine-tuning – it is possible to systematically enumerate symmetry-enhanced, single-particle fermion doubling theorems, as well as materials-relevant models that circumvent symmetry-enhanced fermion doubling.

The elucidation of a (symmetry-enhanced) fermion doubling theorem and an example of its evasion has historically required a significant theoretical effort. For example, in ref. ^73^, it was shown that unpaired fourfold-degenerate Dirac fermions cannot be stabilized in lattice models of 2D, ${{{{{{{\mathcal{T}}}}}}}}$-symmetric SMs. Through an exhaustive analysis of the symmetry-enforced spectral flow in 3D crystals, a 3D ${{{{{{{\mathcal{T}}}}}}}}$-symmetric TCI with an unpaired (anomalous), symmetry-stabilized, fourfold surface Dirac fermion was identified in ref. ^26^. Crucially, using the fourfold Dirac fermion doubling theorem established in ref. ^73^, the authors of ref. ^26^ were able to diagnose the surface fourfold Dirac fermion as anomalous without establishing a bulk or boundary field theory. Lastly, it was subsequently shown in ref. ^58^ that fourfold Dirac fermion doubling can also be evaded in lattice models of 2D magnetic SMs with the symmetry $\{{{{{{{{\mathcal{T}}}}}}}}| {{{{{{{\bf{a}}}}}}}}/2\}$ common to Type-IV 2D symmetry (wallpaper or layer) groups (see SN 15). Hence, one may infer the existence of novel quantized response effects and condensed-matter realizations of high-energy anomalies by exploiting the restrictions imposed by crystal symmetries on lattice models of SMs, TIs, and TCIs.

Because a complete tabulation of the magnetic small (co)reps was previously unavailable, then earlier theoretical searches for magnetic exceptions to fermion doubling theorems, such as ref. ^58^, were performed ad hoc. However, the magnetic small (co)reps, the magnetic compatibility relations, and the MEBRs computed in this work allow, for the first time, the immediate enumeration of the complete set of lattice models of symmetry-stabilized magnetic SMs in three or fewer dimensions. Below, we will outline the method for enumerating the complete set of stable magnetic SMs using the data generated in this work. We will then detail the simplest possible magnetic fermion doubling exception that can be obtained by considering the set of lattice models of 1D magnetic SMs inferred from the 1D MEBRs. Despite the simplicity of the example below, we find that it has not been addressed from the intuitive picture of mean-field magnetic band theory in previous literature. In SN 34, we also introduce a doubling theorem for twofold Dirac fermions in magnetic 2D symmetry groups, which we find to be evaded on the surfaces of the non-axionic magnetic HOTIs discovered in this work (see SN 35).

To begin, by occupying the bands that transform in each connected branch of each MEBR with integer-valued numbers of electrons increasing from one to one less than the dimension of the MEBR (see refs. ^5,6^ and SN 16, 25, and 38), we have obtained the exhaustive list of connectivity-enforced 3D magnetic SMs. The remaining stable 3D SMs can then be obtained through band inversion in lattice models constructed from sums of MEBRs (or branches of decomposable MEBRs, see SN 25) using the magnetic compatibility relations, as well as previously established topological invariants for nodal fermions at low-symmetry **k** points. Specifically, in each MSG, the minimal multiplicity of stable nodal points may be obtained by considering the small (co)reps along all high-symmetry BZ lines and planes [which are accessible through Corepresentations, see SN 13], in addition to the nodal points stabilized by topological invariants evaluated along with closed manifolds in the BZ (e.g. Weyl points, see refs. ^29,55,59–61^). Lastly, the complete set of 2D and 1D lattice models of magnetic SMs may be obtained by restricting the above procedure to MSGs that are isomorphic modulo integer lattice translations to layer and rod groups, respectively (see SN 2 and refs. ^26,54,58^).

**Fig. 4:**
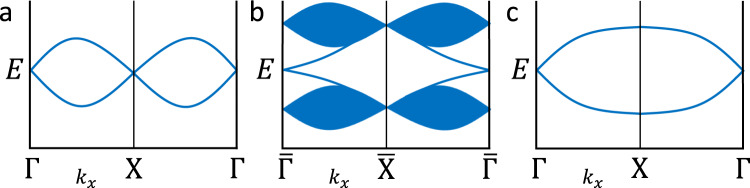
Dirac fermion doubling from elementary band (co)representations. **a** A pair of spinful bands that transform in the double-valued EBR of a Type-II line group generated by $\{{{{{{{{\mathcal{T}}}}}}}}| 0\}$ and lattice translation [isomorphic to Type-II double SG 1.2 $P11^{\prime}$ modulo lattice translations]. At half-filling, there are two, twofold Dirac fermions in (**a**), representing an example of twofold Dirac fermion doubling in 1D. **b** The edge spectrum of a 2D TI features an unpaired twofold Dirac fermion that circumvents the doubling theorem in (a)^17,18,21^. (c) A pair of spinful bands that transform in the double-valued MEBR of a Type-IV magnetic line group generated by $\{{{{{{{{\mathcal{T}}}}}}}}| 1/2\}$ [isomorphic to Type-IV double MSG 1.3 *P*_*S*_1 modulo lattice translations]. At half-filling, the spectrum in (**c**) consists of an unpaired twofold Dirac fermion with the same *k* ⋅ *p* Hamiltonian as the Dirac points at Γ and *X* in (**a**) and the 2D TI edge in (**b**), representing a magnetic exception to twofold Dirac fermion doubling in 1D.

In Fig. 4, we show the simplest example of a fermion doubling exception obtained using the MEBRs. First, in Fig. 4(a), we show a pair of spinful bands in a nonmagnetic 1D crystal that transform in the double-valued EBR of the Type-II 1D double symmetry (line) group generated by $\{{{{{{{{\mathcal{T}}}}}}}}| 0\}$ and lattice translation. At half-filling, the band structure in Fig. 4(a) exhibits two, twofold Dirac fermions per 1D BZ. Additionally, in the absence of chiral symmetry – which is not generically a symmetry of crystalline solids – unpaired nodal points away from Γ and *X* in Fig. 4a cannot be stabilized. Specifically, even if a nodal point stabilized by reflection or rotation symmetry is present at a point *k*_*x*_, $\{{{{{{{{\mathcal{T}}}}}}}}| 0\}$ symmetry mandates the existence of a second stable nodal point at − *k*_*x*_. By further investigating the symmetry-allowed band connectivities in all Type-II 1D (line and rod) supergroups of the line group in Fig. 4(a) (which can be inferred from the Corepresentations, MCOMPREL, and MBANDREP tools in Table 1), we conclude that an odd number of twofold Dirac fermions cannot be stabilized in 1D nonmagnetic, spinful lattice models.

However, it is well established that twofold Dirac fermion doubling in 1D is evaded on the edge of a 2D TI through spectral flow^17,18,21^ [Fig. 4(b)]. Recently, in ref. ^74^, the author performed an intensive, high-energy field-theory calculation demonstrating that a 1D lattice model with an unpaired twofold Dirac fermion could be formulated by invoking an exotic, non-on-site ${{{{{{{\mathcal{T}}}}}}}}$-like symmetry. However, in this work, we recognize that a simpler, alternative interpretation of a non-on-site ${{{{{{{\mathcal{T}}}}}}}}$ symmetry is the antiferromagnetic (AFM) symmetry $\{{{{{{{{\mathcal{T}}}}}}}}| 1/2\}$ common to all Type-IV magnetic line groups (SN 6). Correspondingly, in Fig. 4(c), we show a pair of spinful bands that transform in the double-valued MEBR of a Type-IV magnetic double line group generated by $\{{{{{{{{\mathcal{T}}}}}}}}| 1/2\}$. When the bands in Fig. 4c are half-filled, the band structure features an unpaired twofold Dirac fermion with the same *k* ⋅ *p* Hamiltonian as the anomalous twofold Dirac fermion on the edge of a 2D TI [Fig. 4b]. Hence, the crystal in Fig. 4c represents a magnetic exception to twofold Dirac fermion doubling in 1D, analogous to the magnetic exception to fourfold Dirac fermion doubling in 2D demonstrated in ref. ^58^.

### Symmetry-based indicators of stable band topology in the 1,651 double SSGs

If a set of bands in a crystal is energetically isolated along all high-symmetry BZ lines and planes, then a subset of the topological properties of the bands may be inferred through the eigenvalues of unitary crystal symmetries. Restricting focus to symmetry-indicated stable topological bands, which do not transform in integer-valued linear combinations of EBRs [see SN 27], the crystal symmetry eigenvalues that indicate stable topology [encoded in the small (co)reps of the isolated bands, see SN 13] form the symmetry-based indicators (SIs) of stable band topology [see SN 28 and refs. ^7,30–32,34^]. In each SSG, the SIs consist of an SI group (e.g. ${{\mathbb{Z}}}_{4}\times {{\mathbb{Z}}}_{2}^{3}$) and an SI formula (e.g. the Fu-Kane parity criterion for 3D TIs^20^, see SN 29 for an additional detailed example). The complete SIs of spinful band topology in nonmagnetic 3D crystals – which we term the double SIs of the 230 Type-II double SGs – were previously computed in refs. ^7,31,32^. Following those works, the single and double SI groups in the 1,421 MSGs were computed in ref. ^51^, but the authors of that work did not compute the SI formulas or determine the physical interpretation (*i.e*. the bulk topology and anomalous boundary states) of the magnetic bands with nontrivial SIs [see Fig. 1].

In this work, we have computed the complete set of double SI groups and formulas for spinful band topology in all 1,651 double SSGs. We have further determined symmetry-respecting bulk and anomalous surface and hinge states for all nontrivial values of the double SIs. The SI formulas introduced in this work (see SN 31 and 32) have been unified into a consistent basis in which all previously identified nonmagnetic double SI formulas correspond to established nonmagnetic SM, TI, and TCI phases, and in which the SIs of symmetry-indicated TIs and TCIs with the same bulk topology (e.g. 3D TIs and AXIs with the common nontrivial axion angle *θ* = *π*) are related by intuitive SI subduction relations. To summarize our calculation of the double SIs, we begin by considering a set of bands that is energetically isolated along all high-symmetry lines and planes, such that the Bloch states across all **k** points transform in small (co)reps that satisfy the insulating compatibility relations [see SN 16]. If the bands exhibit nontrivial SIs, then the bands cannot be inverse-Fourier-transformed into exponentially localized, symmetric Wannier orbitals. This can be seen by recognizing that the set of bands does not transform in an integer-valued linear combination of EBRs. Consequently, the set of bands either forms a topological semimetal with nodal points in the BZ interior – which we term a Smith-index SM (SISM), or corresponds to a stable TI or TCI phase with anomalous 2D surface or 1D hinge states^7,17–20,23–34,54^.

Because there are 1,651 double SSGs, then individually calculating the bulk and anomalous surface and hinge states and physical basis for each nontrivial SI in each double SSG is a practically intractable task. However, in this work, we have reduced the size of the calculation by recognizing that the double SIs in each double SSG *G* continue to exhibit unique, nontrivial values – termed the minimal double SIs – when the SI topological bands in *G* are subduced onto a double SSG *M* from the considerably smaller subset of 34 minimal double SSGs. In SN 30, we rigorously detail the procedure for obtaining the minimal double SIs, and in SN 39, we list the minimal double SSG associated to each double SSG. Across all of the minimal double SIs, we have implemented a consistent physical basis for the SI formulas, determined symmetry-respecting topological bulk and boundary states, and formulated layer constructions of the stable TI and TCI phases – the minimal double SIs are summarized in Table 2 and the details of our SI calculations are provided in SN 26.

**Table 2:** The minimal double SIs of spinful band topology in all 1,651 double SSGs.

Minimal Double SIs of Spinful Band Topology the 1,651 Magnetic and Nonmagnetic Double SSGs						
SI	Minimal Double SSG(s)	Bulk Topology		SI	Minimal Double SSG(s)	Bulk Topology
*η*_4*I*_	2.4 $P\bar{1}$	WSM/QAH/AXI		${z}_{4m,\pi }^{\pm }$	83.43 *P*4/*m*	weak TI/weak TCI
*z*_2*I*,*i*_	2.4 $P\bar{1}$	QAH		${z}_{4m,0}^{+}$	84.51 *P*4_2_/*m*	QAH/weak TI/weak TCI
${\eta }_{2I}^{\prime}$	2.4 $P\bar{1}$	AXI		*z*_8_	83.44 $P4/m1^{\prime}$, 123.339 *P*4/*mmm*	AXI/TCI/HOTI
*z*_2*R*_	3.1 *P*2, 41.215 $Ab^{\prime} a^{\prime} 2$	QAH		*z*_3*R*_	147.13 $P\bar{3}$	QAH
*δ*_2*m*_	10.42 *P*2/*m*	QAH/AXI/TCI		*z*_6*R*_	168.109 *P*6	QAH
${z}_{2m,\pi }^{\pm }$	10.42 *P*2/*m*	QAH/weak TI/weak TCI		*δ*_3*m*_	174.133 $P\bar{6}$	QAH/AXI/TCI
*z*_4_	2.5 $P\bar{1}1^{\prime}$, 47.249 *Pmmm*,	AXI/TCI/HOTI		${z}_{3m,\pi }^{\pm }$	174.133 $P\bar{6}$	weak TI/weak TCI
	83.45 $P4^{\prime} /m$					
${z}_{4}^{\prime}$	135.487 $P{4}_{2}^{\prime}/mbc^{\prime}$	AXI/TCI		*δ*_6*m*_	175.137 *P*6/*m*	QAH/AXI/TCI
*z*_2*w*,*i*_	2.5 $P\bar{1}1^{\prime}$, 47.249 *Pmmm*,	weak TI/weak TCI		${z}_{6m,\pi }^{\pm }$	175.137 *P*6/*m*	weak TI/weak TCI
	83.45 $P4^{\prime} /m$					
*z*_4*R*_	75.1 *P*4	QAH		${z}_{6m,0}^{+}$	176.143 *P*6_3_/*m*	QAH/weak TI/weak TCI
${z}_{2R}^{\prime}$,	27.81 $Pc^{\prime} c^{\prime} 2$, 54.342 $Pc^{\prime} c^{\prime} a$,	QAH		*z*_12_	175.138 $P6/m1^{\prime}$, 191.233 *P*6/*mmm*	AXI/TCI/HOTI
${z}_{2R}^{^{\prime\prime} }$	56.369 $Pc^{\prime} c^{\prime} n$, 60.424 $Pb^{\prime} cn^{\prime}$,					
	77.13 *P*4_2_, 110.249 $I{4}_{1}c^{\prime} d^{\prime}$					
*z*_4*S*_	81.33 $P\bar{4}$	QAH		${z}_{12}^{\prime}$	176.144 $P{6}_{3}/m1^{\prime}$	AXI/TCI/HOTI
*δ*_2*S*_	81.33 $P\bar{4}$	WSM		${z}_{4R}^{\prime}$	103.199 $P4c^{\prime} c^{\prime}$	QAH
*z*_2_	81.33 $P\bar{4}$	AXI		${z}_{6R}^{\prime}$	184.195 $P6c^{\prime} c^{\prime}$	QAH
*δ*_4*m*_	83.43 *P*4/*m*	QAH/AXI				

**Fig. 5:**
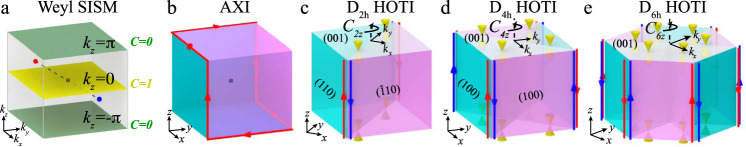
The five families of 3D symmetry-indicated, spinful, strong topological phases. In this work, we have computed the complete set of symmetry-indicated spinful topological phases of 3D magnetic and nonmagnetic crystalline solids (see SN 26). We find that, for spinful bands in 3D crystals that satisfy the insulating the compatibility relations along all high-symmetry lines and planes [see SN 16], there are only five families of symmetry-indicated strong topological phases: (**a**) Smith-index Weyl SMs (Weyl SISMs), (**b**) axion insulators (AXIs) and 3D TIs defined by the nontrivial axion angle^19–21,55,63^ *θ* = *π* [e.g., MnBi_2_Te_4_^41,42^], (**c**) helical TCIs and higher-order TCIs (HOTIs) equivalent to two superposed AXIs with the same orbital hybridization and twofold rotation or rotoinversion symmetry [e.g., bismuth^33^ and MoTe_2_^34^], (**d**) helical TCIs and HOTIs equivalent to four superposed AXIs with the same orbital hybridization^54^ and fourfold rotation or screw symmetry [e.g. SnTe^24,28^], and (**e**) helical TCIs and HOTIs equivalent to six superposed AXIs with the same orbital hybridization and sixfold rotation or screw symmetry. Through the double SIs calculated for this work (Table 2 and SN 31 and 32), we have discovered the existence of helical magnetic HOTIs with mirror-protected hinge states and bulk topology respectively enforced by the mirror and rotation symmetries of (**c**) double MPG 8.1.24 *mmm* [i.e., *D*_2*h*_, see ref. ^11^], (**d**) double MPG 15.1.53 4/*mmm* [*D*_4*h*_], and (**e**) double MPG 27.1.100 6/*mmm* [*D*_6*h*_], where we have labeled MPGs using the notation of the CorepresentationsPG tool (see SN 18). The magnetic HOTIs in (**c**–**e**) are respectively indicated by the minimal double SIs (**c**) *z*_4_ = 2 in double MSG 47.249 *Pmmm*, (**d**) *z*_8_ = 4 in double MSG 123.339 *P*4/*mmm*, and (**e**) *z*_12_ = 6 in double MSG 191.233 *P*6/*mmm* [as well as trivial values for all other independent minimal double SIs, see Table 2 and SN 33 for further details].

In order, this table contains the symbol of each double SI, the minimal double SSG(s) [i.e. the lowest-symmetry SSG(s) in which the double SI predicts nontrivial band topology, see SN 30 and 39], and the bulk topological phase(s) associated to nontrivial values of the double SI. All symmetry-indicated spinful SISM (specifically symmetry-indicated WSM), quantum anomalous Hall (QAH), TI, and TCI phases in magnetic and nonmagnetic crystalline solids necessarily exhibit nontrivial values of at least one of the double SIs listed in this table. We note that, in this table, the symbol AXI refers to both magnetic AXIs and ${{{{{{{\mathcal{T}}}}}}}}$-symmetric 3D TIs, because AXI and 3D TI phases are both defined by the nontrivial bulk axion angle *θ* = *π* [Fig. 5b and refs. ^21,55,63^]. Additionally, the symbols TCI and HOTI respectively indicate helical (i.e. non-axionic) mirror Chern insulators^24^ and HOTIs^26,28,29,31,32^, which include the magnetic HOTIs in Fig. 5(c–e) introduced in this work, as well as the nonmagnetic helical HOTI phases previously identified in bismuth^33^ and MoTe_2_^34^. Specific details of our SI calculations – including explicit SI formulas, TI and TCI layer constructions, tight-binding models, and the minimal double SSG associated to each double SSG – are provided in SN 26 and 39.

Using the subduction relations and layer constructions contained in SN 31, we have determined by direct computation that, for spinful bands in 3D crystals, all symmetry-indicated topological phases are either strong topological Weyl SISMs, AXIs, 3D TIs, helical TCIs or HOTIs, or can be deformed into weak stacks of 2D TIs, mirror TCIs, or Chern insulators with nonzero net Chern numbers in each unit cell [termed QAH states]. Curiously, we find that there are no Type-IV minimal double SSGs (SN 39). This implies that symmetry-indicated spinful SISM, TI, and TCI phases in Type-IV MSGs are actually protected by the symmetries of Type-I or Type-III double MSGs, as opposed to the symmetry $\{{{{{{{{\mathcal{T}}}}}}}}| {{{{{{{\bf{a}}}}}}}}/2\}$ common to Type-IV MSGs [though, as shown in Fig. 4c and in ref. ^58^, there exist topological SM phases unique to Type-IV MSGs]. For example, in ref. ^75^, the authors introduced ${{{{{{{\mathcal{I}}}}}}}}$-symmetric AFM TCIs in which *θ* = *π* was enforced by the symmetry $\{{{{{{{{\mathcal{T}}}}}}}}| {{{{{{{\bf{a}}}}}}}}/2\}$ common to all Type-IV MSGs. However, we have shown that the spinful, symmetry-indicated TCI phases in Type-IV MSGs can be subduced onto Type-I or Type-III MSGs without closing a gap or changing the bulk topology. Hence, the symmetry-indicated AFM TCIs introduced in ref. ^75^ can more simply be understood as ${{{{{{{\mathcal{I}}}}}}}}$-symmetry-enforced AXIs that remain topological when subduced onto the minimal Type-I double MSG 2.4 $P\bar{1}$. Through the layer constructions and double SI dependencies in SN 31 and 39, we have also demonstrated that all of the 3D symmetry-indicated spinful magnetic TCIs with odd numbers of chiral modes on crystal hinges (edges) in the 1,421 double MSGs exhibit the nontrivial axion angle *θ* = *π*, and are therefore AXIs^21,55,63^. Specifically, we find that all of the symmetry-indicated, spinful magnetic TCIs with chiral hinge states are AXIs in which *θ* = *π* is either quantized by ${{{{{{{\mathcal{I}}}}}}}}$, or by one of the rotoinversion symmetries ${C}_{4z}\times {{{{{{{\mathcal{I}}}}}}}}$ or ${C}_{6z}\times {{{{{{{\mathcal{I}}}}}}}}$ (see Table 2). This result is not necessarily intuitive – for example, when cut into a rod with the same point group symmetry as the bulk MSG, an ${{{{{{{\mathcal{I}}}}}}}}$-symmetric AXI in Type-I double MSG 2.4 $P\bar{1}$ exhibits two chiral hinge states, whereas a ${C}_{4z}\times {{{{{{{\mathcal{T}}}}}}}}$-symmetric AXI in Type-III double MSG 83.45 $P4^{\prime} /m$ exhibits four chiral hinge states; nevertheless, as shown in SN 31, both AXI phases exhibit *θ* = *π*. We additionally note that there do not exist symmetry-indicated, spinful magnetic TCIs with even numbers of intrinsic copropagating chiral hinge states (though magnetic TCIs with mirror symmetry may in principle exhibit copropagating chiral hinge modes, depending on the bulk mirror Chern numbers and boundary termination details).

Overall, across the 1,651 double SSGs, we find that there are only five families of 3D symmetry-indicated, spinful, strong topological phases [Fig. 5]: Weyl SISMs, AXIs and 3D TIs, and helical TCIs and HOTIs with twofold, fourfold, and sixfold symmetries. We note that helical TCIs and HOTIs in particular exhibit trivial axion angles *θ* mod 2*π* = 0, and are therefore non-axionic. In this work, we have discovered three novel variants of non-axionic magnetic HOTIs, which are shown in Fig. 5(c-e). Further details for the non-axionic HOTIs in Fig. 5(c-e), including symmetry-enhanced fermion doubling theorems^26,29^ and tight-binding models, are provided in SN 33. When cut into the finite nanorod geometries shown in Fig. 5(c-e), the non-axionic magnetic HOTIs exhibit helical, mirror-protected hinge states. We note that, if the mirror-symmetric HOTI hinges in Fig. 5(c-e) were sanded to expose mirror-symmetric 2D surfaces, each surface would exhibit two anomalous, mirror-protected, twofold Dirac cones, analogous to the mirror-protected helical hinge states of SnTe discussed in ref. ^28^. Lastly, we emphasize that the magnetic HOTIs in Fig. 5(c,e) exhibit the same nontrivial double SI *z*_4_ = 2 as ${{{{{{{\mathcal{T}}}}}}}}$-symmetric helical HOTI phases in supergroups of Type-II double SG 2.5 $P\bar{1}1^{\prime}$ (see Table 2 and refs. ^6,8,9,33,34^). Unlike for AXIs and 3D TIs^19–21,63^, the bulk response theories of helical HOTIs have not yet been elucidated. In light of recent experiments demonstrating incipient signatures of helical higher-order topology in bismuth crystals^33^ and MoTe_2_^76^, the absence of a response theory for helical HOTIs analogous to axion electrodynamics^21,63^ has become an urgent issue. The discovery in this work of helical magnetic HOTI phases whose bulk topology is solely enforced by the combination of unitary (spinful) mirror and rotation symmetries should provide crucial insight towards the elucidation of quantized response effects in helical HOTIs.

## Discussion

The theory of MTQC can also be applied to a wide variety of problems beyond the topological applications highlighted in this work. Most notably, while we have enumerated the spinful stable topological phases with nontrivial double SIs, the analogous enumeration of spinless magnetic SISMs and TCIs with nontrivial single SIs remains an open problem. In particular, whereas bosonic, symmetry-indicated AXI phases protected by ${{{{{{{\mathcal{I}}}}}}}}$ and SU(2) spin-rotation symmetry have been demonstrated in previous works^34,51^, it remains an open question whether there exist symmetry-indicated, non-axionic spinless (bosonic) TCIs. Additionally, while we have restricted consideration to single-particle topological phases, the magnetic (co)reps computed in this work can also be used to characterize correlated systems, including spin (-orbital) liquids^77^ and multipole tensor gauge theories^78^. For example, if a correlated magnetic insulator admits a mean-field slave-rotor description^79^, then the effective Hamiltonian of each quasiparticle species, such as spinon and chargeon on degrees of freedom^80^, can separately be analyzed with MTQC.

## Supplementary information

Supplementary Information

## Data Availability

The data supporting the findings of this study are available within the paper and through the BCS applications listed in Table 1. Additional information regarding the data generated for this study is available from the corresponding authors upon reasonable request.
