# Supplementary material for: Magnetic topological quantum chemistry
Source: Nat Commun. 2021 Oct 13;12:5965. doi: 10.1038/s41467-021-26241-8 (PMC8514474; doi:10.1038/s41467-021-26241-8)
Supplement: Supplementary file 1 — Supplementary Information [file 41467_2021_26241_MOESM1_ESM.pdf]

Supplementary Information for "Magnetic Topological Quantum Chemistry"

Contents

Supplementary Notes 2
1. Introduction to the Supplementary Notes 2
2. Introduction to the Magnetic Space Groups 3
3. Type-I SSGs: Ordinary (Fedorov) Groups (230 MSGs) 4
4. Type-II SSGs: Gray (Nonmagnetic) Groups (230 SSGs) 5
5. Type-III SSGs: Black and White Groups without Black and White Bravais Lattices (674 MSGs) 6
6. Type-IV SSGs: Black and White Groups with Black and White Bravais Lattices (517 MSGs) 7
7. Introduction to the Site-Symmetry Groups and Wyckoff Positions of the MSGs 8
8. Site-Symmetry Groups of the Magnetic Space Groups 8
9. Wyckoff Positions of the Magnetic Space Groups 10
10. Wyckoff Positions in Magnetic Subgroups of Type-II LG $p41'$ 12
11. Introduction to the Small Coreps of the Little Groups and Full Coreps of the MSGs 13
12. Little (Co)Groups, Momentum Stars, and the MKVEC Tool 14
13. Small and Full Coreps and the COREPRESENTATIONS Tool 20
14. Small and Full Coreps at the X and XA Points in Type-III MSG 75.3 $P4'$ 25
15. Small and Full Coreps at the S Point in Type-IV MSG 25.63 P_Cmm2 29
16. Compatibility Relations in the MSGs and the MCOMPREL Tool 33
17. Introduction to the Elementary Band Corepresentations of the MSGs (MEBRs) 41
18. Magnetic Atomic Orbitals and the COREPRESENTATIONSPG Tool 41
19. Irreps and Magnetic Atomic Orbitals in Type-I Single MPG 9.1.29 4 45
20. Coreps and Magnetic Atomic Orbitals in Type-III Single MPG 9.3.31 $4'$ 46
21. Coreps and Atomic Orbitals in Type-II Single SPG 9.2.30 $41'$ 47
22. Inducing Band Corepresentations from Magnetic Atomic Orbitals and the MSITESYM Tool 49
23. Computing the MEBRs and the Exceptional Cases and Introduction to the MBANDREP Tool 54
24. Exceptional Cases in the MSGs 55
25. Statistics for the MEBRs and the MBANDREP Tool 58
26. Introduction to Symmetry-Indicated Magnetic Topological Bands 61
27. Diagnosing Band Topology from Symmetry Eigenvalues 62
28. Symmetry-Based Indicator (SI) Groups and Formulas from the Smith Normal Form 63
29. Double SI Group and Formulas in Type-I Double MSG 3.1 $P2$ 65
30. Minimal Double SIs in the 1,651 Double SSGs 68
31. Double SI Formulas for Spinful Bands with Stable Topology in the 34 Minimal Double SSGs 70
    a. Double SIs in Type-I Double MSG 2.4 $P\bar{1}$ 72
    b. Double SIs in Type-I Double MSG 3.1 $P2$ 76
    c. Double SIs in Type-I Double MSG 10.42 $P2/m$ 76
    d. Double SIs in Type-I Double MSG 47.249 $Pmmm$ 77
    e. Double SIs in Type-I Double MSG 75.1 $P4$ 80
    f. Double SIs in Type-I Double MSG 77.13 $P4_2$ 81
    g. Double SIs in Type-I Double MSG 81.33 $P\bar{4}$ 82
    h. Double SIs in Type-I Double MSG 83.43 $P4/m$ 83
    i. Double SIs in Type-I Double MSG 84.51 $P4_2/m$ 85
    j. Double SIs in Type-I Double MSG 88.81 $I4_1/a$ 86
    k. Double SIs in Type-I Double MSG 123.339 $P4/mmm$ 88
    l. Double SIs in Type-I Double MSG 143.1 $P3$ 90
    m. Double SIs in Type-I Double MSG 147.13 $P\bar{3}$ 90
    n. Double SIs in Type-I Double MSG 168.109 $P6$ 91
    o. Double SIs in Type-I Double MSG 174.133 $P\bar{6}$ 92
    p. Double SIs in Type-I Double MSG 175.137 $P6/m$ 93
    q. Double SIs in Type-I Double MSG 176.143 $P6_3/m$ 94
    r. Double SIs in Type-I Double MSG 191.233 $P6/mmm$ 95
    s. Double SIs in Type-II Double SG 2.5 $P\bar{1}1'$ 97
    t. Double SIs in Type-II Double SG 83.44 $P4/m1'$ 97

u. Double SIs in Type-II Double SG 87.76 $I4/m1'$ 98
v. Double SIs in Type-II Double SG 175.138 $P6/m1'$ 98
w. Double SIs in Type-II Double SG 176.144 $P6_3/m1'$ 99
x. Double SIs in Type-III Double MSG 27.81 $Pc'c'2$ 99
y. Double SIs in Type-III Double MSG 41.215 $Ab'a'2$ 100
z. Double SIs in Type-III Double MSG 54.342 $Pc'c'a$ 102
aa. Double SIs in Type-III Double MSG 56.369 $Pc'c'n$ 102
bb. Double SIs in Type-III Double MSG 60.424 $Pb'cn'$ 103
cc. Double SIs in Type-III Double MSG 83.45 $P4'/m$ 103
dd. Double SIs in Type-III Double MSG 103.199 $P4c'c'$ 105
ee. Double SIs in Type-III Double MSG 110.249 $I4_1c'd'$ 106
ff. Double SIs in Type-III Double MSG 130.429 $P4/nc'c'$ 106
gg. Double SIs in Type-III Double MSG 135.487 $P4_2'/mbc'$ 107
hh. Double SIs in Type-III Double MSG 184.195 $P6c'c'$ 108
32. Summary of the Double SIs in the Minimal Double SSGs 109
33. Introduction to Non-Axionic Spinful Magnetic HOTIs 111
34. Symmetry-Enhanced Fermion Doubling Theorems for Non-Axionic Magnetic HOTIs 111
35. Tight-Binding Models and Boundary States for Non-Axionic Magnetic HOTIs 113
36. Guide to the Larger Supplementary Tables 123
37. Exceptional Composite Band Coreps Induced from Maximal Site-Symmetry Groups 123
38. Maximum and Minimum Dimensions of the Single- and Double-Valued MEBRs 157
39. Minimal SSG Dependencies for the Double SIs in the 1,651 Double SSGs 169

Supplementary References 175

Supplementary Notes

1. Introduction to the Supplementary Notes

In this supplement, we provide proofs and tables that extend Topological Quantum Chemistry (TQC)[1–6] to the magnetic space groups (MSGs), to develop a complete theory of Magnetic Topological Quantum Chemistry (MTQC). MTQC provides, for the first time, a predictive, position-space formulation of the characteristics of band structures – including stable and fragile topology – in all translationally invariant crystalline solids that are characterized by mean-field theory with a static background magnetic field. Most relevant to the physical systems studied in this work, MTQC provides tools for characterizing the symmetry and topology of electronic states in solid-state materials with lattice-commensurate magnetism. We begin in Supplementary Note (SN) 2 by precisely defining the MSGs, drawing connection where possible to the more familiar nonmagnetic space groups (SGs). We then discuss in SN 8 the Wyckoff positions of the MSGs, whose sites are left invariant under the symmetries of site-symmetry groups that are necessarily isomorphic to crystallographic magnetic point groups (MPGs)[7–19]. Next, in SN 11, we introduce crystal momentum $\mathbf{k}$ in the MSGs, and discuss how spatial and magnetic symmetries are represented in momentum space. To enumerate the set of symmetry-independent $\mathbf{k}$ points in each MSG and SG, we have implemented the MKVEC tool (further detailed in SN 12), which is now available on the Bilbao Crystallographic Server (BCS)[13,14]. In SN 13, we then describe how, in this work, we have for the first time derived the complete set of irreducible [small] little group and full [space group] (co)representations [(co)reps] of the MSGs, which can now be accessed on the BCS[13,14] through the Corepresentations tool [further detailed in SN 14 and 15]. Lastly, by combining the results of SN 12 and 13, we then in SN 16 derive the compatibility relations between small (co)reps in the MSGs, which we have made accessible through the MCOMPREL tool on the BCS.

Having established position- and momentum-space characterizations of the MSGs, we then in SN 17 complete the theory of MTQC by enumerating the magnetic elementary band (co)representations [MEBRs][1–6,11,12,20–22], which represent all possible [magnetic] trivial atomic limits. To obtain the MEBRs, we first in SN 18 introduce the minimal magnetic atomic orbitals [*e.g.* $p_x + ip_y$] that correspond to the (co)reps of the magnetic site-symmetry groups, which are isomorphic to MPGs. In SN 18, we additionally detail the CorepresentationsPG tool on the BCS, which we have implemented for this work to provide access to the (co)reps of the magnetic site-symmetry groups of the MSGs. Next, in SN 22, we establish the central machinery of MTQC through which band (co)representations [band (co)reps] in momentum space are induced from site-symmetry (co)reps in position space. We also introduce and detail in SN 18 the MSITESYM tool, through which users may access the small (co)reps subduced from each band (co)rep of each SSG. Finally, in SN 23, we complete the derivation of MTQC by enumerating the MEBRs. In SN 25, we additionally

detail the MBANDREP tool on the BCS, which we have developed for this work to compute and display both the elementary and non-elementary [*i.e.* composite] band (co)reps of the MSGs.

The theory of MTQC uniquely enables us to, for the first time, enumerate all of the symmetry-based indicators of band topology (SIs)[22–31] [*i.e.* generalized Fu-Kane-like symmetry-eigenvalue topological indices[32]] for the double-valued (co)reps of the 1,651 spinful [double] magnetic and nonmagnetic space groups [SSGs]. Specifically, a (co)rep is respectively defined as single- or double-valued if the matrix representatives of time-reversal and rotation symmetries in the (co)rep square to plus or minus the identity[33]. Double groups have both single- and double-valued (co)reps, whereas single groups only have single-valued (co)reps. Electronic [fermionic] states in solid-state materials are generically characterized by double-valued (co)reps of double symmetry groups, though in the absence of spin-dependent interactions [*e.g.* spin-orbit coupling (SOC)], spin-degenerate electronic states may be labeled with single-valued (co)reps. In SN 26, we compute the SI groups and formulas for all symmetry-indicated, spinful, mean-field topological phases in the 1,651 double SSGs. We specifically demonstrate in SN 30 how the SI calculation can be reduced by recognizing that the SIs in all 1,651 double SSGs are dependent on the SIs in a considerably smaller subset of minimal double SSGs. Through the minimal SI calculation, which is provided in explicit detail in SN 31, we discover several novel, helical, magnetic higher-order topological crystalline insulators (HOTIs)[24–28,34–39] whose bulk response theories do not correspond to axion electrodynamics[28,38,40–60]. The *non-axionic* magnetic HOTIs discovered in this work are further detailed in SN 33. Lastly, in SN 36, we provide larger supplementary tables containing additional data generated for this work.

2. Introduction to the Magnetic Space Groups

In this section, we list the basic group theoretic properties of the magnetic space groups (MSGs). To begin, it was established in Supplementary References (SRefs.) 61,62 (and translated into English in SRef. 63) that the Hamiltonians of 3D, periodic systems (*i.e.* crystalline solids) without particle-hole symmetry are invariant under the symmetries contained in at least one of the 1,651 Shubnikov space groups (SSGs). All of the SSGs contain the group of fundamental lattice translations:

$$G_T = T_a \otimes T_b \otimes T_c, \tag{1}$$

where T_i is the group comprised of the set of lattice translations t_i^n, where $n \in \mathbb{Z}$ and:

$$t_i = \{E|\mathbf{t}_i\}, \tag{2}$$

where E is the identity operation. Throughout this work, we will employ a notation [Supplementary Equation (SEq.) (2)] in which t_i is the symmetry operation of a translation by the vector $\mathbf{t}_i$. In SEq. (1), the generating translations $t_{a,b,c}$ must be linearly independent, but are not necessarily orthogonal (though $t_{a,b,c}$ are indeed both linearly independent and orthogonal in many SSGs).

The 1,651 SSGs subdivide into four types, which are distinguished by their antiunitary symmetries[33,61–64]. Of the four types of SSGs, the 1,421 Types-I, III, and IV SSGs characterize magnetic crystals (*i.e.* crystals with lattice-commensurate magnetic order); hence, in this work, we interchangeably denote Type-I, III, and IV SSGs as MSGs or SSGs. Conversely, the 230 Type-II SSGs exclusively characterize nonmagnetic crystals; hence, in this work, we interchangeably refer to Type-II SSGs as SGs or SSGs. Unlike in other recent works on magnetic symmetry and topology[65,66], we will not refer to the 230 Type-II groups as MSGs, to avoid employing terminology in which the Type-II SSGs are "nonmagnetic magnetic SGs." All SSGs (MSGs and SGs) are given in the notation established in SRef. 67 and reproduced on the Bilbao Crystallographic Server (BCS)[13,14]. Because the set of possible G_T in SEq. (1) coincides with the 14 3D nonmagnetic (gray) Bravais lattices, then all 1,651 SSGs can be characterized by the 14 Bravais lattices. However, as we will detail in SN 6, the Type-IV groups – which contain elements of the form $\mathcal{T}(t_i/2)$ where $\mathcal{T}$ is the operation of time-reversal – are also frequently characterized using the 22 "black and white" Bravais lattices that account for the relative positions of localized spins (or classical magnetic moments) [*c.f.* Chapter 7 in SRef. 33]. In this work, we will refer to all 1,651 SGs by their nonmagnetic (gray) Bravais lattice (*i.e.*, the Bravais lattice of their primitive, or "magnetic" unit cell). This choice of Bravais lattice is naturally incorporated into the numbering and notation of Belov, Nerenova, and Smirnova[67] (labeled as the "BNS setting" on the BCS), which we will employ throughout this work. For generality and connection with other works, we also note that on the BCS, information about the SSGs can alternatively be obtained in the convention of Opechowski and Guccione[68] (labeled as the "OG setting" on the BCS); we will not employ, or further discuss, the OG setting in this work.

It is important to highlight the distinction between MSGs and phenomenological descriptions of magnetic order. Specifically, while all magnetic crystals with Type-IV MSGs are antiferromagnets (see SN 6), there are both ferromagnets and antiferromagnets with Type-I or Type-III MSGs[33] (SN 3 and 5, respectively). For each of the three types of

MSGs, we will below provide representative examples of quasi-1D chains with symmetry-allowed magnetic ordering, including phenomenologically distinct magnetic order in crystals with the same Type-I or Type-III MSG (see SN 3 and 5, respectively). Each of the quasi-1D chains shown below is invariant under a crystallographic magnetic rod group (MRG)[10,33,69–71] M_{RG}, *i.e.* a subperiodic group with 3D symmetry operations and 1D translations. Each MRG is isomorphic to an SSG M under the addition of in-plane lattice translations, where the group-subgroup relations between M_{RG} and M depend on the details of the additional translations. For example, when translations in the xy-plane are added to Type-I MRG $(p4_2cm)_{RG}$, the resulting MSG is either Type-I MSG 101.179 $P4_2cm$ or Type-I MSG 105.211 $P4_2mc$, depending on whether the shortest lattice translations are respectively added in the x and y or $x \pm y$ directions[69,70]. In this work, we will refer to quasi-1D chains and rods using the terminology established in SRefs. 28,38,71 in which a chain or rod with the translation symmetry $t_c = \{E|c\}$ is termed *c-directed*. The symbols for the MRGs referenced in this work are given in the convention employed by Litvin in SRef. 10.

3. Type-I SSGs: Ordinary (Fedorov) Groups (230 MSGs)

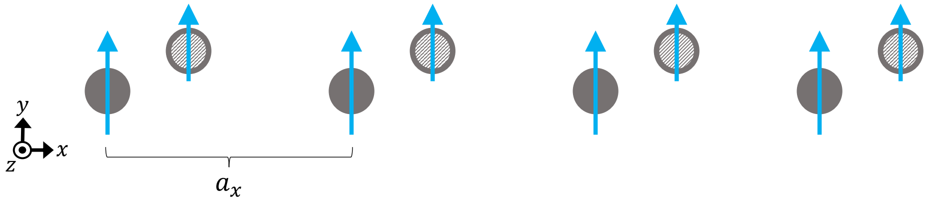

Supplementary Figure 1: A ferromagnetic chain with MRG $(p1)_{RG}$, which is generated by $\{E|1\}$ (t_x) where E is the identity operation, and is isomorphic after the addition of perpendicular lattice translations (*e.g.* t_y and t_z) to Type-I MSG 1.1 $P1$. There are two atoms within each unit cell, where the right-most atom in each cell (hashed circle) exhibits a weaker y-directed magnetic moment than the left-most atom (solid circle), lies away from $x = a_x/2$, and is displaced from the xy-plane ($z \neq 0$ for the hashed atoms). If there was just one atom in each unit cell, if the solid and hashed atoms were moved to be coplanar, or if the magnetic moments were tuned to be the same magnitude, then the chain would respect additional symmetries, such as $\{m_z \times \mathcal{T}|0\}$.

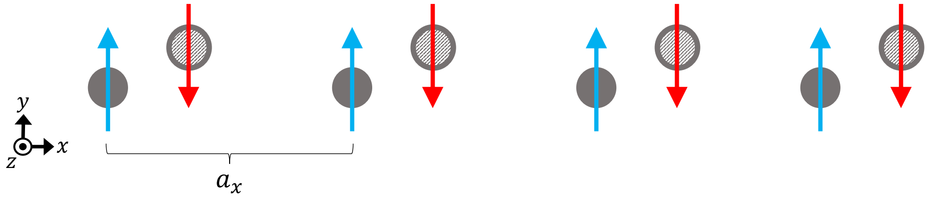

Supplementary Figure 2: An antiferromagnetic chain with MRG $(p1)_{RG}$, which is generated by $\{E|1\}$, and is isomorphic after the addition of perpendicular lattice translations to Type-I MSG 1.1 $P1$. The solid and hashed circles represent magnetic atoms with distinct chemical environments [*e.g.* atoms of the same species with different oxidation states or on-site (chemical) potentials] and equal and opposite magnetic moments. The right-most atom in each cell (*i.e.* the hashed atom with a red magnetic moment) lies away from $x = a_x/2$ and $z = 0$, such that the solid and hashed atoms are neither equally spaced nor coplanar. If the chemical environments of the solid and hashed atoms were tuned to be equivalent, if the solid and hashed atoms were moved to be coplanar, or if the atoms were shifted to be separated by a distance $a_x/2$ in the x-direction, then the chain would have additional symmetries. For example, if the local chemical environment (*i.e.* hoppings and on-site potentials) of the solid and hashed atoms were made equivalent, then the chain would respect $\{m_z \times \mathcal{T}|0\}$ symmetry (as well as additional symmetries), and if the atoms tuned to lie in equivalent chemical environments and shifted to be equally spaced and coplanar, then the chain would respect both $\{C_{2z}|0\}$ and $\{m_y \times \mathcal{T}|1/2\}$ symmetry (as well as additional symmetries).

Each Type-I SSG M_I is exclusively characterized by a set of unitary symmetry operations. The simplest Type-I SSG – MSG 1.1 $P1$ – is isomorphic to G_T [SEq. (1)], and is a common subgroup of all 1,651 SSGs. The Type-I MSGs have historically been termed the *ordinary groups*[33], because Type-I magnetic symmetry groups do not contain antiunitary symmetries that relate classical magnetic moments at different positions in a crystal. Type-I MSGs can characterize a variety of magnetic configurations[33]. For example, Type-I MSG 1.1 $P1$ can characterize crystals with either ferromagnetism [Supplementary Figure (SFig.) 1] or antiferromagnetism (SFig. 2).

4. Type-II SSGs: Gray (Nonmagnetic) Groups (230 SSGs)

Each Type-II SSG M_{II} takes the form:

$$M_{II} = G \cup \{\mathcal{T}|000\}G = G \cup \mathcal{T}G, \tag{3}$$

where G is isomorphic to a Type-I SSG. Because each Type-II SSG contains the element $\{\mathcal{T}|000\}$, then no position in the unit cell of a crystal with a Type-II SSG can host a local magnetic moment. Therefore, crystals invariant under Type-II SSGs are necessarily $\mathcal{T}$-symmetric. The Type-II MSGs have historically been termed the *gray groups*[33], because Type-II groups do not admit the presence of localized magnetic moments, due to $\{\mathcal{T}|000\}$ symmetry at each point in each unit cell. Unlike the symbols of the Type-I SSGs, the symbols of Type-II, III, and IV SSGs contain primes, which denote antiunitary group elements. Because we are discussing both MSGs and (nonmagnetic) SGs in this work, we will employ the notation of SRef. 10 in which the symbols of $\mathcal{T}$-symmetric groups M_{II} are followed by $1'$ to emphasize that $\{\mathcal{T}|000\} \in M_{II}$. For example, in this work, the symbol $P4/mmm$ refers to Type-I MSG 123.339, whereas the symbol $P4/mmm1'$ refers to Type-II SSG 123.340 (which is frequently denoted in other works[1–6] using only the simplified expression "space group 123 $P4/mmm$").

Given a group G and a subgroup H of G, we will find it useful to define the *index* of H in G. Here and throughout this work, we will use cosets to precisely define the group-subgroup index. Specifically, given a group G and a subgroup H, we can define the coset of H represented by an element $g \in G$ as:

$$gH \equiv \{gh|h \in H\}. \tag{4}$$

By construction, SEq. (4) implies that every element $g \in G$ is in one (and only one) coset gH. By definition, G may be decomposed into cosets with respect to H by the *set difference* $G \setminus H$:

$$G = H \cup g_1H \cup g_2H \cup ..., \tag{5}$$

such that:

$$G \setminus H = \{g|g \in G, g \notin H\} = g_1H \cup g_2H \cup ..., \tag{6}$$

where g_iH are (unique) cosets of H defined by $g_iH \neq g_jH$ for $g_{i,j} \in G,\ g_{i,j} \notin H$. If G and H are groups, $E \in G$ and $E \in H$ where E is the identity element, implying that $G \setminus H$ is *not* a group, because $E \notin G \setminus H$. Similarly, there does not exist a case in which $g_i = E$ in SEq. (6), as this would imply that $G \setminus H = H$. We emphasize that the choice of each g_i in SEq. (6) is not unique; there are generically multiple, equivalent ways of expressing the decomposition of $G \setminus H$ into cosets of H. SEq. (6) implies that:

$$G = H \cup (G \setminus H) = H \cup g_1H \cup g_2H \cup ..., \tag{7}$$

from which we define the quotient:

$$G/H = \{H, g_1H, g_2H, ...\}. \tag{8}$$

We briefly pause to note that, if H is additionally a *normal* subgroup of G, such that $gH = Hg$, then we can define a group operation on cosets:

$$g_1Hg_2H = g_1g_2H. \tag{9}$$

Finally, using SEq. (8), we establish the definition of the index $[G : H]$ of the subgroup H of G as:

$$[G : H] = |G/H| = |G|/|H|, \tag{10}$$

where $|G|$, $|H|$, and $|G/H|$ are respectively the number of elements in G, H, and G/H [equal to one plus the number of coset representatives g_i in SEq. (6)]. It is important to note that $|G|$ ($|H|$) in SEq. (10) is necessarily infinite if G ($|H|$) is an infinite group. However, if G and H are *both* infinite, then the index $[G : H] = |G|/|H|$ may still be finite.

It is worth noting that all 1,421 MSGs are index-2 subgroups of 230 Type-II SSGs. For the previous Type-I groups in SN 3, this follows directly from SEq. (3), and for the Type-III and Type-IV groups, this will respectively be proved in SN 5 and 6.

5. Type-III SSGs: Black and White Groups without Black and White Bravais Lattices (674 MSGs)

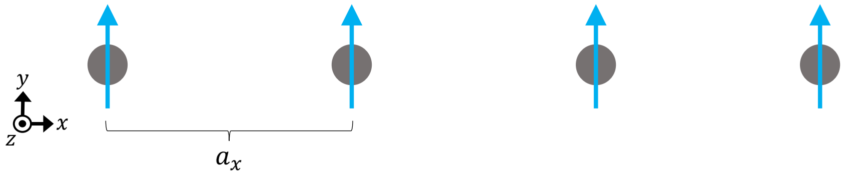

Supplementary Figure 3: A ferromagnetic chain with MRG $(pm'mm')_{RG}$, which is generated by $\{E|1\}$, $\{C_{2x}\times\mathcal{T}|0\}$, $\{C_{2y}|0\}$, and $\{\mathcal{I}|0\}$, and is isomorphic after the addition of perpendicular lattice translations to Type-III MSG 47.252 $Pm'm'm$. The primes in the symbol $(pm'mm')_{RG}$ indicate that the MRG contains the symmetries $\{m_x\times\mathcal{T}|0\}$ and $\{m_z\times\mathcal{T}|0\}$. In the decomposition in SEq. (11), $M_{III}=(pm'mm')_{RG}$, $G=(pmmm)_{RG}$ [isomorphic to Type-I MSG 47.249 $Pmmm$ after the addition of perpendicular lattice translations], and $H=(p12/m1)_{RG}$ [isomorphic to Type-I MSG 10.42 $P2/m$ after the addition of perpendicular lattice translations].

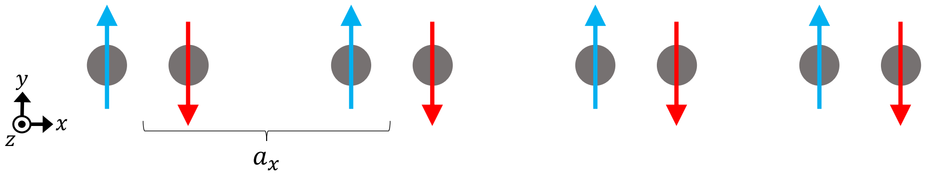

Supplementary Figure 4: An antiferromagnetic chain with MRG $(pmmm')_{RG}$, which is generated by $\{E|1\}$, $\{C_{2x}\times\mathcal{T}|0\}$, $\{C_{2y}\times\mathcal{T}|0\}$, and $\{\mathcal{I}\times\mathcal{T}|0\}$, and is isomorphic after the addition of perpendicular lattice translations to Type-III MSG 47.251 $Pm'mm$. The prime in the symbol $(pmmm')_{RG}$ indicates that the MRG contains $\{m_z\times\mathcal{T}|0\}$ symmetry. The red and blue magnetic moments are equal in magnitude and opposite in direction, and are related by the operation of $\{C_{2z}|0\}$ about the midpoints between adjacent atoms. In the decomposition in SEq. (11), $M_{III}=(pmmm')_{RG}$, $G=(pmmm)_{RG}$ [isomorphic to Type-I MSG 47.249 $Pmmm$ after the addition of perpendicular lattice translations], and $H=(pmm2)_{RG}$ [isomorphic to Type-I MSG 25.57 $Pmm2$ after the addition of perpendicular lattice translations].

Each Type-III SSG M_{III} takes the form:

$$M_{III}=H\cup\mathcal{T}(G\setminus H), \tag{11}$$

where G and H are isomorphic to Type-I SSGs, $H\subset G$, and $G\setminus H$ is a set that contains no elements of the form $\{E|\mathbf{t}\}$, where E is the identity operation and $\mathbf{t}$ is a translation. Hence, $G\setminus H$ in SEq. (11) does not include the identity element $\{E|\mathbf{0}\}$, though $G\setminus H$ is free to contain elements of the form $\{f|\mathbf{0}\}$ where f is a unitary rotation or rotoinversion. Because $G\setminus H$ does not contain pure translations, then it follows that H is a subgroup of G with the same Bravias lattice. Following arguments recently presented in SRef. 72, we will demonstrate that H is an index-2 subgroup of G. To establish that $[G:H]=2$, we will first show that H is an index-2 subgroup of M_{III}. We begin by noting that, given an antiunitary symmetry $g_A\in\mathcal{T}(G\setminus H)$:

$$g_A=\mathcal{T}\times g, \tag{12}$$

where g is a unitary symmetry $g\in G$, $g\notin H$. Hence, g_A^2 is a unitary symmetry operation $g_A^2\in M_{III}$, implying that:

$$g_A^2\in H,\ g_A^2\notin\mathcal{T}(G\setminus H). \tag{13}$$

SEqs. (11), (12), and (13) imply that:

$$M_{III}=g_AM_{III}=g_AH\cup g_A\mathcal{T}(G\setminus H), \tag{14}$$

such that:

$$\mathcal{T}(G\setminus H)=g_AH, \tag{15}$$

implying that H is an index-2 subgroup of M_{III}. SEq. (14) also implies through SEqs. (11) and (12) that:

$$|H| = |\mathcal{T}(G \setminus H)| = |G \setminus H|, \tag{16}$$

establishing that H is also an index-2 subgroup of G:

$$G = H \cup gH, \tag{17}$$

such that $gH = G \setminus H$, consistent with SEqs. (12) and (15). Finally, to see that M_{III} is an index-2 subgroup of a Type-II SSG (specifically $M_{II} = G \cup \mathcal{T}G$), we consider the effects of restoring $\mathcal{T}$ symmetry to SEq. (11):

$$\begin{aligned} M_{III} \cup \mathcal{T}M_{III} &= H \cup \mathcal{T}(G \setminus H) \cup \mathcal{T}H \cup (G \setminus H) \\ &= G \cup \mathcal{T}G \\ &= M_{II}. \end{aligned} \tag{18}$$

Like the previous Type-I MSGs in SN 3, Type-III MSGs can characterize both ferromagnetic (SFig. 3) and antiferromagnetic (SFig. 4) crystals. The symbols for Type-III MSGs contain primes that denote which symmetry operations are formed from the combination of $\mathcal{T}$ and a unitary element of $G \setminus H$ [SEq. (11)]. The Type-III MSGs have historically been termed the *black and white groups without black and white Bravais lattices*, because Type-III groups contain antiunitary symmetries that relate classical magnetic moments at different positions in a crystal, but do not contain the antiferromagnetic translation symmetry $t_0\mathcal{T}$ common to Type-IV MSGs that generates the black and white Bravais lattices (see SN 6 and Chapter 7 in SRef. 33.) Representative examples demonstrating the usage of primes in Type-III magnetic group symbols are presented in SFigs. 3 and 4.

6. Type-IV SSGs: Black and White Groups with Black and White Bravais Lattices (517 MSGs)

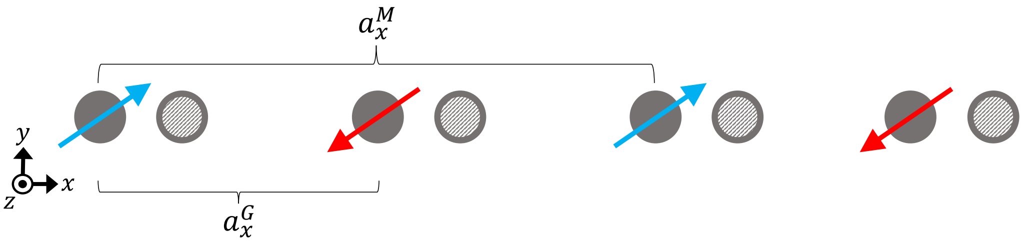

Supplementary Figure 5: An antiferromagnetic chain with MRG $(p_a1)_{RG}$, which is generated by $\{\mathcal{T}|1/2\}$ $(t_{a_x^M/2}\mathcal{T})$, and is isomorphic after the addition of perpendicular lattice translations to Type-IV MSG 1.3 P_S1. The red and blue magnetic moments on the atoms labeled with solid circles are equal in magnitude and opposite in direction. The two nonmagnetic atoms (hashed circles) in each magnetic unit cell are displaced out of the xy-plane, breaking additional symmetries such as $\{m_z \times \mathcal{T}|0\}$. In terms of the black and white Bravais lattices historically employed to characterize (antiferro)magnetic structures[33], the atoms with blue magnetic moments can be taken to occupy white sites, whereas the atoms with red (time-reversed) magnetic moments can be taken to occupy black sites. Throughout this work, we will only use the more familiar gray (nonmagnetic) Bravais lattices to characterize magnetic symmetry groups, because the black and white Bravais lattices add an additional level of complexity that does not factor into any of the group-theoretic calculations that comprise MTQC. Further discussion and a complete enumeration of the black and white Bravais lattices is provided in Chapter 7 in SRef. 33. In the antiferromagnetic chain in this figure, the blue and red magnetic moments are related by $t_{a_x^M/2}\mathcal{T}$. The primitive (magnetic) unit cell of the spin chain has a length a_x^M, whereas the nonmagnetic unit cell, which is realized by restoring $\mathcal{T}$ symmetry [SEq. (22)], has a shorter length $a_x^G = a_x^M/2$. In the decomposition in SEq. (19), $M_{IV} = (p_a1)_{RG}$, $H = (p1)_{RG}$ with the lattice constant $a = a_x^M$ [isomorphic to Type-I MSG 1.1 $P1$ after the addition of perpendicular lattice translations], and $t_0 = t_{a_x^M/2}$. From this, we establish the decomposition in SEqs. (20) and (21), in which $M_{IV} = (p_a1)_{RG}$, $G = (p1)_{RG}$ with the lattice constant $a = a_x^G = a_x^M/2$, and $H = (p1)_{RG}$ with the lattice constant $a = a_x^M$.

Each Type-IV SSG M_{IV} takes the form:

$$M_{IV} = H \cup \mathcal{T}t_0H, \tag{19}$$

in which H is isomorphic to a Type-I SSG and t_0 is a translation whose length is half that of either $t_{a,b,c}$, $t_a + t_b$, $t_a + t_c$, $t_b + t_c$, or $t_a + t_b + t_c$, where $t_{a,b,c}$ are the primitive lattice translations in H[33]. The fractional lattice translations

t_0^n where n mod $2 = 1$ relate positions of alternating color (classical spin orientation) in the black and white Bravais lattice of M_{IV} (see Chapter 7 in SRef. 33), whereas the full lattice translations $t_{a,b,c}$ relate positions with the same color in the nonmagnetic (gray) Bravais lattice of M_{IV}. Hence, historically[33], the Type-IV MSGs have been termed the *black and white groups with black and white Bravais lattices*. As previously with the Type-III groups [see the text surrounding SEq. (12)], we can show that H is an index-2 subgroup of M_{IV}. To demonstrate that $[M_{IV} : H] = 2$, we first rearrange SEq. (19) into the same form as SEq. (11):

$$M_{IV} = H \cup \mathcal{T}(G \setminus H), \tag{20}$$

for which, by construction:

$$G = H \cup t_0 H, \tag{21}$$

such that G is isomorphic to a Type-I SSG with the gray Bravais lattice given by ignoring the colors of the black and white Bravais lattice of M_{IV} (see SFig. 5). To show that M_{IV} is an index-2 subgroup of a Type-II group, we again restore $\mathcal{T}$ symmetry [see SEq. (18)]:

$$\begin{aligned} M_{IV} \cup \mathcal{T} M_{IV} &= H \cup \mathcal{T} t_0 H \cup \mathcal{T} H \cup t_0 H \\ &= G \cup \mathcal{T} G \\ &= M_{II}, \end{aligned} \tag{22}$$

where G is given in SEq. (21). As shown in the text following SEq. (10), SEq. (21) also implies that H is an index-2 subgroup of the Type-I MSG G.

Physically, SEq. (22) implies that the process of "turning off" the magnetism in a crystal with a Type-IV SSG (MSG) M_{IV} generates a nonmagnetic crystal that is invariant under a Type-II group M_{II} with a smaller unit cell than the magnetic unit cell of M_{IV} (SFig. 5), and with the same gray Bravais lattice as G in SEq. (21) (as opposed to the gray Bravais lattice of H). Unlike the previous Type-I and Type-III MSGs in SN 3 and 5, respectively, Type-IV MSGs necessarily characterize crystals with net-zero magnetic moments, because the operation of $t_0\mathcal{T} \in M_{IV}$ [SEq. (19)] relates the spin configuration of one half of the primitive (magnetic) unit cell to its time-reverse in the other half. The symbols for Type-IV MSGs contain subscripts that denote the direction of t_0, and therefore specify the gray (nonmagnetic) Bravais lattice of G in SEqs. (21) and (22).

7. Introduction to the Site-Symmetry Groups and Wyckoff Positions of the MSGs

Next, we will discuss the position-space action of the symmetries of the MSGs. In SN 8, we will introduce the site-symmetry (stabilizer) groups of the MSGs, and in SN 9, we will discuss how the Wyckoff positions of the MSGs are related to those of the $\mathcal{T}$-symmetric SGs.

Throughout the text below, we will provide representative 2D atomic and spin configurations highlighting properties of the site-symmetry groups and Wyckoff positions of the MSGs. The 2D magnetic structures shown in this section each respect the symmetries of a magnetic layer group (MLG) M_{LG} – a subperiodic group with 3D symmetry operations and 2D translations[10,39,69,70,73,74]. Each MLG is isomorphic to (at least one) MSG M modulo out-of-plane lattice translations. Specifically, taking the in-plane translations to be elements of $T_{x,y}$ ($T_{a,b}$), and taking $t_c \in T_c$ (T_z) to be a lattice translation in the z (out-of-plane) direction:

$$M = M_{LG} \cup t_c M_{LG}. \tag{23}$$

In this work, the symbols of MLGs are given in the convention employed by Litvin in SRef. 10.

8. Site-Symmetry Groups of the Magnetic Space Groups

In this section, we will define the *site-symmetry group* $M_{\mathbf{q}}$ at a point $\mathbf{q}$ in crystal that is invariant under an SSG M. To begin, M is composed of unitary symmetry operations:

$$g_{U,i} = \{h_i|\mathbf{t}_i\}, \tag{24}$$

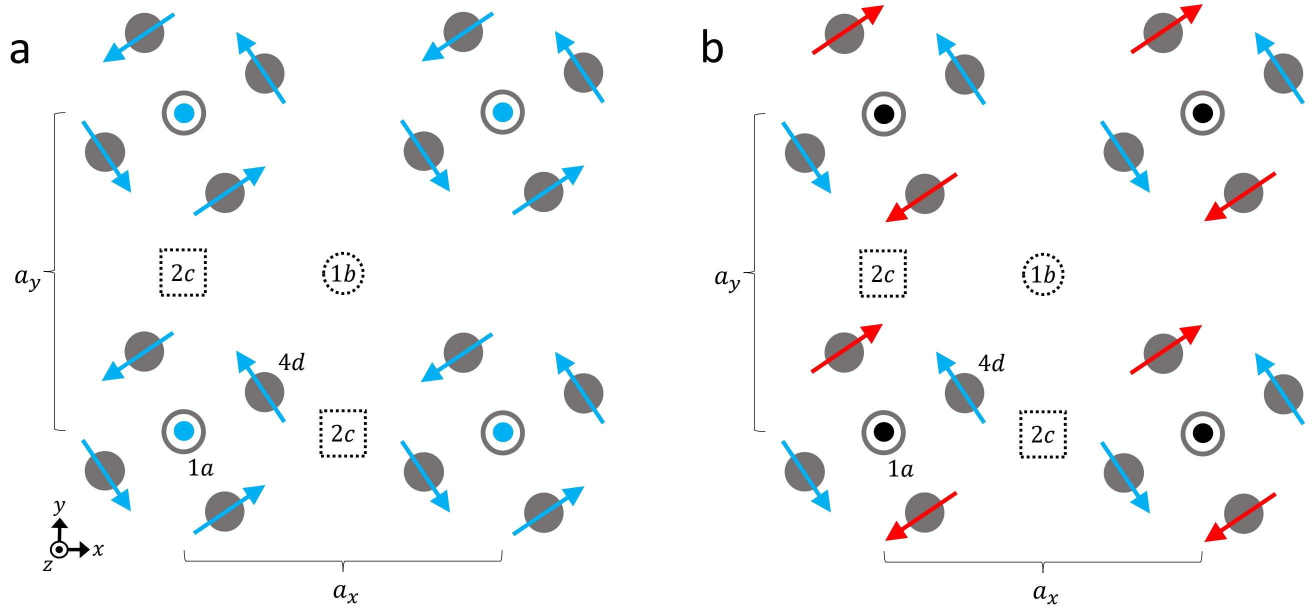

Supplementary Figure 6: (a) A magnetic crystal with Type-I magnetic layer group (MLG)[10,39,69,70,73,74] $p4$, which is generated by $\{E|10\}$ and $\{C_{4z}|00\}$, and is isomorphic after the addition of t_z to Type-I MSG 75.1 $P4$. The atoms on the $1a$ sites in (a) exhibit an additional magnetic moment in the $+\hat{z}$ direction (blue dot), which we have chosen in order to break $\{m_z \times \mathcal{T}|00\}$ symmetry[74] to simplify the symmetry analysis performed in this section. (b) A magnetic crystal with Type-III MLG $p4'$, which is generated by $\{E|10\}$ and $\{C_{4z} \times \mathcal{T}|00\}$, and is isomorphic after the addition of t_z to Type-III MSG 75.3 $P4'$. The red magnetic moments in (b) have the same magnitudes as the blue magnetic moments; they are only colored in red to emphasize that the red moments in (b) are related to the blue moments by the antiunitary symmetry operation ($\{C_{4z} \times \mathcal{T}|00\}$). The atoms on the $1a$ sites in (b) do not exhibit a magnetic moment, and instead are displaced out of the xy-plane in the $+\hat{z}$ direction, which we have indicated with black dots. We have chosen to displace the atoms at the $1a$ position in each unit cell in (b) out of the xy-plane in order to break $\{m_z|00\}$ symmetry, such that the MLGs in (a) and (b) share the same "unprimed" Type-I MLG $G = p4$ [see the text surrounding SEq. (34)]. The $1a$ and $1b$ site-symmetry groups in (a) are isomorphic to Type-I MPG 9.1.29 4, which is generated by C_{4z}, whereas the $1a$ and $1b$ site-symmetry groups in (b) are isomorphic to Type-III MPG 9.3.31 $4'$, which is generated by $C_{4z} \times \mathcal{T}$. Nevertheless, in both (a) and (b), the $2c$ site-symmetry groups are isomorphic to Type-I MPG 3.1.6 2, which is generated by C_{2z}. In both (a) and (b), magnetic moments additionally occupy the $4d$ (general) position, where the site-symmetry groups at $4d$ in both (a) and (b) are isomorphic to Type-I MPG 1.1.1 1, the trivial MPG. The MLGs in (a) and (b) [$p4$ and $p4'$, respectively] are also isomorphic to magnetic wallpaper groups[39,73–75].

and antiunitary symmetry operations:

$$g_{A,j} = \{h_j \times \mathcal{T}|\mathbf{t}_j\}, \tag{25}$$

where each $h_{i,j}$ is a unitary symmetry operation that is either the identity, a rotation, or a rotoinversion. Given a point $\mathbf{q}$ in an infinite crystal, the action of $g_{U,i}$ and $g_{A,j}$ on $\mathbf{q}$ is given by:

$$g_{U,i}\mathbf{q} = h_i\mathbf{q} + \mathbf{t}_i,\ g_{A,j}\mathbf{q} = h_j\mathbf{q} + \mathbf{t}_j, \tag{26}$$

in which only $h_{i,j}$ and $\mathbf{t}_{i,j}$ act on $\mathbf{q}$, because $\mathcal{T}$, by definition, leaves spatial coordinates invariant[33]. As defined in SRef. 1, a site-symmetry group $M_\mathbf{q}$ is spanned by the set of unitary and antiunitary symmetry operations $g \in M$ that return a site $\mathbf{q}$ in an infinite crystal (*i.e.* a point in position space) to itself *in the same unit cell*:

$$g\mathbf{q} = \mathbf{q}, \tag{27}$$

for all $g \in M_\mathbf{q}$. Hence, the site-symmetry group $M_\mathbf{q}$ of $\mathbf{q}$ is finite a subgroup of the SSG M:

$$M_\mathbf{q} \subset M, \tag{28}$$

in which $M_\mathbf{q}$ does not contain elements of the form $\{E|\mathbf{t}\}$ or $\{\mathcal{T}|\mathbf{t}\}$, where E is the identity operation and $\mathbf{t}$ is a translation. Later, in SN 9, we will reintroduce the Wyckoff positions of M containing $\mathbf{q}$, as defined in SRef. 2.

In SEq. (28), $M_{\mathbf{q}}$ is necessarily isomorphic to one of the 122 crystallographic Shubnikov point groups (SPGs)[7–18], which are listed in the MPOINT (http://www.cryst.ehu.es/cryst/mpoint.html)[15–18] and CorepresentationsPG tools on the BCS, in which the SPGs are numbered according to the convention established by Litvin in SRef. 10. The SPGs divide into 32 Type-I magnetic point groups (MPGs), 32 Type-II (nonmagnetic) SPGs, and 58 Type-III MPGs, where the type of an SPG is defined the same way as the type of an SSG [SN 3 and SEqs. (3) and (11), and (11)]. We emphasize that, unlike in the MSGs, which subdivide into Types-I, III, and IV, there are only Type-I and Type-III MPGs. Specifically, there are no Type-IV MPGs, because point groups, unlike space groups, cannot contain operations of the form $\{\mathcal{T}|\mathbf{t}\}$ [SEq. (19)], as $\{\mathcal{T}|\mathbf{t}\}$ does not fix any point in position space [SEq. (26)]. Following the discussions in SN 3 and 5, all Type-I and Type-III MPGs are subgroups of Type-II SPGs. For all 122 SPGs, the group-subgroup relations are provided by Ascher and Janner in SRef. 76, and can be inferred by using the KSUBGROUPSMAG tool on the BCS (http://www.cryst.ehu.es/cgi-bin/cryst/programs/subgrmag1_k.pl)[15–18] on pairs of SSGs that are isomorphic to SPGs modulo integer lattice translations. For example, to see that Type-III MPG 9.3.31 $4'$ and Type-I MPG 9.1.29 4 are both index-2 subgroups of Type-II SPG 9.2.30 $41'$, one can choose the "List of subgroups" option in KSUBGROUPSMAG for Type-II SG 75.2 $P41'$ while specifying the magnetic wavevector $\mathbf{k} = \mathbf{0}$. Documentation and further examples of the output of KSUBGROUPSMAG are provided in Refs 18,77. For this work, we define Type-I MPG 1.1.1 1 as both the *trivial MPG* and the *trivial SPG*, as its only generator is the identity operation E, and because Type-I MPG 1.1.1 1 is the common subgroup of all MPGs and SPGs.

It is important to highlight that all site-symmetry groups in MSGs (*i.e.* Type-I, III, and IV SSGs) are isomorphic to MPGs (*i.e.* Type-I and III SPGs), and correspondingly, that all site-symmetry groups in Type-II (nonmagnetic) SSGs are isomorphic to Type-II SPGs. To show this, we first consider the Type-II SSGs. Because all Type-II SSGs contain the element $\{\mathcal{T}|\mathbf{0}\}$, which fixes all points in space, then all site-symmetry groups in Type-II SSGs also necessarily contain $\{\mathcal{T}|\mathbf{0}\}$, and are therefore isomorphic to Type-II SPGs. Conversely, because MSGs (*i.e.* Type-I, III, and IV SSGs) do not contain $\{\mathcal{T}|\mathbf{0}\}$, then *none* of their site-symmetry groups contain $\{\mathcal{T}|\mathbf{0}\}$ (though they are free to contain antiunitary operations such as $\{C_{4z}\times\mathcal{T}|\mathbf{0}\}$; hence, the site-symmetry groups in MSGs are isomorphic to either Type-I or Type-III MPGs. In Type-I MSGs, all of the site-symmetry groups are isomorphic to Type-I MPGs, as Type-I MSGs do not contain antiunitary symmetry elements (SN 3). However, in each of the Type-III and Type-IV MSGs, site-symmetry groups can be isomorphic to either Type-I or Type-III MPGs. For example, in SFig. 6(a,b), we depict atomic and spin configurations that respect the symmetries of Type-I MLG $p4$ and Type-III MLG $p4'$, respectively [see the text surrounding SEq. (23) for the definition of an MLG]. In $p4$, the $1a$ and $1b$ site-symmetry groups are isomorphic to Type-I MPG 9.1.29 4, whereas in $p4'$, the $1a$ and $1b$ site-symmetry groups are isomorphic to Type-III MPG 9.3.31 $4'$. Nevertheless, in both $p4$ and $p4'$, the $2c$ site-symmetry groups are isomorphic to Type-I MPG 3.1.6 2.

9. Wyckoff Positions of the Magnetic Space Groups

In this section, we will next reintroduce the Wyckoff positions of the MSGs. First, we will below precisely define a Wyckoff position. Then, in SN 10, we will apply the definitions and relations established below to illustrative 2D examples of MLGs derived from the Type-II layer group (LG) $p41'$.

To begin, as discussed in SRef. 1, the Wyckoff positions of an SSG M are defined using the orbits of symmetry sites. We first select a site $\mathbf{q}_\alpha$ in a crystal that is invariant under an SSG M. As defined in the text surrounding SEq. (27), the site-symmetry group $M_{\mathbf{q}_\alpha}$ contains all of the symmetries $g \in M$ that return $\mathbf{q}_\alpha$ to itself. However, there also generically exist symmetries:

$$\tilde{g}_i \in M,\ \tilde{g}_i \notin M_{\mathbf{q}_\alpha} \tag{29}$$

that act to send $\mathbf{q}_\alpha$ to other points $\mathbf{q}_{\alpha'}$ in the crystal, where $\mathbf{q}_{\alpha'}$ may or may not lie in the same unit cell as $\mathbf{q}_\alpha$. We next define the set of symmetries:

$$\{\tilde{g}\} = M \setminus M_{\mathbf{q}_\alpha}. \tag{30}$$

Acting with all of the $\tilde{g}_i \in \{\tilde{g}\}$ on $\mathbf{q}_\alpha$ generates an infinite number of sites $\{\tilde{g}_i\mathbf{q}_\alpha\}$, because M includes lattice translations and $M_{\mathbf{q}_\alpha}$ does not. Additionally, it is possible for two elements $\tilde{g}_{i,j} \in \{\tilde{g}\}$ to map $\mathbf{q}_\alpha$ to the same point. For example, if $\mathbf{q}_\alpha = (x,y,0)$, $\tilde{g}_i = \{C_{2z}|000\}$, and $\tilde{g}_j = \{\mathcal{I}|000\}$, then $\tilde{g}_i\mathbf{q}_\alpha = \tilde{g}_j\mathbf{q}_\alpha = (-x,-y,0)$. Continuing to employ the previous definition from TQC[1–6], we define the *orbit* of $\mathbf{q}_\alpha$ to be the infinite subset of unique points $\{\tilde{g}_i\mathbf{q}_\alpha\} \cup \mathbf{q}_\alpha$. We then define the *Wyckoff orbit* indexed by $\mathbf{q}_\alpha$ as the finite set of points in the orbit – including $\mathbf{q}_\alpha$ itself – that lie in the same unit cell as $\mathbf{q}_\alpha$. In this work, we will summarize the Wyckoff orbit containing $\mathbf{q}_\alpha$ using the notation $\{\mathbf{q}_\alpha\}$, for simplicity. In the Wyckoff orbit of $\mathbf{q}_\alpha$, the index α runs from 1 to n, where n – which is termed the *multiplicity* of the Wyckoff orbit – is the number of unique sites $\mathbf{q}_\alpha$ in the orbit of $\mathbf{q}_\alpha$ that lie in the same unit cell as $\mathbf{q}_\alpha$ plus one for $\mathbf{q}_\alpha$ itself. Given a site-symmetry group $M_{\mathbf{q}_\alpha}$, all of the other site-symmetry groups in the Wyckoff

orbit of $\mathbf{q}_\alpha$ are given by:

$$M_{\mathbf{q}_\beta} = \tilde{g}_{\alpha\beta} M_{\mathbf{q}_\alpha} \tilde{g}_{\alpha\beta}^{-1}, \quad (31)$$

where $\tilde{g}_{\alpha\beta}$ is a symmetry in $M \setminus M_{\mathbf{q}_\alpha}$ [SEq. (29)] for which:

$$\tilde{g}_{\alpha\beta}\mathbf{q}_\alpha = \mathbf{q}_\beta, \quad (32)$$

where $\mathbf{q}_\beta$ is in same Wyckoff orbit as $\mathbf{q}_\alpha$. Hence, all of the site-symmetry groups $M_{\mathbf{q}_\beta}$ in the same Wyckoff orbit as $M_{\mathbf{q}_\alpha}$ are isomorphic and conjugate to $M_{\mathbf{q}_\alpha}$, and to each other. Lastly, we define the *Wyckoff position* containing $\mathbf{q}_\alpha$ as the set of Wyckoff orbits with the same multiplicity in which the coordinates of the sites in the orbit $\{\mathbf{q}_\alpha\}$ can be smoothly deformed into each other without changing the Wyckoff orbit multiplicity. For example, in Type-I MSG 2.4 $P\bar{1}$, which is generated by $\{\mathcal{I}|\mathbf{0}\}$ and 3D lattice translation, the sites $[(0,0,0.1),(0,0,-0.1)]$ and $[(0,0,0.2),(0,0,-0.2)]$ define distinct Wyckoff orbits. Nevertheless, in MSG 2.4 $P\bar{1}$, the two Wyckoff orbits $[(0,0,0.1),(0,0,-0.1)]$ and $[(0,0,0.2),(0,0,-0.2)]$ represent different parameter choices for the same Wyckoff position $[(x,y,z),(-x,-y,-z)]$ (labeled the $2i$ position on the BCS). The coordinates, multiplicities, and site-symmetry groups of the Wyckoff positions of all 1,651 SSGs have previously been made accessible through the MWYCKPOS tool on the BCS (`http://www.cryst.ehu.es/cgi-bin/cryst/programs/magget_wp.pl`)[15–18].

Next, to further determine the maximal Wyckoff positions – which we will later find to be important in calculating the magnetic elementary band (co)representations (SN 17) – we follow the definition established in SRef. 2. First, we recognize that, for each Wyckoff position in the SSGs, there is a set of coordinates that defines the locations of atoms (magnetic atomic orbitals, see SN 18) occupying the Wyckoff position. In high-symmetry Wyckoff positions, some or all of the coordinates have fixed values (*e.g.* 0 or 1/2), whereas in other, lower-symmetry positions, the coordinates have free values (*e.g.* z) that represent distinct Wyckoff orbits in the same Wyckoff position [see the text following SEq. (32)]. For example, in the output of MWYCKPOS on the BCS[15–18] for Type-III MSG 10.45 $P2/m'$, the $1a$ position lies at $(0,0,0)$ and has a site-symmetry group isomorphic to Type-III MPG 5.4.15 $2/m'$, whereas the $2i$ position has sites at $(0,y,0)$ and $(0,-y,0)$, each of which has a site-symmetry group isomorphic to Type-I MPG 3.1.6 2. As an intermediate step towards defining a maximal Wyckoff position, we first establish a definition for connected Wyckoff positions. We define two Wyckoff positions to be *connected* if the coordinates of one of the sites in the lower-symmetry Wyckoff position [*e.g.* $(0,y,0)$ in the $2i$ position in the previous example in MSG 10.45 $P2/m'$] can be adjusted to coincide with the coordinates of the higher-symmetry Wyckoff position [*e.g.* the $1a$ position at $(0,0,0)$ in the previous example in MSG 10.45 $P2/m'$]. From this, we define a *maximal* Wyckoff position to be a Wyckoff position that is not connected to a Wyckoff position with a higher-symmetry site-symmetry group (*i.e.* the site-symmetry group of a maximal Wyckoff position must be a larger supergroup of the site-symmetry group of any Wyckoff position to which it is connected). This definition of a maximal Wyckoff position is identical to the previous definition established in SRefs. 1,2 for the Type-I MSGs and Type-II SSGs; in this work, we have applied the earlier definition to the Type-III and Type-IV MSGs by incorporating the action of the antiunitary symmetries $g_{A,j}$ in Type-III and Type-IV MSGs [see SN 5 and 6 and the text surrounding SEq. (26)]. Specifically, in both this work and in TQC, the set of site-symmetry groups in the maximal Wyckoff positions in an SSG M coincide with the set of maximal site-symmetry subgroups of M. In this work, the only distinction from the earlier discussion of Wyckoff positions in SRefs. 1,2 is the incorporation of the action of antiunitary symmetries through SEq. (26).

We will now discuss the relationship between the Wyckoff positions in the MSGs and the Wyckoff positions in the more familiar Type-II (nonmagnetic) SSGs. First, the Wyckoff positions of the Type-I and Type-III MSGs can straightforwardly be obtained from the Wyckoff positions of the Type-II SSGs. For the Type-I MSGs, this follows directly from the definition of a Type-II SSG (SN 4). Specifically, in a Type-II SSG $M_{II} = G \cup \mathcal{T}G$ [SEq. (3)], all of the site-symmetry groups $M_{II,\mathbf{q}}$ take the form:

$$M_{II,\mathbf{q}} = G_{\mathbf{q}} \cup \mathcal{T}G_{\mathbf{q}}. \quad (33)$$

In each Wyckoff position indexed by a site $\mathbf{q}$ in a crystal invariant under M_{II}, the multiplicity of the Wyckoff position of $\mathbf{q}$ is only determined by the unitary symmetries g of the Type-I subgroup G of M_{II}, because $\mathcal{T}$ symmetry acts as the identity on $\mathbf{q}$ [see SEq. (26) and the surrounding text]. Therefore, in a Type-I MSG G, all of the Wyckoff positions have the same multiplicities and coordinates as the Wyckoff positions in $M_{II} = G \cup \mathcal{T}G$ [SEq. (3)], and all of the site-symmetry groups $G_{\mathbf{q}}$ are isomorphic to the unitary subgroups of $M_{II,\mathbf{q}}$ [SEq. (33)].

Conversely, in a Type-III MSG $M_{III} = H \cup \mathcal{T}(G \setminus H)$ [SEq. (11)], the site-symmetry groups $M_{III,\mathbf{q}}$ can be isomorphic to either Type-I and Type-III MPGs, as previously discussed in SN 8. Nevertheless, we will show below that the multiplicities of the Wyckoff positions in M_{III} are still inherited from the "unprimed" Type-I group G in the definition of M_{III} [SEq. (11)]. Specifically, in this work, we define G to be the "unprimed" group of M_{III}, because G and M_{III} share the same symbols if primes are neglected (*i.e.*, under transforming group elements of the form

$g' = \{h \times \mathcal{T}|\mathbf{t}\} \to \{h|\mathbf{t}\}$). To show this, we first note that, because $\mathcal{T}$ symmetry acts as the identity on spatial coordinates, then:

$$\mathcal{T}\mathbf{q} = \mathbf{q}, \tag{34}$$

for all $\mathbf{q}$ in the 1,651 SSGs. Consequently, in a Type-III MSG M_{III}, only the unitary parts $\{h|\mathbf{t}\}$ of the unitary and antiunitary symmetries in M_{III} can act to send $\mathbf{q}$ to other positions [see SEq. (26) and the surrounding text]. As shown in SN 5, the unitary parts of the unitary and antiunitary symmetries in M_{III} comprise the unprimed Type-I MSG G of M_{III}. Additionally, as shown in SEq. (18), the unprimed group G of M_{III} is also the maximal unitary subgroup of the Type-II SSG $M_{III} \cup \mathcal{T}M_{III}$ (*i.e.* $M_{III} \cup \mathcal{T}M_{III} = G \cup \mathcal{T}G$). Lastly, as shown in the text surrounding SEq. (33), the Wyckoff positions of G are identical to the Wyckoff positions of $G \cup \mathcal{T}G$ [though the site-symmetry groups $G_{\mathbf{q}}$ are the unitary subgroups of the site-symmetry groups $M_{II,\mathbf{q}}$ in $G \cup \mathcal{T}G$]. From this, we conclude that the Type-I (unprimed) MSG G, the Type-II SSG $M_{III} \cup \mathcal{T}M_{III}$, and the Type-III MSG M_{III} all share the same Wyckoff-position multiplicities and coordinates. It therefore follows that each site-symmetry group $M_{III,\mathbf{q}} \subset M_{III}$ is an index-2 subgroup of $M_{II,\mathbf{q}} = G_{\mathbf{q}} \cup \mathcal{T}G_{\mathbf{q}}$ where:

$$M_{II,\mathbf{q}} \subset (M_{III} \cup \mathcal{T}M_{III}). \tag{35}$$

Specifically, $M_{III,\mathbf{q}}$ is either a Type-I site-symmetry group:

$$M_{III,\mathbf{q}} = G_{\mathbf{q}}, \tag{36}$$

or a Type-III site-symmetry group:

$$M_{III,\mathbf{q}} = H_{\mathbf{q}} \cup \mathcal{T}(G_{\mathbf{q}} \setminus H_{\mathbf{q}}), \tag{37}$$

where $H_{\mathbf{q}}$ is a site-symmetry group in the Type-I (maximal unitary) subgroup H of M_{III} [see SEq. (11) and the surrounding text]. We will shortly provide in SN 10 an example demonstrating the relationship between $G_{\mathbf{q}}$, $M_{II,\mathbf{q}}$, and $M_{III,\mathbf{q}}$ in a Type-III magnetic symmetry group.

Unlike in Type-I and Type-III MSGs, the Wyckoff positions in Type-IV MSGs have more complicated dependencies on the Wyckoff positions in the Type-II SSGs. This complication arises because the operation of $t_0\mathcal{T}$ in SEq. (19) enlarges the magnetic unit cell of a crystal with a Type-IV MSG (*i.e.* a_x^M in SFig. 5) relative to the nonmagnetic unit cell of its Type-II supergroup (*i.e.* a_x^G in SFig. 5). Hence, the primitive cell of a Type-IV MSG is always larger than the primitive cell of its Type-II supergroup). Therefore, there is no corresponding notion of an "unprimed" group for the Type-IV MSGs. Instead the multiplicities, coordinates, and site-symmetry groups in Type-IV MSGs must be determined by composing the elements of the site-symmetry groups of the unitary subgroup H in SEq. (19) with the antiunitary (antiferromagnetic) translation symmetry $t_0\mathcal{T}$. An example demonstrating the composition of the unitary site-symmetry group symmetries in H with $t_0\mathcal{T}$ in a Type-IV MSG will later be provided in SN 24.

10. Wyckoff Positions in Magnetic Subgroups of Type-II LG $p41'$

To demonstrate how the site-symmetry groups in Type-I and Type-III MSGs derive from those in Type-II SSGs, we will in this section analyze the examples of Type-II LG $M_{II} = p41'$ and its Type-I and Type-III magnetic subgroups Type-I MLG $G = p4$ and Type-III MLG $M_{III} = p4'$, respectively [SFig. 6(a,b), respectively]. M_{II} is generated by $\{C_{4z}|00\}$, $\{\mathcal{T}|00\}$, and the lattice translation $\{E|10\}$. Using MWYCKPOS on the BCS[15–18] for Type-II SG 75.2 $P41'$, which is isomorphic to $p41'$ modulo T_z [*i.e.* after the addition of out-of-plane lattice translations, see the text surrounding SEq. (23)], we obtain the coordinates of the highest-symmetry (fourfold-symmetric) maximal Wyckoff positions of $p41'$ ($1a$ and $1b$) and the SPGs isomorphic to the fourfold-symmetric maximal site-symmetry groups:

$$\begin{aligned} \mathbf{q}_{1a} &= (0,0),\ M_{II,1a} = 41' = 4 \cup (\mathcal{T})4, \\ \mathbf{q}_{1b} &= (1/2,1/2),\ M_{II,1b} = 41' = 4 \cup (\mathcal{T})4, \end{aligned} \tag{38}$$

where the symbols $41'$ and 4 respectively refer to Type-II SPG 9.2.30 $41'$ and Type-I MPG 9.1.29 4. There is also a lower-symmetry maximal Wyckoff position in Type-II MLG $p41$ in which the site-symmetry groups do not contain fourfold rotation symmetry ($2c$). The coordinates and site-symmetry-group-isomorphic SPGs of the $2c$ position in Type-II MLG $p41'$ are given by:

$$\mathbf{q}_{2c} = \{(1/2,0),\ (0,1/2)\},\ M_{II,2c} = 21' = 2 \cup (\mathcal{T})2, \tag{39}$$

where the symbols $21'$ and 2 respectively refer to Type-II SPG 3.2.7 $21'$ and Type-I MPG 3.1.6 2.

As defined in SEq. (3), the layer group $M_{II} = p41'$ admits a decomposition:

$$p41' = p4 \cup (\mathcal{T})p4, \tag{40}$$

where $p4$, which is generated only by $\{C_{4z}|00\}$ and $\{E|10\}$, is the maximal unitary subgroup of M_{II}. An atomic and spin configuration with MLG $p4$ is shown in SFig. 6(a). Using MWYCKPOS on the BCS[15–18] for MSG 75.1 $P4$, which is isomorphic to $p4$ modulo T_z, we obtain the coordinates and site-symmetry-group-isomorphic MPGs of the maximal Wyckoff positions of $p4$:

$$\begin{aligned} \mathbf{q}_{1a} &= (0,0),\ G_{1a} = 4, \\ \mathbf{q}_{1b} &= (1/2,1/2),\ G_{1b} = 4, \\ \mathbf{q}_{2c} &= \{(1/2,0),\ (0,1/2)\},\ G_{2c} = 2. \end{aligned} \tag{41}$$

where the symbols 4 and 2 respectively refer to Type-I MPG 9.1.29 4 and Type-I MPG 3.1.6 2. As discussed in the text following SEq. (33), we observe that each site-symmetry group $G_{\mathbf{q}}$ in $p4$ [SEq. (41)] is equivalent to the unitary subgroup of the site-symmetry group $M_{II,\mathbf{q}}$ of $p41'$ [SEq. (38)].

Next, we perform the analogous analysis of the Wyckoff positions and site-symmetry groups in Type-III MLG $M_{III} = p4'$, which is generated by $\{C_{4z} \times \mathcal{T}|00\}$ and $\{E|10\}$. As discussed in the text surrounding SEq. (11), $M_{III} = p4'$ admits a decomposition:

$$p4' = p2 \cup \mathcal{T}\left[(p4) \setminus (p2)\right], \tag{42}$$

in which $p2$ is the Type-I MLG generated by $\{E|10\}$, $\{E|01\}$, and $\{C_{2z}|00\} = (\{C_{4z} \times \mathcal{T}|00\})^6$, where the exponent of 6 is necessary to account for the possibility that $p4'$ is a double group (see SN 1 and SRef. 33). Because $p4$ is the unitary subgroup of $p41'$, the SSG that results from restoring $\mathcal{T}$ symmetry to $p4'$ [SEqs. (18) and (42)], then we refer to $p4$ as the "unprimed" group of $p4'$ [see SEq. (34) and the surrounding text]. An atomic and spin configuration with MLG $p4'$ is shown in SFig. 6(b). Using MWYCKPOS on the BCS[15–18] for MSG 75.3 $P4'$, which is isomorphic to $p4'$ modulo T_z, we obtain the coordinates and site-symmetry-group-isomorphic MPGs of the maximal Wyckoff positions of $p4'$:

$$\begin{aligned} \mathbf{q}_{1a} &= (0,0),\ M_{III,1a} = 4' = 2 \cup \mathcal{T}\left[(4) \setminus (2)\right], \\ \mathbf{q}_{1b} &= (1/2,1/2),\ M_{III,1b} = 4' = 2 \cup \mathcal{T}\left[(4) \setminus (2)\right], \\ \mathbf{q}_{2c} &= \{(1/2,0),\ (0,1/2)\},\ M_{III,2c} = G_{2c} = 2, \end{aligned} \tag{43}$$

where the symbols $4'$, 2, and 4 respectively refer to Type-III MPG 9.3.31 $4'$, Type-I MPG 3.1.6 2, and Type-I MPG 9.1.29 4. It is important to emphasize that MLGs $p2$ and $p4'$ do not share the same Bravais lattices: the Bravis lattice of $p2$ is oblique, whereas the Bravis lattice of $p4'$ is square. In $p2$, the sites $\mathbf{q}_{1b}^{p2} = (0,1/2)$ and $\mathbf{q}_{1c}^{p2} = (1/2,0)$ each lie in distinct, multiplicity-1, maximal Wyckoff positions. Conversely, in $p4'$, the symmetry element $\{C_{4z} \times \mathcal{T}|00\}$ relates $\mathbf{q}_{1b}^{p2} = (0,1/2)$ and $\mathbf{q}_{1c}^{p2} = (1/2,0)$, causing the two sites to merge into a single, multiplicity-2, maximal Wyckoff position in $p4'$ [$\mathbf{q}_{2c}$ in SEq. (43)]. All of the site-symmetry groups of $p4'$ are index-2 subgroups of the site-symmetry groups of $p41'$ [SEqs. (38) and (39)]. However, unlike previously in $p4$ [SEq. (41)], some of the site-symmetry groups in SEq. (43) are isomorphic to Type-III MPGs ($M_{III,1a}$ and $M_{III,1b}$), whereas others are isomorphic to Type-I MPGs (G_{2c}). Crucially, for the site-symmetry groups $M_{III,\mathbf{q}}$ in SEq. (43) that are isomorphic to Type-III MPGs, the "unprimed" site-symmetry groups $G_{\mathbf{q}}$ [*i.e.* the site-symmetry groups that result from disregarding $\mathcal{T}$ symmetry, see SEq. (34) and the surrounding text] are still isomorphic to the unitary subgroups of the nonmagnetic site-symmetry groups $M_{II,\mathbf{q}}$ of $p41'$ [SEqs. (38) and (39)]. Specifically, at the $1a$ and $1b$ positions of $p4'$, the site-symmetry groups $M_{III,1a}$ and $M_{III,1b}$ are both isomorphic to Type-III MPG 9.3.31 $4'$, whose unprimed group is Type-I MPG 9.1.29 4. Correspondingly, Type-I MPG 9.1.29 4 is also the unitary subgroup of Type-II SPG $41'$, to which the $1a$ and $1b$ site-symmetry groups of MLG $p41'$ are isomorphic [SEq. (38)].

11. Introduction to the Small Coreps of the Little Groups and Full Coreps of the MSGs

In this section, we will establish the analogous momentum-space description[1–6,33,78] of the MSGs, after having previously established a position-space description of the MSGs in SN 2 and 7. To begin, for an infinite crystal that is invariant under an SSG G, the translation group G_T [SEq. (1)] is a subgroup of G, where G_T is generated by a set

of three linearly-independent primitive translation operations:

$$t_a = \{E|\mathbf{t}_a\},\ t_b = \{E|\mathbf{t}_b\},\ t_c = \{E|\mathbf{t}_c\}. \tag{44}$$

The shape of the unit (primitive) cell, and the (gray) Bravais lattice of G, are determined by the relative lengths and directions of[33] $\mathbf{t}_{a,b,c}$. Because the crystal is periodic and infinite, then it admits a reciprocal, Fourier-transformed description that is also periodic and infinite. In reciprocal space, coordinates are indexed by crystal momentum $\mathbf{k}$, and the shapes of the reciprocal cells [*i.e.* Brillouin zones (BZs)] are determined by the primitive reciprocal lattice vectors $\mathbf{K}_{a,b,c}$, which are defined for a d-dimensional crystal as a set of d vectors $\{\mathbf{K}_j\}$ that satisfy:

$$\mathbf{t}_i \cdot \mathbf{K}_j = 2\pi\delta_{ij}. \tag{45}$$

As previously with the Bravais lattice vectors, the primitive reciprocal lattice vectors $\mathbf{K}_{a,b,c}$ of a 3D crystal must be linearly independent, but are not necessarily orthogonal (though $\mathbf{K}_{a,b,c}$ are indeed both linearly independent and orthogonal in many SSGs). We note that, in some tools on the BCS, both $\mathbf{t}_i$ and $\mathbf{K}_j$ are expressed in reduced, dimensionless units in which factors of the Bravais lattice constants a, b, c and BZ length [2π in SEq. (45)] are suppressed (*i.e.*, units in which $|\mathbf{t}_{a,b,c}| = |\mathbf{K}_{a,b,c}| = 1$). However, throughout this work, unless we are discussing the specific output of tools on the BCS, we will maintain the factor of 2π in SEq. (45), though, like on the BCS, we will employ reduced units in which $a, b, c = 1$ (*i.e.*, units in which $|\mathbf{t}_{a,b,c}| = 1$ and $|\mathbf{K}_{a,b,c}| = 2\pi$).

Similar to the Wyckoff positions in real space, there are also sets of $\mathbf{k}$ points in momentum space that are related by the symmetries of the SSG G. These $\mathbf{k}$ points subdivide into distinct sets, known as *momentum stars*, which we will rigorously define in SN 12. For this work, we have specifically implemented the MKVEC tool on the BCS, through which users can access the momentum stars of the SSGs; examples of the output of MKVEC are provided in SN 12. As we will discuss in SN 12, MKVEC subsumes the earlier KVEC tool (`https://www.cryst.ehu.es/cryst/get_kvec.html`)[13,14,79], which was only capable of generating the momentum stars of the 230 Type-I (unitary) MSGs. Additionally at each point $\mathbf{k}$ in the first BZ, energy states (Bloch wavefunctions) can be labeled by the irreducible "small" (co)reps of the little group[33,80,81] $G_{\mathbf{k}}$, which are defined in SN 13. One of the largest obstacles in constructing MTQC was the previous absence of a complete tabulation of the single-valued (spinless) and double-valued (spinful) small coreps of the little groups of all 1,651 SSGs. Specifically, we cannot calculate the MEBRs (further detailed in SN 17), without a complete tabulation of the full (space group) coreps, which are induced from the small coreps at each of the $\mathbf{k}$ points in a momentum star[2,14,82]. Previously, Miller and Love in SRef. 78 computed the single- and double-valued irreducible small (co)reps of the little groups of each MSG at high-symmetry points and along high-symmetry lines, but not along high-symmetry planes or in the BZ interior, which are required to complete the insulating compatibility relations for each MSG (SN 16) and to compute the MEBRs (SN 17). Additionally, the magnetic small (co)reps computed in SRef. 78 are not publicly available, are displayed in difficult-to-read tables outputted directly from computer code, and are hence difficult to verify. For this work, building on a prescription outlined by Bradley and Cracknell in SRef. 33, we have performed the first ever complete tabulation of the over 100,000 single- and double-valued small coreps at all $\mathbf{k}$ points and full coreps in all momentum stars of all 1,651 SSGs, which we have made freely accessible through the newly available Corepresentations tool on the BCS. Representative examples of the output of Corepresentations are provided in SN 14 and 15. Combined with the small and full coreps previously calculated for the Type-I and II SSGs for TQC[1–6], which can still be obtained through the REPRESENTATIONS DSG tool on the BCS (`http://www.cryst.ehu.es/cgi-bin/cryst/programs/representations.pl?tipogrupo=dbg`), the tools documented in this section represent the completion of over 70 years[33,61–63,78] of group-theoretic efforts to exhaustively enumerate the coreps of the 1,651 SSGs.

Additionally, using the small coreps of the little groups of the SSGs, we can further derive the compatibility relations[3,4,83–86] that constrain the coreps at adjacent $\mathbf{k}$ points throughout the BZ. For this work, we have implemented a new tool – MCOMPREL – through which the compatibility relations between pairs of $\mathbf{k}$ points in any of the 1,651 SSGs can be obtained, including, for the first time, the Type-III and Type-IV MSGs. In SN 16, we detail the methodology employed to implement MCOMPREL, as well as outline some of the subtleties that arise when calculating compatibility relations in the MSGs.

12. Little (Co)Groups, Momentum Stars, and the MKVEC Tool

In this section, we will introduce the concepts of little groups, little co-groups, and momentum stars. We will then demonstrate how the little (co)groups and momentum stars of all 1,651 SSGs can be obtained using the newly available MKVEC tool on the BCS. To begin, we define two points $\mathbf{k}$ and $\mathbf{k}'$ to be *equivalent* if:

$$\mathbf{k} - \mathbf{k}' = \mathbf{K}_{\nu}, \tag{46}$$

where $\mathbf{K}_\nu$ is an integer-valued linear combination of the reciprocal lattice vectors $\mathbf{K}_{a,b,c}$ defined in SEq. (45). In this work, we will employ a condensed notation in which two equivalent points $\mathbf{k}$ and $\mathbf{k}'$ satisfy:

$$\mathbf{k} \equiv \mathbf{k}'. \tag{47}$$

Through SEqs. (46) and (47), we establish a definition of *inequivalent* $\mathbf{k}$ points in which two points $\mathbf{k}$ and $\mathbf{k}'$ are inequivalent if:

$$\mathbf{k} - \mathbf{k}' \neq \mathbf{K}_\nu, \tag{48}$$

for all possible linear combinations of reciprocal lattice vectors $\mathbf{K}_\nu$. We summarize SEq. (48) with a condensed notation in which two inequivalent points $\mathbf{k}$ and $\mathbf{k}'$ satisfy:

$$\mathbf{k} \not\equiv \mathbf{k}'. \tag{49}$$

Consider a symmetry:

$$g = \{\tilde{R}|\mathbf{v}\}, \tag{50}$$

where g is an element of an SSG G. In this work, $\tilde{R}$ denotes an operator, whereas $P_{\tilde{R}}$ denotes the 3×3 matrix representation of the action of the unitary part of $\tilde{R}$ on coordinates in the basis of reciprocal lattice vectors. Hence, $\tilde{R}$ is basis-independent, where as $P_{\tilde{R}}$ is basis-dependent. We note that in earlier works[14,33], symmetry actions have been formulated in terms of $P_{\tilde{R}}$, rather than $\tilde{R}$, requiring the introduction of distinct symmetry actions for unitary and antiunitary symmetries g. As an example of $\tilde{R}$ and $P_{\tilde{R}}$, consider $h = \{m_z \times \mathcal{T}|\mathbf{0}\}$, for which $\tilde{R} = m_z \times \mathcal{T}$ and $P_{\tilde{R}} = \text{diag}(1, 1, -1)$ in the coordinate basis (x, y, z). At each of the $\mathbf{k}$ points in the first BZ of G, the symmetry operations g act on $\mathbf{k}$ as:

$$g\mathbf{k} = \tilde{R}\mathbf{k}, \tag{51}$$

where the tilde on $\tilde{R}$ is used to indicate that $\tilde{R}$ can be either a unitary symmetry of the form $\tilde{R} = R$ or an antiunitary symmetry of the form $\tilde{R} = R \times \mathcal{T}$. In this work, we define two points $\mathbf{k}$ and $\mathbf{k}'$ to be *dependent* if:

$$\mathbf{k}' \equiv g\mathbf{k}, \tag{52}$$

for any symmetry $g \in G$. Given a point $\mathbf{k}$, we then define the subgroup $G_\mathbf{k} \subseteq G$, as the group of symmetries $g \in G_\mathbf{k}$ that act to return $\mathbf{k}$ to itself modulo reciprocal lattice vectors:

$$g\mathbf{k} \equiv \mathbf{k}. \tag{53}$$

Specifically, if $\tilde{R}$ is unitary ($\tilde{R} = R$), then SEq. (53) is satisfied if:

$$\tilde{R}\mathbf{k} = R\mathbf{k} \equiv \mathbf{k}, \tag{54}$$

and if $\tilde{R}$ is antiunitary ($\tilde{R} = R \times \mathcal{T}$), then SEq. (53) is satisfied if:

$$\tilde{R}\mathbf{k} = -R\mathbf{k} \equiv \mathbf{k}. \tag{55}$$

$G_\mathbf{k}$ is defined as the *little group*[33] of $\mathbf{k}$. Because Bravais lattice translations $\{E|\mathbf{t}_{a,b,c}\}$ leave $\mathbf{k}$ points invariant [SEqs. (51)], then $G_\mathbf{k}$ necessarily contains the group of lattice translations G_T [SEq. (1)] at any point $\mathbf{k}$; hence, $G_\mathbf{k}$ is isomorphic to an SSG. We may also define a *little co-group* $\bar{G}_\mathbf{k}$, which is given by the (Shubnikov) point group of $G_\mathbf{k}$. Because translation operations $\mathbf{v}$ leave $\mathbf{k}$ points invariant [SEq. (51)], then symmetries with and without translations [*e.g.* twofold screw symmetry $\{C_{2z}|00\frac{1}{2}\}$ and twofold rotation symmetry $\{C_{2z}|000\}$, respectively] have the same action on $\mathbf{k}$ points. However, as we will shortly discuss in SN 13, the momentum-space [small] (co)reps of $G_\mathbf{k}$, conversely, can differ depending on whether $G_\mathbf{k}$ contains symmetries with or without fractional lattice translations $\mathbf{v}$ [*e.g.* in nonsymmorphic and symmorphic symmetry groups, respectively][33].

In general, given an SSG G and little group $G_\mathbf{k} \subseteq G$, if $G \neq G_\mathbf{k}$, then there exists a set of symmetry elements in the subset:

$$\tilde{g} \in G \setminus G_\mathbf{k}, \tag{56}$$

for which:

$$\tilde{g}\mathbf{k} \not\equiv \mathbf{k}. \tag{57}$$

SEqs. (56) and (57) define a set of m $\mathbf{k}$ points $\{\mathbf{k}_\gamma\}$ in the first BZ consisting of $\mathbf{k}$ and all $\mathbf{k}'$ that are dependent on each other and on $\mathbf{k}$ [defined in SEq. (52)], where the index γ of $\mathbf{k}_\gamma$ runs from 1 to m. The set of points $\{\mathbf{k}_\gamma\}$ is known as the *momentum star* of $\mathbf{k}$ in G, for which m indicates the number of inequivalent $\mathbf{k}$ points in the star. m can alternatively be defined as the number of $\mathbf{k}$ points in the *orbit* of $\mathbf{k}$, in analogy to the discussion of Wyckoff positions and symmetry sites in SN 9. In this work, to distinguish orbits in position space from symmetry-related $\mathbf{k}$ points in momentum space, we will refer to m as the number of *arms* in the star of $\mathbf{k}$, following the convention of SRefs. 33,79. From SEqs. (56) and (57), it follows that, for a point $\mathbf{k}' \equiv \tilde{g}\mathbf{k}$,

$$G_{\mathbf{k}'} = \tilde{g}G_{\mathbf{k}}\tilde{g}^{-1}, \tag{58}$$

such that $G_{\mathbf{k}'}$ is isomorphic (and in fact conjugate) to $G_{\mathbf{k}}$. Continuing to follow the definitions for position-space

List of k-vector types of the Magnetic Space Group *P*21' (No. 3.2)

Unitary subroup: *P*2 (No. 3) in its standard setting.

k-vector type	coordinates of the vectors of the star	magnetic little co-group	k-vector type of the unitary subgroup	coordinates of the vectors of the star in the unitary subgroup	unitary little co-group
GM	(0,0,0)	21'	GM	(0,0,0)	2
A	(1/2,0,1/2)	21'	A	(1/2,0,1/2)	2
B	(0,0,1/2)	21'	B	(0,0,1/2)	2
C	(1/2,1/2,0)	21'	C	(1/2,1/2,0)	2
D	(0,1/2,1/2)	21'	D	(0,1/2,1/2)	2
E	(1/2,1/2,1/2)	21'	E	(1/2,1/2,1/2)	2
Y	(1/2,0,0)	21'	Y	(1/2,0,0)	2
Z	(0,1/2,0)	21'	Z	(0,1/2,0)	2
LD	(0,v,0)	2	LD	(0,v,0)	2
	(0,-v,0)		LE	(0,-v,0)	
U	(1/2,v,1/2)	2	U	(1/2,v,1/2)	2
	(-1/2,-v,-1/2)		UA	(-1/2,-v,-1/2)	
V	(0,v,1/2)	2	V	(0,v,1/2)	2
	(0,-v,-1/2)		VA	(0,-v,-1/2)	
W	(1/2,v,0)	2	W	(1/2,v,0)	2
	(-1/2,-v,0)		WA	(-1/2,-v,0)	
F	(u,0,w),(-u,0,-w)	2'	F	(u,0,w),(-u,0,-w)	1
G	(u,1/2,w),(-u,1/2,-w)	2'	G	(u,1/2,w),(-u,1/2,-w)	1
GP	(u,v,w),(-u,v,-w)	1	GP	(u,v,w),(-u,v,-w)	1
	(-u,-v,-w),(u,-v,w)		GQ	(-u,-v,-w),(u,-v,w)	

Supplementary Figure 7: The output of the MKVEC tool on the BCS for Type-II SSG 3.2 $P21'$. MKVEC, which we introduce in this work, outputs the momentum stars of all 1,651 SSGs, representing an extension of the earlier KVEC tool, which was only capable of generating the momentum stars of the 230 Type-I (unitary) MSGs. From left to right, the columns in the output of MKVEC list the name ("k-vector type") of each momentum star indexed by a point $\mathbf{k}$ in the first BZ of the specified SSG G, the coordinates of the arms of the star containing $\mathbf{k}$ in the standard setting (conventional cell), the little co-group $\bar{G}_{\mathbf{k}}$, the name of the vectors in the star of $\mathbf{k}$ in the unitary subgroup H of G (SN 2), the coordinates of the arms of the star(s) in H that combine to form the star of $\mathbf{k}$ in G, and the Type-I (unitary) magnetic little co-group $\bar{H}_{\mathbf{k}}$ of $\mathbf{k}$ in H. For the labels and coordinates of the arms of each star, we have employed the convention of Stokes, Campbell, and Cordes[82] to be consistent with the ISOTROPY Software Suite, which was developed by Stokes, Hatch, and Campbell. In the example of SSG 3.2 $P21'$ shown in this figure, the unitary subgroup H of G is isomorphic to Type-I MSG 3.1 $P2$. In $H = P2$, there are more momentum stars (right-most three columns) than in $G = P21'$ (left-most three columns), due to the absence of $\{\mathcal{T}|\mathbf{0}\}$ symmetry in H. For example, in H – which is generated by $\{C_{2y}|\mathbf{0}\}$ and lattice translation symmetry, LD $(0, v, 0)$ and LE $(0, -v, 0)$ are distinct, multiplicity-1 momentum stars; however, in G, LD and LE merge into a single multiplicity-2 momentum star (also named LD) $[(0, v, 0), (0, -v, 0)]$.

quantities established in SN 9, we define two momentum stars respectively indexed by arms at $\mathbf{k}$ and $\mathbf{k}'$ to be *connected* if the coordinates of any of the arms in the star of $\mathbf{k}$ [*e.g.* the coordinate v in the LD star with two arms at $(0, v, 0)$ and $(0, -v, 0)$ in SSG 3.2 $P21'$] can be adjusted to coincide with the coordinates of any of the arms in the star of $\mathbf{k}'$ [*e.g.* the Γ point $(0, 0, 0)$ in SSG 3.2 $P21'$, which is the only arm in its star], or vice versa. From this, we then further define a *maximal* momentum star as a momentum star indexed by an arm at $\mathbf{k}$ (also known as a $\mathbf{k}$ vector of maximal symmetry[1–5,87]) for which all connected momentum stars indexed by arms at $\mathbf{k}'$ have little groups $G_{\mathbf{k}'}$ that are proper subgroups of $G_{\mathbf{k}}$:

$$G_{\mathbf{k}'} \subset G_{\mathbf{k}}, \tag{59}$$

for all $\mathbf{k}'$ connected to $\mathbf{k}$. We emphasize that a maximal momentum star may still have arms that lie along high-symmetry lines or planes, rather than high-symmetry $\mathbf{k}$ points; for example, there are maximal momentum stars with arms lying along lines and planes in SSGs that are respectively isomorphic to magnetic rod[10,33,69–71] and wallpaper[39,73,74] groups modulo translations [see the text following SEq. (2) and the text surrounding SEq. (23)]. Because SEqs. (56), (57), (58), and (59) are closely related to the definitions for real-space Wyckoff positions (SN 9), then the momentum stars are sometimes also known as the "momentum-space Wyckoff positions" of G (see SRefs. 79,88 and the KVEC tool on the BCS for more information).

Prior to the completion of this work, the momentum stars and little (co)groups of the Type-I MSGs were made available on the BCS through the KVEC tool[79]. However, the earlier tool – KVEC – only incorporated the action of unitary crystal symmetries. In this work, we introduce a new tool – MKVEC – which additionally incorporates the action of the antiunitary symmetries present in Type-II, III, and IV SSGs (SN 2). As an example, consider the lowest-symmetry momentum stars (general momentum-space Wyckoff positions[79]) in Type-I MSG 3.1 $P2$ and Type-II SSG 3.2 $P21'$ [SFig. 7]. MSG 3.1 $P2$ is generated by $\{C_{2y}|\mathbf{0}\}$ and 3D lattice translations, whereas SSG 3.2 $P21'$ is generated by $\{C_{2y}|\mathbf{0}\}$, $\{\mathcal{T}|\mathbf{0}\}$, and 3D lattice translations. In MSG 3.1 $P2$, the lowest-symmetry star sits at generic momenta in the BZ interior, and has two arms that lie at $\mathbf{k}$ and $C_{2y}\mathbf{k}$ [SEq. (51)]. Conversely, in SSG 3.2 $P21'$, the lowest-symmetry star (GP in SFig. 7) has *four* arms, which lie at $\mathbf{k}$, $C_{2y}\mathbf{k}$, $\mathcal{T}\mathbf{k}$, and $C_{2y}\mathcal{T}\mathbf{k}$.

In SFig. 8, we also show the output of MKVEC for the more complicated example of Type-III MSG 75.3 $P4'$. To explain the output of MKVEC in SFig. 8, we must first define additional terminology. First, in many cases, there exist multiple symmetry groups that are isomorphic to the same SSG. For example, the MSG generated by:

$$\{C_{2y}|\mathbf{0}\},\ \{E|100\},\ \{E|010\},\ \{E|001\}, \tag{60}$$

is isomorphic to the symmetry group generated by:

$$\{C_{2z}|\mathbf{0}\},\ \{E|100\},\ \{E|010\},\ \{E|001\}. \tag{61}$$

Furthermore, the symmetry groups generated by the elements in SEqs. (60) and (61) are both isomorphic to Type-I MSG 3.1 $P2$. In BCS applications, unless otherwise specified, all of the properties associated to a symmetry group are generated in a *standard setting* in which the choice of rotation axes and mirror planes is fixed throughout the BCS. For each symmetry group on the BCS, the standard setting is chosen to be the setting of the symmetry group in the *International Tables for Crystallography* (SRefs. 69,70). For example, unless otherwise specified, the properties of Type-I MSG 3.1 $P2$ are provided on the BCS in the (standard) setting in which MSG 3.1 $P2$ is generated by $\{C_{2y}|\mathbf{0}\}$ and 3D lattice translations [SEq. (60)]. In the nomenclature of this work and the BCS, the symmetry group generated by $\{C_{2z}|\mathbf{0}\}$ and lattice translation [SEq. (61)] is termed a *non-standard* setting of MSG 3.1 $P2$. Next, given an SSG G, we define the *Bravais class* of G to be the highest-symmetry, symmorphic[33], Type-II SSG with the same gray (nonmagnetic) Bravais lattice as G (see SN 2). As discussed in SFig. 8, MKVEC compares the momentum stars of G to the momentum stars of the Bravais class of G, and, when there is a discrepancy, outputs an additional table [the lower table in SFig. 8] indicating the specific parameters for which the momentum stars in G coincide with the momentum stars in the Bravais class of G.

Having established definitions for standard and non-standard SSG settings and Bravais classes [SEq. (61) and the surrounding text], we will now analyze the output of MKVEC for Type-III MSG 75.3 $P4'$ in SFig. 8. $G = P4'$ is generated by:

$$\{C_{4z} \times \mathcal{T}|\mathbf{0}\},\ \{E|100\},\ \{E|001\}, \tag{62}$$

such that the unitary subgroup H of G is generated by $\{C_{2z}|\mathbf{0}\}$ and 3D lattice translations, and is therefore isomorphic to Type-I MSG 3.1 in a non-standard (z-oriented) setting [SEq. (61)]. Unlike in the previous example in SFig. 7, there are two complications that we must consider in generating the momentum stars of the Type-III MSG $G = P4'$ from the momentum stars of a unitary (Type-I) MSG, whose momentum stars were previously computed for the

earlier BCS tool KVEC[13,14,79]. First, in the standard setting, MSG 3.1 $P2$ is generated by $\{C_{2y}|\mathbf{0}\}$ and 3D lattice translations [SEq. (60)], as opposed to the unitary subgroup H of $G = P4'$, which is isomorphic to MSG 3.1 $P2$ in a non-standard setting [see the text following SEq. (62)]. To begin to generate the momentum stars in G, we first employ a transformation matrix P to convert the $\mathbf{k}$ points in the standard (y-oriented) setting of MSG 3.1 $P2$ into the non-standard (z-oriented) basis of H:

$$\mathbf{k}_H = P\mathbf{k}_{P2}, \tag{63}$$

where P is the 3×3 matrix in the left three columns of the gray box at the top of SFig. 8. Next, we account for the difference in Bravais lattice between G and H. Specifically, $G = P4'$ has a primitive tetragonal Bravais lattice, whereas H has a primitive monoclinic Bravais lattice. Because of this, high-symmetry $\mathbf{k}$ points (lines) that were independent in H [*e.g.* $(0, 1/2, w)$ and $(1/2, 0, -w)$ in the upper table in SFig. 8] become merged by the symmetry $\{C_{4z} \times \mathcal{T}|\mathbf{0}\} \in G$ into the same star in G [*e.g.* W in the left-most column of the upper table in SFig. 8].

The need for a transformation matrix P [SEq. (63) and SFig. 8] and the difference in Bravais lattice lead to a potential ambiguity in the momentum-star labeling, namely whether we should employ the labels of an MSG (here 75.3 $P4'$) or those of the unitary subgroup [here 3.1 $P2$ in the non-standard (z-oriented) setting, see the text surrounding SEq. (60)]. We note that this ambiguity does not arise in all MSGs, or at all $\mathbf{k}$ points – a point $\mathbf{k}$ in an MSG G only carries a labeling ambiguity if the $\mathbf{k}$ point has a different label in the Bravais lattice of G than in the Bravais lattice of the unitary subgroup H of G. In the new tools on the BCS created for this work, we resolve a $\mathbf{k}$-point labeling ambiguity by continuing to label the $\mathbf{k}$ point using the momentum stars of G, while labeling the little group (small) coreps at $\mathbf{k}$ (which we will shortly introduce in SN 13) with *both* the momentum star labels in G and with the momentum star labels of the unitary (and possibly rotated) subgroup H (see SFig. 9 for an example of magnetic small corep labeling on the BCS).

List of k-vector types of the Magnetic Space Group *P*4' (No. 75.3)

Unitary subroup: *P*2 (No. 3). Transformation matrix to the standard setting:

$$\begin{pmatrix} 1 & 0 & 0 & 0 \\ 0 & 0 & 1 & 0 \\ 0 & -1 & 0 & 0 \end{pmatrix}$$

k-vector type	coordinates of the vectors of the star	magnetic little co-group	k-vector type of the unitary subgroup	coordinates of the vectors of the star in the unitary subgroup	unitary little co-group
A	(1/2,1/2,1/2)	4'	E	(1/2,-1/2,1/2)	2
GM	(0,0,0)	4'	GM	(0,0,0)	2
LD	(0,0,w)	2	LD	(0,-w,0)	2
M	(1/2,1/2,0)	4'	A	(1/2,0,1/2)	2
V	(1/2,1/2,w)	2	U	(1/2,-w,1/2)	2
Z	(0,0,1/2)	4'	Z	(0,-1/2,0)	2
GP	(u,v,w),(-u,-v,w)	1	GP	(u,-w,v),(-u,-w,-v)	1
	(-v,u,w),(v,-u,w)		GQ	(-v,-w,u),(v,-w,-u)	
W	(0,1/2,w)	2	V	(0,-w,1/2)	2
	(1/2,0,-w)		W	(1/2,w,0)	
WA	(1/2,0,-w)	2	W	(1/2,w,0)	2
	(0,1/2,w)		V	(0,-w,1/2)	

Other labels used in this Bravais class but that are particular cases of vectors of the previous list in this specific magnetic group

k-vector type	coordinates of a representative vector of the star	more general k-vector type	coordinates of the more general k-vector type	specific values of the coordinates	k-vector type of the unitary subgroup	coordinates of the vectors of the star in the unitary subgroup	specific values of the coordinates in the unitary subgroup
R	(0,1/2,-1/2)	W	(0,1/2,w)	w→-1/2	D	(0,1/2,1/2)	
X	(0,1/2,0)	W	(0,1/2,w)	w→0	B	(0,0,1/2)	
RA	(1/2,0,1/2)	WA	(1/2,0,-w)	w→-1/2	C	(1/2,-1/2,0)	
XA	(1/2,0,0)	WA	(1/2,0,-w)	w→0	Y	(1/2,0,0)	
DT	(0,v,0)	GP	(u,v,w)	u→0,w→0	F	(u,0,w)	u→0,w→v
DU	(v,0,0)	GP	(u,v,w)	u→v,v→0,w→0	F	(u,0,w)	u→v,w→0
S	(u,u,-1/2)	GP	(u,v,w)	v→u,w→-1/2	G	(u,1/2,w)	w→u
SA	(u,-u,1/2)	GP	(u,v,w)	v→-u,w→1/2	G	(u,-1/2,w)	w→-u
SM	(u,u,0)	GP	(u,v,w)	v→u,w→0	F	(u,0,w)	w→u
SN	(u,-u,0)	GP	(u,v,w)	v→-u,w→0	F	(u,0,w)	w→-u
T	(u,1/2,-1/2)	GP	(u,v,w)	v→1/2,w→-1/2	G	(u,1/2,w)	w→1/2
TA	(1/2,-u,1/2)	GP	(u,v,w)	u→1/2,v→-u,w→1/2	G	(u,-1/2,w)	u→1/2,w→-u
U	(0,v,-1/2)	GP	(u,v,w)	u→0,w→-1/2	G	(u,1/2,w)	u→0,w→v
UA	(v,0,1/2)	GP	(u,v,w)	u→v,v→0,w→1/2	G	(u,-1/2,w)	u→v,w→0
Y	(u,1/2,0)	GP	(u,v,w)	v→1/2,w→0	F	(u,0,w)	w→1/2
YA	(1/2,-u,0)	GP	(u,v,w)	u→1/2,v→-u,w→0	F	(u,0,w)	u→1/2,w→-u
B	(0,v,w)	GP	(u,v,w)	u→0	GP	(u,v,w)	u→0,v→-w,w→v
BA	(v,0,-w)	GP	(u,v,w)	u→v,v→0,w→-w	GP	(u,v,w)	u→v,v→w,w→0
C	(u,u,w)	GP	(u,v,w)	v→u	GP	(u,v,w)	v→-w,w→u
CA	(u,-u,-w)	GP	(u,v,w)	v→-u,w→-w	GP	(u,v,w)	v→w,w→-u
D	(u,v,0)	GP	(u,v,w)	w→0	F	(u,0,w)	w→v
E	(u,v,1/2)	GP	(u,v,w)	w→1/2	G	(u,-1/2,w)	w→v
F	(u,1/2,w)	GP	(u,v,w)	v→1/2	GP	(u,v,w)	v→-w,w→1/2
FA	(1/2,-u,-w)	GP	(u,v,w)	u→1/2,v→-u,w→-w	GP	(u,v,w)	u→1/2,v→w,w→-u

Supplementary Figure 8: The output of the MKVEC tool on the BCS for Type-III MSG 75.3 $P4'$. Unlike the previous example of SSG 3.2 $P21'$ in SFig. 7, $G = P4'$ and the unitary subgroup H of G have different Bravais lattices. Additionally, the unitary subgroup H is generated by $\{C_{2z}|\mathbf{0}\}$ and 3D lattice translation [SEq. (61)], and is therefore isomorphic to Type-I 3.1 $P2$ in a non-standard (z-oriented) setting that differs from the standard (y-oriented) setting used throughout the BCS [see SEq. (60) for the definitions of standard and non-standard settings]. In MKVEC, we account for the difference in the orientation of the twofold rotation axis between H and the standard setting of MSG 3.1 $P2$ by using the 3×3 P matrix given by the left three columns of the gray box [SEq. (63)]. After using the P matrix to reorient the twofold rotation symmetry in MSG 3.1 $P2$ to align with the twofold axis in H, we then determine which of the momentum stars (*e.g.* GP and GQ) in MSG 3.1 $P2$ (the three right-most columns in the top table) merge into the same momentum star (*e.g.* GP) in MSG 75.3 $P4'$ (the three left-most columns in the top table). MKVEC also refers to the *Bravais classes*, which are defined in the text following SEq. (61). For SSGs G with fewer momentum stars than in the Bravais class of G, MKVEC also outputs the bottom table, which lists additional $\mathbf{k}$ points that represent specific parameters for the same momentum stars in the top table chosen to coincide with distinct momentum stars in the Bravias class of G. For example, in some SSGs G – such as Type-III MSG 75.3 $P4'$ in this figure – two $\mathbf{k}$ points represent different parameter choices for the same star [*e.g.* the X point at $(0, 1/2, 0)$ and the R point at $(0, 1/2, -1/2)$ in the lower table represent different parameter choices for the W star in the upper table], even though the two $\mathbf{k}$ points lie in distinct momentum stars in the Bravais class of G [which, for the example of Type-III MSG 75.3 $P4'$, is the primitive tetragonal Type-II SSG 123.340 $P4/mmm1'$]. To summarize, in Type-III MSG 75.3 $P4'$, the R and X points and W lines are all mutually *connected* [defined in the text following SEq. (58)], and therefore appear as a single entry (W) in the top table, but the R and X points are not connected in the Bravais class of G (Type-II SSG 123.340 $P4/mmm1'$), and therefore appear as distinct entries (R and X) in the bottom table. Further details for obtaining the Bravais class of each SSG are provided in the documentation for MKVEC on the BCS.

13. Small and Full Coreps and the Corepresentations Tool

Having established the definitions of little (co)groups and momentum stars (SN 12), we will now in this section detail our tabulation of the small and full (co)reps of the MSGs. At each $\mathbf{k}$ point in a crystal, the representations of the little group $G_{\mathbf{k}}$ can be used to characterize electronic (Bloch) wavefunctions[33,80,81,89], superconducting- and magnetic-transition order parameters[90–93], magnons[94], and Raman scattering tensors[95]. For the specific purposes of MTQC, we cannot derive the magnetic elementary band (co)representations without knowledge of the set of irreducible full [*i.e.* space group] (co)reps in each momentum star induced from the irreducible small (co)reps in one arm of the star (see SN 17). Therefore, before we can continue towards characterizing energy bands and enumerating band (co)representations across the SSGs, we must tabulate all of the small (co)reps [defined below] of each little group $G_{\mathbf{k}}$ of each $\mathbf{k}$ point in each of the 1,651 SSGs, which we must then use to generate the irreducible full (co)reps in each momentum star of each SSG. Though a partial tabulation consisting of the magnetic small (co)reps at high-symmetry BZ points and along high-symmetry BZ lines was performed by Miller and Love in SRef. 78, we have in this work performed the first *complete* tabulation of the small (co)reps of $G_{\mathbf{k}}$ at all $\mathbf{k}$ points for all 1,651 single and double SSGs.

To begin, because $G_{\mathbf{k}}$ is isomorphic to an SSG [text following SEq. (53)], then $G_{\mathbf{k}}$ is an infinite group, and does not have a finite set of irreducible (co)reps. Historically, several methods have been employed to extract a physically meaningful finite set of (co)reps from $G_{\mathbf{k}}$. One option is to form a finite group from $G_{\mathbf{k}}$. If $\mathbf{k}$ is an isolated high-symmetry point, then we can form the group:

$$^{H}G_{\mathbf{k}} = G_{\mathbf{k}}/T_{\mathbf{k}}, \tag{64}$$

where $T_{\mathbf{k}}$ is the group of translations $\{E|\mathbf{t}_{\mu}\} \in T_{\mathbf{k}}$ for which $\exp(-i\mathbf{k}\cdot\mathbf{t}_{\mu}) = 1$, and where we recall that $/$ is the set quotient [SEq. (8)], as opposed to the set difference $\setminus$ [SEq. (6)]. $^{H}G_{\mathbf{k}}$ is known as "Herring's little group"[33,96]. At high-symmetry $\mathbf{k}$ points in Type-I MSGs or Type-II SSGs, it is shown in SRef. 33 that $^{H}G_{\mathbf{k}}$ is either isomorphic to an abstract finite point group, or to the direct product of an abstract finite point group and a 3D group of lattice translations that is a subgroup of the lattice translations of $G_{\mathbf{k}}$. Hence, a finite number of coreps can be generated from $^{H}G_{\mathbf{k}}$ by either encountering the case in which $^{H}G_{\mathbf{k}}$ is already a finite group, or by taking $^{H}G_{\mathbf{k}}$ modulo the remaining integer lattice translations. The (co)reps of the abstract point subgroups of $^{H}G_{\mathbf{k}}$ for all of the $\mathbf{k}$ points in the single and double Type-I MSGs and Type-II SGs were exhaustively tabulated in SRef. 33. However, the abstract point subgroups of $^{H}G_{\mathbf{k}}$ for all of the $\mathbf{k}$ points in the single and double Type-III and Type-IV MSGs have not been calculated to date. Additionally, when generalizing to high-symmetry BZ lines and planes, we can no longer rely on SEq. (64), because $G_{\mathbf{k}}/T_{\mathbf{k}}$ cannot simply be reduced to a finite group by modding out lattice translations for values of $\mathbf{k}$ away from high-symmetry points; a more complicated procedure involving the central extension of the little co-group $\bar{G}_{\mathbf{k}}$ may instead be employed, as detailed in Chapter 5 of SRef. 33.

In this work, to avoid the complications involved with reducing $G_{\mathbf{k}}$ to a finite group, we will instead employ an alternative approach in which a finite set of (co)reps can be generated for each $G_{\mathbf{k}}$ in each SSG, regardless of whether $\mathbf{k}$ is a high-symmetry BZ point. To begin, because $G_{\mathbf{k}}$ is a space group, then $G_{\mathbf{k}}$ can be expressed as a left coset decomposition with respect to the group of Bravais lattice translations G_T [SEq. (1)]:

$$G_{\mathbf{k}} = \bigcup_{i} g_i G_T = G_T \cup \bigcup_{g_i \notin G_T} g_i G_T = G_T \cup \{\tilde{R}_1|\mathbf{v}_1\}G_T \cup \{\tilde{R}_2|\mathbf{v}_2\}G_T + ..., \tag{65}$$

where the index i in SEq. (65) runs over a set of coset representatives $g_i = \{\tilde{R}_i|\mathbf{v}_i\}$ of $G_{\mathbf{k}}$ for which $g_iG_T \neq g_jG_T$ for $g_{i,j} \in G_{\mathbf{k}}$, such that each coset g_iG_T is unique. In SEq. (65), we use the tilde symbol to emphasize that the symmetry operation $\tilde{R}_i$ can be either unitary ($\tilde{R}_i = R_i$) or antiunitary ($\tilde{R}_i = R_i \times \mathcal{T}$). In the coset decomposition in SEq. (65), $g_i \neq \{E|\mathbf{0}\}$ in the second equality, because $\{E|\mathbf{0}\} \in G_T$. To motivate the coset decomposition in SEq. (65), we can compare $G_{\mathbf{k}}$ to $\bar{G}_{\mathbf{k}}G_T$, where $\bar{G}_{\mathbf{k}}$ is the little co-group [*i.e.* $\bar{G}_{\mathbf{k}}$ is the SPG obtained by setting all of the $\mathbf{v}_i \rightarrow \mathbf{0}$ in SEq. (65), see text following SEq. (55)]. First, we define a *symmorphic* SSG[33] to be an SSG G in which there exists a choice of origin for which each symmetry $g \in G$ takes the form $g = \{\tilde{R}|\mathbf{t}\}$, where $\{E|\mathbf{t}\} \in G_T$ (using the same origin for each symmetry g)[33,69]. This implies that $G_{\mathbf{k}} = \bar{G}_{\mathbf{k}}G_T$ at all $\mathbf{k}$ points. Hence, in symmorphic symmetry groups, we could in principle obtain a finite set of (co)reps of $G_{\mathbf{k}}$ by restricting consideration to the (co)reps of $\bar{G}_{\mathbf{k}}$. However, in an SSG that is not symmorphic (*i.e.* a *nonsymmorphic* SSG), there exist $\mathbf{k}$ points at which $G_{\mathbf{k}} \neq \bar{G}_{\mathbf{k}}G_T$, providing an obstacle towards generically using $\bar{G}_{\mathbf{k}}$ to obtain finite sets of (co)reps of $G_{\mathbf{k}}$. For example, at $\mathbf{k} = (0, \pi, 0)$ in nonsymmorphic Type-I MSG 4.7 $P2_1$ – which is generated by screw symmetry $\{C_{2y}|0\frac{1}{2}0\}$ and the lattice translations $\{E|100\}$ and $\{E|001\}$ – $G_{\mathbf{k}} \neq \bar{G}_{\mathbf{k}}G_T$. We further note that, because all Type-IV SSGs necessarily contain elements of the form $t_0\mathcal{T} = \{\mathcal{T}|\mathbf{t}_0\}$ for which $\{E|\mathbf{t}_0\} \notin G_T$ (*i.e.* t_0 is a fractional lattice translation, see SN 6), then all Type-IV SSGs are nonsymmorphic.

Instead, we will show below that, unlike $\bar{G}_{\mathbf{k}}$, SEq. (65) will allow us to construct a prescription for obtaining a finite set of (co)reps at all $\mathbf{k}$ points in both symmorphic and nonsymmorphic SSGs. First, we recognize that, even though $G_{\mathbf{k}}$ in SEq. (65) is an infinite group, the number of unique cosets $g_i G_T$ of $G_{\mathbf{k}}$ is *finite*. This can be seen by recognizing that E and the finite set $\{\tilde{R}_i\}$ in SEq. (65) comprise the finite little co-group $\bar{G}_{\mathbf{k}}$. Next, we recall that $G_{\mathbf{k}}$ is isomorphic to an SSG, implying that, in principle, there exist infinitely many (co)reps of $G_{\mathbf{k}}$. We therefore impose an additional restriction to (co)reps σ of $G_{\mathbf{k}}$ (not necessarily irreducible) for which lattice translations $t_\mu = \{E|\mathbf{t}_\mu\}$ have the matrix representatives:

$$\Delta_\sigma(t_\mu) = e^{-i\mathbf{k}\cdot\mathbf{t}_\mu}\mathbb{1}_{\chi_\sigma(\{E|\mathbf{0}\})}, \tag{66}$$

where $\mathbb{1}_{\chi_\sigma(\{E|\mathbf{0}\})}$ is the $\chi_\sigma(\{E|\mathbf{0}\})$-dimensional identity matrix. SEq. (66) implies that, given two symmetries $g_i \in G_{\mathbf{k}}$ and $t_\mu g_i \in G_{\mathbf{k}}$ in the same coset $g_i G_T$, where $t_\mu = \{E|\mathbf{t}_\mu\}$ and $t_\mu \in G_T$, the matrix representatives $\Delta_\sigma(g_i)$ and $\Delta_\sigma(t_\mu g_i)$ in σ in SEq. (66) – which is termed a *small*[2,13,33,82] (co)rep of $G_{\mathbf{k}}$ – are related by an overall (Bloch) phase[33]. Specifically:

$$\Delta_\sigma(t_\mu g_i) = e^{-i\mathbf{k}\cdot\mathbf{t}_\mu}\Delta_\sigma(g_i), \tag{67}$$

such that $\Delta_\sigma(t_\mu g_i)$ and $\Delta_\sigma(g_i)$ are unitarily equivalent. Using SEqs. (65), (66), and (67), we can then extract a finite set of irreducible small (co)reps from $G_{\mathbf{k}}$ by restricting focus to the indecomposable small (co)reps whose matrix representatives are not related by an overall phase, or any other unitary transformation. Specifically, we first define two (co)reps σ and σ' of a little group $G_{\mathbf{k}}$ to be *equivalent* if there exists a unitary matrix N that relates the matrix representatives $\Delta_\sigma(g)$ and $\Delta_{\sigma'}(g)$:

$$\Delta_\sigma(g) = N\Delta_{\sigma'}(g)N^\dagger, \tag{68}$$

for all $g \in G_{\mathbf{k}}$ (in which the same matrix N is used for all $g \in G_{\mathbf{k}}$). Then, using SEq. (68), we define the irreducible small (co)reps of $G_{\mathbf{k}}$ as the finite set of inequivalent (co)reps of $G_{\mathbf{k}}$ that cannot be expressed as direct sums of each other and for which the matrix representatives of integer lattice translations take the form of SEq. (66). We further note that, at high-symmetry $\mathbf{k}$ points, the small (co)reps of $G_{\mathbf{k}}$ are equivalent to the (co)reps of ${}^{H}G_{\mathbf{k}}$ [modulo lattice translations, see the text following SEq. (64)], and, along high-symmetry BZ lines, the small (co)reps of $G_{\mathbf{k}}$ are equivalent to the (co)reps of the central extension of the little co-group $\bar{G}_{\mathbf{k}}$ (see Chapter 5 in SRef. 33 for a detailed discussion of the role of the central extension in the group theory of crystalline solids). For Type-I and Type-II SSGs, the little group small (co)reps were previously tabulated by Bradley and Cracknell[33], and were reconstructed in the REPRESENTATIONS DSG tool on the BCS for TQC[1–6]. Conversely, there have been relatively few previous attempts to exhaustively tabulate the small coreps of the Type-III and Type-IV MSGs in an accessible form, though a partial tabulation consisting of the magnetic small (co)reps at high-symmetry BZ points and along high-symmetry BZ lines was performed by Miller and Love in SRef. 78 using little group decompositions of the form of SEq. (65). In this work, we have, for the first time, performed a complete tabulation of the small (co)reps of the little group $G_{\mathbf{k}}$ at each $\mathbf{k}$ point in each of the 1,651 SSGs, which we have made accessible through the Corepresentations tool on the BCS. Across all of the momentum stars of the 1,651 single and double SSGs, the completion of Corepresentations required the computation of over 100,000 single- and double-valued small (co)reps. In the text below, we will detail our methodology for tabulating the small (co)reps; in SN 14 and 15, we will additionally provide representative examples of the output of Corepresentations.

To complete our derivation of the little group small (co)reps, we return to the coset decomposition in SEq. (65). First, we recognize that, if $G_{\mathbf{k}}$ is isomorphic to a Type-I MSG, then its small (co)reps can already be obtained from either the tables in SRef. 33 or through the earlier REPRESENTATIONS DSG tool on the BCS[1–6], and no further calculations are required. Next, we consider the more complicated case in which $G_{\mathbf{k}}$ is isomorphic to a Type-II, III, or IV SSG. In this case, $G_{\mathbf{k}}$ necessarily contains antiunitary elements, and therefore admits a decomposition of the form:

$$G_{\mathbf{k}} = H_{\mathbf{k}} \cup \tilde{g}_A H_{\mathbf{k}}, \tag{69}$$

where $H_{\mathbf{k}}$ is the maximal unitary (index-2, see SN 4, 5, and 6) subgroup of $G_{\mathbf{k}}$, and $\tilde{g}_A$ is an antiunitary symmetry operation of the form:

$$\tilde{g}_A = \{R \times \mathcal{T}|\mathbf{v}\}, \tag{70}$$

where $\tilde{g}_A$ is known as the "representative" antiunitary symmetry operation, R is a unitary point-group symmetry element (proper or improper rotation or the identity E), and either $\mathbf{v} = \mathbf{0}$ or $\mathbf{v}$ is a fractional lattice translation.

As discussed earlier in SN 4, 5, and 6 and summarized in Supplementary Table 1, Type-II, III, and IV SSGs are distinguished by the form of R and $\mathbf{v}$ in SEq. (70).

SSG Definitions in Terms of SEqs. (69) and (70)		
SSG Type	Condition on R	Condition on $\mathbf{v}$
Type-II SSG	$R = E$	$\mathbf{v} = \mathbf{t}_\mu$
Type-III MSG	$R \neq E$	No constraint
Type-IV MSG	$R = E$	$\mathbf{v} \neq \mathbf{t}_\mu$, $\mathbf{v}^2 = \mathbf{t}_\mu$

Supplementary Table 1: Definitions of the SSGs with antiunitary symmetry operations (Types-II, III, and IV, respectively defined in SN 4, 5, and 6). E is the identity operation, and $\mathbf{t}_\mu$ is a Bravais lattice vector, such that $\{E|\mathbf{t}_\mu\} \in G_T$ [SEq. (1)].

Next, for each of the cosets on the right-hand side of SEq. (65) [including G_T itself], we choose one element to place into a set $\tilde{G}_\mathbf{k}$. In this work, we specifically choose the identity element $\{E|\mathbf{0}\}$ from G_T, and then, from each coset $g_i G_T$, we choose one element $g_i = \{\tilde{R}_i|\mathbf{v}_i\}$ for which each component of the translation $\mathbf{v}_i$ is chosen to satisfy $|\mathbf{v}_i \cdot \mathbf{t}_{a,b,c}| < 1$ (in reduced units where the lattice constants $a, b, c = 1$), such that either $\mathbf{v} = \mathbf{0}$ or $\mathbf{v}_i$ is a specific fractional lattice translation for which $g_i = \{\tilde{R}_i|\mathbf{v}_i\}$ is an element of the little group $G_\mathbf{k}$. We note that, if $G_\mathbf{k}$ is isomorphic to a symmorphic SSG [defined in the text following SEq. (65)], then $\tilde{G}_\mathbf{k}$ becomes a finite group [specifically, $\tilde{G}_\mathbf{k} = \bar{G}_\mathbf{k}$ if $G_\mathbf{k}$ is symmorphic, where $\bar{G}_\mathbf{k}$ is the little co-group, see the text following SEq. (55)]. We note that, in this section, we will always consider the more general case in which $\tilde{G}_\mathbf{k}$ is a set, and not necessarily a group. Using $\tilde{H}_\mathbf{k}$ – the maximal unitary subset of $\tilde{G}_\mathbf{k}$ – we can re-express SEq. (65) for a Type-II, III, or IV little group $G_\mathbf{k}$ as:

$$G_\mathbf{k} = \tilde{G}_\mathbf{k} G_\mathbf{k} = \bigcup_i h_i G_T \cup \bigcup_i g_{A,i} G_T = \left(\tilde{H}_\mathbf{k} \cup \tilde{g}_A \tilde{H}_\mathbf{k}\right) G_T, \tag{71}$$

where $\tilde{g}_A$ is the representative antiunitary symmetry operation in SEq. (70), and where the index i in SEq. (71) runs over all unique unitary $(h_i G_T)$ and antiunitary $(g_{A,i} G_T)$ cosets of $G_\mathbf{k}$. Bradley and Cracknell outline a convention[33] for choosing $\tilde{g}_A$ (for example, in Type-II little groups, the most natural choice is $\tilde{g}_A = \{\mathcal{T}|\mathbf{0}\}$); however, below, we will employ a more general procedure that is independent of the form of $\tilde{g}_A$. Because all $g_{A,i} \in \tilde{g}_A \tilde{H}_\mathbf{k}$ in SEq. (71) are antiunitary, and therefore do not have well-defined characters in any small corep of $G_\mathbf{k}$ (where the *character* $\chi_\sigma(h)$ of a unitary symmetry h in the corep σ is defined[33] as $\mathrm{Tr}[\Delta_\sigma(h)]$), then it is straightforward to see that the set of small coreps of $G_\mathbf{k}$ can only be formed from the small irreps of its unitary subgroup $H_\mathbf{k}$, which may become paired by the action of the elements $g_{A,i} \in \tilde{g}_A \tilde{H}_\mathbf{k}$. We note that it is not possible for the irreducible small coreps of $G_\mathbf{k}$ to be composed of more than two irreps of $H_\mathbf{k}$, because $H_\mathbf{k}$ is either isomorphic to $G_\mathbf{k}$ (*i.e.* $G_\mathbf{k}$ is isomorphic to a Type-I MSG, see SN 3), or $H_\mathbf{k}$ is an index-2 subgroup of $G_\mathbf{k}$ (*i.e.* $G_\mathbf{k}$ is isomorphic to a Type-II, III, or IV SSG, see SN 4, 5, and 6, respectively).

Given a small irrep σ of $H_\mathbf{k}$ with a matrix representative $\Delta_\sigma(h)$ for each symmetry $h \in \tilde{H}_\mathbf{k}$, we next define a matrix:

$$\bar{\Delta}_\sigma(h) = \left[\Delta_\sigma(\tilde{g}_A^{-1} h \tilde{g}_A)\right]^*. \tag{72}$$

As shown by Bradley and Cracknell[33], the small coreps $\tilde{\sigma}$ of $G_\mathbf{k}$ can only take one of three forms, which we designate as "types" (a), (b), and (c):

- Type (a): $\bar{\Delta}_\sigma(h)$ is equivalent to $\Delta_\sigma(h)$, such that $\Delta_\sigma(h) = N\bar{\Delta}_\sigma(h)N^\dagger$ for all $h \in \tilde{H}_\mathbf{k}$ [SEq. (68)]. Additionally, for coreps of type (a), the antiunitary matrix representative $\Delta_\sigma(\tilde{g}_A) = NK$, where K is complex conjugation, carries the property that $\Delta_\sigma(\tilde{g}_A^2) = [\Delta_\sigma(\tilde{g}_A)]^2 = NN^* = N^2$ [which is well defined, because $\tilde{g}_A^2 \in \tilde{H}_\mathbf{k} G_T$ in SEq. (71)]. For coreps of type (a), this implies that:

$$\tilde{\sigma} \equiv \sigma, \tag{73}$$

  such that the small corep $\tilde{\sigma}$ of $G_\mathbf{k}$ is equivalent to a small irrep σ of $H_\mathbf{k}$. However, because $G_\mathbf{k}$ and $H_\mathbf{k}$ are different symmetry groups, then the equivalence between $\tilde{\sigma}$ and σ is defined differently than the equivalence that we previously defined between (co)reps of the same symmetry group [see the text surrounding SEq. (68)]. Specifically, in this work, we define an irrep σ of a Type-I (unitary) symmetry group $H_\mathbf{k}$ and a corep $\tilde{\sigma}$ of an index-2 Type-II, III, or IV (antiunitary) supergroup $G_\mathbf{k}$ of $H_\mathbf{k}$ to be equivalent if there exists a unitary matrix N that relates the matrix representatives $\Delta_\sigma(h)$ and $\Delta_{\tilde{\sigma}}(h)$ by $\Delta_\sigma(h) = N\Delta_{\tilde{\sigma}}(h)N^\dagger$ for all of the *unitary* symmetries $h \in H_\mathbf{k}$, $h \in G_\mathbf{k}$ (where the same matrix N is used for all $h \in H_\mathbf{k}$, $h \in G_\mathbf{k}$). In nonmagnetic

(Type-II) SSGs, type (a) coreps are most familiarly encountered at $\mathbf{k}$ points with real symmetry eigenvalues in the absence of SOC. For example, at $\mathbf{k}=\mathbf{0}$ in Type-II SSG 2.5 $P\bar{1}1'$ in the absence of SOC, which is generated by $\{\mathcal{I}|\mathbf{0}\}$, $\{\mathcal{T}|\mathbf{0}\}$, and 3D lattice translations, $G_{\mathbf{k}}$ has two, one-dimensional, single-valued small coreps that each correspond to a singly degenerate, $\mathcal{T}$-invariant Bloch state (per spin)[33]. Type (a) coreps also exist in nonmagnetic SSGs G in the presence of SOC at $\mathcal{T}$-invariant $\mathbf{k}$ points with complex-conjugate pairs of spinful symmetry eigenvalues that are already paired by unitary crystal symmetries in the unitary subgroup $H_{\mathbf{k}}$ of $G_{\mathbf{k}}$. For example, at $\mathbf{k}=\mathbf{0}$ in Type-II SSG 25.57 $Pmm2$ in the presence of SOC, which is generated by $\{m_x|\mathbf{0}\}$, $\{m_y|\mathbf{0}\}$, $\{\mathcal{T}|\mathbf{0}\}$, and 3D lattice translations, $G_{\mathbf{k}}$ has one, two-dimensional small corep that is equivalent to a two-dimensional small irrep σ of $H_{\mathbf{k}}$ with complex-conjugate pairs of $m_{x,y}$ eigenvalues due to the anticommutation relation $\{\Delta_\sigma(m_x),\Delta_\sigma(m_y)\}=0$.

- Type (b): $\bar{\Delta}_\sigma(h)$ is equivalent to $\Delta_\sigma(h)$ for all $h\in\tilde{H}_{\mathbf{k}}$, where equivalence continues to be defined by SEq. (68). However, for coreps of type (b), $\Delta_\sigma(\tilde{g}_A^2)=NN^*=-N^2$, implying through Kramers' theorem that:

$$\tilde{\sigma}=\sigma\oplus\sigma\equiv\sigma\sigma, \tag{74}$$

  such that the small corep $\tilde{\sigma}$ of $G_{\mathbf{k}}$ is formed from pairing two copies of the same small irrep σ of $H_{\mathbf{k}}$. We further note that, because $\tilde{g}_A$ exchanges the two irreps σ that comprise $\tilde{\sigma}$ in SEq. (74), then the matrix representative $\Delta_\sigma(\tilde{g}_A)$ is itself undefined for a single irrep σ. Instead for coreps $\tilde{\sigma}$ of type (b), the antiunitary matrix representative $\Delta_{\tilde{\sigma}}(\tilde{g}_A)$ is only well-defined in the larger space of the two irreps σ in $\tilde{\sigma}$, in which the unitary part of $\Delta_{\tilde{\sigma}}(\tilde{g}_A)$ is block-off-diagonal. In nonmagnetic (Type-II) SSGs, type (b) coreps are most familiarly encountered at $\mathbf{k}$ points with real symmetry eigenvalues in the presence of SOC. For example, at $\mathbf{k}=\mathbf{0}$ in Type-II SSG 2.5 $P\bar{1}1'$ in the presence of SOC, which is generated by $\{\mathcal{I}|\mathbf{0}\}$, $\{\mathcal{T}|\mathbf{0}\}$, and 3D lattice translations, $G_{\mathbf{k}}$ has two, two-dimensional, double-valued small coreps that each correspond to a doubly-degenerate (Kramers) pair of Bloch states with two parity ($\mathcal{I}$) eigenvalues of the same sign[33].

- Type (c): $\bar{\Delta}_\sigma(h)$ is *not* equivalent to $\Delta_\sigma(h)$ [*i.e.*, there does not exist a matrix N that satisfies SEq. (68) for all of the symmetries $h\in\tilde{H}_{\mathbf{k}}$]. Instead, $\bar{\Delta}_\sigma(h)$ is equivalent to $\Delta_{\sigma'}(h)$, where σ' is a *different* small irrep of $H_{\mathbf{k}}$ than σ. This implies that:

$$\tilde{\sigma}=\sigma\oplus\sigma'\equiv\sigma\sigma', \tag{75}$$

  such that the small corep $\tilde{\sigma}$ of $G_{\mathbf{k}}$ is formed from pairing two different small irreps σ and σ' of $H_{\mathbf{k}}$. Unlike in coreps of type (a) or type (b), there is no constraint on the form of the matrix representative $\Delta_\sigma(\tilde{g}_A^2)$ in coreps of type (c). However, like previously in SEq. (74), the unitary part of $\Delta_{\tilde{\sigma}}(\tilde{g}_A)$ for a type (c) corep $\tilde{\sigma}$ [SEq. (75)] is off-diagonal in the block basis of σ and σ', and $\Delta_\sigma(\tilde{g}_A)$ cannot by itself be defined for a single irrep σ or σ'. In nonmagnetic (Type-II) SSGs, type (c) coreps are most familiarly encountered at $\mathbf{k}$ points with complex symmetry characters in $H_{\mathbf{k}}$, whether or not SOC is taken into consideration. For example, at $\mathbf{k}=\mathbf{0}$ in Type-II SSG 6.19 $Pm1'$ in the presence of SOC, which is generated by $\{m_y|\mathbf{0}\}$, $\{\mathcal{T}|\mathbf{0}\}$, and 3D lattice translations, $G_{\mathbf{k}}$ has one, two-dimensional, double-valued small corep that corresponds to a doubly-degenerate (Kramers) pair of Bloch states with a complex-conjugate ($\pm i$) pair of m_y eigenvalues[33].

The above definitions seem to imply that the type of small corep $\tilde{\sigma}$ induced in $G_{\mathbf{k}}$ can only be determined through a careful selection of $\tilde{g}_A$ in SEq. (71), followed by an exhaustive search for equivalence matrices N that satisfy SEq. (68). However, as shown by Bradley and Cracknell[33], we can also diagnose the type of the induced corep simply by calculating the modified Frobenius-Schur indicator[97–99] [*c.f.* Eq. (7.3.48) in SRef. 33]:

$$J_\sigma=\sum_i\chi_\sigma(g_{A,i}^2), \tag{76}$$

where $\chi_\sigma(h)$ is the character of the unitary symmetry operation $h_i=g_{A,i}^2$, $h_i\in H_{\mathbf{k}}$ in the small irrep σ of $H_{\mathbf{k}}$ [which is equal to the trace of the matrix representative $\Delta_\sigma(h_i)$], and where the summation in SEq. (76) runs over all of the antiunitary coset representatives in SEq. (71) (*i.e.*, all of the distinct elements $g_{A,i}\in\tilde{g}_A\tilde{H}_{\mathbf{k}}$). Because σ in SEq. (76) is an irrep, J_σ can only assume one of three values[33,97,98]:

$$J_\sigma=\begin{cases}|\tilde{H}_{\mathbf{k}}|, & \sigma\text{ induces a small corep }\tilde{\sigma}\text{ of type (a) in }G_{\mathbf{k}}\\ -|\tilde{H}_{\mathbf{k}}|, & \sigma\text{ induces a small corep }\tilde{\sigma}\text{ of type (b) in }G_{\mathbf{k}}\\ 0, & \sigma\text{ induces a small corep }\tilde{\sigma}\text{ of type (c) in }G_{\mathbf{k}}\end{cases}, \tag{77}$$

where $|\tilde{H}_{\mathbf{k}}|$ is the number of elements [see the text following SEq. (8)] in the set $\tilde{H}_{\mathbf{k}}$ [SEq. (71)]. In a Type-II little group, $\tilde{g}_A H_{\mathbf{k}} = \{\mathcal{T}|\mathbf{0}\}H_{\mathbf{k}}$, such that:

$$J_{\sigma}^{II} = \mathrm{sgn}\left[\chi_{\sigma}(\mathcal{T}^2)\right]\sum_{i}\chi_{\sigma}(h_i^2), \tag{78}$$

where the summation in SEq. (78) runs over all of the unitary coset representatives in SEq. (71) (*i.e.*, all of the elements $h_i \in \tilde{H}_{\mathbf{k}}$). We note that SEq. (78) is the well-established Herring test[33,96] (*i.e.* the standard Frobenius-Schur indicator[33,97,98]) for determining the "reality" of σ in a nonmagnetic (Type-II) symmetry group. However, for little groups that are isomorphic to Type-III and Type-IV MSGs, there is no analogous simple relationship between the reality of σ and the type of $\tilde{\sigma}$, and the more general formulas in SEqs. (76) and (77) must be employed to determine the type of $\tilde{\sigma}$. To confirm our complete calculation of all of the small coreps $\tilde{\sigma}$ of the SSGs, we have performed both of the independent analyses detailed in this section. Specifically, for all of the unitary subgroup small irreps σ and induced small coreps $\tilde{\sigma}$ of the little groups $G_{\mathbf{k}}$ at all $\mathbf{k}$ points in all 1,651 single and double SSGs, we have checked for all possible equivalences between $\Delta_{\sigma}(h)$ and $\bar{\Delta}_{\sigma}(h)$ [SEq. (72) and the surrounding text], and we have confirmed that the results agree with the values of J_{σ} [SEqs. (76) and (77)]. We will shortly provide in SN 15 an example of the explicit computation of J_{σ} [SEq. (77)] in a magnetic little group.

In addition to calculating the small (co)reps of the little groups of the MSGs, we have also calculated, for the first time, the *full* (co)reps of each momentum star of each MSG. Whereas each small (co)rep is a representation of the little group $G_{\mathbf{k}}$ at a point $\mathbf{k}$, each full (co)rep is a representation of the entire SSG G in the momentum star indexed by $\mathbf{k}$ (SN 12). To calculate the full (co)reps, we adapt the procedure employed in SRefs. 2,14,82 to the most general case of a magnetic or nonmagnetic SSG G. First, we recognize that, given a little group $G_{\mathbf{k}} \subseteq G$, there may exist a set of symmetries:

$$\tilde{g} \in G \setminus G_{\mathbf{k}}, \tag{79}$$

for which:

$$\tilde{g}\mathbf{k} \equiv \mathbf{k}' \not\equiv \mathbf{k}, \tag{80}$$

such that $\mathbf{k}$ and $\mathbf{k}'$ lie in different arms of the same momentum star in G. Because the little group $G_{\mathbf{k}'}$ is conjugate to $G_{\mathbf{k}}$ [SEq. (58)], then the (co)reps at $\mathbf{k}$ and $\mathbf{k}'$ are not independent. Specifically, if there exists a Bloch eigenstate at $\mathbf{k}$ labeled by a (co)rep $\tilde{\sigma}_{\mathbf{k}}$ of $G_{\mathbf{k}}$, then there must also exist a Bloch eigenstate at $\mathbf{k}'$ labeled by a (co)rep $\tilde{\sigma}_{\mathbf{k}'}$ of $G_{\mathbf{k}'}$. For $\tilde{\sigma}_{\mathbf{k}}$ and $\tilde{\sigma}_{\mathbf{k}'}$, the matrix representatives of each unitary symmetry $h \in \tilde{H}_{\mathbf{k}}$ and $\tilde{g}h\tilde{g}^{-1} \in \tilde{g}\tilde{H}_{\mathbf{k}}\tilde{g}^{-1}$ are related by the symmetries $\tilde{g} \in G \setminus G_{\mathbf{k}}$. If $\tilde{g}$ is unitary, then:

$$\Delta_{\tilde{\sigma}_{\mathbf{k}'}}(\tilde{g}h\tilde{g}^{-1}) = \Delta_{\sigma_{\mathbf{k}}}(h), \tag{81}$$

and if $\tilde{g}$ is antiunitary, then:

$$\Delta_{\tilde{\sigma}_{\mathbf{k}'}}(\tilde{g}h\tilde{g}^{-1}) = [\Delta_{\sigma_{\mathbf{k}}}(h)]^*. \tag{82}$$

Finally, we will use SEqs. (81) and (82) for each of the symmetries $\tilde{g} \in G\setminus G_{\mathbf{k}}$, to compute the matrix representatives of the full (co)rep $\tilde{\Sigma}_{\mathbf{k}}$ of G in the star indexed by $\mathbf{k}$. First, we define the full (co)rep of G in the star of $\mathbf{k}$ to be:

$$\tilde{\Sigma}_{\mathbf{k}} = \bigoplus_{i=1}^{m}\tilde{\sigma}_{\mathbf{k}_i}, \tag{83}$$

in which $\mathbf{k}_i$ is the ith arm of the multiplicity-m momentum star of $\mathbf{k}$. In SEq. (83), $\tilde{\Sigma}_{\mathbf{k}}$ is an $m \times \chi_{\tilde{\sigma}_{\mathbf{k}}}(\{E|\mathbf{0}\})$-dimensional full (co)rep of G. The matrix representatives $\Delta_{\tilde{\Sigma}_{\mathbf{k}}}(h)$ of the unitary SSG symmetries $h \in G$ are not necessarily block-diagonal, because $\tilde{\sigma}_{\mathbf{k}}$ and $\tilde{\sigma}_{\mathbf{k}'}$ in SEqs. (81) and (82) may not be equivalent [defined in SEq. (68) and the surrounding text]. Instead we may choose a basis in which $\Delta_{\tilde{\Sigma}_{\mathbf{k}}}(h)$ is block-diagonal if the unitary symmetry $h \in H_{\mathbf{k}_i}$ for all of the points $\mathbf{k}_i$ in the momentum star indexed by $\mathbf{k}$, and is otherwise not block-diagonal.

Rather than list the over 100,000 small and full (co)reps computed for this work in paper-format tables, we have implemented the Corepresentations tool on the BCS, through which the irreducible small and full (co)reps at any $\mathbf{k}$ point and in any momentum star in any SSG can respectively be accessed. Representative examples demonstrating the output of Corepresentations are provided below in SN 14 and 15.

14. Small and Full Coreps at the X and XA Points in Type-III MSG 75.3 $P4'$

In this section, we will determine the small coreps of the little group G_X of the X point in Type-III MSG 75.3 $P4'$, as well as the full coreps induced in the momentum star of X consisting of X (which in some works is alternatively labeled as X' or Y) and XA (which in some works is alternatively labeled X). MSG 75.3 $P4'$ is generated by:

$$\{C_{4z}\times\mathcal{T}|000\},\ \{E|100\},\ \{E|001\}, \tag{84}$$

and the maximal unitary subgroup H of $G = P4'$ [see SEq. (11) and the surrounding text] is generated by:

$$\{C_{2z}|000\},\ \{E|100\},\ \{E|010\},\ \{E|001\}. \tag{85}$$

Hence, H is isomorphic to Type-I MSG 3.1 $P2$ in a non-standard (z-oriented) setting [see the text surrounding SEq. (61) for the definitions of standard and non-standard symmetry-group settings]. SEqs. (84) and (85) imply the decomposition:

$$G = P4' = H \cup \{C_{4z}\times\mathcal{T}|000\}H, \tag{86}$$

in which H is isomorphic to the z-oriented (non-standard) setting of Type-I MSG 3.1 $P2$.

The X point in $G = P4'$ is one arm of a multiplicity-2 momentum star. In the convention of the BCS, the X point lies at:

$$\mathbf{k}_X = 2\pi(0,1/2,0), \tag{87}$$

where the other arm of the momentum star indexed by $\mathbf{k}_X$ lies at:

$$\mathbf{k}_{XA} \equiv (C_{4z}\times\mathcal{T})\mathbf{k}_X \equiv 2\pi(1/2,0,0). \tag{88}$$

For all of the unitary elements $h\in H$:

$$h\mathbf{k}_X \equiv \mathbf{k}_X. \tag{89}$$

However, for all of the antiunitary elements $\tilde{g}\in\{C_{4z}\times\mathcal{T}|000\}H$ in SEq. (86):

$$\tilde{g}\mathbf{k}_X \not\equiv \mathbf{k}_X. \tag{90}$$

SEqs. (89) and (90) imply that the little group G_X is isomorphic to its maximal unitary subgroup H_X. In turn, H_X at the point $\mathbf{k}_X = (0,\pi,0)$ in H is isomorphic to H_B at the point $\mathbf{k}_B = (0,0,\pi)$ in Type-I MSG 3.1 $P2$ in its standard (y-oriented setting, see SFig. 8). Therefore, the small coreps of G_X are simply equivalent to the small irreps of H_X, which are equivalent to the small irreps of H_B in MSG 3.1 $P2$, where representation equivalence is defined in the text surrounding SEq. (68).

In SFigs. 9 and 10, we show the output of the Corepresentations tool for the X point in Type-III MSG 75.3 $P4'$, which has been split into two figures in order to preserve the legibility of the output text. First, in SFig. 9, we show the matrix representatives $\Delta_{\tilde{\sigma}_X}(h)$ of the symmetries $h\in\tilde{H}_X$ [SEq. (65)] in each of the small coreps $\tilde{\sigma}_X$ of H_X. Then, in SFig. 10, we show the matrix representatives $\Delta_{\tilde{\Sigma}_X}(g)$ of the symmetries $g\in G$ in each of the *full* coreps $\tilde{\Sigma}_X$ of G in the star indexed by $\mathbf{k}_X$ [$\{\mathbf{k}_X,\mathbf{k}_{XA}\}$]. Specifically, as shown in SEq. (88), $\mathbf{k}_X$ and $\mathbf{k}_{XA}$ are related by the antiunitary symmetry $\{C_{4z}\times\mathcal{T}|000\}$, for which:

$$\{C_{4z}\times\mathcal{T}|000\}\{C_{2z}|000\}\{(C_{4z}\times\mathcal{T})^{-1}|000\} = \{C_{2z}|000\}. \tag{91}$$

SEqs. (82) and (91) imply that:

$$\Delta_{\tilde{\sigma}_{XA}}(h) = [\Delta_{\tilde{\sigma}_X}(h)]^*, \tag{92}$$

for each unitary symmetry $h\in\tilde{H}_X$ [see the text surrounding SEq. (71)], which is given by:

$$\tilde{H}_X = \Big\{\{E|000\},\ \{C_{2z}|000\},\ \{\bar{E}|000\},\ \{\bar{E}C_{2z}|000\}\Big\}. \tag{93}$$

In SEq. (93), $\bar{E} = C_{1n}$ is the symmetry operation of 360° rotation about an arbitrary axis n, which distinguishes single-valued (spinless) and double-valued (spinful) (co)reps. Throughout the BCS, $\bar{E}$ is also sometimes denoted with the Seitz symbol ${}^{d}1$, as it is in SFigs. 9 and 10.

SEqs. (92) and (93) imply that the full coreps $\tilde{\Sigma}_X$ consist of pairs of single-valued coreps at $\mathbf{k}_X$ and $\mathbf{k}_{XA}$ with the same real (spinless) C_{2z} eigenvalues [labeled ${}^*(X)B_1Y_1$ and ${}^*(X)B_2Y_2$ in SFig. 10], and pairs of double-valued coreps with opposite imaginary (spinful) C_{2z} eigenvalues [labeled ${}^*(X)\bar{B}_3\bar{Y}_4$ and ${}^*(X)\bar{B}_4\bar{Y}_3$ in SFig. 10]. Additionally, because the momentum star $\{\mathbf{k}_X, \mathbf{k}_{XA}\}$ is left invariant under all of the symmetries $g \in P4'$, then the matrix representatives $\Delta_{\tilde{\Sigma}_X}(g)$ are well-defined for all of the symmetries $g \in P4'$. This implies that $\Delta_{\tilde{\Sigma}_X}(g)$ is well defined for both the unitary symmetries $h \in G_X$, as well as the *antiunitary symmetries* $\tilde{g} \in P4' \setminus H$, where H is the maximal unitary subgroup of $G = P4'$ [SEq. (86)], and where H is isomorphic to Type-I MSG 3.1 $P2$ in a non-standard (z-oriented) setting [see the text surrounding SEq. (61)].

For each full corep $\tilde{\Sigma}_{\mathbf{k}}$ of an SSG G in a momentum star indexed by an arm $\mathbf{k}$, Corepresentations outputs the matrix representative $\Delta_{\tilde{\Sigma}_{\mathbf{k}}}(g)$ for each of the unitary and antiunitary symmetries $g \in G$. For example, unlike the table in SFig. 9 for the small coreps of G_X, the table in SFig. 10 for the full coreps of G in the star of $\mathbf{k}_X$ contains the antiunitary matrix representatives $\Delta_{\tilde{\Sigma}_X}(\{C_{4z} \times \mathcal{T}|000\})$. For each full corep $\tilde{\Sigma}_X$ and antiunitary symmetry $g_A \in G$, the full (co)rep table in Corepresentations displays the unitary part of the matrix representative $\Delta_{\tilde{\Sigma}_X}(g_A)$, which is colored in red to indicate that $\Delta_{\tilde{\Sigma}_X}(g_A)$ is antiunitary. In general, $\Delta_{\tilde{\Sigma}_{\mathbf{k}}}(g)$ for each of the unitary and antiunitary symmetries $g \in G$ is block-diagonal if $g \in G_{\mathbf{k}_i}$ for all of the points $\mathbf{k}_i$ in the momentum star indexed by $\mathbf{k}$, and is otherwise non-diagonal. For example, in SFig. 10, each $\Delta_{\tilde{\Sigma}_X}(g)$ is a 2×2 matrix, because each small corep $\tilde{\sigma}_X$ in SFig. 9 is one-dimensional. Additionally, in SFig. 10, each $\Delta_{\tilde{\Sigma}_X}(g)$ is diagonal for each symmetry $g \in G$, $g \in G_X$ and $g \in G_{XA}$ [*e.g.* $\{C_{2z}|000\}$], but is non-diagonal for each symmetry $g \in G$, $g \notin G_X$ or $g \notin G_{XA}$ [*e.g.* $\{C_{4z} \times \mathcal{T}|000\}$].

Irreducible co-representations of the (Double) Magnetic Space Group *P*4' (No. 75.3) and wave-vector X:(0,1/2,0)

Unitary (Double) Space Group: *P*2 (No. 3).
Transformation matrix to its standard setting:

$$\begin{pmatrix} 1 & 0 & 0 & 0 \\ 0 & 0 & 1 & 0 \\ 0 & -1 & 0 & 0 \end{pmatrix}$$

Coordinates of the wave-vector in the standard setting of the unitary subgroup: B:(0,0,1/2)

Magnetic little co-group of the wave-vector: 2

Little co-group of the wave-vector in the unitary subgroup: 2

Irreducible co-representations of the magnetic little group.

The matrices of the representations (the whole representation and the representation of the little group) with dimension smaller than 5 are given explicitly.
When the dimension of the representation is larger than 5, only the non-zero elements are given using the following notation: (i;j)=x means that the (i,j) element of the matrix is x.

Matrix presentation (In red color the antiunitary operations)		Seitz Symbol (In red color the antiunitary operations)	(X)B_1	(X)B_2	(X)$\overline{B}_3$	(X)$\overline{B}_4$
$\begin{pmatrix} 1 & 0 & 0 & t1 \\ 0 & 1 & 0 & t2 \\ 0 & 0 & 1 & t3 \end{pmatrix}$	$\begin{pmatrix} 1 & 0 \\ 0 & 1 \end{pmatrix}$	{1|t1,t2,t3}	$e^{i\pi t2}$	$e^{i\pi t2}$	$e^{i\pi t2}$	$e^{i\pi t2}$
$\begin{pmatrix} -1 & 0 & 0 & 0 \\ 0 & -1 & 0 & 0 \\ 0 & 0 & 1 & 0 \end{pmatrix}$	$\begin{pmatrix} -i & 0 \\ 0 & i \end{pmatrix}$	{2_{001}|0,0,0}	1	-1	-i	i
$\begin{pmatrix} 1 & 0 & 0 & 0 \\ 0 & 1 & 0 & 0 \\ 0 & 0 & 1 & 0 \end{pmatrix}$	$\begin{pmatrix} -1 & 0 \\ 0 & -1 \end{pmatrix}$	{$^{d}1$|0,0,0}	1	1	-1	-1
$\begin{pmatrix} -1 & 0 & 0 & 0 \\ 0 & -1 & 0 & 0 \\ 0 & 0 & 1 & 0 \end{pmatrix}$	$\begin{pmatrix} i & 0 \\ 0 & -i \end{pmatrix}$	{$^{d}2_{001}$|0,0,0}	1	-1	i	-i

Supplementary Figure 9: The output of the Corepresentations tool for the X point in Type-III MSG 75.3 $P4'$, part 1. Even though MSG 75.3 $P4'$ contains antiunitary symmetries, the little group G_X at $\mathbf{k}_X = (0,\pi,0)$ does not contain antiunitary symmetries, and is therefore isomorphic to H_X, its maximal unitary subgroup [see the text following SEq. (90)]. At the top of this figure, the 3×3 matrix in the left-most three columns of the gray box is the transformation matrix P that converts $\mathbf{k}$ points into the standard setting of the unitary subgroup. Specifically, in $G = P4'$ [SEq. (84)], the unitary subgroup H [SEq. (85)] is isomorphic to Type-I MSG 3.1 $P2$ in a non-standard (z-oriented) setting [see the text surrounding SEq. (61)]; as discussed in SFig. 8 and in the text surrounding SEq. (63), the P matrix in the gray box allows quantities – such as momentum stars and small irreps – previously computed on the BCS for Type-I MSGs (here MSG 3.1 $P2$) to be transformed and adapted to the computation of the analogous quantities in SSGs with antiunitary symmetries (Type-II, III, and IV SSGs, see SN 4, 5, and 6, respectively). The table in this figure shows the matrix representatives of the small coreps $\tilde{\sigma}$ of the little group G_X, for which the coreps with (without) overbars are double- (single-) valued. Because G_X in $P4'$ is isomorphic to G_B in Type-I MSG 3.1 $P2$, then the coreps in this figure are labeled $(X)B_i$, and the table in this figure contains the same entries as the table returned by Corepresentations for the B point in $P2$ [up to the orientation of the twofold axis, see the text following SEq. (90) and SFig. 8]. We note that throughout this work, a translation $\mathbf{t}$ is represented at a crystal momentum $\mathbf{k}$ by $\exp(-i\mathbf{k}\cdot\mathbf{t})$ [*i.e.*, in reduced units in which the lattice constants $a,b,c = 1$], whereas on the BCS, $\mathbf{t}$ is represented at $\mathbf{k}$ by the phase $\exp(2\pi i\mathbf{k}\cdot\mathbf{t})$ [*i.e.* with the opposite sign as employed in this work, and in different reduced units in which $\mathbf{t}$ and $\mathbf{k}$ are respectively expressed as multiples of the lattice and reciprocal lattice constants a,b,c and $2\pi/(a,b,c)$]. We additionally note that the output of Corepresentations for the X point in Type-III MSG 75.3 $P4'$ contains an additional table, which is shown in SFig. 10.

The full corepresentation is induced from representations of the following two stars, related by the anti-unitary operations of the magnetic group

X:(0,1/2,0)

XA:(1/2,0,0)

Coordinates of the vectors of the stars in the standard setting of the unitary subgroup:

B:(0,0,1/2)

Y:(1/2,0,0)

Irreducible full co-representations of the magnetic group.

**Matrix presentation** (In red color the antiunitary operations)		**Seitz Symbol** (In red color the antiunitary operations)	*(X)B$_1$Y$_1$	*(X)B$_2$Y$_2$	*(X)$\bar{B}_3\bar{Y}_4$	*(X)$\bar{B}_4\bar{Y}_3$
$\begin{pmatrix} 1 & 0 & 0 & t1 \\ 0 & 1 & 0 & t2 \\ 0 & 0 & 1 & t3 \end{pmatrix}$	$\begin{pmatrix} 1 & 0 \\ 0 & 1 \end{pmatrix}$	{1|t1,t2,t3}	$\begin{pmatrix} e^{i\pi t2} & 0 \\ 0 & e^{i\pi t1} \end{pmatrix}$	$\begin{pmatrix} e^{i\pi t2} & 0 \\ 0 & e^{i\pi t1} \end{pmatrix}$	$\begin{pmatrix} e^{i\pi t2} & 0 \\ 0 & e^{i\pi t1} \end{pmatrix}$	$\begin{pmatrix} e^{i\pi t2} & 0 \\ 0 & e^{i\pi t1} \end{pmatrix}$
$\begin{pmatrix} -1 & 0 & 0 & 0 \\ 0 & -1 & 0 & 0 \\ 0 & 0 & 1 & 0 \end{pmatrix}$	$\begin{pmatrix} -i & 0 \\ 0 & i \end{pmatrix}$	{2_{001}|0,0,0}	$\begin{pmatrix} 1 & 0 \\ 0 & 1 \end{pmatrix}$	$\begin{pmatrix} -1 & 0 \\ 0 & -1 \end{pmatrix}$	$\begin{pmatrix} -i & 0 \\ 0 & i \end{pmatrix}$	$\begin{pmatrix} i & 0 \\ 0 & -i \end{pmatrix}$
$\begin{pmatrix} 1 & 0 & 0 & 0 \\ 0 & 1 & 0 & 0 \\ 0 & 0 & 1 & 0 \end{pmatrix}$	$\begin{pmatrix} -1 & 0 \\ 0 & -1 \end{pmatrix}$	{$^d1$|0,0,0}	$\begin{pmatrix} 1 & 0 \\ 0 & 1 \end{pmatrix}$	$\begin{pmatrix} 1 & 0 \\ 0 & 1 \end{pmatrix}$	$\begin{pmatrix} -1 & 0 \\ 0 & -1 \end{pmatrix}$	$\begin{pmatrix} -1 & 0 \\ 0 & -1 \end{pmatrix}$
$\begin{pmatrix} -1 & 0 & 0 & 0 \\ 0 & -1 & 0 & 0 \\ 0 & 0 & 1 & 0 \end{pmatrix}$	$\begin{pmatrix} i & 0 \\ 0 & -i \end{pmatrix}$	{$^d2_{001}$|0,0,0}	$\begin{pmatrix} 1 & 0 \\ 0 & 1 \end{pmatrix}$	$\begin{pmatrix} -1 & 0 \\ 0 & -1 \end{pmatrix}$	$\begin{pmatrix} i & 0 \\ 0 & -i \end{pmatrix}$	$\begin{pmatrix} -i & 0 \\ 0 & i \end{pmatrix}$
$\begin{pmatrix} 0 & -1 & 0 & 0 \\ 1 & 0 & 0 & 0 \\ 0 & 0 & 1 & 0 \end{pmatrix}'$	$\begin{pmatrix} (1-i)\sqrt{2}/2 & 0 \\ 0 & (1+i)\sqrt{2}/2 \end{pmatrix}$	{$4'^{+}_{001}$|0,0,0}	$\begin{pmatrix} 0 & 1 \\ 1 & 0 \end{pmatrix}$	$\begin{pmatrix} 0 & -1 \\ 1 & 0 \end{pmatrix}$	$\begin{pmatrix} 0 & i \\ 1 & 0 \end{pmatrix}$	$\begin{pmatrix} 0 & -i \\ 1 & 0 \end{pmatrix}$
$\begin{pmatrix} 0 & 1 & 0 & 0 \\ -1 & 0 & 0 & 0 \\ 0 & 0 & 1 & 0 \end{pmatrix}'$	$\begin{pmatrix} (1+i)\sqrt{2}/2 & 0 \\ 0 & (1-i)\sqrt{2}/2 \end{pmatrix}$	{$4'^{-}_{001}$|0,0,0}	$\begin{pmatrix} 0 & 1 \\ 1 & 0 \end{pmatrix}$	$\begin{pmatrix} 0 & 1 \\ -1 & 0 \end{pmatrix}$	$\begin{pmatrix} 0 & -1 \\ -i & 0 \end{pmatrix}$	$\begin{pmatrix} 0 & -1 \\ i & 0 \end{pmatrix}$
$\begin{pmatrix} 0 & -1 & 0 & 0 \\ 1 & 0 & 0 & 0 \\ 0 & 0 & 1 & 0 \end{pmatrix}'$	$\begin{pmatrix} -(1-i)\sqrt{2}/2 & 0 \\ 0 & -(1+i)\sqrt{2}/2 \end{pmatrix}$	{$^d4'^{+}_{001}$|0,0,0}	$\begin{pmatrix} 0 & 1 \\ 1 & 0 \end{pmatrix}$	$\begin{pmatrix} 0 & -1 \\ 1 & 0 \end{pmatrix}$	$\begin{pmatrix} 0 & -i \\ -1 & 0 \end{pmatrix}$	$\begin{pmatrix} 0 & i \\ -1 & 0 \end{pmatrix}$
$\begin{pmatrix} 0 & 1 & 0 & 0 \\ -1 & 0 & 0 & 0 \\ 0 & 0 & 1 & 0 \end{pmatrix}'$	$\begin{pmatrix} -(1+i)\sqrt{2}/2 & 0 \\ 0 & -(1-i)\sqrt{2}/2 \end{pmatrix}$	{$^d4'^{-}_{001}$|0,0,0}	$\begin{pmatrix} 0 & 1 \\ 1 & 0 \end{pmatrix}$	$\begin{pmatrix} 0 & 1 \\ -1 & 0 \end{pmatrix}$	$\begin{pmatrix} 0 & 1 \\ i & 0 \end{pmatrix}$	$\begin{pmatrix} 0 & 1 \\ -i & 0 \end{pmatrix}$

Supplementary Figure 10: The output of the Corepresentations tool for the X point in Type-III MSG 75.3 $P4'$ [SEq. (87)], part 2. The table shown in this figure contains the *full* (SSG) coreps of $G = P4'$ in the star containing $\mathbf{k}_X$ $[(0,\pi,0),(\pi,0,0)]$, see SEqs. (87) and (88)]. For one coset representative in each of the little group cosets in SEq. (65), as well as the SSG symmetries $G \setminus G_X$ [SEq. (79) and the surrounding text], Corepresentations outputs the matrix representatives in each of the irreducible full (co)reps of G in the star indexed by a point $\mathbf{k}$. In the table shown in this figure, the matrix representatives of antiunitary symmetries g_A are labeled in red text, and the matrices listed for each full (co)rep $\tilde{\Sigma}_{\mathbf{k}}$ indicate the unitary part U of the antiunitary matrix representative $\Delta_{\tilde{\Sigma}}(g_A) = UK$, where K is complex conjugation. Each of the full coreps for $G = P4'$ in the star of $\mathbf{k}_X$ is labeled with both (X) as well as B_iY_j, to indicate that the small coreps in each arm $\mathbf{k}_X$ and $\mathbf{k}_{XA}$ in G are respectively equivalent to the small irreps at $\mathbf{k}_B$ and $\mathbf{k}_Y$ in Type-I MSG 3.1 $P2$ [see SFigs. 8 and 9 and the text surrounding SEqs. (63) and (61)]. For each $g \in G$, each $\Delta_{\tilde{\Sigma}_X}(g)$ shown in this figure is a 2×2 matrix, because each small corep $\tilde{\sigma}_X$ in SFig. 9 is one-dimensional. Additionally, each $\Delta_{\tilde{\Sigma}_X}(g)$ is diagonal for each symmetry $g \in G_X$, $g \in G_{XA}$ [*e.g.* $\{C_{2z}|000\}$], but is non-diagonal for each symmetry $g \notin G_X$ or $g \notin G_{XA}$ [*e.g.* $\{C_{4z} \times \mathcal{T}|000\}$].

15. Small and Full Coreps at the S Point in Type-IV MSG 25.63 P_Cmm2

In this section, we will determine the small coreps of the little group G_S of the S point in Type-IV MSG 25.63 P_Cmm2. We will also show that the small coreps of G_S coincide with the full coreps induced in $G = P_Cmm2$ in the momentum star of S, because the S point in Type-IV MSG 25.63 P_Cmm2 is the only arm of a multiplicity-1 star (see SN 12 and the MKVEC tool for more information). To begin, MSG 25.63 P_Cmm2 is generated by:

$$M_x = \left\{ m_x \middle| 000 \right\},\ M_y = \left\{ m_y \middle| 000 \right\},\ \theta = \left\{ \mathcal{T} \middle| \frac{1}{2}\frac{1}{2}0 \right\},\ t_x = \{E|100\},\ t_z = \{E|001\}. \tag{94}$$

The S point in MSG 25.63 P_Cmm2 lies at:

$$\mathbf{k}_S = 2\pi(1/2, 1/2, 0). \tag{95}$$

Unlike in the previous example in SN 14, all of the symmetries in MSG 25.63 P_Cmm2 return $\mathbf{k}_S$ to itself modulo reciprocal lattice vectors ($g\mathbf{k}_S \equiv \mathbf{k}_S$ for all $g \in P_Cmm2$). Therefore, the little group G_S is isomorphic to MSG 25.63 P_Cmm2 itself, and the set $\tilde{G}_S$ [defined in the text surrounding SEq. (71)] is given by

$$G_S = \tilde{G}_S G_T = \left(\tilde{H}_S \cup \theta \tilde{H}_S \right) G_T. \tag{96}$$

with $\tilde{g}_A = \theta$. In SEq. (96), the maximal unitary subset of $\tilde{G}_S$ is given by $\tilde{H}_S$ [in the specific case of the S point in Type-IV MSG 25.63 P_Cmm2, $\tilde{H}_S$ is in fact a finite group, see the text preceding SEq. (71) for more information]:

$$\bar{H}_S = \left\{ \{E|\mathbf{0}\},\ \{m_x|\mathbf{0}\},\ \{m_y|\mathbf{0}\},\ \{C_{2z}|\mathbf{0}\},\ \{\bar{E}|\mathbf{0}\},\ \{\bar{E}m_x|\mathbf{0}\},\ \{\bar{E}m_y|\mathbf{0}\},\ \{\bar{E}C_{2z}|\mathbf{0}\} \right\}, \tag{97}$$

where $\bar{E}$ is defined in the text following SEq. (93). The symmetry operations in $\tilde{H}_S$ in SEq. (97) satisfy:

$$\begin{aligned} m_{x,y} m_{y,x} m_{x,y}^{-1} &= \bar{E} m_{y,x},\ m_{x,y} C_{2z} m_{x,y}^{-1} = \bar{E} C_{2z},\ m_{x,y} \bar{E} m_{x,y}^{-1} = C_{2z} \bar{E} C_{2z}^{-1} = \bar{E}, \\ m_x m_y &= C_{2z},\ \bar{E}^2 = E,\ m_{x,y}^2 = C_{2z}^2 = \bar{E}. \end{aligned} \tag{98}$$

Because all of the symmetries $h \in \tilde{H}_S$ are of the form $\{R|\mathbf{0}\}$, then SEqs. (97) and (98) imply that the small irreps of H_S are equivalent to the irreps of an abstract finite group [see SRef. 33 and the text following SEq. (64)] that is isomorphic[7–18] to Type-I MPG 7.1.20 $mm2$, which has five irreps σ. In Supplementary Table 2, we reproduce the matrix representatives $\Delta_\sigma(h)$ of the small irreps of H_S from the output of the Corepresentations tool for the S point in Type-I MSG 25.57 $Pmm2$, which is the unitary subgroup of Type-IV MSG 25.63 P_Cmm2 (adjusting for the differences in convention between how translations are represented in this work and on the BCS, see SFig. 9). The five irreps in Supplementary Table 2 subdivide into four single-valued, one-dimensional irreps (S_{1-4}) that are distinguished by their spinless $m_{x,y}$ eigenvalues and one double-valued irrep ($\bar{S}_5$) that is two-dimensional because of the anticommutator $\{\Delta_{\bar{S}_5}(\{m_x|\mathbf{0}\}), \Delta_{\bar{S}_5}(\{m_y|\mathbf{0}\})\} = 0$.

To determine the type of the small corep $\tilde{\sigma}$ induced in G_S, we calculate the indicator $J_\sigma = \sum_i \chi_\sigma(g_{A,i}^2)$ [SEqs. (76) and (77)] for each irrep σ in Supplementary Table 2. Using SEq. (96), we determine that there are eight $g_{A,i}$ to consider:

$$g_{A,i} \in \theta \tilde{H}_S, \tag{99}$$

where θ is defined in SEq. (94), and where $\tilde{H}_S$ is defined in SEq. (97) and in Supplementary Table 2. First, we use SEqs. (97) and (98) to determine that $\theta = t_x t_y \bar{E} \theta^{-1}$, $\bar{E}^2 = E$, and that $[\bar{E}, h_i] = 0$ for all $h_i \in \tilde{H}_S$, where $t_x = \{E|100\}$ and $t_y = \{E|010\}$. We then determine that, in the case of $\tilde{G}_S$ in Type-IV MSG 25.63 P_Cmm2, $\chi_\sigma(g_{A,i}^2)$ can be simplified as:

$$\chi_\sigma(g_{A,i}^2) = \chi_\sigma(\theta h_i \theta h_i) = \mathrm{sgn}\left[\chi_\sigma(\bar{E}) \right] \chi_\sigma\left([\theta h_i \theta^{-1} t_x t_y] h_i \right), \tag{100}$$

where $h_i \in \tilde{H}_S$ in SEq. (97). Next, we use SEq. (94) to obtain the relations:

$$\begin{aligned}
\theta C_{2z}\theta^{-1}t_x t_y &= \theta\{C_{2z}|000\}\theta^{-1}\{E|110\} = \{C_{2z}|110\}\{E|110\} = (t_x t_y C_{2z})t_x t_y = (C_{2z}t_x^{-1}t_y^{-1})t_x t_y = C_{2z}, \\
\theta M_x\theta^{-1}t_x t_y &= \theta\{m_x|000\}\theta^{-1}\{E|110\} = \{m_x|100\}\{E|110\} = (t_x M_x)t_x t_y = (M_x t_x^{-1})t_x t_y = t_y M_x, \\
\theta M_y\theta^{-1}t_x t_y &= \theta\{m_y|000\}\theta^{-1}\{E|110\} = \{m_y|010\}\{E|110\} = (t_y M_y)t_x t_y = (M_y t_y^{-1})t_x t_y = t_x M_y, \\
\theta\bar{E}\theta^{-1} &= \theta\{\bar{E}|000\}\theta^{-1} = \{\bar{E}|000\} = \bar{E}.
\end{aligned} \tag{101}$$

SEqs. (100) and (101) imply that J_σ [SEq. (76)] can be further simplified before specifying a value of σ:

$$\begin{aligned}
J_\sigma &= \sum_i \chi_\sigma(g_{A,i}^2) = \text{sgn}\left[\chi_\sigma(\bar{E})\right]\sum_i \chi_\sigma\left([\theta h_i\theta^{-1}t_x t_y]h_i\right) \\
&= 2\,\text{sgn}\left[\chi_\sigma(\bar{E})\right]\left(\chi_\sigma(E) - \chi_\sigma(\bar{E})\right) \\
&= 2\left[\chi_\sigma(\bar{E}) - \chi_\sigma(E)\right].
\end{aligned} \tag{102}$$

Matrix Representatives $\Delta_\sigma(h)$ of the Small Irreps of H_S at the S point [$\mathbf{k}_S = (\pi,\pi,0)$] in Type-I MSG 25.57 $Pmm2$, the Unitary Subgroup of Type-IV MSG 25.63 P_Cmm2					
h	S_1	S_2	S_3	S_4	$\bar{S}_5$
$\{E\|\mathbf{t}_1+\mathbf{t}_2+\mathbf{t}_3\}$	$e^{-i\pi(\mathbf{t}_1+\mathbf{t}_2)}$	$e^{-i\pi(\mathbf{t}_1+\mathbf{t}_2)}$	$e^{-i\pi(\mathbf{t}_1+\mathbf{t}_2)}$	$e^{-i\pi(\mathbf{t}_1+\mathbf{t}_2)}$	$\begin{pmatrix} e^{-i\pi(\mathbf{t}_1+\mathbf{t}_2)} & 0 \\ 0 & e^{-i\pi(\mathbf{t}_1+\mathbf{t}_2)} \end{pmatrix}$
$\{C_{2z}\|000\}$	1	1	-1	-1	$\begin{pmatrix} 0 & -1 \\ 1 & 0 \end{pmatrix}$
$\{m_y\|000\}$	1	-1	-1	1	$\begin{pmatrix} 0 & -i \\ -i & 0 \end{pmatrix}$
$\{m_x\|000\}$	1	-1	1	-1	$\begin{pmatrix} -i & 0 \\ 0 & i \end{pmatrix}$
$\{\bar{E}\|000\}$	1	1	1	1	$\begin{pmatrix} -1 & 0 \\ 0 & -1 \end{pmatrix}$
$\{\bar{E}C_{2z}\|000\}$	1	1	-1	-1	$\begin{pmatrix} 0 & 1 \\ -1 & 0 \end{pmatrix}$
$\{\bar{E}m_y\|000\}$	1	-1	-1	1	$\begin{pmatrix} 0 & i \\ i & 0 \end{pmatrix}$
$\{\bar{E}m_x\|000\}$	1	-1	1	-1	$\begin{pmatrix} i & 0 \\ 0 & -i \end{pmatrix}$

Supplementary Table 2: The matrix representatives $\Delta_\sigma(h)$ of the small irreps σ of the little group H_S of the S point [$\mathbf{k}_S = (\pi,\pi,0)$] in Type-I MSG 25.57 $Pmm2$, the unitary subgroup of Type-IV MSG 25.63 P_Cmm2. Because MSG 25.57 $Pmm2$ is a Type-I MSG (SN 3), then $\tilde{H}_S$ is isomorphic to its maximal unitary subset. The values of $\Delta_\sigma(h)$ in this table have been reproduced from the output of the Corepresentations tool, and adapted to the notation employed throughout this work in which a translation $\mathbf{t}$ is represented at a crystal momentum $\mathbf{k}$ by $\exp(-i\mathbf{k}\cdot\mathbf{t})$ [*i.e.*, in reduced units in which the lattice constants $a,b,c = 1$, see SFig. 9 for further details]. We note that in Corepresentations and in this table, the matrix representatives $\Delta_\sigma(h)$ are shown for each symmetry $h \in \tilde{H}_\mathbf{k}$ except for the element $\{E|000\}$ with $\exp(-i\mathbf{k}_S\cdot\mathbf{t}_\mu) = 1$ [see SEqs. (66) and (67)]; instead the first element h in this table, and in the output of Corepresentations, is chosen to be $\{E|\mathbf{t}_1+\mathbf{t}_2+\mathbf{t}_3\}$, where $\mathbf{t}_{1,2,3}$ are respectively integer-valued multiples of the lattice vectors $\mathbf{t}_{x,y,z}$ (see SFigs. 9 and 11). We make this substitution of $\{E|\mathbf{t}_1+\mathbf{t}_2+\mathbf{t}_3\}$ for $\{E|000\}$ to provide users with information regarding the representations (phases) of translations at $\mathbf{k}$ (here specifically at $\mathbf{k}_S$ [SEq. (95)]), which contribute towards determining the matrix representatives of all of the symmetries in $H_\mathbf{k}$ (as opposed to just the symmetries in $\tilde{H}_\mathbf{k}$), and towards determining the pairing of unitary subgroup small irreps into little group small coreps [see the text surrounding SEqs. (101) and (102), for example]. The overbar on $\sigma = \bar{S}_5$ is used to indicate that $\bar{S}_5$ is double-valued, whereas the irreps without overbars (S_{1-4}) are single-valued.

Remarkably, we find that SEq. (102) only depends on whether σ is single- or double-valued:

$$J_\sigma = \begin{cases} 0, & \text{for } \sigma = S_{1-4} \\ -|\tilde{H}_S|, & \text{for } \sigma = \bar{S}_5 \end{cases}, \tag{103}$$

where $|\tilde{H}_S| = 8$ [SEq. (97)]. Using SEq. (77), we determine that the single-valued, one-dimensional irreps S_{1-4} induce paired, two-dimensional coreps of type (c) [SEq. (75)], whereas the double-valued, two-dimensional irrep $\bar{S}_5$ induces a paired, four-dimensional corep of type (b) [SEq. (74)].

To complete the calculation of the small coreps of G_S in Type-IV MSG 25.63 P_Cmm2, we must determine which of the single-valued irreps S_{1-4} become paired into coreps of type (c). This can be accomplished by computing the matrix representative $\bar{\Delta}_\sigma(h) = \left[\Delta_\sigma(\tilde{g}_A^{-1}h\tilde{g}_A)\right]^*$ [SEq. (72)]. Choosing $g_A = \theta$ and using SEq. (101), we find that, for the single-valued irreps $\sigma = S_{1-4}$:

$$\bar{\Delta}_\sigma(C_{2z}) = \Delta_\sigma(C_{2z}) = \Delta_{\sigma'}(C_{2z}),\ \bar{\Delta}_\sigma(M_{x,y}) = -\Delta_\sigma(M_{x,y}) = \Delta_{\sigma'}(M_{x,y}). \tag{104}$$

Along with SEq. (103), which implies that $\bar{S}_5$ induces a paired corep of type (b), SEq. (104) implies that G_S in Type-IV MSG 25.63 P_Cmm2 has three small coreps:

$$\tilde{\sigma} = S_1S_2,\ S_3S_4,\ \bar{S}_5\bar{S}_5, \tag{105}$$

where S_1S_2 and S_3S_4 are single-valued, two-dimensional coreps and $\bar{S}_5\bar{S}_5$ is a double-valued, four-dimensional corep. Below, we will shortly formulate a $k \cdot p$ Hamiltonian demonstrating that $\bar{S}_5\bar{S}_5$ corresponds to a 3D fourfold Dirac fermion[74,100] that is enforced by spinful mirrors that anticommute with each other $\{\Delta_{\bar{S}_5\bar{S}_5}(M_x), \Delta_{\bar{S}_5\bar{S}_5}(M_y)\} = 0$, and with the matrix representative of θ $\{\Delta_{\bar{S}_5\bar{S}_5}(M_{x,y}), \Delta_{\bar{S}_5\bar{S}_5}(\theta)\} = 0$.

In SFig. 11, we show the output of the Corepresentations tool for the S point in Type-IV MSG 25.63 P_Cmm2, which agrees with the calculation performed in this section to obtain SEq. (105). As previously discussed in SFig. 9 and in the text surrounding SEq. (88), the table in SFig. 11 contains the matrix representatives of the small coreps $\tilde{\sigma}$ [SEq. (105)] of the little group G_S in Type-IV MSG 25.63 P_Cmm2. We note that, like in SFig. 10, the Corepresentations tool also outputs a second table containing the matrix representatives of the full coreps in the momentum star indexed by $\mathbf{k}_S$ [see SN 12 and the text surrounding SEqs. (83) and (95)]. However, because, $\mathbf{k}_S$ in MSG 25.63 P_Cmm2 is the only arm of a multiplicty-1 momentum star (SN 12 and MKVEC), then the second table outputted by Corepresentations is identical to the table shown in SFig. 11. Therefore, for concision, we have omitted the second table outputted by Corepresentations.

We can gain some physical intuition for the small coreps $\tilde{\sigma}$ in SEq. (105) by forming a $k \cdot p$ Hamiltonian characterized by one of the $\tilde{\sigma}$. Focusing on the double-valued, four-dimensional corep $\tilde{\sigma} = \bar{S}_5\bar{S}_5$, which characterizes spinful electronic states, we can re-express the symmetry representation of the generating elements of G_S in Supplementary Table 2 and SEq. (105) as acting on a four-band Hamiltonian $\mathcal{H}(\mathbf{q}) = \mathcal{H}(\mathbf{k} - \mathbf{k}_S)$:

$$\begin{aligned} M_x\mathcal{H}(q_x,q_y,q_z)M_x^{-1} &= \tau^z\sigma^x\mathcal{H}(-q_x,q_y,q_z)\tau^z\sigma^x, \\ M_y\mathcal{H}(q_x,q_y,q_z)M_y^{-1} &= \tau^z\sigma^y\mathcal{H}(q_x,-q_y,q_z)\tau^z\sigma^y, \\ \theta\mathcal{H}(q_x,q_y,q_z)\theta^{-1} &= \tau^x\sigma^y\mathcal{H}^*(-q_x,-q_y,-q_z)\tau^x\sigma^y, \end{aligned} \tag{106}$$

where τ^i and σ^j are 2×2 Pauli matrices, and where we have employed a shorthand in which $\tau^i\sigma^j = \tau^i \otimes \sigma^j$, $\tau^0 \otimes \sigma^j = \sigma^j$, and $\tau^i \otimes \sigma^0 = \tau^i$. We note that we have not included the generating translations of G_S in SEq. (106), because translations are represented as phases in momentum space, and therefore do not by themselves impose constraints on $\mathcal{H}(q_x,q_y,q_z)$. The symmetry representation in SEq. (106) admits a Hamiltonian:

$$\mathcal{H}(\mathbf{q}) = [v_{1x}\sigma^y + v_{2x}\tau^x\sigma^x]q_x + [v_{1y}\sigma^x + v_{2y}\tau^x\sigma^y]q_y + [v_{1z}\tau^z + v_{2z}\tau^x\sigma^z]q_z, \tag{107}$$

that characterizes a linearly dispersing, fourfold condensed matter Dirac fermion with non-degenerate bands away from $\mathbf{q} = \mathbf{0}$. Specifically, in the $q_z = 0$ plane, SEq. (107) coincides with the Hamiltonian of the 2D filling-enforced[73,101] fourfold magnetic Dirac fermion introduced in SRef. 74. Most recently, the methods employed in this section – which we have adapted from SRefs. 74,100,102–107 – were used by the authors of SRef. 72 to construct a complete list of high-symmetry-point multifold fermions in the MSGs. Where there is overlap, the results of SRef. 72 agree with the output of the Corepresentations tool introduced in this work.

Irreducible co-representations of the (Double) Magnetic Space Group P_Cmm2 (No. 25.63) and wave-vector S:(1/2,1/2,0)

Unitary (Double) Space Group: *Pmm2* (No. 25) in its standard setting.

Coordinates of the wave-vector in the standard setting of the unitary subgroup: S:(1/2,1/2,0)

Magnetic little co-group of the wave-vector: *mm*21'

Little co-group of the wave-vector in the unitary subgroup: *mm*2

Irreducible co-representations of the magnetic little group.

The matrices of the representations (the whole representation and the representation of the little group) with dimension smaller than 5 are given explicitly.
When the dimension of the representation is larger than 5, only the non-zero elements are given using the following notation: (i;j)=x means that the (i,j) element of the matrix is x.

**Matrix presentation** (In red color the antiunitary operations)		**Seitz Symbol** (In red color the antiunitary operations)	S_1S_2	S_3S_4	$\overline{S}_5\overline{S}_5$
$\begin{pmatrix} 1 & 0 & 0 & t1 \\ 0 & 1 & 0 & t2 \\ 0 & 0 & 1 & t3 \end{pmatrix}$	$\begin{pmatrix} 1 & 0 \\ 0 & 1 \end{pmatrix}$	{1|t1,t2,t3}	$\begin{pmatrix} e^{i\pi(t1+t2)} & 0 \\ 0 & e^{-i\pi(t1+t2)} \end{pmatrix}$	$\begin{pmatrix} e^{i\pi(t1+t2)} & 0 \\ 0 & e^{-i\pi(t1+t2)} \end{pmatrix}$	$\begin{pmatrix} e^{i\pi(t1+t2)} & 0 & 0 & 0 \\ 0 & e^{i\pi(t1+t2)} & 0 & 0 \\ 0 & 0 & e^{-i\pi(t1+t2)} & 0 \\ 0 & 0 & 0 & e^{-i\pi(t1+t2)} \end{pmatrix}$
$\begin{pmatrix} -1 & 0 & 0 & 0 \\ 0 & -1 & 0 & 0 \\ 0 & 0 & 1 & 0 \end{pmatrix}$	$\begin{pmatrix} -i & 0 \\ 0 & i \end{pmatrix}$	$\{2_{001}\|0,0,0\}$	$\begin{pmatrix} 1 & 0 \\ 0 & 1 \end{pmatrix}$	$\begin{pmatrix} -1 & 0 \\ 0 & -1 \end{pmatrix}$	$\begin{pmatrix} 0 & -1 & 0 & 0 \\ 1 & 0 & 0 & 0 \\ 0 & 0 & 0 & -1 \\ 0 & 0 & 1 & 0 \end{pmatrix}$
$\begin{pmatrix} 1 & 0 & 0 & 0 \\ 0 & -1 & 0 & 0 \\ 0 & 0 & 1 & 0 \end{pmatrix}$	$\begin{pmatrix} 0 & -1 \\ 1 & 0 \end{pmatrix}$	$\{m_{010}\|0,0,0\}$	$\begin{pmatrix} 1 & 0 \\ 0 & -1 \end{pmatrix}$	$\begin{pmatrix} -1 & 0 \\ 0 & 1 \end{pmatrix}$	$\begin{pmatrix} 0 & -i & 0 & 0 \\ -i & 0 & 0 & 0 \\ 0 & 0 & 0 & -i \\ 0 & 0 & -i & 0 \end{pmatrix}$
$\begin{pmatrix} 1 & 0 & 0 & 1/2 \\ 0 & 1 & 0 & 1/2 \\ 0 & 0 & 1 & 0 \end{pmatrix}^{\prime}$	$\begin{pmatrix} 1 & 0 \\ 0 & 1 \end{pmatrix}$	$\{1'\|1/2,1/2,0\}$	$\begin{pmatrix} 0 & 1 \\ 1 & 0 \end{pmatrix}$	$\begin{pmatrix} 0 & 1 \\ 1 & 0 \end{pmatrix}$	$\begin{pmatrix} 0 & 0 & -1 & 0 \\ 0 & 0 & 0 & -1 \\ 1 & 0 & 0 & 0 \\ 0 & 1 & 0 & 0 \end{pmatrix}$
$\begin{pmatrix} -1 & 0 & 0 & 1/2 \\ 0 & -1 & 0 & 1/2 \\ 0 & 0 & 1 & 0 \end{pmatrix}^{\prime}$	$\begin{pmatrix} -i & 0 \\ 0 & i \end{pmatrix}$	$\{2'_{001}\|1/2,1/2,0\}$	$\begin{pmatrix} 0 & 1 \\ 1 & 0 \end{pmatrix}$	$\begin{pmatrix} 0 & -1 \\ -1 & 0 \end{pmatrix}$	$\begin{pmatrix} 0 & 0 & 0 & 1 \\ 0 & 0 & -1 & 0 \\ 0 & -1 & 0 & 0 \\ 1 & 0 & 0 & 0 \end{pmatrix}$
$\begin{pmatrix} 1 & 0 & 0 & 1/2 \\ 0 & -1 & 0 & 1/2 \\ 0 & 0 & 1 & 0 \end{pmatrix}^{\prime}$	$\begin{pmatrix} 0 & -1 \\ 1 & 0 \end{pmatrix}$	$\{m'_{010}\|1/2,1/2,0\}$	$\begin{pmatrix} 0 & -1 \\ 1 & 0 \end{pmatrix}$	$\begin{pmatrix} 0 & 1 \\ -1 & 0 \end{pmatrix}$	$\begin{pmatrix} 0 & 0 & 0 & -i \\ 0 & 0 & -i & 0 \\ 0 & i & 0 & 0 \\ i & 0 & 0 & 0 \end{pmatrix}$

Supplementary Figure 11: The output of the Corepresentations tool for the S point in Type-IV MSG 25.63 P_Cmm2. The table in this figure shows the matrix representatives of the small coreps $\tilde{\sigma}$ of the little group G_S [$\mathbf{k}_S = (\pi,\pi,0)$], for which the coreps with (without) overbars are double- (single-) valued. As discussed in SFig. 9, throughout this work, a translation $\mathbf{t}$ is represented at a crystal momentum $\mathbf{k}$ by $\exp(-i\mathbf{k}\cdot\mathbf{t})$ [*i.e.*, in reduced units in which the lattice constants $a,b,c=1$], whereas on the BCS, $\mathbf{t}$ is represented at $\mathbf{k}$ by the phase $\exp(2\pi i\mathbf{k}\cdot\mathbf{t})$ [*i.e.* with the opposite sign as employed in this work, and in different reduced units in which $\mathbf{t}$ and $\mathbf{k}$ are respectively expressed as multiples of the lattice and reciprocal lattice constants a,b,c and $2\pi/(a,b,c)$]. In the table shown in this figure, the matrix representatives of antiunitary symmetries g_A are labeled in red text, and the matrices listed for each small corep $\tilde{\sigma}_S$ indicate the unitary part U of the antiunitary matrix representative $\Delta_{\tilde{\sigma}_S}(g_A) = UK$, where K is complex conjugation. We note that the output of Corepresentations for the S point in Type-IV MSG 25.63 P_Cmm2 also includes an additional table containing the matrix representatives of the full coreps in the momentum star containing $\mathbf{k}_S$ (see SFig. 10). However, because, $\mathbf{k}_S$ in MSG 25.63 P_Cmm2 is the only arm of a multiplicty-1 momentum star (SN 12 and MKVEC), then the second table outputted by Corepresentations is identical to the table shown in this figure; for concision have therefore omitted the second table outputted by Corepresentations.

16. Compatibility Relations in the MSGs and the MCOMPREL Tool

In this section, building upon the definition of the small coreps of the magnetic little groups established in SN 13, we will now discuss the concept of compatibility relations (defined in detail in the text below), which relate the coreps at different $\mathbf{k}$ points throughout the BZ. To begin, at a given point $\mathbf{k}$ in the first BZ of an SSG, the set of occupied Bloch eigenstates can be labeled by the small coreps of the little group $G_{\mathbf{k}}$. As shown in previous works[1–6,11,12,20–22,83–86], given knowledge of all of the coreps at $\mathbf{k}$, the possible coreps present at a point $\mathbf{k}'$ that is *connected* to $\mathbf{k}$ [defined in the text following SEq. (58)] can be inferred from the group-subgroup relations between $G_{\mathbf{k}}$ and $G_{\mathbf{k}'}$. In this section, we will review how the compatibility relations between the coreps at connected $\mathbf{k}$ points throughout the BZ can be reformulated using the language of graph theory. Finally, we will conclude this section by discussing how the graph-theory interpretation of the compatibility relations can be exploited to determine if a given set of coreps at a small number of high-symmetry $\mathbf{k}$ vectors [specifically, the arms of the maximal momentum stars, see the text surrounding SEq. (59)] are incompatible with the presence of an energy (band) gap at all $\mathbf{k}$ points in the BZ.

To begin, consider two connected points $\mathbf{k}$ and $\mathbf{k}'$ for which the little group $G_{\mathbf{k}}$ is of higher symmetry than the little group $G_{\mathbf{k}'}$, such that $G_{\mathbf{k}'} \subset G_{\mathbf{k}}$. Next, consider a set of occupied Bloch eigenstates to be present at $\mathbf{k}$. The Bloch states at $\mathbf{k}$ can be labeled with a small, *generically-reducible* corep $\tilde{\varsigma}_{\mathbf{k}}$ of $G_{\mathbf{k}}$:

$$\tilde{\varsigma}_{\mathbf{k}} = \bigoplus_i a_i^{\mathbf{k}} \tilde{\sigma}_{i,\mathbf{k}}, \tag{108}$$

where $\tilde{\sigma}_{i,\mathbf{k}}$ is the ith small (irreducible) corep of $G_{\mathbf{k}}$ (SN 13). In SEq. (108), $a_i^{\mathbf{k}}$ is a non-negative integer[108], known as the *multiplicity* of $\tilde{\sigma}_{i,\mathbf{k}}$, that indicates the number of times that $\tilde{\sigma}_{i,\mathbf{k}}$ appears in the decomposition of $\tilde{\varsigma}_{\mathbf{k}}$. The multiplicities $\{a_i^{\mathbf{k}}\}$ are known as the *symmetry data* for each $\mathbf{k}$ point, and the set $\{\tilde{\varsigma}_{\mathbf{k}}\}$ over all of the arms $\mathbf{k}$ of the maximal momentum stars in an SSG [defined in the text surrounding SEq. (59)] is known as the *symmetry data vector*[109]. In SEq. (108), each small corep $\tilde{\sigma}_{i,\mathbf{k}}$ can be further subduced onto the lower-symmetry little group $G_{\mathbf{k}'}$ of a point $\mathbf{k}'$ that is connected to $\mathbf{k}$:

$$\tilde{\sigma}_{i,\mathbf{k}} \downarrow G_{\mathbf{k}'} = \bigoplus_j m_{i,j}^{\mathbf{k},\mathbf{k}'} \tilde{\sigma}_{j,\mathbf{k}'}, \tag{109}$$

where $\tilde{\sigma}_{j,\mathbf{k}'}$ is the jth small (irreducible) corep of $G_{\mathbf{k}'}$ and $m_{i,j}^{\mathbf{k},\mathbf{k}'}$ is the multiplicity of $\tilde{\sigma}_{j,\mathbf{k}'}$ in $\tilde{\sigma}_{i,\mathbf{k}} \downarrow G_{\mathbf{k}'}$. The values of $m_{i,j}^{\mathbf{k},\mathbf{k}'}$ are known as the *compatibility relations*[3,4,83–86] between $\tilde{\sigma}_{i,\mathbf{k}}$ and $\tilde{\sigma}_{j,\mathbf{k}'}$, and are required to be non-negative integers, because they originate from group-subgroup subduction [SEq. (109) and SRef. 108]. For future calculations, it will be useful to re-express SEqs. (108) and (109) as:

$$m^{\mathbf{k},\mathbf{k}'} \tilde{\boldsymbol{\varsigma}}_{\mathbf{k}} = \tilde{\boldsymbol{\varsigma}}_{\mathbf{k}'}, \tag{110}$$

in which $\tilde{\boldsymbol{\varsigma}}_{\mathbf{k}}$ ($\tilde{\boldsymbol{\varsigma}}_{\mathbf{k}'}$) is an $w \times 1$- ($z \times 1$-) dimensional column vector where w (z) is the number of small coreps of $G_{\mathbf{k}}$ ($G_{\mathbf{k}'}$). In the notation of SEq. (110), $\tilde{\boldsymbol{\varsigma}}_{\mathbf{k}}$ and $\tilde{\boldsymbol{\varsigma}}_{\mathbf{k}'}$ contain symmetry data [*i.e.* the multiplicities $a_i^{\mathbf{k}}$ in SEq. (108) and the corresponding multiplicities $a_j^{\mathbf{k}'}$ at $\mathbf{k}'$] indicating the number of Bloch wavefunctions that transform in the ith (j^{th}) small corep $\tilde{\sigma}_{i,\mathbf{k}}$ ($\tilde{\sigma}_{j,\mathbf{k}'}$) of $G_{\mathbf{k}}$ ($G_{\mathbf{k}'}$) in an energetically isolated group of Bloch states at $\mathbf{k}$ ($\mathbf{k}'$). Hence, in SEq. (110), $m^{\mathbf{k},\mathbf{k}'}$ is a $z \times w$-dimensional matrix whose entries are the compatibility relations $m_{i,j}^{\mathbf{k},\mathbf{k}'}$ in SEq. (109).

If $G_{\mathbf{k}}$ and $G_{\mathbf{k}'}$ are Type-I little groups in a Type-I MSG (SN 3), then the compatibility relations $m_{i,j}^{\mathbf{k},\mathbf{k}'}$ for any irrep pair $\tilde{\sigma}_{i,\mathbf{k}}$ and $\tilde{\sigma}_{j,\mathbf{k}'}$ at any pair of connected points $\mathbf{k}$ and $\mathbf{k}'$ can be obtained through the existing DCOMPREL program on the BCS (https://www.cryst.ehu.es/cgi-bin/cryst/programs/dcomprel.pl)[1–6]. However, if $G_{\mathbf{k}}$ or $G_{\mathbf{k}'}$ is isomorphic to an SSG with antiunitary symmetries (Type-II, III, or IV, SN 4, 5, and 6, respectively), then we must perform several additional steps to determine $m_{i,j}^{\mathbf{k},\mathbf{k}'}$. Specifically, if $G_{\mathbf{k}}$ is isomorphic to a Type-II, III, or IV SSG, then, for each small corep $\tilde{\sigma}_{i,\mathbf{k}}$ of $G_{\mathbf{k}}$, we first calculate the subduction:

$$\tilde{\sigma}_{i,\mathbf{k}} \downarrow H_{\mathbf{k}} = \bigoplus_l b_{i,l}^{\mathbf{k}} \sigma_{l,\mathbf{k}}, \tag{111}$$

where $H_{\mathbf{k}}$ is the maximal unitary subgroup of $G_{\mathbf{k}}$, $\sigma_{l,\mathbf{k}}$ is the lth small irrep of $H_{\mathbf{k}}$, and where each coefficient $b_{i,l}^{\mathbf{k}} = 0$, 1, or 2, depending on whether $\tilde{\sigma}_{i,\mathbf{k}}$ is a type (a), (b), or (c) small corep [respectively defined in the text surrounding SEqs. (73), (74), and (75)]. Specifically, if $\tilde{\sigma}_{i,\mathbf{k}}$ is a type (a) [(b)] corep, then, for each value of i, $b_{i,l}^{\mathbf{k}} = 1$ [$b_{i,l}^{\mathbf{k}} = 2$] for one value of l, and $b_{i,l}^{\mathbf{k}} = 0$ for all of the other values of l at fixed i; conversely, if $\tilde{\sigma}_{i,\mathbf{k}}$ is a type (c) corep, then $b_{i,l}^{\mathbf{k}} = 1$

for *two* values of l, and $b^{\mathbf{k}}_{i,l} = 0$ for all of the other values of l at fixed i (see SN 13). This occurs because, if $G_{\mathbf{k}}$ is isomorphic to a Type-II, III, or IV SSG, then $H_{\mathbf{k}}$ is necessarily an index-2 subgroup of $G_{\mathbf{k}}$ (see SN 4, 5, and 6), and because $G_{\mathbf{k}} = H_{\mathbf{k}} \cup g_A H_{\mathbf{k}}$ where g_A is an antiunitary symmetry $g_A \in G_{\mathbf{k}}$, $g_A \notin H_{\mathbf{k}}$. Hence, as shown in SN 13, each of the small coreps $\tilde{\sigma}_{i,\mathbf{k}}$ of $G_{\mathbf{k}}$ is either equivalent to a small irrep $\sigma_{l,\mathbf{k}}$ of $H_{\mathbf{k}}$ such that $b^{\mathbf{k}}_{i,l} = 1$ for only one value of l for each i [type (a) corep, see SEq. (73)], $\tilde{\sigma}_{i,\mathbf{k}}$ is equivalent to the direct sum $\sigma_{l,\mathbf{k}} \oplus \sigma_{l,\mathbf{k}}$ such that $b^{\mathbf{k}}_{i,l} = 2$ for only one value of l for each i [type (b) corep, see SEq. (74)], or $\tilde{\sigma}_{i,\mathbf{k}}$ is equivalent to the direct sum $\sigma_{l1,\mathbf{k}} \oplus \sigma_{l2,\mathbf{k}}$ such that $b^{\mathbf{k}}_{i,l} = 1$ for only two values $l = l1, l2$ for each i [type (c) corep, see SEq. (75)]. The values of $b^{\mathbf{k}}_{i,l}$ in SEq. (111) can be obtained from the Corepresentations tool introduced in this work, which we previously detailed in SN 13. In the notation of SEq. (110), SEq. (111) can be re-expressed as:

$$b^{\mathbf{k}} \tilde{\varsigma}_{\mathbf{k}} = \varsigma_{\mathbf{k}}, \tag{112}$$

in which $\varsigma_{\mathbf{k}}$ is an $x \times 1$-dimensional column vector whose l^{th} entry is the multiplicity of $\sigma_{l,\mathbf{k}}$ in $\tilde{\varsigma}_{\mathbf{k}} \downarrow H_{\mathbf{k}}$, where x is the number of small irreps of $H_{\mathbf{k}}$, and where $b^{\mathbf{k}}$ is a $x \times w$-dimensional matrix whose entries are $b^{\mathbf{k}}_{i,l}$ in SEq. (111). Next, for each small irrep $\sigma_{l,\mathbf{k}}$ of $H_{\mathbf{k}}$, we further subduce onto $H_{\mathbf{k}'}$, the maximal unitary subgroup of $G_{\mathbf{k}'}$:

$$\sigma_{l,\mathbf{k}} \downarrow H_{\mathbf{k}'} = \bigoplus_{s} n^{\mathbf{k},\mathbf{k}'}_{l,s} \sigma_{s,\mathbf{k}'}, \tag{113}$$

where $\sigma_{s,\mathbf{k}'}$ is the sth small irrep of $H_{\mathbf{k}'}$ and $n^{\mathbf{k},\mathbf{k}'}_{l,s}$ is the multiplicity of $\sigma_{s,\mathbf{k}'}$ in $\sigma_{l,\mathbf{k}} \downarrow H_{\mathbf{k}'}$. As with $m^{\mathbf{k},\mathbf{k}'}_{i,j}$ in SEq. (109), the values of $n^{\mathbf{k},\mathbf{k}'}_{l,s}$ in SEq. (113) are required to be non-negative integers, because they originate from group-subgroup subduction[108]. Crucially, because $H_{\mathbf{k}}$ and $H_{\mathbf{k}'}$ are both isomorphic to Type-I MSGs, then the compatibility relations $n^{\mathbf{k},\mathbf{k}'}_{l,s}$ for all possible connected points $\mathbf{k}$ and $\mathbf{k}'$ in all 1,651 SSGs can be determined using the earlier DCOMPREL tool, which is documented in SRef. 3. Following SEq. (110), SEq. (113) can be re-expressed as:

$$n^{\mathbf{k},\mathbf{k}'} \varsigma_{\mathbf{k}} = \varsigma_{\mathbf{k}'}, \tag{114}$$

in which $\varsigma_{\mathbf{k}'}$ is a $y \times 1$-dimensional column vector whose s^{th} entry is the multiplicity of $\sigma_{s,\mathbf{k}'}$ in $\tilde{\varsigma}_{\mathbf{k}'} \downarrow H_{\mathbf{k}'}$, where y is the number of small irreps of $H_{\mathbf{k}'}$, and where $n^{\mathbf{k},\mathbf{k}'}$ is an $y \times x$-dimensional matrix whose entries are the unitary subgroup compatibility relations $n^{\mathbf{k},\mathbf{k}'}_{l,s}$ in SEq. (113). As a last step towards calculating the compatibility relations $m^{\mathbf{k},\mathbf{k}'}_{i,j}$ in SEq. (109), we calculate the subduction onto $H_{\mathbf{k}'}$ for each small corep $\tilde{\sigma}_{j,\mathbf{k}'}$ of $G_{\mathbf{k}'}$:

$$\tilde{\sigma}_{j,\mathbf{k}'} \downarrow H_{\mathbf{k}'} = \bigoplus_{s} c^{\mathbf{k}'}_{j,s} \sigma_{s,\mathbf{k}'}, \tag{115}$$

where $\sigma_{s,\mathbf{k}'}$ is the sth small irrep of $H_{\mathbf{k}'}$, and where, as detailed in the text following SEq. (111), each coefficient $c^{\mathbf{k}'}_{j,s} = 0$, 1, or 2, depending on whether $\tilde{\sigma}_{j,\mathbf{k}'}$ is a type (a), (b), or (c) small corep [defined in the text surrounding SEqs. (73), (74), and (75), respectively]. As previously with $b^{\mathbf{k}}_{i,l}$ in SEq. (111), the values of $c^{\mathbf{k}'}_{j,s}$ in SEq. (115) can also be obtained from the Corepresentations tool introduced in this work (SN 13). Like SEq. (111), SEq. (115) can be re-expressed in the form of SEq. (112):

$$c^{\mathbf{k}'} \tilde{\varsigma}_{\mathbf{k}'} = \varsigma_{\mathbf{k}'}, \tag{116}$$

where $c^{\mathbf{k}'}$ is a $y \times z$-dimensional matrix whose entries are $c^{\mathbf{k}'}_{j,s}$ in SEq. (115). Finally, by combining SEqs. (110), (112), (114), and (116), we determine that:

$$c^{\mathbf{k}'} m^{\mathbf{k},\mathbf{k}'} = n^{\mathbf{k},\mathbf{k}'} b^{\mathbf{k}}. \tag{117}$$

To solve for $m^{\mathbf{k},\mathbf{k}'}$ in SEq. (117), we need to obtain a left inverse for $c^{\mathbf{k}'}$ [*i.e.* a matrix $(c^{\mathbf{k}'})^{-1}$ for which $(c^{\mathbf{k}'})^{-1} c^{\mathbf{k}'} = \mathbb{1}_z$], where $(c^{\mathbf{k}'})^{-1}$ is guaranteed to exist (though not necessarily be unique), because of Frobenius reciprocity[2,108]. Conversely, because $c^{\mathbf{k}'}$ in SEq. (116) is generically non-square and left-invertible, then a right inverse for $c^{\mathbf{k}'}$ does not generically also exist. Frobenius reciprocity specifically implies that we can obtain a left inverse for $c^{\mathbf{k}'}$ through *induction*:

$$\sigma_{s,\mathbf{k}'} \uparrow G_{\mathbf{k}'} = \bigoplus_{j} d^{\mathbf{k}'}_{s,j} \tilde{\sigma}_{j,\mathbf{k}'}, \tag{118}$$

where each coefficient $d^{\mathbf{k}'}_{s,j} = 0$ or 1, independent of whether $\tilde{\sigma}_{j,\mathbf{k}'}$ is a type (a), (b), or (c) small corep [defined in the text surrounding SEqs. (73), (74), and (75), respectively]. Specifically, because $G_{\mathbf{k}'} = H_{\mathbf{k}'} \cup g_A H_{\mathbf{k}'}$ where g_A is an antiunitary symmetry, then regardless of the type of the corep $\tilde{\sigma}_{j,\mathbf{k}'}$, $d^{\mathbf{k}'}_{s,j} = 1$ for one value of j, and $d^{\mathbf{k}'}_{s,j} = 0$ for all other values of j at fixed s (see SN 13). We next re-express SEq. (118) in the form of an inverse of SEq. (116):

$$\left(c^{\mathbf{k}'}\right)^{-1} \varsigma_{\mathbf{k}'} = \tilde{\varsigma}_{\mathbf{k}'}, \tag{119}$$

in which $(c^{\mathbf{k}'})^{-1}$ is the left inverse of $c^{\mathbf{k}'}$ and, crucially:

$$\left[\left(c^{\mathbf{k}'}\right)^{-1}\right]_{sj} = \frac{d^{\mathbf{k}'}_{s,j}}{[G_{\mathbf{k}'} : H_{\mathbf{k}'}]}, \tag{120}$$

where $[G_{\mathbf{k}'} : H_{\mathbf{k}'}]$ is the index of the subgroup $H^{\mathbf{k}'}$ of $G^{\mathbf{k}'}$ [SEq. (10)], which is present in SEq. (120) because induction ($\uparrow$), unlike subduction ($\downarrow$), *does not* preserve dimensionality (*i.e.*, the character of the identity element E)[2,108]. Therefore, independent of the SSG (little group) type of $G_{\mathbf{k}'}$, $(c^{\mathbf{k}'})^{-1}$ in SEq. (119) is necessarily well-defined, and its entries [SEq. (120)] are non-negative, though they are not necessarily integers. Specifically, if $G_{\mathbf{k}'}$ is isomorphic to a Type-II, III, or IV SSG (SN 4, 5, and 6, respectively), then $G_{\mathbf{k}'}$ is necessarily an index-2 supergroup of $H_{\mathbf{k}'}$, such that $[G_{\mathbf{k}'} : H_{\mathbf{k}'}] = 2$, implying that the elements $[(c^{\mathbf{k}'})^{-1}]_{sj}$ in SEq. (120) are non-negative multiples of 1/2. Nevertheless, we have verified that, for all connected little group pairs $G_{\mathbf{k}'} \subset G_{\mathbf{k}}$ in all 1,651 single and double SSGs, the elements of $m^{\mathbf{k},\mathbf{k}'}$ in the expression:

$$m^{\mathbf{k},\mathbf{k}'} = \left(c^{\mathbf{k}'}\right)^{-1} n^{\mathbf{k},\mathbf{k}'} b^{\mathbf{k}}, \tag{121}$$

formed from SEqs. (117), (118), (119), and (120) are non-negative integers, as required by subduction [see the text following SEq. (109)]. SEq. (121) implies that the multiplicities $b^{\mathbf{k}}_{i,l}$ and $c^{\mathbf{k}'}_{j,s}$ obtained from Corepresentations and the unitary subgroup compatibility relations $n^{\mathbf{k},\mathbf{k}'}_{l,s}$ obtained from DCOMPREL determine the compatibility relations $m^{\mathbf{k},\mathbf{k}'}_{i,j}$ between any two small coreps $\tilde{\sigma}_{i,\mathbf{k}}$ and $\tilde{\sigma}_{j,\mathbf{k}'}$ at any two connected points $\mathbf{k}$ and $\mathbf{k}'$ in any of the 1,651 SSGs. To simplify this procedure, we have implemented a new tool – MCOMPREL – through which the values of $m^{\mathbf{k},\mathbf{k}'}_{i,j}$ can be directly obtained without using additional programs on the BCS. Further specific details of the implementation of MCOMPREL are available in the documentation provided on the BCS.

We will now briefly present an example demonstrating the derivation of the multiplicities and compatibility relations at two connected $\mathbf{k}$ points for the double-valued small coreps of Type-III double MSG 83.45 $P4'/m$, which is generated by:

$$\{C_{4z} \times \mathcal{T}|000\},\ \{\mathcal{I}|000\},\ \{E|100\},\ \{E|001\}. \tag{122}$$

In this example, we will specifically obtain the small corep compatibility relations [SEq. (109)] for $G = P4'/m$ at the connected points:

$$\mathbf{k}_{\Gamma} = (0,0,0),\ \mathbf{k}_{LD} = (0,0,w). \tag{123}$$

First, using Corepresentations, we determine that the little group G_{Γ} is isomorphic to Type-III MSG 83.45 $P4'/m$, and has two, two-dimensional, double-valued small coreps $\tilde{\sigma}_{1,\Gamma}$ and $\tilde{\sigma}_{2,\Gamma}$, which are distinguished by their $\{\mathcal{I}|\mathbf{0}\}$ eigenvalues:

$$\chi_{\tilde{\sigma}_{1,\Gamma}}(\{\mathcal{I}|\mathbf{0}\}) = 2,\ \chi_{\tilde{\sigma}_{2,\Gamma}}(\{\mathcal{I}|\mathbf{0}\}) = -2. \tag{124}$$

Next, continuing to employ Corepresentations, we focus on the maximal unitary subgroup H_{Γ} of G_{Γ}. H_{Γ} is isomorphic to Type-I MSG 10.42 $P2/m$, and has *four*, one-dimensional, double-valued small irreps $\sigma_{1-4,\Gamma}$, which are also distinguished by their $\{\mathcal{I}|\mathbf{0}\}$ and $\{C_{2z}|\mathbf{0}\} = (\{C_{4z} \times \mathcal{T}|\mathbf{0}\})^6$ eigenvalues:

$$\begin{aligned}\chi_{\sigma_{1,\Gamma}}(\{\mathcal{I}|\mathbf{0}\}) &= 1,\ \chi_{\sigma_{2,\Gamma}}(\{\mathcal{I}|\mathbf{0}\}) = 1,\ \chi_{\sigma_{3,\Gamma}}(\{\mathcal{I}|\mathbf{0}\}) = -1,\ \chi_{\sigma_{4,\Gamma}}(\{\mathcal{I}|\mathbf{0}\}) = -1,\\ \chi_{\sigma_{1,\Gamma}}(\{C_{2z}|\mathbf{0}\}) &= -i,\ \chi_{\sigma_{2,\Gamma}}(\{C_{2z}|\mathbf{0}\}) = i,\ \chi_{\sigma_{3,\Gamma}}(\{C_{2z}|\mathbf{0}\}) = -i,\ \chi_{\sigma_{4,\Gamma}}(\{C_{2z}|\mathbf{0}\}) = i.\end{aligned} \tag{125}$$

We next subduce the small coreps $\tilde{\sigma}_{i,\Gamma}$ of G_Γ onto H_Γ [SEq. (111)]:

$$\tilde{\sigma}_{1,\Gamma} \downarrow H_\Gamma = \sigma_{1,\Gamma} \oplus \sigma_{2,\Gamma},\ \ \tilde{\sigma}_{2,\Gamma} \downarrow H_\Gamma = \sigma_{3,\Gamma} \oplus \sigma_{4,\Gamma}, \tag{126}$$

which may be summarized by introducing the multiplicity matrix [SEq. (112)]:

$$b^\Gamma = \begin{pmatrix} 1 & 0 \\ 1 & 0 \\ 0 & 1 \\ 0 & 1 \end{pmatrix}. \tag{127}$$

We then focus on the little group G_{LD}, which is isomorphic to Type-III MSG 75.3 $P4'$, and is generated by:

$$\{C_{4z} \times \mathcal{T}|000\},\ \{E|100\},\ \{E|001\}. \tag{128}$$

G_{LD} has only one, two-dimensional double-valued small corep $\tilde{\sigma}_{1,LD}$. The maximal unitary subgroup H_{DT} of G_{DT} is isomorphic to Type-I MSG 3.1 $P2$, and has two, one-dimensional, double-valued small irreps $\sigma_{1,LD}$ and $\sigma_{2,LD}$, which are distinguished by their $\{C_{2z}|\mathbf{0}\}$ eigenvalues:

$$\chi_{\sigma_{1,LD}}(\{C_{2z}|\mathbf{0}\}) = -i,\ \ \chi_{\sigma_{2,LD}}(\{C_{2z}|\mathbf{0}\}) = i. \tag{129}$$

Hence, through subduction [SEq. (115)], we obtain:

$$\tilde{\sigma}_{1,LD} = \sigma_{1,LD} \oplus \sigma_{2,LD}, \tag{130}$$

which may be summarized through the multiplicity matrix [SEq. (116)]:

$$c^{LD} = \begin{pmatrix} 1 \\ 1 \end{pmatrix}. \tag{131}$$

Next, we obtain a left inverse for c^{LD} by establishing that $[G_{LD} : H_{LD}] = 2$ [see SEq. (15) and the surrounding text], and that:

$$\sigma_{1,LD} \uparrow G_{LD} = \sigma_{2,LD} \uparrow G_{LD} = \tilde{\sigma}_{1,LD}. \tag{132}$$

Through SEq. (120), this implies that:

$$\left(c^{LD}\right)^{-1} = \frac{1}{2}\begin{pmatrix} 1 & 1 \end{pmatrix}. \tag{133}$$

As a final step towards computing the corep compatibility relations $m^{\Gamma,LD}$, we use subduction to obtain the unitary subgroup compatibility relations [SEq. (113)]:

$$\begin{aligned} \sigma_{1,\Gamma} \downarrow H_{LD} &= \sigma_{3,\Gamma} \downarrow H_{LD} = \sigma_{1,LD}, \\ \sigma_{2,\Gamma} \downarrow H_{LD} &= \sigma_{4,\Gamma} \downarrow H_{LD} = \sigma_{2,LD}. \end{aligned} \tag{134}$$

consistent with the output of the earlier DCOMPREL tool. SEq. (134) may be summarized by the multiplicity matrix [SEq. (114)]:

$$n^{\Gamma,LD} = \begin{pmatrix} 1 & 0 & 1 & 0 \\ 0 & 1 & 0 & 1 \end{pmatrix}.$$

Lastly, we compute the small corep compatibility relations $m^{\Gamma,LD}$ using SEq. (121):

$$m^{\Gamma,LD} = \left(c^{LD}\right)^{-1} n^{\Gamma,LD} b^\Gamma = \frac{1}{2}\begin{pmatrix} 1 & 1 \end{pmatrix}\begin{pmatrix} 1 & 0 & 1 & 0 \\ 0 & 1 & 0 & 1 \end{pmatrix}\begin{pmatrix} 1 & 0 \\ 1 & 0 \\ 0 & 1 \\ 0 & 1 \end{pmatrix} = \begin{pmatrix} 1 & 1 \end{pmatrix},$$

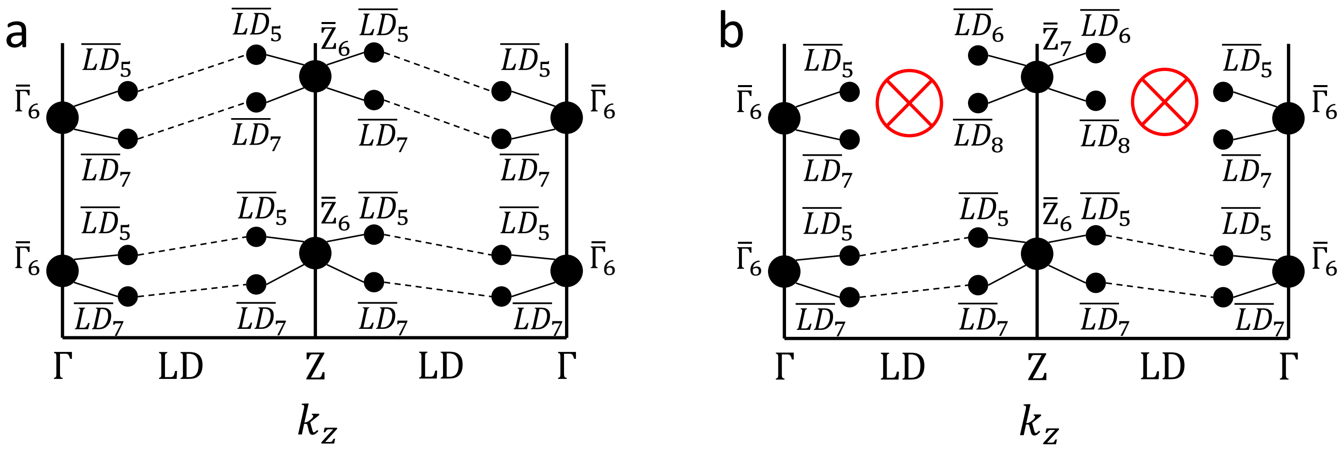

Supplementary Figure 12: Compatibility relations and graphs for magnetic rod group (MRG) $(p422)_{RG}$, which is generated by $\{E|1\}$, $\{C_{4z}|0\}$, and $\{C_{2x}|0\}$, and is isomorphic after the addition of perpendicular lattice translations to Type-I MSG 89.87 $P422$ [see SRefs. 10,33,69–71 and the text following SEq. (2)]. Using the MKVEC tool on the BCS for the $k_x = k_y = 0$ line in MSG 89.87 $P422$, we deduce that there are only three momentum stars (SN 12) in MRG $(p422)_{RG}$: Γ $(k_z = 0)$, Z $(k_z = \pi)$, and LD $(k_z = \pm w)$. Next, using MCOMPREL, we obtain the compatibility relations for MRG $(p422)_{RG}$ [*i.e.*, the values of $m_{i,j}^{\mathbf{k},\mathbf{k}'}$ in SEq. (109)], which, restricting to double-valued (spinful) coreps, are given by $\bar{\Gamma}_6 \downarrow G_{\text{LD}} = \bar{Z}_6 \downarrow G_{\text{LD}} = \overline{\text{LD}}_5 \oplus \overline{\text{LD}}_7$ and $\bar{\Gamma}_7 \downarrow G_{\text{LD}} = \bar{Z}_7 \downarrow G_{\text{LD}} = \overline{\text{LD}}_6 \oplus \overline{\text{LD}}_8$. (a) For a set of four spinful Bloch eigenstates at each k_z point with a symmetry data vector [see SRefs. 109,110 and the text following SEq. (108)] given by $\tilde{\varsigma}_\Gamma = \bar{\Gamma}_6 \oplus \bar{\Gamma}_6$ and $\tilde{\varsigma}_Z = \bar{Z}_6 \oplus \bar{Z}_6$, a separated pair of connected graphs can be formed from the coreps at Γ and Z using the TQC graph-theory methodology detailed in SRefs. 3,4. The symmetry data in (a) is therefore compatible with an insulating (band) gap at a filling $\nu = 4$. (b) Conversely, for a set of four spinful Bloch eigenstates at each k_z point with a symmetry data vector given by $\tilde{\varsigma}_\Gamma = \bar{\Gamma}_6 \oplus \bar{\Gamma}_6$ and $\tilde{\varsigma}_Z = \bar{Z}_6 \oplus \bar{Z}_7$, there does not exist a graph for the coreps at Γ and Z that satisfies the compatibility relations. The symmetry data in (b) is therefore *incompatible* with a band gap at a filling $\nu = 4$, implying that the Bloch eigenstates at Γ and Z are connected to other, unoccupied states (bands) not described by the symmetry data. In the nomenclature of SRefs. 109,110, the symmetry data in (b) consequently corresponds to an "enforced semimetal" (ES).

in agreement with the subduction relations:

$$\tilde{\sigma}_{1,\Gamma} \downarrow G_{LD} = \tilde{\sigma}_{2,\Gamma} \downarrow G_{LD} = \tilde{\sigma}_{1,LD}, \tag{135}$$

as well as the output of the MCOMPREL tool introduced in this work.

One of the key advances of TQC[1–6] and related works[23,24] was to recognize that, for each Type-I MSG and Type-II SSG, there existed a small number of maximal $\mathbf{k}$ vectors [SEq. (59)] from which the connectivity of Bloch eigenstates (*i.e.* energy bands) throughout the entire BZ could be inferred from the symmetry data [$\tilde{\varsigma}_{\mathbf{k}}$ in SEq. (108)]. Specifically, given a symmetry data vector $\{\tilde{\varsigma}_{\mathbf{k}}\}$, the set of small coreps at each $\mathbf{k}$ point can be re-expressed as the nodes of a weighted graph whose edges are required to be consistent with the compatibility relations [*i.e.*, the values of $m_{i,j}^{\mathbf{k},\mathbf{k}'}$ in SEq. (109)]. If such a graph cannot be constructed without violating the compatibility relations, then the bands characterized by the symmetry data vector $\{\tilde{\varsigma}_{\mathbf{k}}\}$ are necessarily connected to other bands, implying that the bulk is a form of topological semimetal [an "enforced semimetal" (ES) in the nomenclature of SRef. 109 (see SFig. 12)]. However, if a graph can be constructed, then it may further be separated into disconnected subgraphs. As we will discuss in SN 17, by collecting the symmetry data induced from (magnetic) atomic orbitals located at maximal Wyckoff positions (SN 9) and using the compatibility relations in MCOMPREL to construct graphs (which may be additionally separable into disconnected subgraphs), we obtain the EBRs of all 1,651 SSGs (specifically the PEBRs of the Type-II SSGs, and the MEBRs of the Type-I, III, and IV MSGs)[1–6,11,12,20–22]. In this work, we will not provide further specific details of the TQC graph theory implementation[3,4]; we will instead simply note that, for a given SSG, once the BCS tools introduced in this work have been used to obtain the momentum stars [MKVEC, see SN 12], small coreps [Corepresentations, see SN 13], and compatibility relations [MCOMPREL, see the text following SEq. (109) in this section] then the previous graph theory construction from TQC can be used without further modification. Concurrently with the preparation of this work, the MSG compatibility relations in MCOMPREL were employed to perform a high-throughput analysis[110] of band connectivity and topology in the $\sim$ 500 magnetic materials on the BCS with well-characterized MSGs[15–18].

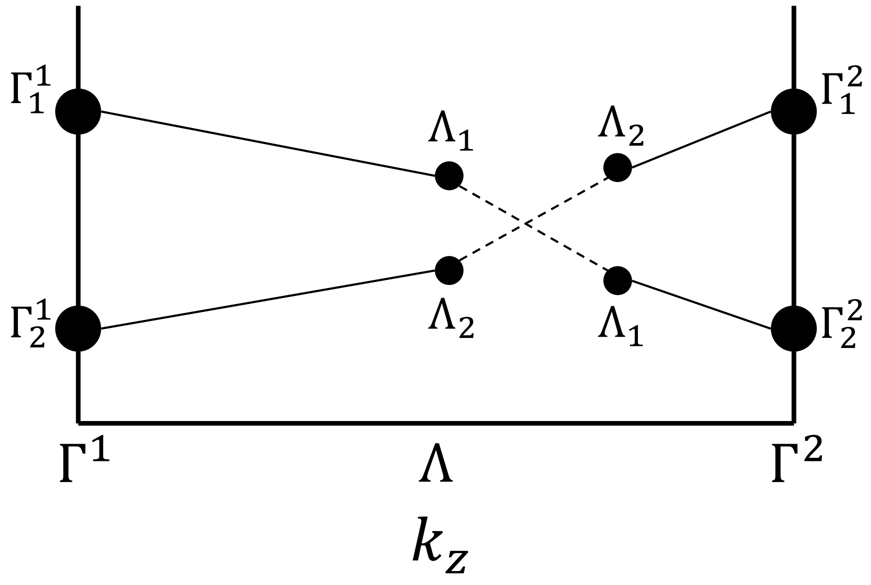

Supplementary Figure 13: Compatibility relations and graphs for MRG $(p2_1)_{RG}$, which is generated by a twofold screw operation ($s_{2_1} = \{C_{2z}|1/2\}$), and is isomorphic after the addition of perpendicular lattice translations to Type-I MSG 4.7 $P2_1$ [see SRefs. 10,33,69–71 and the text following SEq. (2)]. Using the MKVEC tool on the BCS for the $k_x = k_z = 0$ line in MSG 4.7 $P2_1$, we deduce that there is only one, multiplicity-1 momentum star (SN 12) in MRG $(p2_1)_{RG}$, which is labeled LD and lies at $\mathbf{k}_{\text{LD}} = v\hat{\mathbf{z}}$. In an example of the representation monodromy discussed in this section, the matrix representatives of the small irreps of G_{LD} are 4π-periodic in v [SEq. (139)]. Specializing to three values of v respectively given by $v = 0$ (Γ^1), $0 < v < 2\pi$ (Λ), and $v = 2\pi$ (Γ^2), we observe that the compatibility relations [SEq. (140)] imply that the small irreps $\Lambda_{1,2}$ connect to different irreps at the Γ point, depending on whether Γ lies in an odd-numbered BZ (*e.g.* Γ^1 in the first BZ) or in an even-numbered BZ (*e.g.* Γ^2 in the second BZ). Crucially, the irrep labels ($\Gamma_{1,2}^i$) are the same at the Γ point in each BZ, consistent with the restriction that physical observables in pristine crystals are 2π-periodic (*i.e.*, any physical observable in an infinite, periodic system must be the same at any two points $\mathbf{k}$ and $\mathbf{k}'$ that differ by a linear combination of reciprocal lattice vectors $\mathbf{K}_\nu$)[33]. Specifically restricting to spinless Bloch eigenstates, this implies that a pair of states (bands) with the symmetry data vector $\tilde{\varsigma}_{\Gamma^1} = \Gamma_1^1 \oplus \Gamma_2^1$ [see SRefs. 109,110 and the text following SEq. (108)], will be connected at an odd number of $\mathbf{k}$ points in each BZ, where one of the crossing points in each BZ (*i.e.* the intersection of the dashed lines in this figure) is movable, but unremovable[3,4,39,73,74,83,86,101,111–113].

As a final note, there are additional subtleties that come into play in determining the compatibility relations [SEq. (109)] and constructing connectivity graphs (SRefs. 3,4 and SFig. 12) in non-primitive SSGs [defined as SSGs whose gray Bravais lattices are not primitive[33]], SSGs without orthogonal lattice vectors [*e.g.* hexagonal SSGs], and nonsymmorphic SSGs [defined in the text following SEq. (65)]. First, in non-primitive SSGs, and in SSGs whose generating translations [SEq. (1)] are not orthogonal, the construction of a graph (or failure to construct a graph) may depend on the compatibility relations along two distinct paths between the same maximal $\mathbf{k}$ points. For example, in Type-I MSG 209.48 $F432$, given symmetry data at the maximal $\mathbf{k}$ points Γ [$\mathbf{k}_\Gamma = (0,0,0)$, G_Γ is isomorphic to Type-I MSG 209.48 $F432$] and X [$\mathbf{k}_X = (\pi,\pi,0)$, G_X is isomorphic to Type-I MSG 97.151 $I422$], the possibility of constructing a graph depends on the compatibility relations along *both* of the lines DT [$k_{DT} = (0,v,0)$, G_{DT} is isomorphic to Type-I MSG 79.25 $I4$] and SM [$\mathbf{k}_{SM} = (u,u,0)$, G_{SM} is isomorphic to Type-I MSG 5.13 $C2$]. This occurs because, for generic values of v and u, $\mathbf{k}_{DT}$ and $\mathbf{k}_{SM}$ are not related by any of the symmetries $g \in F432$ – if $\mathbf{k}_{DT}$ and $\mathbf{k}_{SM}$ were instead related by symmetries, then $\mathbf{k}_{DT}$ and $\mathbf{k}_{SM}$ would be arms of the same momentum star, and the compatibility relations across the BZ would only depend on the compatibility relations along either DT or SM. We note that, throughout the BCS, $\mathbf{k}$ points are labeled in some applications with Greek letters (*e.g.* Γ), whereas in other applications, the same $\mathbf{k}$ point is labeled with an English abbreviation (*e.g.* GM). Hence, in this work, we will in general employ a mixed notation in which Greek letters and English abbreviations are consistently used throughout each example, where specific labels are chosen to maximize consistency with previous works and with the output of the BCS tools introduced in this work.

As mentioned above, an additional subtlety occurs in nonsymmorphic SSGs. Specifically, as discussed in SRefs. 39, 73,83,86,111–113, because of the *monodromy* of representations throughout the BZ, the compatibility relations in a nonsymmorphic SSG can even differ at two $\mathbf{k}$ points that are related by a reciprocal lattice vector [$\mathbf{K}_\nu$ in SEq. (46)].

For example, consider Type-I magnetic rod group (MRG) $(p2_1)_{RG}$ [SFig. 13], which is generated by the twofold screw symmetry:

$$s_{2_1} = \{C_{2z}|1/2\}, \tag{136}$$

and is isomorphic after the addition of perpendicular lattice translations to Type-I MSG 4.7 $P2_1$ [see SRefs. 10,33,69–71 and the text following SEq. (2)]. Using the MKVEC tool on the BCS for the $k_x = k_z = 0$ line in MSG 4.7 $P2_1$, we deduce that there is only one, multiplicity-1 momentum star (SN 12) in MRG $(p2_1)_{RG}$, which is labeled LD and lies at $\mathbf{k}_{\text{LD}} = v\hat{\mathbf{z}}$. To see the effect of the representation monodromy on the compatibility relations, we will calculate the values of $m_{i,j}^{\mathbf{k},\mathbf{k}'}$ in SEq. (109) at three specific $\mathbf{k}$ points along the rod axis corresponding to different values of v in the same star (LD):

$$\mathbf{k}_{\Gamma^1} = \mathbf{0},\ \mathbf{k}_{\Lambda} = v\hat{\mathbf{z}},\ \mathbf{k}_{\Gamma^2} = 2\pi\hat{\mathbf{z}}, \tag{137}$$

where $\Gamma^{1,2}$ are related by a reciprocal lattice vector:

$$\mathbf{k}_{\Gamma^2} - \mathbf{k}_{\Gamma^1} = 2\pi\hat{\mathbf{z}}. \tag{138}$$

For simplicity, in the current demonstration of the role of representation monodromy in the compatibility relations of MRG $(p2_1)_{RG}$, we will restrict to the case of spinless Bloch eigenstates, which transform in single-valued small coreps. Using the Corepresentations tool (SN 13), we determine that, at generic points in the LD star ($\mathbf{k} = v\hat{\mathbf{z}}$), there are two, one-dimensional small coreps $\text{LD}_{1,2}$, for which the matrix representatives [and characters, see the text surrounding SEq. (71) for more information] of the twofold screw symmetry s_{2_1} [SEq. (136)] are given by:

$$\Delta_{\text{LD}_1}(s_{2_1}) = \chi_{\text{LD}_1}(s_{2_1}) = e^{iv/2},\ \Delta_{\text{LD}_2}(s_{2_1}) = \chi_{\text{LD}_2}(s_{2_1}) = -e^{iv/2}. \tag{139}$$

Evaluated at the $\mathbf{k}$ points in SEq. (137), the matrix representatives of twofold screw in SEq. (139) become:

$$\Delta_{\Gamma_1^1}(s_{2_1}) = 1,\ \Delta_{\Gamma_2^1}(s_{2_1}) = -1,\ \Delta_{\Lambda_1}(s_{2_1}) = e^{iv/2},\ \Delta_{\Lambda_2}(s_{2_1}) = -e^{-iv/2},\ \Delta_{\Gamma_1^2}(s_{2_1}) = -1,\ \Delta_{\Gamma_2^2}(s_{2_1}) = 1, \tag{140}$$

where we have employed a notation for the small irreps at the $\Gamma^{1,2}$ points in which Γ_j^i denotes the jth small irrep of the little group G_{Γ^i} at the $\mathbf{k}$ point Γ^i (*i.e.*, at the Γ point in the ith BZ). Though $G_{\Gamma^1} = G_{\Lambda} = G_{\Gamma^2}$, we can still calculate compatibility relations of the form of SEq. (109):

$$\Gamma_1^1 \downarrow G_{\Lambda} = \Lambda_1,\ \Gamma_2^1 \downarrow G_{\Lambda} = \Lambda_2,\ \Gamma_1^2 \downarrow G_{\Lambda} = \Lambda_2,\ \Gamma_2^2 \downarrow G_{\Lambda} = \Lambda_1. \tag{141}$$

In SEq. (141), we find that, because of the 4π-periodicity of the matrix representatives in SEq. (139), the compatibility relations at $\mathbf{k}_{\Gamma^{1,2}}$ are *different*, despite $\mathbf{k}_{\Gamma^{1,2}}$ differing by a reciprocal lattice vector [SEq. (138)]. This implies that, as shown in SFig. 13, a pair of spinless Bloch states at Γ^1 with the symmetry data $\varsigma_{\Gamma^1} = \Gamma_1^1 \oplus \Gamma_2^1$ [see SRefs. 109, 110 and the text following SEq. (108)] will connect with each other, specifically forming a pair of spinless bands that cross at an odd number of $\mathbf{k}$ points in each BZ, where one of the crossing points in each BZ is movable, but unremovable[3,4,39,73,74,83,86,101,111–113].

If additional symmetries are present in an SSG, such as $\{\mathcal{T}|000\}$ in Type-II SSGs (SN 4), then the effects of representation monodromy on the compatibility relations may be redundant with the constraints imposed by the additional symmetries. Specifically, in Type-II SSGs, $\mathcal{T}$ symmetry relates half of a high-symmetry line to its time-reversal partner, providing further restrictions on corep connectivity that can be used in lieu of comparing the compatibility relations at $\mathbf{k}$ points that differ by a reciprocal lattice vector (*e.g.* $\Gamma^{1,2}$ in SFig. 13)[4]. For example, adding $\mathcal{T}$ symmetry to an s_{2_1}-symmetric rod [see the text surrounding SEq. (136)] both doubles the band connectivity and introduces pinned degeneracies at the high-symmetry points $\mathbf{k}_{\Gamma^1} = \mathbf{0}$ and $\mathbf{k}_{X^1} = \pi\hat{\mathbf{z}}$, obviating the need to consider the compatibility relations at $\mathbf{k}_{\Gamma^2}$. In the case of a rod with $\mathcal{T}$ and s_{2_1} screw symmetry, the pinned degeneracies at high-symmetry points specifically occur at odd electronic fillings [*e.g.* $\nu = 1,3$], and groups of bands connect in "hourglass"-like patterns[39,73,111–113] with odd numbers of moveable-but-unremovable twofold degeneracies in each half of the BZ at fillings $\nu = 2+4n$, $n \in \{\mathbb{Z}^+, 0\}$ [*e.g.* $\nu = 2$]. Consequently, there are only 4 Type-II single and double SSGs in which monodromy constraints must be considered in addition to those imposed by the symmetries of the SSG. In Supplementary Table 3, we list the single and double SSGs in which the monodromy of representations provides necessary constraints on small corep (band) connectivity. The Type-I and Type-II SSGs listed in Supplementary Table 3 were previously calculated for TQC[1–6], whereas the Type-III and Type-IV MSGs listed in Supplementary Table 3 are a new result that we have calculated for the present work. Surprisingly, in Supplementary Table 3, we find that there are only 4 Type-IV single and double MSGs in which representation monodromy must be taken into

account to determine corep connectivity, despite the fact that *all* Type-IV MSGs are nonsymmorphic [see the text following SEq. (65)]. This occurs because each Type-IV MSG [SEq. (19)] necessarily contains a symmetry of the form:

$$\theta = \{\mathcal{T}|\mathbf{t}_0\}, \tag{142}$$

SSGs in Which the Monodromy of Representations Provides Necessary Constraints on Band Connectivity								
Type	Symbol	Number	Symbol	Number	Symbol	Number	Symbol	Number
Type-I	$P2_1$	4.7	Pc	7.24	Cc	9.37	$C222_1$	20.31
	$Pmc2_1$ (S)	26.66	$Pcc2$ (S)	27.78	$Pca2_1$	29.99	$Pnc2$	30.111
	$Pmn2_1$	31.123	$Pna2_1$	33.144	$Cmc2_1$	36.172	$Ccc2$ (S)	37.180
	$P4_1$	76.7	$P4_2$	77.13	$P4_3$	78.19	$I4_1$	80.29
	$P4_2cm$	101.179	$P4_2nm$	102.187	$P4cc$ (S)	103.195	$P4nc$ (S)	104.203
	$P4_2mc$	105.211	$P4_2bc$	106.219	$I4_1md$	109.239	$I4_1cd$	110.245
	$P3_1$	144.4	$P3_2$	145.7	$P3_112$	151.29	$P3_212$	153.37
	$P3c1$	158.57	$R3c$	161.69	$P6_1$	169.113	$P6_5$	170.117
	$P6_2$	171.121	$P6_4$	172.125	$P6_3$	173.129	$P6cc$ (S)	184.191
	$P6_3cm$	185.197	$P6_3mc$	186.203				
Type-II	$P3_11'$	144.5	$P3_21'$	145.8	$P3_1121'$	151.30	$P3_2121'$	153.38
Type-III	$P2_1/m'$ (S)	11.53	$P2'/c$ (S)	13.67	$P2_1'/c$	14.77	$P2_1/c'$	14.78
	$C2'/c$ (S)	15.87	$P2'2'2_1$	17.9	$P2_12_1'2'$	18.19	$P2_1'2_1'2_1$	19.27
	$C2'2'2_1$	20.33	$Pm'a2'$	28.89	$Pc'a2_1'$	29.101	$Pb'a2'$	32.137
	$Pn'a2_1'$	33.146	$Cm'c'2_1$	36.176	$Am'a2'$	40.205	$Ab'a2'$	41.213
	$Pccm'$ (S)	49.268	$Pb'an$ (S)	50.279	$Pm'ma$ (S)	51.291	$Pn'na$ (S)	52.307
	$Pnn'a$	52.308	$Pm'na$	53.323	$Pmna'$	53.325	$Pc'ca$	54.339
	$Pcca'$ (S)	54.341	$Pb'am$ (S)	55.355	$Pc'cn$	56.367	$Pccn'$ (S)	56.368
	$Pbc'm$ (S)	57.380	$Pbcm'$	57.381	$Pn'nm$	58.395	$Pm'mn$ (S)	59.407
	$Pb'cn$	60.419	$Pbc'n$	60.420	$Pbcn'$	60.421	$Pb'ca$	61.435
	$Pn'ma$ (S)	62.443	$Pnm'a$ (S)	62.444	$Pnma'$	62.445	$Cmcm'$ (S)	63.461
	$Cm'c'm'$ (S)	63.465	$Cmca'$	64.473	$Cm'c'a'$ (D)	64.477	$Cccm'$ (S)	66.494
	$Ccca'$ (S)	68.514	$P4_1'$	76.9	$P4_3'$	78.21	$P4_2/m'$	84.54
	$P4_2/n'$	86.70	$I4_1/a'$	88.84	$P4_12'2'$	91.106	$P4_1'2'2$	91.107
	$P4_12_1'2'$	92.114	$P4_22'2'$	93.122	$P4_22_1'2'$	94.130	$P4_32'2'$	95.138
	$P4_3'2'2$	95.139	$P4_32_1'2'$	96.146	$I4_12'2'$	98.160	$P\bar{4}'2'c$ (S)	112.261
	$P\bar{4}'2_1'c$ (S)	114.277	$P\bar{4}'c2'$ (S)	116.294	$P4/m'cc$ (S)	124.353	$P4/n'nc$ (S)	126.377
	$P4/m'nc$ (S)	128.401	$P4/n'cc$ (S)	130.425	$P4_2/m'mc$	131.437	$P4_2/m'cm$	132.449
	$P4_2/n'bc$	133.461	$P4_2/n'nm$	134.473	$P4_2/m'bc$	135.485	$P4_2/m'nm$	136.497
	$P4_2/n'mc$	137.509	$P4_2/n'cm$	138.521	$I4_1/a'md$	141.553	$I4_1/a'cd$	142.563
	$P3_112'$	151.31	$P3_12'1$	152.35	$P3_212'$	153.39	$P3_22'1$	154.43
	$P\bar{3}'c1$ (S)	165.93	$R\bar{3}'c$ (S)	167.105	$P6_3/m'$	176.146	$P6_12'2'$	178.159
	$P6_52'2'$	179.165	$P6_22'2'$	180.171	$P6_42'2'$	181.177	$P6_32'2'$	182.183
	$P\bar{6}'c2'$	188.218	$P\bar{6}'2'c$	190.229	$P6/m'cc$ (S)	192.245	$P6_3/m'cm$	193.255
	$P6_3/m'mc$	194.265						
Type-IV	P_c3_1	144.6	P_c3_2	145.9	P_c3_112	151.32	P_c3_212	153.40

Supplementary Table 3: List of SSGs for which the monodromy of representations imposes additional restrictions on small corep (band) connectivity beyond the constraints imposed by the symmetries of the SSG. The letters (S) and (D) after the symbol of an SSG respectively indicate that the representation monodromy only provides necessary constraints on the connectivity of single- and double-valued coreps of that SSG. In all of the other SSGs listed in this table, the representation monodromy provides necessary constraints on the connectivity of both single- and double-valued small coreps.

which acts the same as $\mathcal{T}$ symmetry ($\{\mathcal{T}|000\}$) on points in $\mathbf{k}$ space [SEq. (51)]:

$$\theta\mathbf{k} = \mathcal{T}\mathbf{k} = -\mathbf{k}. \tag{143}$$

Conversely, in Type-I MSGs, which only contain unitary symmetries (SN 3), and in Type-III MSGs, which only contain unitary symmetries and antiunitary symmetries of the form $\{h\times\mathcal{T}|\mathbf{v}\}$ in which h is a unitary symmetry $h \neq E$ (SN 5), we find that representation monodromy frequently provides necessary constraints on corep connectivity. As shown in Supplementary Table 3, we specifically find that there are 38 Type-I single MSGs, 32 Type-I double MSGs, 92 Type-III single MSGs, and 65 Type-III double MSGs in which the connectivity of small coreps can only be fully determined by considering the effects of representation monodromy on the compatibility relations.

17. Introduction to the Elementary Band Corepresentations of the MSGs (MEBRs)

In the sections below, we will adapt the procedure previously employed in SRefs. 1,4 to obtain the magnetic elementary band corepresentations (MEBRs) of the Type-III and Type-IV single and double MSGs. Along with the Type-I MEBRs of the Type-I MSGs and the physical EBRs (PEBRs) of the Type-II SSGs previously tabulated in SRefs. 1,4, the MEBRs of the Type-III and IV MSGs form the foundation of MTQC. More generally, in this work, we will consider PEBRs and Type-III and Type-IV MEBRs to both be *elementary band corepresentations* (EBRs), because they derive from Type-I MEBRs of Type-I (unitary) MSGs that are related by the action of antiunitary symmetries (SN 13). We note that, previously in TQC[1–6], the Type-I MEBRs of the Type-I MSGs were termed EBRs, to draw contrast with the PEBRs of the Type-II SSGs. However, in this work, we will revise the previous terminology to accomodate the elementary band corepresentations of the Type-III and IV MSGs – in this work, all elementary band (co)representations are in general termed *EBRs*, the elementary band corepresentations of Type-II SSGs remain termed *PEBRs*, and the elementary band (co)representations of Type-I, III, and IV MSGs are respectively termed *Type-I, III, and IV MEBRs*.

Below, we will show that the EBRs provide a basis for all Wannierizable[1,6,114,115], mean-field crystalline insulators, with or without magnetism. First in SN 18, we will introduce the concept of (magnetic) atomic orbitals, which we will then relate to maximally (exponentially) localized, symmetric Wannier functions[114,115]. Importantly, in SN 18, we will establish a rigorous correspondence between (magnetic) atomic orbitals and the (co)reps of Shubnikov point groups (SPGs)[7–18] (as well as site-symmetry groups, see SN 8). Next, in SN 22, we will adapt the central machinery of band induction and small corep subduction from TQC to MTQC. Specifically, in SN 22, we will use the magnetic atomic orbitals introduced in SN 18 to induce band corepresentations, which we will then Fourier transform and subduce onto little groups to obtain dependencies between small coreps in momentum space (SN 13) and site-symmetry group coreps in position space (SN 18). In SN 23, we will then enumerate the MEBRs by inducing band coreps from maximal Wyckoff positions and then excluding the *exceptional* cases (SRefs. 3,4,83–86 and SN 24) of band coreps induced from maximal Wyckoff positions that are non-elementary (*i.e.* composite). Finally, in SN 25, we will provide detailed statistics for the EBRs of all 1,651 SSGs, as well as introduce and detail the MBANDREP tool on the BCS, which we have implemented for this work to access both the EBRs and the composite band coreps induced from each Wyckoff position in each SSG. We note that prior to this work, Evarestov Smirnov, and Egorov in SRef. 12 introduced a method for obtaining the MEBRs of the MSGs and computed representative examples, but did not perform a large-scale tabulation of MEBRs or establish a connection to magnetic band topology. As will be detailed in this section, in this work, we have employed a method equivalent to the procedure in SRef. 12 to perform the first *complete* tabulation of the single- and double-valued MEBRs of the 1,421 MSGs. Furthermore, as detailed in the main text, in this work, we have used the MEBRs to construct the first complete position-space theory of mean-field magnetic band topology – MTQC.

18. Magnetic Atomic Orbitals and the CorepresentationsPG Tool

One of the fundamental advances of TQC was to introduce a *predictive* theory of bulk topology that derived from the *position-space* chemistry of a material or model[1], instead of momentum-space quantities such as (nested) Wilson loops and Berry phases[6,28,38,39,71,112,113,116–126]. Specifically, in TQC, trivial bands in momentum-space are induced from the position-space (co)reps of the site-symmetry groups of the Wyckoff positions in a pristine crystal that is invariant under a particular SSG. As previously discussed in SN 8, site-symmetry groups in SSGs, magnetic or otherwise, are necessarily isomorphic to Shubnikov point groups (SPGs).

In the Type-II (nonmagnetic) SGs first analyzed with TQC, the authors of SRef. 1 exploited a correspondence between the coreps of the site-symmetry groups in solid-state materials and the eigenstates of the Schrödinger Hamil-

tonian for a hydrogen atom (hydrogenic ion). Specifically, because the Schrödinger Hamiltonian for an ion with a single electron is spherically symmetric (isotropic) and nonmagnetic, then the Hamiltonian is invariant under the action of *any* point group, crystallographic or otherwise[9]. In the language of group theory, the Schrödinger Hamiltonian for a hydrogenic ion is invariant under the action of the symmetries of the nonmagnetic (Type-II) group $\text{Pin}^-(3) \cup \mathcal{T} \times \text{Pin}^-(3)$ [see SRefs. 101,127 for a detailed discussion of the relationship between $\text{Pin}^-(3)$, SO(3), and SU(2) in condensed matter physics]. For the purposes of this work, it is sufficient to note that $\text{Pin}^-(3) \cup \mathcal{T} \times \text{Pin}^-(3)$ is composed of spinful rotations [*e.g.* C_{2z}, for which $(C_{2z})^2 = -1$], rotoinversions of the form of the product of spinful rotations and spinless inversion $\mathcal{I}$ [*e.g.* $m_z = C_{2z} \times \mathcal{I}$, for which $(m_z)^2 = -1$, $(\mathcal{I})^2 = +1$], and antiunitary elements of the form of $\mathcal{T}$ multiplied by rotation or rotoinversion [*e.g.* $C_{2z} \times \mathcal{T}$, for which $(\mathcal{T})^2 = -1$, such that $(C_{2z} \times \mathcal{T})^2 = +1$]. Consequently, the infinite group $\text{Pin}^-(3) \cup \mathcal{T} \times \text{Pin}^-(3)$ is a supergroup of *any* finite single or double 3D point group[9,128] [see the text following SEq. (27)]. Returning to the hydrogenic ion, the eigenstates of the Schrödinger Hamiltonian are given by $\boldsymbol{\psi}^\sigma(r,\theta,\phi) = R(r)Y(\theta,\phi)\mathbf{S}^\sigma_{1/2}$, where $\mathbf{S}^\sigma_{1/2}$ is a two-level, fermionic spinor for which $\sigma = \uparrow, \downarrow$. In $\boldsymbol{\psi}^\sigma$, the angular part $Y(\theta,\phi)$ can be expressed in either the basis of spherical or cubic harmonics[128–130]; therefore, in this section, we will denote $Y(\theta,\phi)$ with suppressed angular (l, m_l) or orbital (*e.g.* s, d_{xy}) indices whenever $Y(\theta,\phi)$ appears in a basis-independent expression or statement. Across the set of wavefunctions $\{\boldsymbol{\psi}^\sigma(r,\theta,\phi)\}$, the infinite set of angular and spin parts $\{Y(\theta,\phi)\} \otimes \{\mathbf{S}^\sigma_{1/2}\}$ spans both the infinite set of basis functions of $\text{Pin}^-(3) \cup \mathcal{T} \times \text{Pin}^-(3)$, as well as the infinite set of basis functions of $\text{Pin}^-(3)$, the maximal unitary (Type-I) magnetic subgroup of $\text{Pin}^-(3) \cup \mathcal{T} \times \text{Pin}^-(3)$. We further note that the hydrogenic ion wavefunctions can also be expressed in a basis of coupled spinorbitals $\boldsymbol{\psi}^\sigma(r,\theta,\phi) = R(r)\mathbf{J}^\sigma(\theta,\phi)$. However, the set of all spinful basis functions (spin-orbit-coupled angular parts) $\{\mathbf{J}^\sigma(\theta,\phi)\}$ can be generated using only spinless angular parts and two-level (spin-1/2) spinors,

$$\Big\{\mathbf{J}^\sigma(\theta,\phi)\Big\} = \Big\{Y(\theta,\phi)\Big\} \otimes \Big\{\mathbf{S}^\sigma_{1/2}\Big\}, \tag{144}$$

in which appropriately chosen Clebsch-Gordan coefficients (*c.f.* the tables in SRef. 9) are required to relate $\mathbf{J}^{j,m_j}_l(\theta,\phi)$ and $Y^{m_l}_l(\theta,\phi)\mathbf{S}^\sigma_{1/2}$ for specific values of j, m_j, m_l, and σ. Therefore, for the purposes of this work, we are free to simplify notation by restricting consideration to hydrogenic ion wavefunctions of the form $\boldsymbol{\psi}^\sigma(r,\theta,\phi) = R(r)Y(\theta,\phi)\mathbf{S}^\sigma_{1/2}$. Hence, we may subduce the infinitely many irreducible coreps of $\text{Pin}^-(3) \cup \mathcal{T} \times \text{Pin}^-(3)$ onto any finite SPG $G_\mathbf{q}$ [which can either be a magnetic point group (MPG) or a nonmagnetic SPG, see the text following SEq. (27)], yielding the established result[9,128–130] that the finite set of irreducible (co)reps of $G_\mathbf{q}$ are spanned by the [infinitely overcomplete] set of irreducible coreps of $\text{Pin}^-(3) \cup \mathcal{T} \times \text{Pin}^-(3)$ subduced onto $G_\mathbf{q}$. Specifically, there always exists at least one [and in fact, are infinitely many] corep[s] of $\text{Pin}^-(3) \cup \mathcal{T} \times \text{Pin}^-(3)$ that subduce[s] to each irreducible (co)rep of $G_\mathbf{q}$. We therefore conclude that the set $\{Y(\theta,\phi)\} \otimes \{\mathbf{S}^\sigma_{1/2}\}$ necessarily spans the basis functions of the single- and double-valued (co)reps of any $G_\mathbf{q}$, because the (co)reps of a particular $G_\mathbf{q}$ are formed from the irreps of its maximal unitary subgroup $H_\mathbf{q}$, which is a subgroup of $\text{Pin}^-(3)$.

This establishes a correspondence between appropriately chosen linear combinations of the basis functions in $\{Y(\theta,\phi)\} \otimes \{\mathbf{S}^\sigma_{1/2}\}$ and the (co)reps of $G_\mathbf{q}$. For the nonmagnetic (Type-II) SPGs (site-symmetry groups) studied in TQC[1], the correspondence is intuitive. Specifically, given a Type-II SPG $G_\mathbf{q}$ and a hydrogenic ion wavefunction $\boldsymbol{\psi}^\sigma(r,\theta,\phi) = R(r)Y(\theta,\phi)\mathbf{S}^\sigma_{1/2}$ whose angular part $Y(\theta,\phi)$ is expressed in the basis of atomic orbitals in which it is real-valued (*i.e.* the basis of cubic harmonics[128–130]), one can first determine if $\boldsymbol{\psi}^\sigma(r,\theta,\phi)$ is an eigenstate of the unitary symmetries (*i.e.* proper rotations and rotoinversions) $h \in H_\mathbf{q}$, where $H_\mathbf{q}$ is the maximal unitary subgroup of $G_\mathbf{q}$, and where h includes SU(2) spin rotations if $H_\mathbf{q}$ is a single group. First, if $\boldsymbol{\psi}^\sigma(r,\theta,\phi)$ is an eigenstate of each $h \in H_\mathbf{q}$, then $\boldsymbol{\psi}^\sigma(r,\theta,\phi)$ can be classified by the phase λ_h that it acquires under the action of each $h \in H_\mathbf{q}$ [*i.e.*, by the eigenvalue λ_h of h: $h\boldsymbol{\psi}^\sigma(r,\theta,\phi) = \lambda_h\boldsymbol{\psi}^\sigma(r,\theta,\phi)$]. Conversely, if $\boldsymbol{\psi}^\sigma(r,\theta,\phi)$ is *not* an eigenstate of any of the unitary operations $h \in H_\mathbf{q}$, then one can instead form an orthonormal set of symmetrized wavefunctions $\tilde{\boldsymbol{\psi}}^\sigma(r,\theta,\phi)$ from linear combinations of the wavefunctions in the set $\{h\boldsymbol{\psi}^\sigma(r,\theta,\phi)h^{-1}\}$ taken over all $h \in H_\mathbf{q}$. Using the values of λ_h for each symmetry $h \in H_\mathbf{q}$ acting on $\boldsymbol{\psi}^\sigma(r,\theta,\phi)$ [or on the orthonormal set of symmetrized $\tilde{\boldsymbol{\psi}}^\sigma(r,\theta,\phi)$ formed from $\{h\boldsymbol{\psi}^\sigma(r,\theta,\phi)h^{-1}\}$], each atomic [ionic] orbital [or symmetric set of atomic orbitals] can then be uniquely labeled by a (co)rep of $G_\mathbf{q}$[131,132]. Specifically, for each atomic orbital or symmetric set of orbitals, there is only one (co)rep $\tilde{\rho}$ of $G_\mathbf{q}$ whose characters [see the text following SEq. (71)] satisfy $\chi_{\tilde{\rho}}(h) = \sum_i \lambda_{h,i}$ for each $h \in H_\mathbf{q}$ and wavefunction $\tilde{\boldsymbol{\psi}}^\sigma_i(r,\theta,\phi)$ in the symmetrized, orthonormal basis of $\{h\boldsymbol{\psi}^\sigma(r,\theta,\phi)h^{-1}\}$. Following the terminology employed in TQC[1], we refer to the correspondence between [a set of] atomic orbital[s] and a (co)rep $\tilde{\rho}$ by stating that the atomic orbital [or set of orbitals] "transforms in" the (co)rep $\tilde{\rho}$.

If $\mathcal{T}$ symmetry is relaxed, however, then $G_\mathbf{q}$ necessarily becomes isomorphic to a Type-I or Type-III magnetic point group [MPG, see the text following SEq. (27)]. In the case in which $G_\mathbf{q}$ is isomorphic to an MPG, the correspondence

between (co)reps and atomic orbitals is more opaque. Specifically, the basis functions of the (co)reps of the MPGs are still spanned by the set $\{Y(\theta,\phi)\}\otimes\{\mathbf{S}^{\sigma}_{1/2}\}$, which occurs because each MPG is a subgroup of a Type-II SPG [see the text text following SEq. (27)], which is itself a subgroup of $\text{Pin}^{-}(3)\cup\mathcal{T}\times\text{Pin}^{-}(3)$. However, as we will show in this section, for some MPG (co)reps, the corresponding $\boldsymbol{\psi}^{\sigma}(r,\theta,\phi)$ is only an eigenstate of the unitary symmetries h in the MPG if the angular part $Y(\theta,\phi)$ is expressed in the complex basis of spherical harmonics[128–130]. Therefore, for this work, we introduce the term *magnetic atomic orbital* to reference the basis functions that transform in the lowest-dimensional [*i.e.* in one-dimensional] MPG (co)reps[9]. As we will show in the examples below (SN 19, 20, and 21), the angular parts $Y(\theta,\phi)$ of some magnetic atomic orbitals can be expressed in the real basis of the familiar cubic harmonics (*i.e.* atomic orbitals, such as s and d_{xy}), whereas the angular parts of other magnetic orbitals necessarily take the form of $\mathcal{T}$-breaking linear combinations of cubic harmonics (*i.e.* spherical harmonics, such as $p_x \pm ip_y$ magnetic atomic orbitals).

Because the 3D magnetic atomic orbitals are relatively esoteric, especially when considering the combined effects of SOC and magnetism, then we will leave the complete tabulation of the magnetic atomic orbitals that transform in each (co)rep of each SPG for future works. However, we will still in this work detail representative examples of MPG (co)reps and their corresponding magnetic atomic orbitals. In SN 19, 20, and 21, we will respectively determine the lowest-angular-momentum, spin-degenerate pair of magnetic atomic orbitals that transforms in each single-valued (co)rep of Type-I MPG 9.1.29 4, Type-III MPG 9.3.31 $4'$, and Type-II SPG 9.2.30 $41'$ [as was previously done in SN 8, we will continue to label SPGs employing the notation of the MPOINT tool on the BCS[15–18] in which an SPG is labeled by its number, followed by its symbol].

Lastly, we note that double-valued MPG (co)reps in general correspond to less intuitive tensor products of fermionic spinors and real-space wavefunctions[9,33] [*i.e.*, linear combinations of the basis functions in $\{Y(\theta,\phi)\}\otimes\{\mathbf{S}^{\sigma}_{1/2}\}$]. For example, a $(d_{xy}+id_{x^2-y^2})\otimes\mathbf{S}^{\uparrow}_{z}$ magnetic atomic spinorbital is less familiar than a nonmagnetic spinless d_{xy} orbital. Conversely, single-valued MPG (co)reps correspond to spin-degenerate linear combinations of the basis functions in $\{Y(\theta,\phi)\}\otimes\mathbb{1}_{\sigma}$, where $\mathbb{1}_{\sigma}$ is the 2×2 identity in the space of $\mathbf{S}^{\sigma}_{1/2}$. Hence, for simplicity, in the examples in SN 19, 20, and 21, we will restrict focus to the single-valued (co)reps of single SPGs and their corresponding [spin-degenerate pairs of] magnetic atomic orbitals.

Throughout this section, we will obtain the (co)reps of SPGs through character tables reproduced from the CorepresentationsPG tool on the BCS, which we have implemented for this work. For each of the 122 crystallographic SPGs, CorepresentationsPG outputs the single- and double-valued (co)reps, character tables, and symmetry matrix representatives. CorepresentationsPG subsumes the earlier REPRESENTATIONS DPG tool (`https://www.cryst.ehu.es/cgi-bin/cryst/programs/representations_point.pl?tipogrupo=dbg`), which was implemented for TQC[1,2] to output the irreps and character tables of the 32 single and double Type-I MPGs. In SFig. 14, we show the output of CorepresentationsPG for Type-III MPG 5.3.14 $2'/m$.

Irreducible corepresentations of the Magnetic Point Group 2'/*m* (N. 5.3.14)

Table of characters of the unitary symmetry operations

(1)	(2)	(3)	C_1	C_2	C_3	C_4
GM_1	A'	GM_1	1	1	1	1
GM_2	A''	GM_2	1	-1	1	-1
GM_4GM_3	$^2\overline{E}\,^1\overline{E}$	$\overline{GM}_3\overline{GM}_4$	2	0	-2	0

Lists of unitary symmetry operations in the conjugacy classes

C_1: 1
C_2: m_{010}
C_3: $^{d}1$
C_4: $^{d}m_{010}$

Matrices of the representations of the group

The antiunitary operations are written in red color

N	Matrix presentation		Seitz symbol	GM_1	GM_2	$\overline{GM}_3\overline{GM}_4$
1	$\begin{pmatrix} 1 & 0 & 0 \\ 0 & 1 & 0 \\ 0 & 0 & 1 \end{pmatrix}$	$\begin{pmatrix} 1 & 0 \\ 0 & 1 \end{pmatrix}$	1	1	1	$\begin{pmatrix} 1 & 0 \\ 0 & 1 \end{pmatrix}$
2	$\begin{pmatrix} 1 & 0 & 0 \\ 0 & -1 & 0 \\ 0 & 0 & 1 \end{pmatrix}$	$\begin{pmatrix} 0 & -1 \\ 1 & 0 \end{pmatrix}$	m_{010}	1	-1	$\begin{pmatrix} -i & 0 \\ 0 & i \end{pmatrix}$
7	$\begin{pmatrix} -1 & 0 & 0 \\ 0 & 1 & 0 \\ 0 & 0 & -1 \end{pmatrix}^{I}$	$\begin{pmatrix} 0 & 1 \\ -1 & 0 \end{pmatrix}$	$^{d}2'_{010}$	1	-1	$\begin{pmatrix} 0 & -i \\ -i & 0 \end{pmatrix}$
8	$\begin{pmatrix} -1 & 0 & 0 \\ 0 & -1 & 0 \\ 0 & 0 & -1 \end{pmatrix}^{I}$	$\begin{pmatrix} -1 & 0 \\ 0 & -1 \end{pmatrix}$	$^{d}\bar{1}'$	1	1	$\begin{pmatrix} 0 & 1 \\ -1 & 0 \end{pmatrix}$

Supplementary Figure 14: The output of the CorepresentationsPG tool on the BCS for Type-III MPG 5.3.14 $2'/m$. For each of the 122 crystallographic SPGs (see SN 8 and SRefs. 7–18), CorepresentationsPG outputs the irreducible (co)reps of the SPG, the unitary symmetry operations in the SPG, and the matrix representatives of both the unitary and antiunitary symmetry elements in the SPG. For each antiunitary symmetry $g_{A,i}$ in the SPG, entries in the table of matrix representatives are labeled in red text, and the matrices listed for each (co)rep $\tilde{\rho}$ indicate the unitary part U of the antiunitary matrix representative $\Delta_{\tilde{\rho}}(g_{A,i}) = UK$, where K is complex conjugation. We note that the bottom table only contains a representative subset of the output of CorepresentationsPG for Type-III MPG 5.3.14 $2'/m$, in order to preserve the legibility of the text in this figure.

19. Irreps and Magnetic Atomic Orbitals in Type-I Single MPG 9.1.29 4

Character Table for Type-I Single MPG 9.1.29 4				
Irrep	E	C_{2z}	C_{4z}	C_{4z}^{-1}
A	1	1	1	1
B	1	1	-1	-1
${}^2E$	1	-1	i	$-i$
${}^1E$	1	-1	$-i$	i

Supplementary Table 4: Single-valued irreps and characters for Type-I single MPG 9.1.29 4, reproduced from CorepresentationsPG on the BCS. For each irrep ρ and unitary symmetry element h, elements in the table correspond to the character $\chi_\rho(h) = \text{Tr}[\Delta_\rho(h)]$, where $\Delta_\rho(h)$ is the matrix representative of h in the irrep ρ [see the text following SEq. (65)]. Following the nomenclature established in SN 13, we use the symbol E for the identity element. Because $\chi_\rho[(C_{2z})^2] = \chi_\rho[(C_{4z})^4] = \chi_\rho(E)$ for all of the single-valued irreps ρ of Type-I single MPG 9.1.29 4, then the irreps in this table can only correspond to 0D spinless (spin-degenerate) electronic (bosonic) states.

We begin by examining the magnetic atomic orbitals that transform in irreps of Type-I single MPG 9.1.29 4. In Supplementary Table 4, we reproduce the characters for single MPG 9.1.29 4, obtained from CorepresentationsPG on the BCS. In Supplementary Table 4, and for all of the SPGs discussed in this work, we have labeled (co)reps in the notation of SRef. 33, which is based on the notation employed by Mulliken in SRef. 133. For each irrep ρ and unitary symmetry element h in Supplementary Table 4, we list the character $\chi_\rho(h) = \text{Tr}[\Delta_\rho(h)]$, where $\Delta_\rho(h)$ is the matrix representative of h in the irrep ρ [see the text following SEq. (65)].

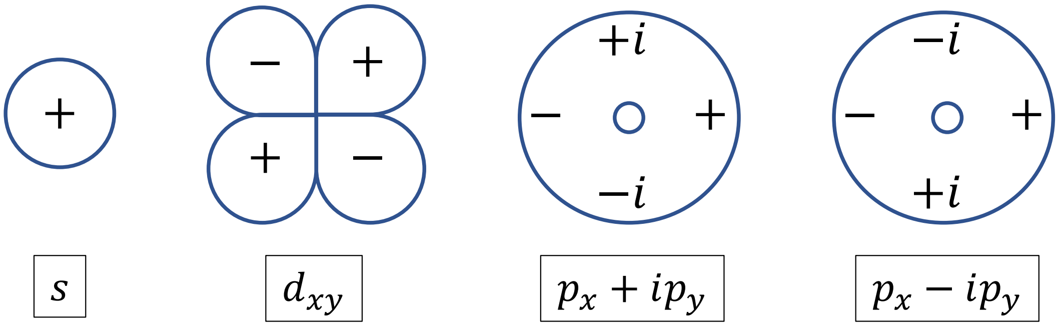

Supplementary Figure 15: The lowest-angular-momentum spinless (*i.e.* spin-degenerate pairs of) magnetic atomic orbitals that transform in[131,132] single-valued irreps of Type-I single MPG 9.1.29 4 (Supplementary Table 4). From left to right, the orbitals specifically transform in the A, B, ${}^2E$, and ${}^1E$ single-valued irreps of MPG 9.1.29 4. While the spinless s (A) and d_{xy} (B) orbitals are the same as their familiar nonmagnetic counterparts, the spinless $p_x \pm ip_y$ (${}^{2,1}E$) orbitals correspond to $\mathcal{T}$-breaking linear combinations of nonmagnetic, spinless $p_{x,y}$ orbitals. Most precisely, the angular parts of the wavefunctions of the spinless s and d_{xy} orbitals are respectively given by the s and d_{xy} cubic harmonics, whereas the angular parts of the spinless $p_x \pm ip_y$ orbitals are given by the $l = 1$, $m_l = \pm 1$ spherical harmonics[128–130].

For each irrep of single-valued Type-I MPG 9.1.29 4 in Supplementary Table 4, we obtain the corresponding lowest-angular-momentum spinless magnetic atomic orbital through the following procedure. First, because we are characterizing electronic states labeled by single-valued irreps, we restrict consideration to spin-degenerate pairs of orbitals $\{\boldsymbol{\psi}^\uparrow(r,\theta,\phi), \boldsymbol{\psi}^\downarrow(r,\theta,\phi)\}$, which we label by the spinless angular part of each orbital in the pair $Y(\theta,\phi)$. Next, we search for the circular harmonics $[Y(\theta,\phi) = Y_l^{m_l}(\theta,\phi)]$ or cubic harmonics $[Y(\theta,\phi) \propto Y_l^{m_l} \pm Y_l^{-m_l}]$[128–130] that are eigenstates of all of the unitary symmetries $h \in H_{\mathbf{q}}$ while carrying the lowest possible values of l and $|m_l|$. This procedure returns four (spin-degenerate pairs of) orbitals – one for each single-valued irrep in Supplementary Table 4 – which we depict in SFig. 15. While the (spin-degenerate pairs) of s (A) and d_{xy} (B) orbitals shown in SFig. 15 are the same as their familiar nonmagnetic counterparts, the $p_x \pm ip_y$ (${}^{2,1}E$) orbitals in SFig. 15 correspond to $\mathcal{T}$-breaking linear combinations of nonmagnetic $p_{x,y}$ orbitals. Specifically, the angular parts of the wavefunctions of the s and d_{xy} orbitals are respectively given by the s and d_{xy} cubic harmonics, whereas the angular parts of the $p_x \pm ip_y$ orbitals are given by the $l = 1$, $m_l = \pm 1$ spherical harmonics[128–130].

20. Coreps and Magnetic Atomic Orbitals in Type-III Single MPG 9.3.31 $4'$

Next, in this section, we will determine the lowest-angular-momentum magnetic atomic orbitals that transform in single-valued coreps of Type-III single MPG 9.3.31 $4'$. As discussed in the text surrounding SEq. (37), a Type-III group $G_{\mathbf{q}}$ can be re-expressed as a coset decomposition with respect to its maximal index-2 unitary subgroup $H_{\mathbf{q}}$. In the case of $G_{\mathbf{q}} = 4'$, the maximal unitary subgroup $H_{\mathbf{q}}$ is isomorphic to Type-I MPG 3.1.6 2, such that the coset decomposition is given by:

$$G_{\mathbf{q}} = 4' = 2 \cup \mathcal{T}(41' \setminus 2) = (E)2 \cup (C_{4z} \times \mathcal{T})2, \tag{145}$$

where 2 and $41'$ respectively refer to Type-I MPG 3.1.6 2 and Type-II SPG 9.2.30 $41'$. SEq. (145) implies that, unlike the previous example of Type-I MPG 3.1.6 2 in SN 19, Type-III MPG 9.3.31 $4'$ contains antiunitary symmetries, which comprise the coset $(C_{4z} \times \mathcal{T})2$.

Character Table for Type-I Single MPG 3.1.6 2		
Irrep	E	C_{2z}
A	1	1
B	1	-1

Supplementary Table 5: Single-valued irreps and characters for Type-I single MPG 3.1.6 2, reproduced from CorepresentationsPG on the BCS. For each irrep ρ and unitary symmetry in the MPG $h \in H_{\mathbf{q}}$ [SEq. (146)], the table lists the character $\chi_\rho(h) = \text{Tr}[\Delta_\rho(h)]$, where $\Delta_\rho(h)$ is the matrix representative of h in ρ [see the text following SEq. (65)]. Following the nomenclature established in SN 13, we use the symbol E for the identity element. Additionally, as previously emphasized in Supplementary Table 4, we again note that, because $\chi_\rho[(C_{2z})^2] = \chi_\rho(E)$ for all of the single-valued ρ in this table, then the irreps ρ can only correspond to spinless (spin-degenerate) electronic (bosonic) states.

To determine the single-valued coreps of Type-III single MPG 9.3.31 $4'$, we begin by examining the single-valued irreps of the maximal unitary subgroup:

$$H_{\mathbf{q}} = 2 = \left\{E,\ C_{2z}\right\}, \tag{146}$$

where 2 refers to Type-I single MPG 3.1.6 2. In Supplementary Table 5, we reproduce the characters for Type-I single MPG 3.1.6 2 from CorepresentationsPG on the BCS. To obtain the single-valued coreps of $G_{\mathbf{q}}$ (Type-III MPG 9.3.31 $4'$), we use the characters in Supplementary Table 5 to calculate the indicator J_ρ, adapted from the modified Frobenius-Schur indicator[33,97–99] J_σ for little group small coreps discussed in the text surrounding SEqs. (76) and (77):

$$J_\rho = \sum_i \chi_\rho(g_{A,i}^2), \tag{147}$$

where the sum in SEq. (147) runs over the two antiunitary elements $g_{A,i}$ in the coset $(C_{4z} \times \mathcal{T})2$ in SEq. (145). For the specific case of Type-III MPG 9.3.31 $4'$, SEqs. (145), (146), and (147) imply that:

$$\begin{aligned} J_\rho &= \chi_\rho(C_{4z}^2 \times \mathcal{T}^2) + \chi_\rho(C_{4z}^6 \times \mathcal{T}^2) \\ &= 2\chi_\rho(C_{2z}), \end{aligned} \tag{148}$$

where we have exploited that[33] $C_{4z}C_{2z} = C_{4z}^3$, and that $\mathcal{T}^2 = (C_{2z})^2 = (C_{4z})^4 = E$ for single groups. Inserting $\rho = A, B$ and the characters $\chi_{A,B}(h)$ from Supplementary Table 5 into SEq. (148), we determine that:

$$J_A = |H_{\mathbf{q}}|,\ J_B = -|H_{\mathbf{q}}|, \tag{149}$$

where $|H_{\mathbf{q}}| = 2$ is the number of elements [see the text following SEq. (8)] in Type-I single MPG 3.1.6 2 [SEq. (146)]. Following the discussion surrounding SEqs. (73), (74), and (77), SEqs. (148) and (149) imply that, in Type-III single MPG 9.3.31 $4'$, $\rho = A$ forms an undoubled, one-dimensional corep of type (a), whereas $\rho = B$ forms a doubled,

two-dimensional corep of type (b). The single-valued coreps of Type-III single MPG 9.3.31 $4'$ are therefore given by:

$$\tilde{\rho} = A,\ BB. \tag{150}$$

In Supplementary Table 6, we reproduce the characters for Type-III single MPG 9.3.31 $4'$, obtained from CorepresentationsPG on the BCS. To obtain the lowest-angular-momentum (spin-degenerate pairs of) magnetic atomic orbitals that transform in each corep in Supplementary Table 6, we follow the procedure previously described at the beginning of this section (SN 18) and in the previous section (SN 19). For the corep A in Supplementary Table 6, we find that the corresponding lowest-angular-momentum atomic orbital is a spinless (*i.e.* spin-degenerate pair of spinful) s orbital(s) (SFig. 15). Conversely, there is no individual spinless magnetic atomic orbital that transforms in the corep BB in Supplementary Table 6, because BB is two-dimensional [*i.e.*, because $\chi_{BB}(E) = 2$]. Instead, we find that the smallest set of magnetic atomic orbitals with the lowest angular momenta that transform in BB are a pair of spinless p orbitals whose lobes are oriented at $C_{4z} \times \mathcal{T}$-related angles in the xy-plane. An example of a pair of orbitals that transform in BB is one spinless p_x plus one spinless p_y orbital, which span the same two-dimensional space (four-dimensional, including spin) as one spinless $p_x + ip_y$ orbital plus one spinless $p_x - ip_y$ orbital (SFig. 15). Intuitively, this can be understood by recognizing that the lowest-angular-momentum magnetic atomic orbital that transforms in the irrep A (B) of Type-I single MPG 3.1.6 2 is a spinless s ($p_x \pm ip_y$) orbital. Under the action of $C_{4z} \times \mathcal{T}$ in SEq. (145), an s orbital is transformed to itself, whereas a $p_x \pm ip_y$ orbital is transformed into a $(p_y \mp ip_x)^* = i(p_x \mp ip_y) \propto p_x \mp ip_y$ orbital. Hence, $A \uparrow G_{\mathbf{q}} = A$ [*i.e.*, the irrep A of $H_{\mathbf{q}}$ induces a type (a) corep A in $G_{\mathbf{q}}$, see the text surrounding SEq. (73)], whereas $B \uparrow G_{\mathbf{q}} = BB$ [*i.e.*, the irrep B of $H_{\mathbf{q}}$ induces a type (b) corep BB in $G_{\mathbf{q}}$, see the text surrounding SEq. (74)].

21. Coreps and Atomic Orbitals in Type-II Single SPG 9.2.30 $41'$

As a final example, in this section, we will determine the lowest-angular-momentum, *nonmagnetic* atomic orbitals that transform in single-valued coreps of Type-II single SPG 9.2.30 $41'$, the $\mathcal{T}$-symmetric supergroup of the MPGs previously analyzed in SN 19 and 20 (Type-I MPG 9.1.29 4 and Type-III MPG 9.3.31 $4'$, respectively). Like a Type-II SSG [SEq. (3)], a Type-II MPG $G_{\mathbf{q}}$ can be re-expressed as a coset decomposition with respect to its maximal index-2 unitary subgroup $H_{\mathbf{q}}$. In the case of $G_{\mathbf{q}} = 41'$, the decomposition is:

$$41' = 4 \cup (\mathcal{T})4, \tag{151}$$

where $H_{\mathbf{q}} = 4$ refers to Type-I single MPG 9.1.29 4, which we previously analyzed in SN 19. $H_{\mathbf{q}} = 4$ contains four elements (Supplementary Table 4):

$$H_{\mathbf{q}} = \left\{E,\ C_{2z},\ C_{4z},\ C_{4z}^{-1}\right\}. \tag{152}$$

Character Table for Type-III Single MPG 9.3.31 $4'$		
Corep	E	C_{2z}
A	1	1
BB	2	-2

Supplementary Table 6: Single-valued coreps and characters for Type-III single MPG 9.3.31 $4'$, reproduced from CorepresentationsPG on the BCS. For each corep $\tilde{\rho}$ and unitary symmetry element $h \in H_{\mathbf{q}}$, where $H_{\mathbf{q}}$ is the maximal unitary subgroup of MPG 9.3.31 $4'$ [Supplementary Table 5 and SEqs. (145) and (146)], the table lists the character $\chi_{\tilde{\rho}}(h) = \mathrm{Tr}[\Delta_{\tilde{\rho}}(h)]$, where $\Delta_{\tilde{\rho}}(h)$ is the matrix representative of h in the corep $\tilde{\rho}$ [see the text following SEq. (65)]. Following the nomenclature established in SN 13, we use the symbol E for the identity element. Because the matrix representatives of the antiunitary symmetries in Type-III MPG 9.3.31 $4'$ [*i.e.*, the antiunitary elements of the coset $(C_{4z} \times \mathcal{T})2$ in SEq. (145)] are also antiunitary, then they do not have well-defined traces, and do not appear in the character table. In SEqs. (148), (149), (150), we show that Type-III single MPG 9.3.31 $4'$ has two single-valued coreps: there is one, one-dimensional, single-valued corep A, which is equivalent [defined in the text surrounding SEq. (73)] to an irrep (A) of $H_{\mathbf{q}}$ (Supplementary Table 5), and there is one, two-dimensional, single-valued corep $BB \equiv B \oplus B$, which is equivalent [defined in the text surrounding SEq. (74)] to two copies of the same irrep (B) of $H_{\mathbf{q}}$.

Using the character table for Type-I MPG 9.1.29 4 (Supplementary Table 4), we previously determined in SN 19 that the four single-valued irreps of $H_{\mathbf{q}} = 4$ given by $\rho = A,\ B,\ {}^2E,\ {}^1E$ respectively correspond to spinless (*i.e.* spin-degenerate pairs of) s, d_{xy}, $p_x + ip_y$, and $p_x - ip_y$ magnetic atomic orbitals.

Character Table for Type-II Single SPG 9.2.30 41′				
Corep	E	C_{2z}	C_{4z}	C_{4z}^{-1}
A	1	1	1	1
B	1	1	-1	-1
${}^1E\ {}^2E$	2	-2	0	0

Supplementary Table 7: Single-valued coreps and characters for Type-II single SPG 9.2.30 41′, reproduced from CorepresentationsPG on the BCS. For each corep $\tilde{\rho}$ and unitary symmetry element $h \in H_{\mathbf{q}}$, where $H_{\mathbf{q}}$ is the maximal unitary subgroup of SPG 9.2.30 (41′) [Supplementary Table 4 and SEqs. (151) and (152)], the table lists the character $\chi_{\tilde{\rho}}(h) = \text{Tr}[\Delta_{\tilde{\rho}}(h)]$, where $\Delta_{\tilde{\rho}}(h)$ is the matrix representative of h in the corep $\tilde{\rho}$ [see the text following SEq. (65)]. Following the nomenclature established in SN 13, we use the symbol E for the identity element. Because the matrix representatives of the antiunitary symmetries in Type-II SPG 9.2.30 41′ [*i.e.*, the antiunitary elements of the coset $(\mathcal{T})4$ in SEq. (151)] are also antiunitary, then they do not have well-defined traces, and do not appear in the character table. In SEqs. (154), (155), and (156), we show that Type-II single MPG 9.2.30 41′ has three single-valued coreps: there are two, one-dimensional, single-valued coreps A and B, which are equivalent [defined in the text surrounding SEq. (73)] to irreps (A and B, respectively) of $H_{\mathbf{q}}$ (Supplementary Table 4), and there is one, two-dimensional, single-valued corep ${}^1E\ {}^2E \equiv {}^1E \oplus {}^2E$, which is formed [defined in the text surrounding SEq. (75)] from pairing two different irreps (${}^1E$ and ${}^2E$) of $H_{\mathbf{q}}$. We note that, because $\chi_{\tilde{\rho}}[(C_{2z})^2] = \chi_{\tilde{\rho}}[(C_{4z})^4] = \chi_{\tilde{\rho}}(E)$ for all of the single-valued $\tilde{\rho}$ in this table, then the coreps $\tilde{\rho}$ can only correspond to 0D spinless (spin-degenerate) electronic (bosonic) states.

To determine the single-valued coreps $\tilde{\rho}$ of Type-II single SPG 9.2.30 41′, we again calculate the indicator J_ρ discussed in the text surrounding SEq. (147):

$$J_\rho = \sum_i \chi_\rho(g_{A,i}^2), \tag{153}$$

where the sum in SEq. (153) runs over the four antiunitary elements $g_{A,i}$ in the coset $(\mathcal{T})4$ in SEq. (151). In the specific case of Type-II single SPG 9.2.30 41′, SEqs. (151), (152), and (153) imply that:

$$\begin{aligned} J_\rho &= \chi_\rho(\mathcal{T}^2) + \chi_\rho(C_{2z}^2 \times \mathcal{T}^2) + \chi_\rho(C_{4z}^2 \times \mathcal{T}^2) + \chi_\rho(C_{4z}^{-2} \times \mathcal{T}^2) \\ &= 2\left[\chi_\rho(E) + \chi_\rho(C_{2z})\right], \end{aligned} \tag{154}$$

where we have exploited that[33] $C_{4z}^2 = C_{2z}$, and that $C_{4z}^{-2} = C_{2z}^{-1} = C_{2z}$ and $\mathcal{T}^2 = (C_{2z})^2 = (C_{4z})^4 = E$ for single groups. Inserting $\rho = A, B, {}^2E, {}^1E$ and the characters from Supplementary Table 4 into SEq. (154), we determine that:

$$J_A = J_B = |H_{\mathbf{q}}|,\ J_{{}^2E} = J_{{}^1E} = 0, \tag{155}$$

where $|H_{\mathbf{q}}| = 4$ is the number of elements [see the text following SEq. (8)] in Type-I single MPG 9.1.29 4 [SEq. (152)]. Following the discussion surrounding SEqs. (73), (75), and (77), SEqs. (154) and (155) imply that, in Type-II single MPG 9.2.30 41′, $\rho = A$ and $\rho = B$ each form undoubled, one-dimensional coreps of type (a), whereas $\rho = {}^2E$ and $\rho = {}^1E$ together form a paired, two-dimensional corep of type (c). The single-valued coreps of Type-II single MPG 9.2.30 41′ are therefore given by:

$$\tilde{\rho} = A,\ B,\ {}^1E\ {}^2E. \tag{156}$$

In Supplementary Table 7, we reproduce the characters for Type-II SPG 9.2.30 41′, obtained from CorepresentationsPG on the BCS. Like in $H_{\mathbf{q}} = 4$, the maximal unitary subgroup of SPG 9.2.30 41′ [see SN 19 and SEqs. (151) and (152)], the lowest-angular-momentum (spin-degenerate pairs of) atomic orbitals that transform in the single-valued coreps A and B of Type-II SPG 9.2.30 41′ are respectively spinless s and spinless d_{xy} orbitals (SFig. 15). Conversely, there is no individual spinless atomic orbital that transforms in the corep ${}^1E\ {}^2E$ in Supplementary Table 7, because ${}^1E\ {}^2E$ is two-dimensional [*i.e.*, because $\chi_{{}^1E\ {}^2E}(E) = 2$]. Instead, we find that the smallest set of atomic orbitals with the lowest angular momenta that together transform in ${}^1E\ {}^2E$ are a Kramers pair of spinless $p_x \pm ip_y$ magnetic atomic orbitals [*i.e.* one spinless $p_x + ip_y$ plus one spinless $p_x - ip_y$ orbital (SFig. 15)], which are usually

denoted more succinctly in other works[1] as "spinless p_x and p_y orbitals", because they span the same two-dimensional space (four-dimensional, including spin) as one spinless p_x orbital plus one spinless p_y orbital. Intuitively, this can be understood by recognizing that the lowest-angular-momentum magnetic atomic orbitals that transform in the irreps A, B, ${}^2E$, ${}^1E$ of Type-I MPG 9.1.29 4 are respectively spinless s, d_{xy}, $p_x + ip_y$, and $p_x - ip_y$ magnetic atomic orbitals (SFig. 15). Under the action of $\mathcal{T}$ symmetry in SEq. (151), an s or d_{xy} orbital is transformed to itself, whereas a $p_x \pm ip_y$ orbital is transformed into a $p_x \mp ip_y$ orbital. Hence, $A \uparrow G_{\mathbf{q}} = A$ and $B \uparrow G_{\mathbf{q}} = B$ [*i.e.*, the irreps A and B of $H_{\mathbf{q}}$ respectively induce the type (a) coreps A and B in $G_{\mathbf{q}}$, see the text surrounding SEq. (73)], whereas ${}^{1,2}E \uparrow G_{\mathbf{q}} = {}^1E\ {}^2E$ [*i.e.*, the irreps ${}^{1,2}E$ of $H_{\mathbf{q}}$ each induce a type (c) corep ${}^1E\ {}^2E$ in $G_{\mathbf{q}}$, see the text surrounding SEq. (75)].

22. Inducing Band Corepresentations from Magnetic Atomic Orbitals and the MSITESYM Tool

Building upon the earlier definitions of site-symmetry groups [SN 8], Wyckoff positions [SN 9], little groups [SN 12], small (co)reps of the SSGs [SN 13], and magnetic atomic orbitals that transform in (co)reps of the site-symmetry groups [SN 18], we will now in this section define the band (co)representations of the SSGs, which are induced from exponentially localized [Wannier[114,115]] orbitals in position space. We will also introduce and detail the MSITESYM tool, through which users may access the small (co)reps subduced from each band (co)representation of each SSG. This section is largely a review of previous works that discuss induced band (co)representations – most notably SRef. 4 – though throughout this section, we will employ a more general terminology than in SRef. 4 that encompasses both magnetic and nonmagnetic band (co)representations. In particular, in this section, we will introduce the term *band corepresentation* to refer to a band representation in an SSG with antiunitary symmetries [*i.e.*, a Type-II, III, or IV SSG (SN 4, 5, and 6, respectively)]. Specific examples demonstrating usage of the theoretical machinery established in this section are provided in SN 24 and 29 for cases of magnetic band (co)representations, and are provided in SRefs. 1–4 for cases of nonmagnetic band corepresentations.

To begin, consider an infinite crystal whose unit cells are furnished with initially decoupled (magnetic) atomic orbitals. The set of atomic orbitals respects the symmetries of the SSG of the crystal G, and, by definition, each orbital at $\mathbf{q}$ occupies a site in a Wyckoff position of G with a site-symmetry group $G_{\mathbf{q}} \in G$ (SN 7). As discussed in SN 9, $G_{\mathbf{q}}$ is a subgroup of G ($G_{\mathbf{q}} \subset G$) that is isomorphic to a Shubnikov point group (SPG) (SN 8) containing a set of symmetries $g \in G_{\mathbf{q}}$, $g \in G$. Generically, there also exist a set of symmetries:

$$\tilde{g} \in G \setminus G_{\mathbf{q}}, \tag{157}$$

for which:

$$\tilde{g}\mathbf{q} = \mathbf{q}', \tag{158}$$

where $\mathbf{q}'$ is a different site than $\mathbf{q}$ in the same unit cell. The set of all sites $\{\mathbf{q}_\alpha\}$ in the same unit cell as $\mathbf{q}$ (including $\mathbf{q}$ itself) form the Wyckoff orbit of $\mathbf{q}$, where the index α on $\mathbf{q}_\alpha$ runs from 1 to n, where n is the multiplicity of the Wyckoff orbit indexed by $\mathbf{q}$ (see SN 9). We emphasize that the choice of $\tilde{g}$ in SEqs. (157) and (158) is not generically unique – for example, in Type-I MSG 10.42 $P2/m$, which is generated by $\{\mathcal{I}|\mathbf{0}\}$, $\{C_{2y}|\mathbf{0}\}$, and 3D lattice translations, the sites $\mathbf{q} = (u, 0, w)$ and $\mathbf{q}' = (-u, 0, -w)$ are related by both $\tilde{g} = \{\mathcal{I}|\mathbf{0}\}$ and $\tilde{g} = \{C_{2y}|\mathbf{0}\}$. We additionally emphasize that the restriction to $\mathbf{q}'$ that lie in the same unit cell as $\mathbf{q}$ is a convention choice that was employed previously in TQC[1–6] that we will continue to employ in MTQC to obtain MEBRs consistent with the PEBRs previously calculated for TQC. More generally, a set of EBRs can be still be computed as long as each $\mathbf{q}'$ is unique and is not related to $\mathbf{q}$ or to any other $\mathbf{q}'$ by an integer-valued linear combination of primitive lattice vectors.

We will find it convenient in this section to initially restrict to the case in which the crystal is furnished by a set of (magnetic) atomic orbitals at each site of a single Wyckoff position indexed by $\mathbf{q}$ that transforms in one and only one (*i.e.* in an irreducible) (co)rep $\tilde{\rho}_{\mathbf{q}}$ of the site-symmetry group $G_{\mathbf{q}}$. Because reducible [composite] site-symmetry [band] (co)representations may be expressed as direct sums of irreducible [elementary] site-symmetry [band] (co)representations, then, at the end of this section, we will straightforwardly relax this restriction and consider the more general case in which the unit cell contains larger sets of atomic orbitals that transform in direct sums of site-symmetry (co)reps. In the language of SRefs. 114,115, each magnetic atomic orbital at $\mathbf{q}$ (including spin) corresponds to an exponentially (maximally) localized (spinful), symmetric Wannier orbital. Specifically, while maximally localized, symmetric Wannier and magnetic atomic orbitals are not required to have the same radial parts [aside from the Wannier orbital exhibiting exponential or sharper localization], we can establish a correspondence between Wannier and atomic orbitals by restricting focus to the angular parts, which, for symmetrized [sets of] orbitals, necessarily transform in (co)reps of the 122 crystallographic SPGs [see SN 18 and SRefs. 7–18].

Next, because a Wyckoff position generically contains more than one site [*i.e.* the multiplicity of the Wyckoff position $n \geq 1$], then, given a [set of] Wannier orbital[s] that transform in a single irreducible, D-dimensional (co)rep of the site-symmetry group $G_{\mathbf{q}}$, in order to preserve the symmetry of the SSG G, there must additionally be D-dimensional [sets of] Wannier orbitals on each of the $n-1$ additional sites in the Wyckoff position, leading to a total of $n \times D$ Wannier orbitals in each unit cell. For each [set of] orbital[s] at $\mathbf{q}$ that transforms in the (co)rep $\tilde{\rho}_{\mathbf{q}}$ of $G_{\mathbf{q}}$, there is therefore also an orbital [or set of orbitals] at each site $\mathbf{q}' = \tilde{g}\mathbf{q} \bmod \mathbf{t}_{a,b,c}$ for each symmetry $\tilde{g} \in G \setminus G_{\mathbf{q}}$ that transform[s] in an irreducible (co)rep $\tilde{\rho}_{\mathbf{q}'}$ of:

$$G_{\mathbf{q}'} = \tilde{g} G_{\mathbf{q}} \tilde{g}^{-1}, \tag{159}$$

where $G_{\mathbf{q}'}$ is isomorphic and conjugate to $G_{\mathbf{q}}$. It is important to note that even though $G_{\mathbf{q}'}$ is isomorphic to $G_{\mathbf{q}}$, and even though $G_{\mathbf{q}}$ and $G_{\mathbf{q}'}$ are both isomorphic to the same Shubnikov point group (SPG, see SN 8) M, the symmetries $\tilde{g} \in G \setminus G_{\mathbf{q}}$ require that the orbitals [and (co)reps] at $\mathbf{q}'$ are conjugate to those at $\mathbf{q}$. For example, if $\mathbf{q}$ and $\mathbf{q}'$ are related by the symmetry $\{C_{4z}|\mathbf{0}\}$ in an SSG G, then a p_x orbital at $\mathbf{q}$ must be accompanied by a $p_y = C_{4z} p_x C_{4z}^{-1}$ orbital at $\mathbf{q}'$ in order to preserve $\{C_{4z}|\mathbf{0}\} \in G$. Employing the terminology previously established in SRefs. 1–6, this can be summarized by stating that the orbital[s] that transform in $\tilde{\rho}_{\mathbf{q}}$ – along with the orbital[s] that transform[s] in the conjugate (co)reps $\tilde{\rho}_{\mathbf{q}'}$ of each of the other $n-1$ sites in the Wyckoff position of $\mathbf{q}$ – *occupy* the Wyckoff position indexed by $\mathbf{q}$. To formally define the conjugate site-symmetry (co)reps $\tilde{\rho}_{\mathbf{q}'}$, we first establish that, given a unitary symmetry $h \in H_{\mathbf{q}}$ – the maximal unitary subgroup of $G_{\mathbf{q}}$ – the matrix representative of h in $\tilde{\rho}_{\mathbf{k}}$ is denoted as $\Delta_{\tilde{\rho}_{\mathbf{q}}}(h)$, for which the character of h in $\tilde{\rho}_{\mathbf{k}}$ is given by $\text{Tr}[\Delta_{\tilde{\rho}_{\mathbf{q}}}(h)]$. In this notation, it is clear that the matrix representative $\Delta_{\tilde{\rho}_{\mathbf{q}'}}(ghg^{-1})$ of the conjugate symmetry $\tilde{g}h\tilde{g}^{-1} \in G_{\mathbf{q}'}$ does not generically equal $\Delta_{\tilde{\rho}_{\mathbf{q}'}}(h)$ (which itself may not be well defined, because h is not required to be an element of both $G_{\mathbf{q}}$ and $G_{\mathbf{q}'}$). Instead, the matrix representatives of the conjugate symmetries $\tilde{g}h\tilde{g}^{-1} \in G_{\mathbf{q}'}$ are conjugate to the matrix representatives of $h \in G_{\mathbf{q}}$; specifically, if $\tilde{g}$ is unitary, then:

$$\Delta_{\tilde{\rho}_{\mathbf{q}'}}(\tilde{g}h\tilde{g}^{-1}) = \Delta_{\tilde{\rho}_{\mathbf{q}}}(h), \tag{160}$$

and if $\tilde{g}$ is antiunitary, then:

$$\Delta_{\tilde{\rho}_{\mathbf{q}'}}(\tilde{g}h\tilde{g}^{-1}) = \left[\Delta_{\tilde{\rho}_{\mathbf{q}}}(h)\right]^{*}. \tag{161}$$

The central principle of TQC, which we will here extend to MTQC, is that, when a set of of magnetic atomic orbital[s] that transform in an irreducible site-symmetry (co)rep $\tilde{\rho}_{\mathbf{q}}$ occupy the Wyckoff position of $\mathbf{q}$, the orbitals induce a (co)rep of the SSG G:

$$\tilde{\rho}_{\mathbf{q}} \uparrow G = \tilde{\rho}_{\mathbf{q}}^{G}, \tag{162}$$

where $\tilde{\rho}_{\mathbf{q}}^{G}$ is a *band (co)representation* [band (co)rep]. Crucially, the action of induction ($\uparrow$), unlike subduction ($\downarrow$), *does not* preserve dimensionality (*i.e.* the character of the identity element E), such that $\chi_{\tilde{\rho}_{\mathbf{q}}^{G}}(E) \neq \chi_{\tilde{\rho}_{\mathbf{q}}}(E)$. Instead:

$$\chi_{\tilde{\rho}_{\mathbf{q}}^{G}}(E) = \chi_{\tilde{\rho}_{\mathbf{q}}}(E) \times [G : G_{\mathbf{q}}] = \chi_{\tilde{\rho}_{\mathbf{q}}}(E) \times n \times N, \tag{163}$$

where n is the multiplicity of the Wyckoff position indexed by $\mathbf{q}$ and N is the number of unit cells in the crystal. We next take N to be very large (*i.e.* countably infinite), reflecting our goal of applying MTQC to theoretical models of infinite crystals to predict the topology of mesoscopic solid-state systems. The (now infinite) factor of N on the right-hand side of SEq. (163) originates from the infinite subgroup index $[G : G_{\mathbf{q}}]$ of $G_{\mathbf{q}}$ in G [defined in the text surrounding SEq. (10)], which occurs because the site-symmetry group $G_{\mathbf{q}}$ is finite, whereas the SSG G is infinite. This can be seen by recognizing that $G_T \not\subseteq G_{\mathbf{q}}$, $G_T \subseteq G$, in which G_T is the infinite group of 3D lattice translations [SEq. (1)].

Most importantly, as shown in SRef. 4, SEq. (162) can be decomposed into a sum of *full* (co)reps:

$$\tilde{\rho}_{\mathbf{q}}^{G} = \bigoplus_{\tilde{\mathbf{k}}} \tilde{\Sigma}_{\tilde{\mathbf{k}},\mathbf{q}}^{G}, \tag{164}$$

where the sum in SEq. (164) instead runs over each of the points $\tilde{\mathbf{k}}$ in the *irreducible wedge* of the first BZ[134,135], which is defined as the set of points $\tilde{\mathbf{k}}$ in the first BZ containing one and only one arm of each momentum star [see SN 12]. In SEq. (164), $\tilde{\Sigma}_{\tilde{\mathbf{k}},\mathbf{q}}^{G}$ is a generically *reducible* full [*i.e.* space group] (co)rep of the star of the SSG G indexed

by $\tilde{\mathbf{k}}$ [SEq. (83)]. Hence:

$$\tilde{\Sigma}^{G}_{\tilde{\mathbf{k}},\mathbf{q}} = \bigoplus_{i} b_{i}^{\tilde{\mathbf{k}},\mathbf{q}}\tilde{\Sigma}_{i,\tilde{\mathbf{k}}}, \tag{165}$$

where $\tilde{\Sigma}_{i,\tilde{\mathbf{k}}}$ is the ith irreducible full (co)rep of the star of G indexed by $\tilde{\mathbf{k}}$, and where $b_{i}^{\tilde{\mathbf{k}},\mathbf{q}}$ is the multiplicity of $\tilde{\Sigma}_{i,\tilde{\mathbf{k}}}$ in the decomposition of $\tilde{\Sigma}^{G}_{\tilde{\mathbf{k}},\mathbf{q}}$ [*i.e.*, $b_{i}^{\tilde{\mathbf{k}},\mathbf{q}}$ is a non-negative integer that indicates the number of times that the irreducible full (co)rep $\tilde{\Sigma}_{i,\tilde{\mathbf{k}}}$ appears in $\tilde{\Sigma}^{G}_{\tilde{\mathbf{k}},\mathbf{q}}$, see the text surrounding SEq. (108)]. Using SEq. (83), SEq. (164) can be further re-expressed in terms of the generically reducible small (co)reps $\tilde{\sigma}^{G}_{\mathbf{k},\mathbf{q}}$ at each $\mathbf{k}$ point:

$$\tilde{\rho}^{G}_{\mathbf{q}} = \bigoplus_{\tilde{\mathbf{k}}}\tilde{\Sigma}^{G}_{\tilde{\mathbf{k}},\mathbf{q}} = \bigoplus_{\tilde{\mathbf{k}}}\bigoplus_{\mathbf{k}=\tilde{\mathbf{k}}}^{\mathbf{k}_{m_{\tilde{\mathbf{k}}}}}\tilde{\sigma}^{G}_{\mathbf{k},\mathbf{q}} = \bigoplus_{\mathbf{k}}\tilde{\sigma}^{G}_{\mathbf{k},\mathbf{q}} \tag{166}$$

where $m_{\tilde{\mathbf{k}}}$ is the number of arms in the star of $\tilde{\mathbf{k}}$ [see the text surrounding SEq. (58)], such that $\mathbf{k}$ runs from $\tilde{\mathbf{k}}$ to $\mathbf{k}_{m_{\tilde{\mathbf{k}}}}$ for each star indexed by $\tilde{\mathbf{k}}$ in the sum in the second equality, and where the sum on the right-hand side of SEq. (166) runs over each of the N (infinitely many) points $\mathbf{k}$ in the first BZ.

Further intuition for SEqs. (163), (164), and (166), can be obtained by comparing the relative dimensionality of $\tilde{\rho}_{\mathbf{q}}$, $\tilde{\rho}^{G}_{\mathbf{q}}$, $\tilde{\Sigma}^{G}_{\tilde{\mathbf{k}},\mathbf{q}}$, and $\tilde{\sigma}^{G}_{\mathbf{k},\mathbf{q}}$. First, while $\chi_{\tilde{\rho}^{G}_{\mathbf{q}}}(E)$ is infinite [SEq. (163)], the component $\tilde{\Sigma}^{G}_{\tilde{\mathbf{k}},\mathbf{q}}$ in the Fourier decomposition of the band (co)rep $\tilde{\rho}^{G}_{\mathbf{q}}$ in SEq. (165) is *finite-dimensional*, and there are instead an infinite number of [generically reducible] full (co)reps $\tilde{\Sigma}^{G}_{\tilde{\mathbf{k}},\mathbf{q}}$ – one at each of the infinitely many $\tilde{\mathbf{k}}$ points in the irreducible wedge of the first BZ [defined in the text following SEq. (164)]. To see this, we compute the dimensionality of $\tilde{\Sigma}^{G}_{\tilde{\mathbf{k}},\mathbf{q}}$, which is defined as the character of the identity operation E:

$$\chi_{\tilde{\Sigma}^{G}_{\tilde{\mathbf{k}},\mathbf{q}}}(E) = \chi_{\tilde{\rho}_{\mathbf{q}}}(E)\times n\times m_{\tilde{\mathbf{k}}}, \tag{167}$$

in which n is the multiplicity of the Wyckoff position indexed by $\mathbf{q}$ (SN 9), and $m_{\tilde{\mathbf{k}}}$ is the number of arms in the star of $\tilde{\mathbf{k}}$ [see the text surrounding SEq. (58)]. Conversely, the [generically reducible] small (co)rep $\tilde{\sigma}^{G}_{\mathbf{k},\mathbf{q}}$ in SEq. (166) generically has a smaller (finite) dimensionality than $\tilde{\Sigma}^{G}_{\tilde{\mathbf{k}},\mathbf{q}}$. To see this, we first subduce $\tilde{\sigma}^{G}_{\mathbf{k},\mathbf{q}}$ onto the little group $G_{\mathbf{k}}$:

$$\tilde{\sigma}^{G}_{\mathbf{k},\mathbf{q}}\downarrow G_{\mathbf{k}} = \tilde{\varsigma}_{\mathbf{k},\mathbf{q}}, \tag{168}$$

where $\tilde{\varsigma}_{\mathbf{k},\mathbf{q}}$ is the symmetry data [see the text following SEq. (108)] induced by the (co)rep $\tilde{\rho}_{\mathbf{q}}$ of the site-symmetry group $G_{\mathbf{q}}$ into the SSG G [SEq. (162)] and then subduced onto the little group $G_{\mathbf{k}}$ of the point $\mathbf{k}$ in the first BZ. We note that, because $\tilde{\sigma}^{G}_{\mathbf{k},\mathbf{q}}$ is already a [generically reducible] small (co)rep of $G_{\mathbf{k}}$ [SEq. (166)], then $\tilde{\sigma}^{G}_{\mathbf{k},\mathbf{q}}\downarrow G_{\mathbf{k}}$ in SEq. (168) is a redundant expression. However, in this work, we will continue to employ the expression $\tilde{\sigma}^{G}_{\mathbf{k},\mathbf{q}}\downarrow G_{\mathbf{k}}$ on the left-hand side of SEq. (168) for consistency with earlier works on TQC[1,4]. Next, we compute the dimensionality of the subduced symmetry data $\tilde{\varsigma}_{\mathbf{k},\mathbf{q}}$:

$$\chi_{\tilde{\varsigma}_{\mathbf{k},\mathbf{q}}}(E) = \chi_{\tilde{\rho}_{\mathbf{q}}}(E)\times n, \tag{169}$$

where n continues to be the multiplicity of the Wyckoff position indexed by $\mathbf{q}$ (SN 9). Physically, because the set of site-symmetry (co)reps $\{\tilde{\rho}_{\mathbf{q}_{\alpha}}\}$ corresponds to $\chi_{\tilde{\rho}_{\mathbf{q}}}(E)\times n$ magnetic atomic [Wannier] orbitals [SN 18] occupying the n sites $\mathbf{q}_{\alpha}$ in the Wyckoff position of $\mathbf{q}$, and therefore characterizes $\chi_{\tilde{\rho}_{\mathbf{q}}}(E)\times n$ bands in momentum space, then the subduced symmetry data $\tilde{\varsigma}_{\mathbf{k},\mathbf{q}}$ [SEq. (168)] correspond to a (set of) $\chi_{\tilde{\rho}_{\mathbf{q}}}(E)\times n$ *Bloch* states at $\mathbf{k}$. This can be summarized by the statement that the $\chi_{\tilde{\rho}_{\mathbf{q}}}(E)\times n$ Bloch states at $\mathbf{k}$ *transform* in $\tilde{\varsigma}_{\mathbf{k},\mathbf{q}}$, analogous to the correspondence between orbitals and position-space SPG [site-symmetry group] (co)reps established in SN 18.

Though $\tilde{\rho}_{\mathbf{q}}$ is an irreducible (co)rep of the site-symmetry group $G_{\mathbf{q}}$, this *does not* imply that $\tilde{\varsigma}_{\mathbf{k},\mathbf{q}} = \tilde{\sigma}^{G}_{\mathbf{k},\mathbf{q}}\downarrow G_{\mathbf{k}}$ in [SEq. (166)] is an *irreducible* small (co)rep of $G_{\mathbf{k}}$. In fact, generically, $\tilde{\varsigma}_{\mathbf{k},\mathbf{q}}$ is a *reducible* small (co)rep of $G_{\mathbf{k}}$, such that:

$$\tilde{\sigma}^{G}_{\mathbf{k},\mathbf{q}}\downarrow G_{\mathbf{k}} = \tilde{\varsigma}_{\mathbf{k},\mathbf{q}} = \bigoplus_{j} a_{j}^{\mathbf{k},\mathbf{q}}\tilde{\sigma}_{j,\mathbf{k}}, \tag{170}$$

where $\tilde{\sigma}_{j,\mathbf{k}}$ is the jth irreducible small (co)rep of $G_{\mathbf{k}}$ and $a_j^{\mathbf{k},\mathbf{q}}$ is a non-negative integer corresponding to the multiplicity of $\tilde{\sigma}_{j,\mathbf{k}}$ in the decomposition of $\tilde{\varsigma}_{\mathbf{k},\mathbf{q}}$. To obtain the multiplicities $a_j^{\mathbf{k},\mathbf{q}}$ in SEq. (170), we can re-express SEq. (170) in terms of the characters $\chi_{\tilde{\varsigma}_{\mathbf{k},\mathbf{q}}}(h_i)$ and $\chi_{\tilde{\sigma}_{j,\mathbf{k}}}(h_i)$ of each of the unitary symmetries $h_i \in |\tilde{H}_{\mathbf{k}}|$, the maximal unitary subset of the set of coset representatives $\tilde{G}_{\mathbf{k}}$ [text preceding SEq. (71)] of $G_{\mathbf{k}}$ with respect to the group of lattice translations G_T [SEq. (1)]:

$$\chi_{\tilde{\varsigma}_{\mathbf{k},\mathbf{q}}}(h_i) = \sum_j a_j^{\mathbf{k},\mathbf{q}} \chi_{\tilde{\sigma}_{j,\mathbf{k}}}(h_i). \tag{171}$$

As we will show below, it is important to emphasize that the values of $a_j^{\mathbf{k},\mathbf{q}}$ in SEqs. (170) and (171) are determined by the choice of the (co)rep $\tilde{\rho}_{\mathbf{q}}$ of the site-symmetry group $G_{\mathbf{q}}$ [*i.e.* the (magnetic) atomic orbitals occupying the Wyckoff position indexed by $\mathbf{q}$] in SEq. (162). This can be seen by first recognizing the values of $\tilde{\sigma}_{j,\mathbf{k}}$ in SEqs. (170) and (171) are limited to the finite set of small (co)reps of $G_{\mathbf{k}}$, which can be obtained through the Corepresentations tool, as previously described in SN 13. Next, we recognize that $\tilde{\varsigma}_{\mathbf{k},\mathbf{q}}$ is a component of the Fourier decomposition of the induced band (co)rep $\tilde{\rho}_{\mathbf{q}}^G = \tilde{\rho}_{\mathbf{q}} \uparrow G$ [SEqs. (162) and (166)]. Specifically, SEqs. (162) and (166) imply that, for a given little group $G_{\mathbf{k}}$, the characters $\chi_{\tilde{\varsigma}_{\mathbf{k},\mathbf{q}}}(h_i)$ for each unitary symmetry $h_i \in \tilde{H}_{\mathbf{k}}$ [the maximal unitary subset of $\tilde{G}_{\mathbf{k}}$, see the text preceding SEq. (71)] are given by:

$$\chi_{\tilde{\varsigma}_{\mathbf{k},\mathbf{q}}}(h_i) = \sum_{\alpha=1}^{n} \chi_{\tilde{\varsigma}_{\mathbf{k},\mathbf{q}_\alpha}}(h_i), \tag{172}$$

where α runs over each of the n sites $\mathbf{q}_\alpha$ in the Wyckoff position of $\mathbf{q}$ (including $\mathbf{q}$ itself, see SN 9), and where, as will shortly be detailed below:

$$\chi_{\tilde{\varsigma}_{\mathbf{k},\mathbf{q}_\alpha}}(h_i) = \begin{cases} e^{-i\mathbf{k}\cdot(\mathbf{q}_\alpha - h_i\mathbf{q}_\alpha)} \chi_{\tilde{\rho}_{\mathbf{q}_\alpha}}\left(\{E|\mathbf{q}_\alpha - h_i\mathbf{q}_\alpha\}\, h_i\right) & , \text{ if } \{E|\mathbf{q}_\alpha - h_i\mathbf{q}_\alpha\}\, h_i \in G_{\mathbf{q}_\alpha} \\ 0 & , \text{ if } \{E|\mathbf{q}_\alpha - h_i\mathbf{q}_\alpha\}\, h_i \notin G_{\mathbf{q}_\alpha} \end{cases}. \tag{173}$$

When $\chi_{\tilde{\varsigma}_{\mathbf{k},\mathbf{q}_\alpha}}(h_i) \neq 0$ in SEq. (173), the vectors $\mathbf{q}_\alpha - h_i\mathbf{q}_\alpha$ are necessarily integer-valued linear combinations of lattice vectors [*i.e.* $\{E|\mathbf{q}_\alpha - h_i\mathbf{q}_\alpha\} \in G_T$, where G_T is defined in SEq. (1)][4]. This occurs because the symmetries $h_i \in \tilde{H}_{\mathbf{k}}$ may shift the location of a site $\mathbf{q}_\alpha$ in the Wyckoff position of $\mathbf{q}$ to a site $h_i\mathbf{q}_\alpha$ in an adjacent unit cell that only differs from $\mathbf{q}_\alpha$ by a linear combination of lattice vectors (if $\mathbf{q}_\alpha - h_i\mathbf{q}_\alpha$ were not a lattice vector, then $\{E|\mathbf{q}_\alpha - h_i\mathbf{q}_\alpha\}h_i$ would instead be one of the symmetries $\{E|\mathbf{q}_\alpha - h_i\mathbf{q}_\alpha\}h_i \notin G_{\mathbf{q}_\alpha}$ that exchanges sites within the Wyckoff position of $\mathbf{q}$, and $\chi_{\tilde{\rho}_{\mathbf{q}_\alpha}}(\{E|\mathbf{q}_\alpha - h_i\mathbf{q}_\alpha\}h_i)$ in SEq. (173) would not be well defined). The (co)reps $\tilde{\rho}_{\mathbf{q}_\alpha}$ of the sites $\mathbf{q}_\alpha$ in SEqs. (172) and (173) are determined from the site-symmetry (co)rep $\tilde{\rho}_{\mathbf{q}}$ by conjugation with the symmetries $\tilde{g} \in G$, $\tilde{g} \notin G_{\mathbf{q}}$, as described in the text surrounding SEqs. (160) and (161).

Finally, using SEqs. (172) and (173) for each of the unitary symmetries $h_i \in \tilde{H}_{\mathbf{k}}$ [SEq. (71)], we obtain $|\tilde{H}_{\mathbf{k}}|$ equations of the form of SEq. (171) for the multiplicities $a_j^{\mathbf{k},\mathbf{q}}$, which can be condensed into a matrix equation in which the summation over j in SEq. (171) is implicit:

$$\boldsymbol{\chi}_{\tilde{\varsigma}_{\mathbf{k},\mathbf{q}}} = \mathcal{G}_{\mathbf{k}} \mathbf{a}^{\mathbf{k},\mathbf{q}}, \tag{174}$$

where $\boldsymbol{\chi}_{\tilde{\varsigma}_{\mathbf{k},\mathbf{q}}}$ is an $|\tilde{H}_{\mathbf{k}}| \times 1$-dimensional column vector whose i^{th} entry is the value of $\chi_{\tilde{\varsigma}_{\mathbf{k},\mathbf{q}}}(h_i)$ inherited from the site-symmetry group (co)rep $\tilde{\rho}_{\mathbf{q}}$ through SEqs. (172) and (173), and where $\mathbf{a}^{\mathbf{k},\mathbf{q}}$ is an $l \times 1$-dimensional column vector whose j^{th} entry is the multiplicity $a_j^{\mathbf{k},\mathbf{q}}$ of the small (co)rep $\tilde{\sigma}_{j,\mathbf{k}}$ of the little group $G_{\mathbf{k}}$ in the decomposition of the subduced symmetry data $\tilde{\varsigma}_{\mathbf{k},\mathbf{q}}$, where l is the number of small (co)reps of $G_{\mathbf{k}}$. In SEq. (174), $\mathcal{G}_{\mathbf{k}}$ is an $|\tilde{H}_{\mathbf{k}}| \times l$-dimensional, generically non-square matrix whose ij^{th} element is given by the character of the unitary symmetry $h_i \in G_{\mathbf{k}}$ in the small (co)rep $\tilde{\sigma}_{j,\mathbf{k}}$ of $G_{\mathbf{k}}$:

$$[\mathcal{G}_{\mathbf{k}}]_{ij} = \chi_{\tilde{\sigma}_{j,\mathbf{k}}}(h_i). \tag{175}$$

Consequently, $\mathcal{G}_{\mathbf{k}}$ is simply the transpose of the character table for $G_{\mathbf{k}}$ (see SFigs. 9, 10, and 11 and Supplementary Table 2, for example). Crucially, because the rows (and columns) of character tables are orthogonal[33,128], then the

columns (and rows) of $\mathcal{G}_{\mathbf{k}}$ are also orthogonal. This implies that the left inverse $\mathcal{G}_{\mathbf{k}}^{-1}$ of $\mathcal{G}_{\mathbf{k}}$ is simply given by:

$$\mathcal{G}_{\mathbf{k}}^{-1} = \frac{1}{|\tilde{H}_{\mathbf{k}}|}\mathcal{G}_{\mathbf{k}}^{\dagger}, \tag{176}$$

such that:

$$\mathcal{G}_{\mathbf{k}}^{\dagger}\mathcal{G}_{\mathbf{k}} = |\tilde{H}_{\mathbf{k}}|\mathbb{1}, \tag{177}$$

where $\mathbb{1}$ in SEq. (177) is the $l \times l$ identity. As a final step, we left-multiply SEq. (174) by $\mathcal{G}_{\mathbf{k}}^{-1}$ [SEq. (176)] to solve for $\mathbf{a}^{\mathbf{k},\mathbf{q}}$:

$$\mathbf{a}^{\mathbf{k},\mathbf{q}} = \frac{1}{|\tilde{H}_{\mathbf{k}}|}\mathcal{G}_{\mathbf{k}}^{\dagger}\boldsymbol{\chi}_{\tilde{\varsigma}_{\mathbf{k},\mathbf{q}}}, \tag{178}$$

thus obtaining the multiplicities $a_j^{\mathbf{k},\mathbf{q}}$ in SEqs. (170) and (171). We note that SEq. (178) is in fact the matrix form of the Schur orthogonality relation (*i.e.* the so-called "magic formula")[2].

For this work, we have implemented the MSITESYM tool on the BCS to output the multiplicities [$a_j^{\mathbf{k},\mathbf{q}}$ in SEqs. (170) and (171)] of the small (co)reps $\tilde{\sigma}_{j,\mathbf{k}}$ subduced in the little group $G_{\mathbf{k}}$ of each $\mathbf{k}$ point [SEq. (168)] from the band (co)rep $\tilde{\rho}_{\mathbf{q}}^{G}$ induced into each SSG G [SEq. (162)] from each irreducible (co)rep $\tilde{\rho}_{\mathbf{q}}$ of one site-symmetry group $G_{\mathbf{q}}$ in each Wyckoff position of G. MSITESYM subsumes the earlier DSITESYM tool (`https://www.cryst.ehu.es/cgi-bin/cryst/programs/dsitesym.pl`)[1–3], which was previously implemented for TQC to provide direct access to the single- and double-valued small irreps subduced onto a given $G_{\mathbf{k}}$ from the band rep $\rho_{\mathbf{q}}^{G}$ induced from each site-symmetry irrep $\rho_{\mathbf{q}}$ in each of the 230 Type-I MSGs. In SFig. 16, we show the output of MSITESYM for Type-III MSG 75.3 $P4'$ at the A point in momentum space and the $1b$ Wyckoff position in position space.

In summary, we have demonstrated in this section how decoupled Wannier orbitals that transform in site-symmetry (co)reps in position space induce band (co)reps [SEq. (162)], which in turn subduce small (co)reps at each point in momentum space that correspond to Bloch states (bands) [SEq. (168)]. It is straightforward to see that, if additional Wannier orbitals are added that either transform in different (co)reps of site-symmetry groups in the same Wyckoff position, or occupy a different Wyckoff position, then additional bands will also be present in the energy spectrum, corresponding to additional small (co)reps in the symmetry data at each $\mathbf{k}$ point. Therefore, we have also shown that

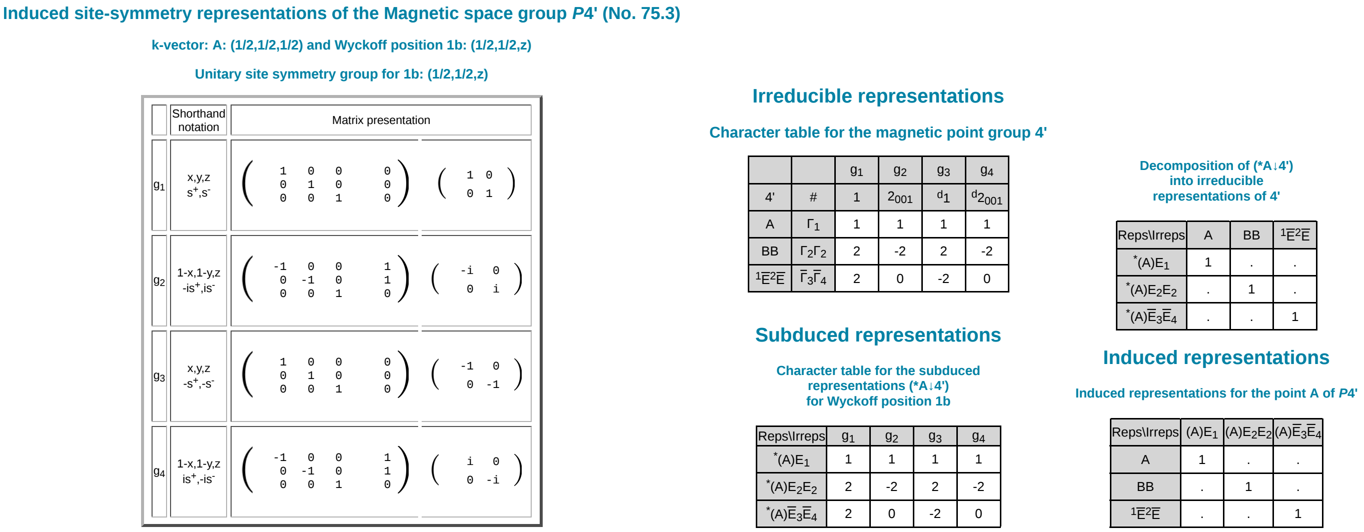

Induced site-symmetry representations of the Magnetic space group *P4'* (No. 75.3)

k-vector: A: (1/2,1/2,1/2) and Wyckoff position 1b: (1/2,1/2,z)

Unitary site symmetry group for 1b: (1/2,1/2,z)

	Shorthand notation	Matrix presentation	
g_1	x,y,z<br>s^+,s^-	$\begin{pmatrix} 1 & 0 & 0 & 0 \\ 0 & 1 & 0 & 0 \\ 0 & 0 & 1 & 0 \end{pmatrix}$	$\begin{pmatrix} 1 & 0 \\ 0 & 1 \end{pmatrix}$
g_2	1-x,1-y,z<br>$-is^+,is^-$	$\begin{pmatrix} -1 & 0 & 0 & 1 \\ 0 & -1 & 0 & 1 \\ 0 & 0 & 1 & 0 \end{pmatrix}$	$\begin{pmatrix} -i & 0 \\ 0 & i \end{pmatrix}$
g_3	x,y,z<br>$-s^+,-s^-$	$\begin{pmatrix} 1 & 0 & 0 & 0 \\ 0 & 1 & 0 & 0 \\ 0 & 0 & 1 & 0 \end{pmatrix}$	$\begin{pmatrix} -1 & 0 \\ 0 & -1 \end{pmatrix}$
g_4	1-x,1-y,z<br>$is^+,-is^-$	$\begin{pmatrix} -1 & 0 & 0 & 1 \\ 0 & -1 & 0 & 1 \\ 0 & 0 & 1 & 0 \end{pmatrix}$	$\begin{pmatrix} i & 0 \\ 0 & -i \end{pmatrix}$

Irreducible representations

Character table for the magnetic point group 4'

		g_1	g_2	g_3	g_4
4'	#	1	2_{001}	$^{d}1$	$^{d}2_{001}$
A	Γ_1	1	1	1	1
BB	$\Gamma_2\Gamma_2$	2	-2	2	-2
$^1\overline{E}{}^2\overline{E}$	$\overline{\Gamma}_3\overline{\Gamma}_4$	2	0	-2	0

Decomposition of (*A↓4') into irreducible representations of 4'

Reps\Irreps	A	BB	$^1\overline{E}{}^2\overline{E}$
*(A)E_1	1	.	.
*(A)E_2E_2	.	1	.
*(A)$\overline{E}_3\overline{E}_4$	.	.	1

Subduced representations

Character table for the subduced representations (*A↓4') for Wyckoff position 1b

Reps\Irreps	g_1	g_2	g_3	g_4
*(A)E_1	1	1	1	1
*(A)E_2E_2	2	-2	2	-2
*(A)$\overline{E}_3\overline{E}_4$	2	0	-2	0

Induced representations

Induced representations for the point A of *P4'*

Reps\Irreps	(A)E_1	(A)E_2E_2	(A)$\overline{E}_3\overline{E}_4$
A	1	.	.
BB	.	1	.
$^1\overline{E}{}^2\overline{E}$	.	.	1

Supplementary Figure 16: The output of the MSITESYM tool on the BCS for Type-III MSG 75.3 $P4'$ at the A point in momentum space and the $1b$ Wyckoff position in position space. For one $\mathbf{k}$ point in each momentum star (see SN 12) and one site $\mathbf{q}$ in each Wyckoff position in each SSG (see SN 9), MSITESYM outputs the irreducible (co)reps of the site-symmetry group $G_{\mathbf{q}}$ (see SN 18), the small (co)reps of the little group $G_{\mathbf{k}}$ (see SN 13), and the multiplicities $a_j^{\mathbf{k},\mathbf{q}}$ in SEqs. (170) and (171). MSITESYM subsumes the earlier DSITESYM tool[1–3], which was previously implemented for TQC to provide direct access to the single- and double-valued small irreps subduced onto a given $G_{\mathbf{k}}$ from the band rep induced from each site-symmetry irrep in each of the 230 Type-I MSGs.

arbitrary sets of bands induced from Wannier orbitals transform in a linear combination of band (co)reps. In the subsequent section, SN 23, we will determine the minimal, or *elementary*, band (co)reps [EBRs, composed of PEBRs in Type-II SSGs and MEBRs in Type-I, III, and IV MSGs][1–6,11,12,20–22] that span all linear combinations of band (co)reps induced from maximally localized, symmetric Wannier orbitals.

23. Computing the MEBRs and the Exceptional Cases and Introduction to the MBANDREP Tool

In this section, we will use the results of SN 22 to determine which of the induced band (co)reps in each SSG are *elementary* – which we will rigorously define in this section – thus establishing the complete theory of MTQC. We will specifically obtain the MEBRs of the Type-III and Type-IV MSGs, which, along with the MEBRs of the Type-I MSGs and the PEBRs of the Type-II SSGs previously tabulated in SRefs. 1,4, form the complete set of EBRs of all of the 1,651 single and double SSGs. We note that previously in TQC[1–6], the Type-I MEBRs of the Type-I MSGs were termed EBRs, to draw contrast with the PEBRs of the Type-II SSGs. However, in this work, we will revise the previous terminology to accomodate the elementary band coreps of the Type-III and IV MSGs – in this work, all elementary band (co)reps are in general termed EBRs, the elementary band coreps of Type-II SSGs remain termed PEBRs, and the elementary band (co)reps of Type-I, III, and IV MSGs are respectively termed Type-I, III, and IV MEBRs. Finally, we note that prior to this work, Evarestov Smirnov, and Egorov in SRef. 12 introduced a method for obtaining the MEBRs of the MSGs and computed representative examples, but did not perform a large-scale tabulation of MEBRs – the calculations performed in this section represent the first *complete* tabulation of the MEBRs of the 1,421 single and double MSGs.

To begin, we previously established in SN 22 that, if a set of [magnetic] atomic orbitals transforming in an irreducible (co)rep $\tilde{\rho}_{\mathbf{q},1}$ of a site-symmetry group $G_{\mathbf{q}}$ is placed at $\mathbf{q}$ in each unit cell of a crystal that is invariant under an SSG G, then $\tilde{\rho}_{\mathbf{q},1}$ induces a band (co)rep $\tilde{\rho}^G_{\mathbf{q},1} = \tilde{\rho}_{\mathbf{q},1} \uparrow G$ [SEq. (162)]. From this, we may then consider the case in which additional orbitals are subsequently added at $\mathbf{q}$ that transform in the (co)rep $\tilde{\rho}_{\mathbf{q},2}$, such that the total set of Wannier orbitals at $\mathbf{q}$ transforms in the *reducible* site-symmetry (co)rep $\tilde{\rho}_{\mathbf{q},T} = \tilde{\rho}_{\mathbf{q},1} \oplus \tilde{\rho}_{\mathbf{q},2}$. Because representation induction is distributive[4], then it follows that:

$$\tilde{\rho}_{\mathbf{q},T} \uparrow G = (\tilde{\rho}_{\mathbf{q},1} \oplus \tilde{\rho}_{\mathbf{q},2}) \uparrow G = \tilde{\rho}^G_{\mathbf{q},T}, \tag{179}$$

such that:

$$\tilde{\rho}^G_{\mathbf{q},T} = (\tilde{\rho}_{\mathbf{q},1} \uparrow G) \oplus (\tilde{\rho}_{\mathbf{q},2} \uparrow G) = \tilde{\rho}^G_{\mathbf{q},1} \oplus \tilde{\rho}^G_{\mathbf{q},2}. \tag{180}$$

SEq. (180) implies that $\tilde{\rho}^G_{\mathbf{q},T}$ is a *composite band (co)rep*, because $\tilde{\rho}^G_{\mathbf{q},T}$ is equivalent to a sum of two other band (co)reps [$\tilde{\rho}^G_{\mathbf{q},1}$ and $\tilde{\rho}^G_{\mathbf{q},2}$]. In this work, we define two band (co)reps $\tilde{\rho}^G_{\mathbf{q},T}$ and $\tilde{\rho}^G_{\mathbf{q},1} \oplus \tilde{\rho}^G_{\mathbf{q},2}$ to be equivalent through the existence of a relation of the form of SEq. (180). If two band (co)reps $\tilde{\rho}^G_{\mathbf{q},1}$ and $\tilde{\rho}^G_{\mathbf{q},2}$ are equivalent, then this also implies the existence of a unitary matrix-valued function $S(\mathbf{k}, t, h)$ that is smooth and non-singular in $\mathbf{k}$ and continuous in t that interpolates for each unitary symmetry $h \in G$ between the full [space group] (co)rep matrix representatives $\Delta_{\tilde{\Sigma}^G_{\mathbf{k},\mathbf{q},T}}(h)$ $[t = 0]$ and $\Delta_{\tilde{\Sigma}^G_{\mathbf{k},\mathbf{q},1} \oplus \tilde{\Sigma}^G_{\mathbf{k},\mathbf{q},2}}(h)$ $[t = 1]$ in the decomposition [see the text surrounding SEqs. (83) and (164) and SRefs. 1–6,11,12,20–22 for further details]:

$$\tilde{\rho}^G_{\mathbf{q},T} = \bigoplus_{\mathbf{k}} \tilde{\Sigma}^G_{\mathbf{k},\mathbf{q},T},\ \ \tilde{\rho}^G_{\mathbf{q},1} \oplus \tilde{\rho}^G_{\mathbf{q},2} = \bigoplus_{\mathbf{k}} \tilde{\Sigma}^G_{\mathbf{k},\mathbf{q},1} \oplus \tilde{\Sigma}^G_{\mathbf{k},\mathbf{q},2}. \tag{181}$$

If a band (co)rep is not equivalent to a direct sum of other band reps, then we define the band (co)rep to be *elementary* [*i.e.*, an EBR][1,4,83–85].

In order to complete the theory of MTQC, we must perform a complete enumeration of the EBRs in all of the 1,651 single and double SSGs. Specifically, because EBRs are induced from (magnetic) Wannier orbitals (SN 22), then any set of bands that transforms in a direct sum of EBRs is Wannierizable, and therefore, does not exhibit stable or fragile[136–148] topology[1–6]. With complete knowledge of the EBRs, we will then be able to identify the bands that do not transform in linear combinations of EBRs, which, as we will show in SN 26 correspond to stable topological (crystalline) insulators and topological semimetals.

To obtain an initial bound on the sites in each SSG from which EBRs may be induced, we first recognize that, if a site $\mathbf{q}_0$ indexes a Wyckoff position that is non-maximal, then $G_{\mathbf{q}_0} \subset G_{\mathbf{q}}$ where $\mathbf{q}$ is a site in a maximal Wyckoff position that is connected to the Wyckoff position containing $\mathbf{q}_0$ (see SN 9 for definitions of connected and maximal Wyckoff positions). Taking $\tilde{\rho}_{\mathbf{q}_0}$ to be (co)rep of the site-symmetry group $G_{\mathbf{q}_0}$, then, through the transitive property

of induction[4]:

$$\tilde{\rho}_{\mathbf{q},0}\uparrow G=\tilde{\rho}^G_{\mathbf{q},0}=\tilde{\rho}_{\mathbf{q},0}\uparrow G_{\mathbf{q}}\uparrow G=\left(\bigoplus_{i=1}^{z}b_i^{\mathbf{q}_0,\mathbf{q}}\tilde{\rho}_{\mathbf{q},i}\right)\uparrow G=\bigoplus_{i=1}^{z}b_i^{\mathbf{q}_0,\mathbf{q}}\tilde{\rho}^G_{\mathbf{q},i}, \tag{182}$$

where z is the number of unique irreducible (co)reps $\tilde{\rho}_{\mathbf{q},i}$ in $G_{\mathbf{q}}$, $b_i^{\mathbf{q}_0,\mathbf{q}}$ is a non-negative integer, and where at least one $b_i^{\mathbf{q}_0,\mathbf{q}}$ is nonzero. SEq. (182) implies that any band (co)rep $\tilde{\rho}^G_{\mathbf{q},0}$ induced from a site $\mathbf{q}_0$ in a non-maximal Wyckoff position is equivalent to a sum of band (co)reps induced from a site $\mathbf{q}$ in a maximal Wyckoff position; therefore $\tilde{\rho}^G_{\mathbf{q},0}$ is either a composite band (co)rep, or is equivalent to an EBR induced from $\mathbf{q}$. Consequently, the complete set of EBRs is contained within the set of band (co)reps induced from the sites of the maximal Wyckoff positions of each SSG.

Hence, in this work, we will obtain the EBRs of all single and double SSGs in two steps. First, we will restrict consideration to the band (co)reps induced by the irreducible (co)reps of the site-symmetry groups of the maximal Wyckoff positions of each SSG. We will then in SN 24 filter out the composite band (co)reps induced from sites in maximal Wyckoff positions, which are known as the *exceptional cases*[3,4,83–86]; the remaining band (co)reps comprise the EBRs. In SN 25, we will then provide additional statistics for the EBRs of all SSGs – including the MEBRs of the Type-III and IV MSGs introduced in this work – as well as detail the MBANDREP tool on the BCS that we have implemented for this work to access the EBRs and composite band (co)reps induced from each Wyckoff position in each of the 1,651 single and double SSGs.

24. Exceptional Cases in the MSGs

In most cases, when a (co)rep $\tilde{\rho}_{\mathbf{q}}$ of a site-symmetry group $G_{\mathbf{q}}$ in a maximal Wyckoff position [see SN 9] is induced into an SSG G, the resulting band (co)rep $\tilde{\rho}^G_{\mathbf{q}}=\tilde{\rho}_{\mathbf{q}}\uparrow G$ [SEq. (162)] is an EBR [defined in the text following SEq. (180)]. However, in some *exceptional* cases, $\tilde{\rho}^G_{\mathbf{q}}=\tilde{\rho}_{\mathbf{q}}\uparrow G$ is instead a *composite* band (co)rep. In SRef. 4, it was determined that exceptional cases specifically occur under the following conditions:

1. Two maximal Wyckoff positions indexed by $\mathbf{q}$ and $\mathbf{q}'$ in an SSG G are both connected to the same site $\mathbf{q}_0$ in a non-maximal Wyckoff position. In SRef. 4, $G_{\mathbf{q}'}$ is termed the *reducing group*, and $G_{\mathbf{q}_0}=G_{\mathbf{q}}\cap G_{\mathbf{q}'}$ is termed the *intersection group*.

2. There exists an irreducible (co)rep $\tilde{\rho}_{\mathbf{q}_0}$ of $G_{\mathbf{q}_0}$ for which $\tilde{\rho}_{\mathbf{q}_0}\uparrow G_{\mathbf{q}}$ is equivalent to an irreducible (co)rep of $G_{\mathbf{q}}$.

3. For the same irreducible (co)rep $\tilde{\rho}_{\mathbf{q}_0}$ of $G_{\mathbf{q}_0}$, $\tilde{\rho}_{\mathbf{q}_0}\uparrow G_{\mathbf{q}'}$ is equivalent to a *reducible* (co)rep of $G_{\mathbf{q}'}$.

These three conditions may be summarized through the equivalence relations:

$$\tilde{\rho}_{\mathbf{q}_0}\uparrow G_{\mathbf{q}}\uparrow G=\tilde{\rho}_{\mathbf{q}}\uparrow G=\tilde{\rho}^G_{\mathbf{q}}=\tilde{\rho}_{\mathbf{q}_0}\uparrow G_{\mathbf{q}'}\uparrow G=\tilde{\rho}_{\mathbf{q}'}\uparrow G=\tilde{\rho}^G_{\mathbf{q}'}, \tag{183}$$

in which $\tilde{\rho}_{\mathbf{q}'}$ is a reducible (co)rep of $G_{\mathbf{q}'}$, such that $\tilde{\rho}^G_{\mathbf{q}'}$ is a composite band (co)rep, implying that the equivalent band (co)rep $\tilde{\rho}^G_{\mathbf{q}}$ is also a composite band (co)rep, despite $\tilde{\rho}_{\mathbf{q}}$ being an irreducible (co)rep of $G_{\mathbf{q}}$.

In the Type-I and Type-II SSGs previously analyzed in TQC[1–6], the exceptional cases all occurred in SSGs with point groups that were either isomorphic to Type-I MPG 8.1.24 mmm or to MPGs with higher-fold rotation, rotoinversion, or $\mathcal{T}$ symmetries [*c.f.* Tables S10, S11, and S12 in SRef. 1]. Conversely, in this work, we find there are exceptional composite band coreps in some of the lowest-symmetry Type-III and Type-IV MSGs. Previously in TQC[1–6], it was specifically recognized that if two maximal Wyckoff positions in the same symmetry group have the same multiplicity, but the band (co)reps induced from the Wyckoff positions have different dimensionality, then it is possible that at least one of the induced band (co)reps is composite. In this section we will consider the example of double magnetic rod group [MRG] $(p_c\bar{1})_{RG}$ [SFig. 17], which we have selected because the $2a$ and $2b$ Wyckoff positions both have a multiplicity of 2, but the band coreps induced from $2a$ are two-dimensional, whereas the band corep induced from $2b$ is four-dimensional [and indeed exceptional-case composite].

MRG $(p_c\bar{1})_{RG}$ is generated by:

$$\{\mathcal{I}|0\},\ \{\mathcal{T}|1/2\}, \tag{184}$$

and is isomorphic after the addition of perpendicular lattice translations to Type-IV double MSG 2.7 $P_S\bar{1}$ [see SRefs. 10,33,69–71 and the text following SEq. (2)]. Using MWYCKPOS on the BCS[15–18] for Type-IV MSG 2.7 $P_S\bar{1}$ and restricting to Wyckoff positions with $x=y=0$ in the reduced notation of MWYCKPOS, we obtain the

coordinates and site-symmetry-group-isomorphic MPGs of the Wyckoff positions of MRG $(p_c\bar{1})_{RG}$:

$$\begin{aligned}\mathbf{q}_{2a} &= 0,\ 1/2,\ G_{2a}=\bar{1},\\ \mathbf{q}_{2b} &= 1/4,\ 3/4,\ G_{2b}=\bar{1}',\\ \mathbf{q}_{4c} &= z,\ (1/2)-z,\ (1/2)+z,\ 1-z,\ G_{4c}=1,\end{aligned} \tag{185}$$

where we have employed units in which $a_z = 1$ in SFig. 17. In SEq. (185), the symbols $\bar{1}$, $\bar{1}'$, and 1 respectively refer to Type-I MPG 2.1.3 $\bar{1}$, Type-III MPG 2.3.5 $\bar{1}'$, and Type-I MPG 1.1.1 1 [the trivial MPG, see the text following SEq. (27)]. In SEq. (185), the $2a$ and $2b$ positions are maximal, whereas $4c$ is the (non-maximal) general position. First, we will examine the site-symmetry groups of the $\mathbf{q}_{2a}$ maximal Wyckoff position, which are isomorphic to Type-I double MPG 2.1.3 $\bar{1}$. G_{2a} contains only four symmetry operations and is equal to its maximal unitary subgroup H_{2a}:

$$G_{2a} = H_{2a} = \left\{\{E|0\},\ \{\mathcal{I}|0\},\ \{\bar{E}|0\},\ \{\bar{E}\mathcal{I}|0\}\right\}, \tag{186}$$

where E is the identity operation, and $\bar{E} = C_{1n}$ is the symmetry operation of 360° rotation about an arbitrary axis n, which distinguishes single-valued (spinless) and double-valued (spinful) coreps. Using the CorepresentationsPG tool on the BCS for MPG 2.1.3 $\bar{1}$, we determine that there are only two double-valued irreducible coreps of G_{2a}:

$$\tilde{\rho}_{2a} = \left(\bar{A}_g\right)_{2a},\ \left(\bar{A}_u\right)_{2a}, \tag{187}$$

for which:

$$\begin{aligned}\chi_{\left(\bar{A}_g\right)_{2a}}\left(\{E|0\}\right) &= \chi_{\left(\bar{A}_u\right)_{2a}}\left(\{E|0\}\right) = -\chi_{\left(\bar{A}_g\right)_{2a}}\left(\{\bar{E}|0\}\right) = -\chi_{\left(\bar{A}_u\right)_{2a}}\left(\{\bar{E}|0\}\right) = 1,\\ \chi_{\left(\bar{A}_g\right)_{2a}}\left(\{\mathcal{I}|0\}\right) &= -\chi_{\left(\bar{A}_g\right)_{2a}}\left(\{\bar{E}\mathcal{I}|0\}\right) = 1,\ \chi_{\left(\bar{A}_u\right)_{2a}}\left(\{\mathcal{I}|0\}\right) = -\chi_{\left(\bar{A}_u\right)_{2a}}\left(\{\bar{E}\mathcal{I}|0\}\right) = -1,\end{aligned} \tag{188}$$

implying that the lowest-angular-momentum spinful magnetic atomic orbitals (see SN 18) that transform in $\left(\bar{A}_g\right)_{2a}$

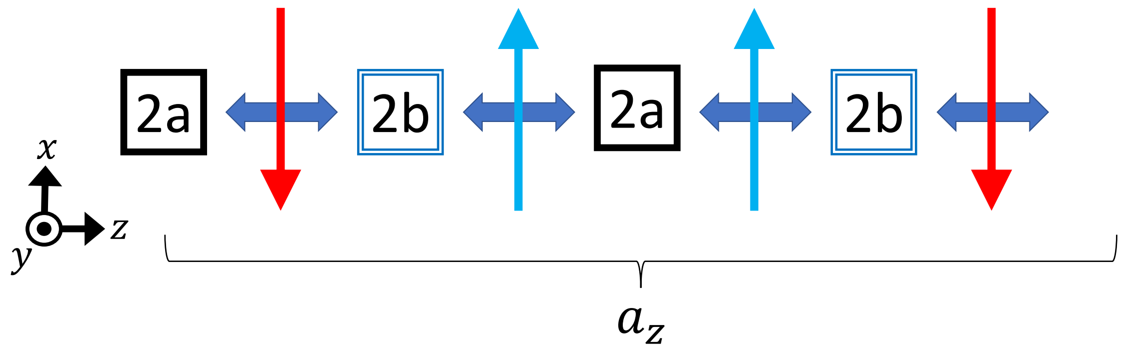

Supplementary Figure 17: An antiferromagnetic chain with magnetic rod group (MRG) $(p_c\bar{1})_{RG}$, which is generated by $\{\mathcal{I}|0\}$ and $\{\mathcal{T}|1/2\}$ $(t_{a_z/2}\mathcal{T})$ and is isomorphic after the addition of perpendicular lattice translations to Type-IV MSG 2.7 $P_S\bar{1}$ [see SRefs. 10,33,69–71 and the text following SEq. (2)]. There are three Wyckoff positions in MRG $(p_c\bar{1})_{RG}$ – $2a$, $2b$, and $4c$ – of which only $2a$ and $2b$ are maximal [SEq. (185)]. The site-symmetry group G_{2a} of sites in the maximal $2a$ position contains $\{\mathcal{I}|0\}$ [SEq. (186)], whereas the site-symmetry group G_{2b} of sites in the maximal $2b$ position instead contains $\{\mathcal{I}\times\mathcal{T}|1/2\}$ [SEq. (189)]; the site-symmetry group G_{4c} of sites in the general $4c$ position does not contain either $\{\mathcal{I}|0\}$ or $\{\mathcal{I}\times\mathcal{T}|1/2\}$ [SEq. (193)]. Four $\{\mathcal{I}\times\mathcal{T}|1/2\}$-related spinful s orbitals occupying the $2b$ position in $G = (p_c\bar{1})_{RG}$ divide into two pairs that each transform in the two-dimensional irreducible double-valued corep $\left(\bar{A}\bar{A}\right)_{2b}$ of G_{2b} [SEq. (191)], which is a necessary – but crucially not sufficient – condition for the four-dimensional band corep $\left(\bar{A}\bar{A}\right)^G_{2b} = \left(\bar{A}\bar{A}\right)_{2b}\uparrow G$ to be an EBR [see SEq. (182) and the surrounding text]. Indeed, in MRG $(p_c\bar{1})_{RG}$, we find that the four spinful s orbitals at $2b$ can be moved through the $4c$ position to $2a$ without breaking a symmetry or closing a gap. When the four s orbitals are moved to $2a$, the four orbitals form two pairs of spinful bonding and antibonding orbitals that each transform in the two-dimensional *reducible* corep $\left(\bar{A}_g\right)_{2a}\oplus\left(\bar{A}_u\right)_{2a}$ of G_{2a} [SEq. (191)], and induce a four-dimensional *composite* band corep $\left(\bar{A}_g\right)^G_{2a}\oplus\left(\bar{A}_u\right)^G_{2a}$. Because $\left(\bar{A}_g\right)^G_{2a}\oplus\left(\bar{A}_u\right)^G_{2a} = \left(\bar{A}\right)^G_{4c} = \left(\bar{A}\bar{A}\right)^G_{2b}$ [SEqs. (198) and (200)], then we conclude that $\left(\bar{A}\bar{A}\right)^G_{2b}$ is an *exceptional* case of a composite band corep induced from an irreducible corep of a site-symmetry group in a maximal Wyckoff position.

and $\left(\bar{A}_u\right)_{2a}$ are spin-split (singly-degenerate) s and p orbitals, respectively. We next examine the site-symmetry groups of the $\mathbf{q}_{2b}$ maximal Wyckoff position in SEq. (185) and SFig. 17, which are isomorphic to Type-III double MPG 2.3.5 $\bar{1}'$. G_{2b} also contains four symmetry operations:

$$G_{2b} = \left\{\{E|0\},\ \{\mathcal{I}\times\mathcal{T}|1/2\},\ \{\bar{E}|0\},\ \{\bar{E}\mathcal{I}\times\mathcal{T}|1/2\}\right\}, \tag{189}$$

in which only $\{E|0\}$ and $\{\bar{E}|0\}$ are unitary. Hence the maximal unitary subgroup H_{2b} of G_{2b} is given by:

$$H_{2b} = \left\{\{E|0\},\ \{\bar{E}|0\}\right\}, \tag{190}$$

such that H_{2b} is isomorphic to the trivial MPG [Type-I MPG 1.1.1 1, see the text following SEq. (27)]. As discussed in SRef. 33, $(\mathcal{I}\times\mathcal{T})^2 = \bar{E}$ in double SPGs, and $\chi_{\tilde{\rho}}(\{\bar{E}|0\}) = -\chi_{\tilde{\rho}}(\{E|0\})$ for double-valued coreps $\tilde{\rho}$. From this, in agreement with the output of the CorepresentationsPG tool on the BCS for Type-III double MPG 2.3.5 $\bar{1}'$, we determine that G_{2b} has only one, two-dimensional, double-valued irreducible corep [see SEq. (147) and the surrounding text]:

$$\tilde{\rho}_{2b} = \left(\bar{A}\bar{A}\right)_{2b}, \tag{191}$$

for which:

$$\chi_{\left(\bar{A}\bar{A}\right)_{2b}}\left(\{E|0\}\right) = -\chi_{\left(\bar{A}\bar{A}\right)_{2b}}\left(\{\bar{E}|0\}\right) = 2, \tag{192}$$

implying that the lowest-angular-momentum spinful magnetic atomic orbitals that transform in $\left(\bar{A}\bar{A}\right)_{2b}$ are an $\{\mathcal{I}\times\mathcal{T}|1/2\}$-related pair of spinful s orbitals, which are twofold-degenerate because $\chi_{\left(\bar{A}\bar{A}\right)_{2b}}([\{\mathcal{I}\times\mathcal{T}|1/2\}]^2) = -\chi_{\left(\bar{A}\bar{A}\right)_{2b}}(\{E|0\}) = -2$. Lastly, the site-symmetry groups in the $\mathbf{q}_{4c}$ position in SEq. (185) and SFig. 17 are isomorphic to the trivial MPG [Type-I MPG 1.1.1 1, see the text following SEq. (27)], and are thus equal to their maximal unitary subgroups H_{4c}:

$$G_{4c} = H_{4c} = \left\{\{E|0\},\ \{\bar{E}|0\}\right\}. \tag{193}$$

There is only one, one-dimensional, double-valued irreducible corep of G_{4c}:

$$\tilde{\rho}_{4c} = \left(\bar{A}\right)_{4c}, \tag{194}$$

for which:

$$\chi_{\left(\bar{A}\right)_{4c}}\left(\{E|0\}\right) = -\chi_{\left(\bar{A}\right)_{4c}}\left(\{\bar{E}|0\}\right) = 1. \tag{195}$$

SEq. (195) implies that the lowest-angular-momentum spinful magnetic atomic orbital that transforms in $\left(\bar{A}\right)_{4c}$ is a spin-split (singly-degenerate) s orbital.

Next, to determine if any of the band coreps induced from the maximal $2a$ and $2b$ Wyckoff positions in SEq. (185) and SFig. 17 are exceptional cases (*i.e.* composite), we induce band coreps from the intermediate $4c$ position that is connected to $2a$ and $2b$ [SEq. (183) and the surrounding text]. First, we focus on band coreps induced from $4c$ through $2b$. Because G_{4c} is an index-2 subgroup of G_{2b} ($[G_{2b} : G_{4c}] = 2$, see SEqs. (10), (189), and (193)), and because G_{4c} and G_{2b} have isomorphic unitary subgroups $H_{4c} = H_{2b}$ [SEq. (190) and (193)], then:

$$\left(\bar{A}\right)_{4c}\uparrow G_{2b} = \left(\bar{A}\bar{A}\right)_{2b}. \tag{196}$$

where $\left(\bar{A}\bar{A}\right)_{2b}$ is the irreducible corep of G_{2b} [SEq. (191)]. SEq. (196) implies that, for:

$$G = (p_c\bar{1})_{RG}, \tag{197}$$

it is possible for $\left(\bar{A}\right)_{4c}^{G} = \left(\bar{A}\right)_{4c} \uparrow G$ to be an EBR, because:

$$\left(\bar{A}\right)_{4c}^{G} = \left(\bar{A}\right)_{4c} \uparrow G_{2b} \uparrow G = \left(\bar{A}\bar{A}\right)_{2b} \uparrow G = \left(\bar{A}\bar{A}\right)_{2b}^{G}, \tag{198}$$

such that $\left(\bar{A}\bar{A}\right)_{2b}^{G}$ is a band corep induced from an irreducible corep of a site-symmetry group in a maximal Wyckoff position [see SEq. (182) and the surrounding text].

However, to determine if $\left(\bar{A}\bar{A}\right)_{2b}^{G}$ is indeed an EBR, we must also calculate the band coreps induced from $4c$ through $2a$, which are equivalent to $\left(\bar{A}\bar{A}\right)_{2b}^{G}$ [SEq. (183)]. Because G_{4c} is an index-2 subgroup of G_{2a} ($[G_{2a} : G_{4c}] = 2$, see SEqs. (10), (186), and (193)), because $\{E|0\} \in G_{2a}$, $\{E|0\} \in G_{4c}$, and because $\{\mathcal{I}|0\} \in G_{2a}$, $\{\mathcal{I}|0\} \notin G_{4c}$, then:

$$\left(\bar{A}\right)_{4c} \uparrow G_{2a} = \left(\bar{A}_g\right)_{2a} \oplus \left(\bar{A}_u\right)_{2a}, \tag{199}$$

where $\left(\bar{A}_g\right)_{2a}$ and $\left(\bar{A}_u\right)_{2a}$ are the irreducible coreps of G_{2a} [SEq. (187)], implying that $\left(\bar{A}_g\right)_{2a} \oplus \left(\bar{A}_u\right)_{2a}$ is a *reducible* corep of G_{2a}. SEq. (199) indicates that $\left(\bar{A}\right)_{4c}^{G} = \left(\bar{A}\right)_{4c} \uparrow G$ is not an EBR, but is instead a composite band corep, because

$$\left(\bar{A}\right)_{4c}^{G} = \left(\bar{A}\right)_{4c} \uparrow G_{2a} \uparrow G = \left[\left(\bar{A}_g\right)_{2a} \oplus \left(\bar{A}_u\right)_{2a}\right] \uparrow G = \left(\bar{A}_g\right)_{2a}^{G} \oplus \left(\bar{A}_u\right)_{2a}^{G}. \tag{200}$$

Because $\left(\bar{A}_g\right)_{2a}^{G} \oplus \left(\bar{A}_u\right)_{2a}^{G} = \left(\bar{A}\right)_{4c}^{G} = \left(\bar{A}\bar{A}\right)_{2b}^{G}$ [SEqs. (198) and (200)], then we conclude that $\left(\bar{A}\bar{A}\right)_{2b}^{G}$ is an exceptional case of a composite band corep induced from an irreducible corep of a site-symmetry group in a maximal Wyckoff position.

We can gain physical intuition for why $\left(\bar{A}\bar{A}\right)_{2b}^{G}$ is an exceptional-case composite band corep from the orbitals and spins depicted in SFig. 17. We begin with two $\{\mathcal{I}\times\mathcal{T}|1/2\}$-related pairs of spin-up and spin-down s orbitals that occupy $2b$ (*i.e.* four total spinful s orbitals separated into $\{\mathcal{I}\times\mathcal{T}|1/2\}$-reversed pairs at each of the two sites in the $2b$ position), where each pair transforms in the two-dimensional irreducible site-symmetry corep $\left(\bar{A}\bar{A}\right)_{2b}$. We are then free to move the four orbitals to $2a$ without breaking a symmetry of $(p_c\bar{1})_{RG}$ or closing a gap to introduce additional Wannier orbitals (which, conversely, is required in the closely-related obstructed-atomic-limit Wannier-sliding transitions discussed in SRefs. 1,71,149). When the four spinful s orbitals reach $2a$, the four orbitals form two bonding and antibonding pairs that each transform in the two-dimensional reducible site-symmetry corep $\left(\bar{A}_g\right)_{2a} \oplus \left(\bar{A}_u\right)_{2a}$ of G_{2a} [SEq. (187)], which induces a four-dimensional composite band corep $\left(\bar{A}_g\right)_{2a}^{G} \oplus \left(\bar{A}_u\right)_{2a}^{G}$ of $(p_c\bar{1})_{RG}$, indicating that $\left(\bar{A}\bar{A}\right)_{2a}^{G}$ is an exceptional composite band corep.

In SN 37, we provide a complete enumeration of all of the exceptional cases in the 1,651 single and double SSGs. For the Type-I MSGs and Type-II SGs previously analyzed in TQC[1–6], the exceptional cases listed in SN 37 agree with the previous tabulations performed in SRefs. 1,4. As shown in the text following SEq. (182), any band (co)rep induced from an irreducible (co)rep of a site in a maximal Wyckoff position that is not listed in the tables in SN 37 is an EBR. Hence, by calculating all of the band (co)reps induced from the irreducible (co)reps of the site-symmetry groups of the maximal Wyckoff positions of the 1,651 single and double SSGs, and then subsequently excluding the exceptional cases listed in SN 37, we obtain the complete list of single- and double-valued EBRs of the SSGs, completing the theory of MTQC.

25. Statistics for the MEBRs and the MBANDREP Tool

In this section, we provide general statistics for the EBRs previously obtained in SN 24 [which include the MEBRs of the Type-I MSGs and PEBRs of the Type-II SSGs previously tabulated for TQC[1–6], as well as the MEBRs of the Type-III and Type-IV MSGs calculated for the present work]. We additionally detail in this section the MBANDREP tool on the BCS, which we have implemented for this work to access both the elementary and non-elementary band (co)reps of all 1,651 single and double SSGs.

To begin, in Supplementary Tables 8 and 9, we provide the number of elementary and composite band (co)reps of the 1,651 single and double SSGs, respectively. Supplementary Tables 8 and 9 include the number of *exceptional* cases [SN 24 and 37] in which an irreducible (co)rep of a site-symmetry group of a site in a maximal Wyckoff position does not induce an EBR. For the Type-I MSGs and Type-II SGs analyzed in TQC[1–6], the band (co)rep statistics in Supplementary Tables 8 and 9 agree with the calculations previously performed in SRefs. 1,4. In Supplementary Tables 8 and 9, we also list the number of EBRs that can be decomposed into disconnected branches [*i.e. decomposable*

or "split" EBRs with disconnected subgraphs, see SN 16 and SRefs. 1,3,5,6,87,109,136]. As shown in SRefs. 1,5,6, 87,109, at least one disconnected piece of each decomposable EBR is topologically nontrivial, either in a stable or fragile sense[136–148]. In SN 26, we will provide a complete enumeration of the symmetry-based indicators of stable band topology[22–27,29–31] in the 1,651 double SSGs, which can be used to diagnose the stable topological indices of the disconnected branches of the decomposable double-valued EBRs in Supplementary Table 9. Lastly, to provide complete statistics for all of the band (co)reps that can be induced by *any* set of magnetic atomic orbitals in any Wyckoff position in a magnetic crystal, we additionally list in Supplementary Tables 8 and 9 the number of composite band (co)reps that can be induced from the unique irreducible (co)reps of the site-symmetry groups of the *non-maximal* Wyckoff positions in SSGs of the same type. Specifically, we obtain the numbers listed in the "Unique Non-Maximal Band (Co)reps" columns in Supplementary Tables 8 and 9 by summing over the composite band (co)reps induced from each unique irreducible (co)rep of one site-symmetry group in each non-maximal Wyckoff position in each SSG of the same type.

Single SSG Type	Number of SSGs	Number of EBRs [Decomposable EBRs]	Exceptional Cases	Unique Non-Maximal Band (Co)reps
Type-I	230	3,383 [219]	40	1,931
Type-II	230	3,141 [156]	39	1,852
Type-III	674	7,492 [833]	151	5,279
Type-IV	517	6,190 [699]	130	4,501
Total	1,651	20,206 [1,907]	360	13,563

Supplementary Table 8: Single-valued band (co)reps of the 1,651 single SSGs. In order, the columns in this table list the type of the single SSG (SN 2), the number of single SSGs of each type, the total number of single-valued elementary band (co)reps [EBRs] of the SSGs of the same type [see the text surrounding SEq. (180)], the total number of exceptional composite single-valued band (co)reps of the SSGs of the same type (SN 24 and 37), and the total number of composite single-valued band (co)reps induced from unique irreducible (co)reps of the site-symmetry groups of the non-maximal Wyckoff positions in SSGs of the same type.

Double SSG Type	Number of SSGs	Number of EBRs [Decomposable EBRs]	Exceptional Cases	Unique Non-Maximal Band (Co)reps
Type-I	230	2,258 [355]	107	1,589
Type-II	230	1,616 [426]	0	1,001
Type-III	674	5,047 [662]	591	4,882
Type-IV	517	3,882 [639]	556	3,984
Total	1,651	12,803 [2,082]	1,254	11,456

Supplementary Table 9: Double-valued band (co)reps of the 1,651 double SSGs. In order, the columns in this table list the type of the double SSG (SN 2), the number of double SSGs of each type, the total number of double-valued EBRs of the SSGs of the same type [see the text surrounding SEq. (180)], the total number of exceptional composite double-valued band (co)reps of the SSGs of the same type (SN 24 and 37), and the total number of composite double-valued band (co)reps induced from unique irreducible (co)reps of the site-symmetry groups of the non-maximal Wyckoff positions in SSGs of the same type.

Next, in SN 38, we provide tables of the minimum and maximum EBR dimension in each single and double SSG. In particular, the minimum EBR dimensions in the double SSGs in SN 38 provide an upper bound on the *minimal*

insulating filling of each double SSG[65,66,73,74,101,103,150,151], which is defined as the set of electronic fillings at which a short-range-entangled insulating phase is permitted for arbitrarily strong, SSG-symmetry-preserving interactions, analogous to the Lieb-Schultz-Mattis filling constraints for a 1D spin chain[152]. In the cases in which the minimum-dimension EBRs in an SSG are decomposable, a tighter bound on the minimal insulating filling can be further obtained by determining the minimum disconnected branch dimension of each decomposable EBR[87,151]. Hence, the minimum double-valued EBR dimensions of the Type-III and Type-IV MSGs listed in SN 38 provide upper bounds on the minimal electronic fillings at which short-range-entangled magnetic insulating phases are permitted in each Type-III and Type-IV MSG – at fillings that violate these bounds, any gapped, MSG-symmetric insulator must therefore exhibit long-range-entangled, magnetic topological order. Due to complications arising from the antiunitary symmetries of Type-III and Type-IV MSGs (see SN 5 and 6, respectively), the search for $\mathcal{T}$-breaking, long-range-entangled MSG-symmetric, insulating topological phases has thus far only been addressed from the perspective of minimal insulating filling in a handful of recent works[65,66,153]. For each single and double SSG, we have specifically confirmed that the minimum EBR dimension listed in SN 38 is consistent with the minimum atomic insulator dimension previously calculated in SRef. 65. In summary, the Type-III and Type-IV MEBRs computed in this work provide new information

Band co-representations of the Magnetic Double Space Group *P*6/*m*'*m*'*m*' (No. 191.241) and Wyckoff position 2d:(1/3,2/3,1/2)

Unitary subgroup: *P*622 (No. 177) in its standard setting.

Magnetic point group isomorphic to the site-symmetry group of the Wyckoff position: $\bar{6}$'*m*'2
and unitary subgroup: 32

The second column gives the coordinates of the k-vectors in the standard setting of the unitary subgroup.

Minimal set of paths and compatibility relations to analyse the connectivity

Show all types of k-vectors

Band-rep.		$A_1\uparrow G(2)$	$A_2\uparrow G(2)$	$E\uparrow G(4)$	$^1\bar{E}\uparrow G(2)$	$^2\bar{E}\uparrow G(2)$	$\bar{E}_1\uparrow G(4)$
Band-type		elementary	elementary	elementary	elementary	elementary	elementary
Decomposable \Indecomposable		Indecomposable	Indecomposable	Decomposable	Indecomposable	Indecomposable	Decomposable
Γ:(0,0,0)	Γ:(0,0,0)	$\Gamma_1(1) \oplus \Gamma_4(1)$	$\Gamma_2(1) \oplus \Gamma_3(1)$	$\Gamma_5(2) \oplus \Gamma_6(2)$	$\bar{\Gamma}_7(2)$	$\bar{\Gamma}_7(2)$	$\bar{\Gamma}_8(2) \oplus \bar{\Gamma}_9(2)$
A:(0,0,1/2)	A:(0,0,1/2)	$A_2(1) \oplus A_3(1)$	$A_1(1) \oplus A_4(1)$	$A_5(2) \oplus A_6(2)$	$\bar{A}_7(2)$	$\bar{A}_7(2)$	$\bar{A}_8(2) \oplus \bar{A}_9(2)$
H:(1/3,1/3,1/2)	H:(1/3,1/3,1/2)	$H_3(2)$	$H_3(2)$	$H_1(1) \oplus H_2(1) \oplus H_3(2)$	$\bar{H}_6(2)$	$\bar{H}_6(2)$	$\bar{H}_4\bar{H}_5(2) \oplus \bar{H}_6(2)$
K:(1/3,1/3,0)	K:(1/3,1/3,0)	$K_3(2)$	$K_3(2)$	$K_1(1) \oplus K_2(1) \oplus K_3(2)$	$\bar{K}_6(2)$	$\bar{K}_6(2)$	$\bar{K}_4\bar{K}_5(2) \oplus \bar{K}_6(2)$
L:(1/2,0,1/2)	L:(1/2,0,1/2)	$L_2(1) \oplus L_3(1)$	$L_1(1) \oplus L_4(1)$	$L_1(1) \oplus L_2(1) \oplus L_3(1) \oplus L_4(1)$	$\bar{L}_5(2)$	$\bar{L}_5(2)$	$2\ \bar{L}_5(2)$
M:(1/2,0,0)	M:(1/2,0,0)	$M_1(1) \oplus M_4(1)$	$M_2(1) \oplus M_3(1)$	$M_1(1) \oplus M_2(1) \oplus M_3(1) \oplus M_4(1)$	$\bar{M}_5(2)$	$\bar{M}_5(2)$	$2\ \bar{M}_5(2)$

Supplementary Figure 18: The output of the MBANDREP tool for the $2d$ Wyckoff position in Type-III MSG 191.241 $P6/m'm'm'$. Similar to the earlier BANDREP tool implemented for TQC[1–6], MBANDREP allows users to choose between the EBRs of each SSG and the band (co)reps induced from each Wyckoff position in the SSG. When the Wyckoff position option is selected in MBANDREP, users can additionally select non-maximal Wyckoff positions to access the unique composite band (co)reps discussed in Supplementary Tables 8 and 9 and the surrounding text [though we have only shown the output of MBANDREP for a maximal Wyckoff position in this figure]. Specifically, to generate this figure, we have selected the Wyckoff position option in MBANDREP for the $2d$ position in Type-III MSG 191.241 $P6/m'm'm'$. For each irreducible (co)rep $\tilde{\rho}_{\mathbf{q}}$ of one site-symmetry group $G_{\mathbf{q}}$ in each Wyckoff position in each SSG, MBANDREP outputs whether the induced band (co)rep $\tilde{\rho}_{\mathbf{q}}^G = \tilde{\rho}_{\mathbf{q}} \uparrow G$ is elementary, indicates whether $\tilde{\rho}_{\mathbf{q}}^G$ is decomposable[1,3,5,6,87,109,136], and lists the subduced small (co)reps in $\tilde{\sigma}_{\mathbf{k},\mathbf{q}}^G \downarrow G_{\mathbf{k}}$ [SEq. (170)] for each maximal $\mathbf{k}$ vector [SEq. (59) and the surrounding text] in the notation of the Corepresentations tool introduced in this work [see SN 13]. If an EBR is decomposable, users may click on the “Decomposable” button in MBANDREP to access a list of the allowed decompositions [branches] of the band (co)rep.

– including small (co)rep characters [SN 13] and compatibility relations [SN 16] – applicable to the search for novel long-range-entangled topological phases with magnetic crystal symmetries.

Finally, for this work, we have implemented the MBANDREP tool on the BCS to access both the elementary and non-elementary band (co)reps of all the 1,651 single and double SSGs. MBANDREP thus subsumes the earlier BANDREP tool (https://www.cryst.ehu.es/cgi-bin/cryst/programs/bandrep.pl)[1,2], which was previously implemented for TQC[1–6] to access the band (co)reps of the Type-I and Type-II SSGs. Unlike the earlier BANDREP tool, MBANDREP does not provide separate options for accessing band (co)reps with and without $\mathcal{T}$ symmetry, which are instead separately listed in MBANDREP under Type-II and Type-I SSGs, respectively [see SN 4 and 3, respectively]. In SFig. 18, we reproduce the output of MBANDREP for the $2d$ Wyckoff position in Type-III MSG 191.241 $P6/m'm'm'$.

26. Introduction to Symmetry-Indicated Magnetic Topological Bands

In the previous sections of this supplement, we established the theory of MTQC. The building blocks of MTQC are topologically trivial bands that transform in direct sums of EBRs [defined in the text surrounding SEq. (180)], and, consequently, can be inverse-Fourier-transformed into (magnetic) Wannier orbitals in position space [see SN 18]. Generically, however, energetically isolated bands [specifically, bands that satisfy the insulating compatibility relations along all high-symmetry BZ lines and planes, see SN 16] are not required to be equivalent [defined in the text following SEq. (180)] to integer-valued linear combinations of EBRs. As we will show in this section, if a band B is not equivalent to an integer linear combination of EBRs, then B either corresponds to a topological semimetal whose nodal points lie away from the high-symmetry BZ lines and planes (along which bands satisfy the insulating compatibility relations)[29,154], or is the Fourier-transformed description of a stable topological insulator or topological crystalline insulator (TI or TCI, respectively)[25,28,32,38,41,118,155–159].

When unitary crystal symmetries – such as spatial inversion ($\mathcal{I}$) or fourfold rotoinversion ($C_4 \times \mathcal{I}$) – are present in the SSG of the 3D bulk, then the stable topology of a set of energetically-isolated bands (along all high-symmetry BZ lines and planes) may be diagnosed using symmetry eigenvalues through a *symmetry-based indicator (SI) formula*. By exhaustion, it has been demonstrated[24–28,32,34–36,39,41,112,123,160–164] that $\mathcal{T}$-symmetric, symmetry-indicated, stable 3D TIs and TCIs necessarily exhibit anomalous 2D surface and 1D hinge states crossing the bulk gap, where the surface and hinge states are respectively protected by the symmetries of Type-II surface wallpaper groups and hinge frieze or line groups[73,75,165,166]. The quintessential SI formula in 3D is the Fu-Kane parity ($\mathcal{I}$) criterion for diagnosing 3D $\mathcal{T}$-symmetric TIs[32]. More recently, it was shown in SRefs. 22–31 that the compatibility relations and EBRs in an SSG can be used to generate a set of linearly independent SI formulas for stable topological bands that respect the symmetries of the SSG. The procedure introduced in SRefs. 23–25 returns the SI group (*e.g.* $\mathbb{Z}_4 \times \mathbb{Z}_2^3$) as well as the SI formula for the SSG in an arbitrary basis. Previously, in SRef. 65, the authors derived the SI groups of all 1,651 single and double SSGs, but not the SI formulas or the physical interpretation (*i.e.* the bulk topology and anomalous boundary states) of the magnetic bands with nontrivial SIs.

In the sections below, restricting consideration to the double-valued (co)reps of the 1,651 double SSGs, which characterize spinful electronic states in solid-state materials[33], we will go beyond the analysis in SRef. 65 and generate the SI formulas in a consistent and physically-motivated basis. In the *physical* SI formula basis introduced in this work, all previously identified nonmagnetic double SI formulas correspond to established nonmagnetic semimetallic, TI, and TCI phases. Additionally, in the physical SI formula basis, the SIs of symmetry-indicated TIs and TCIs with the same bulk topology (*e.g.* 3D TIs and AXIs with the common nontrivial axion angle $\theta = \pi$) are related by intuitive SI subduction relations. We will also introduce layer constructions[26,167,168] in the minimal double SSGs (defined in SN 31) for each TCI phase that admits a decomposition into layered 2D Chern insulators, TIs and mirror TCIs, which we will then use to determine symmetry-respecting bulk and anomalous surface and hinge states for all topological bands in the minimal double SSGs. First, in SN 27, we will review the method employed in SRefs. 23,26–28,35 in which the multiplicities of small (co)reps are used to determine the symmetry-indicated topology of energetically isolated bands. Next, in SN 28, we will introduce the Smith normal form[169] decomposition of the EBR matrix of an SSG G, through which one can infer the SIs in G. Then, in SN 30, we will detail a procedure for obtaining a set of minimal SIs on which the SIs in all 1,651 double SSGs are dependent. In the following section – SN 31 – we will then compute the minimal SI formulas for spinful topological phases in the 34 minimal double SSGs containing the minimal SIs in the self-consistent, physical basis described above. In SN 31, we will also formulate layer constructions – where possible – for the symmetry-indicated TI and TCI phases in the minimal double SSGs. We have confirmed that the spinful SI groups obtained in this work agree with the previous tabulation of magnetic and nonmagnetic SI groups in the 1,651 double SSGs performed in SRef. 65. The results of the calculations that we will perform in SN 31 will be summarized in SN 32. Lastly, in SN 33, we will further detail the helical (*i.e.* non-axionic) magnetic higher-order TCI (HOTI) phases[24–28,34–39] discovered in this work through the SI calculations performed in SN 31.

For the spinful helical magnetic HOTI phases discovered in this work, we will specifically detail symmetry-enhanced fermion doubling theorems[36,39,74,111] in SN 34, and will provide tight-binding models in SN 35.

27. Diagnosing Band Topology from Symmetry Eigenvalues

In this section, we will review the procedure by which a symmetry data vector B [see SRefs. 109,110 and the text following SEq. (108)] derived from the band structure of a material or model can be evaluated for nontrivial topology. The discussion in this section is largely a review of previous works on stable[22,24,26,27,29–31,109,110,170,171] and fragile[5,6,136–141,143–148] topology. To begin, in a given SSG G, if a set of bands is energetically isolated from all of the other bands in the spectrum at all high-symmetry $\mathbf{k}$ points and along all high-symmetry BZ lines and planes, then we may extract the symmetry data $B_{\mathbf{k}}$ at each point $\mathbf{k}$. As discussed in SN 16, the symmetry data $B_{\mathbf{k}}$ is composed of the multiplicities of the irreducible small (co)reps of the little group $G_{\mathbf{k}}$ that correspond to the set of energetically isolated Bloch eigenstates at $\mathbf{k}$ [see the text following SEq. (169)]. Given symmetry data $B_{\mathbf{k}}$ at a point $\mathbf{k}$, the symmetry data $B_{\mathbf{k}'}$ at a point $\mathbf{k}'$ that is connected to $\mathbf{k}$ [defined in the text following SEq. (58)] is fully determined by $B_{\mathbf{k}}$ through the compatibility relations $m^{\mathbf{k},\mathbf{k}'}$ [SEq. (110)] if the bands that transform in the symmetry data vector B are energetically isolated at all high-symmetry $\mathbf{k}$ points and along all high-symmetry BZ lines and planes. Hence, we may summarize the complete set of $B_{\mathbf{k}}$ in B with the symmetry data at a smaller number of $\mathbf{k}$ vectors consisting of one point $\mathbf{k}$ within each of the maximal momentum stars in G [defined in the text surrounding SEq. (59)]:

$$B = (m(\tilde{\sigma}_{1,\mathbf{k}_1}), m(\tilde{\sigma}_{2,\mathbf{k}_1}), \cdots, m(\tilde{\sigma}_{1,\mathbf{k}_2}), m(\tilde{\sigma}_{2,\mathbf{k}_2}), \cdots)^T, \tag{201}$$

where $m(\tilde{\sigma}_{l,\mathbf{k}_n})$ denotes the multiplicity of the lth small (co)rep of $G_{\mathbf{k}_n}$, and where B contains N_B entries. The multiplicities $m(\tilde{\sigma}_{l,\mathbf{k}_n})$ in B must obey a set of linear constraints imposed by the compatibility relations $\mathcal{CR}$, such that:

$$\mathcal{CR}\cdot B = 0, \tag{202}$$

in which each row of $\mathcal{CR}$ provides a linear constraint on B, and where the entries in $\mathcal{CR}$ are given by $m^{\mathbf{k},\mathbf{k}'}(m^{\mathbf{k}'',\mathbf{k}'})^{-1}$ taken over all pairs $\mathbf{k}$ and $\mathbf{k}''$ of maximal $\mathbf{k}$ vectors in G and all symmetry-unrelated $\mathbf{k}$ vectors $\mathbf{k}'$ that are mutually connected [defined in the text following SEq. (58)] to $\mathbf{k}$ and $\mathbf{k}''$. We emphasize that $(m^{\mathbf{k}'',\mathbf{k}'})^{-1}$, like $(c^{\mathbf{k}'})^{-1}$ in SEq. (119), is guaranteed to exist (though not necessarily be unique) through Frobenius reciprocity[2,108], because the elements of $m^{\mathbf{k}'',\mathbf{k}'}$ are defined through subduction in SEq. (109) [see the text surrounding SEqs. (110) and (117)].

In particular, the symmetry data of an EBR contain the multiplicities of small coreps that are induced from site-symmetry coreps in position space [see the text surrounding SEq. (162)]. For each SSG G, we may define an *EBR matrix*:

$$\mathcal{EBR} = (B^{\tilde{\rho}_{1,\mathbf{q}_1}}, B^{\tilde{\rho}_{2,\mathbf{q}_1}}, \cdots B^{\tilde{\rho}_{1,\mathbf{q}_2}}, B^{\tilde{\rho}_{2,\mathbf{q}_2}}, \cdots), \tag{203}$$

in which each column $B^{\tilde{\rho}_{j,\mathbf{q}_i}}$ contains the symmetry data vector of the EBR of G induced from the jth (co)rep $\tilde{\rho}_{j,\mathbf{q}_i}$ of the site-symmetry group $G_{\mathbf{q}_i}$ in the maximal Wyckoff position indexed by $\mathbf{q}_i$ (see SN 9). In the SSG G, we define the number of EBRs as $N_{\mathcal{EBR}}$, such that $\mathcal{EBR}$ in SEq. (203) is an $N_B\times N_{\mathcal{EBR}}$-dimensional matrix. By definition, an EBR must correspond to a set of Bloch states that are energetically isolated at all high-symmetry $\mathbf{k}$ points and along all high-symmetry BZ lines and planes, such that each $B^{\tilde{\rho}_{j,\mathbf{q}_i}}$ in SEq. (203) satisfies the compatibility relations:

$$\mathcal{CR}\cdot\mathcal{EBR} = 0. \tag{204}$$

We find that, in each of the 1,651 single and double SSGs, the rank of $\mathcal{EBR}$ is always equal to the dimension of the kernel of $\mathcal{CR}$ over the rational numbers, implying that the columns of $\mathcal{EBR}$ are at least a complete – and are in general an overcomplete – basis set of the kernel of $\mathcal{CR}$.

Given a set of bands that is energetically isolated at all high-symmetry $\mathbf{k}$ points and along all high-symmetry BZ lines and planes, the symmetry data vector B of the bands can be expressed in terms of $\mathcal{EBR}$:

$$[B]_a = \sum_b [\mathcal{EBR}]_{ab} p_b(B) = [\mathcal{EBR}\cdot p(B)]_a, \tag{205}$$

in which $p(B)$ is a vector of EBR multiplicities:

$$p(B) = (p(\tilde{\rho}_{1,\mathbf{q}_1}), p(\tilde{\rho}_{2,\mathbf{q}_1}), \cdots p(\tilde{\rho}_{1,\mathbf{q}_2}), p(\tilde{\rho}_{2,\mathbf{q}_2}), \cdots)^T, \tag{206}$$

where $p(\tilde{\rho}_{j,\mathbf{q}_i})$ indicates the multiplicity of the EBR symmetry data vector $B^{\tilde{\rho}_{j,\mathbf{q}_i}}$ in B [see the text folowing SEq. (203)], and where each $p(\tilde{\rho}_{j,\mathbf{q}_i})$ is rational, but not necessarily integer-valued. For all possible symmetry data vectors B that satisfy the compatibility relations, a decomposition of the form of SEqs. (205) and (206) is always permitted, because the symmetry data of the EBRs spans the set of symmetry data vectors that satisfy the compatibility relations in each SSG [*i.e.* because $\mathcal{EBR}$ spans the kernel of $\mathcal{CR}$, see SEq. (204) and the surrounding text][3,4,24,26,109,110,170,171]. When $\text{rank}(\mathcal{EBR}) = N_{\mathcal{EBR}}$, the multiplicities $p(\tilde{\rho}_{j,\mathbf{q}_i})$ in SEq. (206) are unique; however, when $\text{rank}(\mathcal{EBR}) < N_{\mathcal{EBR}}$, then $p(B)$ is not unique.

As discussed in several previous works[3–6,22–26,29–31,109,110,136,137,139–141,143–148,170,171], the values of $p(\tilde{\rho}_{j,\mathbf{q}_i})$ can be used to infer the topology of the bands that transform in B. Specifically, given a symmetry data vector B that satisfies the compatibility relations, there are three possibilities for the components of $p(B)$ in SEq. (206):

1. In each of the possible $p(B)$-vector solutions to SEq. (205), at least one of the multiplicities $p(\tilde{\rho}_{j,\mathbf{q}_i})$ is not an integer (but is still rational)[24].

2. There exists at least one solution to SEq. (205) in which all of the multiplicities $p(\tilde{\rho}_{j,\mathbf{q}_i}) \in \mathbb{Z}$, though there do not exist solutions in which all of the multiplicities $p(\tilde{\rho}_{j,\mathbf{q}_i}) \in \{\mathbb{Z}^+, 0\}$; therefore, at least one $p(\tilde{\rho}_{j,\mathbf{q}_i})$ is negative in the solution in which $p(\tilde{\rho}_{j,\mathbf{q}_i}) \in \mathbb{Z}$ for all i and j.

3. There exists at least one solution to SEq. (205) in which all of the multiplicities $p(\tilde{\rho}_{j,\mathbf{q}_i}) \in \{\mathbb{Z}^+, 0\}$.

In case 3, B contains the same small (co)reps as a direct sum of EBRs, such that the bands that transform in B exhibit the same symmetry eigenvalues as a trivial insulator. We note that this does not exclude the possibility that the bands that transform in B exhibit non-symmetry-indicated topology[6,28,38,71,124,149]. In case 2, it is possible to add EBRs to the bands that transform in B until the direct sum of the bands that transform in B and the added EBRs realizes a set of bands with a symmetry data vector B' classified by case 3. Therefore, as shown in SRefs. 5,6,136–141,143–148, in case 2, the bands that transform in B exhibit symmetry-indicated *fragile* topology. In the nomenclature of SRefs. 109,110,172, the symmetry data vectors in cases 2 and 3 correspond to "linear combinations of EBRs" [LCEBR]. Finally, in case 1, there does not exist an integer-valued linear combination of EBRs that can be added to the bands that transform in B to produce a set of bands with integer-valued $p(\tilde{\rho}_{j,\mathbf{q}_i})$. Consequently, as shown in SRefs. 24,26,27,109,110,170–172, the bands that transform in B in case 1 are not Wannierizable, and either correspond to a topological semimetal that satisfies the compatibility relations[29], or to a symmetry-indicated *stable* TI or TCI with anomalous surface or hinge states.

28. Symmetry-Based Indicator (SI) Groups and Formulas from the Smith Normal Form

In this section, we will introduce the method employed in this work to calculate the SI groups and formulas for spinful stable topological phases in all 1,651 double SSGs. In SN 29, we will then as an example provide an explicit calculation of the SI groups and formulas for double-valued irreps in Type-I double MSG 3.1 $P2$. Variants of the method described in this section were previously introduced in SRefs. 22–31. We will leave the enumeration of the symmetry-indicated fragile bands in the 1,651 single and double SSGs for future works. To begin, if the entries of a matrix are integer-valued, then the matrix carries a unique Smith normal form[169]. Consequently, given an SSG G, the EBR matrix $\mathcal{EBR}$ [defined in SEq. (203)] – whose entries are the integer-valued multiplicities of induced small (co)reps – can be decomposed into the Smith normal form:

$$\mathcal{EBR} = L_{\mathcal{EBR}} \Lambda_{\mathcal{EBR}} R_{\mathcal{EBR}}, \tag{207}$$

where $L_{\mathcal{EBR}}$ is an $N_B \times N_B$-dimensional unimodular matrix with integer-valued entries, $R_{\mathcal{EBR}}$ is an $N_{\mathcal{EBR}} \times N_{\mathcal{EBR}}$-dimensional unimodular matrix, and $\Lambda_{\mathcal{EBR}}$ is an $N_B \times N_{\mathcal{EBR}}$-dimensional (*i.e.* generically non-square) matrix with integer-valued entries $[\Lambda_{\mathcal{EBR}}]_{ij}$ for which:

$$[\Lambda_{\mathcal{EBR}}]_{ij} = 0 \text{ if } i \neq j. \tag{208}$$

Consequently, $\Lambda_{\mathcal{EBR}}$ – which is *the Smith normal form* of $\mathcal{EBR}$ – generically appears as:

$$\Lambda_{\mathcal{EBR}} = \begin{pmatrix} \lambda_1 & \cdots & 0 & 0 & \cdots & 0 \\ \vdots & \ddots & \vdots & \vdots & \ddots & \vdots \\ 0 & \cdots & \lambda_r & 0 & \cdots & 0 \\ 0 & \cdots & 0 & 0 & \cdots & 0 \\ \vdots & \ddots & \vdots & \vdots & \ddots & \vdots \\ 0 & \cdots & 0 & 0 & \cdots & 0 \end{pmatrix}, \tag{209}$$

in which $1 \leq \lambda_1 \leq \lambda_2 \leq \cdots \lambda_r$ are positive integers and $r = \text{rank}(\mathcal{EBR})$. We note that, in contrast to $\Lambda_{\mathcal{EBR}}$, $L_{\mathcal{EBR}}$ and $R_{\mathcal{EBR}}$ in SEq. (207) are not unique. For example, given $\mathcal{EBR}$ and $\Lambda_{\mathcal{EBR}}$, for any choice of $L_{\mathcal{EBR}}$ and $R_{\mathcal{EBR}}$, $L'_{\mathcal{EBR}} = -L_{\mathcal{EBR}}$ and $R'_{\mathcal{EBR}} = -R_{\mathcal{EBR}}$ always also satisfy the decomposition in SEq. (207).

Next, we consider all possible bands that transform in the most general symmetry data vector B in G that satisfies the compatibility relations [SEq. (202)]:

$$B = (m(\tilde{\sigma}_{1,\mathbf{k}_1}), m(\tilde{\sigma}_{2,\mathbf{k}_1}), \cdots, m(\tilde{\sigma}_{1,\mathbf{k}_2}), m(\tilde{\sigma}_{2,\mathbf{k}_2}), \cdots)^T, \tag{210}$$

where $m(\tilde{\sigma}_{l,\mathbf{k}_n})$ denotes the multiplicity of the lth small (co)rep of the little group $G_{\mathbf{k}_n}$. Previously, in the text following SEq. (206), we described a procedure for diagnosing whether bands that satisfy the compatibility relations exhibit symmetry-indicated stable topology. In the following text, we will now additionally describe a method for *classifying* stable band topology, which we will accomplish by parameterizing the space of solutions to SEq. (205). First, we act on both sides of SEq. (205) with the left inverse $L^{-1}_{\mathcal{EBR}}$, which is guaranteed to exist, because $L_{\mathcal{EBR}}$ is an integer, unimodular matrix [see the text following SEq. (207)]:

$$L^{-1}_{\mathcal{EBR}} B = \Lambda_{\mathcal{EBR}} R_{\mathcal{EBR}} \cdot p(B). \tag{211}$$

Because only the first r rows of $\Lambda_{\mathcal{EBR}}$ are nonzero, then, in order for a solution $p(B)$ to exist in SEq. (211), the $(r+1)^{\text{th}}$ to the $N^{\text{th}}_{\mathcal{EBR}}$ rows of $L^{-1}_{\mathcal{EBR}} B$ must be zero. However, the $(r+1)^{\text{th}}$ to the $N^{\text{th}}_{\mathcal{EBR}}$ rows of $L^{-1}_{\mathcal{EBR}} B$ are guaranteed to be zero, because $\mathcal{EBR}$ spans the kernel of $\mathcal{CR}$ [defined in SEq. (202)], and because B satisfies the compatibility relations. Hence, we obtain a solution for $p(B)$ in SEq. (211).

For each nonzero λ_i in SEq. (209), we next construct an r-dimensional vector $y(B)$ by multiplying B by $L^{-1}_{\mathcal{EBR}}$ and the pseudoinverse of $\Lambda_{\mathcal{EBR}}$ [SEq. (209)]:

$$[y]_i(B) = \frac{1}{\lambda_i}[L^{-1}_{\mathcal{EBR}} \cdot B]_i = [R_{\mathcal{EBR}} \cdot p(B)]_i, \ i = 1 \cdots r, \tag{212}$$

in which the entries $[y]_i(B)$ are rational numbers. We then re-express B in terms of $y(B)$ using SEq. (212):

$$[B]_j = \sum_{i=1}^{r} [L_{\mathcal{EBR}}]_{ji} [y]_i(B) \lambda_i. \tag{213}$$

Because $L_{\mathcal{EBR}}$ is unimodular, then the correspondance between the components of B and $y(B)$ is one-to-one. Conversely, the correspondence between $y(B)$ and $p(B)$ is generically one-to-many. Specifically, given $y(B)$, the most general solution for $p(B)$ takes the form:

$$p(B) = R^{-1}_{\mathcal{EBR}} \cdot (y_1(B), y_2(B), \cdots, y_r(B), k_1, k_2, \cdots, k_{N_{\mathcal{EBR}}-r})^T, \tag{214}$$

in which k_i are rational-valued free parameters.

To diagnose the stable topology of bands whose symmetry data satisfy the compatibility relations in G, we therefore restrict focus to the first r components of $p(B)$. Because $R_{\mathcal{EBR}}$ is a unimodular matrix, then the components of $p(B)$ are integer-valued if and only if $y_i(B)$ and k_i are integer-valued for all i, which reduces to the requirement that the values of $y_i(B)$ are integer-valued, because the values of k_i are free parameters in SEq. (214). Finally, using the values of $y_i(B)$, we define:

$$z_i(B) = (y_i(B)\lambda_i) \bmod \lambda_i = [L^{-1}_{\mathcal{EBR}} \cdot B]_i \bmod \lambda_i, \ i = i_0 \cdots r, \tag{215}$$

in which we have defined i_0 to be the smallest value of i for which $\lambda_{i_0} > 1$, and where each $y_i(B)$ is integer-valued

if and only if $z_i(B) = 0$. When B is expressed in terms of the most general small (co)rep multiplicities that satisfy the compatibility relations [*i.e.* in the form of SEq. (210)], then the $z_i(B)$ – which are implicitly functions of the small (co)rep multiplicities $m(\tilde{\sigma}_{l,\mathbf{k}_n})$ – are known as the *SI formulas* of G[23,24]. Correspondingly, the *representative* B_i *vector* for each i is defined as the ith column of $L_{\mathcal{EBR}}$ for which $z_i(B_i) = (L^{-1}_{\mathcal{EBR}} \cdot L_{\mathcal{EBR}})_{ii} \bmod \lambda_i = 1$.

Next, given a specific symmetry data vector B' with fixed values of $m(\tilde{\sigma}_{l,\mathbf{k}_n})$ that satisfy the compatibility relations, we may calculate the values $z_i(B')$, which necessarily satisfy $\{z_i(B') \in \mathbb{Z} | 0 \leq z_i(B') \leq \lambda_i - 1\}$. Hence, given B', the appearance of nonzero $z_i(B')$ in SEq. (215) implies that the components of $y(B')$ and $p(B')$ are not integer-valued, and that the bands that transform in B' exhibit stable topology. From this, we define the *SI vector of* B' as:

$$\mathbf{z}^G(B') = (z_{i_0}(B'), z_{i_0+1}(B'), \cdots, z_r(B'))^T, \tag{216}$$

where $z_i(B') \in \mathbb{Z}_{\lambda_i}$. Notably, the SI vectors of the representative B_i vectors satisfy $\mathbf{z}^G_j(B_i) = (L^{-1}_{\mathcal{EBR}} L_{\mathcal{EBR}})_{ji} \bmod \lambda_j = \delta_{ji} \bmod \lambda_j$. Lastly, using the values of λ_i obtained from SEqs. (209), (214), and (215), we define the *SI group* of G:

$$Z^G = \bigotimes_{i=i_0}^{r} \mathbb{Z}_{\lambda_i}. \tag{217}$$

Consequently, in G, the bands that transform in the representative B_i vectors may be summed with each other and with the EBRs of G to generate $|Z^G| - 1$ classes of stable topological bands that are not related by linear combinations of EBRs, as well as one class of (generically trivial) bands whose symmetry data vectors $\tilde{B}$ map to the trivial (identity) element of the SI group [$\mathbf{z}^G(\tilde{B}) = \mathbf{0}$ in SEqs. (215) and (216)]. Specifically, the SI group is spanned by summing the representative topological bands (*e.g.* $2B_i = B_i \oplus B_i$), such that $\mathbf{z}^G_i(nB_i) = n \bmod \lambda_i$ where $n \in \mathbb{Z}^+$. One stable topological band from each of the $|Z^G| - 1$ classes of stable topological bands and one integer-valued linear combination of EBRs that transforms in one $\tilde{B}$ vector together form a nonunique set of $|Z^G|$ bands that we designate in this work as the *SI topological bands.*

Using the method described in this section, we have obtained the SI formulas and groups for the double-valued (co)reps of all 1,651 double SSGs, which we term the *double SIs.* We have confirmed that the SI groups obtained in our calculations agree with the previous tabulation performed in SRef. 65. However, in general, both the SI formulas and the representative B_i vectors are computed in an arbitrary basis that is generically not the natural (physical) basis for classifying topological phases. Specifically, additional bulk- and boundary-state[25,27] or layer-construction[26] calculations must be performed to determine the semimetallic, TI, or TCI phases that correspond to each possible value of $z_i(B)$. Later, in SN 30, 39, and 31, we will determine a self-consistent, physically motivated basis and the corresponding bulk topology for the double SIs in all 1,651 double SSGs.

29. Double SI Group and Formulas in Type-I Double MSG 3.1 *P*2

As an example of the Smith normal form calculation described in SRefs. 22–31 and in the text following SEq. (207), we will in this section calculate the double SI group and formulas of Type-I double MSG 3.1 $P2$.

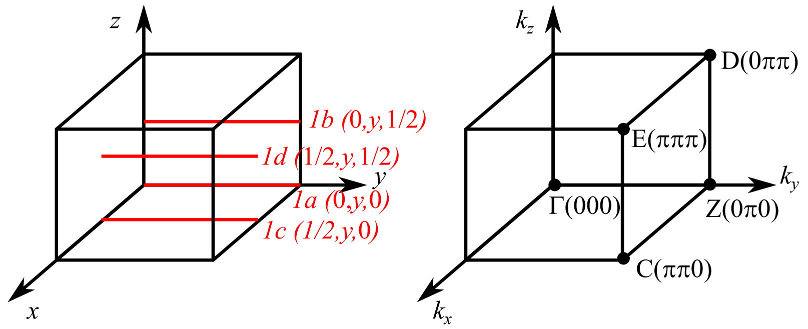

Supplementary Figure 19: The unit cell and BZ of Type-I MSG 3.1 $P2$. (Left panel) The unit cell of MSG 3.1 $P2$ with the maximal Wyckoff positions [SEq. (223)] labeled with red lines. (Right panel) The BZ of MSG 3.1 $P2$ with the maximal $\mathbf{k}$ vectors in SEq. (219), as well as the Γ point [$\mathbf{k}_\Gamma = (0, 0, 0)$], labeled with black circles.

First, using MGENPOS on the BCS[15–18], we determine that MSG 3.1 $P2$ is generated by:

$$\{C_{2y}|000\},\ \{E|100\},\ \{E|010\},\ \{E|001\}. \tag{218}$$

Next, using the MKVEC tool (see SN 12), we determine that there are four maximal momentum stars in $M = P2$ [defined in the text surrounding SEq. (59)]. Using MCOMPREL (see SN 16), we then find that, due to the compatibility relations, the small irrep multiplicities throughout the BZ of M are entirely determined by the irrep multiplicities at only one of the high-symmetry points in each of the four maximal momentum stars (SFig. 19):

$$\mathbf{k}_Z = 2\pi(0,1/2,0),\ \mathbf{k}_E = 2\pi(1/2,1/2,1/2),\ \mathbf{k}_D = 2\pi(0,1/2,1/2),\ \mathbf{k}_C = 2\pi(1/2,1/2,0). \tag{219}$$

At each of the four $\mathbf{k}$ points in SEq. (219), there are only two double-valued small irreps $\bar{\sigma}_{\mathbf{k}}^{\pm\frac{1}{2}}$ for which:

$$\chi_{\bar{\sigma}_{\mathbf{k}}^{\mp\frac{1}{2}}}(\{C_{2y}|\mathbf{0}\}) = \pm i. \tag{220}$$

In the notation of the Corepresentations tool on the BCS (SN 13):

$$\begin{aligned}\bar{\sigma}_{\mathbf{k}}^{\frac{1}{2}} &= \overline{Z}_3,\ \overline{E}_3,\ \overline{D}_3,\ \text{and}\ \overline{C}_3\ \text{for}\ \mathbf{k}=\mathbf{k}_Z,\ \mathbf{k}_E,\ \mathbf{k}_D,\ \text{and}\ \mathbf{k}_C,\ \text{respectively},\\ \bar{\sigma}_{\mathbf{k}}^{-\frac{1}{2}} &= \overline{Z}_4,\ \overline{E}_4,\ \overline{D}_4,\ \text{and}\ \overline{C}_4\ \text{for}\ \mathbf{k}=\mathbf{k}_Z,\ \mathbf{k}_E,\ \mathbf{k}_D,\ \text{and}\ \mathbf{k}_C,\ \text{respectively},\end{aligned} \tag{221}$$

such that the most general symmetry data vector B that satisfies the compatibility relations of M is given by:

$$B = (m(\overline{Z}_3), m(\overline{Z}_4), m(\overline{E}_3), m(\overline{E}_4), m(\overline{D}_3), m(\overline{D}_4), m(\overline{C}_3), m(\overline{C}_4))^T. \tag{222}$$

To calculate the Smith normal form of M described in the text surrounding SEq. (207), we next determine the symmetry data vectors of the EBRs of M. Using MWYCKPOS on the BCS[15–18], we find that M has four, multiplicity-1 maximal Wyckoff positions (defined in SN 9), which are indexed by the sites (SFig. 19):

$$\mathbf{q}_{1a} = (0,y,0),\ \mathbf{q}_{1b} = (0,y,1/2),\ \mathbf{q}_{1c} = (1/2,y,0),\ \mathbf{q}_{1d} = (1/2,y,1/2), \tag{223}$$

where $y \in [-1/2, 1/2)$, such that each of the sites in SEq. (223) lies along a line of $\{C_{2y}|\mathbf{0}\}$ symmetry (modulo integer lattice translations). At each of the four sites in SEq. (223), the site-symmetry group $G_{\mathbf{q}}$ is isomorphic to Type-I double MPG 3.1.6 2, which is generated by C_{2y}. Using the CorepresentationsPG tool (SN 18), we determine that each site-symmetry group $G_{\mathbf{q}}$ in M has two double valued irreps $(^1\overline{E})_{\mathbf{q}}$ and $(^2\overline{E})_{\mathbf{q}}$, where:

$$\chi_{(^1\overline{E})_{\mathbf{q}}}(C_{2y}) = i,\ \chi_{(^2\overline{E})_{\mathbf{q}}}(C_{2y}) = -i. \tag{224}$$

To obtain the symmetry data vectors of the EBRs of M, we use the MBANDREP tool introduced in this work [SN 23, see also SEqs. (162) and (173)], the output of which is reproduced below in the condensed notation of SRefs. 38,142:

$$\begin{aligned}
&(^2\overline{E})_{1a}\uparrow M = \overline{Z}_3\oplus\overline{E}_3\oplus\overline{D}_3\oplus\overline{C}_3, && (^1\overline{E})_{1a}\uparrow M = \overline{Z}_4\oplus\overline{E}_4\oplus\overline{D}_4\oplus\overline{C}_4,\\
&(^2\overline{E})_{1b}\uparrow M = \overline{Z}_3\oplus\overline{E}_4\oplus\overline{D}_4\oplus\overline{C}_3, && (^1\overline{E})_{1b}\uparrow M = \overline{Z}_4\oplus\overline{E}_3\oplus\overline{D}_3\oplus\overline{C}_4,\\
&(^2\overline{E})_{1c}\uparrow M = \overline{Z}_3\oplus\overline{E}_4\oplus\overline{D}_3\oplus\overline{C}_4, && (^1\overline{E})_{1c}\uparrow M = \overline{Z}_4\oplus\overline{E}_3\oplus\overline{D}_4\oplus\overline{C}_3,\\
&(^2\overline{E})_{1d}\uparrow M = \overline{Z}_3\oplus\overline{E}_3\oplus\overline{D}_4\oplus\overline{C}_4, && (^1\overline{E})_{1d}\uparrow M = \overline{Z}_4\oplus\overline{E}_4\oplus\overline{D}_3\oplus\overline{C}_3.
\end{aligned} \tag{225}$$

Using SEq. (225), we next construct the $\mathcal{EBR}$ matrix [SEq. (203)]:

$$\mathcal{EBR} = (B^{({}^2\overline{E})_{1a}}, B^{({}^1\overline{E})_{1a}}, B^{({}^2\overline{E})_{1b}}, B^{({}^1\overline{E})_{1b}}, B^{({}^2\overline{E})_{1c}}, B^{({}^1\overline{E})_{1c}}, B^{({}^2\overline{E})_{1d}}, B^{({}^1\overline{E})_{1d}}),$$

$$\mathcal{EBR} = \begin{pmatrix} 1 & 0 & 1 & 0 & 1 & 0 & 1 & 0 \\ 0 & 1 & 0 & 1 & 0 & 1 & 0 & 1 \\ 1 & 0 & 0 & 1 & 0 & 1 & 1 & 0 \\ 0 & 1 & 1 & 0 & 1 & 0 & 0 & 1 \\ 1 & 0 & 0 & 1 & 1 & 0 & 0 & 1 \\ 0 & 1 & 1 & 0 & 0 & 1 & 1 & 0 \\ 1 & 0 & 1 & 0 & 0 & 1 & 0 & 1 \\ 0 & 1 & 0 & 1 & 1 & 0 & 1 & 0 \end{pmatrix}, \tag{226}$$

in which the eight columns respectively correspond to the eight EBR symmetry data vectors of M given in the order of SEq. (225), and the eight rows respectively correspond to small irrep multiplicities given in the order of SEq. (222).

$\mathcal{EBR}$ in SEq. (226) admits a Smith normal decomposition [SEq. (207)]:

$$L_{\mathcal{EBR}} = \begin{pmatrix} 1 & -1 & 0 & -1 & -1 & 0 & 0 & 0 \\ 0 & 1 & 1 & 1 & 1 & 0 & 0 & 0 \\ 1 & 0 & 1 & -1 & 0 & 0 & 0 & 0 \\ 0 & 0 & 0 & 1 & 0 & 0 & 0 & 1 \\ 1 & -1 & 1 & 0 & 0 & 0 & 0 & 0 \\ 0 & 1 & 0 & 0 & 0 & 0 & 1 & 0 \\ 1 & 0 & 0 & 0 & 0 & 1 & 0 & 0 \\ 0 & 0 & 1 & 0 & 0 & 0 & 0 & 0 \end{pmatrix}, \Lambda_{\mathcal{EBR}} = \begin{pmatrix} 1 & 0 & 0 & 0 & 0 & 0 & 0 & 0 \\ 0 & 1 & 0 & 0 & 0 & 0 & 0 & 0 \\ 0 & 0 & 1 & 0 & 0 & 0 & 0 & 0 \\ 0 & 0 & 0 & 1 & 0 & 0 & 0 & 0 \\ 0 & 0 & 0 & 0 & 2 & 0 & 0 & 0 \\ 0 & 0 & 0 & 0 & 0 & 0 & 0 & 0 \\ 0 & 0 & 0 & 0 & 0 & 0 & 0 & 0 \\ 0 & 0 & 0 & 0 & 0 & 0 & 0 & 0 \end{pmatrix}, R_{\mathcal{EBR}} = \begin{pmatrix} 1 & 0 & 1 & 0 & 0 & 1 & 0 & 1 \\ 0 & 1 & 1 & 0 & 0 & 1 & 1 & 0 \\ 0 & 1 & 0 & 1 & 1 & 0 & 1 & 0 \\ 0 & 1 & 1 & 0 & 1 & 0 & 0 & 1 \\ 0 & -1 & -1 & 0 & -1 & 0 & -1 & 0 \\ 0 & 0 & 0 & 0 & 0 & 0 & 0 & 1 \\ 0 & 0 & 0 & 0 & 0 & 1 & 0 & 0 \\ 0 & 0 & 0 & 1 & 0 & 0 & 0 & 0 \end{pmatrix}, \tag{227}$$

in which the left inverse of $L_{\mathcal{EBR}}$ is given by:

$$L^{-1}_{\mathcal{EBR}} = \begin{pmatrix} 1 & 1 & 0 & 0 & 0 & 0 & 0 & -1 \\ 1 & 1 & 0 & 0 & -1 & 0 & 0 & 0 \\ 0 & 0 & 0 & 0 & 0 & 0 & 0 & 1 \\ 1 & 1 & -1 & 0 & 0 & 0 & 0 & 0 \\ -2 & -1 & 1 & 0 & 1 & 0 & 0 & -1 \\ -1 & -1 & 0 & 0 & 0 & 0 & 1 & 1 \\ -1 & -1 & 0 & 0 & 1 & 1 & 0 & 0 \\ -1 & -1 & 1 & 1 & 0 & 0 & 0 & 0 \end{pmatrix}. \tag{228}$$

As described in the text surrounding SEq. (215), we first examine the nonzero values in $\Lambda_{\mathcal{EBR}}$ to isolate the rows of $L^{-1}_{\mathcal{EBR}}$ that contain SI formulas for M. There is only a single entry $\lambda_i > 1$ in $\Lambda_{\mathcal{EBR}}$ in SEq. (227): $\lambda_5 = 2$. This implies that the double SI group of M [SEq. (217)] is:

$$Z^M = \mathbb{Z}_2, \tag{229}$$

and that the fifth row of $L^{-1}_{\mathcal{EBR}}$ contains the formula for a $\mathbb{Z}_2$-valued double SI:

$$z_{2R}(B) = -2m(\overline{Z}_3) - m(\overline{Z}_4) + m(\overline{E}_3) + m(\overline{D}_3) - m(\overline{C}_4) \bmod 2, \tag{230}$$

which can be re-expressed using the modulo 2 operation as:

$$z_{2R}(B) = m(\overline{Z}_4) + m(\overline{E}_3) + m(\overline{D}_3) + m(\overline{C}_4) \bmod 2. \tag{231}$$

Recognizing that $z_{2R}(B') = 0$ for any EBR symmetry data vector B', we next add the symmetry data vectors $({}^1\overline{E})_{1a} \uparrow M$ and $({}^2\overline{E})_{1b} \uparrow M$ from SEq. (225) to SEq. (231) to rotate $z_{2R}(B)$ into a more recognizable form:

$$z_{2R}(B) = m(\overline{Z}_3) + m(\overline{E}_3) + m(\overline{D}_3) + m(\overline{C}_3) \bmod 2. \tag{232}$$

Specifically, using the small irrep label substitution in SEq. (221), we recognize $z_{2R}(B)$ as the formula from SRefs. 156, 164 for the Chern number modulo 2 in the $k_y = \pi$ plane:

$$z_{2R} = \sum_{K=Z,E,D,C} n_K^{\frac{1}{2}} \bmod 2, \tag{233}$$

in which we have substituted $\mathbf{k} \to K$ for notational consistency with previous works[26,29]. Because $\{C_{2y}|\mathbf{0}\}$ is a symmetry of every BZ plane of constant k_y (see SFig. 19), then in an insulating phase, the compatibility relations require that the $\{C_{2y}|\mathbf{0}\}$ eigenvalues of the occupied bands along each of the $\{C_{2y}|\mathbf{0}\}$-invariant lines $k_{x,y} = 0, \pi$ are the same at each k_y. Hence $z_{2R} = 1$ implies that the $k_y = \pi$ and $k_y = 0$ planes *both* exhibit odd Chern numbers, such that the occupied bands either correspond to a 3D quantum anomalous Hall (QAH) insulator with an odd number of chiral modes per k_y on surfaces whose normal vectors point in the xz-plane, or to a Weyl semimetal with an even number of Weyl points between $k_y = 0, \pi$.

30. Minimal Double SIs in the 1,651 Double SSGs

Because there are 805 double SSGs G for which the double SI group $|Z^G| > 1$ (see Supplementary Table 10), then individually calculating the bulk and anomalous surface and hinge states and physical basis for each stable topological symmetry data vector in each SSG is a practically intractable task. However, in this section, we will detail a procedure for identifying a considerably smaller set of *minimal SSGs* with *minimal double SIs*, on which the double SIs in all 1,651 double SSGs are dependent. Specifically, by recognizing that the symmetry-indicated spinful topological semimetals, TIs, and TCIs in non-minimal double SSGs are indicated by the same bulk symmetries as spinful topological semimetals, TIs, and TCIs in the minimal double SSGs, we will reduce the calculation of the physical double-SI-formula bases and symmetry-respecting bulk and boundary states to a smaller, tractable problem.

Statistics of the Double SIs		
Type	SSGs with $\lvert Z^G\rvert > 1$	Minimal SSGs
Type-I	126	18
Type-II	117	5
Type-III	286	11
Type-IV	276	0
Total	805	34

Supplementary Table 10: Statistics for the double SIs of the 1,651 double SSGs. In order, each row of this table contains the type of the double SSG [see SN 2], the number of double SSGs with nontrivial double SI groups [$|Z^G| > 1$, see SEq. (217) and the surrounding text], and the number of minimal double SSGs with minimal double SIs.

To begin, consider a double SSG G and a subgroup M of G that is isomorphic to an SSG. Using the procedure detailed in SN 28, we then calculate the double SI groups $Z^{G,M}$, double SI formulas (in their original, arbitrary bases), and the symmetry data vectors B_i^G and B_j^M of the SI topological bands in G and M, respectively. We next restrict consideration to the case in which the double SI groups $Z^{G,M}$ are both nontrivial (*i.e.* $|Z^{G,M}| \neq 1|$). Lastly, we determine whether the SI topological bands in G subduce to inequivalent SI topological bands in M, in which case, we consider the double SIs in G to be *dependent* on the double SIs in M. Specifically, for an SSG G and a subgroup M of G that is isomorphic to an SSG (but not necessarily an SSG with the same Bravais lattice as G), the double SIs in G are dependent on the double SIs in M if and only if:

1. $|Z^G| \leq |Z^M|$.

2. For each SI topological band in G with a symmetry data vector B_i^G [defined in the text following SEq. (217)], the *subduced SI vector* $\mathbf{z}^M(B_i^G \downarrow M)$ [SEq. (216)] exhibits a distinct value for each choice of i. Specifically, given any two SI topological bands $B_{i_1}^G$ and $B_{i_2}^G$ in G for which $\mathbf{z}^G(B_{i_1}^G) \neq \mathbf{z}^G(B_{i_2}^G)$, the SIs in G can only be dependent on the SIs in M if $\mathbf{z}^M(B_{i_1}^G \downarrow M) \neq \mathbf{z}^M(B_{i_2}^G \downarrow M)$ for all choices of $B_{i_1}^G$ and $B_{i_2}^G$.

The above requirements indicate the conditions under which the double SIs in G are dependent on the double SIs in M. However, there may also exist subgroups M' of M where the double SIs in *both* G and M are dependent on the double SIs in M'. Hence, given an SSG M for which $|Z^M| > 1$, if there does not an exist a subgroup M' of M

for which the double SIs in M are dependent on the double SIs in M', then we define M as a *minimal double SSG*. Correspondingly, we define the *minimal double SIs* of the 1,651 double SSGs as the double SIs of the minimal double SSGs.

We note that in this work, we have employed a more narrow definition than in other previous works[26,29] for minimal SIs. Specifically, in SRefs. 26,29 the authors considered cases in which the SIs in G in are neither dependent on the SIs in the subgroups $M \subset G$ and $M' \subset G$ (where M is not isomorphic to M'), but where the SIs in G are still spanned by the *combined* SIs in M and M'. As we will show below, using our narrower definition of minimal SIs, we still obtain a manageable number of minimal double SSGs.

Next, given a minimal SSG M and an SSG G in which the SIs are dependent on the SIs in M, it follows that all of the symmetry-indicated stable topological semimetals, TIs, and TCIs in G are indicated by the same bulk symmetries that indicate the bulk topology in M. Specifically, this dependency occurs because the set of SI topological bands in G subduced onto M is spanned by the SI topological bands in M modulo EBRs of M, and because the EBRs of M do not exhibit topological bulk, surface, or hinge states[26,27,38], as they are Wannierizable[1–6].

Conversely, if the bulk bands of a symmetry-indicated TI or TCI in G are subduced onto an SSG M where the SIs in G are dependent on the SIs in M, the subduced topological insulating phase in M may exhibit different anomalous boundary states. For example, when symmetry-indicated 3D TIs – such as an insulator with $z_2 = 1$ in Type-II double SG 81.34 $P\bar{4}1'$ (see SRef. 26) – are subduced to magnetic axion insulator (AXI)[28,38,40–60] phases in minimal MSGs (in this case, Type-I double MSG 81.33 $P\bar{4}$, see SN 39), the twofold surface Dirac cones of the parent 3D TI become gapped on surfaces in which the Dirac cones are only protected by $\mathcal{T}$ symmetry (see SRefs. 34,36,38,123,173), revealing a symmetric-sample-spanning network of chiral hinge modes. More generally, given a TI or TCI that respects the symmetries in the bulk SSG G, the anomalous 2D surface states on a surface with a Miller index vector $\hat{\mathbf{n}}$ are necessarily protected by the symmetries of a wallpaper subgroup of G[73,75] that leaves $\hat{\mathbf{n}}$ invariant. However, when the occupied topological bands are subduced onto a subgroup $M \subset G$ where M is isomorphic to an SSG, it is not generically guaranteed that the 2D surface states on the $\hat{\mathbf{n}}$-normal surface are still gapless, because the $\hat{\mathbf{n}}$-normal surface only respects the symmetries of a wallpaper subgroup of M. Nevertheless, we find that a finite (0D) geometry can in many cases be chosen for a symmetry-indicated TI or TCI that respects the symmetries of a bulk SSG G such that, upon subducing the bulk bands onto a subgroup $M \subset G$, the boundary states do not become gapped. Importantly, 3D TIs that subduce to magnetic AXIs[32,41,160,174] represent a notable exception, because *all* 2D surfaces of 3D TIs exhibit odd numbers of twofold Dirac cones, whereas there do not exist magnetic AXIs in which all 2D surfaces are gapless[28,38,40–60].

Furthermore, we note that it is also possible for an SI topological band B_i^G in G to correspond to a gapless (semimetallic) phase even if a subduced SI topological band $B_i^G \downarrow M$ corresponds to a gapped (TI or TCI) phase. An example occurs in Type-IV double MSG 75.5 P_C4 and its minimal double subgroup Type-I MSG 75.1 $P4$. As we will show below in SN 31 e, all of the double SIs in $M = P4$ are compatible with rotation-symmetry-indicated QAH states. However, because:

$$G = P_C4 = P4 \cup \{\mathcal{T}|\mathbf{t}_c/2\}P4, \tag{234}$$

then G contains the antiunitary symmetry $\{C_{2z} \times \mathcal{T}|\mathbf{t}_c/2\}$, which enforces the presence of gapless (Weyl) points in the $k_z = 0, \pi$ planes for all nontrivial values of the SIs in G[26,36,175]. This can be seen by recognizing that $\{C_{2z} \times \mathcal{T}|\mathbf{t}_c/2\}$ symmetry can protect gapless points in 2D systems (*e.g.* high-symmetry BZ-planes), and that the Chern numbers of the occupied bands in $\{C_{2z} \times \mathcal{T}|\mathbf{t}_c/2\}$-invariant planes (*e.g.* $k_z = 0, \pi$) are required by symmetry to vanish. After the submission of this work, the authors of SRef. 176 performed a complete enumeration of the cases in which an SI topological band B_i^G in G corresponds to a gapless phase while the SI topological band $B_i^G \downarrow M$ in the subgroup $M \subset G$ is compatible with a gapped topological phase.

In this work, we have exhaustively calculated the double SI groups and formulas of all 1,651 double SSGs, and have determined that, remarkably, there are only 34 minimal double SSGs (see Supplementary Table 10):

1. Minimal Type-I Double MSGs (18 MSGs): 2.4 $P\bar{1}$, 3.1 $P2$, 10.42 $P2/m$, 47.249 $Pmmm$, 75.1 $P4$, 77.13 $P4_2$, 81.33 $P\bar{4}$, 83.43 $P4/m$, 84.51 $P4_2/m$, 88.81 $I4_1/a$, 123.339 $P4/mmm$, 143.1 $P3$, 147.13 $P\bar{3}$, 168.109 $P6$, 174.133 $P\bar{6}$, 175.137 $P6/m$, 176.143 $P6_3/m$, 191.233 $P6/mmm$.

2. Minimal Type-II Double SGs (5 SGs): 2.5 $P\bar{1}1'$, 83.44 $P4/m1'$, 87.76 $I4/m1'$ 175.138 $P6/m1'$ 176.144 $P6_3/m1'$.

3. Minimal Type-III Double MSGs (11 MSGs): 27.81 $Pc'c'2$, 41.215 $Ab'a'2$, 54.342 $Pc'c'a$, 56.369 $Pc'c'n$, 60.424 $Pb'cn'$, 83.45 $P4'/m$, 103.199 $P4c'c'$, 110.249 $I4_1c'd'$, 130.429 $P4/nc'c'$, 135.487 $P4_2'/mbc'$, 184.195 $P6c'c'$.

Interestingly, we observe that there are no minimal Type-IV double MSGs (see Supplementary Table 10). As discussed in the main text, this implies that symmetry-indicated spinful topological phases in Type-IV MSGs are actually enforced by the symmetries of lower-symmetry Type-I or Type-III double MSGs. For example, we find that

the inversion- ($\mathcal{I}$-) symmetric antiferromagnetic (AFM) TCIs introduced in SRef. 177, which respect the symmetries of Type-IV MSGs containing $\{\mathcal{I}|\mathbf{0}\}$, in fact subduce to $\mathcal{I}$-symmetric AXIs[28,38,40–60] in Type-I double MSG 2.4 $P\bar{1}$ (see SN 31 a for the double SI group and formulas of double MSG 2.4 $P\bar{1}$). Previously, in SRef. 26, the authors determined that the double SIs in all Type-II double SGs are dependent on the double SIs in one of six Type-II double SGs: 2.5 $P\bar{1}1'$, 81.34 $P\bar{4}1'$, 83.44 $P4/m1'$, 174.134 $P\bar{6}1'$, 175.138 $P6/m1'$, and 176.144 $P6_3/m1'$. However, in this work, we find that Type-II SGs 81.34 $P\bar{4}1'$ and 174.134 $P\bar{6}1'$ are no longer minimal double SSGs after including magnetic subgroups of Type-II SGs, because their double SIs are respectively dependent on the double SIs in Type-I double MSGs 83.33 $P\bar{4}$ and 174.133 $P\bar{6}$. Additionally, for the purposes of this work, we have included Type-II SG 87.76 I4/m1' in our list of minimal double SSGs, because its double SI formulas can only be spanned by subducing SI topological bands onto two different minimal double SSGs (Type-II SGs 2.5 $P\bar{1}1'$ and 83.44 $P4/m1'$), rather than one. In the Supplementary Table in SN 39, we provide a complete enumeration of the minimal double SSGs with the minimal double SIs on which the double SIs in each double SSGs are dependent.

31. Double SI Formulas for Spinful Bands with Stable Topology in the 34 Minimal Double SSGs

Previously, in SN 30, we determined that the SIs in each of the 1,651 double SSGs are fully dependent on the minimal double SIs in one of 34 minimal double SSGs (the minimal double SSG associated to each double SSG is listed in the Supplementary Table in SN 39). In this section, we will present the minimal double SIs in all 34 minimal double SSGs, and hence, the minimal double SIs of spinful band topology in all 1,651 double SSGs. We will additionally transform the double SI formulas into a unified basis – which we term a *physical* basis – in which the double SIs for previously established spinful topological semimetals (SMs), TIs, and TCIs[32,36,39,41,112,123,160–163,178–182] take the same form as the double SIs introduced in previous works[22–31,34,35,164]. In a physical basis, the SIs for topological phases with the same response theories [*e.g.* a $z_8 = 1$ 3D TI in Type-II SG 123.340 $P4/mmm1'$ and an $\eta_{4I} = 2$ magnetic AXI in MSG 2.4 $P\bar{1}$, see SRef. 26 and SN 31 a] are related through simple relations obtained from group-subgroup subduction [*e.g.* the relation $\eta_{4I} = 2(z_8 \bmod 2)$ introduced in this work].

Below, for each minimal double SSG G, we will list the SI group Z^G [SEq. (217)] and the SI formula(s). We will additionally formulate layer constructions for the gapped (TI and TCI) phases, where admitted (see SRefs. 26,168 for further discussions of cases in which TI and TCI phases do not admit layer constructions). For the symmetry-indicated 3D QAH phases that we identify in the 34 minimal double SSGs, the anomalous boundary states are chiral modes along surfaces perpendicular to the Chern-layer stacking direction[65,178,182]. We will show that the remaining 3D symmetry-indicated, spinful, gapped topological phases in the 34 minimal double SSGs are 3D TI and TCI phases – which we will show to consist of AXIs[28,38,40–60] with chiral hinge states, 3D TIs with twofold-degenerate, $\mathcal{T}$-symmetry-protected surface Dirac cones[32,41,160,174], helical mirror TCIs with mirror-protected surface states[161,162], and higher-order TCIs (HOTIs) with mirror- or $\mathcal{T}$-protected helical hinge states[24–28,34–39]. We emphasize that, employing the convention of SRefs. 34,39, a 2D crystal surface can only respect the symmetries of a wallpaper group, whereas a 1D hinge may either respect the symmetries of a frieze group or a line group [defined in SRefs. 73,75,165,166], depending on how the finite sample is cut from an infinite crystal. In this work, we define a helical (*i.e.* non-axionic) TCI phase to be higher-order topological if the TCI phase, when cut into a nanorod geometry, exhibits anomalous helical states that run along nanorod edges that are parallel to bulk rotation axes, where each edge is left invariant under a frieze or line group that contains either $\mathcal{T}$ symmetry or a mirror line parallel to the nanorod edge.

For each of the 34 minimal double SSGs, we will additionally identify the minimal layer constructions necessary to span the subset of SI topological bands [defined in the text following SEq. (217)] corresponding to gapped (TI and TCI) phases; however, as we will detail below, we find that some of the symmetry-indicated spinful TI and TCI phases in the 34 minimal double SSGs are not layer-constructable. Specifically, as demonstrated in SRef. 26, a large subset of the previously identified TI and TCI phases in each Type-II SG G can be modeled by placing decoupled, flat layers of Chern insulators, 2D TIs, and 2D TCIs in each unit cell of a crystal that respects the symmetries of G. In this work, we find that a subset of the AXI phases in the minimal double SSGs cannot be constructed from layers of 2D TIs and TCIs. We conjecture that the AXI phases without layer constructions can still be constructed using the "topological crystal" framework discussed in SRef. 168, which incorporates cell complexes of 2D TIs and TCIs.

Throughout this section, we will obtain the properties of each minimal double SSG using tools on the BCS. Specifically, we will obtain the generators for each minimal double SSG using the MGENPOS tool[15–18], the maximal Wyckoff positions using the MWYCKPOS tool[15–18], the maximal momentum stars using the MKVEC tool (see SN 12), the small (co)reps using the Corepresentations tool (see SN 13), and the EBRs using the MBANDREP tool (see SN 23).

In this work, we will provide each double SI formula in the notation of SN 29 and SRefs. 26,29. For centrosymmetric SSGs (*i.e.* SSGs that contain $\{\mathcal{I}|\mathbf{0}\}$ in at least one definition of the unit cell origin), we will use the symbols $n_K^{\pm}$ to

respectively indicate the number of Bloch eigenstates at the inversion-invariant point $\mathbf{k} = K$ with the parity ($\{\mathcal{I}|\mathbf{0}\}$) eigenvalues ± 1 in a given energy range (which is typically runs over the occupied bands). For SSGs that contain rotation symmetries of the form $\{C_n|\mathbf{0}\}$ or screw symmetries of the form $\{C_{nl}|\mathbf{t}_l/b\}$ in at least one definition of the unit cell origin, we will use the symbol n_K^j to indicate the number of Bloch eigenstates at the C_n-rotation(or screw)-invariant point $\mathbf{k} = K$ with the rotation eigenvalue $e^{-i\frac{2\pi}{n}j}$ in a given energy range. Because we are restricting focus in this work to the double SIs of spinful band topology in the 1,651 SSGs, the factor of j in each rotation eigenvalue $e^{-i\frac{2\pi}{n}j}$ is half-integer-valued; in this work, we term j the *angular momentum* (taken modulo n) of the rotation- or screw-invariant Bloch eigenstates at K. Next, for SSGs that contain fourfold rotoinversion symmetries of the form $\{S_4|\mathbf{0}\} = \{C_4 \times \mathcal{I}|\mathbf{0}\}$ (but not fourfold rotation symmetries of the form $\{C_4|\mathbf{0}\}$) in at least one definition of the unit cell origin, we will use the symbol n_K^j to indicate the number of Bloch eigenstates at the S_4-invariant point $\mathbf{k} = K$ with the $\{S_4|\mathbf{0}\}$ rotoinversion eigenvalues $e^{-i\frac{2\pi}{4}j}$ in a given energy range. Generically, n_K^j ($j = \pm\frac{1}{2}, \pm\frac{3}{2}$) is defined using $\{S_4|\mathbf{0}\}$ eigenvalues *only if* the point K is $\{S_4|\mathbf{0}\}$-invariant, but not $\{C_4|\mathbf{0}\}$-invariant. Conversely, if K is $\{C_4|\mathbf{0}\}$-invariant, then n_K^j ($j = \pm\frac{1}{2}, \pm\frac{3}{2}$) is always defined using the eigenvalues of $\{C_4|\mathbf{0}\}$. Lastly, for SSGs that contain both mirror symmetries of the form $\{m_l|\mathbf{0}\}$ and rotation symmetries of the form $\{C_{nl}|\mathbf{0}\}$ or screw symmetries of the form $\{C_{nl}|\mathbf{t}_l/b\}$ in at least one definition of the unit cell origin, we will use the symbols $n_K^{j,\pm i}$ to respectively indicate the number of Bloch eigenstates at the rotation- or screw-invariant point $\mathbf{k} = K$ with the rotation or screw eigenvalue $e^{-i\frac{2\pi}{n}j}$ and the mirror eigenvalue $\pm i$ in a given energy range.

Before we will derive the double SIs in the 34 minimal double SSGs, we will first summarize our labeling convention for double SIs. First, for double SIs that have the same SI formulas as the nonmagnetic double SIs introduced in SRefs. 26, we have followed the labeling convention established in SRef. 26:

1. $z_{2w,i}$ ($i = 1, 2, 3$) are the weak TI SIs in the $k_i = \pi$ planes, or the weak mirror Chern numbers modulo 2 in the $k_i = \pi$ planes in the absence of $\{\mathcal{T}|\mathbf{0}\}$ symmetry.

2. $z_{nm,k}$ ($n = 4, 3, 6$, $k = 0, \pi$) are the mirror Chern numbers (modulo n) in the $k_z = k$ plane indicated by rotation eigenvalues in SSGs 83.44 $P4/m1'$, 174.134 $P\bar{6}1'$, 175.138 $P6/m1'$ for $n = 4, 3, 6$, respectively. In this work, we will use the symbol $z^{\pm}_{nm,k}$ to represent the Chern numbers of sets of bands with mirror eigenvalues $\pm i$, respectively.

3. z_4, z_2, z_8, z_{12}, and z'_{12} indicate strong 3D TIs and helical TCIs and HOTIs in SSGs 2.5 $P\bar{1}1'$, 81.34 $P\bar{4}1'$, 83.44 $P4/m1'$, 175.138 $P6/m1'$, 176.144 $P6_3/m1'$, respectively. Odd values of z_4, z_2, z_8, z_{12}, and z'_{12} correspond to strong TIs. $z_4 = 2$, $z_8 = 4$, $z_{12} = 6$, $z'_{12} = 6$ correspond to non-axionic HOTI phases with helical hinge states or mirror TCIs with even mirror Chern numbers (see SN 31 s through 31 w and SRef. 26).

If the double SIs in a Type-II SSG G continue to indicate stable topological phases in a magnetic subgroup M of G, then we will use the same double SI labels and formulas in G and M.

We additionally find that there are Type-I and Type-III double MSGs with new double SIs that are not subduced from Type-II SSGs (see Supplementary Table 10). For these minimal *magnetic* double SIs, we have adopted a convention in which:

1. z_{nR} ($n = 2, 3, 4, 6$) represent Chern numbers (modulo n) indicated by rotation eigenvalues.

2. z'_{nR} and z''_{nR} ($n = 2, 3, 4, 6$) represent doubled Chern numbers indicated by rotation eigenvalues [*i.e.* $z'_{nR} = (C/2) \bmod n$] in nonsymmorphic MSGs.

3. η_{4I} is defined in MSG 2.4 $P\bar{1}$. Odd values of η_{4I} correspond to Weyl semimetals, and $\eta_{4I} = 2$ corresponds to an AXI provided that the net Chern numbers are zero and there are no Weyl points in the BZ interior. We use the symbol "η" rather than "z" to distinguish η_{4I} from the double SI z_4 in the minimal double SSGs 2.5 $P\bar{1}1'$, 47.249 $Pmmm$, and 83.45 $P4'/m$ and from the double SI z'_4 in double MSG 135.487 $P4'_2/mbc'$.

4. $z_{2I,i}$ ($i = 1, 2, 3$) are defined in double MSG 2.4 $P\bar{1}$, and respectively represent the Chern numbers modulo 2 in the $k_i = \pi$ planes indicated by $\mathcal{I}$ (parity) eigenvalues. We have used the subscript "I" to distinguish $z_{2I,i}$ from z_{2R} (the Chern number modulo 2 indicated by C_2 rotation eigenvalues) and $z_{2w,i}$ (the weak TI and TCI parity indices discussed above).

5. $\eta'_{2I} = \frac{1}{2}\eta_{4I}$ represents a doubled variant of η_{4I} that is present in SSGs in which symmetry requires η_{4I} to be even.

6. δ_{nm} ($n = 2, 3, 4, 6$) represent the differences between the mirror Chern numbers in the $k_z = 0, \pi$ planes (modulo n).

7. z_{4S} and δ_{2S} are defined in MSG 81.33 $P\bar{4}$. Respectively, z_{4S} and δ_{2S} represent the total Chern number (modulo 4) in the $k_z = \pi$ plane and twice the difference of the total Chern numbers in the $k_z = 0, \pi$ planes (*i.e.* $\delta_{2S} = [(C_{k_z=\pi} - C_{k_z=0})/2] \text{ mod } 2$).

8. z'_4 in MSG 135.487 $P4'_2/mbc'$ represents a different $\mathbb{Z}_4$-valued doubled variant of the double SI η_{4I} than the double SI z_4 discussed above [*e.g.* $\eta_{4I} = (2z'_4) \text{ mod } 4$].

9. For all of the symbols of the double SIs, the first number (n) in the subscript indicates that the corresponding double SI takes integer values in the range $[0, n-1]$.

a. Double SIs in Type-I Double MSG 2.4 $P\bar{1}$

The double MSG 2.4 $P\bar{1}$ is generated by $\{E|100\}$, $\{E|010\}$, $\{E|001\}$, and $\{\mathcal{I}|\mathbf{0}\}$. The SIs of MSG 2.4 $P\bar{1}$ were previously analyzed in SRefs. 65,183; the previous analyses performed in SRefs. 65,183 agree with the analysis performed in this section.

Double SIs – The double MSG 2.4 $P\bar{1}$ has the SI group $\mathbb{Z}_4 \times \mathbb{Z}_2^3$. We define the four SIs of double MSG 2.4 $P\bar{1}$ to be $(\eta_{4I}, z_{2I,1}, z_{2I,2}, z_{2I,3})$, and we define the four SI formulas to be:

$$\eta_{4I} = \sum_K n_K^- \text{ mod } 4 = \sum_K \frac{1}{2}(n_K^- - n_K^+) \text{ mod } 4, \tag{235}$$

and:

$$z_{2I,i=1,2,3} = C_{k_i=\pi} \text{ mod } 2 = \sum_{K, K_i=\pi} n_K^- \text{ mod } 2, \tag{236}$$

where K runs over the eight $\mathcal{I}$-invariant momenta in the first BZ, and $n_K^{\pm}$ are the number of Bloch states with ± 1 parity ($\mathcal{I}$) eigenvalues at K in the group of bands under consideration (typically the occupied bands). We find that $z_{2I,i}$ indicates the parity of the momentum-space Chern number in the $k_i = \pi$ plane, in agreement with the Chern number SI formulas previously introduced in SRefs. 156,164. Correspondingly, we find that η_{4I} mod 2 is the parity of the difference between the Chern numbers in the $k_z = 0$ and $k_z = \pi$ planes. Because a 3D $|C| = 1$ Weyl point is equivalent to the quantum critical point[178] between 2D Chern insulating phases with $|\Delta C| = 1$, then this implies that $\eta_{4I} = 1, 3$ correspond to Weyl SM (WSM) phases that satisfy the insulating compatibility relations (see SN 16), similar to the WSM and nodal-line SM phases previously analyzed in SRefs. 28,29,52,154,178,184,185. The boundary states of the $\eta_{4I} = 1, 3$ WSM phases differ from each other by a chiral hinge state or gapless surface states, because, as we will show below, the SI difference $\Delta\eta_{4I} = 3 - 1 = 2$ either corresponds to an AXI or a 3D QAH state. In this work, we refer to symmetry-indicated SM phases that satisfy the insulating compatibility relations as *Smith-index SMs* (SISMs).

Layer constructions – We will now formulate layer constructions of the symmetry-indicated spinful TI and TCI phases in double MSG 2.4 $P\bar{1}$. In each unit cell, we will use the relative 3D coordinates (x, y, z) to index layer positions, where the unit cell is defined as lying within $0 \leq x, y, z < 1$. In position space, an $\mathcal{I}$ center at $(0, 0, 0)$ transforms the coordinates (x, y, z) to $(-x, -y, -z)$. For a position $\mathbf{r}$ to be considered $\mathcal{I}$-invariant, we require that:

$$\mathcal{I}\mathbf{r} = \mathbf{r} \text{ mod } (1,0,0) \text{ mod } (0,1,0) \text{ mod } (0,0,1). \tag{237}$$

Consequently, the eight maximal Wyckoff positions (*i.e.* the $\mathcal{I}$ centers) in MSG 2.4 $P\bar{1}$ lie at $x, y, z = 0, 1/2$.

We next study the layer constructions of the insulating subset of the SI topological bands (*i.e.* the symmetry-indicated topological phases that do not correspond to Weyl SISMs with odd η_{4I} indices). We first introduce the layer construction generators, each of which is equivalent to a 3D QAH insulator[43,44,65,182], where the double SIs for each layer construction are given in the order $(\eta_{4I}, z_{2I,1}, z_{2I,2}, z_{2I,3})$:

1. An $\hat{\mathbf{x}}$-normal Chern layer with $C_x = \pm 1$ in the $x = 0$ plane has the SIs (2100).

2. An $\hat{\mathbf{x}}$-normal Chern layer with $C_x = \pm 1$ in the $x = \frac{1}{2}$ plane has the SIs (0100).

3. A $\hat{\mathbf{y}}$-normal Chern layer with $C_y = \pm 1$ in the $y = 0$ plane has the SIs (2010).

4. A $\hat{\mathbf{y}}$-normal Chern layer with $C_y = \pm 1$ in the $y = \frac{1}{2}$ plane has the SIs (0010).

5. A $\hat{\mathbf{z}}$-normal Chern layer with $C_z = \pm 1$ in the $z = 0$ plane has the SIs (2001).

6. A $\hat{\mathbf{z}}$-normal Chern layer with $C_z = \pm 1$ in the $z = \frac{1}{2}$ plane has the SIs (0001).

For the double SIs of the above layer constructions, we have adopted the convention used in SRefs. 26,29 in which commas are suppressed for specific values of the SIs [*e.g.* $(\eta_{4I}, z_{2I,1}, z_{2I,2}, z_{2I,3}) = (2100)$]. Below, we will detail the explicit calculations that we have performed to calculate the SIs of each layer construction, focusing on the cases of $\hat{\mathbf{z}}$-normal Chern layers with $C_z = 1$ respectively placed at $z = 0$ and $z = \frac{1}{2}$. In this work, we will only consider layer constructions of stable topological phases (as opposed to fragile phases, see SN 27 and SRefs. 5,6,136–141,143–148), which do not depend of the positions of layers with trivial 2D stable topological invariants [*i.e.* layers of 2D fragile phases or (obstructed) atomic limits][26]. Hence, for stable topological phases that admit layer constructions, the stable SIs are fully determined by the positions, orientations, and 2D stable topology of the layers.

First, we consider a crystal in double MSG 2.4 $P\bar{1}$ that is constructed of layered, $\hat{\mathbf{z}}$-normal Chern insulators with $C_z = 1$ that lie at $z = 0$ in each unit cell. We assume, without loss of generality, that each Chern insulator originates from placing one valence (occupied) spinful s orbital at $(x, y) = (0, 0)$, placing one conduction (unoccupied) spinful p orbital at $(x, y) = (0, 0)$, and then inverting bands at $(k_x, k_y) = (0, 0)$, resulting in the occupied parity eigenvalues:

$$\lambda'(0,0) = -1,\ \lambda'(\pi,0) = 1,\ \lambda'(0,\pi) = 1,\ \lambda'(\pi,\pi) = 1. \tag{238}$$

As shown in SRefs. 156,164 the Chern number C_z of each layer satisfies:

$$(-1)^{C_z} = \prod_{K} \prod_{n\in\text{occ}} \lambda'_n(K), \tag{239}$$

where K runs over the four $\mathcal{I}$-invariant momenta in SEq. (238), and $\lambda'_{K,n}$ is the parity eigenvalue of the nth energetically isolated band at K [though for the specific case that we are discussing, there is only one isolated (valence) band $n = 1$]. The parity eigenvalues shown in SEq. (238) indicate that each layer carries a nontrivial Chern number $C_z \text{ mod } 2 = 1$.

Next, we express the occupied band of each Chern layer in a basis of hybrid Wannier functions[114,115] in which states within the layers are exponentially localized in z and depend on the crystal momenta $k_{x,y}$. We then return to momentum space by Fourier-transforming the z component of the hybrid Bloch-Wannier wavefunction of the occupied band:

$$|\psi_{\mathbf{k}}\rangle = \frac{1}{\sqrt{N_z}} \sum_{z=0,\pm1\cdots} e^{-izk_z} |\psi_{k_x,k_y,z}\rangle, \tag{240}$$

in which N_z is the number of unit cells in the crystal in the z-direction. In the hybrid basis of (k_x, k_y, z):

$$\mathcal{I}(k_x, k_y, z) = (-k_x, -k_y, -z), \tag{241}$$

and hybrid coordinates $\mathbf{h}$ are considered to be $\mathcal{I}$-invariant if:

$$\mathcal{I}\mathbf{h} = \mathbf{h} \text{ mod } (2\pi, 0, 0) \text{ mod } (0, 2\pi, 0) \text{ mod } (0, 0, 1). \tag{242}$$

However, it is important to emphasize that the hybrid wavefunction $|\psi_{k_x,k_y,z}\rangle$ of each layer, unlike the Bloch wavefunction $|\psi_{\mathbf{k}}\rangle$, is generically not an eigenstate of $\mathcal{I}$:

$$\mathcal{I}|\psi_{k_x,k_y,z}\rangle = \lambda'(k_x, k_y)|\psi_{k_x,k_y,-z}\rangle, \tag{243}$$

in which $\lambda'(k_x, k_y)$ is the parity eigenvalue of the occupied band in the 2D BZ of a single Chern insulator. For a crystal in double MSG 2.4 $P\bar{1}$ furnished by $\hat{\mathbf{z}}$-normal, $C_z = 1$ Chern layers in the $z = 0$ plane of each cell, this implies

that the parity eigenvalues at the $\mathcal{I}$-invariant $\mathbf{k}$ points are given by:

$$\begin{aligned}
\mathcal{I}|\psi_{\mathbf{k}}\rangle &= \frac{1}{\sqrt{N_z}} \sum_{z=0,\pm1\cdots} e^{-izk_z}\mathcal{I}|\psi_{k_x,k_y,z}\rangle \\
&= \frac{1}{\sqrt{N_z}} \sum_{z=0,\pm1\cdots} e^{-izk_z}\lambda'(k_x,k_y)|\psi_{k_x,k_y,-z}\rangle \\
&= \lambda'(k_x,k_y)\left[\frac{1}{\sqrt{N_z}} \sum_{z=0,\pm1\cdots} e^{izk_z}|\psi_{k_x,k_y,z}\rangle\right] \\
&= \lambda'(k_x,k_y)\left[\frac{1}{\sqrt{N_z}} \sum_{z=0,\pm1\cdots} e^{-izk_z}\left[e^{ik_z}\right]^{2z}|\psi_{k_x,k_y,z}\rangle\right] \\
&= \lambda'(k_x,k_y)\left[\frac{1}{\sqrt{N_z}} \sum_{z=0,\pm1\cdots} e^{-izk_z}|\psi_{k_x,k_y,z}\rangle\right] \\
&= \lambda'(k_x,k_y)|\psi_{\mathbf{k}}\rangle,
\end{aligned} \tag{244}$$

where in the fifth line, we have used the relation $[e^{ik_z}]^{2z} = 1$ for $\mathcal{I}$-invariant momenta $k_z = 0, \pi$ and $z \in \mathbb{Z}$. Through SEq. (244), we determine that the 3D parity eigenvalues $\lambda(k_x,k_y,k_z)$ satisfy $\lambda(k_x,k_y,k_z) = \lambda'(k_x,k_y)$. From the parity eigenvalues of each layer listed in SEq. (238), this implies that:

$$\begin{aligned}
\lambda(0,0,0) &= -1,\ \lambda(\pi,0,0) = 1,\ \lambda(0,\pi,0) = 1,\ \lambda(\pi,\pi,0) = 1, \\
\lambda(0,0,\pi) &= -1,\ \lambda(\pi,0,\pi) = 1,\ \lambda(0,\pi,\pi) = 1,\ \lambda(\pi,\pi,\pi) = 1.
\end{aligned} \tag{245}$$

Substituting the parity eigenvalues from SEq. (245) into SEqs. (235) and (236), we obtain the SIs (2001) for a 3D crystal in double MSG 2.4 $P\bar{1}$ with $\hat{\mathbf{z}}$-normal $C_z = 1$ Chern insulators placed at $z = 0$ in each cell.

We next consider the case in which the 3D crystal is furnished with layers of $\hat{\mathbf{z}}$-normal, $C_z = 1$ Chern insulators that lie at $z \bmod 1 = \frac{1}{2}$. The Bloch wavefunction of the occupied band of the 3D crystal takes the form:

$$|\psi_{\mathbf{k}}\rangle = \frac{1}{\sqrt{N_z}} \sum_{z=\pm\frac{1}{2},\pm\frac{3}{2}\cdots} e^{-izk_z}|\psi_{k_x,k_y,z}\rangle. \tag{246}$$

Unlike previously in SEq. (244), for a crystal in double MSG 2.4 $P\bar{1}$ furnished by $\hat{\mathbf{z}}$-normal, $C_z = 1$ Chern insulator in the $z = \frac{1}{2}$ plane of each unit cell, the parity eigenvalues at the $\mathcal{I}$-invariant $\mathbf{k}$ points are given by:

$$\begin{aligned}
\mathcal{I}|\psi_{\mathbf{k}}\rangle &= \frac{1}{\sqrt{N_z}} \sum_{z=\pm\frac{1}{2},\pm\frac{3}{2}\cdots} e^{-izk_z}\mathcal{I}|\psi_{k_x,k_y,z}\rangle \\
&= \frac{1}{\sqrt{N_z}} \sum_{z=\pm\frac{1}{2},\pm\frac{3}{2}\cdots} e^{-izk_z}\lambda'(k_x,k_y)|\psi_{k_x,k_y,-z}\rangle \\
&= \lambda'(k_x,k_y)\left[\frac{1}{\sqrt{N_z}} \sum_{z=\pm\frac{1}{2},\pm\frac{3}{2}\cdots} e^{izk_z}|\psi_{k_x,k_y,z}\rangle\right] \\
&= \lambda'(k_x,k_y)\left[\frac{1}{\sqrt{N_z}} \sum_{z=\pm\frac{1}{2},\pm\frac{3}{2}\cdots} e^{-izk_z}\left[e^{ik_z}\right]^{2z}|\psi_{k_x,k_y,z}\rangle\right] \\
&= \lambda'(k_x,k_y)e^{ik_z}\left[\frac{1}{\sqrt{N_z}} \sum_{z=\pm\frac{1}{2},\pm\frac{3}{2}\cdots} e^{-izk_z}|\psi_{k_x,k_y,z}\rangle\right] \\
&= \lambda'(k_x,k_y)e^{ik_z}|\psi_{\mathbf{k}}\rangle,
\end{aligned} \tag{247}$$

where in the fifth line, we have exploited that the summation is taken over half-integer values of z. SEq. (247) implies that the 3D parity eigenvalues $\lambda(k_x,k_y,k_z)$ satisfy $\lambda(k_x,k_y,k_z) = e^{ik_z}\lambda'(k_x,k_y)$. From the parity eigenvalues of each

layer listed in SEq. (238), this indicates that:

$$\begin{aligned}\lambda(0,0,0) &= -1,\ \lambda(\pi,0,0)=1,\ \lambda(0,\pi,0)=1,\ \lambda(\pi,\pi,0)=1,\\ \lambda(0,0,\pi) &= 1,\ \lambda(\pi,0,\pi)=-1,\ \lambda(0,\pi,\pi)=-1,\ \lambda(\pi,\pi,\pi)=-1.\end{aligned} \tag{248}$$

Substituting the parity eigenvalues from SEq. (248) into SEqs. (235) and (236), we obtain the SIs (0001) for a 3D crystal of $\hat{\mathbf{z}}$-normal $C_z = 1$ Chern insulators placed at $z = \frac{1}{2}$ in double MSG 2.4 $P\bar{1}$.

For the remainder of this work, we will not explicitly calculate the 3D symmetry eigenvalues that are implied by each layer construction. However, because the unitary symmetries of magnetic crystals are drawn from the same set as the unitary symmetries of nonmagnetic crystals [*i.e.* because the unitary subgroups of both Type-II SGs and Type-III and IV MSGs are isomorphic to Type-I MSGs, see SN 2], then the symmetry eigenvalues of the magnetic layer constructions introduced in this work can be extrapolated from the analogous analyses of nonmagnetic layer constructions in SRef. 26.

The inversion $\mathbb{Z}_2$ invariant and AXIs – We find that $\eta_{4I} = 2$ if and only if the $\mathcal{I}$-center at the origin (000) is occupied by a layer with an odd Chern number. For 3D QAH states (*i.e.* 3D insulators with nonzero Chern numbers), the $\eta_{4I} = 0, 2$ phases have the same bulk response. For example, layer constructions 1 and 2 for double MSG 2.4 $P\bar{1}$ – which exhibit $\eta_{4I} = 2, 0$, respectively – are related by a shift of origin from (000) to $(00\frac{1}{2})$. Nevertheless, the boundary states of insulators with $\eta_{4I} = 0, 2$ are distinct. For a finite-size sample with an $\mathcal{I}$ center at (000), the state with $\eta_{4I} = 2$ has a single Chern layer passing through the $\mathcal{I}$ center, and pairs of Chern layers at positions $(00, \pm z)$. In the finite sample, the total Chern number is therefore odd, and there is an $\mathcal{I}$-symmetric chiral hinge (or surface) mode surrounding the sample guaranteed by the net-odd Chern number. However, in the state with $\eta_{4I} = 0$, all of the Chern layers appear in pairs at the positions $(00, \pm z)$, such that the total Chern number is even. This implies the possibility of a completely gapped finite sample (*i.e.* a total sample Chern number of zero).

For 3D insulators with vanishing Chern numbers, $\eta_{4I} = 0, 2$ correspond to trivial insulators and AXIs, respectively. For example, the TCI constructed by one $C_z = 1$ layer in the $z = 0$ plane and one $C_z = -1$ layer in the $z = \frac{1}{2}$ plane is an AXI with the double SIs (2000)[47,53,54,57,58,65,183]. The chiral hinge states of the AXI can be understood by observing that the chiral modes on the boundary of the layered crystal alternate in direction, and can hence pairwise annihilate – when the finite-sized crystal is $\mathcal{I}$-symmetric, there is an unpaired chiral mode that is equivalent to a boundary-encircling chiral hinge state. Specializing to the even sector of η_{4I}, this implies that an inversion $\mathbb{Z}_2$ invariant may be defined as:

$$\eta'_{2I} = \frac{1}{2}\eta_{4I} \bmod 2. \tag{249}$$

We emphasize that it is η_{4I} – as opposed to η'_{2I} – that is returned by the Smith normal form calculation (see SN 28) for double MSG 2.4 $P\bar{1}$. The non-minimal index η'_{2I} is integer-valued only for 3D insulators or WSMs with even numbers of Weyl points in each half of the bulk BZ. Nevertheless, as we will show below, in many higher-symmetry double SSGs in which the SIs depend on the SIs in double MSG 2.4 $P\bar{1}$ (see SN 39), the Smith normal form calculation *does* return η'_{2I}. From previous works[28,38,40–60], we recognize that $\eta'_{2I} = 1$ is related to the axion angle $\theta = \pi$:

$$\theta \bmod 2\pi = \pi\eta'_{2I}. \tag{250}$$

However, it is crucial to note that the axion angle $\theta = \pi$ does not always indicate an axionic band-insulating phase (*i.e.* an AXI or 3D TI, see SRefs. 28,32,38,40–60,160). For example, consider the case of a crystal in MSG 2.4 $P\bar{1}$ furnished by one $C_z = 1$ layer in the $z = 0$ plane and one $C_z = 1$ layer in the $z = \frac{1}{2}$ plane – the bulk topological phase is *not* an AXI, but is instead a 3D QAH insulator with $C_z = 2$ per unit cell. For the $C_z = 2$ QAH insulator, the SIs [(2000)] are the same as those of the AXI discussed in the text surrounding SEqs. (249) and (250), indicating that $\theta = \pi\eta'_{2I} = \pi$, despite the fact that the bulk is not an AXI. This can be understood by recognizing that the axion θ angle is origin-dependent when the $\mathbb{Z}$-valued, *non-symmetry-indicated* Chern numbers of a 3D crystal do not vanish[47,57,58,186,187], and hence θ can still be nonzero in a 3D QAH phase depending on the choice of origin. Therefore, in order for SEq. (250) to indicate the *origin-independent* θ angle of an AXI, it is additionally required that the total Chern numbers $C_{x,y,z}$ vanish in each unit cell. Lastly, we note that SEq. (250) differs by π from the definition of θ as a "Chern number polarization" employed in SRefs. 47,57,186. Hence, in 3D insulators with non-vanishing position-space Chern numbers (*i.e.* nonzero total Chern numbers in any direction summed across the layers in each position-space unit cell) and origin- (*i.e.* convention-) dependent θ angles, the SIs introduced in this work [*e.g.* SEq. (250)] return values of θ that are shifted from the values in SRefs. 47,57,186 by π. Importantly, however, both SEq. (250) and the Chern number polarization in SRefs. 47,57,186 correctly diagnose the convention-independent bulk θ angle of AXIs to be $\theta = \pi$.

Relationship with the SIs in other double SSGs – As shown in SRefs. 24,26,27, the double SIs in double SG 2.5 $P\bar{1}1'$ take the same form as SEqs. (236) and (235) under the replacement of n_K^- with $n_K^-/2$ [*i.e.* the number of energetically isolated *Kramers pairs* of Bloch states at K]. The SI topological bands in double SG 2.5 $P\bar{1}1'$ subduced onto double MSG 2.4 $P\bar{1}$ imply the double SI dependencies:

$$(z_4, z_{2w,1}, z_{2w,2}, z_{2w,3})_{P\bar{1}1'} \to (\eta_{4I}, z_{2I,1}, z_{2I,2}, z_{2I,3})_{P\bar{1}} = (2(z_4 \bmod 2), 000)_{P\bar{1}}. \tag{251}$$

b. Double SIs in Type-I Double MSG 3.1 P2

The double MSG 3.1 $P2$ is generated by $\{E|100\}$, $\{E|010\}$, $\{E|001\}$, and $\{C_{2y}|\mathbf{0}\}$, and has the double SI group $\mathbb{Z}_2$. We first recall the formula established in SRefs. 43,156,164 for the parity of the Chern number in a $\hat{\mathbf{y}}$-normal 2D insulator with $\{C_{2y}|00\}$ symmetry:

$$(-1)^{C_y} = \prod_{n\in occ}\prod_{K}\zeta_n(K), \tag{252}$$

where C_y is the Chern number in the y-direction, $\zeta_n(K)$ is the $\{C_{2y}|00\}$ eigenvalue of the nth energetically isolated state at K, and K runs over the four $\{C_{2y}|00\}$-invariant momenta in 2D. Using SEq. (252), we define the double SI z_{2R} of MSG 3.1 $P2$ to be the parity of the Chern number C_y in the $k_y = \pi$ plane:

$$z_{2R} = C_{k_y=\pi} \bmod 2 = \sum_{K=Z,D,C,E} n_K^{\frac{1}{2}} \bmod 2, \tag{253}$$

where $n_K^{\frac{1}{2}}$ is the number of energetically isolated states with the $\{C_{2y}|\mathbf{0}\}$ eigenvalue $-i$ [corresponding to an angular momentum (modulo 2) of $j = \frac{1}{2}$] at K. For 3D insulating phases, the Chern numbers in all of the BZ planes of constant k_y for $-\pi \le k_y < \pi$ must be the same (otherwise, there would be bulk Weyl points, and the bulk would not be an insulator). Hence, a 3D insulator with $z_{2R} = 1$ is a 3D QAH state with $C_y \bmod 2 = 1$.

If the symmetry operation $\{\mathcal{T}|\mathbf{0}\}$ were added, a crystal in double MSG 3.1 $P2$ would become invariant under Type-II double SG 3.2 $P21'$. In $P21'$, states at the four TRIM points K in SEq. (253) form Kramers pairs with opposite $\{C_{2y}|\mathbf{0}\}$ eigenvalues, causing $n_K^{\frac{1}{2}}$ to be even, and z_{2R} to be zero. This agrees with the absence of double SIs in Type-II double SG 3.2 $P21'$ (see SN 39), and the requirement that the position-space Chern numbers $C_{x,y,z}$ vanish in a nonmagnetic ($\mathcal{T}$-symmetric) crystal[188,189].

c. Double SIs in Type-I Double MSG 10.42 P2/m

The double MSG 10.42 $P2/m$ is generated by $\{E|100\}$, $\{E|010\}$, $\{E|001\}$, $\{C_{2y}|\mathbf{0}\}$, and $\{m_y|\mathbf{0}\}$.

SIs – The double MSG 10.42 $P2/m$ has the SI group $\mathbb{Z}_2^3$. In the physical basis, the three double SIs of double MSG 10.42 $P2/m$ ($\delta_{2m}, z_{2m,\pi}^+, z_{2m,\pi}^-$) have the respective SI formulas:

$$\delta_{2m} = C_\pi^+ - C_0^- \bmod 2 = \sum_{K=Z,D,C,E} n_K^{\frac{1}{2},+i} - \sum_{K=\Gamma,A,B,Y} n_K^{\frac{1}{2},-i} \bmod 2, \tag{254}$$

$$z_{2m,\pi}^+ = C_\pi^+ \bmod 2 = \sum_{K=Z,D,C,E} n_K^{\frac{1}{2},+i} \bmod 2, \tag{255}$$

$$z_{2m,\pi}^- = C_\pi^- \bmod 2 = \sum_{K=Z,D,C,E} n_K^{\frac{1}{2},-i} \bmod 2, \tag{256}$$

where $n_K^{j,\pm i}$ is the number of occupied states with angular momentum j, the $\{C_{2y}|0\}$ eigenvalue $e^{-i\pi j}$, and the $\{m_y|0\}$ eigenvalue $\pm i$. Because the matrix representative of $\{m_y|0\}$ commutes with the matrix representative of $\{C_{2y}|0\}$ in all double-valued small irreps at each of the $\mathcal{I}$-invariant $\mathbf{k}$ points in double MSG 10.42 $P2/m$, then $z_{2m,\pi}^\pm$, respectively indicate the Chern number parities in the mirror sector of the $k_y = \pi$ plane with $\{m_y|0\}$ eigenvalue $\pm i$. As discussed in SN 29, if the bulk is a 3D insulator, then the occupied states in the $k_y = 0, \pi$ planes have the same total Chern

numbers ($i.e$ the sum of the Chern numbers over the two mirror sectors in each of the $k_y = 0, \pi$ planes is the same), because the insulating compatibility relations require that the occupied bands in the $k_y = 0, \pi$ planes have the same $\{C_{2y}|0\}$ eigenvalues.

Layer constructions – To diagnose the topology associated to each nontrivial value of the double SIs, we employ the layer construction method. We denote the Chern numbers of the occupied bands in each mirror sector – which we term the *mirror sector Chern numbers* – as $(C^+_{k_y=0}, C^-_{k_y=0}, C^+_{k_y=\pi}, C^-_{k_y=\pi})$. The insulating compatibility relations require that $C^+_{k_y=0} + C^-_{k_y=0} \bmod 2 = C^+_{k_y=\pi} + C^-_{k_y=\pi} \bmod 2$. We emphasize that the double SIs in SEqs. (254), (255), and (256) are fully determined by the above mirror sector Chern numbers $(C^+_{k_y=0}, C^-_{k_y=0}, C^+_{k_y=\pi}, C^-_{k_y=\pi})$. We next calculate the minimal double SIs of double MSG 10.42 $P2/m$ in the order $(\delta_{2m}, z^+_{2m,\pi}, z^-_{2m,\pi})$, as well as the subduced double SIs $(\eta_{4I}, z_{2I,1}, z_{2I,2}, z_{2I,3})_{P\bar{1}}$ in the subgroup double MSG 2.4 $P\bar{1}$ for a physical comparison and to identify symmetry-indicated AXI phases in MSG 10.42 $P2/m$.

1. A $\hat{\mathbf{y}}$-normal layer with $C^+_y = 1$, $C^-_y = 0$ in the $y = 0$ plane has the mirror sector Chern numbers =(1010) and the SIs (110). The subgroup SIs are $(\eta_{4I}, z_{2I,1}, z_{2I,2}, z_{2I,3})_{P\bar{1}} = (2010)_{P\bar{1}}$.

2. A $\hat{\mathbf{y}}$-normal layer with $C^+_y = 0$, $C^-_y = 1$ in the $y = 0$ plane has the mirror sector Chern numbers (0101) and the SIs (101). The subgroup SIs are $(2010)_{P\bar{1}}$.

3. A $\hat{\mathbf{y}}$-normal layer with $C^+_y = 1$, $C^-_y = 0$ in the $y = \frac{1}{2}$ plane has the mirror sector Chern numbers (1001) and the SIs (001). The subgroup SIs are $(0010)_{P\bar{1}}$.

4. A $\hat{\mathbf{y}}$-normal layer with $C^+_y = 0$, $C^-_y = 1$ in the $y = \frac{1}{2}$ plane has the mirror sector Chern numbers (0110) and the SIs (010). The subgroup SIs are $(0010)_{P\bar{1}}$.

Relationship with the SIs in other double SSGs – To identify the AXI phases in double MSG 10.42 $P2/m$, we subduce the SIs onto the SIs of double MSG 2.4 $P\bar{1}$:

$$\left(\delta_{2m}, z^+_{2m,\pi}, z^-_{2m,\pi}\right)_{P2/m} \rightarrow (\eta_{4I}, z_{2I,1}, z_{2I,2}, z_{2I,3})_{P\bar{1}} = \left(2\delta_{2m}, 0,\ z^+_{2m,\pi} + z^-_{2m,\pi},\ 0\right)_{P\bar{1}}. \tag{257}$$

We find that both the (100) and (111) states in double MSG 10.42 $P2/m$ are consistent with AXI phases [but may also, for example, be 3D QAH phases, see SN 31 a]. We label the four layer constructions as $L_{1,2,3,4}$. The (100) and (111) states in double MSG 10.42 $P2/m$ can be constructed as $L_1 - L_4$ and $L_1 - L_3$, respectively. Lastly, $-L_3$ ($-L_4$) has the same construction as L_3 (L_4), except for a difference in the position-space mirror sector Chern number $C^+_y = -1$ ($C^-_y = -1$).

Lastly, Type-II double SSG 10.43 $P2/m1'$ – the double SSG that results from adding $\{\mathcal{T}|\mathbf{0}\}$ symmetry to Type-I double MSG 10.42 $P2/m$ – has the SI group $\mathbb{Z}_4 \times \mathbb{Z}_2^3$. The subduction relations between the double SIs in double SSG 10.43 $P2/m1'$ and double MSG 10.42 $P2/m$ are given by:

$$(z_4, z_{2w,1}, z_{2w,2}, z_{2w,3})_{P2/m1'} \rightarrow (\delta_{2m}, z^+_{2m,\pi}, z^-_{2m,\pi})_{P2/m} = (z_4 \bmod 2, z_{2w,2}, z_{2w,2})_{P2/m}. \tag{258}$$

d. Double SIs in Type-I Double MSG 47.249 *Pmmm*

The double MSG 47.249 $Pmmm$ is generated by $\{E|100\}$, $\{E|010\}$, $\{E|001\}$, $\{m_x|\mathbf{0}\}$, $\{m_y|\mathbf{0}\}$, and $\{\mathcal{I}|\mathbf{0}\}$.

SIs – The double MSG 47.249 $Pmmm$ has the SI group $\mathbb{Z}_4 \times \mathbb{Z}_2^3$. In double-valued small irreps of the little groups at the $\mathcal{I}$-invariant $\mathbf{k}$ points, the matrix representatives of perpendicular mirror symmetries ($e.g.$ $\{m_x|\mathbf{0}\}$ and $\{m_y|\mathbf{0}\}$) anticommute. Hence, Bloch states at the eight $\mathcal{I}$-invariant momenta must be at least twofold degenerate (and in fact are exactly twofold degenerate in double MSG 47.249 $Pmmm$). The double SIs can be chosen to be the same as the double SIs of SSG 47.250 $Pmmm1'$, because the addition of $\mathcal{T}$ symmetry to double MSG 47.249 $Pmmm$ does not change the dimensions and characters of the small irreps at the high-symmetry BZ points or the compatibility relations between the high-symmetry-point small irreps. In the physical basis, the $\mathbb{Z}_4$ double SI is:

$$z_4 = \sum_K \frac{1}{4}(n^-_K - n^+_K) \bmod 4, \tag{259}$$

where K indexes all $\mathcal{I}$-invariant momenta and $n^{\pm}_K$ is the number of occupied states with ± 1 parity ($\mathcal{I}$) eigenvalues at K. z_4 has the same form as η_{4I} [SEq. (235)], but carries an additional prefactor of 1/2. The extra factor of 1/2 in SEq. (259) can be understood from the double degeneracy of the Bloch states at the $\mathcal{I}$-invariant TRIM points, where the two states in each doublet have the same parity eigenvalues and complex-conjugate pairs of spinful mirror

eigenvalues $\pm i$, due to the anticommutation relations discussed above. Hence, the SI formula for z_4 [SEq. (259)] is simply one half of the SI formula for η_{4I} [SEq. (235)] (before applying the modulo 4 operation). The three $\mathbb{Z}_2$ SIs are the mirror Chern number parities in the $k_{1,2,3} = \pi$ planes:

$$z_{2w,i=1,2,3} = \sum_{K, K_i = \pi} \frac{1}{2} n_K^- \bmod 2. \tag{260}$$

Specifically, because an in-plane mirror operation reverses the sign of a 2D Chern number, and because all of the mirror planes in the bulk BZ have additional in-plane mirror symmetries (*e.g.* the Hamiltonian in each BZ mirror plane must respect the symmetries of magnetic layer group[10,39,69,70,73,74] $pmmm$), then the net Chern number in each BZ mirror plane in double MSG 47.249 $Pmmm$ must vanish. For a group of bands in a mirror-invariant BZ (position-space) plane for which $C_{k_i}^+ = -C_{k_i}^-$ ($C^+ = -C^-$), we then define the *mirror Chern number*[161,162] to be $|C_{k_i}^+|$ ($|C^+|$).

Layer constructions – To diagnose the topology associated to each nontrivial value of the double SIs, we employ the layer construction method. In the layer constructions below, $C^+ = -C^-$ due to the net-zero Chern numbers enforced by the mirror symmetries. Hence, we will omit C^- in further discussions of the topology in double MSG 47.249 $Pmmm$. The layer constructions for the double SIs $(z_4, z_{2w,1}, z_{2w,2}, z_{2w,3})$ of MSG 47.249 $Pmmm$ are given by:

1. An $\hat{\mathbf{x}}$-normal mirror Chern layer with $C_x^+ = 1$ in the $x = 0$ plane has the mirror sector Chern numbers $(C_{k_x=0}^+, C_{k_x=\pi}^+) = (11)$ and the SIs (2100).

2. An $\hat{\mathbf{x}}$-normal mirror Chern layer with $C_x^+ = 1$ in the $x = \frac{1}{2}$ plane has the mirror sector Chern numbers $(C_{k_x=0}^+, C_{k_x=\pi}^+) = (1, -1)$ and the SIs (0100).

3. A $\hat{\mathbf{y}}$-normal mirror Chern layer with $C_y^+ = 1$ in the $y = 0$ plane has the mirror sector Chern numbers $(C_{k_y=0}^+, C_{k_y=\pi}^+) = (11)$ and the SIs (2010).

4. A $\hat{\mathbf{y}}$-normal mirror Chern layer with $C_y^+ = 1$ in the $y = \frac{1}{2}$ plane has the mirror sector Chern numbers $(C_{k_y=0}^+, C_{k_y=\pi}^+) = (1, -1)$ and the SIs (0010).

5. A $\hat{\mathbf{z}}$-normal mirror Chern layer with $C_z^+ = 1$ in the $z = 0$ plane has the mirror sector Chern numbers $(C_{k_z=0}^+, C_{k_z=\pi}^+) = (11)$ and the SIs (2001).

6. A $\hat{\mathbf{z}}$-normal mirror Chern layer with $C_z^+ = 1$ in the $z = \frac{1}{2}$ plane has the mirror sector Chern numbers $(C_{k_z=0}^+, C_{k_z=\pi}^+) = (1, -1)$ and the SIs (0001).

The layer-construction calculations in this section parallel with the previous calculations in SN 31 a of the layer constructions of the insulating phases in double MSG 2.4 $P\bar{1}$. Hence, we will only consider layer construction 5 as an example of the generalization from the layer constructions and bulk topology in double MSG 2.4 $P\bar{1}$ to that in double MSG 47.249 $Pmmm$.

In layer construction 5, we take each layer to consist of a $\hat{\mathbf{z}}$-normal 2D mirror Chern insulator ($C_z^+ = -C_z^- = 1$) with the occupied parity ($\mathcal{I}$) eigenvalues $\lambda'_{1,2}(k_x, k_y) = --, ++, ++, ++$ at $(k_x, k_y) = (00), (0\pi), (\pi 0), (\pi\pi)$, respectively. The subscripts $1, 2$ on $\lambda'_{1,2}(k_x, k_y)$ represent the $\{m_z|\mathbf{0}\}$ eigenvalue sectors i and $-i$, respectively. Applying the Fourier transformation in SEq. (240), we find that the parity eigenvalues of the 3D system are given by $\lambda_{1,2}(k_x, k_y, k_z) = \lambda'_{1,2}(k_x, k_y)$ [SEq. (244)]. This implies that $\lambda_{1,2}(k_x, k_y, k_z) = --, ++, ++, ++, --, ++, ++, ++$ for $(k_x, k_y, k_z) = (000), (0\pi 0), (\pi 00), (\pi\pi 0), (00\pi), (0\pi\pi), (\pi 0\pi), (\pi\pi\pi)$, respectively. Substituting the parity eigenvalues of layer construction 5 into SEqs. (259) and (260), we obtain the SIs (2001).

Axion insulators – We find that states with odd z_4 SIs cannot be constructed from layers of 2D stable topological phases. However, we may still use subduction relations to determine the bulk topology of insulators with odd values of z_4. First, as we will show below, (1000) and (3000) subduce to $(2000)_{P\bar{1}}$ in MSG 2.4 $P\bar{1}$. Hence, if the (1000) and (3000) phases in double MSG 47.249 $Pmmm$ are insulating, then the bulk insulator must either be an AXI or a 3D QAH state. Because the net Chern numbers $C_{x,y,z} = 0$ must vanish if the bulk is gapped, due to the mirror symmetries of double MSG 47.249 $Pmmm$, then the (1000) and (3000) states must be AXIs. This result can also be understood by subducing from a $\mathcal{T}$-symmetric SSG. Specifically, because (1000) and (3000) in MSG 47.249 $Pmmm$ can respectively be subduced from $(1000)_{Pmmm1'}$ and $(3000)_{Pmmm1'}$ in Type-II SG 47.250 $Pmmm1'$, which correspond to $\mathcal{T}$-symmetric 3D TIs with $\theta = \pi$[24,26,27], then (1000) and (3000) are compatible with bulk-gapped states. Hence, we conclude that 3D insulators with (1000) and (3000) in double MSG 47.249 $Pmmm$ are AXIs, *without ambiguity*. We

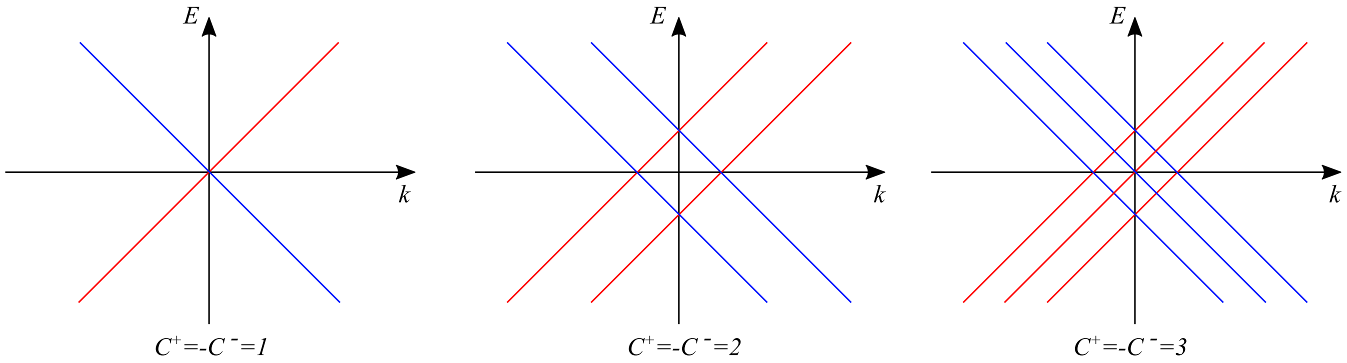

Supplementary Figure 20: Surface Dirac points protected by mirror Chern numbers. In this figure, we respectively depict the surface states of insulators with the bulk mirror Chern numbers $C^{+} = -C^{-} = 1, 2, 3$, where $C^{\pm}$ respectively refer to the Chern number in the mirror sector with eigenvalue $\pm i$. In each panel, we depict a topological surface band structure along a mirror-invariant surface BZ line, where the red and blue lines respectively indicate bands with the mirror eigenvalues i and $-i$. At half-filling, the number of twofold surface Dirac points is given by the mirror Chern number $|C^{+}|$, where $|C^{+}| = |C^{-}|$.

conjecture that the (1000) and (3000) AXIs in MSG 47.249 $Pmmm$ can be constructed using the topological crystal method[168], which additionally incorporates cell complexes of 2D Chern insulators, TIs, and TCIs.

Helical HOTI phases protected by mirror – First, the double SIs $(2000)_{Pmmm1'}$ of Type-II double SSG 47.250 $Pmmm1'$ correspond to a helical (non-axionic, *i.e.* θ mod $2\pi = 0$) HOTI protected by $\mathcal{I}$ and $\mathcal{T}$ symmetries. In the $\mathcal{I}$- and $\mathcal{T}$-symmetric HOTI phase, an odd number of helical modes encircle a finite sample with $\mathcal{I}$-symmetry. Because double SSG 47.250 $Pmmm1'$ contains $\{m_{x,y,z}|\mathbf{0}\}$ symmetries, then a single helical hinge mode on a boundary must also be pinned to the hinge projection of a bulk mirror plane, and must indicate a bulk mirror Chern number $C_m = 2$ (because a mirror-invariant hinge is a 1D domain wall between two 2D surfaces with two massive twofold Dirac cones with oppositely-signed masses related by mirror symmetry, see SRefs. 26,34,39).

Returning to the magnetic subgroup Type-I MSG 47.249 $Pmmm$ of Type-II SG 47.250 $Pmmm1'$, we denote the six layer constructions introduced in this section as L_a $(a = 1\cdots 6)$, respectively. Without loss of generality, we consider $(2n+1)L_1 \oplus (2m+1)L_2$. We next consider a 90° hinge of a z-directed, mmm-invariant rod that lies between $x+y > 0$, $x - y < 0$, where the rod is centered at the origin. On the 1D hinge, $(2n + 1)L_1$, which has the mirror sector Chern numbers $C^{+}_{x} = -C^{-}_{y} = 2n + 1$ and has 2D TCI layers at $x = 0, \pm 1\cdots$, will contribute $2n + 1$ helical modes to the hinge at $x = y = 0$.

We next note that the bulk mirror Chern numbers $(C^{+}_{k_x=0}, C^{+}_{k_x=\pi})$ are $(2n+2m+2, 2n-2m)$. If $n-m$ mod $2 = 0$, then $C^{+}_{k_x=0}$ mod $4 = 2$ and $C^{+}_{k_x=\pi}$ mod $4 = 0$, and if $n-m$ mod $2 = 1$, then $C^{+}_{k_x=0}$ mod $4 = 0$ and $C^{+}_{k_x=\pi}$ mod $4 = 2$. In general, the SIs (2000) can be constructed as $(2n + 1)L_1 \oplus (2m + 1)L_2$, or $(2n + 1)L_3 \oplus (2m + 1)L_4$, or $(2n+1)L_5 \oplus (2m+1)L_6$ $(m, n \in \mathbb{Z})$, or through any superposition of an odd number of the aforementioned layer constructions. Hence, there exists a direction $i \in \{x, y, z\}$ such that the mirror Chern numbers in the i direction are either $(C^{+}_{k_i=0}$ mod $4, C^{+}_{k_i=\pi}$ mod $4) = (2, 0)$ or $(C^{+}_{k_i=0}$ mod $4, C^{+}_{k_i=\pi}$ mod $4) = (0, 2)$. Therefore, the bulk of the (2000) is a mirror TCI. Nevertheless, in this work, we refer to the (2000) phase in double MSG 47.249 $Pmmm$ as a helical HOTI, because the (2000) phase of double MSG 47.249 can be connected to a $(z_4, z_{2w,1}, z_{2w,2}, z_{2w,3})_{Pmmm1'} = (2000)_{Pmmm1'}$ mirror TCI phase in the $\mathcal{T}$-symmetric supergroup Type-II double SG 47.250 $Pmmm1'$ without closing a bulk or surface gap. In turn, the $(2000)_{Pmmm1'}$ TCI phase subduces to an $\mathcal{I}$- and $\mathcal{T}$-protected $(z_4, z_{2w,1}, z_{2w,2}, z_{2w,3})_{P\bar{1}1'} = (2000)_{P\bar{1}1'}$ helical HOTI in Type-II double SG 2.5 $P\bar{1}1'$ [see SN 31 s and SRefs. 24,26–28]. To summarize, there exists at least one mirror-symmetric surface in the (2000) HOTI state that has $2 + 4n$ $(n \in \{\mathbb{Z}^{+}, 0\})$ twofold Dirac points, in agreement with nontrivial even bulk mirror Chern number. We depict the anomalous surface and hinge states of the (2000) HOTI phase in SFig. 21(a).

For completeness, we next consider the boundary states of the layer construction $(2n + 1)L_1 \oplus (2m + 1)L_2$. The Chern numbers in the $\{m_x|\mathbf{0}\}$ mirror sectors are $C^{+}_{k_x=0} = -C^{-}_{k_x=0} = 2n + 2m + 2$, $C^{+}_{k_x=\pi} = -C^{-}_{k_x=\pi} = 2n - 2m$. We consider either a $\hat{\mathbf{y}}$- or a $\hat{\mathbf{z}}$-normal surface, either of which preserves $\{m_x|\mathbf{0}\}$ mirror symmetry. In the 2D surface BZ, the bulk Chern number $C^{+}_{k_x=0}$ mandates the presence of $|2n + 2m + 2|$ twofold Dirac points on the $k_x = 0$ line (see SFig. 20), and $C^{+}_{k_x=\pi}$ mandates the presence of $|2n - 2m|$ Dirac points on the $k_x = \pi$ line. Hence the total number of twofold surface Dirac points is $|2n + 2m + 2| + |2n - 2m|$ mod $4 = 2$. Similarly, $(2n + 1)L_3 \oplus (2m + 1)L_4$ and $(2n + 1)L_5 \oplus (2m + 1)L_6$ will exhibit $2 + 4n$ $(n \in \{\mathbb{Z}^{+}, 0\})$ twofold Dirac points on $\{m_y|\mathbf{0}\}$- and $\{m_z|\mathbf{0}\}$-preserving surfaces, respectively. In SN 34, we will prove that, on surfaces of the (2000) state that respect the symmetries of

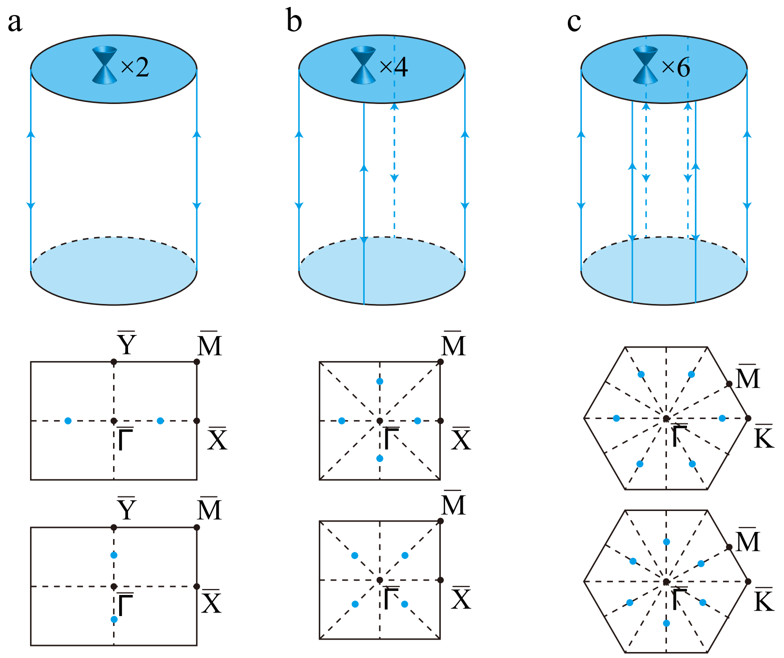

Supplementary Figure 21: The boundary states of the non-axionic magnetic HOTI phases in double MSGs (a) 47.249 $Pmmm$, (b) 123.339 $P4/mmm$, (c) 191.233 $P6/mmm$. In the top panel, we show the mirror-protected surface twofold Dirac cones and side-surface helical hinge modes of each non-axionic magnetic HOTI (see SN 33 for further details). The symmetry groups of the top ($\hat{\mathbf{z}}$-normal) surfaces are Type-I magnetic wallpaper groups (a) pmm, (b) $p4m$, and (c) $p6m$ (see SRefs. 36,39,73–75 and SN 33). We note that, in this work, we have labeled wallpaper groups – which are also sometimes termed *plane groups* – using the short notation previously employed in SRefs. 39,71,73; in the long notation of the Get Plane Gen tool on the BCS[13,14], the magnetic wallpaper groups in (a-c) pmm, $p4m$, and $p6m$ are respectively labeled by the symbols $p2mm$, $p4mm$, and $p6mm$. In (a-c), the helical hinge states are pinned to the hinge projections of the bulk mirror planes, and therefore originate from nontrivial bulk mirror Chern numbers. In the middle and bottom panels, we depict two possible configurations of anomalous twofold Dirac points in the top-surface BZ, where the dashed lines represent the top-surface projections of bulk mirror planes. In SN 34, we will introduce magnetic Dirac fermion doubling theorems for 2D insulators with the magnetic wallpaper groups of the top surfaces in (a-c). The fermion doubling theorems for the top-surface wallpaper groups in (a-c) are respectively circumvented by the non-axionic magnetic HOTI phases discovered in this work.

Type-I double magnetic wallpaper group[36,39,73–75] pmm, the presence of $2+4n$ ($n \in \{\mathbb{Z}^+, 0\}$) twofold surface Dirac points circumvents the fermion multiplication theorem for 2D lattices with double magnetic wallpaper group pmm.

Relationship with the SIs in other double SSGs – To identity the AXI phases, we subduce the SIs onto double MSG 2.4 $P\bar{1}$. As explained in the text following SEq. (259), because each doublet of Bloch states at an $\mathcal{I}$-invariant $\mathbf{k}$ point in MSG 47.249 $Pmmm$ has the same parity eigenvalues (and complex-conjugate mirror eigenvalues), then z_4 is simply a doubling of η_{4I}. Hence $\eta_{4I} = 2z_4 \mod 4$. Similarly, $z_{2I,i} = 2z_{2w,i} \mod 2 = 0$. In summary:

$$(z_4, z_{2w,1}, z_{2w,2}, z_{2w,3})_{Pmmm} \rightarrow (\eta_{4I}, z_{2I,1}, z_{2I,2}, z_{2I,3})_{P\bar{1}} = (2z_4 \mod 4, 000)_{P\bar{1}}. \tag{261}$$

Hence, the (1000) and (3000) states in double MSG 47.249 $Pmmm$, if gapped, correspond to AXIs.

Lastly, the correspondence between the double SIs of Type-I double MSG 47.249 $Pmmm$ and the double SIs of Type-II double SSG 47.250 $Pmmm1'$ is one-to-one.

e. *Double SIs in Type-I Double MSG 75.1 P4*

The double MSG 75.1 $P4$ is generated by $\{E|100\}$, $\{E|010\}$, $\{E|001\}$, and $\{C_{4z}|\mathbf{0}\}$.

The double SI group of double MSG 75.1 $P4$ is $\mathbb{Z}_4$. To determine the physical basis for the double SIs, we first recall the formula for the Chern number in the presence of fourfold rotation symmetry[164]:

$$i^C = (-1)^{N_{\text{occ}}} \prod_{n \in occ} \xi_n(00)\xi_n(\pi\pi)\zeta_n(0\pi), \tag{262}$$

where $\xi_n(K)$ is the $\{C_{4z}|\mathbf{0}\}$ eigenvalue of the nth occupied state at K, and $\zeta_n(K)$ is the $\{C_{2z}|\mathbf{0}\}$ eigenvalue of the nth occupied state at K. We can define the SI as the Chern number in the $k_z=\pi$ plane modulo 4:

$$\begin{aligned} z_{4R} = C_{k_z=\pi} \bmod 4 &= 2N_{\text{occ}} + \sum_{K=Z,A}\left(-\frac{1}{2}n_K^{\frac{1}{2}} + \frac{1}{2}n_K^{-\frac{1}{2}} - \frac{3}{2}n_K^{\frac{3}{2}} + \frac{3}{2}n_K^{-\frac{3}{2}}\right) - n_R^{\frac{1}{2}} + n_R^{-\frac{1}{2}} \bmod 4 \\ &= \sum_{K=Z,A}\left(-\frac{1}{2}n_K^{\frac{1}{2}} + \frac{1}{2}n_K^{-\frac{1}{2}} - \frac{3}{2}n_K^{\frac{3}{2}} + \frac{3}{2}n_K^{-\frac{3}{2}}\right) + n_R^{\frac{1}{2}} - n_R^{-\frac{1}{2}} \bmod 4, \end{aligned} \tag{263}$$

where $n_{Z,A}^{\frac{1}{2},-\frac{1}{2},\frac{3}{2},-\frac{3}{2}}$ are the number of occupied states with $\{C_{4z}|\mathbf{0}\}$ eigenvalues $e^{-i\frac{\pi}{4}}$, $e^{i\frac{\pi}{4}}$, $e^{-i\frac{3\pi}{4}}$, $e^{i\frac{3\pi}{4}}$, respectively, and $n_R^{\frac{1}{2},-\frac{1}{2}}$ are the number of occupied states with $\{C_{2z}|\mathbf{0}\}$ eigenvalues $e^{-i\frac{\pi}{2}}$, $e^{i\frac{\pi}{2}}$, respectively. In deriving SEq. (263), we have used the relation $N_{\text{occ}} = n_R^{\frac{1}{2}} + n_R^{-\frac{1}{2}}$.

Due to the compatibility relations and the fact that a chiral fermion in 3D occurs when there is a change in a momentum-space Chern number, a 3D insulator must satisfy $C_{k_z=\pi} = C_{k_z}$ for all k_z. Hence, we may have equivalently defined the SI z_{4R} using the occupied $\{C_{4z}|\mathbf{0}\}$ and $\{C_{2z}|\mathbf{0}\}$ eigenvalues in $k_z=0$ plane, or in any other BZ plane of constant k_z. In general, in this work, in order to match the convention employed in SRef. 26, we will use the rotation eigenvalues in the $k_i=\pi$ plane to define double SIs in the physical basis. To summarize, if a 3D system is insulating and exhibits $z_{4R}\neq 0$, then the system is in a 3D QAH state with $C_{k_z=0} = C_{k_z=\pi}$ and $z_{4R} = C_{k_z=0} \bmod 4$.

Because the physical meaning of the double SIs is straightforward (*i.e.* the nontrivial phases are 3D QAH states composed of stacks of Chern insulators), then will not provide explicit layer constructions for double MSG 75.1 $P4$.

If we impose $\mathcal{T}$-symmetry, then the position-space Chern numbers must vanish, which enforces z_{4R} to be zero. Furthermore, if we add $\mathcal{T}$ symmetry to a system that respects double MSG 75.1 $P4$, we specifically find that the SI group becomes trivial.

f. *Double SIs in Type-I Double MSG 77.13 $P4_2$*

The double MSG 77.13 $P4_2$ is generated by $\{E|100\}$, $\{E|010\}$, $\{E|001\}$, and $\{C_{4z}|00\frac{1}{2}\}$.

The SI group of double MSG 77.13 $P4_2$ is $\mathbb{Z}_2$. We can define the SI as half of the Chern number C_0 in the $k_z=0$ plane modulo 2 (where we will show below that C_0 is always even due to the screw symmetry $\{C_{4z}|00\frac{1}{2}\}$):

$$\begin{aligned} z'_{2R} =& \frac{C_0}{2} \bmod 2 \\ =& N_{\text{occ}} - \frac{1}{4}n_\Gamma^{\frac{1}{2}} + \frac{1}{4}n_\Gamma^{-\frac{1}{2}} - \frac{3}{4}n_\Gamma^{\frac{3}{2}} + \frac{3}{4}n_\Gamma^{-\frac{3}{2}} - \frac{1}{4}n_M^{\frac{1}{2}} + \frac{1}{4}n_M^{-\frac{1}{2}} - \frac{3}{4}n_M^{\frac{3}{2}} + \frac{3}{4}n_M^{-\frac{3}{2}} - \frac{1}{2}n_X^{\frac{1}{2}} + \frac{1}{2}n_X^{-\frac{1}{2}} \bmod 2, \end{aligned} \tag{264}$$

where $n_{\Gamma,M}^{\frac{1}{2},-\frac{1}{2},\frac{3}{2},-\frac{3}{2}}$ are the number of occupied states with $\{C_{4z}|00\frac{1}{2}\}$ eigenvalues $e^{-i\frac{\pi}{4}}$, $e^{i\frac{\pi}{4}}$, $e^{-i\frac{3\pi}{4}}$, $e^{i\frac{3\pi}{4}}$, respectively, and $n_X^{\frac{1}{2},-\frac{1}{2}}$ are the number of occupied states with $\{C_{2z}|\mathbf{0}\}$ eigenvalues $e^{-i\frac{\pi}{2}}$, $e^{i\frac{\pi}{2}}$, respectively. For Chern number SIs determined by screw symmetry eigenvalues, we note that we may, in general, either define the SI using the screw eigenvalues in the $k_i=0$ plane or the eigenvalues in the $k_i=\pi$ plane. However, as we will shortly see in the case of double MSG 84.51 $P4_2/m$ in SN 31 i, if a mirror symmetry is also present whose matrix representatives do not commute with those of screw symmetry at all $\mathbf{k}$ points where both symmetries are in the little group $G_{\mathbf{k}}$, then additional constraints are imposed on the small (co)rep characters of screw. Hence, in this work, we will only use screw eigenvalues in the $k_i=0$ plane to define double SIs.

Due to the monodromy of small (co)reps in nonsymmorphic SSGs [see SN 16], the overall sign of each eigenvalue of $\{C_{4z}|00\frac{1}{2}\}$ changes when k_z is advanced through a period of the reciprocal lattice. This implies the compatibility relations: $n_\Gamma^{\frac{1}{2}} = n_\Gamma^{-\frac{3}{2}}$, $n_\Gamma^{-\frac{1}{2}} = n_\Gamma^{\frac{3}{2}}$, $n_M^{\frac{1}{2}} = n_M^{-\frac{3}{2}}$, $n_M^{-\frac{1}{2}} = n_M^{\frac{3}{2}}$. Imposing the compatibility relation constraints on SEq. (263) [and substituting Γ, M, X for Z, A, R, respectively], we find that the Chern number C_0 is always even.

We therefore define $(C_0/2) \bmod 2$ [as opposed to $C_0 \bmod 4$] to be the SI z'_{2R} of double MSG 77.13 $P4_2$. Using $N_{\text{occ}} = n_X^{\frac{1}{2}} + n_X^{-\frac{1}{2}}$, and the compatibility relations, we then simplify SEq. (264) to be:

$$z'_{2R} = -\frac{1}{2}n_\Gamma^{\frac{3}{2}} + \frac{1}{2}n_\Gamma^{-\frac{3}{2}} - \frac{1}{2}n_M^{\frac{3}{2}} + \frac{1}{2}n_M^{-\frac{3}{2}} + \frac{1}{2}n_X^{\frac{1}{2}} - \frac{1}{2}n_X^{-\frac{1}{2}} \bmod 2. \tag{265}$$

If an insulating state has $z'_{2R}=1$, then the state is a 3D QAH phase with $C_0 \bmod 4 = 2$ in the z-direction.

We can also understand the even Chern number from the perspective of layer constructions. Specifically, if a Chern layer is placed in the $z = z_0$ plane, then, because of the $\{C_{4z}|00\frac{1}{2}\}$ symmetry, there must be another Chern layer with the same Chern number in the $z = z_0 + \frac{1}{2}$ plane.

If we impose $\mathcal{T}$ symmetry then the position-space Chern numbers must vanish, which enforces z'_{2R} to be zero.

g. Double SIs in Type-I Double MSG 81.33 $P\bar{4}$

The double MSG 81.33 $P\bar{4}$ is generated by $\{E|100\}$, $\{E|010\}$, $\{E|001\}$, and $\{S_{4z}|\mathbf{0}\}$.

SIs – The double MSG 81.33 $P\bar{4}$ has the SI group $\mathbb{Z}_4 \times \mathbb{Z}_2^2$. We choose the $\mathbb{Z}_4$ SI to be the Chern number in the $k_z = \pi$ plane modulo 4:

$$z_{4S} = C_{k_z=\pi} \bmod 4 = -\frac{1}{2}n_Z^{\frac{1}{2}} + \frac{1}{2}n_Z^{-\frac{1}{2}} - \frac{3}{2}n_Z^{\frac{3}{2}} + \frac{3}{2}n_Z^{-\frac{3}{2}} - \frac{1}{2}n_A^{\frac{1}{2}} + \frac{1}{2}n_A^{-\frac{1}{2}} - \frac{3}{2}n_A^{\frac{3}{2}} + \frac{3}{2}n_A^{-\frac{3}{2}} + n_R^{\frac{1}{2}} - n_R^{-\frac{1}{2}} \bmod 4, \quad (266)$$

where $n_{Z,A}^{\frac{1}{2},-\frac{1}{2},\frac{3}{2},-\frac{3}{2}}$ are the number of occupied states with $\{S_{4z}|\mathbf{0}\}$ eigenvalues $e^{-i\frac{\pi}{4}}$, $e^{i\frac{\pi}{4}}$, $e^{-i\frac{3\pi}{4}}$, $e^{i\frac{3\pi}{4}}$, respectively, and $n_R^{\frac{1}{2},-\frac{1}{2}}$ are the number of occupied states with $\{C_{2z}|\mathbf{0}\}$ eigenvalues $e^{-i\frac{\pi}{2}}$, $e^{i\frac{\pi}{2}}$, respectively. Due to the compatibility relations, the occupied bands in the $k_z = 0, \pi$ planes must have the same $\{C_{2z}|\mathbf{0}\}$ rotation eigenvalues; hence, the Chern numbers $C_{k_z=0}$ and $C_{k_z=\pi}$ have the same parity ($C_{k_z=0} \bmod 2 = C_{k_z=\pi} \bmod 2$). We define the first $\mathbb{Z}_2$ SI to be half of the difference between the Chern numbers in the $k_z = 0, \pi$ planes, taken modulo 2:

$$\begin{aligned}\delta_{2S} =& \frac{C_{k_z=\pi} - C_{k_z=0}}{2} \bmod 2\\ =& -\frac{1}{4}n_Z^{\frac{1}{2}} + \frac{1}{4}n_Z^{-\frac{1}{2}} - \frac{3}{4}n_Z^{\frac{3}{2}} + \frac{3}{4}n_Z^{-\frac{3}{2}} - \frac{1}{4}n_A^{\frac{1}{2}} + \frac{1}{4}n_A^{-\frac{1}{2}} - \frac{3}{4}n_A^{\frac{3}{2}} + \frac{3}{4}n_A^{-\frac{3}{2}} + \frac{1}{2}n_R^{\frac{1}{2}} - \frac{1}{2}n_R^{-\frac{1}{2}}\\ &+ \frac{1}{4}n_\Gamma^{\frac{1}{2}} - \frac{1}{4}n_\Gamma^{-\frac{1}{2}} + \frac{3}{4}n_\Gamma^{\frac{3}{2}} - \frac{3}{4}n_\Gamma^{-\frac{3}{2}} + \frac{1}{4}n_M^{\frac{1}{2}} - \frac{1}{4}n_M^{-\frac{1}{2}} + \frac{3}{4}n_M^{\frac{3}{2}} - \frac{3}{4}n_M^{-\frac{3}{2}} - \frac{1}{2}n_X^{\frac{1}{2}} + \frac{1}{2}n_X^{-\frac{1}{2}} \bmod 2. \end{aligned} \quad (267)$$

Next using the relations:

$$n_{\Gamma,M}^{\frac{1}{2}} + n_{\Gamma,M}^{-\frac{3}{2}} = n_{Z,A}^{\frac{1}{2}} + n_{Z,A}^{-\frac{3}{2}},\ n_{\Gamma,M}^{-\frac{1}{2}} + n_{\Gamma.M}^{\frac{3}{2}} = n_{Z,A}^{-\frac{1}{2}} + n_{Z,A}^{\frac{3}{2}},\ n_X^{\frac{1}{2}} = n_R^{\frac{1}{2}},\ n_X^{-\frac{1}{2}} = n_R^{-\frac{1}{2}}, \quad (268)$$

we simplify δ_{2S}:

$$\delta_{2S} = -n_Z^{\frac{3}{2}} + n_Z^{-\frac{3}{2}} - n_A^{\frac{3}{2}} + n_A^{-\frac{3}{2}} + n_\Gamma^{\frac{3}{2}} - n_\Gamma^{-\frac{3}{2}} + n_M^{\frac{3}{2}} - n_M^{-\frac{3}{2}} \bmod 2. \quad (269)$$

Because of the difference of 2 (modulo 4) in the Chern numbers in the $k_z = 0, \pi$ planes indicated by $\delta_{2S} = 1$, we deduce that $\delta_{2S} = 1$ indicates a WSM with $2 + 4n$ ($n \in \{\mathbb{Z}^+, 0\}$) Weyl points between $k_z = 0$ and $k_z = \pi$. Additionally, the Chern number in the $k_z = 0$ plane (modulo 4) is completely determined by the compatibility relations and the SIs – specifically, $C_{k_z=0} \bmod 4 = z_{4S} - 2\delta_{2S} \bmod 4$. We note that during the preparation of this work, an SI equivalent to δ_{2S} was introduced in SRef. 175 as an intermediate quantity relevant to the high-throughput numerical identification of nonmagnetic solid-state WSMs.

The second $\mathbb{Z}_2$ SI in double MSG 81.33 $P\bar{4}$ is given by:

$$z_2 = \sum_{K=\Gamma,M,Z,A} \frac{n_K^{\frac{1}{2}} - n_K^{-\frac{3}{2}}}{2} \bmod 2. \quad (270)$$

Below, we will show that z_2 is in one-to-one correspondence with the WSM and 3D TI invariant $(z_2)_{P\bar{4}1'}$ in the Type-II double SSG 81.34 $P\bar{4}1'$ generated by adding $\{\mathcal{T}|\mathbf{0}\}$ symmetry to double MSG 81.33 $P\bar{4}$, where $(z_2)_{P\bar{4}1'}$ was previously introduced in SRef. 26. Hence, we will show below that a gapped state with $z_2 = 1$ is compatible with a fourfold-rotoinversion- (S_4) -protected AXI phase if $z_{4S} = \delta_{2S} = 0$.

Layer constructions – We next employ the layer construction method to diagnose the topology of the symmetry-indicated topological insulating phases in double MSG 81.33 $P\bar{4}$, where the double SIs of each layer construction are given in the order $(z_{4S}, \delta_{2S}, z_2)$:

1. A $\hat{\mathbf{z}}$-normal Chern layer with $C_z = 1$ in the $z = 0$ plane can be realized by a 3D insulator whose occupied bands transform in the small irreps $\frac{1}{2}, \frac{3}{2}, -\frac{1}{2}, \frac{1}{2}, \frac{3}{2}$, and $-\frac{1}{2}$ at Γ, M, X, Z, A, and R ($N_{\text{occ}} = 1$), respectively. The SIs of this layer construction are (101).

2. A $\hat{\mathbf{z}}$-normal Chern layer with $C_z = 1$ in the $z = \frac{1}{2}$ plane can be realized by a 3D insulator whose occupied bands transform in the small irreps $\frac{1}{2}, \frac{3}{2}, -\frac{1}{2}, -\frac{3}{2}, -\frac{1}{2}$, and $-\frac{1}{2}$ at Γ, M, X, Z, A, and R ($N_{\text{occ}} = 1$), respectively. The SIs of this layer construction are (100).

Both of the layer constructions are 3D QAH states with $C = 1$ in the z-direction. Because $S_{4z} = \mathcal{I}C_{4z}^{-1}$, and $\mathcal{I}$ leads to an additional minus sign in the occupied $S_{4z} = \mathcal{I}C_{4z}^{-1}$ eigenvalue in the $k_z = \pi$ plane contributed by the layer $z = \frac{1}{2}$ (*i.e.* $e^{-i\frac{2\pi}{4}j} \rightarrow -e^{-i\frac{2\pi}{4}j}$, see SN 31 a), then the $\{S_{4z}|\mathbf{0}\}$ eigenvalues at Z and A in the $z = \frac{1}{2}$ layer construction have opposite signs compared to the occupied $\{S_{4z}|\mathbf{0}\}$ eigenvalues at Γ and M, respectively. We additionally note that the occupied C_{2z} eigenvalues are required to be the same at R and X due to the compatibility relations.

The S_4 $\mathbb{Z}_2$ invariant and axion insulators – When the total Chern number is zero and the bulk is insulating, the axion angle θ is given by $\theta \bmod 2\pi = \pi z_2$, where z_2 is termed the S_4 $\mathbb{Z}_2$ invariant. We may construct an AXI phase by placing a Chern layer with $C_z = 1$ in the $z = 0$ plane and a Chern layer with $C_z = -1$ in the $z = \frac{1}{2}$ plane. The AXI phase has the SIs (001). However, we emphasize that the total Chern number cannot be completely determined by the SIs. For example, the 3D QAH state consisting of a Chern layer with $C_z = 3$ in the $z = 0$ plane and a Chern layer with $C_z = 1$ in the $z = \frac{1}{2}$ plane also has the SIs (001).

Relationship with the SIs in other double SSGs – Double SSG 83.34 $P\bar{4}1'$, which is the double SSG that results from adding $\{\mathcal{T}|\mathbf{0}\}$ symmetry to Type-I double MSG 81.33 $P\bar{4}$ – has the SI group $\mathbb{Z}_2$. The $\mathbb{Z}_2$ double SI in double SSG 83.34 $P\bar{4}1'$ either corresponds to a $\mathcal{T}$-invariant WSM, or to a $\mathcal{T}$-symmetric 3D TI[26]. Consequently, a 3D TI phase in double SSG 83.34 $P\bar{4}1'$ must subduce to an AXI in double MSG 83.33 $P\bar{4}$ if $\{S_{4z}|\mathbf{0}\}$ and primitive lattice translation symmetries are preserved while breaking $\mathcal{T}$, because both insulators share the common nontrivial axion angle $\theta = \pi$. Hence, the double SI subduction relations are given by:

$$(z_2)_{P\bar{4}1'} \rightarrow (z_{4S}, \delta_{2S}, z_2)_{P\bar{4}} = (00, z_2)_{P\bar{4}}. \tag{271}$$

h. Double SIs in Type-I Double MSG 83.43 $P4/m$

The double MSG 83.43 $P4/m$ is generated by $\{E|100\}$, $\{E|010\}$, $\{E|001\}$, $\{C_{4z}|\mathbf{0}\}$, and $\{m_z|\mathbf{0}\}$.

SIs – The double MSG 83.43 $P4/m$ has the SI group $\mathbb{Z}_4^3$. We choose the three $\mathbb{Z}_4$-valued SIs to be:

$$\begin{aligned}\delta_{4m} &= -C^+_{k_z=\pi} + C^-_{k_z=0} \bmod 4 \\ &= -\sum_{K=Z,A}\left(-\frac{1}{2}n_K^{\frac{1}{2},+i} + \frac{1}{2}n_K^{-\frac{1}{2},+i} - \frac{3}{2}n_K^{\frac{3}{2},+i} + \frac{3}{2}n_K^{-\frac{3}{2},+i}\right) + n_R^{\frac{1}{2},+i} - n_R^{-\frac{1}{2},+i} \\ &\quad + \sum_{K=\Gamma,M}\left(-\frac{1}{2}n_K^{\frac{1}{2},-i} + \frac{1}{2}n_K^{-\frac{1}{2},-i} - \frac{3}{2}n_K^{\frac{3}{2},-i} + \frac{3}{2}n_K^{-\frac{3}{2},-i}\right) - n_X^{\frac{1}{2},-i} + n_X^{-\frac{1}{2},-i} \bmod 4,\end{aligned} \tag{272}$$

$$z^+_{4m,\pi} = C^+_{k_z=\pi} \bmod 4 = \sum_{K=Z,A}\left(-\frac{1}{2}n_K^{\frac{1}{2},+i} + \frac{1}{2}n_K^{-\frac{1}{2},+i} - \frac{3}{2}n_K^{\frac{3}{2},+i} + \frac{3}{2}n_K^{-\frac{3}{2},+i}\right) + n_R^{\frac{1}{2},+i} - n_R^{-\frac{1}{2},+i} \bmod 4, \tag{273}$$

$$z^-_{4m,\pi} = C^-_{k_z=\pi} \bmod 4 = \sum_{K=Z,A}\left(-\frac{1}{2}n_K^{\frac{1}{2},-i} + \frac{1}{2}n_K^{-\frac{1}{2},-i} - \frac{3}{2}n_K^{\frac{3}{2},-i} + \frac{3}{2}n_K^{-\frac{3}{2},-i}\right) + n_R^{\frac{1}{2},-i} - n_R^{-\frac{1}{2},-i} \bmod 4, \tag{274}$$

where the $\pm i$ superscripts indicate the signs of the mirror eigenvalues. In SEqs. (272), (273), and (274), we have defined δ_{4m} to be $-C^+_{k_z=\pi} + C^-_{k_z=0}$, rather than $C^+_{k_z=\pi} - C^-_{k_z=0}$, such that the double SI z_8 in double MSG 123.339 $P4/mmm$, which we will shortly define in SN 31 k, is related to δ_{4m} through the subduction relation $\delta_{4m} = z_8 \bmod 4$.

Layer constructions – To diagnose the topology associated to each nontrivial value of the double SIs ($\delta_{4m}, z^+_{4m,\pi}, z^-_{4m,\pi}$), we employ the layer construction method. We denote the Chern number in each mirror sector in the $k_z = 0, \pi$ planes as ($C^+_{k_z=0}, C^-_{k_z=0}, C^+_{k_z=\pi}, C^-_{k_z=\pi}$), respectively. We will also calculate the subduced SIs in the subgroups double MSG 2.4 $P\bar{1}$ and double MSG 81.33 $P\bar{4}$, which we will shortly use to determine the double SI subduction relations. The layer constructions for Type-I double MSG 83.43 $P4/m$ are given by:

1. A $\hat{\mathbf{z}}$-normal layer with $C_z^+ = 1$, $C_z^- = 0$ in the $z = 0$ plane has the mirror sector Chern numbers (1010) and the SIs (310). The subduced subgroup SIs are $(\eta_{4I}, z_{2I,1}, z_{2I,2}, z_{2I,3})_{P\bar{1}} = (2001)_{P\bar{1}}$, $(z_{4S}, \delta_{2S}, z_2)_{P\bar{4}} = (101)_{P\bar{4}}$.

2. A $\hat{\mathbf{z}}$-normal layer with $C_z^+ = 0$, $C_z^- = 1$ in the $z = 0$ plane has the mirror sector Chern numbers (0101) and the SIs (101). The subduced subgroup SIs are $(2001)_{P\bar{1}}$, $(101)_{P\bar{4}}$.

3. A $\hat{\mathbf{z}}$-normal layer with $C_z^+ = 1$, $C_z^- = 0$ in the $z = \frac{1}{2}$ plane has the mirror sector Chern numbers (1001) and the SIs (001). The subduced subgroup SIs are $(0001)_{P\bar{1}}$, $(100)_{P\bar{4}}$.

4. A $\hat{\mathbf{z}}$-normal layer with $C_z^+ = 0$, $C_z^- = 1$ in the $z = \frac{1}{2}$ plane has the mirror sector Chern numbers (0110) and the SIs (010). The subduced subgroup SIs are $(0001)_{P\bar{1}}$, $(100)_{P\bar{4}}$.

We emphasize that Chern insulators whose normal vectors lie in the xy-plane are disallowed by $\{m_z|\mathbf{0}\}$ symmetry.

Relationship with the SIs in other double SSGs – In order to identify the AXI phases, we will subduce the SIs in double MSG 83.43 $P4/m$ onto the SIs in double MSG 2.4 $P\bar{1}$ and double MSG 81.33 $P\bar{4}$. The subduction relations are given by:

$$\left(\delta_{4m}, z_{4m,\pi}^{+}, z_{4m,\pi}^{-}\right)_{P4/m} \to (\eta_{4I}, z_{2I,1}, z_{2I,2}, z_{2I,3})_{P\bar{1}} = \left(2(\delta_{4m} \bmod 2), 0, 0,\ z_{4m,\pi}^{+} + z_{4m,\pi}^{-} \bmod 2\right)_{P\bar{1}}, \tag{275}$$

$$\left(\delta_{4m}, z_{4m,\pi}^{+}, z_{4m,\pi}^{-}\right)_{P4/m} \to (z_{4S}, \delta_{2S}, z_{2})_{P\bar{4}} = \left(z_{4m,\pi}^{+} + z_{4m,\pi}^{-} \bmod 4,\ 0,\ \delta_{4m} \bmod 2\right)_{P\bar{4}}, \tag{276}$$

which imply that $\eta'_{2I} = \frac{1}{2}\eta_{4I} = z_2 = \delta_{4m} \bmod 2$ [see SEqs. (249) and (270)]. In MSG 2.4 $P\bar{1}$ and MSG 81.33 $P\bar{4}$, we previously found in SN 31 a and 31 g that the $\eta'_{2I} = 1$ and $z_2 = 1$ states are AXIs protected by $\{\mathcal{I}|\mathbf{0}\}$ and $\{S_{4z}|\mathbf{0}\}$, respectively (provided that the non-symmetry-indicated net Chern numbers are zero). Hence, the AXI phases in MSG 83.43 $P4/m$ are simultaneously protected by $\{\mathcal{I}|\mathbf{0}\}$ and $\{S_{4z}|\mathbf{0}\}$.

Lastly, we will study the effects of imposing $\mathcal{T}$ symmetry. Adding $\{\mathcal{T}|\mathbf{0}\}$ symmetry to Type-I double MSG 83.43 $P4/m$ generates the Type-II double SSG 83.44 $P4/m1'$, which has the SI group $\mathbb{Z}_8 \times \mathbb{Z}_4 \times \mathbb{Z}_2$. The SIs in double SSG 83.44 $P4/m1'$ are related to the SIs in double MSG 83.43 $P4/m$ through the subduction relations:

$$(z_8, z_{4m,\pi}, z_{2w,1})_{P4/m1'} \to \left(\delta_{4m}, z_{4m,\pi}^{+}, z_{4m,\pi}^{-}\right)_{P4/m} = (z_8 \bmod 4, -z_{4m,\pi}, z_{4m,\pi})_{P4/m}. \tag{277}$$

The subduction relations imply that strong 3D TIs in double SSG 83.44 $P4/m1'$ indicated by odd z_8 and mirror TCIs indicated by z_8 mod 4 and $z_{4m,\pi}$ will continue to exhibit symmetry-indicated nontrivial topology if $\{\mathcal{T}|\mathbf{0}\}$ is broken while preserving the symmetries of double MSG 83.43 $P4/m$. Conversely, the weak TI phases indicated by $z_{2w,1}$ and the rotation-anomaly HOTI indicated by $z_8 = 4$ in double SSG 83.44 $P4/m1'$ no longer exhibit symmetry-indicated stable topology when subduced onto double MSG 83.43 $P4/m$. Specifically, the SIs $(400)_{P4/m1'}$ correspond to either a mirror TCI phase with $C_{k_z=0}^{+} \bmod 4 = 8$ or $C_{k_z=\pi}^{+} \bmod 8 = 4$ or a HOTI with vanishing mirror Chern numbers[26]. The HOTI phase has a gapless top ($\hat{\mathbf{z}}$-normal) surface[36] with $4 + 8n$ ($n \in \{\mathbb{Z}^+, 0\}$) twofold Dirac cones that are locally protected by $\{C_{2z} \times \mathcal{T}|\mathbf{0}\}$ symmetry and are anomalous due to surface and bulk $\{C_{4z}|\mathbf{0}\}$ symmetry (see SN 34 and SRef. 36). The HOTI phase, when cut into a $4/m1'$-symmetric rod geometry, exhibits $4 + 8n$ helical hinge states that are locally protected by $\mathcal{T}$ symmetry and globally protected by $\{C_{4z}|\mathbf{0}\}$ symmetry. If $\mathcal{T}$ symmetry is relaxed, then the HOTI hinge states must become gapped, because there are no side-surface mirror lines to protect helical spectral flow in the absence of $\mathcal{T}$ symmetry in MSG 83.33 $P4/m$ (see SN 33). We leave the finer question of whether any non-symmetry-indicated crystalline topology in MSG 83.33 $P4/m$ is subduced from the $(400)_{P4/m1'}$ HOTI phase in double SSG 83.44 $P4/m1'$ for future works.

i. Double SIs in Type-I Double MSG 84.51 $P4_2/m$

The double MSG 84.51 $P4_2/m$ is generated by $\{E|100\}$, $\{E|010\}$, $\{E|001\}$, $\{C_{4z}|00\frac{1}{2}\}$, and $\{m_z|\mathbf{0}\}$.

SIs – The double MSG 84.51 $P4_2/m$ has the SI group $\mathbb{Z}_4 \times \mathbb{Z}_2$. We define the two SIs to be:

$$\begin{aligned} z^+_{4m,0} =& C^+_{k_z=0} \bmod 4 \\ =& \sum_{K=\Gamma,M}\left(-\frac{1}{2}n_K^{\frac{1}{2},+i}+\frac{1}{2}n_K^{-\frac{1}{2},+i}-\frac{3}{2}n_K^{\frac{3}{2},+i}+\frac{3}{2}n_K^{-\frac{3}{2},+i}\right)+n_X^{\frac{1}{2},+i}-n_X^{-\frac{1}{2},+i} \bmod 4, \end{aligned} \tag{278}$$

$$\delta_{2m} = C^+_{k_z=\pi} - C^-_{k_z=0} \bmod 2, \tag{279}$$

where an explicit formula for δ_{2m} was previously provided in SEq. (254). Because the matrix representatives of $\{C_{4z}|00\frac{1}{2}\}$ and $\{m_z|\mathbf{0}\}$ do not commute in all of the small irreps at the $\mathbf{k}$ points in the $k_z=\pi$ plane at which $\{C_{4z}|00\frac{1}{2}\}$ and $\{m_z|\mathbf{0}\}$ are both elements of the little group, then we cannot determine the mirror sector Chern numbers (modulo 4) in the $k_z=\pi$ plane using $\{C_{4z}|00\frac{1}{2}\}$ eigenvalues. Conversely, because the matrix representatives of $\{C_{2z}|\mathbf{0}\}$ and $\{m_z|\mathbf{0}\}$ *commute* in all of the small irreps at the $\mathbf{k}$ points in the $k_z=\pi$ plane at which $\{C_{2z}|\mathbf{0}\}$ and $\{m_z|\mathbf{0}\}$ are both elements of the little group, then we *can* determine the mirror sector Chern numbers (modulo 2) in the $k_z=\pi$ plane using the occupied $\{C_{2z}|\mathbf{0}\}$ eigenvalues. We thus specifically determine that $\delta_{2m} = C^+_{k_z=\pi} - C^-_{k_z=0} \bmod 2$.

Layer constructions – We find that all of the double SIs in double MSG 84.51 $P4_2/m$ can be realized by layer constructions. Before introducing the layer constructions, we first note that the mirror planes in double MSG 84.51 $P4_2/m$ lie at $z=0,\frac{1}{2}$. However, the $\mathcal{I}$ centers lie in the $z=0,\frac{1}{2}$ planes, whereas, conversely, the S_4 centers lie in the $z=\frac{1}{4},\frac{3}{4}$ planes. For each layer construction, we also compute the subduced SIs in the subgroup MSG 2.4 $P\bar{1}$, which we will shortly use to determine the SI subduction relations. The layer constructions of the double SIs $(z^+_{4m,0},\delta_{2m})$ in double MSG 84.51 $P4_2/m$ are given by:

1. A $\hat{\mathbf{z}}$-normal layer with $C_z^+=1$, $C_z^-=0$ in the $z=0$ plane. Due to the $\{C_{4z}|00\frac{1}{2}\}$ symmetry, there is another $C_z^+=1$, $C_z^-=0$ layer in the $z=\frac{1}{2}$ plane. The mirror sector Chern numbers in momentum space are $(C^+_{k_z=0},C^-_{k_z=0},C^+_{k_z=\pi},C^-_{k_z=\pi})=(2011)$, where the subscripts 0 and π indicate values of k_z. The SIs are (21). The subduced subgroup SIs are $(\eta_{4I},z_{2I,1},z_{2I,2},z_{2I,3})_{P\bar{1}}=(2000)_{P\bar{1}}$, $(z_{4S},\delta_{2S},z_2)_{P\bar{4}}=(200)_{P\bar{4}}$.

2. A $\hat{\mathbf{z}}$-normal layer with $C_z^+=0$, $C_z^-=1$ in the $z=0$ plane. Due to the $\{C_{4z}|00\frac{1}{2}\}$ symmetry, there is another $C_z^+=0$, $C_z^-=1$ layer in the $z=\frac{1}{2}$ plane. The mirror sector Chern numbers in momentum space are $(C^+_{k_z=0},C^-_{k_z=0},C^+_{k_z=\pi},C^-_{k_z=\pi})=(0211)$. The SIs are (01). The subduced subgroup SIs are $(2000)_{P\bar{1}}$, $(200)_{P\bar{4}}$.

3. A $\hat{\mathbf{z}}$-normal layer with $C_z=1$ in the $z=\frac{1}{4}$ plane. Due to the $\{C_{4z}|00\frac{1}{2}\}$ symmetry, there is another $C_z=1$ layer in the $z=\frac{3}{4}$ plane. The mirror sector Chern numbers in momentum space are $(C^+_{k_z=0},C^-_{k_z=0},C^+_{k_z=\pi},C^-_{k_z=\pi})=(1111)$. The SIs are (10). The subduced subgroup SIs are $(0000)_{P\bar{1}}$, $(201)_{P\bar{4}}$.

Relationship with the SIs in other double SSGs – In order to later identify the AXI phases, we subduce the SIs onto double MSG 2.4 $P\bar{1}$ and double MSG 81.33 $P\bar{4}$:

$$(z^+_{4m,0},\delta_{2m})_{P4_2/m} \to (\eta_{4I},z_{2I,1},z_{2I,2},z_{2I,3})_{P\bar{1}} = (2\delta_{2m},000)_{P\bar{1}}, \tag{280}$$

$$(z^+_{4m,0},\delta_{2m})_{P4_2/m} \to (z_{4S},\delta_{2S},z_2)_{P\bar{4}} = (2z_{4m,0}-2\delta_{2m} \bmod 4,\ 0,\ z^+_{4m,0} \bmod 2)_{P\bar{4}}. \tag{281}$$

We next study the effects of imposing $\mathcal{T}$ symmetry. The double SSG 84.52 $P4_2/m1'$ – the SSG generated by adding $\{\mathcal{T}|\mathbf{0}\}$ symmetry to MSG 84.51 $P4_2/m$ – has the SI group $\mathbb{Z}_4\times\mathbb{Z}_2$. The $\mathbb{Z}_4$ SI is the parity index z_4 (*i.e.* the $\mathcal{I}$ z_4 index), and the $\mathbb{Z}_2$ SI is the $\mathcal{I}$ $z_{2w,1}$ index. Hence, the subduction relations are given by:

$$(z_4,z_{2w,1})_{P4_2/m1'} \to (z^+_{4m,0},\delta_{2m})_{P4_2/m} = (z_4, z_4 \bmod 2)_{P4_2/m}, \tag{282}$$

implying that adding $\{\mathcal{T}|\mathbf{0}\}$ to an insulating phase in double MSG 84.51 $P4_2/m$ results in an insulator with the SIs $\delta_{2m}=z_4 \bmod 2$. Furthermore, in an insulator, it is required that $C^+_{k_z=0}+C^-_{k_z=0}=C^+_{k_z=\pi}+C^-_{k_z=\pi}=2C^+_{k_z=\pi}$. $\{\mathcal{T}|\mathbf{0}\}$ further enforces $C^+_{k_z=\pi}=0$, $C^+_{k_z=0}=-C^-_{k_z=0}$, such that $\delta_{2m}=C^+_{k_z=\pi}-C^-_{k_z=0} \bmod 2 = C^+_{k_z=0} \bmod 2 = z_4 \bmod 2$.

Axion insulators – Because the S_4 centers in position space do not coincide with the $\mathcal{I}$ centers in MSG 84.51 $P4_2/m$, then the S_4 invariant $z_2=z^+_{4m,0} \bmod 2$ is free to differ from the $\mathcal{I}$ invariant $\eta'_{2I}=\delta_{2m}$. An AXI phase must

have vanishing position-space Chern numbers, as well as $z_2 = \eta'_{2I} = 1$, due to the definitions $\theta \bmod 2\pi = \pi\eta'_{2I}$ and $\theta \bmod 2\pi = \pi z_2$ (see SN 31 a and 31 g, respectively). Thus, in order to guarantee that the net Chern numbers vanish, we may, for example, only construct an AXI phase with $C = 1$ layers at $z = 0, \frac{1}{2}$ if $C = -1$ layers are additionally placed at $z = \frac{1}{4}, \frac{3}{4}$. In this example of an AXI, the $C = 1$ ($C = -1$) Chern layers occupy the $\mathcal{I}$ (S_4 centers).

j. Double SIs in Type-I Double MSG 88.81 $I4_1/a$

The double MSG 88.81 $I4_1/a$ is generated by $\{E|-\frac{1}{2},\frac{1}{2},\frac{1}{2}\}$, $\{E|\frac{1}{2},-\frac{1}{2},\frac{1}{2}\}$, $\{E|\frac{1}{2},\frac{1}{2},-\frac{1}{2}\}$, $\{C_{4z}|\frac{3}{4}\frac{1}{4}\frac{1}{4}\}$, and $\{\mathcal{I}|\mathbf{0}\}$.

SIs – The double MSG 88.81 $I4_1/a$ has the SI group $\mathbb{Z}_2^2$. As we will explicitly derive later in this section, the first $\mathbb{Z}_2$ SI η'_{2I} is related by subduction to the $\mathcal{I}$ invariant η'_{2I} in double MSG 2.4 $P\bar{1}$ [SEq. (249)]:

$$\eta'_{2I} = \frac{\eta_{4I}}{2} \bmod 2 = \frac{1}{2}n_{\Gamma}^{-} + \frac{1}{2}n_{M}^{-} + \frac{1}{2}n_{X}^{-} + \frac{1}{2}n_{X}^{+} + \frac{3}{2}n_{N}^{-} + \frac{1}{2}n_{N}^{+} \bmod 2. \tag{283}$$

The second $\mathbb{Z}_2$ SI z_2 is related by subduction to the S_4 invariant z_2 in double MSG 81.33 $P\bar{4}$ [SEq. (270)]:

$$z_2 = \frac{n_{\Gamma}^{\frac{1}{2}} - n_{\Gamma}^{-\frac{3}{2}}}{2} + \frac{n_{P}^{\frac{1}{2}} - n_{P}^{-\frac{3}{2}} + n_{P}^{\frac{3}{2}} - n_{P}^{-\frac{1}{2}}}{2}. \tag{284}$$

Layer constructions – The double MSG 88.81 has a body-centered lattice generated by:

$$\mathbf{a}_1 = (-\frac{1}{2},\frac{1}{2},\frac{1}{2}), \qquad \mathbf{a}_2 = (\frac{1}{2},-\frac{1}{2},\frac{1}{2}), \qquad \mathbf{a}_3 = (\frac{1}{2},\frac{1}{2},-\frac{1}{2}). \tag{285}$$

There are two types of maximal Wyckoff positions: $\mathcal{I}$ centers:

$$8c: \qquad (0,0,0), \qquad (\frac{1}{2},0,\frac{1}{2}), \qquad (\frac{3}{4},\frac{1}{4},\frac{1}{4}), \qquad (\frac{3}{4},\frac{3}{4},\frac{3}{4}), \tag{286}$$

$$8d: \qquad (0,0,\frac{1}{2}), \qquad (\frac{1}{2},0,0), \qquad (\frac{3}{4},\frac{1}{4},\frac{3}{4}), \qquad (\frac{3}{4},\frac{3}{4},\frac{1}{4}), \tag{287}$$

and S_4 ($\{S_{4z}|\frac{1}{4}\frac{3}{4}\frac{3}{4}\} = \{C_{4z}|\frac{1}{4}\frac{3}{4}\frac{3}{4}\}\{\mathcal{I}|\mathbf{0}\}$) centers:

$$4a: \qquad (0,\frac{1}{4},\frac{1}{8}), \qquad (\frac{1}{2},\frac{1}{4},\frac{3}{8}), \tag{288}$$

$$4b: \qquad (0,\frac{1}{4},\frac{5}{8}), \qquad (\frac{1}{2},\frac{1}{4},\frac{7}{8}), \tag{289}$$

using the notation of the MWYCKPOS tool on the BCS[15–18], and where all coordinates are given in the conventional cell. We consider the following layer constructions:

1. A $\hat{\mathbf{z}}$-normal Chern layer with $C_z = 1$ in the $z = 0$ plane. The screw symmetry operation additionally generates Chern layers with $C_z = 1$ in the $z = \frac{1}{4}, \frac{1}{2}, \frac{3}{4} \cdots$ planes. All of the $\mathcal{I}$ centers are occupied, all of the S_4 centers are unoccupied, and the total Chern number in each unit cell is $C_z = 2$, such that $\eta'_{2I} = 1$, $z_2 = 0$.

2. A $\hat{\mathbf{z}}$-normal Chern layer with $C_z = 1$ in the $z = \frac{1}{8}$ plane. The screw symmetry operation additionally generates Chern layers with $C_z = 1$ in the $z = \frac{3}{8}, \frac{5}{8}, \frac{7}{8} \cdots$ planes. All of the $\mathcal{I}$ centers are unoccupied, all of the S_4 centers are occupied, and the total Chern number in each unit cell is $C_z = 2$, such that $\eta'_{2I} = 0$, $z_2 = 1$.

Axion insulators – Because the S_4 centers do not coincide with the $\mathcal{I}$ centers in position space in double MSG 88.81 $I4_1/a$, then the S_4 invariant z_2 is free to differ from the $\mathcal{I}$ invariant η'_{2I}. An AXI phase must have vanishing position-space Chern numbers, as well as $z_2 = \eta'_{2I} = 1$, due to the definitions $\theta \bmod 2\pi = \pi\eta'_{2I}$ and $\theta \bmod 2\pi = \pi z_2$ (see SN 31 a and 31 g, respectively). Hence, to generate an AXI with vanishing position-space Chern numbers by placing $C = 1$ layers at $z = 0, \frac{1}{4}, \frac{1}{2}, \frac{3}{4}$, we must also place $C = -1$ layers at $z = \frac{1}{8}, \frac{3}{8}, \frac{5}{8}, \frac{7}{8}$, such that the Chern layers with $C = 1$ ($C = -1$) occupy the $\mathcal{I}$ (S_4) centers.

Relationship with the SIs in other double SSGs – We will now study the effects of imposing $\mathcal{T}$ symmetry. The double SSG 88.82 $I4_1/a1'$ – which is generated by adding $\{\mathcal{T}|\mathbf{0}\}$ to Type-I double MSG 88.81 $I4_1/a$ – has the SI

group $\mathbb{Z}_4$. The subduction relations for the double SIs are given by:

$$(z_4)_{I4_1/a1'} \to (\eta'_{2I}, z_2)_{I4_1/a} = (z_4 \bmod 2, z_4 \bmod 2)_{I4_1/a}. \tag{290}$$

Hence, a symmetry-indicated 3D TI in $I4_1/a1'$ will necessarily become an $\mathcal{I}$- or S_4-protected AXI if $\mathcal{T}$ symmetry is relaxed while preserving the symmetries of MSG 88.81 $I4_1/a$, because infinitesimal $\mathcal{T}$-breaking in a 3D insulator cannot change the momentum-space Chern numbers of the occupied bands in any 2D BZ plane.

Subduction of η'_{2I} onto double MSG 2.4 $P\bar{1}$ – In MSG 88.81 $I4_1/a$, the reciprocal lattice is generated by:

$$\mathbf{b}_1 = (0, 2\pi, 2\pi), \qquad \mathbf{b}_2 = (2\pi, 0, 2\pi), \qquad \mathbf{b}_3 = (2\pi, 2\pi, 0). \tag{291}$$

There are four inequivalent, $\mathcal{I}$-invariant momenta:

$$\Gamma(0,0,0), \qquad M(2\pi,0,0), \qquad X(\pi,\pi,0), \qquad N(\pi,0,\pi), \tag{292}$$

where the equivalence between $\mathbf{k}$ points is defined in SEq. (48) and the surrounding text, and where the coordinates of Γ, M, X, and N in SEq. (292) are given in the conventional cell.

The star of X has two arms – $X_1(\pi,\pi,0)$ and $X_2(\pi,-\pi,0)$, which are related by the screw operation $\{C_{4z}|\frac{3}{4}\frac{1}{4}\frac{1}{4}\}$. If $|\psi_{X_1}\rangle$ is a Bloch state at X_1, then $|\psi_{X_2}\rangle = \{C_{4z}|\frac{3}{4}\frac{1}{4}\frac{1}{4}\}|\psi_{X_1}\rangle$ is a state at X_2. Taking $|\psi_{X_1}\rangle$ to have the parity ($\mathcal{I}$) eigenvalue ξ, we will now determine the parity eigenvalue of $|\psi_{X_2}\rangle$. Because:

$$\{\mathcal{I}|\mathbf{0}\}\{C_{4z}|\frac{3}{4}\frac{1}{4}\frac{1}{4}\}\{\mathcal{I}|\mathbf{0}\}^{-1} = \{E| -\frac{3}{2}, -\frac{1}{2}, -\frac{1}{2}\}\{C_{4z}|\frac{3}{4}\frac{1}{4}\frac{1}{4}\}, \tag{293}$$

then:

$$\{\mathcal{I}|\mathbf{0}\}|\psi_{X_2}\rangle = \{E| -\frac{3}{2}, -\frac{1}{2}, -\frac{1}{2}\}\xi|\psi_{X_2}\rangle = -\xi|\psi_{X_2}\rangle. \tag{294}$$

Hence, taking the parity eigenvalue of $|\psi_{X_1}\rangle$ to be ξ, the parity eigenvalue of $|\psi_{X_2}\rangle$ is $-\xi$.

Next, the star of N has four arms: $N_1(\pi,0,\pi)$, $N_2(0,\pi,\pi)$, $N_3(-\pi,0,\pi)$, and $N_4(0,-\pi,\pi)$, which are related to N_1 by the operations, $\{E|\mathbf{0}\}$, $\{C_{4z}|\frac{3}{4}\frac{1}{4}\frac{1}{4}\}$, $\{C_{2z}|\frac{1}{2}0\frac{1}{2}\}$, and $\{C_{4z}^{-1}|\frac{3}{4}\frac{3}{4}\frac{3}{4}\}$, respectively. Because:

$$\{\mathcal{I}|\mathbf{0}\}\{C_{4z}|\frac{3}{4}\frac{1}{4}\frac{1}{4}\}\{\mathcal{I}|\mathbf{0}\}^{-1} = \{E| -\frac{3}{2}, -\frac{1}{2}, -\frac{1}{2}\}\{C_{4z}|\frac{3}{4}\frac{1}{4}\frac{1}{4}\}, \tag{295}$$

$$\{\mathcal{I}|\mathbf{0}\}\{C_{2z}|\frac{1}{2}0\frac{1}{2}\}\{\mathcal{I}|\mathbf{0}\}^{-1} = \{E| -1, 0, -1\}\{C_{2z}|\frac{1}{2}0\frac{1}{2}\}, \tag{296}$$

$$\{\mathcal{I}|\mathbf{0}\}\{C_{4z}^{-1}|\frac{3}{4}\frac{3}{4}\frac{3}{4}\}\{\mathcal{I}|\mathbf{0}\}^{-1} = \{E| -\frac{3}{2}, -\frac{3}{2}, -\frac{3}{2}\}\{C_{4z}^{-1}|\frac{3}{4}\frac{3}{4}\frac{3}{4}\}, \tag{297}$$

then the extra phase factor in the SI for the occupied parity eigenvalue at $\mathbf{k}_{N_\alpha}$ is given by $e^{-i\mathbf{t}_\alpha\cdot\mathbf{k}_{N_\alpha}}$ ($\alpha = 2,3,4$), where $\mathbf{t}_\alpha$ is the extra translation determined above, and where $\mathbf{k}_{N_\alpha}$ is the momentum N_α. The parity SI phases at N_2, N_3, and N_4 are thus -1, 1, and 1, respectively.

To determine the $\mathcal{I}$ double SI η_{4I}, we apply SEq. (235) to the parity eigenvalue multiplicities at the eight $\mathcal{I}$-invariant momenta Γ, M, $X_{1,2}$, and $N_{1,2,3,4}$, respectively:

$$\eta_{4I} = n_\Gamma^- + n_M^- + n_X^- + n_X^+ + 3n_N^- + n_N^+ \bmod 4. \tag{298}$$

We find that the parity eigenvalues enforce that $\eta_{4I} \bmod 2 = 0$. Hence, the $\mathcal{I}$ double SI in MSG 88.81 $I4_1/a$ is $\eta'_{2I} = \frac{1}{2}\eta_{4I}$ [SEq. (249)].

Subduction of z_2 onto double MSG 81.33 $P\bar{4}$ – There are three inequivalent S_4-invariant momenta:

$$\Gamma(0,0,0), \qquad M(2\pi,0,0), \qquad P(\pi,\pi,\pi). \tag{299}$$

First, the star of P has two arms – $P_1(\pi,\pi,\pi)$ and $P_2(-\pi,-\pi,-\pi)$, which are related by $\mathcal{I}$. Because,

$$\{S_{4z}|\frac{1}{4}\frac{3}{4}\frac{3}{4}\}\{\mathcal{I}|\mathbf{0}\}\{S_{4z}|\frac{1}{4}\frac{3}{4}\frac{3}{4}\}^{-1} = \{E|\frac{1}{2}, \frac{3}{2}, \frac{3}{2}\}\{\mathcal{I}|\mathbf{0}\}, \tag{300}$$

and:

$$\exp\left(i\left(\frac{1}{2},\frac{3}{2},\frac{3}{2}\right)\cdot(-\pi,-\pi,-\pi)\right)=\exp\left(i\frac{\pi}{2}\right), \tag{301}$$

then, if P_1 has a Bloch state with the $\{S_{4z}|\frac{1}{4},\frac{3}{4},\frac{3}{4}\}$ eigenvalue $e^{-i\frac{\pi}{2}j}$, P_2 is required to have a Bloch state with the $\{S_{4z}|\frac{1}{4},\frac{3}{4},\frac{3}{4}\}$ eigenvalue $e^{-i\frac{\pi}{2}(j-1)}$. We thus conclude that:

$$n_{P_1}^{j}=n_{P_2}^{j-1}. \tag{302}$$

To determine the S_4 double SI z_2, we apply SEq. (270) to the $\{S_4|\frac{1}{4}\frac{3}{4}\frac{3}{4}\}$ eigenvalue multiplicities at the four $\{S_4|\frac{1}{4}\frac{3}{4}\frac{3}{4}\}$-invariant momenta Γ, M, and $P_{1,2}$:

$$z_2=\frac{n_\Gamma^{\frac{1}{2}}-n_\Gamma^{-\frac{3}{2}}}{2}+\frac{n_M^{\frac{1}{2}}-n_M^{-\frac{3}{2}}}{2}+\frac{n_P^{\frac{1}{2}}-n_P^{-\frac{3}{2}}+n_P^{\frac{3}{2}}-n_P^{-\frac{1}{2}}}{2} \bmod 2. \tag{303}$$

Using the Corepresentations, MCOMPREL, and MBANDREP tools on the BCS introduced in this work (see SN 13, 16, and 23, respectively), we find that $n_M^{\frac{1}{2}}=n_M^{-\frac{3}{2}}$ is required in any insulating state in double MSG 88.81 $I4_1/a$. Hence, the factor of $\frac{n_M^{\frac{1}{2}}-n_M^{-\frac{3}{2}}}{2}$ can be omitted in SEq. (303), leading to a final expression:

$$z_2=\frac{n_\Gamma^{\frac{1}{2}}-n_\Gamma^{-\frac{3}{2}}}{2}+\frac{n_P^{\frac{1}{2}}-n_P^{-\frac{3}{2}}+n_P^{\frac{3}{2}}-n_P^{-\frac{1}{2}}}{2} \bmod 2. \tag{304}$$

k. Double SIs in Type-I Double MSG 123.339 $P4/mmm$

The double MSG 123.339 $P4/mmm$ is generated by $\{E|100\}$, $\{E|010\}$, $\{E|001\}$, $\{C_{4z}|\mathbf{0}\}$, $\{m_x|\mathbf{0}\}$, and $\{\mathcal{I}|\mathbf{0}\}$.

SIs – The double MSG 123.339 $P4/mmm$ has the SI group $\mathbb{Z}_8\times\mathbb{Z}_4\times\mathbb{Z}_2$. In double-valued small irreps of the little groups at the $\mathcal{I}$-invariant $\mathbf{k}$ points, the matrix representatives of perpendicular mirror symmetries (*e.g.* $\{m_x|\mathbf{0}\}$ and $\{m_y|\mathbf{0}\}$) anticommute. Hence, Bloch states at the eight $\mathcal{I}$-invariant momenta must be at least twofold degenerate (and in fact are exactly twofold degenerate in double MSG 123.339 $P4/mmm$). The double SIs can be chosen to be the same as the double SIs of SSG 123.340 $P4/mmm1'$ (previously introduced in SRefs. 25–27), because the addition of $\mathcal{T}$ symmetry to double MSG 123.339 $P4/mmm$ does not change the dimensions and characters of the small irreps at the high-symmetry BZ points or the compatibility relations between the high-symmetry-point small irreps. In the physical basis, the $\mathbb{Z}_8$ double SI is:

$$z_8=\frac{3}{2}n^{\frac{3}{2},+}-\frac{3}{2}n^{\frac{3}{2},-}-\frac{1}{2}n^{\frac{1}{2},+}+\frac{1}{2}n^{\frac{1}{2},-} \bmod 8, \tag{305}$$

$$n^{j,\pm}=\sum_{K=\Gamma,M,Z,A}n_K^{j,\pm}+\sum_{K=X,R}n_K^{\frac{1}{2},\pm}, \tag{306}$$

where $n_K^{j,\pm}$ is the number of states with parity ($\mathcal{I}$) eigenvalue ±1 and angular momentum j (modulo 4) at the momentum K, which corresponds to the $\{C_{4z}|\mathbf{0}\}$ eigenvalue $e^{-i\frac{2\pi}{4}j}$ at $K=\Gamma,M,Z,A$, and the $\{C_{2z}|\mathbf{0}\}$ eigenvalue $e^{-i\frac{\pi}{2}j}$ at $K=X,R$. The $\mathbb{Z}_4$ SI is $z_{4m,\pi}^{-}$, which indicates the Chern number in the negative mirror sector in the $k_z=\pi$ plane $z_{4m,\pi}^{-}$, and is related by subduction to the same SI ($z_{4m,\pi}^{-}$) in double MSG 83.43 $P4/m$ [SEq. (274)]. The $\mathbb{Z}_2$ SI corresponds to the weak TCI $\mathcal{I}$ invariant $z_{2w,1}$ for the mirror Chern number (modulo 2) in the $k_{x,y}=\pi$ planes, and is related by subduction to the same SI ($z_{2w,1}$) in double MSG 47.249 $Pmmm$ [SEq. (260)].

Layer constructions – To diagnose the topology associated to each nontrivial value of the double SIs, we employ the layer construction method. In the layer constructions below, $C^+=-C^-$ due to the net-zero Chern numbers enforced by the mirror symmetries. Hence, we will omit C^- in further discussions of the topology in double MSG 123.339 $P4/mmm$. The layer constructions for the double SIs $(z_8, z_{4m,\pi}^{+}, z_{2w,1})$ in MSG 123.339 $P4/mmm$ are given by:

1. A $\hat{\mathbf{z}}$-normal layer with $C_z^+=1$ in the $z=0$ plane has the SIs (230).

2. A $\hat{\mathbf{z}}$-normal layer with $C_z^+=1$ in the $z=\frac{1}{2}$ plane has the SIs (010).

3. An $\hat{\mathbf{x}}$-normal layer with $C_x^+ = 1$ in the $x = 0$ plane has the SIs (401). We emphasize that, in this layer construction, there is also a superposed $\hat{\mathbf{y}}$-normal layer with $C_y^+ = 1$ in the $y = 0$ plane implied by the $\{C_{4z}|\mathbf{0}\}$ rotation symmetry.

4. An $\hat{\mathbf{x}}$-normal layer with $C_x^+ = 1$ in the $x = \frac{1}{2}$ plane has the SIs (001). We emphasize that, in this layer construction, there is also a superposed $\hat{\mathbf{y}}$-normal layer with $C_y^+ = 1$ in the $y = \frac{1}{2}$ plane implied by the $\{C_{4z}|\mathbf{0}\}$ rotation symmetry.

5. An $(\hat{\mathbf{x}}+\hat{\mathbf{y}})$-normal layer with $C_{x+y}^+ = 1$ in the $x+y = 0$ plane has the SIs (400). We emphasize that, in this layer construction, there is also a superposed $(\hat{\mathbf{x}}-\hat{\mathbf{y}})$-normal layer with $C_{x-y}^+ = 1$ in the $x-y = 0$ plane implied by the $\{C_{4z}|\mathbf{0}\}$ rotation symmetry.

Axion insulators – We find that states with odd z_8 SIs cannot be constructed from layers of 2D stable topological phases. However, we may still use subduction relations to determine the bulk topology of insulators with odd values of z_8. First, as we will show below, (100), (300), (500), and (700) subduce to $(2000)_{P\bar{1}}$ in MSG 2.4 $P\bar{1}$. Hence, if the (100), (300), (500), and (700) phases in double MSG 123.339 $P4/mmm$ are insulating, then the bulk insulator must either be an AXI or a 3D QAH state. Because the net Chern numbers $C_{x,y,z} = 0$ must vanish if the bulk is gapped, due to the mirror symmetries of MSG 123.339 $P4/mmm$, then the (100), (300), (500), and (700) states must be AXIs. This result can also be understood by subducing from a $\mathcal{T}$-symmetric SSG. Specifically, because (100), (300), (500), and (700) in double MSG 123.339 $P4/mmm$ can respectively be subduced from $(100)_{P4/mmm1'}$, $(300)_{P4/mmm1'}$, $(500)_{P4/mmm1'}$, and $(700)_{P4/mmm1'}$ in Type-II SG 123.340 $P4/mmm1'$, which correspond to $\mathcal{T}$-symmetric 3D TIs with $\theta = \pi$[24,26,27], then (100), (300), (500), and (700) are compatible with bulk-gapped states. Hence, we conclude that 3D insulators with (100), (300), (500), and (700) in double MSG 123.349 $P4/mmm$ are AXIs, *without ambiguity*. We conjecture that (100), (300), (500), and (700) AXIs in double MSG 123.349 $P4/mmm$ can be constructed using the topological crystal method[168], which additionally incorporates cell complexes of 2D Chern insulators, TIs, and TCIs.

Helical HOTI phases protected by mirror and C_4 rotation symmetry – First, the double SIs $(400)_{P4/mmm1'}$ of Type-II double SSG 123.340 $P4/mmm1'$ either correspond to a rotation-anomaly (non-axionic, *i.e.* $\theta \bmod 2\pi = 0$) HOTI protected by C_4 and $\mathcal{T}$ symmetries, or a mirror TCI with $C_{m_z} \bmod 8 = 4$ (*c.f.* Table 7 in the Supplementary Material of SRef. 26). In the C_4- and $\mathcal{T}$-symmetric HOTI phase, there are $4+8n$ ($n \in \{\mathbb{Z}^+, 0\}$) helical hinge modes on a z-directed, C_4- and $\mathcal{T}$-symmetric rod, and $4+8n$ twofold Dirac points on the top ($\hat{\mathbf{z}}$-normal) rod surface that are locally protected by mirror symmetry (see SN 33). Because double SSG 123.340 $P4/mmm1'$ contains $\{m_{x,y}|\mathbf{0}\}$ symmetries, then four of the helical hinge modes on the boundary of a $4/mmm1'$-symmetric sample must also be pinned to the hinge projections of bulk mirror planes whose normal vectors lie in the xy-plane, and must be indicated by bulk mirror Chern numbers. Hence, when $\mathcal{T}$ symmetry is relaxed in a fourfold rotation-anomaly $(400)_{P4/mmm1'}$ HOTI phase in Type-II double SSG 123.340 $P4/mmm1'$ while preserving the symmetries of MSG 123.339 $P4/mmm$, the surface and hinge states will remain gapless and anomalous [see SFig. 21(b) and SN 35].

We will next prove that there are $4+8n$ twofold Dirac points on the top surface of a $4/mmm$-symmetric nanorod of the (400) fourfold rotation-anomaly magnetic HOTI phase in double MSG 123.339 $P4/mmm$ introduced in this work. We denote the five layer constructions as L_a ($a = 1\cdots5$), respectively. The fourfold rotation-anomaly HOTI phase can be constructed as $(2n+1)L_3 \oplus (2m+1)L_4$, or $(2n+1)L_5$, or through any superposition of odd number of the aforementioned layer constructions. Adding $4n'L_1$ or $4m'L_2$, which have the SIs (000), to the layer-constructed HOTI phase will not change the top surface spectrum, because L_1 and L_2 consist of horizontal (*i.e.* $\hat{\mathbf{z}}$-normal) layers, and hence only contribute surface and hinge states on boundaries whose normal vectors lie in the xy-plane.

We will thus focus on the top surface spectra of the $(2n+1)L_3 \oplus (2m+1)L_4$ and $(2n+1)L_5$ layer constructions. We first consider $(2n+1)L_3 \oplus (2m+1)L_4$. The Chern numbers in the m_x mirror sectors are $C^+_{k_x=0} = -C^-_{k_x=0} = 2n+2m+2$, $C^+_{k_x=\pi} = -C^-_{k_x=\pi} = 2n-2m$. Due to the C_4 symmetry, the Chern numbers in the m_y mirror sectors are $C^+_{k_y=0} = -C^-_{k_x=0} = 2n+2m+2$, $C^+_{k_y=\pi} = -C^-_{k_x=\pi} = 2n-2m$. In the 2D top surface BZ, $C^+_{k_x=0}$ ($C^+_{k_y=0}$) mandates the presence of $|2n+2m+2|$ twofold Dirac points on the $k_x = 0$ ($k_y = 0$) line, and $C^+_{k_x=\pi}$ ($C^+_{k_y=\pi}$) mandates the presence of $|2n-2m|$ twofold Dirac points on the $k_x = \pi$ ($k_y = \pi$) line. Hence, the total number of Dirac points is $2|2n+2m+2| + 2|2n-2m| \bmod 8 = 4$.

Lastly, we consider the layer construction $(2n+1)L_5$. As shown in Supplementary Note 5 in SRef. 26 and in Table 6 of the Supplementary Material of SRef. 26, the mirror sector Chern numbers are given by $C^+_{k_x+k_y=0} = -C^-_{k_x+k_y=0} = 4n+2$, $C^+_{k_x-k_y=0} = -C^-_{k_x-k_y=0} = 4n+2$, and $C^+_{k_x\pm k_y=\pi} = -C^-_{k_x\pm k_y=\pi} = 0$. To understand this result, one can enlarge the unit cell to a supercell with the lattice vectors $(1,1,0)$ and $(1,-1,0)$. We emphasize that mirror (sector) Chern numbers do not change upon enlarging the unit cell if the number of layers per cell does not change; hence we can compute the mirror sector Chern numbers in the supercell. We define $x' = \frac{1}{2}x + \frac{1}{2}y$, $y' = \frac{1}{2}x - \frac{1}{2}y$, and correspondingly define $k'_x = k_x + k_y$, $k'_y = k_x - k_y$. As shown in SN 31 a, the Chern numbers of the layers at $x' = 0$ ($y' = 0$) and

$x' = \frac{1}{2}$ ($y' = \frac{1}{2}$) contribute with the same signs towards $C^{+}_{k'_x=0} = -C^{-}_{k'_x=0}$ ($C^{+}_{k'_y=0} = -C^{-}_{k'_y=0}$) and with opposite signs towards $C^{+}_{k'_x=\pi} = -C^{-}_{k'_x=\pi}$ ($C^{+}_{k'_y=\pi} = -C^{-}_{k'_y=\pi}$). Hence, $C^{+}_{k'_x=0} = -C^{-}_{k'_x=0} = 4n+2$, $C^{+}_{k'_x=\pi} = -C^{-}_{k'_x=\pi} = 0$, $C^{+}_{k'_y=0} = -C^{-}_{k'_y=0} = 4n+2$, and $C^{+}_{k'_y=\pi} = -C^{-}_{k'_y=\pi} = 0$. In the 2D top surface BZ, $C^{+}_{k_x+k_y=0}$ ($C^{+}_{k_x-k_y=0}$) mandates the presence of $|4n+2|$ Dirac points on the $k_x+k_y=0$ ($k_x-k_y=0$) line. We additionally note that the mirror sector Chern numbers $C^{+} = -C^{-}$ mandate the presence of $|C^{+}|$ twofold Dirac points on the surface, as shown in SFig. 20. In summary, the total number of top-surface twofold Dirac points in the first surface BZ is $2|4n+2| \bmod 8 = 4$.

In SN 34, we will prove that, on the top surface of the (400) HOTI state – which respects the symmetries of Type-I double magnetic wallpaper group[36,39,73–75] $p4m$ – the presence of $4+8n$ ($n \in \{\mathbb{Z}^{+}, 0\}$) twofold surface Dirac points circumvents the fermion multiplication theorem for 2D lattices with double magnetic wallpaper group $p4m$.

Relationship with the SIs in other double SSGs – To identify the AXI phases, we subduce the SIs onto double MSG 2.4 $P\bar{1}$:

$$(z_8, z^{+}_{4m,\pi}, z_{2w,1})_{P4/mmm} \rightarrow (\eta_{4I}, z_{2I,1}, z_{2I,2}, z_{2I,3})_{P\bar{1}} = (2(z_8 \bmod 2), 000)_{P\bar{1}}. \tag{307}$$

Because the AXI $\mathcal{I}$ SI $\eta_{2I'} = \frac{1}{2}\eta_{4I} = z_8 \bmod 2$ [SEq. (249)], then we conclude that insulators with odd z_8 SIs in double MSG 123.339 $P4/mmm$ are AXIs.

l. Double SIs in Type-I Double MSG 143.1 $P3$

The double MSG 143.1 $P3$ is generated by $\{E|100\}$, $\{E|010\}$, $\{E|001\}$, and $\{C_{3z}|\mathbf{0}\}$, where the angle between the $\{E|100\}$ and $\{E|010\}$ translations is chosen to be $2\pi/3$ for consistency with the $\{C_{3z}|\mathbf{0}\}$ rotation symmetry.

The double MSG 143.1 $P3$ has the SI group $\mathbb{Z}_3$. To determine the physical basis for the double SIs, we first recall the formula for the 2D Chern number in the presence of threefold rotation symmetry[164]:

$$e^{i\frac{2\pi}{3}C} = (-1)^{N_{\text{occ}}} \prod_{n\in\text{occ}} \theta_n(\Gamma)\theta_n(\mathcal{K})\theta_n(\mathcal{KA}), \tag{308}$$

where $\theta_n(\Gamma, \mathcal{K}, \mathcal{KA})$ is the $\{C_{3z}|\mathbf{0}\}$ eigenvalue of the nth occupied state at the corresponding momentum (where it is important to distinguish the $\{C_{3z}|\mathbf{0}\}$ eigenvalues θ_n from the axion angle θ). We can define the SI as the Chern number in the $k_z = \pi$ plane modulo 3:

$$z_{3R} = C_{k_z=\pi} \bmod 3 = \frac{3}{2}N_{\text{occ}} + \sum_{K=A,H,HA}\left(-\frac{1}{2}n_K^{\frac{1}{2}} + \frac{1}{2}n_K^{-\frac{1}{2}} + \frac{3}{2}n_K^{\frac{3}{2}}\right) \bmod 3, \tag{309}$$

where the superscripts $j = -\frac{1}{2}, \frac{1}{2}, \frac{3}{2}$ represent the $\{C_{3z}|\mathbf{0}\}$ eigenvalues $e^{-i\frac{2\pi}{3}j} = e^{-i\frac{\pi}{3}}, e^{i\frac{\pi}{3}}, -1$, respectively, and N_{occ} is the number of occupied bands. Because $\frac{3}{2}N_{\text{occ}} = \sum_{K=A,H,HA} \frac{1}{2}n_K^{-\frac{1}{2}} + \frac{1}{2}n_K^{\frac{1}{2}} + \frac{1}{2}n_K^{\frac{3}{2}}$, then SEq. (309) can be simplified:

$$z_{3R} = \sum_{K=A,H,HA}\left(n_K^{-\frac{1}{2}} - n_K^{\frac{3}{2}}\right) \bmod 3. \tag{310}$$

Due to the compatibility relations and the fact that a chiral fermion in 3D occurs when there is a change in a momentum-space Chern number, a 3D insulator must satisfy $C_{k_z=\pi} = C_{k_z}$ for all k_z. Hence, we may have equivalently defined the SI z_{3R} using the occupied $\{C_{3z}|\mathbf{0}\}$ eigenvalues in $k_z = 0$ plane, or in any other BZ plane of constant k_z. To summarize, if a 3D system is insulating and exhibits $z_{3R} \neq 0$, then the system is in a 3D QAH state with $C_{k_z=0} = C_{k_z=\pi}$ and $z_{3R} = C_{k_z=0} \bmod 3$.

Because the physical meaning of the double SIs is straightforward (*i.e.* the nontrivial phases are 3D QAH states composed of stacks of Chern insulators), then will not provide explicit layer constructions for double MSG 143.1 $P3$.

If we impose $\mathcal{T}$-symmetry, then the position-space Chern numbers must vanish, which enforces z_{3R} to be zero. Furthermore, if we add $\mathcal{T}$ symmetry to a system that respects double MSG 143.1 $P3$, we specifically find that the SI group becomes trivial.

m. Double SIs in Type-I Double MSG 147.13 $P\bar{3}$

The double MSG 147.13 $P\bar{3}$ is generated by $\{E|100\}$, $\{E|010\}$, $\{E|001\}$, $\{C_{3z}|\mathbf{0}\}$, and $\{\mathcal{I}|\mathbf{0}\}$, where the angle between the $\{E|100\}$ and $\{E|010\}$ translations is chosen to be $2\pi/3$ for consistency with the $\{C_{3z}|\mathbf{0}\}$ rotation symmetry.

SIs – The double MSG 147.13 $P\bar{3}$ has the SI group $\mathbb{Z}_{12}\times\mathbb{Z}_2\sim\mathbb{Z}_4\times\mathbb{Z}_3\times\mathbb{Z}_2$. We find that the $\mathbb{Z}_4$, $\mathbb{Z}_3$, and $\mathbb{Z}_2$ double SIs in double MSG 147.13 $P\bar{3}$ all subduce to previously introduced double SIs. First, the $\mathbb{Z}_4$ SI subduces to η_{4I} in double MSG 2.4 $P\bar{1}$ [SEq. (235)], where, as shown in SN 31 a, $\eta_{4I}=1,3$ indicate WSM phases, $\eta_{4I}=2$ indicates that an insulator is either an AXI or in a 3D QAH state, and $\eta_{4I}=0$ indicates that an insulator is either topologically trivial or in a 3D QAH state. The $\mathbb{Z}_3$ SI subduces to z_{3R} in double MSG 143.1 $P3$ [SEq. (310)]. In insulating states, z_{3R} indicates the Chern number modulo 3 in BZ planes of constant k_z. Lastly, the $\mathbb{Z}_2$ SI subduces to $z_{2I,3}$ in double MSG 2.4 $P\bar{1}$ [SEq. (236)], where $z_{2I,3}$ indicates the Chern number modulo 2 in the $k_z=\pi$ plane. In summary, the double SIs in double MSG 147.13 $P\bar{3}$ in the physical basis are given by the previously-defined double SIs $(\eta_{4I},z_{3R},z_{2I,3})$.

Layer constructions – In Cartesian coordinates (x,y,z), the primitive lattice translation vectors in double MSG 147.13 $P\bar{3}$ – $\{E|100\}$, $\{E|010\}$, and $\{E|001\}$ – respectively correspond to $\mathbf{t}_1=(0,-1,0)$, $\mathbf{t}_2=(\frac{\sqrt{3}}{2},\frac{1}{2},0)$, and $\mathbf{t}_3=(0,0,1)$. We consider the following four layer constructions, where the double SIs of each layer construction are given in the order $(\eta_{4I},z_{3R},z_{2I,3})$:

1. A $\hat{\mathbf{z}}$-normal Chern layer with $C_z=1$ in the $z=0$ plane has the SIs (211).

2. A $\hat{\mathbf{z}}$-normal Chern layer with $C_z=-2$ in the $z=0$ plane has the SIs (010).

3. A $\hat{\mathbf{z}}$-normal Chern layer with $C_z=1$ in the $z=\frac{1}{2}$ plane has the SIs (011).

4. An $\hat{\mathbf{x}}$-normal Chern layer with $C_x=1$ in the $x=0$ plane has the SIs (200). We emphasize that, in this layer construction, there are also $|C|=1$ Chern layers in the $C_{3z}\hat{\mathbf{x}}$ and $C_{3z}^2\hat{\mathbf{x}}$ directions implied by the $\{C_{3z}|\mathbf{0}\}$ rotation symmetry.

We label the four layer constructions as $L_{1,2,3,4}$, respectively. We note that $-2L_1$ and $-2L_3$ exhibit the same symmetry-indicated topology as L_2, where $-L_1$ $(-L_3)$ has the same construction as L_1 (L_3), except for a sign change in the Chern number $C_z\to -C_z$.

Total Chern number modulo 6 – The Chern number at $k_z=\pi$ can be determined modulo 6:

$$C_{k_z=\pi}\bmod 6=-2z_{3R}+3z_{2I,3}\bmod 6. \tag{311}$$

SEq. (311) takes the same form as the SI introduced in SRef. 164 for the Chern number in a 2D insulator with sixfold rotation symmetry, which occurs because the point group of double MSG 147.13 $P\bar{3}$ (isomorphic to Type-I MPG 17.1.62 $\bar{3}$) exhibits sixfold symmetry generated by C_{3z} and $\mathcal{I}$[9,33]. In general, we find that, if $\eta_{4I}=0,2$, then the Chern number of the occupied bands in the $k_z=0$ plane is the same as the Chern number of the occupied bands in the $k_z=\pi$ plane (modulo 6). Lastly, if $\eta_{4I}=1,3$, then the Chern number of the occupied bands in the $k_z=0$ plane differs from the Chern number of the occupied bands in the $k_z=\pi$ plane by 3 (modulo 6), implying the presence of an odd number of Weyl points in the BZ between $k_z=0,\pi$, in agreement with the odd value of η_{4I} (see SN 31 a).

Relationship with the SIs in other double SSGs – The double SSG 147.14 $P\bar{3}1'$ – which is generated by adding $\{\mathcal{T}|\mathbf{0}\}$ symmetry to double MSG 147.13 $P\bar{3}$ – has the double SI group $\mathbb{Z}_4\times\mathbb{Z}_2$. The SIs in double SSG 147.14 $P\bar{3}1'$ are related to the SIs in double MSG 147.13 $P\bar{3}$ through the subduction relations:

$$(z_4,z_{2w,3})_{P\bar{3}1'}\to(\eta_{4I},z_{3R},z_{2I,3})_{P\bar{3}}=(2(z_4\bmod 2),00)_{P\bar{3}}. \tag{312}$$

n. Double SIs in Type-I Double MSG 168.109 $P6$

The double MSG 168.109 $P6$ is generated by $\{E|100\}$, $\{E|010\}$, $\{E|001\}$, and $\{C_{6z}|\mathbf{0}\}$, where the angle between the $\{E|100\}$ and $\{E|010\}$ translations is chosen to be $2\pi/3$ for consistency with the $\{C_{3z}|\mathbf{0}\}=(\{C_{6z}|\mathbf{0}\})^2$ rotation symmetry.

The double MSG 168.109 $P6$ has the SI group $\mathbb{Z}_6$. To determine the physical basis for the double SIs, we first recall the formula for the 2D Chern number in the presence of sixfold symmetries[164]:

$$e^{i\frac{2\pi}{6}C}=(-1)^{N_{\rm occ}}\prod_{n\in{\rm occ}}\eta_n(\Gamma)\theta_n(\mathcal{K})\zeta_n(M), \tag{313}$$

where $\eta_n(\Gamma)$, $\theta_n(\mathcal{K})$, $\zeta_n(M)$ are the $\{C_6|\mathbf{0}\}$, $\{C_3|\mathbf{0}\}$, and $\{C_2|\mathbf{0}\}$ eigenvalues of the $n^{\rm th}$ occupied state at Γ, $\mathcal{K}$, and

M, respectively. We define the SI as the Chern number in the $k_z = \pi$ plane modulo 6:

$$z_{6R} = C_{k_z=\pi} \bmod 6 = 3N_{\rm occ} - \frac{1}{2}n_A^{\frac{1}{2}} + \frac{1}{2}n_A^{-\frac{1}{2}} - \frac{3}{2}n_A^{\frac{3}{2}} + \frac{3}{2}n_A^{-\frac{3}{2}} - \frac{5}{2}n_A^{\frac{5}{2}} + \frac{5}{2}n_A^{-\frac{5}{2}} - n_H^{\frac{1}{2}} + n_H^{-\frac{1}{2}} + 3n_H^{\frac{3}{2}} - \frac{3}{2}n_L^{\frac{1}{2}} + \frac{3}{2}n_L^{-\frac{1}{2}} \bmod 6$$
$$= -\frac{1}{2}n_A^{\frac{1}{2}} + \frac{1}{2}n_A^{-\frac{1}{2}} - \frac{3}{2}n_A^{\frac{3}{2}} + \frac{3}{2}n_A^{-\frac{3}{2}} - \frac{5}{2}n_A^{\frac{5}{2}} + \frac{5}{2}n_A^{-\frac{5}{2}} - n_H^{\frac{1}{2}} + n_H^{-\frac{1}{2}} + 3n_H^{\frac{3}{2}} + \frac{3}{2}n_L^{\frac{1}{2}} - \frac{3}{2}n_L^{-\frac{1}{2}} \bmod 6, \quad (314)$$

where the superscripts n_A^j represent the $\{C_{6z}|\mathbf{0}\}$ eigenvalues $e^{-i\frac{2\pi}{6}j}$ at A, n_H^j is the number of occupied states with $\{C_{3z}|\mathbf{0}\}$ eigenvalue $e^{-i\frac{2\pi}{3}j}$ at H, and where n_L^j is the number of states with $\{C_{2z}|\mathbf{0}\}$ eigenvalue $e^{-i\frac{\pi}{2}j}$ at L. Due to the compatibility relations and the fact that a chiral fermion in 3D occurs when there is a change in a momentum-space Chern number, a 3D insulator must satisfy $C_{k_z=\pi} = C_{k_z}$ for all k_z. Hence, we may have equivalently defined the SI z_{6R} using the occupied rotation symmetry eigenvalues in the $k_z = 0$ plane, or in any other BZ plane of constant k_z. To summarize, if a 3D system is insulating and exhibits $z_{6R} \neq 0$, then the system is in a 3D QAH state with $C_{k_z=0} = C_{k_z=\pi}$ and $z_{6R} = C_{k_z=0} \bmod 6$.

Because the physical meaning of the double SIs is straightforward (*i.e.* the nontrivial phases are 3D QAH states composed of stacks of Chern insulators), then will not provide explicit layer constructions for double MSG 168.109 $P6$.

If we impose $\mathcal{T}$-symmetry, then the position-space Chern numbers must vanish, which enforces z_{6R} to be zero. Furthermore, if we add $\mathcal{T}$ symmetry to a system that respects double MSG 168.109 $P6$, we specifically find that the SI group becomes trivial.

o. Double SIs in Type-I Double MSG 174.133 $P\bar{6}$

The double MSG 174.133 $P\bar{6}$ is generated by $\{E|100\}$, $\{E|010\}$, $\{E|001\}$, $\{C_{3z}|\mathbf{0}\}$, and $\{m_z|\mathbf{0}\}$, where the angle between the $\{E|100\}$ and $\{E|010\}$ translations is chosen to be $2\pi/3$ for consistency with the $\{C_{3z}|\mathbf{0}\} = (\{C_{6z}|\mathbf{0}\})^2$ rotation symmetry.

SIs – The double MSG 174.133 $P\bar{6}$ has the SI group $\mathbb{Z}_3^3$. In the physical basis, the three $\mathbb{Z}_3$-valued SIs are: $(\delta_{3m}, z^+_{3m,\pi}, z^-_{3m,\pi})$, for which the SI formulas are:

$$\delta_{3m} = C^+_{k_z=\pi} - C^-_{k_z=0} \bmod 3 = \sum_{K=A,H,HA}\left(n_K^{-\frac{1}{2},+i} - n_K^{\frac{3}{2},+i}\right) - \sum_{K=\Gamma,\mathcal{K},\mathcal{KA}}\left(n_K^{-\frac{1}{2},-i} - n_K^{\frac{3}{2},-i}\right) \bmod 3, \quad (315)$$

$$z^+_{3m,\pi} = C^+_{k_z=\pi} \bmod 3 = \sum_{K=A,H,HA}\left(n_K^{-\frac{1}{2},+i} - n_K^{\frac{3}{2},+i}\right) \bmod 3, \quad (316)$$

$$z^-_{3m,\pi} = C^-_{k_z=\pi} \bmod 3 = \sum_{K=A,H,HA}\left(n_K^{-\frac{1}{2},-i} - n_K^{\frac{3}{2},-i}\right) \bmod 3, \quad (317)$$

such that a 3D insulator with $z^+_{3m,\pi} \neq -z^-_{3m,\pi} \bmod 3$ is in a 3D QAH state. The compatibility relations require the $k_z = 0, \pi$ planes to have the same occupied C_{3z} eigenvalues, and hence the same Chern numbers (modulo 3). In an insulating state (*i.e.* in the absence of bulk Weyl points), it is further required that $C^+_{k_z=0} + C^-_{k_z=0} = C^+_{k_z=\pi} + C^-_{k_z=\pi}$ and $C^+_{k_z=0} - C^-_{k_z=\pi} \bmod 3 = C^+_{k_z=\pi} - C^-_{k_z=0} \bmod 3 = \delta_{3m}$. As we will show below, in insulators with net-zero position-space Chern numbers, AXI phases may be stabilized in double MSG 174.133 $P\bar{6}$ by S_6 rotoinversion symmetry, but will not be symmetry indicated, because the strong index δ_{3m} is $\mathbb{Z}_3$-valued, whereas the axion angle θ is $\mathbb{Z}_2$-valued (if quantized).

Layer constructions – To diagnose the topology associated to each nontrivial value of the double SIs $(\delta_{3m}, z^+_{3m,\pi}, z^-_{3m,\pi})$, we employ the layer construction method. In each layer construction, we denote the mirror sector Chern numbers of the occupied bands at $k_z = 0, \pi$ as $(C^+_{k_z=0}, C^-_{k_z=0}, C^+_{k_z=\pi}, C^-_{k_z=\pi})$. The layer constructions for Type-I double MSG 174.133 $P\bar{6}$ are given by:

1. A $\hat{\mathbf{z}}$-normal Chern layer with $C_z^+ = 1$, $C_z^- = 0$ in the $z = 0$ plane has the mirror sector Chern numbers (1010) and the SIs (110).

2. A $\hat{\mathbf{z}}$-normal Chern layer with $C_z^+ = 0$, $C_z^- = 1$ in the $z = 0$ plane has the mirror sector Chern numbers (0101) and the SIs (201).

3. A $\hat{\mathbf{z}}$-normal Chern layer with $C_z^+ = 1$, $C_z^- = 0$ in the $z = \frac{1}{2}$ plane has the mirror sector Chern numbers (1001) and the SIs (001).

4. A $\hat{\mathbf{z}}$-normal Chern layer with $C_z^+ = 0$, $C_z^- = 1$ in the $z = \frac{1}{2}$ plane has the mirror sector Chern numbers (0110) and the SIs (010).

Relationship with the SIs in other double SSGs – We next study the effects of imposing $\mathcal{T}$ symmetry. The double SSG 174.134 $P\bar{6}1'$ – the SSG generated by adding $\{\mathcal{T}|\mathbf{0}\}$ symmetry to MSG 174.133 $P\bar{6}$ – has the SI group $\mathbb{Z}_3 \times \mathbb{Z}_3$. The SIs in double SSG 174.134 $P\bar{6}1'$ are related to the SIs in double MSG 174.133 $P\bar{6}$ through the subduction relations:

$$(z_{3m,0}, z_{3m,\pi})_{P\bar{6}1'} \to (\delta_{3m}, z^+_{3m,\pi}, z^-_{3m,\pi})_{P\bar{6}} = (z_{3m,\pi} + z_{3m,0} \bmod 3,\ z_{3m,\pi},\ -z_{3m,\pi} \bmod 3)_{P\bar{6}}. \tag{318}$$

In Type-II double SSG 174.134 $P\bar{6}1'$ insulators with net-odd odd mirror Chern numbers[26] are 3D TIs. However, because $z_{3m,0}$ and $z_{3m,\pi}$ only indicate the mirror Chern numbers in the $k_z = 0, \pi$ planes modulo 3, then there is no relationship between $z_{3m,0}$ and $z_{3m,\pi}$ and the axion (3D TI) angle θ. Specifically, consider a 3D TI in double SSG 174.134 $P\bar{6}1'$ with $(z_{3m,0}, z_{3m,\pi})_{P\bar{6}1'} = (10)_{\bar{6}1'}$, where the bulk axion angle $\theta = \pi$. Taking three superposed copies of the 3D TI results in an insulator with the SIs $(00)_{\bar{6}1'}$ and the axion angle $\theta = \pi$. Hence, $z_{3m,0}$ and $z_{3m,\pi}$ are individually (and as a set) independent of θ, because $z_{3m,0}$ and $z_{3m,\pi}$ are $\mathbb{Z}_3$-valued, whereas θ is $\mathbb{Z}_2$-valued (if quantized). We thus conclude that, while axionic mirror TCI phases can be stabilized by $\{m_z|\mathbf{0}\}$ mirror and $\{S_6|\mathbf{0}\}$ rotoinversion symmetries in the magnetic subgroup double MSG 174.133 $P\bar{6}$ of double SSG 174.134 $P\bar{6}1'$, θ is not symmetry-indicated in double MSG 174.133 $P\bar{6}$.

p. Double SIs in Type-I Double MSG 175.137 $P6/m$

The double MSG 175.137 $P6/m$ is generated by $\{E|100\}$, $\{E|010\}$, $\{E|001\}$, $\{C_{6z}|\mathbf{0}\}$, and $\{\mathcal{I}|\mathbf{0}\}$, where the angle between the $\{E|100\}$ and $\{E|010\}$ translations is chosen to be $2\pi/3$ for consistency with the $\{C_{3z}|\mathbf{0}\} = (\{C_{6z}|\mathbf{0}\})^2$ rotation symmetry. We note that double MSG 175.137 $P6/m$ additionally contains a mirror symmetry operation: $\{m_z|\mathbf{0}\} = \{C_{6z}|\mathbf{0}\}^3\{\mathcal{I}|\mathbf{0}\}$.

SIs – The double MSG 175.137 $P6/m$ has the SI group $\mathbb{Z}_6^3$. In the physical basis, the three $\mathbb{Z}_6$-valued SIs are: $(\delta_{6m}, z^+_{6m,\pi}, z^-_{6m,\pi})$, for which the SI formulas are:

$$\begin{aligned}\delta_{6m} = C^+_{k_z=\pi} - C^-_{k_z=0} \bmod 6 = &-\frac{1}{2}n_A^{\frac{1}{2},+i} + \frac{1}{2}n_A^{-\frac{1}{2},+i} - \frac{3}{2}n_A^{\frac{3}{2},+i} + \frac{3}{2}n_A^{-\frac{3}{2},+i} - \frac{5}{2}n_A^{\frac{5}{2},+i} + \frac{5}{2}n_A^{-\frac{5}{2},+i}\\ &- n_H^{\frac{1}{2},+i} + n_H^{-\frac{1}{2},+i} + 3n_H^{\frac{3}{2},+i} + \frac{3}{2}n_L^{\frac{1}{2},+i} - \frac{3}{2}n_L^{-\frac{1}{2},+i}\\ &+ \frac{1}{2}n_\Gamma^{\frac{1}{2},-i} - \frac{1}{2}n_\Gamma^{-\frac{1}{2},-i} + \frac{3}{2}n_\Gamma^{\frac{3}{2},-i} - \frac{3}{2}n_\Gamma^{-\frac{3}{2},-i} + \frac{5}{2}n_\Gamma^{\frac{5}{2},-i} - \frac{5}{2}n_\Gamma^{-\frac{5}{2},-i}\\ &+ n_K^{\frac{1}{2},-i} - n_K^{-\frac{1}{2},-i} - 3n_K^{\frac{3}{2},-i} - \frac{3}{2}n_M^{\frac{1}{2},-i} + \frac{3}{2}n_M^{-\frac{1}{2},-i} \bmod 6,\end{aligned} \tag{319}$$

$$\begin{aligned}z^+_{6m,\pi} = C^+_{k_z=\pi} \bmod 6 = &-\frac{1}{2}n_A^{\frac{1}{2},+i} + \frac{1}{2}n_A^{-\frac{1}{2},+i} - \frac{3}{2}n_A^{\frac{3}{2},+i} + \frac{3}{2}n_A^{-\frac{3}{2},+i} - \frac{5}{2}n_A^{\frac{5}{2},+i} + \frac{5}{2}n_A^{-\frac{5}{2},+i}\\ &- n_H^{\frac{1}{2},+i} + n_H^{-\frac{1}{2},+i} + 3n_H^{\frac{3}{2},+i} + \frac{3}{2}n_L^{\frac{1}{2},+i} - \frac{3}{2}n_L^{-\frac{1}{2},+i} \bmod 6,\end{aligned} \tag{320}$$

$$\begin{aligned}z^-_{6m,\pi} = C^-_{k_z=\pi} \bmod 6 = &-\frac{1}{2}n_A^{\frac{1}{2},-i} + \frac{1}{2}n_A^{-\frac{1}{2},-i} - \frac{3}{2}n_A^{\frac{3}{2},-i} + \frac{3}{2}n_A^{-\frac{3}{2},-i} - \frac{5}{2}n_A^{\frac{5}{2},-i} + \frac{5}{2}n_A^{-\frac{5}{2},-i}\\ &- n_H^{\frac{1}{2},-i} + n_H^{-\frac{1}{2},-i} + 3n_H^{\frac{3}{2},-i} + \frac{3}{2}n_L^{\frac{1}{2},-i} - \frac{3}{2}n_L^{-\frac{1}{2},-i} \bmod 6,\end{aligned} \tag{321}$$

such that a 3D insulator with $z^+_{6m,\pi} \neq -z^-_{6m,\pi} \bmod 6$ is in a 3D QAH state. As we will show below, in insulators with net-zero position-space Chern numbers, odd values of δ_{6m} indicate mirror TCI phases with $\theta = \pi$. The compatibility relations require that the occupied bands in the $k_z = 0, \pi$ planes have the same rotation symmetry eigenvalues, and hence the same Chern numbers (modulo 6). In an insulating state (*i.e.* in the absence of bulk Weyl points), it is further required that $C^+_{k_z=0} + C^-_{k_z=0} = C^+_{k_z=\pi} + C^-_{k_z=\pi}$ and $C^+_{k_z=0} - C^-_{k_z=\pi} \bmod 6 = C^+_{k_z=\pi} - C^-_{k_z=0} \bmod 6 = \delta_{6m}$.

Layer constructions – To diagnose the topology associated to each nontrivial value of the double SIs $(\delta_{6m}, z^+_{6m,\pi}, z^-_{6m,\pi})$, we employ the layer construction method. In each layer construction, we denote the mirror

sector Chern numbers of the occupied bands at $k_z = 0, \pi$ as $(C^+_{k_z=0}, C^-_{k_z=0}, C^+_{k_z=\pi}, C^-_{k_z=\pi})$, and additionally compute the subduced SIs $(\eta_{4I}, z_{2I,1}, z_{2I,2}, z_{2I,3})_{P\bar{1}}$ in the subgroup double MSG 2.4 $P\bar{1}$ (see SN 31 a). The layer constructions for Type-I double MSG 175.137 $P6/m$ are given by:

1. A $\hat{\mathbf{z}}$-normal Chern layer with $C^+_z = 1$, $C^-_z = 0$ at $z = 0$ has the mirror sector Chern numbers (1010) and the SIs (110). The subduced subgroup SIs are $(\eta_{4I}, z_{2I,1}, z_{2I,2}, z_{2I,3})_{P\bar{1}} = (2001)_{P\bar{1}}$.

2. A $\hat{\mathbf{z}}$-normal Chern layer with $C^+_z = 0$, $C^-_z = 1$ at $z = 0$ has the mirror sector Chern numbers (0101) and the SIs (501). The subduced subgroup SIs are $(2001)_{P\bar{1}}$.

3. A $\hat{\mathbf{z}}$-normal Chern layer with $C^+_z = 1$, $C^-_z = 0$ at $z = \frac{1}{2}$ has the mirror sector Chern numbers (1001) and the SIs (001). The subduced subgroup SIs are $(0001)_{P\bar{1}}$.

4. A $\hat{\mathbf{z}}$-normal Chern layer with $C^+_z = 0$, $C^-_z = 1$ at $z = \frac{1}{2}$ has the mirror sector Chern numbers (0110) and the SIs (010). The subduced subgroup SIs are $(0001)_{P\bar{1}}$.

Relationship with the SIs in other double SSGs – To identify the AXI phases in double MSG 175.137 $P6/m$, we subduce the SIs onto double MSG 2.4 $P\bar{1}$:

$$\left(\delta_{6m}, z^+_{6m,\pi}, z^-_{6m,\pi}\right)_{P6/m} \to (\eta_{4I}, z_{2I,1}, z_{2I,2}, z_{2I,3})_{P\bar{1}} = \left(2(\delta_{6m} \bmod 2), 0, 0, z^+_{6m,\pi} + z^-_{6m,\pi} \bmod 2\right)_{P\bar{1}}. \tag{322}$$

SEq. (322) implies that the $\mathcal{I}$ AXI index $\eta_{2I'}$ [SEq. (249)] is related to δ_{6m} by $\eta_{2I'} = \frac{1}{2}\eta_{4I} = \delta_{6m} \bmod 2$, such that gapped states with $\delta_{6m} \bmod 2 = 1$ and $z^+_{6m,\pi} + z^-_{6m,\pi} \bmod 2 = 0$ in MSG 175.137 $P6/m$ are AXIs if the non-symmetry-indicated Chern numbers vanish.

Lastly, we study the effects of imposing $\mathcal{T}$ symmetry. The double SSG 175.138 $P6/m1'$ – the SSG generated by adding $\{\mathcal{T}|\mathbf{0}\}$ to double MSG 175.137 $P6/m$ – has the SI group $\mathbb{Z}_{12} \times \mathbb{Z}_6$. The SIs in double SSG 175.138 $P6/m1'$ are related to the SIs in double MSG 175.137 $P6/m$ through the subduction relations:

$$(z_{12}, z_{6m,\pi})_{P6/m1'} \to (\delta_{6m}, z^+_{6m,\pi}, z^-_{6m,\pi})_{P6/m} = (z_{12} \bmod 6, z_{6m,\pi}, -z_{6m,\pi} \bmod 6)_{P6/m}. \tag{323}$$

q. *Double SIs in Type-I Double MSG 176.143 $P6_3/m$*

The double MSG 176.143 $P6_3/m$ is generated by $\{E|100\}$, $\{E|010\}$, $\{E|001\}$, $\{C_{6z}|00\frac{1}{2}\}$, and $\{\mathcal{I}|\mathbf{0}\}$, where the angle between the $\{E|100\}$ and $\{E|010\}$ translations is chosen to be $2\pi/3$ for consistency with the $\{C_{3z}|\mathbf{0}\} = \{E|00\bar{1}\}\{C_{6z}|00\frac{1}{2}\}^2$ rotation symmetry. We note that double MSG 176.143 $P6_3/m$ additionally contains a mirror symmetry operation: $\{m_z|00\frac{1}{2}\} = \{E|00\bar{1}\}\{C_{6z}|00\frac{1}{2}\}^3\{\mathcal{I}|\mathbf{0}\}$.

SIs – The double MSG 176.143 $P6_3/m$ has the SI group $\mathbb{Z}_6 \times \mathbb{Z}_3$. In the physical basis, the SIs are $(z^+_{6m,0}, \delta_{3m})$, where $\delta_{3m} = C^+_{k_z=\pi} - C^-_{k_z=0} \bmod 3$ subduces to the same SI (δ_{3m}) in double MSG 174.133 $P\bar{6}$ [SEq. (315)]. The SI formula for $z^+_{6m,0}$ is given by:

$$\begin{aligned} z^+_{6m,0} = C^+_{k_z=0} \bmod 6 = &-\frac{1}{2}n_\Gamma^{\frac{1}{2},+i} + \frac{1}{2}n_\Gamma^{-\frac{1}{2},+i} - \frac{3}{2}n_\Gamma^{\frac{3}{2},+i} + \frac{3}{2}n_\Gamma^{-\frac{3}{2},+i} - \frac{5}{2}n_\Gamma^{\frac{5}{2},+i} + \frac{5}{2}n_\Gamma^{-\frac{5}{2},+i} \\ &- n_{\mathcal{K}}^{\frac{1}{2},+i} + n_{\mathcal{K}}^{-\frac{1}{2},+i} + 3n_{\mathcal{K}}^{\frac{3}{2},+i} + \frac{3}{2}n_M^{\frac{1}{2},+i} - \frac{3}{2}n_M^{-\frac{1}{2},+i} \bmod 6, \end{aligned} \tag{324}$$

where $n_K^{j,+i}$ is the number of occupied states with mirror $\{m_z|00\frac{1}{2}\}$ eigenvalue i and rotation eigenvalue $e^{-i\frac{2\pi}{n}j}$ ($n = 6, 3, 2$ for $K = \Gamma$, $\mathcal{K}$, and M, respectively). As we will show below, insulators with $z^+_{6m,0} \bmod 2 = 1$ and net-zero position-space Chern numbers in double MSG 176.143 $P6_3/m$ are AXIs – all of the other insulators in double MSG 176.143 $P6_3/m$ with nontrivial SIs are 3D QAH states.

Layer constructions – To diagnose the topology associated to each nontrivial value of the double SIs $(z^+_{6m,0}, \delta_{3m})$, we employ the layer construction method. In each layer construction, we denote the mirror sector Chern numbers of the occupied bands at $k_z = 0, \pi$ as $(C^+_{k_z=0}, C^-_{k_z=0}, C^+_{k_z=\pi}, C^-_{k_z=\pi})$, and additionally compute the subduced SIs $(\eta_{4I}, z_{2I,1}, z_{2I,2}, z_{2I,3})_{P\bar{1}}$ in the subgroup double MSG 2.4 $P\bar{1}$ (see SN 31 a). We note that, while the $\mathcal{I}$ centers in MSG 176.143 $P6_3/m$ lie in the $z = 0, \frac{1}{2}$ planes, the mirror planes lie at $z = \frac{1}{4}, \frac{3}{4}$ in each cell. The layer constructions for Type-I double MSG 176.143 $P6_3/m$ are given by:

1. A $\hat{\mathbf{z}}$-normal layer with $C_z = 1$ in the $z = 0, \frac{1}{2}$ planes has the mirror sector Chern numbers (1111) and the SIs (10). The subduced subgroup SIs are $(\eta_{4I}, z_{2I,1}, z_{2I,2}, z_{2I,3})_{P\bar{1}} = (2000)_{P\bar{1}}$.

2. A $\hat{\mathbf{z}}$-normal layer with $C_z^+ = 1$, $C_z^- = 0$ in the $z = \frac{1}{4}, \frac{3}{4}$ planes has the mirror sector Chern numbers (2011) and the SIs (21). The subduced subgroup SIs are $(0000)_{P\bar{1}}$.

3. A $\hat{\mathbf{z}}$-normal layer with $C_z^+ = 0$, $C_z^- = 1$ layer in the $z = \frac{1}{4}, \frac{3}{4}$ planes has the mirror sector Chern numbers (0211) and the SIs (02). The subduced subgroup SIs are $(0000)_{P\bar{1}}$.

Relationship with the SIs in other double SSGs – To identify the AXI phases, we subduce the SIs in double MSG 176.143 $P6_3/m$ onto double MSG 2.4 $P\bar{1}$:

$$(z_{6m,0}^+, \delta_{3m}) \rightarrow (\eta_{4I}, z_{2I,1}, z_{2I,2}, z_{2I,3})_{P\bar{1}} = (2(z_{6m,0}^+ \bmod 2), 000)_{P\bar{1}}, \tag{325}$$

which implies that the $\mathcal{I}$ AXI index $\eta_{2I'}$ [SEq. (249)] is related to $z_{6m,0}^+$ by $\eta_{2I'} = \frac{1}{2}\eta_{4I} = z_{6m,0}^+ \bmod 2$. Hence, we conclude that insulators in double MSG 176.143 $P6_3/m$ with $z_{6m,0}^+ \bmod 2 = 1$ and net-zero position-space Chern numbers are AXIs.

Lastly, we study the effects of imposing $\mathcal{T}$ symmetry. The double SSG 176.144 $P6_3/m1'$ – the SSG generated by adding $\{\mathcal{T}|\mathbf{0}\}$ to double MSG 176.143 $P6_3/m$ – has the SI group $\mathbb{Z}_{12}$. The SIs in double SSG 176.144 $P6_3/m1'$ are related to the SIs in double MSG 176.143 $P6_3/m$ through the subduction relations:

$$(z'_{12})_{P6_3/m1'} \rightarrow (z_{6m,0}^+, \delta_{3m})_{P6_3/m} = (z'_{12} \bmod 6, z'_{12} \bmod 3)_{P6_3/m}. \tag{326}$$

r. Double SIs in Type-I Double MSG 191.233 $P6/mmm$

The double MSG 191.233 $P6/mmm$ is generated by $\{E|100\}$, $\{E|010\}$, $\{E|001\}$, $\{C_{6z}|\mathbf{0}\}$, $\{\mathcal{I}|\mathbf{0}\}$, and $\{m_x|\mathbf{0}\}$, where the angle between the $\{E|100\}$ and $\{E|010\}$ translations is chosen to be $2\pi/3$ for consistency with the $\{C_{3z}|\mathbf{0}\} = (\{C_{6z}|\mathbf{0}\})^2$ rotation symmetry. We note that double MSG 191.233 $P6/mmm$ additionally contains a mirror symmetry operation: $\{m_z|\mathbf{0}\} = \{C_{6z}|\mathbf{0}\}^3\{\mathcal{I}|\mathbf{0}\}$. In Cartesian coordinates (x, y, z), the primitive lattice translation vectors in double MSG 191.233 $P6/mmm$ – $\{E|100\}$, $\{E|010\}$, and $\{E|001\}$ – respectively correspond to $\mathbf{t}_1 = (0, -1, 0)$, $\mathbf{t}_2 = (\frac{\sqrt{3}}{2}, \frac{1}{2}, 0)$, and $\mathbf{t}_3 = (0, 0, 1)$.

SIs – The double MSG 191.233 $P6/mmm$ has the SI group $\mathbb{Z}_{12} \times \mathbb{Z}_6$. In double-valued small irreps of the little groups at the $\mathcal{I}$-invariant $\mathbf{k}$ points, the matrix representatives of perpendicular mirror symmetries (*e.g.* $\{m_x|\mathbf{0}\}$ and $\{m_y|\mathbf{0}\}$) anticommute. Hence, Bloch states at the eight $\mathcal{I}$-invariant momenta must be at least twofold degenerate (and in fact are exactly twofold degenerate in double MSG 191.233 $P6/mmm$). The double SIs can be chosen to be the same as the double SIs of SSG 191.234 $P6/mmm1'$ (previously introduced in SRef. 26), because the addition of $\mathcal{T}$ symmetry to double MSG 191.233 $P6/mmm$ does not change the dimensions and characters of the small irreps at the high-symmetry BZ points or the compatibility relations between the high-symmetry-point small irreps. In the physical basis, the $\mathbb{Z}_{12}$ double SI is:

$$z_{12} = \delta_{6m} + 3[(\delta_{6m} - z_4) \bmod 4] \bmod 12, \tag{327}$$

where δ_{6m} is computed by subduction onto double MSG 175.137 $P6/m$ [SEq. (319)], and z_4 is computed by subduction onto double MSG 2.4 $P\bar{1}$ [SEq. (259)]. As we will show below, odd values of the strong index z_{12} indicate mirror TCI phases with $\theta = \pi$ (*i.e.* AXIs), and nontrivial even values indicate non-axionic (helical) magnetic TCI and HOTI phases. Lastly, in the physical basis, the $\mathbb{Z}_6$-valued double SI is the weak TCI invariant $z_{6m,\pi}^+$ for the mirror Chern number (modulo 6) in the $k_z = \pi$ plane, and can also be computed by subduction onto double MSG 175.137 $P6/m$ [SEq. (320)].

Layer constructions – To diagnose the topology associated to each nontrivial value of the double SIs, we employ the layer construction method. In the layer constructions below, $C^+ = -C^-$ due to the net-zero Chern numbers enforced by the mirror symmetries. Hence, we will omit C^- in further discussions of the topology in double MSG 191.233 $P6/mmm$. The layer constructions for the double SIs $(z_{12}, z_{6m,\pi}^+)$ in MSG 191.233 $P6/mmm$ are given by:

1. A $\hat{\mathbf{z}}$-normal layer with $C_z^+ = 1$ in the $z = 0$ plane has the SIs (21).

2. A $\hat{\mathbf{z}}$-normal layer with $C_z^+ = 1$ in the $z = \frac{1}{2}$ plane has the SIs (05).

3. An $\hat{\mathbf{x}}$-normal layer with $C_x^+ = 1$ in the $x = 0$ plane has the SIs (60). We emphasize that, in this layer construction, there are also $|C^+| = 1$ mirror Chern layers in the $C_{6z}\hat{\mathbf{x}}$, $C_{6z}^2\hat{\mathbf{x}}$, $C_{6z}^3\hat{\mathbf{x}}$, $C_{6z}^4\hat{\mathbf{x}}$, and $C_{6z}^5\hat{\mathbf{x}}$ directions implied by the $\{C_{6z}|\mathbf{0}\}$ rotation symmetry.

4. A $\hat{\mathbf{y}}$-normal layer with $C_y^+ = 1$ in the $y = 0$ plane has the SIs (60). We emphasize that, in this layer construction, there are also $|C^+| = 1$ mirror Chern layers in the $C_{6z}\hat{\mathbf{y}}$, $C_{6z}^2\hat{\mathbf{y}}$, $C_{6z}^3\hat{\mathbf{y}}$, $C_{6z}^4\hat{\mathbf{y}}$, and $C_{6z}^5\hat{\mathbf{y}}$ directions implied by the $\{C_{6z}|\mathbf{0}\}$ rotation symmetry.

Axion insulators – We find that states with odd z_{12} SIs cannot be constructed from layers of 2D stable topological phases. However, we may still use subduction relations to determine the bulk topology of insulators with odd values of z_{12}. First, as we will show below, insulators in double MSG 191.233 $P6/mmm$ with z_{12} mod 2 = 1 subduce to $(2000)_{P\bar{1}}$ in MSG 2.4 $P\bar{1}$. Hence, if the z_{12} mod 2 = 1 phases in double MSG 191.233 $P6/mmm$ are insulating, then the bulk insulator must either be an AXI or a 3D QAH state. Because the net Chern numbers $C_{x,y,z} = 0$ must vanish if the bulk is gapped, due to the mirror symmetries of double MSG 191.233 $P6/mmm$, then insulators with z_{12} mod 2 = 1 in double MSG 191.233 $P6/mmm$ must be AXIs. This result can also be understood by subducing from a $\mathcal{T}$-symmetric SSG. Specifically, because insulators with the double SIs z_{12} mod 2 = 1 in double MSG 191.233 $P6/mmm$ can be subduced from insulators with $(z_{12})_{P6/mmm1'}$ mod 2 = 1 in Type-II SG 191.234 $P6/mmm1'$, which correspond to $\mathcal{T}$-symmetric 3D TIs with $\theta = \pi$[24,26,27], then the double SIs z_{12} mod 2 = 1 in double MSG 191.233 $P6/mmm$ are compatible with bulk-gapped states. Hence, we conclude that 3D insulators with z_{12} mod 2 = 1 in double MSG 191.233 $P6/mmm$ are AXIs, *without ambiguity*. We conjecture that z_{12} mod 2 = 1 AXIs in double MSG 191.233 $P6/mmm$ can be constructed using the topological crystal method[168], which additionally incorporates cell complexes of 2D Chern insulators, TIs, and TCIs.

Helical HOTI phases protected by mirror and C_6 rotation symmetry – First, the double SIs $(60)_{P6/mmm1'}$ of Type-II double SSG SG 191.234 $P6/mmm1'$ either correspond to a rotation-anomaly (non-axionic, *i.e.* θ mod $2\pi = 0$) HOTI protected by C_6 and $\mathcal{T}$ symmetries, or a mirror TCI with C_{m_z} mod 12 = 6 (*c.f.* Table 7 in the Supplementary Material of SRef. 26). In the C_6- and $\mathcal{T}$-symmetric HOTI phase, there are $6 + 12n$ ($n \in \{\mathbb{Z}^+, 0\}$) helical hinge modes on a z-directed, C_6- and $\mathcal{T}$-symmetric rod, and $6+12n$ twofold Dirac points on the top ($\hat{\mathbf{z}}$-normal) rod surface that are locally protected by mirror symmetry (see SN 33). Because double SSG SG 191.234 $P6/mmm1'$ contains $\{m_{x,y}|\mathbf{0}\}$ symmetries (as well as their conjugates under C_{6z} symmetry), then six of the helical hinge modes on the boundary of a $6/mmm1'$-symmetric sample must also be pinned to the hinge projections of bulk mirror planes whose normal vectors lie in the xy-plane, and must be indicated by bulk mirror Chern numbers. Hence, when $\mathcal{T}$ symmetry is relaxed in a sixfold rotation-anomaly $(60)_{P6/mmm1'}$ HOTI phase in Type-II double SSG 191.234 $P6/mmm1'$ while preserving the symmetries of MSG 191.233 $P6/mmm$, the surface and hinge states will remain gapless and anomalous [see SFig. 21(c) and SN 35].

We will next prove that there are $6+12n$ twofold Dirac points on the top surface of a $6/mmm$-symmetric nanorod of the (60) sixfold rotation-anomaly magnetic HOTI phase in double MSG 191.233 $P6/mmm$ introduced in this work. We denote the four layer constructions as L_a ($a = 1\cdots4$), respectively. First, we note that the (60) mirror TCI phase with C_{m_z} mod 12 = 6 can be constructed as $(6m+3)L_1 \oplus (6m'+3)L_2$. Next, the sixfold rotation-anomaly HOTI phase can be constructed as $(2n+1)L_3$, or $(2n+1)L_4$, or through any superposition of an odd number of the aforementioned layer constructions. Adding $6L_1$ or $6L_2$, which have SIs (00), to the layer-constructed HOTI phase will not change the top surface spectrum, because L_1 and L_2 consist of horizontal (*i.e.* $\hat{\mathbf{z}}$-normal) layers, and hence only contribute surface and hinge states on boundaries whose normal vectors lie in the xy-plane.

We will thus focus on the top surface spectra of the $(2n+1)L_3$ and $(2n+1)L_4$ layer constructions. We first consider $(2n+1)L_3$. As shown in Supplementary Note 5 in SRef. 26 and in Table 6 of the Supplementary Material of SRef. 26, the Chern numbers in the m_x mirror sectors are given by $C^+_{k_x=0} = -C^-_{k_x=0} = 4n+2$, $C^+_{k_x=\pi} = -C^-_{k_x=\pi} = 0$. In the 2D top surface BZ, $C^+_{k_x=0}$ mandates the presence of $|4n+2|$ twofold Dirac points on the $k_x = 0$ line. Due to the C_{6z} symmetry, there must also be $2|4n+2|$ twofold Dirac points on the C_{6z} and C_{6z}^{-1} conjugates of the $k_x = 0$ line. Hence, the total number of top-surface Dirac points is $3|4n+2|$ mod 12 = 6. Lastly, we note that performing the analogous analysis on the $(2n+1)L_4$ layer construction also returns the same number of mirror-protected twofold Dirac points on the top surface $(6+12n)$.

In SN 34, we will prove that, on the top surface of the (60) HOTI state – which respects the symmetries of Type-I double magnetic wallpaper group[36,39,73–75] $p6m$ – the presence of $6+12n$ ($n \in \{\mathbb{Z}^+, 0\}$) twofold surface Dirac points circumvents the fermion multiplication theorem for 2D lattices with double magnetic wallpaper group $p6m$.

Relationship with the SIs in other double SSGs – To identify the AXI phases, we subduce the SIs onto double MSG 2.4 $P\bar{1}$:

$$(z_{12}, z^+_{6m,\pi})_{P6/mmm} \rightarrow (\eta_{4I}, z_{2I,1}, z_{2I,2}, z_{2I,3})_{P\bar{1}} = (2(z_{12} \bmod 2), 000)_{P\bar{1}}. \tag{328}$$

Because the AXI $\mathcal{I}$ SI $\eta_{2I'} = \frac{1}{2}\eta_{4I} = z_{12}$ mod 2 [SEq. (249)], then we conclude that insulators with odd z_{12} SIs in double MSG 191.233 $P6/mmm$ are AXIs.

s. Double SIs in Type-II Double SG 2.5 $P\bar{1}1'$

Using the definition of a minimal double SSG established in SN 30, we find that there are only five minimal Type-II double SSGs: 2.5 $P\bar{1}1'$, 83.44 $P4/m1'$, 87.76 $I4/m1'$, 175.138 $P6/m1'$, and 176.144 $P6_3/m1'$. The SIs, SI formulas, and physical interpretation of the SIs in the Type-II double SSGs were previously determined in SRefs. 24,26,27. In the physical basis employed in this work, the SI formulas, physical interpretations, and layer constructions of the double SIs in the above minimal Type-II double SSGs are provided in SRef. 26. Here and below [SN 31 t, 31 u, 31 v, and 31 w, respectively], we will briefly review the established SI formulas and physical interpretations of the double SIs in the five minimal Type-II double SSGs.

To begin, the double SSG 2.5 $P\bar{1}1'$ is generated by $\{E|100\}$, $\{E|010\}$, $\{E|001\}$, $\{\mathcal{I}|\mathbf{0}\}$, and $\{\mathcal{T}|\mathbf{0}\}$.

The SI group is $\mathbb{Z}_4\times\mathbb{Z}_2^3$. In the physical basis, the four double SIs $(z_4, z_{2w,1}, z_{2w,2}, z_{2w,3})$ of double SSG 2.5 $P\bar{1}1'$ have the respective SI formulas:

$$z_4 = \sum_K \frac{1}{2} n_K^- = \sum_K \frac{n_K^- - n_K^+}{4} \text{ mod } 4, \tag{329}$$

$$z_{2w,i} = \sum_{K, K_i=\pi} \frac{1}{2} n_K^- = \sum_{K, K_i=\pi} \frac{n_K^- - n_K^+}{4} \text{ mod } 2 \qquad (i=1,2,3), \tag{330}$$

where K runs over the eight $\mathcal{I}$-invariant momenta in the first BZ, and $n_K^{\pm}$ are the number of Bloch states with ± 1 parity ($\mathcal{I}$) eigenvalues at K in the group of bands under consideration. The double SIs $(z_4, z_{2w,1}, z_{2w,2}, z_{2w,3})_{P\bar{1}1'}$ in double SSG 2.5 $P\bar{1}1'$ have the same SI formulas as the double SIs in $(z_4, z_{2w,1}, z_{2w,2}, z_{2w,3})_{Pmmm}$ in double MSG 47.249 $Pmmm$ [SEqs. (259) and (260)], which we previously analyzed in SN 31 d.

The physical interpretations of the double SIs in Type-II double SSG 2.5 $P\bar{1}1'$ are given below:[26]:

1. $z_4 = 1, 3$ indicate strong 3D TIs protected by $\mathcal{T}$ symmetry.
2. For $z_4 = 0, 2$, $z_{2w,i} = 1$ indicates a weak TI phase that can be deformed into a stack of 2D TIs whose normal vectors point in the i-direction [*e.g.*, the double SIs $(z_4, z_{2w,1}, z_{2w,2}, z_{2w,3})_{P\bar{1}1'} = (2110)_{P\bar{1}1'}$ indicate a weak TI that is equivalent to a stack of 2D TIs oriented in the $x+y$-direction].
3. For $z_{2w,1} = z_{2w,2} = z_{2w,3} = 0$, $z_4 = 2$ indicates a non-axionic helical HOTI protected by $\mathcal{I}$ and $\mathcal{T}$ symmetries with a sample-encircling helical hinge mode (see Supplementary Note 5 in SRef. 26).

t. Double SIs in Type-II Double SG 83.44 $P4/m1'$

The double SSG 83.44 $P4/m1'$ is generated by $\{E|100\}$, $\{E|010\}$, $\{E|001\}$, $\{\mathcal{I}|\mathbf{0}\}$, $\{C_{4z}|\mathbf{0}\}$, and $\{\mathcal{T}|\mathbf{0}\}$.

The double SSG 83.44 $P4/m1'$ has the SI group $\mathbb{Z}_8\times\mathbb{Z}_4\times\mathbb{Z}_2$. In the physical basis, the $\mathbb{Z}_8$ double SI has the SI formula:

$$z_8 = \frac{3}{2} n^{\frac{3}{2},+} - \frac{3}{2} n^{\frac{3}{2},-} - \frac{1}{2} n^{\frac{1}{2},+} + \frac{1}{2} n^{\frac{1}{2},-} \text{ mod } 8, \tag{331}$$

$$n^{j,\pm} = \sum_{K=\Gamma,M,Z,A} n_K^{j,\pm} + \sum_{K=X,R} n_K^{\frac{1}{2},\pm}, \tag{332}$$

where $n_K^{j,\pm}$ $(K=\Gamma,M,Z,A)$ are the number of states at the momentum K with parity ($\mathcal{I}$) eigenvalue ± 1 and $\{C_{4z}|\mathbf{0}\}$ eigenvalue angular momentum j (modulo 4), and $n_K^{j,\pm}$ $(K=X,R)$ are the number of states at the momentum K with parity eigenvalue ± 1 and angular momentum j (modulo 2). The $\mathbb{Z}_4$ SI subduces to the weak TCI invariant $z_{4m,\pi}^-$ in double MSG 83.43 $P4/m$ [SEq. (274)], and the $\mathbb{Z}_2$ SI subduces to the weak TI invariant $z_{2w,1}$ in double SSG 2.5 $P\bar{1}1'$ [SEq. (260)]. We note that in SRef. 26, $z_{4m,\pi}^-$ is instead labeled $z_{4m,\pi}$. As a set, the three double SIs $(z_8, z_{4m,\pi}^-, z_{2w,1})_{P4/m1'}$ in double SSG 83.44 $P4/m1'$ have the same SI formulas as the double SIs $(z_8, z_{4m,\pi}^-, z_{2w,1})_{P4/mmm}$ in double MSG 123.339 $P4/mmm$, which we previously analyzed in SN 31 k.

The physical interpretations of the double SIs in Type-II double SSG 83.44 $P4/m1'$ are given below[26]:

1. $z_{2w,1} = 1$ indicates the presence of nontrivial weak TI indices in the $k_{x,y} = \pi$ planes.
2. Nonzero values of $z^{-}_{4m,\pi}$ indicate nontrivial mirror sector Chern numbers in the $k_z = \pi$ plane: $z^{-}_{4m,\pi} = C^{-}_{k_z=\pi} \bmod 4 = -C^{+}_{k_z=\pi} \bmod 4$ [see SEq. (274) and the surrounding text].
3. $z_8 \neq 0, 4$ indicate nontrivial mirror sector Chern numbers in the $k_z = 0, \pi$ planes: $C^{-}_{k_z=0} - C^{+}_{k_z=\pi} \bmod 4 = z_8$ [see SN 31 h for the subduction relations between $(z_8)_{P4/m1'}$ in double SSG 83.44 $P4/m1'$ and the double SIs in double MSG 83.43 $P4/m$]. $z_8 \bmod 2 = 1$ specifically indicates strong 3D TI phases.
4. For $z_{2w,1} = z^{-}_{4m,\pi} = 0$, $z_8 = 4$ either indicates a mirror TCI phase with $C_{m_z} \bmod 8 = 4$, or a non-axionic fourfold rotation-anomaly HOTI phase with C_{4z}- and $\mathcal{T}$-symmetry-protected bulk topology and $4 + 8n$ ($n \in \{\mathbb{Z}^+, 0\}$) $\mathcal{T}$-protected helical hinge modes (see Supplementary Note 5 in SRef. 26).

u. Double SIs in Type-II Double SG 87.76 $I4/m1'$

The double SSG 87.76 $I4/m1'$ is generated by $\{E|\bar{\frac{1}{2}}\frac{1}{2}\frac{1}{2}\}$, $\{E|\frac{1}{2}\bar{\frac{1}{2}}\frac{1}{2}\}$, $\{E|\frac{1}{2}\frac{1}{2}\bar{\frac{1}{2}}\}$, $\{\mathcal{I}|\mathbf{0}\}$, $\{C_{4z}|\mathbf{0}\}$, and $\{\mathcal{T}|\mathbf{0}\}$.
The double SSG 87.76 $I4/m1'$ has the SI group $\mathbb{Z}_8 \times \mathbb{Z}_2$. In the physical basis, the $\mathbb{Z}_8$ double SI subduces to $(z_8)_{P4/m1'}$ in double SSG 83.44 $P4/m1'$ (see SN 31 t):

$$z_8 = \frac{3}{2}n^{\frac{3}{2},+} - \frac{3}{2}n^{\frac{3}{2},-} - \frac{1}{2}n^{\frac{1}{2},+} + \frac{1}{2}n^{\frac{1}{2},-} \bmod 8, \tag{333}$$

in which $n^{j,\pm}$ are given by:

$$n^{j,\pm} = \sum_{K=\Gamma,M} n^{j,\pm}_K + \sum_{K=X,N} n^{\frac{1}{2},\pm}_K + \sum_{K=P} n^{\pm j}_K, \tag{334}$$

where $n^{j,\pm}_K$ ($K = \Gamma, M$) are the number of states at the momentum K with parity eigenvalue ± 1 and $\{C_{4z}|\mathbf{0}\}$ eigenvalue angular momentum j (modulo 4), $n^{j,\pm}_K$ ($K = X, N$) are the number of states at the momentum K with parity eigenvalue ± 1 and angular momentum j (modulo 2), and $n^{\pm j}_P$ are the number of states at the momentum P with $\{S_{4z}|0\}$ eigenvalue $e^{\mp i\frac{2\pi}{4}j}$. The $\mathbb{Z}_2$ SI subduces to the weak TI invariant $z_{2w,1}$ in double SSG 2.5 $P\bar{1}1'$ [SEq. (260)].
The physical interpretations of the double SIs in Type-II double SSG 87.76 $I4/m1'$ are closely related to the physical interpretations of the double SIs in double SSG 83.44 $P4/m1'$ previously determined in SN 31 t and SRef. 26:

1. $z_{2w,1} = 1$ indicates the presence of nontrivial weak TI indices in the $k_{x,y} = \pi$ planes in the primitive-cell BZ.
2. $z_8 \neq 0, 4$ indicate nontrivial mirror sector Chern numbers in the $k_z = 0$ plane: $C^{-}_{k_z=0} \bmod 4 = -C^{+}_{k_z=0} \bmod 4 = z_8$ (noting that the $k_z = 0, \pi$ planes are related by reciprocal lattice vectors, because the Bravais lattice of SSG 87.76 $I4/m1'$ is body-centered tetragonal[33]). $z_8 \bmod 2 = 1$ specifically indicates strong 3D TI phases.
3. For $z_{2w,1} = 0$, $z_8 = 4$ either indicates a mirror TCI phase with $C_{m_z} \bmod 8 = 4$, or a non-axionic fourfold rotation-anomaly HOTI phase with C_{4z}- and $\mathcal{T}$-symmetry-protected bulk topology and $4 + 8n$ ($n \in \{\mathbb{Z}^+, 0\}$) $\mathcal{T}$-protected helical hinge modes (see Supplementary Note 5 in SRef. 26).

v. Double SIs in Type-II Double SG 175.138 $P6/m1'$

The double SSG 175.138 $P6/m1'$ is generated by $\{E|100\}$, $\{E|010\}$, $\{E|001\}$, $\{\mathcal{I}|\mathbf{0}\}$, $\{C_{6z}|\mathbf{0}\}$, and $\{\mathcal{T}|\mathbf{0}\}$.
The double SSG 175.138 $P6/m1'$ has the SI group $\mathbb{Z}_{12} \times \mathbb{Z}_6$. In the physical basis, the SI formula of the $\mathbb{Z}_{12}$ SI can be expressed in terms of previously established double SIs[26]:

$$z_{12} = \delta_{6m} + 3[(\delta_{6m} - z_4) \bmod 4] \bmod 12, \tag{335}$$

where δ_{6m} is computed by subduction onto double MSG 175.137 $P6/m$ [SEq. (319)], and z_4 is computed by subduction onto double SSG 2.5 $P\bar{1}1'$ [see SN 31 s]. Additionally, in the physical basis, the $\mathbb{Z}_6$ SI is the weak TCI invariant $z^{+}_{6m,\pi}$ for the mirror Chern number (modulo 6) in the $k_z = \pi$ plane, and can also be computed by subduction onto double

MSG 175.137 $P6/m$ [SEq. (320)]. We note that in SRef. 26, $z^+_{6m,\pi}$ is instead labeled $z_{6m,\pi}$. As a set, the two double SIs $(z_{12}, z^+_{6m,\pi})_{P6/m1'}$ in double SSG 175.138 $P6/m1'$ have the same SI formulas as the double SIs $(z_{12}, z^+_{6m,\pi})_{P6/mmm}$ in double MSG 191.233 $P6/mmm$, which we previously analyzed in SN 31 r.

The physical interpretations of the double SIs in double SSG 175.138 $P6/m1'$ are given below[26]:

1. Nonzero values of $z^+_{6m,\pi}$ indicate nontrivial mirror sector Chern numbers in the $k_z = \pi$ plane: $z^+_{6m,\pi} = C^+_{k_z=\pi} \bmod 6 = -C^-_{k_z=\pi} \bmod 6$ [see SEq. (320) and the surrounding text].

2. $z_{12} \neq 0, 6$ indicate nontrivial mirror sector Chern numbers in the $k_z = 0, \pi$ planes: $C^+_{k_z=\pi} - C^-_{k_z=0} \bmod 6 = z_{12}$ [see SN 31 p for the subduction relations between $(z_{12})_{P6/m1'}$ in double SSG 175.138 $P6/m1'$ and the double SIs in double MSG 175.137 $P6/m$]. $z_{12} \bmod 2 = 1$ specifically indicates strong 3D TI phases.

3. For $z^+_{6m,\pi} = 0$, $z_{12} = 6$ either indicates a mirror TCI phase with $C_{m_z} \bmod 12 = 6$, or a non-axionic sixfold rotation-anomaly HOTI phase with C_{6z}- and $\mathcal{T}$-symmetry-protected bulk topology and $6 + 12n$ $(n \in \{\mathbb{Z}^+, 0\})$ $\mathcal{T}$-protected helical hinge modes (see Supplementary Note 5 in SRef. 26).

w. *Double SIs in Type-II Double SG 176.144 $P6_3/m1'$*

The double SSG 176.144 $P6_3/m1'$ is generated by $\{E|100\}$, $\{E|010\}$, $\{E|001\}$, $\{\mathcal{I}|\mathbf{0}\}$, $\{C_{6z}|00\frac{1}{2}\}$, and $\{\mathcal{T}|\mathbf{0}\}$.

The double SSG 176.144 $P6_3/m1'$ has the SI group $\mathbb{Z}_{12}$. In the physical basis, the SI formula of the $\mathbb{Z}_{12}$ SI can be expressed in terms of previously established double SIs[26]:

$$z'_{12} = z^+_{6m,0} + 3[(z^+_{6m,0} - z_4) \bmod 4] \bmod 12, \tag{336}$$

where $z^+_{6m,0}$ is computed by subduction onto double MSG 176.143 $P6_3/m$ [SEq. (324)], and z_4 is computed by subduction onto double SSG 2.5 $P\bar{1}1'$ [see SN 31 s]. We note that, unlike previously in double SSG 175.138 $P6/m1'$ (SN 31 v), the mirror sector Chern numbers in the $k_z = \pi$ plane individually vanish $C^{\pm}_{k_z=\pi} = 0$ for any group of bands in double SSG 176.144 $P6_3/m1'$. This can be seen by first recognizing that the matrix representatives of $\{\mathcal{T}C_{6z}|00\frac{1}{2}\}$ and $\{m_z|00\frac{1}{2}\}$ anticommute in any small corep of any little group in the $k_z = \pi$ plane that contains both $\{\mathcal{T}C_{6z}|00\frac{1}{2}\}$ and $\{m_z|00\frac{1}{2}\}$. Hence, if $|\psi\rangle$ is a Bloch eigenstate of $\{m_z|00\frac{1}{2}\}$ at a $\mathbf{k}$ point in the $k_z = \pi$ plane with the $\{m_z|00\frac{1}{2}\}$ eigenvalue i, then $\{\mathcal{T}C_{6z}|00\frac{1}{2}\}|\psi\rangle$ is also an eigenstate of $\{m_z|00\frac{1}{2}\}$ with the same eigenvalue (i). Consequently, there is an effective time-reversal symmetry ($\{\mathcal{T}C_{6z}|00\frac{1}{2}\}$) within each mirror sector, which enforces that the mirror sector Chern numbers in the $k_z = \pi$ plane individually vanish.

The physical interpretations of the double SIs in double SSG 176.144 $P6_3/m1'$ are given below[26]:

1. $z'_{12} \neq 0, 6$ indicate nontrivial mirror sector Chern numbers in the $k_z = 0$ plane: $C^+_{k_z=0} \bmod 6 = -C^-_{k_z=0} \bmod 6 = z'_{12}$ [see SN 31 q for the subduction relations between $(z'_{12})_{P6_3/m1'}$ in double SSG 176.144 $P6_3/m1'$ and the double SIs in double MSG 176.143 $P6_3/m$]. $z'_{12} \bmod 2 = 1$ specifically indicates strong 3D TI phases.

2. $z'_{12} = 6$ either indicates a mirror TCI phase with $C_{m_z} \bmod 12 = 6$, or a non-axionic sixfold rotation-anomaly HOTI phase with 6_3-screw- and $\mathcal{T}$-symmetry-protected bulk topology and $6 + 12n$ $(n \in \{\mathbb{Z}^+, 0\})$ $\mathcal{T}$-protected helical hinge modes (see Supplementary Note 5 in SRef. 26).

x. *Double SIs in Type-III Double MSG 27.81 $Pc'c'2$*

Finally, beginning here with double MSG 27.81 $Pc'c'2$ and continuing below, we will introduce the physical-basis SI formulas and the physical interpretations of the double SIs in the 11 minimal Type-III double MSGs (see SN 30). To begin, the double MSG 27.81 $Pc'c'2$ is generated by $\{E|100\}$, $\{E|010\}$, $\{E|001\}$, $\{C_{2z}|\mathbf{0}\}$, and $\{\mathcal{T}m_x|00\frac{1}{2}\}$.

SI – The double MSG 27.81 $Pc'c'2$ has the SI group $\mathbb{Z}_2$. As we will shortly demonstrate, in the physical basis, the double SI z'_{2R} indicates the *even-valued* Chern number in the $k_z = \pi$ plane (modulo 4): $C_{k_z=\pi} \bmod 4 = 2z'_{2R}$. Hence, insulators with $z'_{2R} = 1$ are 3D QAH states with $C_z \bmod 4 = 2$.

We will first demonstrate that Bloch states at the $\{C_{2z}|\mathbf{0}\}$-invariant momenta in the $k_z = \pi$ plane in double MSG 27.81 $Pc'c'2$ form doubly-degenerate pairs with the same $\{C_{2z}|\mathbf{0}\}$ eigenvalues. To begin, in the $k_z = \pi$ plane, the matrix representative of $\{\mathcal{T}m_x|00\frac{1}{2}\}$ squares to minus the identity in all double-valued small coreps. Hence, all of the irreducible small coreps in the $k_z = \pi$ plane along the $\{\mathcal{T}m_x|00\frac{1}{2}\}$-invariant lines $k_y = 0, \pi$ must be at least twofold degenerate (and in fact are exactly twofold degenerate in double MSG 27.81 $Pc'c'2$). Next, the matrix representatives

of $\{\mathcal{T}m_x|00\frac{1}{2}\}$ and $\{C_{2z}|\mathbf{0}\}$ anticommute in all small coreps at the $\{C_{2z}|\mathbf{0}\}$-invariant points $k_{x,y}=0,\pi$ in the $k_z=\pi$ plane. This implies that, if $|\psi\rangle$ is a Bloch state at $k_{x,y}=0,\pi$ in the $k_z=\pi$ plane for which $\{C_{2z}|\mathbf{0}\}|\psi\rangle=i|\psi\rangle$, then:

$$\{C_{2z}|\mathbf{0}\}\{\mathcal{T}m_x|00\frac{1}{2}\}|\psi\rangle=-\{\mathcal{T}m_x|00\frac{1}{2}\}\{C_{2z}|\mathbf{0}\}|\psi\rangle=i\{\mathcal{T}m_x|00\frac{1}{2}\}|\psi\rangle. \tag{337}$$

SEq. (337) implies that both Bloch states in each $\{\mathcal{T}m_x|00\frac{1}{2}\}$ doublet at $k_{x,y}=0,\pi$, $k_z=\pi$ must have the *same* $\{C_{2z}|\mathbf{0}\}$ eigenvalues. We therefore define the $\mathbb{Z}_2$ SI as the parity of the number of doublets with $\{C_{2z}|\mathbf{0}\}$ eigenvalue $-i$ in the $k_z=\pi$ plane:

$$z'_{2R}=\sum_{K=Z,T,U,R}\frac{1}{2}n_K^{\frac{1}{2}} \bmod 2=\frac{C_{k_z=\pi}}{2} \bmod 2, \tag{338}$$

where $n_K^{\frac{1}{2}}$ is the number of states with $\{C_{2z}|\mathbf{0}\}$ eigenvalue $-i$, such that $\frac{1}{2}n_K^{\frac{1}{2}}$ is the number of *doublets* in which both Bloch states have the $\{C_{2z}|\mathbf{0}\}$ eigenvalues $-i$.

Layer constructions – To diagnose the topology associated to $z'_{2R}=1$, we employ the layer construction method. We begin by placing a $\hat{\mathbf{z}}$-normal Chern layer with $C_z=1$ in the $z=0$ plane. Due to the $\{Tm_x|00\frac{1}{2}\}$ symmetry in double MSG 27.81 $Pc'c'2$, there must be another Chern layer with $C_z=1$ in the $z=\frac{1}{2}$ plane, such that the total Chern number per cell is $C_z=2$, and the Chern number of the occupied bands in the $k_z=\pi$ plane is $C_{k_z=\pi}=2$. Hence, in this layer construction of a 3D QAH state with $C_z=2$, the $\mathbb{Z}_2$ SI is nontrivial $z'_{2R}=1$.

Relationship with the SIs in other double SSGs – We next compute the subduction relations between the SIs in double MSG 27.81 $Pc'c'2$ and the SIs in the maximal unitary subgroup double MSG 3.1 $P2$ (see SN 31 b):

$$(z'_{2R})_{Pc'c'2}\rightarrow(z_{2R})_{P2}=(0)_{P2}. \tag{339}$$

SEqs. (338) and (339) imply that symmetry-indicated 3D QAH states with $z'_{2R}=1$ in double MSG 27.81 $Pc'c'2$ necessarily subduce to *non-symmetry-indicated* 3D QAH states with $(z_{2R})_{P2}=0$ in double MSG 3.1 $P2$, in agreement with the physical-basis double SI relations $C_{k_z=\pi} \bmod 4=2z'_{2R}$ and $C_{k_z=\pi} \bmod 2=z_{2R}$ [taking the twofold axis in double MSG 3.1 $P2$ to be oriented in the z-direction, see SEq. (253) and the surrounding text].

Lastly, if we impose $\mathcal{T}$ symmetry, then the position-space Chern numbers must vanish, which enforces z'_{2R} to be zero. Correspondingly, in double SSG 27.79 $Pcc21'$ – the SSG generated by adding $\{\mathcal{T}|\mathbf{0}\}$ to double MSG 27.81 $Pc'c'2$ – the double SI group is trivial.

y. Double SIs in Type-III Double MSG 41.215 Ab′a′2

The double MSG 41.215 $Ab'a'2$ is generated by $\{E|100\}$, $\{E|0\frac{1}{2}\frac{1}{2}\}$, $\{E|0\frac{1}{2}\bar{\frac{1}{2}}\}$, $\{C_{2z}|\mathbf{0}\}$, and $\{\mathcal{T}m_x|\frac{1}{2}\frac{1}{2}0\}$. The primitive lattice vectors are:

$$\mathbf{a}_1=(1,0,0),\qquad \mathbf{a}_2=(0,\frac{1}{2},\frac{1}{2}),\qquad \mathbf{a}_3=(0,\frac{1}{2},-\frac{1}{2}), \tag{340}$$

and the reciprocal lattice vectors of the primitive cell are:

$$\mathbf{b}_1=2\pi(1,0,0),\qquad \mathbf{b}_2=2\pi(0,1,1),\qquad \mathbf{b}_3=2\pi(0,1,-1). \tag{341}$$

In the conventional (super)cell, the lattice vectors are:

$$\mathbf{a}'_1=(1,0,0),\qquad \mathbf{a}'_2=\mathbf{a}_2+\mathbf{a}_3=(0,1,0),\qquad \mathbf{a}'_3=\mathbf{a}_2-\mathbf{a}_3=(0,0,1). \tag{342}$$

and the reciprocal lattice vectors of the conventional cell are:

$$\mathbf{b}'_1=2\pi(1,0,0),\qquad \mathbf{b}'_2=2\pi(0,1,0),\qquad \mathbf{b}'_3=2\pi(0,0,1). \tag{343}$$

In the analysis below of the double SIs in double MSG 41.215 $Ab'a'2$, we will refer to coordinates in the basis of the conventional cell for consistency with the convention employed in the BCS applications implemented for MTQC.

SI – The double MSG 41.215 $Ab'a'2$ has the SI group $\mathbb{Z}_2$. In the physical basis, the $\mathbb{Z}_2$ SI has the SI formula:

$$z_{2R}=n_\Gamma^{\frac{1}{2}} \bmod 2, \tag{344}$$

where $n_{\Gamma}^{\frac{1}{2}}$ is the number of occupied states with $\{C_{2z}|\mathbf{0}\}$ eigenvalues $-i$ at Γ. Below, we will demonstrate that $z_{2R} = C_z \bmod 2$ where C_z is the total position-space Chern number in the primitive cell (or equivalently z_{2R} indicates the even-valued Chern number C_z in the conventional cell modulo 4), such that insulators in double MSG 41.215 $Ab'a'2$ with $z_{2R} = 1$ are 3D QAH states.

Layer constructions – To diagnose the topology associated to $z_{2R} = 1$, we employ the layer construction method. We begin by placing a $\hat{\mathbf{z}}$-normal Chern insulator with $C_z = 1$ in the $z = 0$ plane. In the conventional cell, the system has $\{\mathcal{T}m_x|\frac{1}{2}\frac{1}{2}0\}$ and $\{C_{2z}|\mathbf{0}\}$ symmetries, as well as the conventional-cell translation symmetries $\{E|100\}$ and $\{E|010\}$. Because a minimal Chern insulator has one occupied band[156], then, in the conventional supercell – which is twice as large as the primitive cell – the system has two occupied bands. Below, we will demonstrate that a set of occupied bands compatible with this layer construction exhibits $z_{2R} = 1$.

We next determine the constraints imposed by symmetry on the occupied $\{C_{2z}|\mathbf{0}\}$ eigenvalues at the momenta $\Gamma(0,0,0)$, $Z(\pi,0,0)$, $(0,\pi,0)$, and $(\pi,\pi,0)$ [where we note that $(0,\pi,0)$ and $(\pi,\pi,0)$ are not high-symmetry points in MSG 41.215 $Ab'a'2$, see MKVEC (SN 12)]. Because $\{\mathcal{T}m_x|\frac{1}{2}\frac{1}{2}0\}^2 = \{E|010\}$, then the matrix representative of $\{\mathcal{T}m_x|\frac{1}{2}\frac{1}{2}0\}$ squares to minus the identity in all small coreps in the $k_y = \pi$ plane, and states in the $k_y = \pi$ plane must be at least [and are in fact exactly] twofold degenerate, whereas states in the $k_y = 0$ plane are not required by $\{\mathcal{T}m_x|\frac{1}{2}\frac{1}{2}0\}$ to be doubly degenerate [and are in fact singly degenerate at $\Gamma(0,0,0)$]. We then consider a Bloch eigenstate $|\psi(k_x,\pi,0)\rangle$ ($k_x \in \{0,\pi\}$) with $\{C_{2z}|\mathbf{0}\}$ eigenvalue $\xi \in \{i,-i\}$, and compute the $\{C_{2z}|\mathbf{0}\}$ eigenvalue of the state $\{Tm_x|\frac{1}{2}\frac{1}{2}0\}|\psi(k_x,\pi,0)\rangle$:

$$\{C_{2z}|\mathbf{0}\}\{Tm_x|\frac{1}{2}\frac{1}{2}0\}|\psi(k_x,\pi,0)\rangle = -\{E|\bar{1}\bar{1}0\}\{Tm_x|\frac{1}{2}\frac{1}{2}0\}\xi|\psi(k_x,\pi,0)\rangle = e^{-ik_x}\xi^*\{Tm_x|\frac{1}{2}\frac{1}{2}0\}|\psi(k_x,\pi,0)\rangle. \quad (345)$$

SEq. (345) implies that doublets at $(\pi,\pi,0)$ consist of Bloch states with the same $\{C_{2z}|\mathbf{0}\}$ eigenvalues, but that the two states in each doublet at $(0,\pi,0)$ have oppositely-signed $\{C_{2z}|\mathbf{0}\}$ eigenvalues. Next, we consider there to be a state $|\psi(k_x,0,0)\rangle$ ($k_x \in \{0,\pi\}$) with $\{C_{2z}|\mathbf{0}\}$ eigenvalue $\xi \in \{i,-i\}$, and compute the $\{C_{2z}|\mathbf{0}\}$ eigenvalue of $\{Tm_x|\frac{1}{2}\frac{1}{2}0\}|\psi(k_x,0,0)\rangle$:

$$\{C_{2z}|\mathbf{0}\}\{Tm_x|\frac{1}{2}\frac{1}{2}0\}|\psi(k_x,0,0)\rangle = -\{E|\bar{1}\bar{1}0\}\{Tm_x|\frac{1}{2}\frac{1}{2}0\}\xi|\psi(k_x,0,0)\rangle = -e^{-ik_x}\xi^*\{Tm_x|\frac{1}{2}\frac{1}{2}0\}|\psi(k_x,0,0)\rangle. \quad (346)$$

SEq. (346) implies that Bloch states at $Z(\pi,0,0)$ are doubly degenerate with complex-conjugate pairs of $\{C_{2z}|\mathbf{0}\}$ eigenvalues $\{i,-i\}$.

We have thus determined that Bloch states at $\Gamma(0,0,0)$ are singly degenerate, Bloch states at $Z(\pi,0,0)$ and $(0,\pi,0)$ are doubly degenerate and have opposite $\{C_{2z}|\mathbf{0}\}$ eigenvalues, and that Bloch states at $(\pi,\pi,0)$ are doubly degenerate and have the same $\{C_{2z}|\mathbf{0}\}$ eigenvalues. Thus, one possible set of occupied $\{C_{2z}|\mathbf{0}\}$ eigenvalues that satisfy the above constraints and the compatibility relations are $(-i,+i)$, $(-i,+i)$, $(-i,+i)$, $(+i,+i)$ at $\Gamma(0,0,0)$, $Z(\pi,0,0)$, $(0,\pi,0)$, $(\pi,\pi,0)$, respectively. Next, we consider the $\{C_{2z}|\mathbf{0}\}$ eigenvalues at the remaining two high-symmetry $\mathbf{k}$ points: $Y(0,2\pi,0)$ and $T(\pi,2\pi,0)$. Because $\mathbf{k}_Y - \boldsymbol{b}_3 = (0,0,2\pi)$, then the occupied states at Y must have the same $\{C_{2z}|\mathbf{0}\}$ rotation eigenvalues as the occupied states at $\Gamma(0,0,0)$ for bands that satisfy the compatibility relations. Next, because $\mathbf{k}_T - \boldsymbol{b}_3 = (\pi,0,2\pi)$, then the occupied states at T must have the same $\{C_{2z}|\mathbf{0}\}$ rotation eigenvalues as the occupied states at $Z(\pi,0,0)$ for bands that satisfy the compatibility relations. In summary, the $\{C_{2z}|\mathbf{0}\}$ eigenvalues of the occupied bands at the high-symmetry $\mathbf{k}$ points are given by:

	$\Gamma(000)$	$Z(\pi 00)$	$Y(0,2\pi,0)$	$T(\pi,2\pi,0)$
$\{C_{2z}\|\mathbf{0}\}$	$-i,+i$	$-i,+i$	$-i,+i$	$-i,+i$

(347)

Using SEq. (344), we determine that the occupied bands have $z_{2R} = 1$. Next, using the established formula for the parity of the Chern numbers in the $k_z = 0,\pi$ planes in terms of $\{C_{2z}|\mathbf{0}\}$ rotation eigenvalues[156,164] [which is equivalent to SEq. (253)], we conclude that $C_{k_z=0,\pi} \bmod 2 = 1$, which is compatible with the layer construction for $z_{2R} = 1$ introduced in the text preceding SEq. (345). Thus, we conclude that insulators with $z_{2R} = 1$ are 3D QAH states with $C_z \bmod 2 = 1$ in the primitive cell.

Lastly, if we impose $\mathcal{T}$ symmetry, then the position-space Chern numbers must vanish, which enforces z'_{2R} to be zero. Correspondingly, in double SSG 41.212 $Aba21'$ – the SSG generated by adding $\{\mathcal{T}|\mathbf{0}\}$ to double MSG 41.215 $Ab'a'2$ – the double SI group is trivial.

z. Double SIs in Type-III Double MSG 54.342 $Pc'c'a$

The double MSG 54.342 $Pc'c'a$ is generated by $\{E|100\}$, $\{E|010\}$, $\{E|001\}$, $\{\mathcal{I}|\mathbf{0}\}$, $\{C_{2z}|\frac{1}{2}00\}$, and $\{\mathcal{T}m_y|00\frac{1}{2}\}$.

SIs – The double MSG 54.342 $Pc'c'a$ has the SI group $\mathbb{Z}_2\times\mathbb{Z}_2$. In the physical basis, the double SIs of double MSG 54.342 $Pc'c'a$ (η'_{2I}, z'_{2R}) individually subduce to previously introduced double SIs. First, the $\mathcal{I}$ AXI index η'_{2I} subduces to the non-minimal index $(\eta'_{2I})_{P\bar{1}}$ in double MSG 2.4 $P\bar{1}$ (see SN 31 a). Next, the even Chern number SI $2z'_{2R}=C_{k_z=\pi}$ mod 4 subduces to the same SI $(z'_{2R})_{Pc'c'2}$ in double MSG 27.81 $Pc'c'2$ [see SEq. (338) and the surrounding text]. Hence, as we will show below, an insulator with $(\eta'_{2I}, z'_{2R})=(10)$ in double MSG 54.342 $Pc'c'a$ is an $\mathcal{I}$-protected AXI if the non-symmetry-indicated Chern numbers vanish, and an insulator with $z'_{2R}=1$ is a 3D QAH state with C_z mod $4=2$.

Layer constructions – We find that all of the double SIs in double MSG 54.342 $Pc'c'a$ can be realized by layer constructions. The layer constructions for the double SIs (η'_{2I}, z'_{2R}) in double MSG 54.342 $Pc'c'a$ are given by:

1. A $\hat{\mathbf{z}}$-normal Chern layer with $C_z=1$ at the $z=0$ has the SIs (11). We emphasize that, in this layer construction, there is also a $C_z=1$ Chern layer in the $z=\frac{1}{2}$ plane implied by the $\{\mathcal{T}m_y|00\frac{1}{2}\}$ symmetry operation. This layer construction is a 3D QAH state with $C_{k_z}=2$ in all BZ planes of constant k_z.

2. An $\hat{\mathbf{x}}$-normal Chern layer with $C_x=1$ at the $x=0$ plane has the SIs (10). We emphasize that, in this layer construction, there is also a $C_x=-1$ Chern layer in the $x=\frac{1}{2}$ plane implied by the $\{C_{2z}|\frac{1}{2}00\}$ symmetry. Because this layer construction consists of layers with alternating odd Chern numbers occupying $\mathcal{I}$ centers, then this layer construction is an $\mathcal{I}$-protected AXI (see SN 31 a and SRefs. 28,38,40–60).

Relationship with the SIs in other double SSGs – The SIs in double MSG 54.342 $Pc'c'a$ are related to the SIs in double MSG 2.4 $P\bar{1}$ through the subduction relations:

$$(\eta'_{2I}, z'_{2R})_{Pc'c'a}\rightarrow(\eta_{4I}, z_{2I,1}, z_{2I,2}, z_{2I,3})_{P\bar{1}}=(2\eta'_{2I}, 000)_{P\bar{1}}. \tag{348}$$

Lastly, we study the effects of imposing $\mathcal{T}$ symmetry. The double SSG 54.338 $Pcca1'$ – the SSG generated by adding $\{\mathcal{T}|\mathbf{0}\}$ symmetry to double MSG 54.342 $Pc'c'a$ – has the SI group $\mathbb{Z}_4\times\mathbb{Z}_2$[26]. The SIs in double SSG 54.338 $Pcca1'$ are related to the SIs in double MSG 54.342 $Pc'c'a$ through the subduction relations:

$$(z_4, z_{2w,2})_{Pcca1'}\rightarrow(\eta'_{2I}, z'_{2R})_{Pc'c'a}=(z_4 \bmod 2, 0)_{Pc'c'a}. \tag{349}$$

aa. Double SIs in Type-III Double MSG 56.369 $Pc'c'n$

The double MSG 56.369 $Pc'c'n$ is generated by $\{E|100\}$, $\{E|010\}$, $\{E|001\}$, $\{\mathcal{I}|\mathbf{0}\}$, $\{C_{2z}|\frac{1}{2}\frac{1}{2}0\}$, and $\{\mathcal{T}m_y|0\frac{1}{2}\frac{1}{2}\}$.

SIs – The double MSG 56.369 $Pc'c'n$ has the SI group $\mathbb{Z}_2\times\mathbb{Z}_2$. In the physical basis, the double SIs of double MSG 56.369 $Pc'c'n$ (η'_{2I}, z'_{2R}) individually subduce to previously introduced double SIs. First, the $\mathcal{I}$ AXI index η'_{2I} subduces to the non-minimal index $(\eta'_{2I})_{P\bar{1}}$ in double MSG 2.4 $P\bar{1}$ (see SN 31 a). Next, the even Chern number SI $2z'_{2R}=C_{k_z=\pi}$ mod 4 subduces to the same SI $(z'_{2R})_{Pc'c'2}$ in double MSG 27.81 $Pc'c'2$ [see SEq. (338) and the surrounding text]. Hence, as we will show below, an insulator with $(\eta'_{2I}, z'_{2R})=(10)$ in double MSG 56.369 $Pc'c'n$ is an $\mathcal{I}$-protected AXI if the non-symmetry-indicated Chern numbers vanish, and an insulator with $z'_{2R}=1$ is a 3D QAH state with C_z mod $4=2$.

Layer constructions – We find that all of the double SIs in double MSG 56.369 $Pc'c'n$ can be realized by layer constructions. The layer constructions for the double SIs (η'_{2I}, z'_{2R}) in double MSG 56.369 $Pc'c'n$ are given by:

1. A $\hat{\mathbf{z}}$-normal Chern layer with $C_z=1$ at the $z=0$ has the SIs (11). We emphasize that, in this layer construction, there is also a $C_z=1$ Chern layer in the $z=\frac{1}{2}$ plane implied by the $\{\mathcal{T}m_y|0\frac{1}{2}\frac{1}{2}\}$ symmetry operation. This layer construction is a 3D QAH state with $C_{k_z}=2$ in all BZ planes of constant k_z.

2. An $\hat{\mathbf{x}}$-normal Chern layer with $C_x=1$ at the $x=0$ plane has the SIs (10). We emphasize that, in this layer construction, there is also a $C_x=-1$ Chern layer in the $x=\frac{1}{2}$ plane implied by the $\{C_{2z}|\frac{1}{2}\frac{1}{2}0\}$ symmetry. Because this layer construction consists of layers with alternating odd Chern numbers occupying $\mathcal{I}$ centers, then this layer construction is an $\mathcal{I}$-protected AXI (see SN 31 a and SRefs. 28,38,40–60).

Relationship with the SIs in other double SSGs – The SIs in double MSG 56.369 $Pc'c'n$ are related to the SIs in double MSG 2.4 $P\bar{1}$ through the subduction relations:

$$(\eta'_{2I}, z'_{2R})_{Pc'c'n}\rightarrow(\eta_{4I}, z_{2I,1}, z_{2I,2}, z_{2I,3})_{P\bar{1}}=(2\eta'_{2I}, 000)_{P\bar{1}}. \tag{350}$$

Lastly, we study the effects of imposing $\mathcal{T}$ symmetry. The double SSG 56.366 $Pccn1'$ – the SSG generated by adding $\{\mathcal{T}|\mathbf{0}\}$ symmetry to double MSG 56.369 $Pc'c'n$ – has the SI group $\mathbb{Z}_4$[26]. The SIs in double SSG 56.366 $Pccn1'$ are related to the SIs in double MSG 56.369 $Pc'c'n$ through the subduction relations:

$$(z_4)_{Pccn1'} \to (\eta'_{2I}, z'_{2R})_{Pc'c'n} = (z_4 \bmod 2, 0)_{Pc'c'n}. \tag{351}$$

bb. Double SIs in Type-III Double MSG 60.424 $Pb'cn'$

The double MSG 60.424 $Pb'cn'$ is generated by $\{E|100\}$, $\{E|010\}$, $\{E|001\}$, $\{\mathcal{I}|\mathbf{0}\}$, $\{C_{2y}|00\frac{1}{2}\}$, and $\{\mathcal{T}m_x|\frac{1}{2}\frac{1}{2}0\}$.

SIs – The double MSG 60.424 $Pb'cn'$ has the SI group $\mathbb{Z}_2 \times \mathbb{Z}_2$. In the physical basis, the double SIs of double MSG 60.424 $Pb'cn'$ are (η'_{2I}, z'_{2R}). As previously in double MSGs 54.342 $Pc'c'a$ and 56.369 $Pc'c'n$ (SN 31 z and 31 aa, respectively), η'_{2I} is the $\mathcal{I}$ AXI index, and subduces to the non-minimal index $(\eta'_{2I})_{P\bar{1}}$ in double MSG 2.4 $P\bar{1}$.

However, unlike previously in double MSGs 54.342 $Pc'c'a$ and 56.369 $Pc'c'n$, z'_{2R} does not subduce to a previously introduced minimal double SI. Nevertheless, we will shortly use layer constructions to demonstrate that like in double MSGs 54.342 $Pc'c'a$ and 56.369 $Pc'c'n$, z'_{2R} indicates the value of a Chern number – here the position-space Chern number C_y – modulo 4. Hence, an insulator with $(\eta'_{2I}, z'_{2R}) = (10)$ in double MSG 60.424 $Pb'cn'$ is an $\mathcal{I}$-protected AXI if the non-symmetry-indicated Chern numbers vanish, and an insulator with $z'_{2R} = 1$ is a 3D QAH state with $C_y \bmod 4 = 2$. Using the Smith normal form of the EBR matrix [see SN 28] and the definition of the $\mathbb{Z}_2$ AXI parity index η'_{2I} obtained by subduction onto double MSG 2.4 $P\bar{1}$ [see the text surrounding SEq. (249)], we define the second $\mathbb{Z}_2$ SI in double MSG 60.424 $Pb'cn'$ to be:

$$z'_{2R} = \eta'_{2I} + m(\overline{\Gamma}_3) \bmod 2, \tag{352}$$

where $m(\overline{\mathbf{k}}_i)$ is the multiplicity of the small corep $\overline{\mathbf{k}}_i$ of the little group $G_{\mathbf{k}}$ in the symmetry data vector of the occupied bands [where the symmetry data vector of a group of bands is defined in the text following SEq. (108)].

Layer constructions – We find that all of the double SIs in double MSG 60.424 $Pb'cn'$ can be realized by layer constructions. The layer constructions for the double SIs (η'_{2I}, z'_{2R}) in double MSG 60.424 $Pb'cn'$ are given by:

1. A $\hat{\mathbf{y}}$-normal Chern layer with $C_y = 1$ at the $y = 0$ has the SIs (11). We emphasize that, in this layer construction, there is also a $C_y = 1$ Chern layer in the $y = \frac{1}{2}$ plane implied by the $\{\mathcal{T}m_x|\frac{1}{2}\frac{1}{2}0\}$ symmetry operation. This layer construction is a 3D QAH state with $C_{k_y} = 2$ in all BZ planes of constant k_y.

2. An $\hat{\mathbf{z}}$-normal Chern layer with $C_z = 1$ at the $z = 0$ plane has the SIs (10). We emphasize that, in this layer construction, there is also a $C_z = -1$ Chern layer in the $z = \frac{1}{2}$ plane implied by the $\{C_{2y}|00\frac{1}{2}\}$ symmetry. Because this layer construction consists of layers with alternating odd Chern numbers occupying $\mathcal{I}$ centers, then this layer construction is an $\mathcal{I}$-protected AXI (see SN 31 a and SRefs. 28,38,40–60).

Relationship with the SIs in other double SSGs – The SIs in double MSG 60.424 $Pb'cn'$ are related to the SIs in double MSG 2.4 $P\bar{1}$ through the subduction relations:

$$(\eta'_{2I}, z'_{2R})_{Pb'cn'} \to (\eta_{4I}, z_{2I,1}, z_{2I,2}, z_{2I,3})_{P\bar{1}} = (2\eta'_{2I}, 000)_{P\bar{1}}. \tag{353}$$

Lastly, we study the effects of imposing $\mathcal{T}$ symmetry. The double SSG 60.418 $Pbcn1'$ – the SSG generated by adding $\{\mathcal{T}|\mathbf{0}\}$ symmetry to double MSG 60.424 $Pb'cn'$ – has the SI group $\mathbb{Z}_4$[26]. The SIs in double SSG 60.418 $Pbcn1'$ are related to the SIs in double MSG 60.424 $Pb'cn'$ through the subduction relations:

$$(z_4)_{Pbcn1'} \to (\eta'_{2I}, z'_{2R})_{Pb'cn'} = (z_4 \bmod 2, 0)_{Pb'cn'}. \tag{354}$$

cc. Double SIs in Type-III Double MSG 83.45 $P4'/m$

The double MSG 83.45 $P4'/m$ is generated by $\{E|100\}$, $\{E|010\}$, $\{E|001\}$, $\{\mathcal{I}|\mathbf{0}\}$, and $\{\mathcal{T}C_{4z}|\mathbf{0}\}$. We note that double MSG 83.45 $P4'/m$ additionally contains a mirror symmetry operation: $\{m_z|\mathbf{0}\} = \{\mathcal{I}|\mathbf{0}\}\{\mathcal{T}C_{4z}|\mathbf{0}\}^2$.

SIs – The double MSG 83.45 $P4'/m$ has the SI group $\mathbb{Z}_4 \times \mathbb{Z}_2$. In double-valued small coreps of the little groups at the $\mathcal{I}$- and $\mathcal{T}C_{4z}$-invariant $\mathbf{k}$ points Γ [$\mathbf{k}_\Gamma = (000)$], M [$\mathbf{k}_M = (\pi\pi 0)$], Z [$\mathbf{k}_Z = (00\pi)$], and A [$\mathbf{k}_A = (\pi\pi\pi)$], the matrix representatives of $\{\mathcal{I}|\mathbf{0}\}$, $\{\mathcal{T}C_{4z}|\mathbf{0}\}$, and $\{m_z|\mathbf{0}\}$ commute. Hence, Bloch states $|\psi\rangle$ at the Γ, M, Z, and A points may be simultaneously labeled with both parity ($\{\mathcal{I}|\mathbf{0}\}$) and mirror eigenvalues. Taking $|\psi\rangle$ to be a state at an $\mathcal{I}$- and $\mathcal{T}C_{4z}$-invariant $\mathbf{k}$ point with $\{m_z|\mathbf{0}\}$ eigenvalue i, and parity eigenvalue $\xi \in \{-1, 1\}$, we next compute the

$\{m_z|\mathbf{0}\}$ eigenvalues of the state $\{\mathcal{T}C_{4z}|\mathbf{0}\}|\psi\rangle$:

$$\{m_z|\mathbf{0}\}\{\mathcal{T}C_{4z}|\mathbf{0}\}|\psi\rangle = \{\mathcal{T}C_{4z}|\mathbf{0}\}\{m_z|\mathbf{0}\}|\psi\rangle = -i\{\mathcal{T}C_{4z}|\mathbf{0}\}|\psi\rangle, \tag{355}$$

and the $\{\mathcal{I}|\mathbf{0}\}$ eigenvalues of $\{\mathcal{T}C_{4z}|\mathbf{0}\}|\psi\rangle$:

$$\{\mathcal{I}|\mathbf{0}\}\{\mathcal{T}C_{4z}|\mathbf{0}\}|\psi\rangle = \{\mathcal{T}C_{4z}|\mathbf{0}\}\{\mathcal{I}|\mathbf{0}\}|\psi\rangle = \xi\{\mathcal{T}C_{4z}|\mathbf{0}\}|\psi\rangle. \tag{356}$$

SEqs. (355) and (356) imply that the Bloch states at Γ, M, Z, and A form doublets with complex-conjugate $\{m_z|\mathbf{0}\}$ eigenvalues and the same parity eigenvalues. At the $\mathcal{I}$-invariant $\mathbf{k}$ points X [$\mathbf{k}_X = (0\pi0)$], XA [$\mathbf{k}_{XA} = (\pi00)$], R [$\mathbf{k}_R = (0\pi\pi)$], and RA [$\mathbf{k}_{RA} = (\pi0\pi)$] at which $\{\mathcal{T}C_{4z}|\mathbf{0}\}$ is not an element of the little group $G_\mathbf{k}$, Bloch states are instead singly degenerate (see the output of the Corepresentations tool introduced in this work for the double-valued small coreps of double MSG 83.45 $P4'/m$, where Corepresentations is detailed in SN 13). However, the insulating compatibility relations require that there is always an even number of singly-degenerate occupied Bloch states at X, XA, R, and RA, which are required to subdivide into pairs of states (at different energies) with complex-conjugate $\{m_z|\mathbf{0}\}$ eigenvalues and the same parity eigenvalues (see the output of the MCOMPREL tool introduced in this work for the double-valued small coreps of double MSG 83.45 $P4'/m$, where MCOMPREL is detailed in SN 16).

Therefore, like in other centrosymmetric SSGs in which insulators with nontrivial SIs have even numbers of occupied bands that subdivide at each $\mathcal{I}$-invariant $\mathbf{k}$ point into doublets with the same parity eigenvalues [*e.g.* double MSG 47.249 $Pmmm$ and double SSG 2.5 $P\bar{1}1'$, see SN 31 d and 31 s, respectively], the double SIs of MSG 83.45 $P4'/m$ in the physical basis $(z_4, z_{2w,3})$ have the respective SI formulas:

$$z_4 = \sum_K \frac{1}{2} n_K^- = \sum_K \frac{n_K^- - n_K^+}{4} \bmod 4, \tag{357}$$

$$z_{2w,3} = \sum_{K,K_3=\pi} \frac{1}{2} n_K^- = \sum_{K,K_3=\pi} \frac{n_K^- - n_K^+}{4} \bmod 2, \tag{358}$$

where K runs over the eight $\mathcal{I}$-invariant momenta in the first BZ, and $n_K^\pm$ are the number of Bloch states with ±1 parity eigenvalues at K in the group of bands under consideration. Like in double MSG 47.249 $Pmmm$ (see SN 31 d), insulators with $z_4 \bmod 2 = 1$ are TCIs with $\theta = \pi$ (specifically AXIs with the same $C_{4z} \times \mathcal{T}$-symmetric configuration of chiral hinge states as the magnetic HOTIs introduced in SRef. 34), $z_{2w,3}$ indicates the mirror Chern number in the $k_z = \pi$ plane modulo 2 ($z_{2w,3} = C^+_{k_z=\pi} \bmod 2 = C^-_{k_z=\pi} \bmod 2$), and the double SIs $(z_4, z_{2w,3}) = (20)$ indicate a helical (non-axionic) magnetic mirror TCI with $C_{m_z} \bmod 4 = 2$.

Layer constructions – To diagnose the topology associated to each nontrivial value of the double SIs, we employ the layer construction method. In the layer constructions below, $C^+ = -C^-$ due to the net-zero Chern numbers enforced by the symmetries $\{\mathcal{T}C_{4z}|\mathbf{0}\}$ and $\{m_z|\mathbf{0}\}$. Hence, we will omit C^- in further discussions of the topology in double MSG 83.45 $P4'/m$. The layer constructions for the double SIs $(z_4, z_{2w,3})$ of MSG 83.45 $P4'/m$ are given by:

1. A $\hat{\mathbf{z}}$-normal mirror Chern layer with $C_z^+ = 1$ in the $z = 0$ plane has the mirror sector Chern numbers $(C^+_{k_z=0}, C^+_{k_z=\pi}) = (11)$ and the SIs (21).

2. A $\hat{\mathbf{z}}$-normal mirror Chern layer with $C_z^+ = 1$ in the $z = \frac{1}{2}$ plane has the mirror sector Chern numbers $(C^+_{k_z=0}, C^+_{k_z=\pi}) = (1, -1)$ and the SIs (01).

Axion insulators – We find that states with odd z_4 SIs cannot be constructed from layers of 2D stable topological phases. However, we may still use subduction relations to determine the bulk topology of insulators with odd values of z_4. First, as we will show below, (10) and (30) subduce to $(2000)_{P\bar{1}}$ in MSG 2.4 $P\bar{1}$. Hence, if the (10) and (30) phases in double MSG 83.45 $P4'/m$ are insulating, then the bulk insulator must either be an AXI or a 3D QAH state. Because the net Chern numbers $C_{x,y,z} = 0$ must vanish if the bulk is gapped, due to the symmetries $\{\mathcal{T}C_{4z}|\mathbf{0}\}$ and $\{m_z|\mathbf{0}\}$ of double MSG 83.45 $P4'/m$, then the (10) and (30) states must be AXIs. As we will show below, this result can also be understood by subducing from a $\mathcal{T}$-symmetric SSG. Specifically, because $z_4 \bmod 2 = 1$, $z_{2w,3} = 0$ states in MSG 83.45 $P4'/m$ can respectively be subduced from insulators with $z_8 \bmod 2 = 1$, $z^-_{4m,\pi} = z_{2w,1} = 0$ in Type-II double SG 83.44 $P4/m1'$, which correspond to $\mathcal{T}$-symmetric 3D TIs with $\theta = \pi$ (see SN 31 t and SRefs. 24–27), then (10) and (30) are compatible with bulk-gapped states in double MSG 83.45 $P4'/m$. Hence, we conclude that 3D insulators with (10) and (30) in double MSG 83.45 $P4'/m$ are AXIs, *without ambiguity*. We conjecture that the (10) and (30) AXIs in double MSG 83.45 $P4'/m$ can be constructed using the topological crystal method[168], which additionally incorporates cell complexes of 2D Chern insulators, TIs, and TCIs.

Relationship with the SIs in other double SSGs – The SIs in double MSG 83.45 $P4'/m$ are related to the SIs in double MSG 2.4 $P\bar{1}$ through the subduction relations:

$$(z_4, z_{2w,3})_{P4'/m} \to (\eta_{4I}, z_{2I,1}, z_{2I,2}, z_{2I,3})_{P\bar{1}} = (2(z_4 \bmod 2), 000)_{P\bar{1}}. \tag{359}$$

Lastly, we study the effects of imposing $\mathcal{T}$ symmetry. The double SSG 83.44 $P4/1m1'$ – the SSG generated by adding $\{\mathcal{T}|\mathbf{0}\}$ symmetry to double MSG 83.45 $P4'/m$ – has the SI group $\mathbb{Z}_8\times\mathbb{Z}_4\times\mathbb{Z}_2$ (see SN 31 t and SRefs. 24–27). The SIs in double SSG 83.44 $P4/1m1'$ are related to the SIs in double MSG 83.45 $P4'/m$ through the subduction relations:

$$(z_8, z_{4m,\pi}^{-}, z_{2w,1})_{P4/m1'} \to (z_4, z_{2w,3})_{P4'/m} = (z_8 \bmod 4, z_{4m,\pi}^{-} \bmod 2)_{P4'/m}. \tag{360}$$

dd. Double SIs in Type-III Double MSG 103.199 $P4c'c'$

The double MSG 103.199 $P4c'c'$ is generated by $\{E|100\}$, $\{E|010\}$, $\{E|001\}$, $\{C_{4z}|\mathbf{0}\}$, and $\{\mathcal{T}m_y|00\frac{1}{2}\}$.

SI – The double MSG 103.199 $P4c'c'$ has the SI group $\mathbb{Z}_4$. As we will shortly demonstrate, in the physical basis, the double SI z'_{4R} indicates the *even-valued* Chern number in the $k_z=\pi$ plane (modulo 8): $C_{k_z=\pi} \bmod 8 = 2z'_{4R}$. Hence, insulators with nontrivial values of z'_{4R} are 3D QAH states.

We first emphasize that Bloch states at the $\{C_{4z}|\mathbf{0}\}$-invariant momenta in the $k_z=\pi$ plane in double MSG 103.199 $P4c'c'$ form doubly-degenerate pairs with the same $\{C_{4z}|\mathbf{0}\}$ eigenvalues. Specifically, in the $k_z=\pi$ plane, the matrix representative of $\{\mathcal{T}m_y|00\frac{1}{2}\}$ squares to minus the identity in all double-valued small coreps. Furthermore, using the Corepresentations tool introduced in this work (detailed in SN 13), we determine that, in all of $\{\mathcal{T}m_y|00\frac{1}{2}\}$-paired doublets at the $\{C_{4z}|\mathbf{0}\}$-invariant $\mathbf{k}$ points $k_x=k_y=0,\pi$ in the $k_z=\pi$ plane, both states have the same $\{C_{4z}|\mathbf{0}\}$ (and $\{C_{2z}|\mathbf{0}\}$) eigenvalues. Additionally, using the output of Corepresentations for the double-valued small coreps of double MSG 103.199 $P4c'c'$, we find that, at the $\{C_{4z}|\mathbf{0}\}$-invariant $\mathbf{k}$ points $(k_x,k_y)=(0\pi)$ and $(\pi 0)$, both of the Bloch states within each doublet have the same $\{C_{2z}|\mathbf{0}\}$ eigenvalues.

We therefore define the $\mathbb{Z}_4$ SI to be half of the even-valued Chern number (modulo 4) of the occupied bands in the $k_z=\pi$ plane:

$$z'_{4R} = \sum_{K=Z,A}\left(-\frac{1}{4}n_K^{\frac{1}{2}} + \frac{1}{4}n_K^{-\frac{1}{2}} - \frac{3}{4}n_K^{\frac{3}{2}} + \frac{3}{4}n_K^{-\frac{3}{2}}\right) + \frac{1}{2}n_R^{\frac{1}{2}} - \frac{1}{2}n_R^{-\frac{1}{2}} \bmod 4 = \frac{C_{k_z=\pi}}{2} \bmod 4. \tag{361}$$

where $n_{Z,A}^{\frac{1}{2},-\frac{1}{2},\frac{3}{2},-\frac{3}{2}}$ are the number of occupied states with $\{C_{4z}|\mathbf{0}\}$ eigenvalues $e^{-i\frac{\pi}{4}}$, $e^{i\frac{\pi}{4}}$, $e^{-i\frac{3\pi}{4}}$, $e^{i\frac{3\pi}{4}}$, respectively, and $n_R^{\frac{1}{2},-\frac{1}{2}}$ are the number of occupied states with $\{C_{2z}|\mathbf{0}\}$ eigenvalues $e^{-i\frac{\pi}{2}}$, $e^{i\frac{\pi}{2}}$, respectively.

Layer constructions – To diagnose the topology associated to nontrivial values of z'_{4R}, we employ the layer construction method. We begin by placing a $\hat{\mathbf{z}}$-normal Chern layer with $C_z=1$ in the $z=0$ plane. Due to the $\{\mathcal{T}m_y|00\frac{1}{2}\}$ symmetry in double MSG 103.199 $P4c'c'$, there must be another Chern layer with $C_z=1$ in the $z=\frac{1}{2}$ plane, such that the total Chern number per cell is $C_z=2$, and the Chern number of the occupied bands in the $k_z=\pi$ plane is $C_{k_z=\pi}=2$. Hence, in this layer construction of a 3D QAH state, $C_z=2$ and $z'_{4R}=1$.

Relationship with the SIs in other double SSGs – We next compute the subduction relations between the SIs in double MSG 103.199 $P4c'c'$ and the SIs in the maximal unitary subgroup double MSG 75.1 $P4$ (see SN 31 e):

$$(z'_{4R})_{P4c'c'} \to (z_{4R})_{P4} = (2(z'_{4R} \bmod 2))_{P4}. \tag{362}$$

SEq. (362) implies that symmetry-indicated 3D QAH states with $z'_{4R} \bmod 2 = 1$ in double MSG 103.199 $P4c'c'$ subduce to symmetry-indicated 3D QAH states with $(z_{4R})_{P4}=2$ in double MSG 75.1 $P4$, whereas symmetry-indicated 3D QAH states with $z'_{4R}=2$ in double MSG 103.199 $P4c'c'$ necessarily subduce to *non-symmetry-indicated* 3D QAH states with $(z_{4R})_{P4}=0$ in double MSG 75.1 $P4$, in agreement with the physical-basis double SI relations $C_{k_z=\pi} \bmod 8 = 2z'_{4R}$ and $C_{k_z=\pi} \bmod 4 = z_{4R}$ [see SEq. (263) and the surrounding text].

Lastly, if we impose $\mathcal{T}$ symmetry, then the position-space Chern numbers must vanish, which enforces z'_{4R} to be zero. Correspondingly, in double SSG 103.196 $P4cc1'$ – the SSG generated by adding $\{\mathcal{T}|\mathbf{0}\}$ to double MSG 103.199 $P4c'c'$ – the double SI group is trivial.

ee. Double SIs in Type-III Double MSG 110.249 $I4_1c'd'$

The double MSG 110.249 $I4_1c'd'$ is generated by $\{E|\bar{\frac{1}{2}}\frac{1}{2}\frac{1}{2}\}$, $\{E|\frac{1}{2}\bar{\frac{1}{2}}\frac{1}{2}\}$, $\{E|\frac{1}{2}\frac{1}{2}\bar{\frac{1}{2}}\}$, $\{C_{4z}|0\frac{1}{2}\frac{1}{4}\}$, and $\{\mathcal{T}m_x|\frac{1}{2}\frac{1}{2}0\}$. The Bravais lattice of double MSG 110.249 $I4_1c'd'$ is body-centered. Correspondingly, in the primitive cell, the lattice vectors are:

$$\mathbf{a}_1 = (-\frac{1}{2}, \frac{1}{2}, \frac{1}{2}), \quad \mathbf{a}_2 = (\frac{1}{2}, -\frac{1}{2}, \frac{1}{2}), \quad \mathbf{a}_3 = (\frac{1}{2}, \frac{1}{2}, -\frac{1}{2}), \tag{363}$$

and the reciprocal lattice vectors are:

$$\mathbf{b}_1 = 2\pi(0, 1, 1), \quad \mathbf{b}_2 = 2\pi(1, 0, 1), \quad \mathbf{b}_3 = 2\pi(1, 1, 0). \tag{364}$$

We additionally note that $\{C_{4z}|0\frac{1}{2}\frac{1}{4}\}^2 = \{C_{2z}|\frac{1}{2}\frac{1}{2}\frac{1}{2}\} = \{E|\frac{1}{2}\frac{1}{2}\frac{1}{2}\}\{C_{2z}|\mathbf{0}\}$, where $\{E|\frac{1}{2}\frac{1}{2}\frac{1}{2}\}$ is a primitive translation symmetry. Hence, the 4_1 screw symmetry operation $\{C_{4z}|0\frac{1}{2}\frac{1}{4}\}$ only contains a *half* lattice translation in the z direction in the primitive cell, such that double MSG 110.249 $I4_1c'd'$ also contains the rotation symmetry $\{C_{2z}|\mathbf{0}\}$.

SI – The double MSG 110.249 $I4_1c'd'$ has the SI group $\mathbb{Z}_2$. In the physical basis, the $\mathbb{Z}_2$ SI has the SI formula:

$$z''_{2R} = m(\overline{\Gamma}_6) \bmod 2 = \frac{C_{k_z=0}}{2} \bmod 2, \tag{365}$$

where $m(\overline{\Gamma}_6)$ is the multiplicity of the double-valued small corep $\overline{\Gamma}_6$ in the symmetry data $\tilde{\varsigma}_\Gamma$ corresponding to the occupied states at Γ [$\mathbf{k}_\Gamma = (0, 0, 0)$], where the symmetry data at a $\mathbf{k}$ point is defined in the text following SEq. (108), and where the $\{C_{4z}|0\frac{1}{2}\frac{1}{4}\}$ and $\{C_{2z}|\mathbf{0}\}$ characters of the irreducible small coreps $\tilde{\sigma}$ at Γ are given by:

	$\overline{\Gamma}_5$	$\overline{\Gamma}_6$	$\overline{\Gamma}_7$	$\overline{\Gamma}_8$
$\chi_{\tilde{\sigma}}(\{C_{4z}\|0\frac{1}{2}\frac{1}{4}\})$	$e^{i\frac{3\pi}{4}}$	$e^{-i\frac{\pi}{4}}$	$e^{-i\frac{3\pi}{4}}$	$e^{i\frac{\pi}{4}}$
$\chi_{\tilde{\sigma}}(\{C_{2z}\|\mathbf{0}\})$	$-i$	$-i$	i	i

(366)

Hence, as we will show below, insulators in double MSG 110.249 $I4_1c'd'$ with $z''_{2R} = 1$ are 3D QAH states with $C_z \bmod 4 = 2$ per primitive cell.

Layer constructions – To diagnose the topology associated to $z''_{2R} = 1$, we employ the layer construction method. We begin by placing a $\hat{\mathbf{z}}$-normal Chern insulator with $C_z = 1$ in the $z = 0$ plane. Due to the $\{C_{4z}|0\frac{1}{2}\frac{1}{4}\}$ screw symmetry in double MSG 110.249 $I4_1c'd'$, there must be a second $\hat{\mathbf{z}}$-normal Chern insulator with $C_z = 1$ in the $z = \frac{1}{4}$ plane. Using the established formula for the parity of the Chern number in terms of $\{C_{2z}|\mathbf{0}\}$ rotation eigenvalues[156,164] and the constraints imposed by the compatibility relations on the eigenvalues of the 4_1 screw symmetry $\{C_{4z}|0\frac{1}{2}\frac{1}{4}\}$ in an insulating state (see the output of the MCOMPREL tool introduced in this work for the double-valued small coreps of double MSG 110.249 $I4_1c'd'$, where MCOMPREL is detailed in SN 16), we find that a set of symmetry data compatible with this layer construction is given by $\tilde{\varsigma}_\Gamma = \overline{\Gamma}_5 + \overline{\Gamma}_6 + \overline{\Gamma}_7 + \overline{\Gamma}_8$. Hence, $z''_{2R} = 1$ in this layer construction of a 3D QAH state, in agreement with the net position-space Chern number $C_z = 2$ per primitive cell.

Lastly, if we impose $\mathcal{T}$ symmetry, then the position-space Chern numbers must vanish, which enforces z'_{2R} to be zero. Correspondingly, in double SSG 110.246 $I4_1cd1'$ – the SSG generated by adding $\{\mathcal{T}|\mathbf{0}\}$ to double MSG 110.249 $I4_1c'd'$ – the double SI group is trivial.

ff. Double SIs in Type-III Double MSG 130.429 $P4/nc'c'$

The double MSG 130.429 $P4/nc'c'$ is generated by $\{E|100\}$, $\{E|010\}$, $\{E|001\}$, $\{C_{4z}|\frac{1}{2}00\}$, $\{\mathcal{I}|\mathbf{0}\}$, and $\{\mathcal{T}m_{1\bar{1}0}|00\frac{1}{2}\}$.

SIs – The double MSG 130.429 $P4/nc'c'$ has the SI group $\mathbb{Z}_4 \times \mathbb{Z}_2$. In the physical basis, the double SIs of double MSG 130.429 $P4/nc'c'$ (z'_{4R}, η'_{2I}) individually subduce to previously introduced double SIs. First, the even Chern number SI $2z'_{4R} = C_{k_z=\pi} \bmod 8$ subduces to the same SI $(z'_{4R})_{P4c'c'}$ in double MSG 103.199 $P4c'c'$ [see SEq. (361) and the surrounding text]. Next, the $\mathcal{I}$ AXI index η'_{2I} subduces to the non-minimal index $(\eta'_{2I})_{P\bar{1}}$ in double MSG 2.4 $P\bar{1}$ (see SN 31 a). Hence, as we will show below, an insulator with $(z'_{4R}, \eta'_{2I}) = (01)$ in double MSG 130.429 $P4/nc'c'$ is an $\mathcal{I}$-protected AXI if the non-symmetry-indicated Chern numbers vanish, and insulators with $z'_{4R} \neq 0$ are 3D QAH states.

Layer constructions – We find that in double MSG 130.429 $P4/nc'c'$, the 3D QAH states – but not the AXI states – can be realized by layer constructions. The double SIs (z'_{4R}, η'_{2I}) of the symmetry-indicated 3D QAH states in double MSG 130.429 $P4/nc'c'$ are spanned by superpositions of the following layer construction:

1. A $\hat{\mathbf{z}}$-normal Chern layer with $C_z = 1$ at the $z = 0$ has the SIs (11). We emphasize that, in this layer construction, there is also a $C_z = 1$ Chern layer in the $z = \frac{1}{2}$ plane implied by the $\{\mathcal{T}m_{1\bar{1}0}|00\frac{1}{2}\}$ symmetry operation. This layer construction is a 3D QAH state with $C_{k_z} = 2$ in all BZ planes of constant k_z.

Axion insulators and 3D QAH states – We find that states with the double SIs $(z'_{4R}, \eta'_{2I}) = (01)$ in double MSG 130.429 $P4/nc'c'$ cannot be constructed from layers of 2D stable topological phases. However, we may still use subduction relations to determine the bulk topology of insulators with the double SIs (01). First, as we will show below, (01) subduces to $(2000)_{P\bar{1}}$ in MSG 2.4 $P\bar{1}$. Hence, if a (01) state in double MSG 130.429 $P4/nc'c'$ is insulating, then the bulk insulator must either be an AXI or a 3D QAH state, and will specifically be an AXI if the non-symmetry-indicated Chern numbers vanish. As we will show below, this result can also be understood by subducing from a $\mathcal{T}$-symmetric SSG. Specifically, because (01) states in double MSG 130.429 $P4/nc'c'$ can be subduced from insulators with $(z_4)_{P4/ncc1'}$ mod $2 = 1$ in Type-II double SG 130.424 $P4/ncc1'$, which correspond to $\mathcal{T}$-symmetric 3D TIs with $\theta = \pi$[26], then the double SIs (01) are compatible with a bulk-gapped state in double MSG 130.429 $P4/nc'c'$. This provides further evidence that 3D insulators with (01) and net-zero position-space Chern numbers in double MSG 130.429 $P4/nc'c'$ are AXIs. We conjecture that (01) AXIs in double MSG 130.429 $P4/nc'c'$ can be constructed using the topological crystal method[168], which additionally incorporates cell complexes of 2D Chern insulators, TIs, and TCIs.

Relationship with the SIs in other double SSGs – The SIs in double MSG 130.429 $P4/nc'c'$ are related to the SIs in double MSG 2.4 $P\bar{1}$ through the subduction relations:

$$(z'_{4R}, \eta'_{2I})_{P4/nc'c'} \rightarrow (\eta_{4I}, z_{2I,1}, z_{2I,2}, z_{2I,3})_{P\bar{1}} = (2\eta'_{2I}, 000)_{P\bar{1}}. \tag{367}$$

Lastly, we study the effects of imposing $\mathcal{T}$ symmetry. The double SSG 130.424 $P4/ncc1'$ – the SSG generated by adding $\{\mathcal{T}|\mathbf{0}\}$ symmetry to double MSG 130.429 $P4/nc'c'$ – has the SI group $\mathbb{Z}_4$[26]. The SIs in double SSG 130.424 $P4/ncc1'$ are related to the SIs in double MSG 130.429 $P4/nc'c'$ through the subduction relations:

$$(z_4)_{P4/ncc1'} \rightarrow (z'_{4R}, \eta'_{2I})_{P4/nc'c'} = (0, z_4 \bmod 2)_{P4/nc'c'}. \tag{368}$$

gg. Double SIs in Type-III Double MSG 135.487 $P4'_2/mbc'$

The double MSG 135.487 $P4'_2/mbc'$ is generated by $\{E|100\}$, $\{E|010\}$, $\{E|001\}$, $\{\mathcal{I}|\mathbf{0}\}$, $\{m_z|\mathbf{0}\}$, $\{C_{2x}|\frac{1}{2}\frac{1}{2}0\}$, and $\{\mathcal{T}C_{4z}|00\frac{1}{2}\}$.

SI – The double MSG 135.487 $P4'_2/mbc'$ has the SI group $\mathbb{Z}_4$. At the $\mathcal{I}$-invariant momenta, the double-valued irreducible small coreps are either two- or four-dimensional. An expression for the SI formula of the $\mathbb{Z}_4$ double SI computed from the Smith normal form of the EBR matrix (see SN 28) is given by:

$$z'_4 = 2m(\overline{\Gamma}_5) - m(\overline{\Gamma}_6) - m(\overline{M}_5) + 2m(\overline{X}_3), \tag{369}$$

where $m(\overline{\mathbf{k}}_i)$ is the multiplicity of the small corep $\overline{\mathbf{k}}_i$ of the little group $G_{\mathbf{k}}$ in the symmetry data vector of the occupied bands [where the symmetry data vector of a group of bands is defined in the text following SEq. (108)]. As we will shortly show below through layer constructions, like in double MSGs 47.249 $Pmmm$ and 83.45 $P4'/m$, insulators with z'_4 mod $2 = 1$ are TCIs with $\theta = \pi$ (*i.e.* AXIs), and $z'_4 = 2$ indicates a helical (non-axionic) magnetic mirror TCI with C_{m_z} mod $4 = 2$.

Layer constructions – We find that in double MSG 135.487 $P4'_2/mbc'$, the non-axionic magnetic TCI phases – but not the AXI phases – can be realized by layer constructions. The double SI $z'_4 = 2$ of a symmetry-indicated non-axionic TCI phase in $P4'_2/mbc'$ with $C_{m_z} = 2$ is realized by the following layer construction:

1. A $\hat{\mathbf{z}}$-normal mirror Chern layer with $C_z^+ = -C_z^- = 1$ in the $z = 0$ plane has the double SI $z'_4 = 2$. We emphasize that, in this layer construction, there is also a $\hat{\mathbf{z}}$-normal mirror Chern layer with $C_z^+ = -C_z^- = 1$ in the $z = \frac{1}{2}$ plane implied by the $\{\mathcal{T}C_{4z}|00\frac{1}{2}\}$ symmetry operation.

Axion insulators – We find that states with odd z'_4 SIs cannot be constructed from layers of 2D stable topological phases. However, we may still use subduction relations to determine the bulk topology of insulators with odd values of z'_4. First, as we will show below, the double SIs $z'_4 = 1, 3$ in double MSG 135.487 $P4'_2/mbc'$ subduce to $(2000)_{P\bar{1}}$ in MSG 2.4 $P\bar{1}$. Hence, if the z'_4 mod $2 = 1$ phases in double MSG 135.487 $P4'_2/mbc'$ are insulating, then the bulk insulator must either be an AXI or a 3D QAH state. Because the net Chern numbers $C_{x,y,z} = 0$ must vanish if the bulk is gapped, due to the symmetries $\{m_z|\mathbf{0}\}$ and $\{C_{2x}|\frac{1}{2}\frac{1}{2}0\}$ of double MSG 135.487 $P4'_2/mbc'$, then the $z'_4 = 1, 3$ states must be AXIs. As we will show below, this result can also be understood by subducing from a $\mathcal{T}$-symmetric SSG.

Specifically, because $z'_4 \bmod 2 = 1$ states in MSG 135.487 $P4'_2/mbc'$ can respectively be subduced from insulators with $z_4 = 1,3$ in Type-II double SG 135.484 $P4_2/mbc1'$, which correspond to $\mathcal{T}$-symmetric 3D TIs with $\theta = \pi$[26], then the double SIs $z'_4 = 1,3$ are compatible with bulk-gapped states in double MSG 135.487 $P4'_2/mbc'$. Hence, we conclude that 3D insulators with $z'_4 \bmod 2 = 1$ in double MSG 135.487 $P4'_2/mbc'$ are AXIs, *without ambiguity*. We conjecture that the $z'_4 = 1,3$ AXIs in double MSG 135.487 $P4'_2/mbc'$ can be constructed using the topological crystal method[168], which additionally incorporates cell complexes of 2D Chern insulators, TIs, and TCIs.

Relationship with the SIs in other double SSGs – The SIs in double MSG 135.487 $P4'_2/mbc'$ are related to the SIs in double MSG 2.4 $P\bar{1}$ through the subduction relations:

$$(z'_4)_{P4'_2/mbc'} \rightarrow (\eta_{4I}, z_{2I,1}, z_{2I,2}, z_{2I,3})_{P\bar{1}} = (2(z'_4 \bmod 2), 000)_{P\bar{1}}. \tag{370}$$

Lastly, we study the effects of imposing $\mathcal{T}$ symmetry. The double SSG 135.484 $P4_2/mbc1'$ – the SSG generated by adding $\{\mathcal{T}|\mathbf{0}\}$ symmetry to double MSG 135.487 $P4'_2/mbc'$ – has the SI group $\mathbb{Z}_4$[26]. The SIs in double SSG 135.484 $P4_2/mbc1'$ are in one-to-one correspondence with the SIs in double MSG 135.487 $P4'_2/mbc'$:

$$(z_4)_{P4_2/mbc1'} \rightarrow (z'_4)_{P4'_2/mbc'} = (z_4)_{P4'_2/mbc'}. \tag{371}$$

Nevertheless, because the EBRs in Type-II double SSG 135.484 $P4_2/mbc1'$ and the MEBRs in Type-III double MSG 135.487 $P4'_2/mbc'$ are not in one-to-one correspondence, then we will continue throughout this work to employ separate labels (z_4 and z'_4 respectively) for the double SIs in double SSGs 135.484 $P4_2/mbc1'$ and 135.487 $P4'_2/mbc'$.

hh. Double SIs in Type-III Double MSG 184.195 $P6c'c'$

The double MSG 184.195 $P6c'c'$ is generated by $\{E|100\}$, $\{E|010\}$, $\{E|001\}$, $\{C_{6z}|\mathbf{0}\}$ and $\{\mathcal{T}m_x|00\frac{1}{2}\}$, where the angle between the $\{E|100\}$ and $\{E|010\}$ translations is chosen to be $2\pi/3$ for consistency with the $\{C_{3z}|\mathbf{0}\} = (\{C_{6z}|\mathbf{0}\})^2$ rotation symmetry.

SI – The double MSG 184.195 $P6c'c'$ has the SI group $\mathbb{Z}_6$. As we will shortly demonstrate, in the physical basis, the double SI z'_{6R} indicates the *even-valued* Chern number in the $k_z = \pi$ plane (modulo 12): $C_{k_z=\pi} \bmod 12 = 2z'_{6R}$. Hence, insulators with nontrivial values of z'_{6R} are 3D QAH states.

First, using the Corepresentations tool introduced in this work (detailed in SN 13), we determine that Bloch states at the $\{C_{nz}|\mathbf{0}\}$-invariant ($n = 2,3,6$) $\mathbf{k}$ points in the $k_z = \pi$ plane in double MSG 184.195 $P6c'c'$ form doubly-degenerate pairs with the same $\{C_{nz}|\mathbf{0}\}$ rotation symmetry eigenvalues. We therefore define the $\mathbb{Z}_6$ SI to be half of the even-valued Chern number (modulo 6) of the occupied bands in the $k_z = \pi$ plane:

$$\begin{aligned} z'_{6R} &= \frac{1}{2}\left(-\frac{1}{2}n_A^{\frac{1}{2}} + \frac{1}{2}n_A^{-\frac{1}{2}} - \frac{3}{2}n_A^{\frac{3}{2}} + \frac{3}{2}n_A^{-\frac{3}{2}} - \frac{5}{2}n_A^{\frac{5}{2}} + \frac{5}{2}n_A^{-\frac{5}{2}} - n_H^{\frac{1}{2}} + n_H^{-\frac{1}{2}} + 3n_H^{\frac{3}{2}} + \frac{3}{2}n_L^{\frac{1}{2}} - \frac{3}{2}n_L^{-\frac{1}{2}}\right) \bmod 6 \\ &= \frac{C_{k_z=\pi}}{2} \bmod 6, \end{aligned} \tag{372}$$

where the superscripts n_A^j represent the $\{C_{6z}|\mathbf{0}\}$ eigenvalues $e^{-i\frac{2\pi}{6}j}$ at A, n_H^j is the number of occupied states with $\{C_{3z}|\mathbf{0}\}$ eigenvalue $e^{-i\frac{2\pi}{3}j}$ at H, and where n_L^j is the number of states with $\{C_{2z}|\mathbf{0}\}$ eigenvalue $e^{-i\frac{\pi}{2}j}$ at L.

Layer constructions – To diagnose the topology associated to nontrivial values of z'_{6R}, we employ the layer construction method. We begin by placing a $\hat{\mathbf{z}}$-normal Chern layer with $C_z = 1$ in the $z = 0$ plane. Due to the $\{\mathcal{T}m_x|00\frac{1}{2}\}$ symmetry in double MSG 184.195 $P6c'c'$, there must be another Chern layer with $C_z = 1$ in the $z = \frac{1}{2}$ plane, such that the total Chern number per cell is $C_z = 2$, and the Chern number of the occupied bands in the $k_z = \pi$ plane is $C_{k_z=\pi} = 2$. Hence, in this layer construction of a 3D QAH state, $C_z = 2$ and $z'_{6R} = 1$.

Relationship with the SIs in other double SSGs – We next compute the subduction relations between the SIs in double MSG 184.195 $P6c'c'$ and the SIs in the maximal unitary subgroup double MSG 168.109 $P6$ (see SN 31 n):

$$(z'_{6R})_{P6c'c'} \rightarrow (z_{6R})_{P6} = (2(z'_{6R} \bmod 3))_{P6}. \tag{373}$$

SEq. (373) implies that symmetry-indicated 3D QAH states with $z'_{6R} \bmod 3 \neq 0$ in double MSG 184.195 $P6c'c'$ subduce to symmetry-indicated 3D QAH states with even values of $(z_{6R})_{P6}$ in double MSG 168.109 $P6$, whereas symmetry-indicated 3D QAH states with $z'_{6R} \bmod 3 = 0$ in double MSG 184.195 $P6c'c'$ necessarily subduce to *non-symmetry-indicated* 3D QAH states with $(z_{6R})_{P6} = 0$ in double MSG 168.109 $P6$, in agreement with the physical-basis double SI relations $C_{k_z=\pi} \bmod 12 = 2z'_{6R}$ and $C_{k_z=\pi} \bmod 6 = z_{6R}$ [see SEq. (314) and the surrounding text].

Lastly, if we impose $\mathcal{T}$ symmetry, then the position-space Chern numbers must vanish, which enforces z'_{6R} to be zero. Correspondingly, in double SSG 184.192 $P6cc1'$ – the SSG generated by adding $\{\mathcal{T}|\mathbf{0}\}$ to double MSG 184.195 $P6c'c'$ – the double SI group is trivial.

32. Summary of the Double SIs in the Minimal Double SSGs

In this section, we will summarize and review the results of the minimal double SI calculations performed in SN 31. In Supplementary Table 11, we present a summary of the complete, independent, minimal double SIs of spinful band topology in the 1,651 SSGs. All symmetry-indicated spinful SISM [specifically symmetry-indicated WSM, see the text following SEq. (236)], TI, and TCI phases in crystalline solids necessarily exhibit nontrivial values of at least one of the double SIs listed in Supplementary Table 11.

We note that, in Supplementary Table 11, some minimal double SSGs G are associated to a smaller set of SIs than the SI group Z^G. This occurs because, in some cases, some – but not all – of the double SIs in G have already been established in subgroups M of G (*i.e.*, the double SIs in G are not dependent on the double SIs in M, even though some of the double SIs in G are the same as the double SIs in M, see SN 30 for the definition of dependent SIs). For example, the indicator group of double MSG 147.13 $P\bar{3}$ is $\mathbb{Z}_{12}\times\mathbb{Z}_2$, whereas double MSG 147.13 $P\bar{3}$ is only associated in Supplementary Table 11 to the $\mathbb{Z}_3$-valued index z_{3R}. In the minimal double MSG 147.13 $P\bar{3}$ [SN 31 m], the double SIs (η_{4I}, z_{3R}, $z_{2I,3}$) are not dependent on the double SIs in any individual lower-symmetry double MSG. However, the double SIs η_{4I} and $z_{2I,3}$ also appear in the minimal double MSG 2.4 $P\bar{1}$, where the definitions of η_{4I} and $z_{2I,3}$ [the product of the parity eigenvalues of a set of bands at all of the $\mathcal{I}$-invariant $\mathbf{k}$ points and in the $k_3=\pi$ plane, respectively, see SEq. (235)] is the same in both double MSG 147.13 $P\bar{3}$ and double MSG 2.4 $P\bar{1}$. Correspondingly, when the spinful SI topological bands of double MSG 147.13 $P\bar{3}$ are subduced onto the subgroup double MSG 2.4 $P\bar{1}$, the values of $(\eta_{4I})_{P\bar{3}}$ and $(z_{2I,3})_{P\bar{3}}$ for the spinful SI topological bands of double MSG 147.13 $P\bar{3}$ are the same as the values of $(\eta_{4I})_{P\bar{1}}$ and $(z_{2I,3})_{P\bar{1}}$ for the SI topological bands subduced onto double MSG 2.4 $P\bar{1}$. Hence, double MSG 147.13 $P\bar{3}$ is not associated to η_{4I} or $z_{2I,3}$ in Supplementary Table 11, even though the double SIs (η_{4I}, z_{3R}, $z_{2I,3}$) of double MSG 147.13 $P\bar{3}$ include η_{4I} and $z_{2I,3}$.

Additionally, in Supplementary Table 11, some double SIs are associated to more than one minimal double SSG. This occurs when minimal double SIs that indicate the same bulk topology arise in two minimal double SSGs G and M for which neither $G\not\subset M$ nor $M\not\subset G$. For example, z_4 in Supplementary Table 11 is associated to both double SG 2.5 $P\bar{1}1'$ and double MSG 47.249 $Pmmm$. In both double SG 2.5 $P\bar{1}1'$ and double MSG 47.249 $Pmmm$, $z_4=2$ indicates a non-axionic HOTI phase with helical hinge states through the $\mathbb{Z}_4$-valued parity eigenvalue formula introduced in SRefs. 24,26,27 [reproduced in SEq. (259)]. We will further analyze the $z_4=2$ non-axionic magnetic HOTI phase protected by the symmetries of double MSG 47.249 $Pmmm$ in SN 33.

Independent Minimal Double SIs of Spinful Band Topology in the 1,651 Magnetic and Nonmagnetic Double SSGs		
SI	Bulk Topology	Minimal Double SSG(s) [Double SI Formula(s)]
η_{4I}	WSM/QAH/AXI	2.4 $P\bar{1}$ [SEq. (235)]
$z_{2I,i}$	QAH: $C_{k_i=\pi}$ mod 2	2.4 $P\bar{1}$ [SEq. (236)]
η'_{2I}	AXI	2.4 $P\bar{1}$ [SEq. (249)]
z_{2R}	QAH: C_y mod 2	3.1 $P2$ [SEq. (253)], 41.215 $Ab'a'2$ [SEq. (344)]
δ_{2m}	QAH/AXI/TCI: $C^+_{k_y=\pi} - C^-_{k_y=0}$ mod 2	10.42 $P2/m$ [SEq. (254)]
$z^+_{2m,\pi}$	QAH/weak TI/weak TCI: $C^+_{k_y=\pi}$ mod 2	10.42 $P2/m$ [SEq. (255)]
$z^-_{2m,\pi}$	QAH/weak TI/weak TCI: $C^-_{k_y=\pi}$ mod 2	10.42 $P2/m$ [SEq. (256)]
z_4	AXI/TCI/HOTI	2.5 $P\bar{1}1'$, 47.249 $Pmmm$, 83.45 $P4'/m$ [SEq. (259)]
$z_{2w,i}$	weak TI/weak TCI: $C^+_{k_i=\pi}$ mod 2	2.5 $P\bar{1}1'$, 47.249 $Pmmm$, 83.45 $P4'/m$ [SEq. (260)] (†)
z_{4R}	QAH: C_z mod 4	75.1 $P4$ [SEq. (263)]
z'_{2R}, z''_{2R}	QAH: $C_{y,z}/2$ mod 2	77.13 $P4_2$ [SEq. (264)], 27.81 $Pc'c'2$ [SEq. (338)], 54.342 $Pc'c'a$ [SEq. (338)], 56.369 $Pc'c'n$ [SEq. (338)], 60.424 $Pb'cn'$ [SEq. (352)], 110.249 $I4_1c'd'$ [SEq. (365)] (‡)
z_{4S}	QAH: C_z mod 4	81.33 $P\bar{4}$ [SEq. (266)]
δ_{2S}	WSM	81.33 $P\bar{4}$ [SEq. (269)]
z_2	AXI	81.33 $P\bar{4}$ [SEq. (270)]
δ_{4m}	QAH/AXI: $C^+_{k_z=\pi} - C^-_{k_z=0}$ mod 4	83.43 $P4/m$ [SEq. (272)]
$z^+_{4m,\pi}$	weak TI/weak TCI: $C^+_{k_z=\pi}$ mod 4	83.43 $P4/m$ [SEq. (273)]
$z^-_{4m,\pi}$	weak TI/weak TCI: $C^-_{k_z=\pi}$ mod 4	83.43 $P4/m$ [SEq. (274)]
$z^+_{4m,0}$	QAH/weak TI/weak TCI: $C^+_{k_z=0}$ mod 4	84.51 $P4_2/m$ [SEq. (278)]
z_8	AXI/TCI/HOTI	83.44 $P4/m1'$, 123.339 $P4/mmm$ [SEq. (305)]
z_{3R}	QAH: C_z mod 3	147.13 $P\bar{3}$ [SEq. (310)]
z_{6R}	QAH: C_z mod 6	168.109 $P6$ [SEq. (314)]
δ_{3m}	QAH/AXI/TCI: $C^+_{k_z=\pi} - C^-_{k_z=\pi}$ mod 3	174.133 $P\bar{6}$ [SEq. (315)]
$z^+_{3m,\pi}$	weak TI/weak TCI: $C^+_{k_z=\pi}$ mod 3	174.133 $P\bar{6}$ [SEq. (316)]
$z^-_{3m,\pi}$	weak TI/weak TCI: $C^-_{k_z=\pi}$ mod 3	174.133 $P\bar{6}$ [SEq. (317)]
δ_{6m}	QAH/AXI/TCI: $C^+_{k_z=\pi} - C^-_{k_z=\pi}$ mod 6	175.137 $P6/m$ [SEq. (319)]
$z^+_{6m,\pi}$	weak TI/weak TCI: $C^+_{k_z=\pi}$ mod 6	175.137 $P6/m$ [SEq. (320)]
$z^-_{6m,\pi}$	weak TI/weak TCI: $C^-_{k_z=\pi}$ mod 6	175.137 $P6/m$ [SEq. (321)]
$z^+_{6m,0}$	QAH/weak TI/weak TCI: $C^+_{k_z=0}$ mod 6	176.143 $P6_3/m$ [SEq. (324)]
z_{12}	AXI/TCI/HOTI	175.138 $P6/m1'$, 191.233 $P6/mmm$ [SEq. (327)]
z'_{12}	AXI/TCI/HOTI	176.144 $P6_3/m1'$ [SEq. (336)]
z'_{4R}	QAH: $C_z/2$ mod 4	103.199 $P4c'c'$ [SEq. (361)]
z'_4	AXI/TCI	135.487 $P4'_2/mbc'$ [SEq. (369)]
z'_{6R}	QAH: $C_z/2$ mod 6	184.195 $P6c'c'$ [SEq. (372)]

Supplementary Table 11: The independent minimal double SIs of spinful band topology in all 1,651 double SSGs. In order, this table contains the symbol of each double SI, the bulk topological phase(s) associated to nontrivial values of the double SI including – where applicable – the momentum- or position-space Chern numbers indicated by the double SI, and the minimal double SSG(s) associated to the double SI [*i.e.* the lowest-symmetry SSG(s) in which the double SI predicts nontrivial band topology, see SN 30 and 39], as well as the equation in SN 31 containing the explicit double SI formula in terms of crystal symmetry eigenvalues. All symmetry-indicated spinful SISM [specifically symmetry-indicated WSM, see the text following SEq. (236)], TI, and TCI phases in crystalline solids necessarily exhibit nontrivial values of at least one of the double SIs listed in this table. We note that, in this table, the symbol "AXI" refers to both magnetic AXIs and $\mathcal{T}$-symmetric 3D TIs, because AXI and 3D TI phases are both defined by the nontrivial bulk axion angle $\theta = \pi$[28,32,38,40–60,160]. Additionally, the symbols "TCI" and "HOTI" respectively indicate helical (*i.e.* non-axionic) mirror Chern insulators and HOTIs[24–28,34–36,39,112,161,162,164], which include the magnetic HOTIs introduced in this work (see SN 33). We have placed a † symbol after MSG 83.45 $P4'/m$ in the row for $z_{2w,i}$ to emphasize that, of the three $z_{2w,i}$, only $z_{2w,3}$ is a minimal double SI in MSG 83.45 $P4'/m$ (where minimal double SIs are defined in SN 30). We have placed a ‡ symbol after MSG 110.249 $I4_1c'd'$ in the row for the indices z'_{2R} and z''_{2R} to emphasize that the position-space Chern number C_z (modulo 2) is indicated by z''_{2R} only in the primitive cell of a crystal in MSG 110.249 $I4_1c'd'$ – in the conventional cell, the position-space Chern number is given by C_z mod 8 $= 4z''_{2R}$ [see the text surrounding SEq. (365)].

33. Introduction to Non-Axionic Spinful Magnetic HOTIs

In the sections below, we will further analyze the spinful helical magnetic HOTI phases discovered in this work. As discussed in the main text and in SN 31 and 32, we have discovered helical magnetic (*i.e.* $\{\mathcal{T}|\mathbf{0}\}$-broken) HOTI phases indicated by $z_4 = 2$ in double MSG 47.249 $Pmmm$, $z_8 = 4$ in double MSG 123.339 $P4/mmm$, and $z_{12} = 6$ in double MSG 191.233 $P6/mmm$, as well as trivial values for all other independent minimal double SIs in Supplementary Table 11. In this work, we refer to the helical magnetic HOTIs that will be analyzed in this section as *non-axionic*, because the helical HOTIs exhibit trivial axion angles $\theta \bmod 2\pi = 0$ [see SRefs. 28,32,38,40–60,160 for further discussions of chiral HOTIs (*i.e.* AXIs), which conversely exhibit nontrivial axion angles $\theta = \pi$]. When terminated in nanorod geometries, the helical magnetic HOTIs generically exhibit even numbers of massive or massless twofold surface Dirac cones, and domain walls between surfaces with oppositely-signed masses bind mirror-protected helical hinge states. As we will show in SN 35, the helical magnetic HOTIs discovered in this work can be connected to nonmagnetic "rotation-anomaly" TCIs[25,36] without closing a bulk or surface gap or gapping the anomalous surface or hinge states. First, in SN 34, we will introduce the symmetry-enhanced fermion doubling theorems[36,39,74,111] for twofold Dirac fermions in the surface wallpaper groups of the helical magnetic HOTIs, which we will then use to diagnose the 2D surface states as anomalous. Unlike in SRef. 36, the twofold Dirac fermion doubling theorems introduced in SN 34 do not require $\{\mathcal{T}|\mathbf{0}\}$ to be enforced, and are instead only enforced by the spinful unitary magnetic symmetries of Type-I magnetic double wallpaper groups. Lastly, in SN 35, we will introduce tight-binding models for the helical magnetic HOTI phases, which we will use to explicitly demonstrate the presence of anomalous, mirror-protected 2D surface and 1D hinge states.

34. Symmetry-Enhanced Fermion Doubling Theorems for Non-Axionic Magnetic HOTIs

In this section, we will derive 2D symmetry-enhanced fermion doubling theorems[36,39,74,111] for the surface wallpaper groups[73,75] of spinful, helical magnetic HOTIs. Through the doubling theorems established in this section, we will demonstrate that the 2D, twofold Dirac surface states of helical magnetic HOTIs are anomalous (see SN 35 for tight-binding models and surface- and hinge-state calculations for helical magnetic HOTIs).

To begin, in each BZ of a 2D crystal, the parity anomaly excludes the presence of a single (*i.e.* unpaired) twofold-degenerate, linearly dispersing, ($\{\mathcal{T}|\mathbf{0}\}$- or magnetic-) symmetry-stabilized Dirac fermion[39,188,190–192]. However, on the 2D surfaces of 3D TIs[32,160,193,194] (and some AXIs, see SRefs. 28,38,40–60), unpaired twofold Dirac fermions are anomalously stabilized by the combination of surface wallpaper group symmetries and spectral (Wannier) flow. As shown in SRefs. 36,39, for 3D crystals whose surface wallpaper groups contain additional rotation and reflection symmetries, there also exist *symmetry-enhanced* fermion doubling theorems that may similarly be evaded through a combination of wallpaper group symmetry and spectral flow.

In SRef. 36, the authors specifically defined the *fermion multiplication theorem* for twofold Dirac fermions in nonmagnetic [Type-II, see SN 4] double wallpaper groups [which we will in this section take to be $\hat{\mathbf{z}}$-normal] that contain the symmetries $\{\mathcal{T}|\mathbf{0}\}$ and $\{C_{2z}|\mathbf{0}\}$, as well as, optionally, $\{C_{4z}|\mathbf{0}\}$ or $\{C_{6z}|\mathbf{0}\}$. To derive the fermion multiplication for nonmagnetic double wallpaper groups, we begin by exploiting the formulas derived in SRef. 164 for Berry phase in 2D crystals with rotation symmetries. Specifically, in SRef. 164, it was shown that the Berry phase Θ_2 in one half of the BZ of a 2D crystal with $\{C_{2z}|\mathbf{0}\}$ symmetry [SFig. 22(a)] is given by:

$$e^{i\Theta_2} = (-1)^{N_{occ}} \prod_{m\in occ} \zeta_m(\bar{\Gamma})\zeta_n(\bar{X})\zeta_m(\bar{Y})\zeta_m(\bar{M}), \tag{374}$$

that the Berry phase Θ_4 in one quarter of the BZ of a 2D crystal with $\{C_{4z}|\mathbf{0}\}$ symmetry [SFig. 22(b)] is given by:

$$e^{i\Theta_4} = (-1)^{N_{occ}} \prod_{m\in occ} \xi_m(\bar{\Gamma})\xi_m(\bar{M})\zeta_m(\bar{X}), \tag{375}$$

and that the Berry phase Θ_6 in one sixth of the BZ of a 2D crystal with $\{C_{6z}|\mathbf{0}\}$ symmetry [SFig. 22(c)] is given by:

$$e^{i\Theta_6} = (-1)^{N_{occ}} \prod_{m\in occ} \eta_m(\bar{\Gamma})\theta_m(\bar{K})\zeta_m(\bar{M}), \tag{376}$$

where $\zeta_m(K)$, $\xi_m(K)$, $\eta_m(K)$, $\theta_m(K)$ respectively refer to the C_{2z}, C_{4z}, C_{6z}, and C_{3z} eigenvalues of the mth Bloch state at K, and where N_{occ} is the number of Bloch states at each high-symmetry $\mathbf{k}$ point in a given energy range.

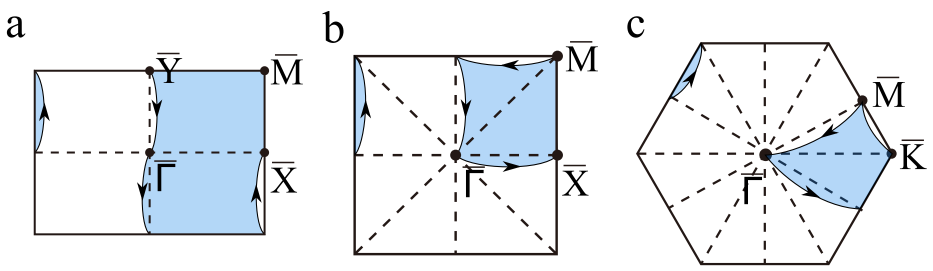

Supplementary Figure 22: The 2D BZs of wallpaper groups with even-fold rotation symmetries. (a) The 2D BZ of Type-I magnetic wallpaper group pmm [isomorphic to Type-I MSG 25.57 $Pmm2$ modulo out-of-plane lattice translations] or Type-II nonmagnetic wallpaper group $pmm1'$ [isomorphic to Type-II SG 25.58 $Pmm21'$ modulo out-of-plane lattice translations]. (b) The 2D BZ of Type-I magnetic wallpaper group $p4m$ [isomorphic to Type-I MSG 99.163 $P4mm$ modulo out-of-plane lattice translations] or Type-II nonmagnetic wallpaper group $p4m1'$ [isomorphic to Type-II SG 99.164 $P4mm1'$ modulo out-of-plane lattice translations]. (c) The 2D BZ of Type-I magnetic wallpaper group $p6m$ [isomorphic to Type-I MSG 183.185 $P6mm$ modulo out-of-plane lattice translations] or Type-II nonmagnetic wallpaper group $p6m1'$ [isomorphic to Type-II SG 183.186 $P6mm1'$ modulo out-of-plane lattice translations]. The dashed lines in (a-c) indicate mirror lines. The blue patches in (a-c) respectively indicate patches of the 2D BZ whose area is one half, one quarter, and one sixth of the first 2D BZ; the boundaries of the blue patches are explicitly chosen to avoid coinciding with the mirror lines. SEqs. (374), (375), and (376) respectively indicate the combinations of rotation symmetry eigenvalues that correspond to the quantized Berry phases $\Theta_{2,4,6} = 0, \pi$ in the blue patches in (a-c).

In SRef. 164, it was shown that $\Theta_{2,4,6} = \pi$ in SEqs. (374), (375), and (376) respectively indicates a nontrivial bulk Chern number. However, in the presence of $\{\mathcal{T}|\mathbf{0}\}$ or in-plane mirror symmetries, the Chern number is required to vanish[32,36,38,41,42,47,154,160,173]. In wallpaper groups with C_{2z}, C_{4z}, or C_{6z} rotation symmetry and either $\{\mathcal{T}|\mathbf{0}\}$ or in-plane mirror lines, the disagreement between $\Theta_{2,4,6} = \pi$ and the symmetry restriction that the Chern number vanish can be resolved by recognizing that a twofold Dirac fermion respectively placed in each half, quarter, and sixth of the 2D BZ also provides a source of π Berry phase indicated by $\Theta_{2,4,6} = \pi$[32,39,160,188,190–194]. In nonmagnetic (Type-II) wallpaper groups with C_{2z}, C_{4z}, or C_{6z} rotation symmetry, or in Type-I magnetic wallpaper groups with mirror and C_{2z}, C_{4z}, or C_{6z} rotation symmetry, $\Theta_{2,4,6}$ therefore respectively indicate the number of twofold Dirac cones in each BZ modulo 4, 8, and 12. Specifically, if $\Theta_{2,4,6} = \pi$ ($\Theta_{2,4,6} = 0$), there must be an odd (even) number of twofold Dirac cones in the blue BZ patches in SFig. 22(a-c), respectively implying the presence of $2 + 4a$ ($4a$), $4 + 8a$ ($8a$), or $6 + 12a$ ($12a$) twofold Dirac fermions in each BZ [where $a \in \{\mathbb{Z}^+, 0\}$]. In nonmagnetic wallpaper groups with C_{2z}, C_{4z}, or C_{6z} rotation symmetry, the Dirac fermions are stabilized by $\{C_{2z} \times \mathcal{T}|\mathbf{0}\}$ symmetry[36,38,154,173], and in the magnetic wallpaper groups in SFig. 22(a-c), the Dirac fermions are stabilized by mirror symmetry.

However, in SRef. 164, it was shown that $\Theta_{2,4,6} = 0$ for all 2D spinful lattice models with $\{\mathcal{T}|\mathbf{0}\}$ symmetry, due to the constraints imposed by $\mathcal{T}$ symmetry on the eigenvalues of spinful rotation symmetries. Below, we will show that $\Theta_{2,4,6} = 0$ is also required in all 2D spinful lattice models that respect the symmetries of Type-I magnetic double wallpaper groups containing $\{m_x|\mathbf{0}\}$ and $\{m_y|\mathbf{0}\}$ and C_{2z}, C_{4z}, or C_{6z} rotation symmetries (*i.e.* Type-I magnetic double wallpaper groups pmm, $p4m$, and $p6m$, respectively[36,39,73–75]). We note that throughout this work, the symbols of wallpaper groups – which are also sometimes termed *plane groups* – are given in the short notation previously employed in SRefs. 39,71,73; in the long notation of the Get Plane Gen tool on the BCS[13,14], magnetic wallpaper groups pmm, $p4m$, and $p6m$ are respectively labeled by the symbols $p2mm$, $p4mm$, and $p6mm$. For each C_{2z}-symmetric wallpaper group in SFig. 22(a-c), we will choose a patch of the 2D BZ whose boundary intersects the rotation-invariant $\mathbf{k}$ points in SEqs. (374), (375), and (376), respectively, while avoiding the mirror lines, which may host mirror-symmetry-stabilized Dirac fermions.

First, in double magnetic wallpaper group pmm [isomorphic to Type-I MSG 25.57 $Pmm2$ modulo out-of-plane lattice translations], the matrix representatives of $\{C_{2z}|\mathbf{0}\}$ and $\{m_x|\mathbf{0}\}$ anticommute at each of the four C_{2z}-invariant $\mathbf{k}$ points in SFig. 22(a) and SEq. (374) [this result can be obtained by applying the Corepresentations tool detailed in SN 13 to MSG 25.57 $Pmm2$]. Consequently, in 2D lattice models constructed from MEBRs, all of the small irreps σ at the four C_{2z}-invariant $\mathbf{k}$ points are two-dimensional, and exhibit net-zero C_{2z} eigenvalues: $\chi_\sigma(\{C_{2z}|\mathbf{0}\}) = 0$. For any set of energetically isolated multiplets of Bloch states at the four C_{2z}-invariant points in SFig. 22(a) and SEq. (374), this implies that $\Theta_2 \bmod 2\pi = 0$. Consequently, spinful lattice models in double magnetic wallpaper group pmm must exhibit even numbers of twofold Dirac fermions in each half of the 2D BZ in SFig. 22(a).

Similarly, for double magnetic wallpaper groups $p4m$ [isomorphic to Type-I MSG 99.163 $P4mm$ modulo out-of-plane lattice translations] and $p6m$ [isomorphic to Type-I MSG 183.185 $P6mm$ modulo out-of-plane lattice translations], it can be shown through the Corepresentations tool (see SN 13) that the spinful rotation eigenvalues of energetically

isolated multiplets of Bloch states must also appear in complex-conjugate pairs. This respectively implies that, for spinful lattice models in double magnetic wallpaper groups $p4m$ and $p6m$, $\Theta_{4,6} \bmod 2\pi = 0$. Consequently, spinful lattice models in double magnetic wallpaper group $p4m$ [$p6m$] must exhibit even numbers of twofold Dirac fermions in each quarter [sixth] of the 2D BZ in SFig. 22(b) [SFig. 22(c)].

As we will shortly see in SN 35, the surfaces of the helical magnetic HOTIs discovered in this work exhibit odd numbers of twofold Dirac cones in the blue regions of the 2D BZs shown in SFig. 22(a-c), representing anomalous exceptions to the magnetic fermion multiplication theorem derived in this section.

35. Tight-Binding Models and Boundary States for Non-Axionic Magnetic HOTIs

Through the double SIs computed in SN 31, we have discovered three novel variants of spinful, helical magnetic HOTIs with trivial axion angles $\theta \bmod 2\pi = 0$. In this section, we will provide tight-binding models and surface- and hinge-state calculations for the three non-axionic magnetic HOTI phases discovered in this work. For each phase, we will also demonstrate how the top ($\hat{\mathbf{z}}$-normal) surface states circumvent a magnetic fermion multiplication theorem (see SN 34). We will leave the development of bulk (nested) Wilson loop invariants[28,38,39,112,123] for the helical magnetic HOTI phases for future works. However, we note that, like the fourfold-rotation-anomaly HOTI phase in SnTe[25,34,161,162], in the helical magnetic HOTIs modeled in this section, the occupied bands in half of the bulk mirror planes that project to the $\hat{\mathbf{z}}$-normal surface (*e.g.* $\{m_{x\pm y}|\mathbf{0}\}$) exhibit mirror Chern numbers $C_m \bmod 4 = 2$, whereas the other half (*e.g.* $\{m_{x,y}|\mathbf{0}\}$) exhibit $C_m \bmod 4 = 0$ (see SFig. 21).

D_{2h} HOTI in double MSG 47.249 Pmmm – We will here analyze the helical magnetic TCI phase protected by the symmetries of double MPG mmm 8.1.24 [D_{2h}] (see SN 8 and 18 and SRefs. 7–18), which we term the *D_{2h} HOTI* (as previously in SN 8 and 18, we will continue to label MPGs in this section employing the notation of the MPOINT tool on the BCS[15–18] in which an MPG is labeled by its number, followed by its symbol). As discussed in SN 31 d, the double SIs $(z_4, z_{2w,1}, z_{2w,2}, z_{2w,3}) = (2000)$ in double MSG 47.249 $Pmmm$ indicate a mirror TCI for which the mirror Chern numbers $C_{m_x} \bmod 2 = C_{m_y} \bmod 2 = C_{m_z} \bmod 2 = 0$, $C_{m_x} + C_{m_y} + C_{m_z} \bmod 4 = 2$. Nevertheless, in this work, we refer to the (2000) phase in double MSG 47.249 $Pmmm$ as a helical HOTI for two reasons. First, as discussed in SN 31 d, the (2000) phase of double MSG 47.249 can be connected to a $(z_4, z_{2w,1}, z_{2w,2}, z_{2w,3})_{Pmmm1'} = (2000)_{Pmmm1'}$ mirror TCI phase in the $\mathcal{T}$-symmetric supergroup Type-II double SG 47.250 $Pmmm1'$ without closing a bulk or surface gap. In turn, the $(2000)_{Pmmm1'}$ TCI phase subduces to an $\mathcal{I}$- and $\mathcal{T}$-protected $(z_4, z_{2w,1}, z_{2w,2}, z_{2w,3})_{P\bar{1}1'} = (2000)_{P\bar{1}1'}$ helical HOTI in Type-II double SG 2.5 $P\bar{1}1'$ [see SN 31 s and SRefs. 24,26–28]. Second, in each of the momentum-space mirror planes in double MSG 47.249 $Pmmm$, a nontrivial mirror Chern number cannot be identified by the 2D symmetry-based mirror Chern indices implied by the Chern number SI formulas in SRef. 164. Specifically, each momentum-space mirror plane in double MSG 47.249 $Pmmm$ has only mirror, twofold rotation, and inversion symmetries, which can only indicate the mirror Chern number modulo 2. For example, in the $k_z = \pi$ plane of the bulk BZ in double MSG 47.249 $Pmmm$, the only SI of stable 2D topology is $z_{2w,3}$, which only indicates $C_{m_z}(k_z = \pi) \bmod 2$ (see SN 31 d). Hence, the nontrivial *even* mirror Chern numbers of the (2000) phase in double MSG 47.249 $Pmmm$ can only be inferred from the 3D double SIs $(z_4, z_{2w,1}, z_{2w,2}, z_{2w,3})$, and cannot be inferred from symmetry-indicated momentum-space mirror Chern numbers evaluated in BZ planes. As we will show in this section, and as discussed in previous works[26,27,34], TCI surface states may in general be interpreted as HOTI hinge modes if a finite sample is cut into a geometry in which the bulk mirror planes project to 1D hinges, as opposed to flat 2D surfaces.

To model the D_{2h} HOTI phase in double MSG 47.249 $Pmmm$, we begin by introducing the Bernevig-Hughes-Zhang Hamiltonian for a 3D TI[32,41,160,195]:

$$H_{\mathrm{TI}}(\mathbf{k}) = -\tau^z\left(2 - \sum_{i=x,y,z} \cos k_i\right) + \sum_{i=x,y,z} \tau^x \sigma^i \sin k_i, \tag{377}$$

where τ^i and σ^j are each 2×2 Pauli matrices, and where we have employed a notation in which $\tau^i\sigma^j = \tau^i \otimes \sigma^j$ and factors of the 2×2 identity matrices τ^0 and σ^0 are suppressed. SEq. (377) respects $\mathcal{I}$ and spinful $\mathcal{T}$ symmetries, which are represented through the symmetry action:

$$\mathcal{I}H_{\mathrm{TI}}(\mathbf{k})\mathcal{I}^{-1} = \tau^z H_{\mathrm{TI}}(-\mathbf{k})\tau^z,\ \mathcal{T}H_{\mathrm{TI}}(\mathbf{k})\mathcal{T}^{-1} = \sigma^y H^*_{\mathrm{TI}}(-\mathbf{k})\sigma^y. \tag{378}$$

We next construct the helical D_{2h} HOTI phase by first superposing two copies of the 3D TI phase of SEq. (377), and then introducing perturbative couplings to break $\mathcal{T}$ symmetry:

$$H^{Pmmm}_{\mathrm{HOTI}}(\mathbf{k}) = \mu^0 H_{\mathrm{TI}}(\mathbf{k}) + \Delta_0\mu^y\tau^y \sin k_x + \Delta_1[\mu^z(\tau^z + \tau^0) + (\tau^z + \tau^0)\sigma^z \sin k_x \sin k_y], \tag{379}$$

Bands		$\Gamma(000)$	$X(\pi 00)$	$Y(0\pi 0)$	$Z(00\pi)$	$S(\pi\pi 0)$	$T(0\pi\pi)$	$U(\pi 0\pi)$	$R(\pi\pi\pi)$
1-2	Energy	-1	-1.6	-1.6	-1.6	-3.6	-3.6	-3.6	-5.6
	σ	$\overline{\Gamma}_6$	$\overline{X}_5$	$\overline{Y}_5$	$\overline{Z}_5$	$\overline{S}_5$	$\overline{T}_5$	$\overline{U}_5$	$\overline{R}_5$
	$\Delta_\sigma(\mathcal{I})$	$-\xi^0$	ξ^0	ξ^0	ξ^0	ξ^0	ξ^0	ξ^0	ξ^0
3-4	Energy	-1	-0.4	-0.4	-0.4	-2.4	-2.4	-2.4	-4.4
	σ	$\overline{\Gamma}_6$	$\overline{X}_5$	$\overline{Y}_5$	$\overline{Z}_5$	$\overline{S}_5$	$\overline{T}_5$	$\overline{U}_5$	$\overline{R}_5$
	$\Delta_\sigma(\mathcal{I})$	$-\xi^0$	ξ^0	ξ^0	ξ^0	ξ^0	ξ^0	ξ^0	ξ^0

Supplementary Table 12: The double-valued small irreps corresponding to the four occupied bulk bands of the helical D_{2h} magnetic HOTI phase of SEq. (379) [SFig. 23(b)]. At each of the eight $\mathcal{I}$-invariant $\mathbf{k}$ points in MSG 47.249 $Pmmm$ [given in the notation $\mathbf{k}(k_x k_y k_z)$ and obtained through MKVEC, see SN 12 and SFig. 23(a)], we list the occupied band index and energy, the label of the double-valued small irrep σ that corresponds to each pair of occupied Bloch states at $\mathbf{k}$ in the notation of the Corepresentations tool [see SN 13], and the matrix representative $\Delta_\sigma(\mathcal{I})$ in the basis of the 2×2 Pauli matrices ξ^i.

in which μ^i is a 2×2 Pauli matrix that indexes the two coupled 3D TI models, and where we have employed a notation in which $\mu^i\tau^j\sigma^k = \mu^i\otimes\tau^j\otimes\sigma^k$ and factors of the 2×2 identity matrices μ^0, τ^0, σ^0 are suppressed in terms other than $\mu^0 H_{\mathrm{TI}}(\mathbf{k})$ when the identity matrices are not summed with other Pauli matrices. $H^{Pmmm}_{\mathrm{HOTI}}(\mathbf{k})$ in SEq. (379) respects the symmetries of double MPG mmm 8.1.24 $[D_{2h}]$, whose generating elements are represented through the action:

$$\begin{aligned}\mathcal{I}H^{Pmmm}_{\mathrm{HOTI}}(\mathbf{k})\mathcal{I}^{-1} &= \tau^z H^{Pmmm}_{\mathrm{HOTI}}(-\mathbf{k})\tau^z,\\ C_{2x}H^{Pmmm}_{\mathrm{HOTI}}(\mathbf{k})C_{2x}^{-1} &= \sigma^x H^{Pmmm}_{\mathrm{HOTI}}(C_{2x}\mathbf{k})\sigma^x,\\ C_{2y}H^{Pmmm}_{\mathrm{HOTI}}(\mathbf{k})C_{2y}^{-1} &= \mu^z\sigma^y H^{Pmmm}_{\mathrm{HOTI}}(C_{2y}\mathbf{k})\mu^z\sigma^y.\end{aligned} \tag{380}$$

Because $H^{Pmmm}_{\mathrm{HOTI}}(\mathbf{k})$ in SEq. (379) also respects the group of 3D orthogonal lattice translations, then SEq. (380) implies that $H^{Pmmm}_{\mathrm{HOTI}}(\mathbf{k})$ respects the symmetries of double MSG 47.249 $Pmmm$. In SEq. (379), the Δ_0 and Δ_1 terms break $\mathcal{T}$ symmetry. The Δ_0 term vanishes at the eight $\mathcal{I}$-invariant $\mathbf{k}$ points $k_{x,y,z} = 0,\pi$ [SFig. 23(a)], whereas the Δ_1 term is generically nonzero at all values of $\mathbf{k}$.

To realize the helical D_{2h} HOTI phase of $H^{Pmmm}_{\mathrm{HOTI}}(\mathbf{k})$, we choose $\Delta_0 = 1$, $\Delta_1 = 0.3$ in SEq. (379). We have chosen a relatively small value of Δ_1 to ensure that the band ordering remains the same as in the $\mathcal{T}$-symmetric limit in which $\Delta_{0,1}$ vanish. Specifically, as discussed in SN 31 d and earlier in this section, in the $\mathcal{T}$-symmetric limit, $H^{Pmmm}_{\mathrm{HOTI}}(\mathbf{k})$ realizes a twofold-rotation-anomaly, helical, nonmagnetic HOTI phase with a nontrivial bulk mirror Chern number[24,26–28,35,36] indicated by the double SIs $(z_4, z_{2w,1}, z_{2w,2}, z_{2w,3}) = (2000)$ in the Type-II double SG 47.250 $Pmmm1'$. In SFig. 23(b), we plot the bulk band structure of SEq. (379); we emphasize that SEq. (379) contains additional, extraneous (artificial) symmetries beyond those of double MSG 47.249 $Pmmm$. Hence, the band structure in SFig. 23(b) exhibits additional degeneracies away from the Fermi level – such as the occupied fourfold degeneracy at Γ – that are not robust to symmetry-preserving perturbations.

To diagnose the topology of SEq. (379), we will perform two sets of calculations. First, we will calculate the double SIs of the four occupied bands. Then, we will demonstrate the presence of anomalous surface and hinge states when SEq. (379) is terminated in a finite, D_{2h}-symmetric nanorod geometry [SFig. 23(c)]. To begin, in Supplementary Table 12, we list the double-valued small irreps that correspond to the four occupied spinful Bloch eigenstates at each of the eight $\mathcal{I}$-invariant $\mathbf{k}$ points in MSG 47.249 $Pmmm$ [SFig. 23(a)]. The matrix representative $\Delta_\sigma(\mathcal{I})$ of each two-dimensional small irrep σ in Supplementary Table 12 is diagonal, indicating that each pair of Bloch states at each $\mathcal{I}$-invariant $\mathbf{k}$ point has two parity eigenvalues with the same sign. From Supplementary Table 12, we obtain the occupied parity eigenvalue multiplicities:

$$n^-_\Gamma = 4,\ n^+_\Gamma = 0,\ n^-_K = 0, n^+_K = 4 \text{ for } K = X,Y,Z,S,T,U,R. \tag{381}$$

Substituting SEq. (381) into the double SI formulas in Type-I double MSG 47.249 $Pmmm$ [SEqs. (259) and (260)], we find that:

$$z_4 = \sum_K \frac{1}{2} n^-_K \bmod 2 = \sum_K \frac{n^-_K - n^+_K}{4} \bmod 4 = \frac{4-28}{4} \bmod 4 = 2, \tag{382}$$

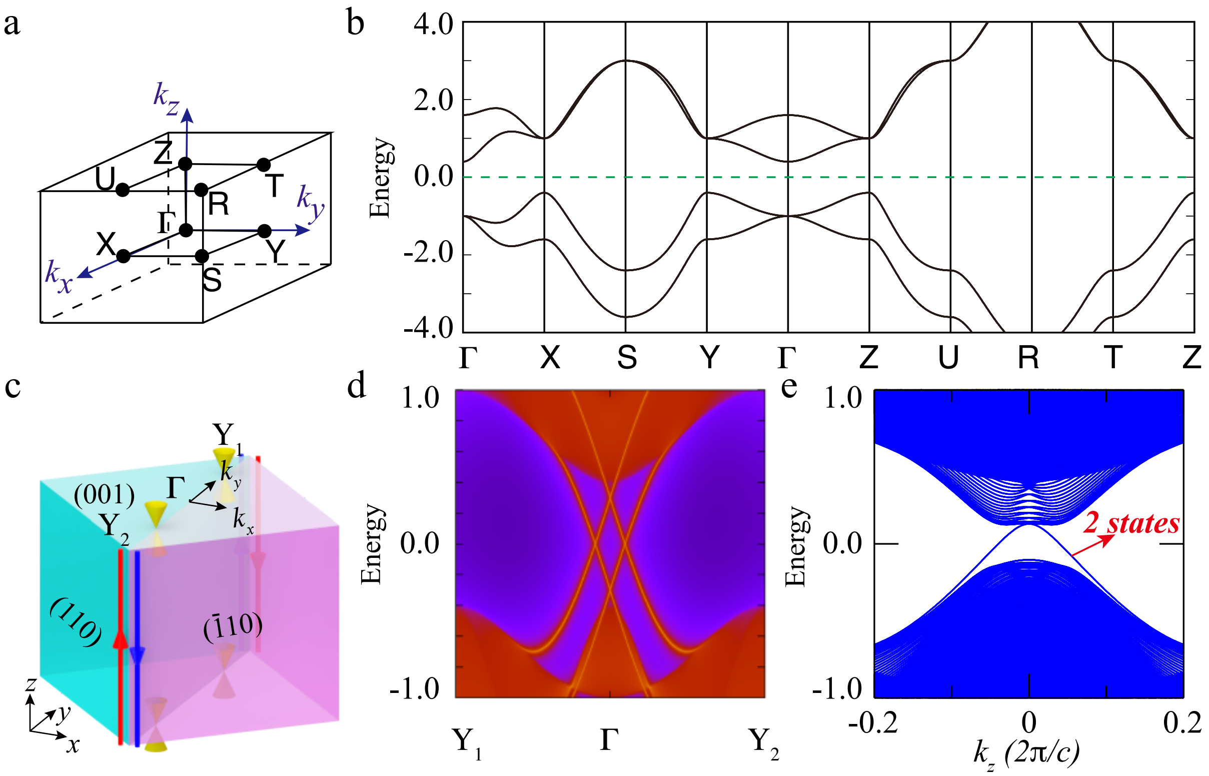

Supplementary Figure 23: Surface and hinge states of the helical magnetic D_{2h} HOTI phase in double MSG 47.249 $Pmmm$. (a) The bulk BZ. (b) The bulk band structure obtained from SEq. (379) with $\Delta_0 = 1$ and $\Delta_1 = 0.3$. We note that SEq. (379) contains additional, extraneous symmetries beyond those of double MSG 47.249 $Pmmm$, such that the band structure in (b) exhibits additional degeneracies away from the Fermi level – such as the occupied fourfold degeneracy at Γ – that are not robust to symmetry-preserving perturbations. (c) Schematic of the top ($\hat{\mathbf{z}}$-normal) surface states and nanorod hinge states. The top surface of the rectangular nanorod in (c) respects the symmetries of Type-I double magnetic wallpaper group pmm, and the hinges respect the symmetries of frieze groups that contain either $\{m_x|0\}$ or $\{m_y|0\}$ (see SN 31 and 34 and SRefs. 34,39,73–75). (d) The top surface spectrum plotted along k_y, obtained from surface Green's functions calculated for the model in (b) terminated in a z-directed slab geometry. In (d), the surface bands exhibit mirror Chern $C_{m_x} = 2$ spectral flow. We have verified through surface-state calculations that the slab surface spectrum along k_x does not exhibit spectral flow, and that $C_{m_z} = 0$. Together, this implies that the top surface exhibits two twofold Dirac cones, circumventing the fermion multiplication theorem for double magnetic wallpaper group pmm derived in SN 34, and implies that the bulk is a D_{2h} HOTI. (e) The spectrum of an infinite, z-directed, $m_{x,y}$-symmetric nanorod of the model in (b) features two pairs of hinge-localized helical modes (four total hinge states), demonstrating that the model in (b) exhibits higher-order spectral flow.

and:

$$\begin{aligned}
z_{2w,1} &= \sum_{K=X,S,U,R} \frac{1}{2} n_K^- \bmod 2 = 0, \\
z_{2w,2} &= \sum_{K=Y,S,T,R} \frac{1}{2} n_K^- \bmod 2 = 0, \\
z_{2w,3} &= \sum_{K=Z,T,U,R} \frac{1}{2} n_K^- \bmod 2 = 0, \qquad (383)
\end{aligned}$$

such that the occupied bands of SEq. (379) shown in SFig. 23(b) exhibit the double SIs $(z_4, z_{2w,1}, z_{2w,2}, z_{2w,3}) = (2000)$.

Previously, in SN 31 d, we showed that the double SIs $(z_4, z_{2w,1}, z_{2w,2}, z_{2w,3}) = (2000)$ in double MSG 47.249 $Pmmm$ indicate a mirror TCI phase that we designate in this work to be a helical D_{2h} HOTI. To demonstrate that SEq. (379), with the parameters used to obtain SFig. 23(b), exhibits the anomalous surface and hinge states of a D_{2h} HOTI, we have performed two boundary state calculations. First, as shown in SFig. 23(d), we have calculated the top ($\hat{\mathbf{z}}$-normal) surface spectrum of $H_{\text{HOTI}}^{Pmmm}(\mathbf{k})$ terminated in a z-directed slab geometry. The top surface of a crystal in MSG 47.249 $Pmmm$ respects the symmetries of Type-I magnetic wallpaper group pmm (see SN 31 and 34

and SRefs. 34,39,73–75). The slab surface spectrum in SFig. 23(d) exhibits mirror Chern $C_{m_x} = 2$ spectral flow, and we have additionally verified through surface-state calculations that $C_{m_y} = 0$. Together, this implies that the top surface exhibits two twofold Dirac cones, circumventing the fermion multiplication theorem for double magnetic wallpaper group pmm derived in SN 34. We next calculate the spectrum of an infinite, z-directed, $m_{x,y}$-symmetric nanorod of $H_{\text{HOTI}}^{Pmmm}(\mathbf{k})$ [SFig. 23(e)]. We observe two pairs of hinge-localized helical modes in the nanorod spectrum in SFig. 23(e), confirming that $H_{\text{HOTI}}^{Pmmm}(\mathbf{k})$ exhibits the higher-order spectral flow of a D_{2h} HOTI.

D_{4h} HOTI in double MSG 123.339 $P4/mmm$ – We will next analyze the helical magnetic HOTI phase protected by the symmetries of double MPG 15.1.53 $4/mmm$ [D_{4h}] (see SN 8 and 18 and SRefs. 7–18), which we term the *D_{4h} HOTI.* As discussed in SN 31 k, the double SIs $(z_8, z_{4m,\pi}^{-}, z_{2w,1}) = (400)$ in double MSG 123.339 $P4/mmm$ either indicate a mirror TCI with mirror Chern number C_{m_z} mod $8 = 4$, or indicate a helical D_{4h} HOTI phase in which half of the z-projecting mirror planes (*e.g.* the $\{m_{x\pm y}|\mathbf{0}\}$-invariant planes) exhibit C_m mod $4 = 2$, the other half (*e.g.* the $\{m_{x,y}|\mathbf{0}\}$-invariant planes) exhibit C_m mod $4 = 0$, and $C_{m_z} = 0$ [see SFig. 21(b)]. To construct the helical D_{4h} HOTI phase, we first superpose two copies of the 3D TI phase of SEq. (377), but crucially, in a manner in which the two 3D TIs are formed from different orbital hybridization [*e.g.* $s - p_z$ and $s - f_{xyz}$]. As we will see, this implies that the two superposed 3D TIs exhibit different valence C_{4z} eigenvalues (see SRef. 71 for closely related discussions of orbital hybridization and anomalous corner modes in 2D TIs and 3D Dirac semimetals). We next add perturbative couplings to break $\mathcal{T}$ symmetry, resulting in the 3D Hamiltonian:

$$H_{\text{HOTI}}^{P4/mmm}(\mathbf{k}) = \mu^0 H_{\text{TI}}(\mathbf{k}) + \Delta_0(\mu^x + \mu^y)(2\tau^y - \sigma^z \sin k_z)(\cos k_x - \cos k_y) + \Delta_1\mu^z, \tag{384}$$

employing the notation detailed in the text following SEq. (379). $H_{\text{HOTI}}^{P4/mmm}(\mathbf{k})$ respects the symmetries of double MPG 15.1.53 $4/mmm$ [D_{4h}], whose generating elements are represented through the action:

$$\begin{aligned} \mathcal{I}H_{\text{HOTI}}^{P4/mmm}(\mathbf{k})\mathcal{I}^{-1} &= \mu^z\tau^z H_{\text{HOTI}}^{P4/mmm}(-\mathbf{k})\mu^z\tau^z, \\ C_{4z}H_{\text{HOTI}}^{P4/mmm}(\mathbf{k})C_{4z}^{-1} &= \mu^z e^{-i\frac{\pi}{4}\sigma^z} H_{\text{HOTI}}^{P4/mmm}(C_{4z}\mathbf{k})\mu^z e^{i\frac{\pi}{4}\sigma^z}, \\ C_{2x}H_{\text{HOTI}}^{P4/mmm}(\mathbf{k})C_{2x}^{-1} &= \sigma^x H_{\text{HOTI}}^{P4/mmm}(C_{2x}\mathbf{k})\sigma^x. \end{aligned} \tag{385}$$

Because $H_{\text{HOTI}}^{P4/mmm}(\mathbf{k})$ in SEq. (384) also respects the group of 3D tetragonal lattice translations, then SEq. (385) implies that $H_{\text{HOTI}}^{P4/mmm}(\mathbf{k})$ respects the symmetries of double MSG 123.339 $P4/mmm$. In SEq. (384), the Δ_0 term breaks $\mathcal{T}$ symmetry, and the Δ_1 term breaks the extraneous exchange symmetry represented by $\mu^x + \mu^y$ between the two superposed 3D TIs at all $\mathbf{k}$ points.

To realize the helical D_{4h} HOTI phase of $H_{\text{HOTI}}^{P4/mmm}(\mathbf{k})$, we choose $\Delta_0 = 0.5$ and $\Delta_1 = 0.2$ in SEq. (384). We have chosen a relatively small value of Δ_1 to ensure that the band ordering remains the same as in the $\mathcal{T}$-symmetric limit in which Δ_0 vanishes. Specifically, as discussed in SN 31 k, in the $\mathcal{T}$-symmetric limit, $H_{\text{HOTI}}^{P4/mmm}(\mathbf{k})$ realizes the same fourfold-rotation-anomaly, helical, nonmagnetic HOTI phase indicated by the double SIs in $(z_8, z_{4m,\pi}^{-}, z_{2w,1}) = (400)$ in the Type-II double SG 123.340 $P4/mmm1'$ as a tetragonal supercell of the well-studied TCI SnTe[25–27,34,36,161,162]. In SFig. 24(b), we plot the bulk band structure of SEq. (384).

To diagnose the topology of SEq. (384), we will perform two sets of calculations. First, we will calculate the double SIs of the four occupied bands. Then, we will demonstrate the presence of anomalous surface and hinge states when SEq. (384) is terminated in a finite, D_{4h}-symmetric nanorod geometry [SFig. 24(c)]. To begin, in Supplementary Table 13, we list the double-valued small irreps that correspond to the four occupied spinful Bloch eigenstates at the six high-symmetry $\mathbf{k}$ points shown in SFig. 24(a). From the matrix representative $\Delta_\sigma(h)$ of each two-dimensional small irrep for each of the representative unitary symmetries h of the little group $G_{\mathbf{k}}$ [*e.g.* C_{4z} and $\mathcal{I}$, see SEq. (71) and the surrounding text], we may infer the symmetry eigenvalues of the four occupied bands. Using the matrix representatives in Supplementary Table 13, we then compute the auxiliary variables [see SEq. (306) and the surrounding text]:

$$\begin{aligned} n^{\frac{3}{2},+} &= \sum_{K=\Gamma,M,Z,A} n_K^{\frac{3}{2},+} + \sum_{K=X,R} n^{\frac{1}{2},+} = 1 + 4 = 5, \\ n^{\frac{3}{2},-} &= \sum_{K=\Gamma,M,Z,A} n_K^{\frac{3}{2},-} + \sum_{K=X,R} n^{\frac{1}{2},-} = 3 + 4 = 7, \\ n^{\frac{1}{2},+} &= \sum_{K=\Gamma,M,Z,A} n_K^{\frac{1}{2},+} + \sum_{K=X,R} n^{\frac{1}{2},+} = 1 + 4 = 5, \\ n^{\frac{1}{2},-} &= \sum_{K=\Gamma,M,Z,A} n_K^{\frac{1}{2},-} + \sum_{K=X,R} n^{\frac{1}{2},-} = 3 + 4 = 7, \end{aligned} \tag{386}$$

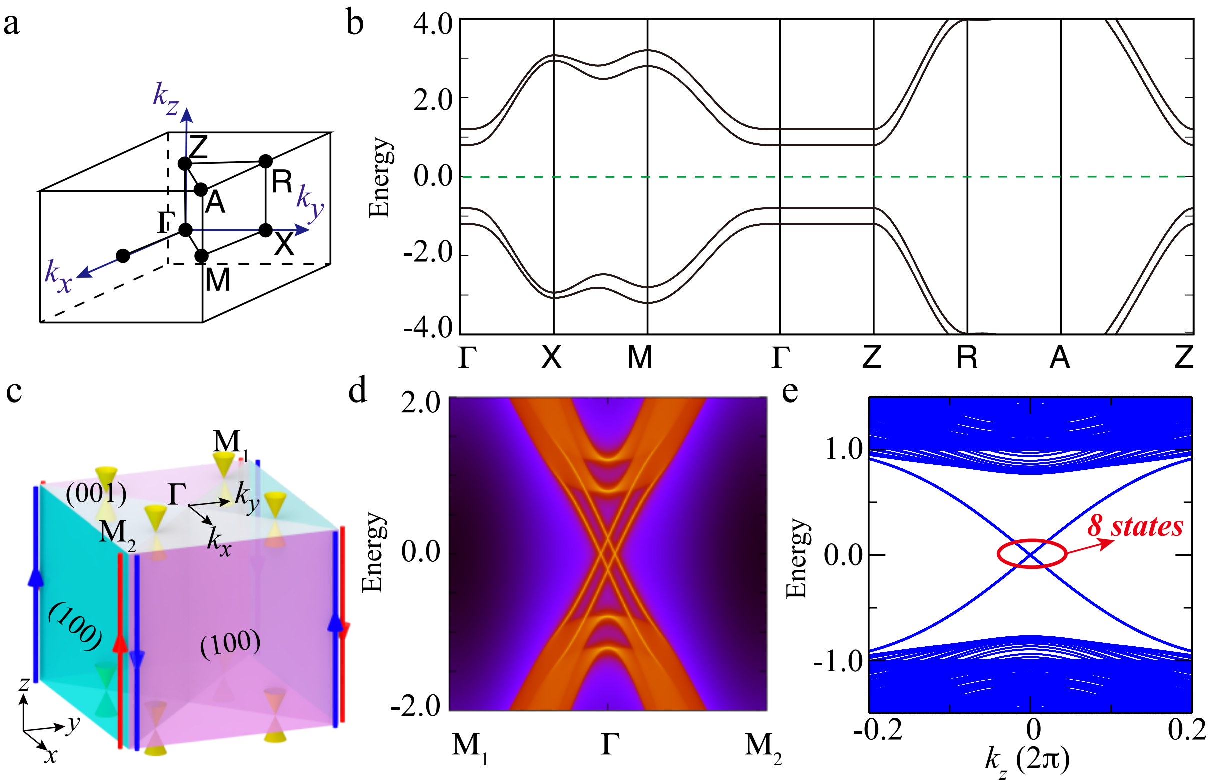

Supplementary Figure 24: Surface and hinge states of the helical magnetic D_{4h} HOTI phase in double MSG 123.339 $P4/mmm$. (a) The bulk BZ. (b) The bulk band structure obtained from SEq. (384) with $\Delta_0 = 0.5$ and $\Delta_1 = 0.2$. (c) Schematic of the top ($\hat{\mathbf{z}}$-normal) surface states and nanorod hinge states. The top surface of the square nanorod in (c) respects the symmetries of Type-I double magnetic wallpaper group $p4m$, and the hinges respect the symmetries of frieze groups that contain either $\{m_{x\pm y}|0\}$ (see SN 31 and 34 and SRefs. 34,39,73–75). (d) The top surface spectrum plotted along k_{x-y}, obtained from surface Green's functions calculated for the model in (b) terminated in a z-directed slab geometry. In (d), the surface bands exhibit mirror Chern $C_{m_{x+y}} = 2$ spectral flow. We have verified through surface-state calculations that the C_{4z}-related slab surface spectrum along k_{x+y} also exhibits $C_{m_{x-y}} = 2$ spectral flow, that the surface spectrum along $k_{x,y}$ exhibits trivial $C_{m_{y,x}} = 0$ spectral flow, and that $C_{m_z} = 0$. Together, this implies that the top surface exhibits four twofold Dirac cones, circumventing the fermion multiplication theorem for double magnetic wallpaper group $p4m$ derived in SN 34, and implies that the bulk is a D_{4h} HOTI. (e) The spectrum of an infinite, C_{4z}- and $m_{x\pm y}$-symmetric nanorod of the model in (b) features four pairs of hinge-localized helical modes (eight total hinge states), demonstrating that the model in (b) exhibits higher-order spectral flow.

where $n_K^{j,\pm}$ is the number of occupied states with the C_{4z} eigenvalues $e^{-i\frac{\pi}{2}j}$ and the parity ($\mathcal{I}$) eigenvalues ± 1 at K [which is only well-defined at the four C_{4z}-invariant momenta $K = \Gamma, M, Z, A$, see SFig. 24(a)]. Additionally, in SEq. (386), $n_K^{\frac{1}{2},\pm}$ is the number of occupied states with the C_{2z} eigenvalues $-i$ and the parity eigenvalues ± 1 at the points $K = X, R$. Substituting SEq. (386) into the double SI formula for z_8 in Type-I double MSG 123.339 $P4/mmm$ [SEq. (305)], we obtain:

$$z_8 = \frac{3(n^{\frac{3}{2},+} - n^{\frac{3}{2},-}) - (n^{\frac{1}{2},+} - n^{\frac{1}{2},-})}{2} \bmod 8 = \frac{3 \times (5-7) - (5-7)}{2} \bmod 8 = 4. \tag{387}$$

To complete the double SI calculation, we must also determine the values of $z_{4m,\pi}^{-}$ and $z_{2w,1}$ (see SN 31 k). To compute $z_{4m,\pi}^{-}$ and $z_{2w,1}$, we first use Supplementary Table 13 to calculate the number of C_{4z} eigenvalues at Z and A in each mirror sector:

$$\begin{aligned} n_Z^{\frac{1}{2},-i} &= 1,\ n_Z^{-\frac{1}{2},-i} = 0,\ n_Z^{\frac{3}{2},-i} = 1,\ n_Z^{-\frac{3}{2},-i} = 0, \\ n_A^{\frac{1}{2},-i} &= 1,\ n_A^{-\frac{1}{2},-i} = 0,\ n_A^{\frac{3}{2},-i} = 1,\ n_A^{-\frac{3}{2},-i} = 0, \end{aligned} \tag{388}$$

Bands		$\Gamma(000)$	$Z(00\pi)$	$M(\pi\pi 0)$	$A(\pi\pi\pi)$
1-2	Energy	-1.2	-1.2	-3.2	-5.2
	σ	$\overline{\Gamma}_6$	$\overline{Z}_8$	$\overline{M}_8$	$\overline{A}_8$
	$\Delta_\sigma(\mathcal{I})$	ξ^0	$-\xi^0$	$-\xi^0$	$-\xi^0$
	$\Delta_\sigma(C_{4z})$	$e^{-i\frac{3\pi}{4}\xi^z}$	$e^{-i\frac{3\pi}{4}\xi^z}$	$e^{-i\frac{3\pi}{4}\xi^z}$	$e^{-i\frac{3\pi}{4}\xi^z}$
	$\Delta_\sigma(m_z)$	$i\xi^z$	$-i\xi^z$	$-i\xi^z$	$-i\xi^z$
3-4	Energy	-0.8	-0.8	-2.8	-4.8
	σ	$\overline{\Gamma}_9$	$\overline{Z}_7$	$\overline{M}_7$	$\overline{A}_7$
	$\Delta_\sigma(\mathcal{I})$	$-\xi^0$	ξ^0	ξ^0	ξ^0
	$\Delta_\sigma(C_{4z})$	$e^{-i\frac{\pi}{4}\xi^z}$	$e^{-i\frac{\pi}{4}\xi^z}$	$e^{-i\frac{\pi}{4}\xi^z}$	$e^{-i\frac{\pi}{4}\xi^z}$
	$\Delta_\sigma(m_z)$	$i\xi^z$	$-i\xi^z$	$-i\xi^z$	$-i\xi^z$

Bands		$X(0\pi 0)$	$R(0\pi\pi)$
1-2	Energy	-3.07	-4.27
	σ	$\overline{X}_6$	$\overline{R}_6$
	$\Delta_\sigma(\mathcal{I})$	$-\xi^0$	$-\xi^0$
	$\Delta_\sigma(C_{2z})$	$-i\xi^z$	$-i\xi^z$
	$\Delta_\sigma(m_z)$	$i\xi^z$	$i\xi^z$
3-4	Energy	-2.94	-3.98
	σ	$\overline{X}_5$	$\overline{R}_5$
	$\Delta_\sigma(\mathcal{I})$	ξ^0	ξ^0
	$\Delta_\sigma(C_{2z})$	$-i\xi^z$	$-i\xi^z$
	$\Delta_\sigma(m_z)$	$-i\xi^z$	$-i\xi^z$

Supplementary Table 13: The double-valued small irreps corresponding to the four occupied bulk bands of the helical D_{4h} magnetic HOTI phase of SEq. (384) [SFig. 24(b)]. At one $\mathbf{k}$ point in each of the six maximal momentum stars in MSG 123.339 $P4/mmm$ [given in the notation $\mathbf{k}(k_xk_yk_z)$ and obtained through MKVEC, see SN 12 and SFig. 24(a)], we list the occupied band index and energy, the label of the double-valued small irrep σ that corresponds to each pair of occupied Bloch states at $\mathbf{k}$ in the notation of the Corepresentations tool [see SN 13], and the matrix representatives $\Delta_\sigma(h)$ of the representative unitary symmetries h of the little group $G_{\mathbf{k}}$ [see SEq. (71) and the surrounding text] in the basis of the 2×2 Pauli matrices ξ^i.

as well as the number of C_{2z} eigenvalues at R in each mirror sector:

$$n_R^{\frac{1}{2},-i} = n_R^{-\frac{1}{2},-i} = 1. \tag{389}$$

From SEqs. (388) and (389), we then compute $z^-_{4m,\pi}$ [SEq. (274)]:

$$\begin{aligned} z^-_{4m,\pi} &= \sum_{K=Z,A}\left(-\frac{1}{2}n_K^{\frac{1}{2},-i} + \frac{1}{2}n_K^{-\frac{1}{2},-i} - \frac{3}{2}n_K^{\frac{3}{2},-i} + \frac{3}{2}n_K^{-\frac{3}{2},-i}\right) + n_R^{\frac{1}{2},-i} - n_R^{-\frac{1}{2},-i} \bmod 4 \\ &= -\frac{1}{2}(1+1) + \frac{1}{2}(0+0) - \frac{3}{2}(1+1) + \frac{3}{2}(0+0) + 1 - 1 \bmod 4 = 0. \end{aligned} \tag{390}$$

Lastly, using SEqs. (386), (388), and (389), we compute $z_{2w,1}$ [SEq. (270)]:

$$z_{2w,1} = \sum_{K=X',R',M,A}\frac{1}{2}n_K^- \bmod 2 = \frac{1}{2}(2+2+2+2) \bmod 2 = 0, \tag{391}$$

where $X' = C_{4z}^{-1}X$ and $R' = C_{4z}^{-1}R$. SEq. (391) implies that the occupied bands of SEq. (384) shown in SFig. 24(b) exhibit the double SIs $(z_8, z^-_{4m,\pi}, z_{2w,1}) = (400)$.

Previously, in SN 31 k, we showed that the double SIs $(z_8, z^-_{4m,\pi}, z_{2w,1}) = (400)$ in double MSG 123.339 $P4/mmm$ either indicate a mirror TCI with mirror Chern number $C_{m_z} \bmod 8 = 4$, or indicate a helical D_{4h} HOTI phase in which half of the z-projecting mirror planes (*e.g.* the $\{m_{x\pm y}|\mathbf{0}\}$-invariant planes) exhibit $C_m \bmod 4 = 2$, the other half (*e.g.* the $\{m_{x,y}|\mathbf{0}\}$-invariant planes) exhibit $C_m \bmod 4 = 0$, and $C_{m_z} = 0$ [see SFig. 21(b)]. To demonstrate that SEq. (384), with the parameters used to obtain SFig. 24(b), is a D_{4h} HOTI, we have performed two boundary state calculations. First, as shown in SFig. 24(d), we have calculated the top ($\hat{\mathbf{z}}$-normal) surface spectrum of $H^{P4/mmm}_{\text{HOTI}}(\mathbf{k})$ terminated in a z-directed slab geometry. The top surface of a crystal in double MSG 123.339 $P4/mmm$ respects the symmetries of Type-I magnetic wallpaper group $p4m$ (see SN 31 and 34 and SRefs. 34,39,73–75). The slab surface spectrum in SFig. 24(d) exhibits four twofold Dirac cones, circumventing the fermion multiplication theorem for double magnetic wallpaper group $p4m$ derived in SN 34. We next calculate the spectrum of an infinite, z-directed, C_{4z}- and $m_{x\pm y}$-symmetric nanorod of $H^{P4/mmm}_{\text{HOTI}}(\mathbf{k})$ [SFig. 24(e)]. We observe four pairs of hinge-localized helical modes in the nanorod spectrum in SFig. 24(e), confirming that $H^{P4/mmm}_{\text{HOTI}}(\mathbf{k})$ exhibits the higher-order spectral flow of a D_{4h} HOTI.

D_{6h} HOTI in double MSG 191.233 $P6/mmm$ – Finally, we will now analyze the helical magnetic HOTI phase protected by the symmetries of double MPG 27.100 $6/mmm$ [D_{6h}] (see SN 8 and 18 and SRefs. 7–18), which we term the *D_{6h} HOTI*. As discussed in SN 31 r, the double SIs $(z_{12}, z^+_{6m,\pi}) = (60)$ in double MSG 191.233 $P6/mmm$ either indicate a mirror TCI with mirror Chern number $C_{m_z} \bmod 12 = 6$, or indicate a helical D_{6h} HOTI in which half

of the z-projecting mirror planes (*e.g.* the $\{m_x|\mathbf{0}\}$-, $\{C_{6z}m_xC_{6z}^{-1}|\mathbf{0}\}$-, and $\{C_{6z}^{-1}m_xC_{6z}|\mathbf{0}\}$-invariant planes) exhibit C_m mod $4 = 2$, the other half (*e.g.* the $\{m_y|\mathbf{0}\}$-, $\{C_{6z}m_yC_{6z}^{-1}|\mathbf{0}\}$-, and $\{C_{6z}^{-1}m_yC_{6z}|\mathbf{0}\}$-invariant planes) exhibit C_m mod $4 = 0$, and $C_{m_z} = 0$ [see SFig. 21(c)].

To construct the helical D_{6h} HOTI phase, we begin by introducing the hexagonal lattice vectors:

$$\mathbf{t}_1 = (0,-1,0),\ \mathbf{t}_2 = (\sqrt{3}/2,1/2,0),\ \mathbf{t}_3 = (0,0,1), \tag{392}$$

and reciprocal lattice vectors:

$$\mathbf{b}_1 = (\sqrt{3}/3,-1,0),\ \mathbf{b}_2 = (2\sqrt{3}/3,0,0),\ \mathbf{b}_3 = (0,0,1). \tag{393}$$

We define the first BZ to consist of the points $\mathbf{k} = \sum_{i=1,2,3} k_i\mathbf{b}_i,\ k_i \in [-\pi,\pi)$ [see SFig. 25(a)].

Next, we introduce a model for a 3D TI with hexagonal lattice vectors:

$$H_{\text{TI}}^{P6/mmm1'}(\mathbf{k}) = \tau^z M(\mathbf{k}) + \tau^x\bar{\sigma}^1\sin(2k_1+k_2) + \tau^x\bar{\sigma}^2\sin(k_2-k_1) + \tau^x\bar{\sigma}^3\sin(k_1+2k_2) + \tau^x\sigma^z\sin(k_3), \tag{394}$$

where we have employed the notation detailed in the text following SEq. (377), and where:

$$M(\mathbf{k}) = 3 - \sum_{i=1,2,3}\cos(k_i) - \cos(k_1+k_2). \tag{395}$$

In SEq. (394), we have employed a canonical Pauli matrix transformation given by:

$$\bar{\sigma}^1 = \frac{\sqrt{3}}{2}\sigma^x - \frac{1}{2}\sigma^y,\ \bar{\sigma}^2 = \sigma^y,\ \bar{\sigma}^3 = \frac{\sqrt{3}}{2}\sigma^x + \frac{1}{2}\sigma^y. \tag{396}$$

SEq. (394) respects $\mathcal{I}$ and spinful $\mathcal{T}$ symmetries, which are represented through the symmetry action:

$$\mathcal{I}H_{\text{TI}}^{P6/mmm1'}(\mathbf{k})\mathcal{I}^{-1} = \tau^z H_{\text{TI}}^{P6/mmm1'}(-\mathbf{k})\tau^z,\ \mathcal{T}H_{\text{TI}}^{P6/mmm1'}(\mathbf{k})\mathcal{T}^{-1} = \sigma^y[H_{\text{TI}}^{P6/mmm1'}(-\mathbf{k})]^*\sigma^y. \tag{397}$$

As was done for the D_{4h} HOTI earlier in this section, we next superpose two copies of the 3D TI phase of SEq. (394), but again in a manner in which the two 3D TIs are formed from different orbital hybridization, such that the occupied bands of the two 3D TIs exhibit different C_{6z} and C_{3z} eigenvalues [see SRef. 71 and the text preceding SEq. (384)]. We then add perturbative couplings to break $\mathcal{T}$ symmetry, resulting in the 3D Hamiltonian:

$$H_{\text{HOTI}}^{P6/mmm}(\mathbf{k}) = \mu^0 H_{\text{TI}}^{P6/mmm1'}(\mathbf{k}) + (\mu^x+\mu^y)[(\tau^x+\tau^y) + \frac{1}{2}\sigma^z\sin(k_3)]f(\mathbf{k}) + \Delta_1\mu^z(\tau^z+\tau^0), \tag{398}$$

where we have employed the notation detailed in the text following SEq. (379), and where:

$$f(\mathbf{k}) = \Delta_0[\sin(k_2-k_1) + \sin(2k_1+k_2) - \sin(k_1+2k_2)]. \tag{399}$$

$H_{\text{HOTI}}^{P6/mmm}(\mathbf{k})$ respects the symmetries of double MPG 27.1.100 $6/mmm$ $[D_{6h}]$, whose generating elements are represented through the action:

$$\begin{aligned}
\mathcal{I}H_{\text{HOTI}}^{P6/mmm}(\mathbf{k})\mathcal{I}^{-1} &= \tau^z H_{\text{HOTI}}^{P6/mmm}(-\mathbf{k})\tau^z,\\
C_{6z}H_{\text{HOTI}}^{P6/mmm}(\mathbf{k})C_{6z}^{-1} &= \mu^z e^{-i\frac{\pi}{6}\sigma^z}H_{\text{HOTI}}^{P6/mmm}(C_{6z}\mathbf{k})\mu^z e^{i\frac{\pi}{6}\sigma^z},\\
C_{2y}H_{\text{HOTI}}^{P6/mmm}(\mathbf{k})C_{2y}^{-1} &= \mu^z\sigma^x H_{\text{HOTI}}^{P6/mmm}(C_{2y}\mathbf{k})\mu^z\sigma^x.
\end{aligned} \tag{400}$$

Because $H_{\text{HOTI}}^{P6/mmm}(\mathbf{k})$ in SEq. (399) also respects the group of 3D hexagonal lattice translations, then SEq. (400) implies that $H_{\text{HOTI}}^{P6/mmm}(\mathbf{k})$ respects the symmetries of double MSG 191.233 $P6/mmm$. In SEqs. (398) and (399), the Δ_0 term breaks $\mathcal{T}$ symmetry, and the Δ_1 term breaks the extraneous exchange symmetry represented by $\mu^x+\mu^y$ between the two superposed hexagonal 3D TIs in the $\tau^+ = \frac{1}{2}[\tau^z+\tau^0]$ subspace at all $\mathbf{k}$ points.

To realize the helical D_{6h} HOTI phase of $H_{\text{HOTI}}^{P6/mmm}(\mathbf{k})$, we choose $\Delta_0 = 2$ and $\Delta_1 = 0.4$ in SEqs. (398) and (399). We have chosen a relatively small value of Δ_1 to ensure that the band ordering remains the same as in the $\mathcal{T}$-symmetric limit in which Δ_0 vanishes. Specifically, as discussed in SN 31 r, in the $\mathcal{T}$-symmetric limit, $H_{\text{HOTI}}^{P6/mmm}(\mathbf{k})$ realizes a sixfold-rotation-anomaly, helical, nonmagnetic HOTI phase[26,27,36] indicated by the double SIs $(z_{12}, z_{6m,\pi}^+) = (60)$ in

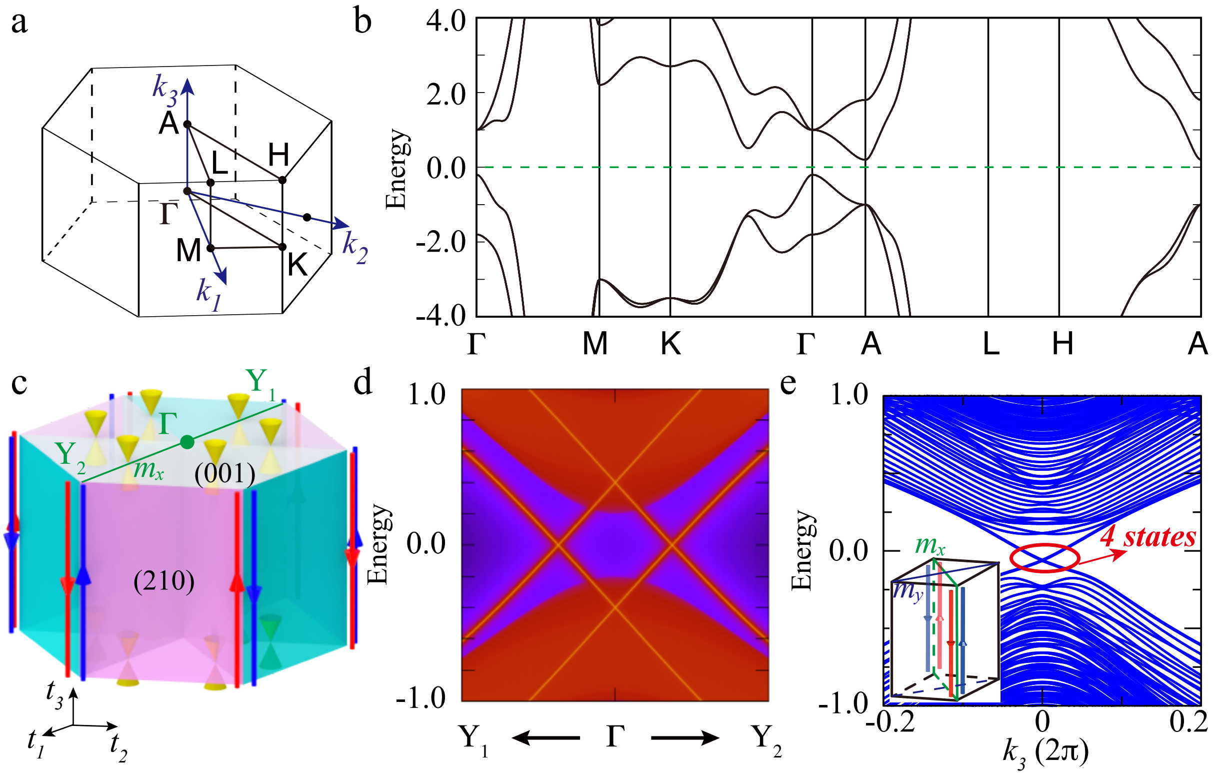

Supplementary Figure 25: Surface and hinge states of the helical magnetic D_{6h} HOTI phase in double MSG 191.233 $P6/mmm$. (a) The bulk BZ. (b) The bulk band structure obtained from SEqs. (398) and (399) with $\Delta_0 = 2$ and $\Delta_1 = 0.4$. We note that SEq. (398) contains additional symmetries beyond those of double MSG 191.233 $P6/mmm$, such that the band structure in (b) exhibits additional degeneracies away from the Fermi level – such as the unoccupied fourfold degeneracy at Γ – that are not robust to symmetry-preserving perturbations. (c) Schematic of the top ($\hat{\mathbf{z}}$-normal) surface states and nanorod hinge states. The top surface of the hexagonal nanorod in (c) respects the symmetries of Type-I double magnetic wallpaper group $p6m$, and the hinges respect the symmetries of frieze groups with mirror lines parallel to the hinge translation direction (see SN 31 and 34 and SRefs. 34,39,73–75). (d) The top surface spectrum plotted along $k_y = 0$ [see SEqs. (392) and (393)] obtained from surface Green's functions calculated for the model in (b) terminated in a $\hat{\mathbf{z}}$- ($\mathbf{t}_3$-) normal slab geometry. In (d), the surface bands exhibit mirror Chern $C_{m_x} = 2$ spectral flow. We have verified through surface-state calculations that the C_{6z}-related slab surface spectrum along the $C_6 m_x C_6^{-1}$- and $C_6^{-1} m_x C_6$-invariant surface mirror lines in $p6m$ [see SFig. 22(c)] also exhibits mirror Chern $C_m = 2$ spectral flow, that the surface spectrum along the other three surface mirror lines [*i.e.* the m_y-, $C_6 m_y C_6^{-1}$-, and $C_6^{-1} m_y C_6$-invariant lines] exhibits trivial $C_m = 0$ spectral flow, and that $C_{m_z} = 0$. Together, this implies that the top surface exhibits six twofold Dirac cones, circumventing the fermion multiplication theorem for double magnetic wallpaper group $p6m$ derived in SN 34, and implies that the bulk is a D_{6h} HOTI. (e) Unlike in SFigs. 23(e) and 24(e), it is numerically simpler to implement a hinge-state calculation in which the bulk insulator in (b) is cut into a z-directed nanorod that preserves $\mathcal{I}$ and $m_{x,y}$ symmetries, but does not preserve C_{3z} and C_{6z} rotation symmetries [see the inset panel in (e)]. In (e), we show the spectrum of a z-directed, $m_{x,y}$-symmetric nanorod of the model in (b); the nanorod in (e) features two pairs of helical hinge states along the m_x-invariant hinges (four total hinge states), and does not exhibit any other states crossing the gap. The nanorod spectrum in (e) implies that a D_{6h}-symmetric nanorod of the model in (b) [*i.e.* a nanorod with m_x, $C_{6z} m_x C_{6z}^{-1}$, and $C_{6z}^{-1} m_x C_{6z}$ symmetries], would feature six pairs of hinge-localized helical modes [twelve total hinge states], demonstrating that the model in (b) exhibits the higher-order spectral flow of a D_{6h} helical magnetic HOTI.

the Type-II double SG 191.234 $P6/mmm1'$. In SFig. 25(b), we plot the bulk band structure of SEqs. (398) and (399); we emphasize that SEqs. (398) and (399) contain additional, extraneous (artificial) symmetries beyond those of double MSG 191.233 $P6/mmm$. Hence, the band structure in SFig. 25(b) exhibits additional degeneracies away from the Fermi level – such as the unoccupied fourfold degeneracy at Γ – that are not robust to symmetry-preserving perturbations.

To diagnose the topology of SEqs. (398) and (399), we will perform two sets of calculations. First, we will calculate the double SIs of the four occupied bands. Then, we will demonstrate the presence of anomalous surface and hinge states when SEqs. (398) and (399) are terminated in a finite, D_{6h}-symmetric nanorod geometry [SFig. 25(c)]. To begin, in Supplementary Table 14, we list the double-valued small irreps that correspond to the four occupied spinful Bloch eigenstates at the six high-symmetry $\mathbf{k}$ points shown in SFig. 25(a). From the matrix representative $\Delta_\sigma(h)$ of

Bands		$\Gamma(000)$	$A(00\pi)$
1-2	Energy	-1.8	-1
	σ	$\overline{\Gamma}_8$	$\overline{A}_{11}$
	$\Delta_\sigma(\mathcal{I})$	ξ^0	$-\xi^0$
	$\Delta_\sigma(C_{6z})$	$e^{-i\frac{5\pi}{6}\xi^z}$	$e^{-i\frac{5\pi}{6}\xi^z}$
	$\Delta_\sigma(m_z)$	$-i\xi^z$	$i\xi^z$
3-4	Energy	-0.2	-1
	σ	$\overline{\Gamma}_9$	$\overline{A}_{12}$
	$\Delta_\sigma(\mathcal{I})$	ξ^0	$-\xi^0$
	$\Delta_\sigma(C_{6z})$	$e^{-i\frac{\pi}{6}\xi^z}$	$e^{-i\frac{\pi}{6}\xi^z}$
	$\Delta_\sigma(m_z)$	$-i\xi^z$	$i\xi^z$

Bands		$K(\frac{2\pi}{3}\frac{2\pi}{3}0)$	$H(\frac{2\pi}{3}\frac{2\pi}{3}\pi)$
1-2	Energy	-3.5	-5.5
	σ	$\overline{K}_8$	$\overline{H}_8$
	$\Delta_\sigma(C_{3z})$	$e^{-i\frac{\pi}{3}\xi^z}$	$e^{-i\frac{\pi}{3}\xi^z}$
	$\Delta_\sigma(m_z)$	$-i\xi^z$	$-i\xi^z$
3-4	Energy	-3.5	-5.5
	σ	$\overline{K}_9$	$\overline{H}_9$
	$\Delta_\sigma(C_{3z})$	$e^{i\frac{\pi}{3}\xi^z}$	$e^{i\frac{\pi}{3}\xi^z}$
	$\Delta_\sigma(m_z)$	$-i\xi^z$	$-i\xi^z$

Bands		$M(\pi00)$	$L(\pi0\pi)$
1-2	Energy	-3	-5
	σ	$\overline{M}_6$	$\overline{L}_6$
	$\Delta_\sigma(\mathcal{I})$	$-\xi^0$	$-\xi^0$
	$\Delta_\sigma(m_z)$	$i\xi^z$	$i\xi^z$
3-4	Energy	-3	-5
	σ	$\overline{M}_6$	$\overline{L}_6$
	$\Delta_\sigma(\mathcal{I})$	$-\xi^0$	$-\xi^0$
	$\Delta_\sigma(m_z)$	$i\xi^z$	$i\xi^z$

Supplementary Table 14: The double-valued small irreps corresponding to the four occupied bulk bands of the helical D_{6h} magnetic HOTI phase of SEq. (398) [SFig. 25(b)]. At one **k** point in each of the six maximal momentum stars in MSG 191.233 $P6/mmm$ [given in the notation $\mathbf{k}(k_1k_2k_3)$ and obtained through MKVEC, see SN 12 and SFig. 25(a)], we list the occupied band index and energy, the label of the double-valued small irrep σ that corresponds to each pair of occupied Bloch states at **k** in the notation of the Corepresentations tool [see SN 13], and the matrix representatives $\Delta_\sigma(h)$ of the representative unitary symmetries h of the little group $G_{\mathbf{k}}$ [see SEq. (71) and the surrounding text] in the basis of the 2×2 Pauli matrices ξ^i.

each two-dimensional small irrep for each of the representative unitary symmetries h of the little group $G_{\mathbf{k}}$ [*e.g.* C_{6z}, C_{3z}, and $\mathcal{I}$, see SEq. (71) and the surrounding text], we may infer the symmetry eigenvalues of the four occupied bands.

In SN 31 r, we previously expressed the double SI z_{12} in terms of other double SIs in double MSGs with lower symmetry than double MSG 191.233 $P6/mmm$ [SEq. (327)]:

$$z_{12} = \delta_{6m} + 3[(\delta_{6m} - z_4) \bmod 4] \bmod 12, \tag{401}$$

where z_4 and δ_{6m} are respectively defined in SEqs. (259) and (319). Using the matrix representatives in Supplementary Table 14, we first determine the parity eigenvalue multiplicities:

$$n_\Gamma^- = 0,\ n_\Gamma^+ = 4,\ n_A^- = 4,\ n_A^+ = 0,\ n_M^- = 4,\ n_M^+ = 0,\ n_L^- = 4,\ n_L^+ = 0. \tag{402}$$

In MSG 191.233 $P6/mmm$, the M and L points lie within multiplicity-3 momentum stars (see SN 12 and MKVEC); therefore, the eight $\mathcal{I}$-invariant momenta in MSG 191.233 $P6/mmm$ are given by:

$$k_{\mathcal{I}} = \left\{\Gamma, A, M, (C_{6z})M, (C_{6z})^2M, L, (C_{6z})L, (C_{6z})^2L\right\}. \tag{403}$$

SEqs. (402) and (403) imply that:

$$\begin{aligned} z_4 &= \sum_{K\in K_{\mathcal{I}}} \frac{n_K^- - n_K^+}{4} \bmod 4 \\ &= \frac{n_\Gamma^- - n_\Gamma^+}{4} + \frac{n_A^- - n_A^+}{4} + 3\frac{n_M^- - n_M^+}{4} + 3\frac{n_L^- - n_L^+}{4} \bmod 4 \\ &= -1 + 1 + 3 + 3 \bmod 4 = 2. \end{aligned} \tag{404}$$

Next, to compute δ_{6m}, we use Supplementary Table 14 to obtain the rotation eigenvalues in each mirror sector:

$$\begin{aligned} n_A^{\frac{1}{2},i} &= 1,\ n_A^{-\frac{1}{2},i} = 0,\ n_A^{\frac{3}{2},i} = 0,\ n_A^{-\frac{3}{2},i} = 0,\ n_A^{\frac{5}{2},i} = 1,\ n_A^{-\frac{5}{2},i} = 0, \\ n_H^{\frac{1}{2},i} &= 1,\ n_H^{-\frac{1}{2},i} = 1,\ n_H^{\frac{3}{2},i} = 0,\ n_L^{\frac{1}{2},i} = 2,\ n_L^{-\frac{1}{2},i} = 0, \\ n_\Gamma^{\frac{1}{2},-i} &= 1,\ n_\Gamma^{-\frac{1}{2},-i} = 0,\ n_\Gamma^{\frac{3}{2},-i} = 0,\ n_\Gamma^{-\frac{3}{2},-i} = 0,\ n_\Gamma^{\frac{5}{2},-i} = 1,\ n_\Gamma^{-\frac{5}{2},-i} = 0, \\ n_K^{\frac{1}{2},-i} &= 1,\ n_K^{-\frac{1}{2},-i} = 1,\ n_K^{\frac{3}{2},-i} = 0,\ n_M^{\frac{1}{2},-i} = 0,\ n_M^{-\frac{1}{2},-i} = 2. \end{aligned} \tag{405}$$

Using SEqs. (319) and (405), we then compute δ_{6m}:

$$\begin{aligned}\delta_{6m} &= -\frac{1}{2}n_A^{\frac{1}{2},+i}+\frac{1}{2}n_A^{-\frac{1}{2},+i}-\frac{3}{2}n_A^{\frac{3}{2},+i}+\frac{3}{2}n_A^{-\frac{3}{2},+i}-\frac{5}{2}n_A^{\frac{5}{2},+i}+\frac{5}{2}n_A^{-\frac{5}{2},+i}\\ &- n_H^{\frac{1}{2},+i}+n_H^{-\frac{1}{2},+i}+3n_H^{\frac{3}{2},+i}+\frac{3}{2}n_L^{\frac{1}{2},+i}-\frac{3}{2}n_L^{-\frac{1}{2},+i}\\ &+ \frac{1}{2}n_\Gamma^{\frac{1}{2},-i}-\frac{1}{2}n_\Gamma^{-\frac{1}{2},-i}+\frac{3}{2}n_\Gamma^{\frac{3}{2},-i}-\frac{3}{2}n_\Gamma^{-\frac{3}{2},-i}+\frac{5}{2}n_\Gamma^{\frac{5}{2},-i}-\frac{5}{2}n_\Gamma^{-\frac{5}{2},-i}\\ &+ n_K^{\frac{1}{2},-i}-n_K^{-\frac{1}{2},-i}-3n_K^{\frac{3}{2},-i}-\frac{3}{2}n_M^{\frac{1}{2},-i}+\frac{3}{2}n_M^{-\frac{1}{2},-i} \mod 6\\ &= (-\frac{1}{2}-\frac{5}{2}-1+1+3)+(\frac{1}{2}+\frac{5}{2}+1-1+3) \mod 6 = 0.\end{aligned} \tag{406}$$

From SEqs. (401), (404), and (406), we next compute z_{12}:

$$z_{12}=\delta_{6m}+3[(\delta_{6m}-z_4) \mod 4] \mod 12 = 0+3\times 2 \mod 12 = 6. \tag{407}$$

Lastly, to complete the calculation of the double SIs in MSG 191.233 $P6/mmm$, we compute $z^+_{6m,\pi}$ [SEq. (320)]:

$$\begin{aligned}z^+_{6m,\pi} &= -\frac{1}{2}n_A^{\frac{1}{2},+i}+\frac{1}{2}n_A^{-\frac{1}{2},+i}-\frac{3}{2}n_A^{\frac{3}{2},+i}+\frac{3}{2}n_A^{-\frac{3}{2},+i}-\frac{5}{2}n_A^{\frac{5}{2},+i}+\frac{5}{2}n_A^{-\frac{5}{2},+i}\\ &- n_H^{\frac{1}{2},+i}+n_H^{-\frac{1}{2},+i}+3n_H^{\frac{3}{2},+i}+\frac{3}{2}n_L^{\frac{1}{2},+i}-\frac{3}{2}n_L^{-\frac{1}{2},+i} \mod 6\\ &= -\frac{1}{2}-\frac{5}{2}-1+1+3 \mod 6 = 0.\end{aligned} \tag{408}$$

From SEqs. (407) and (408), we determine that the occupied bands of SEqs. (398) and (399) shown in SFig. 25(b) exhibit the double SIs $(z_{12}, z^+_{6m,\pi}) = (60)$.

Previously, in SN 31 r, we showed that the double SIs $(z_{12}, z^+_{6m,\pi}) = (60)$ in double MSG 191.233 $P6/mmm$ either indicate a mirror TCI with mirror Chern number $C_{m_z} \mod 12 = 6$ or indicate a helical D_{6h} HOTI phase in which half of the z-projecting mirror planes [*e.g.* the $\{m_x|\mathbf{0}\}$-, $\{C_{6z}m_xC_{6z}^{-1}|\mathbf{0}\}$-, and $\{C_{6z}^{-1}m_xC_{6z}|\mathbf{0}\}$-invariant planes] exhibit $C_m \mod 4 = 2$, the other half [*e.g.* the $\{m_y|\mathbf{0}\}$-, $\{C_{6z}m_yC_{6z}^{-1}|\mathbf{0}\}$-, and $\{C_{6z}^{-1}m_yC_{6z}|\mathbf{0}\}$-invariant planes] exhibit $C_m \mod 4 = 0$, and $C_{m_z} = 0$ [see SFig. 21(c)]. To demonstrate that SEq. (398), with the parameters used to obtain SFig. 25(b), is a D_{6h} HOTI, we have performed two boundary state calculations. First, as shown in SFig. 25(d), we have calculated the top surface spectrum of $H^{P6/mmm}_{\text{HOTI}}(\mathbf{k})$ terminated in a $\hat{\mathbf{z}}$- ($\mathbf{t}_3$-) normal slab geometry. The top surface of a crystal in double MSG 191.233 $P6/mmm$ respects the symmetries of Type-I magnetic wallpaper group $p6m$ (see SN 31 and 34 and SRefs. 34,39,73–75). The slab surface spectrum in SFig. 25(d) exhibits six twofold Dirac cones, circumventing the fermion multiplication theorem for double magnetic wallpaper group $p6m$ derived in SN 34. We then calculate the spectrum of an infinite, z-directed, $m_{x,y}$-symmetric nanorod of $H^{P6/mmm}_{\text{HOTI}}(\mathbf{k})$, which we find to exhibit the higher-order spectral flow of a D_{6h} HOTI [SFig. 25(e)].

36. Guide to the Larger Supplementary Tables

In the sections below, we will provide further supplementary tables containing additional data generated for this work. First, in SN 37, we will provide a complete tabulation of the exceptional composite band (co)reps of the 1,651 single and double SSGs [see SN 24]. Then, in SN 38, we will tabulate the minimum and maximum EBR dimension in each single and double SSG. Finally, in SN 39, we will list the minimal double SSG with the minimal double SIs on which the double SIs in each double SSGs are dependent (see SN 30).

37. Exceptional Composite Band Coreps Induced from Maximal Site-Symmetry Groups

In this section, we provide a complete tabulation of the exceptional cases [defined in detail in SN 24] in the 1,651 single and double SSGs in which an irreducible (co)rep of a site-symmetry group of a site in a maximal Wyckoff position does not induce an elementary band (co)rep [EBR]. For the Type-I MSGs and Type-II SGs analyzed in TQC[1–6], the exceptional cases listed in the tables below agree with the previous tabulations performed in SRefs. 1,4. Among the tables provided in this section, there is no table of exceptional cases in the Type-II double SGs, because, as previously shown in SRefs. 1,4 and in Supplementary Table 9, there are no exceptional composite band coreps in the Type-II double SGs.

Supplementary Table 15: Exceptional composite band reps induced from site-symmetry irreps in the Type-I single MSGs (SN 3). In order, the columns in this table list the number of the MSG in the BNS setting and the symbol of the MSG, the letter of the maximal Wyckoff position containing $\mathbf{q}$ and the single-valued irrep of the site-symmetry group $G_{\mathbf{q}}$, the symbol of the MPG isomorphic to the site-symmetry group $G_{\mathbf{q}}$ in the Hermann-Mauguin notation of the MPOINT tool on the BCS[15–18] and the number of the MPG isomorphic to $G_{\mathbf{q}}$ in the convention established by Litvin in SRef. 10, the symbol and number of the MPG isomorphic to the reducing group $G_{\mathbf{q}'}$, the symbol and number of the MPG isomorphic to the intersection group $G_{\mathbf{q}_0} = G_{\mathbf{q}} \cap G_{\mathbf{q}'}$, and the dimension d of the exceptional composite band rep. See SN 24 for further information regarding exceptional composite band reps.

MSG		Irrep	$G_{\mathbf{q}}$		$G_{\mathbf{q}'}$		$G_{\mathbf{q}_0}$		d
124.351	$P4/mcc$	a,E	422	12.1.40	$4/m$	11.1.35	4	9.1.29	4
124.351	$P4/mcc$	c,E	422	12.1.40	$4/m$	11.1.35	4	9.1.29	4
131.435	$P4_2/mmc$	e,E	$\bar{4}2m$	14.1.48	mmm	8.1.24	mm2	7.1.20	4
131.435	$P4_2/mmc$	f,E	$\bar{4}2m$	14.1.48	mmm	8.1.24	mm2	7.1.20	4
132.447	$P4_2/mcm$	b,E	$\bar{4}2m$	14.1.48	mmm	8.1.24	mm2	7.1.20	4
132.447	$P4_2/mcm$	d,E	$\bar{4}2m$	14.1.48	mmm	8.1.24	mm2	7.1.20	4
139.531	$I4/mmm$	d,E	$\bar{4}2m$	14.1.48	mmm	8.1.24	mm2	7.1.20	4
140.541	$I4/mcm$	a,E	422	12.1.40	$4/m$	11.1.35	4	9.1.29	4
140.541	$I4/mcm$	b,E	$\bar{4}2m$	14.1.48	mmm	8.1.24	mm2	7.1.20	4
163.79	$P\bar{3}1c$	a,E	32	18.1.65	$\bar{3}$	17.1.62	3	16.1.60	4
165.91	$P\bar{3}c1$	a,E	32	18.1.65	$\bar{3}$	17.1.62	3	16.1.60	4
167.103	$R\bar{3}c$	a,E	32	18.1.65	$\bar{3}$	17.1.62	3	16.1.60	4
188.215	$P\bar{6}c2$	a,E	32	18.1.65	$\bar{6}$	22.1.79	3	16.1.60	4
188.215	$P\bar{6}c2$	c,E	32	18.1.65	$\bar{6}$	22.1.79	3	16.1.60	4
188.215	$P\bar{6}c2$	e,E	32	18.1.65	$\bar{6}$	22.1.79	3	16.1.60	4
190.227	$P\bar{6}2c$	a,E	32	18.1.65	$\bar{6}$	22.1.79	3	16.1.60	4
192.243	$P6/mcc$	a,E_2	622	24.1.87	$6/m$	23.1.82	6	21.1.76	4
192.243	$P6/mcc$	a,E_1	622	24.1.87	$6/m$	23.1.82	6	21.1.76	4
192.243	$P6/mcc$	c,E	32	18.1.65	$\bar{6}$	22.1.79	3	16.1.60	8
193.253	$P6_3/mcm$	d,E	32	18.1.65	$\bar{6}$	22.1.79	3	16.1.60	8
207.40	$P432$	c,E	422	12.1.40	432	30.1.112	4	9.1.29	6
207.40	$P432$	d,E	422	12.1.40	432	30.1.112	4	9.1.29	6
208.44	$P4_232$	b,E	32	18.1.65	23	28.1.107	3	16.1.60	8
208.44	$P4_232$	c,E	32	18.1.65	23	28.1.107	3	16.1.60	8
210.52	$F4_132$	c,E	32	18.1.65	23	28.1.107	3	16.1.60	8
210.52	$F4_132$	d,E	32	18.1.65	23	28.1.107	3	16.1.60	8
211.56	$I432$	b,E	422	12.1.40	432	30.1.112	4	9.1.29	6
211.56	$I432$	c,E	32	18.1.65	432	30.1.112	3	16.1.60	8
215.70	$P\bar{4}3m$	c,E	$\bar{4}2m$	14.1.48	$\bar{4}3m$	31.1.115	mm2	7.1.20	6
215.70	$P\bar{4}3m$	d,E	$\bar{4}2m$	14.1.48	$\bar{4}3m$	31.1.115	mm2	7.1.20	6
217.78	$I\bar{4}3m$	b,E	$\bar{4}2m$	14.1.48	$\bar{4}3m$	31.1.115	mm2	7.1.20	6

222.98	$Pn\bar{3}n$	b,E	422	12.1.40	432	30.1.112	4	9.1.29	12
223.104	$Pm\bar{3}n$	c,E	$\bar{4}2m$	14.1.48	mmm	8.1.24	mm2	7.1.20	12
223.104	$Pm\bar{3}n$	d,E	$\bar{4}2m$	14.1.48	mmm	8.1.24	mm2	7.1.20	12
223.104	$Pm\bar{3}n$	e,E	32	18.1.65	$m\bar{3}$	29.1.109	3	16.1.60	16
224.110	$Pn\bar{3}m$	d,E	$\bar{4}2m$	14.1.48	$\bar{4}3m$	31.1.115	mm2	7.1.20	12
226.122	$Fm\bar{3}c$	c,E	$\bar{4}2m$	14.1.48	$m\bar{3}$	29.1.109	mm2	7.1.20	12
228.134	$Fd\bar{3}c$	b,E	32	18.1.65	23	28.1.107	3	16.1.60	16
			32	18.1.65	$\bar{3}$	17.1.62	3	16.1.60	16
229.140	$Im\bar{3}m$	d,E	$\bar{4}2m$	14.1.48	$4/mmm$	15.1.53	mm2	7.1.20	12
230.145	$Ia\bar{3}d$	b,E	32	18.1.65	$\bar{3}$	17.1.62	3	16.1.60	16

Supplementary Table 16: Exceptional composite band reps induced from site-symmetry irreps in the Type-I double MSGs (SN 3). In order, the columns in this table list the number of the MSG in the BNS setting and the symbol of the MSG, the letter of the maximal Wyckoff position containing $\mathbf{q}$ and the double-valued irrep of the site-symmetry group $G_{\mathbf{q}}$, the symbol of the MPG isomorphic to the site-symmetry group $G_{\mathbf{q}}$ in the Hermann-Mauguin notation of the MPOINT tool on the BCS[15–18] and the number of the MPG isomorphic to $G_{\mathbf{q}}$ in the convention established by Litvin in SRef. 10, the symbol and number of the MPG isomorphic to the reducing group $G_{\mathbf{q}'}$, the symbol and number of the MPG isomorphic to the intersection group $G_{\mathbf{q}_0} = G_{\mathbf{q}} \cap G_{\mathbf{q}'}$, and the dimension d of the exceptional composite band rep. See SN 24 for further information regarding exceptional composite band reps.

SSG		Irrep	$G_{\mathbf{q}}$		$G_{\mathbf{q}'}$		$G_{\mathbf{q}_0}$		d
49.265	$Pccm$	e,$\bar{E}$	222	6.1.17	$2/m$	5.1.12	2	3.1.6	4
49.265	$Pccm$	f,$\bar{E}$	222	6.1.17	$2/m$	5.1.12	2	3.1.6	4
49.265	$Pccm$	g,$\bar{E}$	222	6.1.17	$2/m$	5.1.12	2	3.1.6	4
49.265	$Pccm$	h,$\bar{E}$	222	6.1.17	$2/m$	5.1.12	2	3.1.6	4
51.289	$Pmma$	e,$\bar{E}$	$mm2$	7.1.20	$2/m$	5.1.12	m	4.1.9	4
51.289	$Pmma$	f,$\bar{E}$	$mm2$	7.1.20	$2/m$	5.1.12	m	4.1.9	4
63.457	$Cmcm$	c,$\bar{E}$	$mm2$	7.1.20	$2/m$	5.1.12	m	4.1.9	4
66.491	$Cccm$	a,$\bar{E}$	222	6.1.17	$2/m$	5.1.12	2	3.1.6	4
66.491	$Cccm$	b,$\bar{E}$	222	6.1.17	$2/m$	5.1.12	2	3.1.6	4
67.501	$Cmma$	a,$\bar{E}$	222	6.1.17	$2/m$	5.1.12	2	3.1.6	4
67.501	$Cmma$	b,$\bar{E}$	222	6.1.17	$2/m$	5.1.12	2	3.1.6	4
67.501	$Cmma$	g,$\bar{E}$	$mm2$	7.1.20	$2/m$	5.1.12	m	4.1.9	4
69.521	$Fmmm$	f,$\bar{E}$	222	6.1.17	$2/m$	5.1.12	2	3.1.6	4
72.539	$Ibam$	a,$\bar{E}$	222	6.1.17	$2/m$	5.1.12	2	3.1.6	4
72.539	$Ibam$	b,$\bar{E}$	222	6.1.17	$2/m$	5.1.12	2	3.1.6	4
74.554	$Imma$	e,$\bar{E}$	$mm2$	7.1.20	$2/m$	5.1.12	m	4.1.9	4
89.87	$P422$	e,$\bar{E}$	222	6.1.17	422	12.1.40	2	3.1.6	4
89.87	$P422$	f,$\bar{E}$	222	6.1.17	422	12.1.40	2	3.1.6	4
97.151	$I422$	c,$\bar{E}$	222	6.1.17	422	12.1.40	2	3.1.6	4
99.163	$P4mm$	c,$\bar{E}$	$mm2$	7.1.20	$4mm$	13.1.44	m	4.1.9	4
107.227	$I4mm$	b,$\bar{E}$	$mm2$	7.1.20	$4mm$	13.1.44	m	4.1.9	4
111.251	$P\bar{4}2m$	e,$\bar{E}$	222	6.1.17	$\bar{4}2m$	14.1.48	2	3.1.6	4
111.251	$P\bar{4}2m$	f,$\bar{E}$	222	6.1.17	$\bar{4}2m$	14.1.48	2	3.1.6	4
112.259	$P\bar{4}2c$	a,$\bar{E}$	222	6.1.17	$\bar{4}$	10.1.32	2	3.1.6	4
112.259	$P\bar{4}2c$	c,$\bar{E}$	222	6.1.17	$\bar{4}$	10.1.32	2	3.1.6	4
115.283	$P\bar{4}m2$	g,$\bar{E}$	$mm2$	7.1.20	$\bar{4}2m$	14.1.48	m	4.1.9	4
116.291	$P\bar{4}c2$	a,$\bar{E}$	222	6.1.17	$\bar{4}$	10.1.32	2	3.1.6	4
116.291	$P\bar{4}c2$	b,$\bar{E}$	222	6.1.17	$\bar{4}$	10.1.32	2	3.1.6	4
120.321	$I\bar{4}c2$	a,$\bar{E}$	222	6.1.17	$\bar{4}$	10.1.32	2	3.1.6	4
120.321	$I\bar{4}c2$	d,$\bar{E}$	222	6.1.17	$\bar{4}$	10.1.32	2	3.1.6	4
121.327	$I\bar{4}2m$	c,$\bar{E}$	222	6.1.17	$\bar{4}2m$	14.1.48	2	3.1.6	4
			222	6.1.17	$\bar{4}$	10.1.32	2	3.1.6	4
124.351	$P4/mcc$	a,$\bar{E}_2$	422	12.1.40	$4/m$	11.1.35	4	9.1.29	4
124.351	$P4/mcc$	a,$\bar{E}_1$	422	12.1.40	$4/m$	11.1.35	4	9.1.29	4
124.351	$P4/mcc$	c,$\bar{E}_2$	422	12.1.40	$4/m$	11.1.35	4	9.1.29	4
124.351	$P4/mcc$	c,$\bar{E}_1$	422	12.1.40	$4/m$	11.1.35	4	9.1.29	4
124.351	$P4/mcc$	f,$\bar{E}$	222	6.1.17	422	12.1.40	2	3.1.6	8
			222	6.1.17	$2/m$	5.1.12	2	3.1.6	8

126.375	$P4/nnc$	c,$\overline{E}$	222	6.1.17	422	12.1.40	2	3.1.6	8
			222	6.1.17	$\bar{4}$	10.1.32	2	3.1.6	8
128.399	$P4/mnc$	d,$\overline{E}$	222	6.1.17	$2/m$	5.1.12	2	3.1.6	8
130.423	$P4/ncc$	a,$\overline{E}$	222	6.1.17	$\bar{4}$	10.1.32	2	3.1.6	8
132.447	$P4_2/mcm$	e,$\overline{E}$	222	6.1.17	$\bar{4}2m$	14.1.48	2	3.1.6	8
			222	6.1.17	$2/m$	5.1.12	2	3.1.6	8
133.459	$P4_2/nbc$	b,$\overline{E}$	222	6.1.17	$\bar{4}$	10.1.32	2	3.1.6	8
134.471	$P4_2/nnm$	c,$\overline{E}$	222	6.1.17	$\bar{4}2m$	14.1.48	2	3.1.6	8
134.471	$P4_2/nnm$	d,$\overline{E}$	222	6.1.17	$2/m$	5.1.12	2	3.1.6	8
135.483	$P4_2/mbc$	d,$\overline{E}$	222	6.1.17	$2/m$	5.1.12	2	3.1.6	8
137.507	$P4_2/nmc$	d,$\overline{E}$	$mm2$	7.1.20	$\bar{4}2m$	14.1.48	m	4.1.9	8
138.519	$P4_2/ncm$	a,$\overline{E}$	222	6.1.17	$\bar{4}$	10.1.32	2	3.1.6	8
			222	6.1.17	$2/m$	5.1.12	2	3.1.6	8
138.519	$P4_2/ncm$	e,$\overline{E}$	$mm2$	7.1.20	$2/m$	5.1.12	m	4.1.9	8
140.541	$I4/mcm$	a,$\overline{E}_2$	422	12.1.40	$4/m$	11.1.35	4	9.1.29	4
140.541	$I4/mcm$	a,$\overline{E}_1$	422	12.1.40	$4/m$	11.1.35	4	9.1.29	4
142.561	$I4_1/acd$	b,$\overline{E}$	222	6.1.17	$\bar{4}$	10.1.32	2	3.1.6	8
163.79	$P\bar{3}1c$	a,$\overline{E}_1$	32	18.1.65	$\bar{3}$	17.1.62	3	16.1.60	4
165.91	$P\bar{3}c1$	a,$\overline{E}_1$	32	18.1.65	$\bar{3}$	17.1.62	3	16.1.60	4
167.103	$R\bar{3}c$	a,$\overline{E}_1$	32	18.1.65	$\bar{3}$	17.1.62	3	16.1.60	4
177.149	$P622$	f,$\overline{E}$	222	6.1.17	622	24.1.87	2	3.1.6	6
			222	6.1.17	32	18.1.65	2	3.1.6	6
177.149	$P622$	g,$\overline{E}$	222	6.1.17	622	24.1.87	2	3.1.6	6
			222	6.1.17	32	18.1.65	2	3.1.6	6
183.185	$P6mm$	c,$\overline{E}$	$mm2$	7.1.20	$6mm$	25.1.91	m	4.1.9	6
			$mm2$	7.1.20	$3m$	19.1.68	m	4.1.9	6
188.215	$P\bar{6}c2$	a,$\overline{E}_1$	32	18.1.65	$\bar{6}$	22.1.79	3	16.1.60	4
188.215	$P\bar{6}c2$	c,$\overline{E}_1$	32	18.1.65	$\bar{6}$	22.1.79	3	16.1.60	4
188.215	$P\bar{6}c2$	e,$\overline{E}_1$	32	18.1.65	$\bar{6}$	22.1.79	3	16.1.60	4
190.227	$P\bar{6}2c$	a,$\overline{E}_1$	32	18.1.65	$\bar{6}$	22.1.79	3	16.1.60	4
192.243	$P6/mcc$	a,$\overline{E}_3$	622	24.1.87	$6/m$	23.1.82	6	21.1.76	4
192.243	$P6/mcc$	a,$\overline{E}_2$	622	24.1.87	$6/m$	23.1.82	6	21.1.76	4
192.243	$P6/mcc$	a,$\overline{E}_1$	622	24.1.87	$6/m$	23.1.82	6	21.1.76	4
192.243	$P6/mcc$	c,$\overline{E}_1$	32	18.1.65	$\bar{6}$	22.1.79	3	16.1.60	8
192.243	$P6/mcc$	f,$\overline{E}$	222	6.1.17	622	24.1.87	2	3.1.6	12
			222	6.1.17	32	18.1.65	2	3.1.6	12
			222	6.1.17	$2/m$	5.1.12	2	3.1.6	12
193.253	$P6_3/mcm$	a,$\overline{E}_3$	$\bar{6}m2$	26.1.95	$\bar{3}m1$	20.1.71	3m	19.1.68	4
193.253	$P6_3/mcm$	d,$\overline{E}_1$	32	18.1.65	$\bar{6}$	22.1.79	3	16.1.60	8
194.263	$P6_3/mmc$	b,$\overline{E}_3$	$\bar{6}m2$	26.1.95	$\bar{3}m1$	20.1.71	3m	19.1.68	4
195.1	$P23$	c,$\overline{E}$	222	6.1.17	23	28.1.107	2	3.1.6	6
195.1	$P23$	d,$\overline{E}$	222	6.1.17	23	28.1.107	2	3.1.6	6
197.7	$I23$	b,$\overline{E}$	222	6.1.17	23	28.1.107	2	3.1.6	6
201.18	$Pn\bar{3}$	d,$\overline{E}$	222	6.1.17	23	28.1.107	2	3.1.6	12
207.40	$P432$	c,$\overline{E}_2$	422	12.1.40	432	30.1.112	4	9.1.29	6
207.40	$P432$	c,$\overline{E}_1$	422	12.1.40	432	30.1.112	4	9.1.29	6
207.40	$P432$	d,$\overline{E}_2$	422	12.1.40	432	30.1.112	4	9.1.29	6
207.40	$P432$	d,$\overline{E}_1$	422	12.1.40	432	30.1.112	4	9.1.29	6
208.44	$P4_232$	b,$\overline{E}_1$	32	18.1.65	23	28.1.107	3	16.1.60	8
208.44	$P4_232$	c,$\overline{E}_1$	32	18.1.65	23	28.1.107	3	16.1.60	8
208.44	$P4_232$	d,$\overline{E}$	222	6.1.17	23	28.1.107	2	3.1.6	12
208.44	$P4_232$	e,$\overline{E}$	222	6.1.17	32	18.1.65	2	3.1.6	12
208.44	$P4_232$	f,$\overline{E}$	222	6.1.17	32	18.1.65	2	3.1.6	12
209.48	$F432$	d,$\overline{E}$	222	6.1.17	432	30.1.112	2	3.1.6	12
			222	6.1.17	23	28.1.107	2	3.1.6	12
210.52	$F4_132$	c,$\overline{E}_1$	32	18.1.65	23	28.1.107	3	16.1.60	8
210.52	$F4_132$	d,$\overline{E}_1$	32	18.1.65	23	28.1.107	3	16.1.60	8
211.56	$I432$	b,$\overline{E}_2$	422	12.1.40	432	30.1.112	4	9.1.29	6
211.56	$I432$	b,$\overline{E}_1$	422	12.1.40	432	30.1.112	4	9.1.29	6
211.56	$I432$	c,$\overline{E}_1$	32	18.1.65	432	30.1.112	3	16.1.60	8
211.56	$I432$	d,$\overline{E}$	222	6.1.17	422	12.1.40	2	3.1.6	12
			222	6.1.17	32	18.1.65	2	3.1.6	12
214.67	$I4_132$	c,$\overline{E}$	222	6.1.17	32	18.1.65	2	3.1.6	12
214.67	$I4_132$	d,$\overline{E}$	222	6.1.17	32	18.1.65	2	3.1.6	12
218.81	$P\bar{4}3n$	b,$\overline{E}$	222	6.1.17	23	28.1.107	2	3.1.6	12
			222	6.1.17	$\bar{4}$	10.1.32	2	3.1.6	12
222.98	$Pn\bar{3}n$	b,$\overline{E}_2$	422	12.1.40	432	30.1.112	4	9.1.29	12
222.98	$Pn\bar{3}n$	b,$\overline{E}_1$	422	12.1.40	432	30.1.112	4	9.1.29	12
223.104	$Pm\bar{3}n$	e,$\overline{E}_1$	32	18.1.65	$m\bar{3}$	29.1.109	3	16.1.60	16
224.110	$Pn\bar{3}m$	a,$\overline{F}$	$\bar{4}3m$	31.1.115	$\bar{3}m1$	20.1.71	3m	19.1.68	8

224.110	$Pn\bar{3}m$	f,$\overline{E}$	222	6.1.17	$\bar{3}m1$	20.1.71	2	3.1.6	24
			222	6.1.17	$\bar{4}2m$	14.1.48	2	3.1.6	24
225.116	$Fm\bar{3}m$	c,$\overline{F}$	$\bar{4}3m$	31.1.115	$m\bar{3}m$	32.1.118	3m	19.1.68	8
227.128	$Fd\bar{3}m$	a,$\overline{F}$	$\bar{4}3m$	31.1.115	$\bar{3}m1$	20.1.71	3m	19.1.68	8
227.128	$Fd\bar{3}m$	b,$\overline{F}$	$\bar{4}3m$	31.1.115	$\bar{3}m1$	20.1.71	3m	19.1.68	8
228.134	$Fd\bar{3}c$	b,$\overline{E}_1$	32	18.1.65	23	28.1.107	3	16.1.60	16
			32	18.1.65	$\bar{3}$	17.1.62	3	16.1.60	16
230.145	$Ia\bar{3}d$	b,$\overline{E}_1$	32	18.1.65	$\bar{3}$	17.1.62	3	16.1.60	16
230.145	$Ia\bar{3}d$	c,$\overline{E}$	222	6.1.17	32	18.1.65	2	3.1.6	24
			222	6.1.17	$\bar{4}$	10.1.32	2	3.1.6	24

Supplementary Table 17: Exceptional composite band coreps induced from site-symmetry coreps in the Type-II single SGs (SN 4). In order, the columns in this table list the number of the SG in the BNS setting and the symbol of the SG, the letter of the maximal Wyckoff position containing $\mathbf{q}$ and the single-valued corep of the site-symmetry group $G_{\mathbf{q}}$, the symbol of the SPG isomorphic to the site-symmetry group $G_{\mathbf{q}}$ in the Hermann-Mauguin notation of the MPOINT tool on the BCS[15–18] and the number of the SPG isomorphic to $G_{\mathbf{q}}$ in the convention established by Litvin in SRef. 10, the symbol and number of the SPG isomorphic to the reducing group $G_{\mathbf{q}'}$, the symbol and number of the SPG isomorphic to the intersection group $G_{\mathbf{q}_0} = G_{\mathbf{q}} \cap G_{\mathbf{q}'}$, and the dimension d of the exceptional composite band corep. See SN 24 for further information regarding exceptional composite band coreps.

SG		Corep	$G_{\mathbf{q}}$		$G_{\mathbf{q}'}$		$G_{\mathbf{q}_0}$		d
84.52	$P4_2/m1'$	e, ${}^1E\,{}^2E$	$\bar{4}1'$	10.2.33	$2/m1'$	5.2.13	21'	3.2.7	4
84.52	$P4_2/m1'$	f, ${}^1E\,{}^2E$	$\bar{4}1'$	10.2.33	$2/m1'$	5.2.13	21'	3.2.7	4
87.76	$I4/m1'$	d, ${}^1E\,{}^2E$	$\bar{4}1'$	10.2.33	$2/m1'$	5.2.13	21'	3.2.7	4
112.260	$P\bar{4}2c1'$	e, ${}^1E\,{}^2E$	$\bar{4}1'$	10.2.33	$2221'$	6.2.18	21'	3.2.7	4
112.260	$P\bar{4}2c1'$	f, ${}^1E\,{}^2E$	$\bar{4}1'$	10.2.33	$2221'$	6.2.18	21'	3.2.7	4
116.292	$P\bar{4}c21'$	c, ${}^1E\,{}^2E$	$\bar{4}1'$	10.2.33	$2221'$	6.2.18	21'	3.2.7	4
116.292	$P\bar{4}c21'$	d, ${}^1E\,{}^2E$	$\bar{4}1'$	10.2.33	$2221'$	6.2.18	21'	3.2.7	4
120.322	$I\bar{4}c21'$	b, ${}^1E\,{}^2E$	$\bar{4}1'$	10.2.33	$2221'$	6.2.18	21'	3.2.7	4
120.322	$I\bar{4}c21'$	c, ${}^1E\,{}^2E$	$\bar{4}1'$	10.2.33	$2221'$	6.2.18	21'	3.2.7	4
121.328	$I\bar{4}2m1'$	d, ${}^1E\,{}^2E$	$\bar{4}1'$	10.2.33	$2221'$	6.2.18	21'	3.2.7	4
126.376	$P4/nnc1'$	d, ${}^1E\,{}^2E$	$\bar{4}1'$	10.2.33	$2221'$	6.2.18	21'	3.2.7	8
130.424	$P4/ncc1'$	b, ${}^1E\,{}^2E$	$\bar{4}1'$	10.2.33	$2221'$	6.2.18	21'	3.2.7	8
131.436	$P4_2/mmc1'$	e,E	$\bar{4}2m1'$	14.2.49	$mmm1'$	8.2.25	mm21'	7.2.21	4
131.436	$P4_2/mmc1'$	f,E	$\bar{4}2m1'$	14.2.49	$mmm1'$	8.2.25	mm21'	7.2.21	4
132.448	$P4_2/mcm1'$	b,E	$\bar{4}2m1'$	14.2.49	$mmm1'$	8.2.25	mm21'	7.2.21	4
132.448	$P4_2/mcm1'$	d,E	$\bar{4}2m1'$	14.2.49	$mmm1'$	8.2.25	mm21'	7.2.21	4
133.460	$P4_2/nbc1'$	d, ${}^1E\,{}^2E$	$\bar{4}1'$	10.2.33	$2221'$	6.2.18	21'	3.2.7	8
135.484	$P4_2/mbc1'$	b, ${}^1E\,{}^2E$	$\bar{4}1'$	10.2.33	$2/m1'$	5.2.13	21'	3.2.7	8
136.496	$P4_2/mnm1'$	d, ${}^1E\,{}^2E$	$\bar{4}1'$	10.2.33	$2/m1'$	5.2.13	21'	3.2.7	8
138.520	$P4_2/ncm1'$	b, ${}^1E\,{}^2E$	$\bar{4}1'$	10.2.33	$2221'$	6.2.18	21'	3.2.7	8
139.532	$I4/mmm1'$	d,E	$\bar{4}2m1'$	14.2.49	$mmm1'$	8.2.25	mm21'	7.2.21	4
140.542	$I4/mcm1'$	b,E	$\bar{4}2m1'$	14.2.49	$mmm1'$	8.2.25	mm21'	7.2.21	4
142.562	$I4_1/acd1'$	a, ${}^1E\,{}^2E$	$\bar{4}1'$	10.2.33	$2221'$	6.2.18	21'	3.2.7	8
215.71	$P\bar{4}3m1'$	c,E	$\bar{4}2m1'$	14.2.49	$\bar{4}3m'$	31.2.116	mm21'	7.2.21	6
215.71	$P\bar{4}3m1'$	d,E	$\bar{4}2m1'$	14.2.49	$\bar{4}3m'$	31.2.116	mm21'	7.2.21	6
217.79	$I\bar{4}3m1'$	b,E	$\bar{4}2m1'$	14.2.49	$\bar{4}3m'$	31.2.116	mm21'	7.2.21	6
217.79	$I\bar{4}3m1'$	d, ${}^1E\,{}^2E$	$\bar{4}1'$	10.2.33	$\bar{4}2m1'$	14.2.49	21'	3.2.7	12
218.82	$P\bar{4}3n1'$	c, ${}^1E\,{}^2E$	$\bar{4}1'$	10.2.33	$2221'$	6.2.18	21'	3.2.7	12
218.82	$P\bar{4}3n1'$	d, ${}^1E\,{}^2E$	$\bar{4}1'$	10.2.33	$2221'$	6.2.18	21'	3.2.7	12
219.86	$F\bar{4}3c1'$	c, ${}^1E\,{}^2E$	$\bar{4}1'$	10.2.33	$231'$	28.2.108	21'	3.2.7	12
219.86	$F\bar{4}3c1'$	d, ${}^1E\,{}^2E$	$\bar{4}1'$	10.2.33	$231'$	28.2.108	21'	3.2.7	12
222.99	$Pn\bar{3}n1'$	d, ${}^1E\,{}^2E$	$\bar{4}1'$	10.2.33	$4221'$	12.2.41	21'	3.2.7	24
223.105	$Pm\bar{3}n1'$	c,E	$\bar{4}2m1'$	14.2.49	$mmm1'$	8.2.25	mm21'	7.2.21	12
223.105	$Pm\bar{3}n1'$	d,E	$\bar{4}2m1'$	14.2.49	$mmm1'$	8.2.25	mm21'	7.2.21	12
224.111	$Pn\bar{3}m1'$	d,E	$\bar{4}2m1'$	14.2.49	$\bar{4}3m'$	31.2.116	mm21'	7.2.21	12
226.123	$Fm\bar{3}c1'$	c,E	$\bar{4}2m1'$	14.2.49	$m\bar{3}1'$	29.2.110	mm21'	7.2.21	12
228.135	$Fd\bar{3}c1'$	d, ${}^1E\,{}^2E$	$\bar{4}1'$	10.2.33	$231'$	28.2.108	21'	3.2.7	24
229.141	$Im\bar{3}m1'$	d,E	$\bar{4}2m1'$	14.2.49	$4/mmm1'$	15.2.54	mm21'	7.2.21	12
230.146	$Ia\bar{3}d1'$	d, ${}^1E\,{}^2E$	$\bar{4}1'$	10.2.33	$2221'$	6.2.18	21'	3.2.7	24

Supplementary Table 18: Exceptional composite band coreps induced from site-symmetry coreps in the Type-III single MSGs (SN 5). In order, the columns in this table list the number of the MSG in the BNS setting and the symbol of the MSG, the letter of the maximal Wyckoff position containing $\mathbf{q}$ and the single-valued corep of the site-symmetry group $G_{\mathbf{q}}$, the symbol of the MSG isomorphic to the site-symmetry group $G_{\mathbf{q}}$ in the Hermann-Mauguin notation of the MPOINT tool on the BCS[15–18] and the number of the MSG isomorphic to $G_{\mathbf{q}}$ in the convention established by Litvin in SRef. 10, the symbol and number of the MPG isomorphic to the reducing group $G_{\mathbf{q}'}$, the symbol and number of the MPG isomorphic to the intersection group $G_{\mathbf{q}_0} = G_{\mathbf{q}} \cap G_{\mathbf{q}'}$, and the dimension d of the exceptional composite band corep. See SN 24 for further information regarding exceptional composite band coreps.

MSG		Corep	$G_{\mathbf{q}}$		$G_{\mathbf{q}'}$		$G_{\mathbf{q}_0}$		d
84.53	$P4_2'/m$	e,BB	$\bar{4}'$	10.3.34	$2/m$	5.1.12	2	3.1.6	4
84.53	$P4_2'/m$	f,BB	$\bar{4}'$	10.3.34	$2/m$	5.1.12	2	3.1.6	4
84.54	$P4_2/m'$	e,BB	$\bar{4}'$	10.3.34	$2/m'$	5.4.15	2	3.1.6	4
84.54	$P4_2/m'$	f,BB	$\bar{4}'$	10.3.34	$2/m'$	5.4.15	2	3.1.6	4
87.77	$I4'/m$	d,BB	$\bar{4}'$	10.3.34	$2/m$	5.1.12	2	3.1.6	4
87.78	$I4/m'$	d,BB	$\bar{4}'$	10.3.34	$2/m'$	5.4.15	2	3.1.6	4
112.261	$P\bar{4}'2'c$	e,BB	$\bar{4}'$	10.3.34	$2'2'2$	6.3.19	2	3.1.6	4
112.261	$P\bar{4}'2'c$	f,BB	$\bar{4}'$	10.3.34	$2'2'2$	6.3.19	2	3.1.6	4
112.262	$P\bar{4}'2c'$	e,BB	$\bar{4}'$	10.3.34	222	6.1.17	2	3.1.6	4
112.262	$P\bar{4}'2c'$	f,BB	$\bar{4}'$	10.3.34	222	6.1.17	2	3.1.6	4
116.293	$P\bar{4}'c'2$	c,BB	$\bar{4}'$	10.3.34	222	6.1.17	2	3.1.6	4
116.293	$P\bar{4}'c'2$	d,BB	$\bar{4}'$	10.3.34	222	6.1.17	2	3.1.6	4
116.294	$P\bar{4}'c2'$	c,BB	$\bar{4}'$	10.3.34	$2'2'2$	6.3.19	2	3.1.6	4
116.294	$P\bar{4}'c2'$	d,BB	$\bar{4}'$	10.3.34	$2'2'2$	6.3.19	2	3.1.6	4
120.323	$I\bar{4}'c'2$	b,BB	$\bar{4}'$	10.3.34	222	6.1.17	2	3.1.6	4
120.323	$I\bar{4}'c'2$	c,BB	$\bar{4}'$	10.3.34	222	6.1.17	2	3.1.6	4
120.324	$I\bar{4}'c2'$	b,BB	$\bar{4}'$	10.3.34	$2'2'2$	6.3.19	2	3.1.6	4
120.324	$I\bar{4}'c2'$	c,BB	$\bar{4}'$	10.3.34	$2'2'2$	6.3.19	2	3.1.6	4
121.329	$I\bar{4}'2'm$	d,BB	$\bar{4}'$	10.3.34	$2'2'2$	6.3.19	2	3.1.6	4
121.330	$I\bar{4}'2m'$	d,BB	$\bar{4}'$	10.3.34	222	6.1.17	2	3.1.6	4
124.353	$P4/m'cc$	b,${}^1E\,{}^2E$	$4/m'$	11.4.38	$42'2'$	12.4.43	4	9.1.29	4
124.353	$P4/m'cc$	d,${}^1E\,{}^2E$	$4/m'$	11.4.38	$42'2'$	12.4.43	4	9.1.29	4
126.377	$P4/n'nc$	d,BB	$\bar{4}'$	10.3.34	$2'2'2$	6.3.19	2	3.1.6	8
126.378	$P4'/nn'c$	d,BB	$\bar{4}'$	10.3.34	$2'2'2$	6.3.19	2	3.1.6	8
126.379	$P4'/nnc'$	d,BB	$\bar{4}'$	10.3.34	222	6.1.17	2	3.1.6	8
126.383	$P4/n'n'c'$	d,BB	$\bar{4}'$	10.3.34	222	6.1.17	2	3.1.6	8
130.425	$P4/n'cc$	b,BB	$\bar{4}'$	10.3.34	$2'2'2$	6.3.19	2	3.1.6	8
130.426	$P4'/nc'c$	b,BB	$\bar{4}'$	10.3.34	222	6.1.17	2	3.1.6	8
130.427	$P4'/ncc'$	b,BB	$\bar{4}'$	10.3.34	$2'2'2$	6.3.19	2	3.1.6	8
130.431	$P4/n'c'c'$	b,BB	$\bar{4}'$	10.3.34	222	6.1.17	2	3.1.6	8
131.437	$P4_2/m'mc$	e,B_1B_2	$\bar{4}'2'm$	14.3.50	$m'mm$	8.3.26	mm2	7.1.20	4
131.437	$P4_2/m'mc$	f,B_1B_2	$\bar{4}'2'm$	14.3.50	$m'mm$	8.3.26	mm2	7.1.20	4
131.438	$P4_2'/mm'c$	e,B_2B_3	$\bar{4}'2m'$	14.4.51	$m'm'm$	8.4.27	m'm'2	7.4.23	4
131.438	$P4_2'/mm'c$	f,B_2B_3	$\bar{4}'2m'$	14.4.51	$m'm'm$	8.4.27	m'm'2	7.4.23	4
131.439	$P4_2'/mmc'$	e,B_1B_2	$\bar{4}'2'm$	14.3.50	mmm	8.1.24	mm2	7.1.20	4
131.439	$P4_2'/mmc'$	f,B_1B_2	$\bar{4}'2'm$	14.3.50	mmm	8.1.24	mm2	7.1.20	4
131.442	$P4_2'/m'mc'$	e,E	$\bar{4}2m$	14.1.48	$m'mm$	8.3.26	mm2	7.1.20	4
131.442	$P4_2'/m'mc'$	f,E	$\bar{4}2m$	14.1.48	$m'mm$	8.3.26	mm2	7.1.20	4
131.443	$P4_2/m'm'c'$	e,B_2B_3	$\bar{4}'2m'$	14.4.51	$m'm'm'$	8.5.28	m'm'2	7.4.23	4
131.443	$P4_2/m'm'c'$	f,B_2B_3	$\bar{4}'2m'$	14.4.51	$m'm'm'$	8.5.28	m'm'2	7.4.23	4
132.449	$P4_2/m'cm$	b,B_1B_2	$\bar{4}'2'm$	14.3.50	$m'mm$	8.3.26	mm2	7.1.20	4
132.449	$P4_2/m'cm$	d,B_1B_2	$\bar{4}'2'm$	14.3.50	$m'mm$	8.3.26	mm2	7.1.20	4
132.450	$P4_2'/mc'm$	b,B_1B_2	$\bar{4}'2'm$	14.3.50	mmm	8.1.24	mm2	7.1.20	4
132.450	$P4_2'/mc'm$	d,B_1B_2	$\bar{4}'2'm$	14.3.50	mmm	8.1.24	mm2	7.1.20	4
132.451	$P4_2'/mcm'$	b,B_2B_3	$\bar{4}'2m'$	14.4.51	$m'm'm$	8.4.27	m'm'2	7.4.23	4
132.451	$P4_2'/mcm'$	d,B_2B_3	$\bar{4}'2m'$	14.4.51	$m'm'm$	8.4.27	m'm'2	7.4.23	4
132.452	$P4_2'/m'c'm$	b,E	$\bar{4}2m$	14.1.48	$m'mm$	8.3.26	mm2	7.1.20	4
132.452	$P4_2'/m'c'm$	d,E	$\bar{4}2m$	14.1.48	$m'mm$	8.3.26	mm2	7.1.20	4
132.455	$P4_2/m'c'm'$	b,B_2B_3	$\bar{4}'2m'$	14.4.51	$m'm'm'$	8.5.28	m'm'2	7.4.23	4
132.455	$P4_2/m'c'm'$	d,B_2B_3	$\bar{4}'2m'$	14.4.51	$m'm'm'$	8.5.28	m'm'2	7.4.23	4
133.461	$P4_2/n'bc$	d,BB	$\bar{4}'$	10.3.34	$2'2'2$	6.3.19	2	3.1.6	8
133.462	$P4_2'/nb'c$	d,BB	$\bar{4}'$	10.3.34	$2'2'2$	6.3.19	2	3.1.6	8

133.463	$P4_2'/nbc'$	d,BB	$\bar{4}'$	10.3.34	222	6.1.17	2	3.1.6	8
133.467	$P4_2/n'b'c'$	d,BB	$\bar{4}'$	10.3.34	222	6.1.17	2	3.1.6	8
135.485	$P4_2/m'bc$	b,BB	$\bar{4}'$	10.3.34	$2/m'$	5.4.15	2	3.1.6	8
135.486	$P4_2'/mb'c$	b,BB	$\bar{4}'$	10.3.34	$2/m$	5.1.12	2	3.1.6	8
135.487	$P4_2'/mbc'$	b,BB	$\bar{4}'$	10.3.34	$2/m$	5.1.12	2	3.1.6	8
135.491	$P4_2/m'b'c'$	b,BB	$\bar{4}'$	10.3.34	$2/m'$	5.4.15	2	3.1.6	8
136.497	$P4_2/m'nm$	d,BB	$\bar{4}'$	10.3.34	$2/m'$	5.4.15	2	3.1.6	8
136.498	$P4_2'/mn'm$	d,BB	$\bar{4}'$	10.3.34	$2/m$	5.1.12	2	3.1.6	8
136.499	$P4_2'/mnm'$	d,BB	$\bar{4}'$	10.3.34	$2/m$	5.1.12	2	3.1.6	8
136.503	$P4_2/m'n'm'$	d,BB	$\bar{4}'$	10.3.34	$2/m'$	5.4.15	2	3.1.6	8
138.521	$P4_2/n'cm$	b,BB	$\bar{4}'$	10.3.34	$2'2'2$	6.3.19	2	3.1.6	8
138.522	$P4_2'/nc'm$	b,BB	$\bar{4}'$	10.3.34	222	6.1.17	2	3.1.6	8
138.523	$P4_2'/ncm'$	b,BB	$\bar{4}'$	10.3.34	$2'2'2$	6.3.19	2	3.1.6	8
138.527	$P4_2/n'c'm'$	b,BB	$\bar{4}'$	10.3.34	222	6.1.17	2	3.1.6	8
139.533	$I4/m'mm$	d,B_1B_2	$\bar{4}'2'm$	14.3.50	$m'mm$	8.3.26	mm2	7.1.20	4
139.534	$I4'/mm'm$	d,B_2B_3	$\bar{4}'2m'$	14.4.51	$m'm'm$	8.4.27	m'm'2	7.4.23	4
139.535	$I4'/mmm'$	d,B_1B_2	$\bar{4}'2'm$	14.3.50	mmm	8.1.24	mm2	7.1.20	4
139.538	$I4'/m'mm'$	d,E	$\bar{4}2m$	14.1.48	$m'mm$	8.3.26	mm2	7.1.20	4
139.539	$I4/m'm'm'$	d,B_2B_3	$\bar{4}'2m'$	14.4.51	$m'm'm'$	8.5.28	m'm'2	7.4.23	4
140.543	$I4/m'cm$	b,B_1B_2	$\bar{4}'2'm$	14.3.50	$m'mm$	8.3.26	mm2	7.1.20	4
140.543	$I4/m'cm$	c,${}^1E\,{}^2E$	$4/m'$	11.4.38	$42'2'$	12.4.43	4	9.1.29	4
140.544	$I4'/mc'm$	b,B_1B_2	$\bar{4}'2'm$	14.3.50	mmm	8.1.24	mm2	7.1.20	4
140.545	$I4'/mcm'$	b,B_2B_3	$\bar{4}'2m'$	14.4.51	$m'm'm$	8.4.27	m'm'2	7.4.23	4
140.546	$I4'/m'c'm$	b,E	$\bar{4}2m$	14.1.48	$m'mm$	8.3.26	mm2	7.1.20	4
140.549	$I4/m'c'm'$	b,B_2B_3	$\bar{4}'2m'$	14.4.51	$m'm'm'$	8.5.28	m'm'2	7.4.23	4
142.563	$I4_1/a'cd$	a,BB	$\bar{4}'$	10.3.34	$2'2'2$	6.3.19	2	3.1.6	8
142.564	$I4_1'/ac'd$	a,BB	$\bar{4}'$	10.3.34	222	6.1.17	2	3.1.6	8
142.565	$I4_1'/acd'$	a,BB	$\bar{4}'$	10.3.34	$2'2'2$	6.3.19	2	3.1.6	8
142.569	$I4_1/a'c'd'$	a,BB	$\bar{4}'$	10.3.34	222	6.1.17	2	3.1.6	8
163.81	$P\bar{3}'1c$	b,${}^1E\,{}^2E$	$\bar{3}'$	17.3.64	$32'1$	18.3.67	3	16.1.60	4
165.93	$P\bar{3}'c1$	b,${}^1E\,{}^2E$	$\bar{3}'$	17.3.64	$32'1$	18.3.67	3	16.1.60	4
167.105	$R\bar{3}'c$	b,${}^1E\,{}^2E$	$\bar{3}'$	17.3.64	$32'1$	18.3.67	3	16.1.60	4
176.145	$P6_3'/m$	b,${}^1E\,{}^2E$	$\bar{3}'$	17.3.64	$\bar{6}$	22.1.79	3	16.1.60	4
176.147	$P6_3'/m'$	a,${}^1E\,{}^2E$	$\bar{6}'$	22.3.81	$\bar{3}$	17.1.62	3	16.1.60	4
182.181	$P6_3'2'2$	b,E	32	18.1.65	$32'1$	18.3.67	3	16.1.60	4
182.182	$P6_3'22'$	a,E	32	18.1.65	$32'1$	18.3.67	3	16.1.60	4
188.218	$P\bar{6}'c2'$	b,${}^1E\,{}^2E$	$\bar{6}'$	22.3.81	$32'1$	18.3.67	3	16.1.60	4
188.218	$P\bar{6}'c2'$	d,${}^1E\,{}^2E$	$\bar{6}'$	22.3.81	$32'1$	18.3.67	3	16.1.60	4
188.218	$P\bar{6}'c2'$	f,${}^1E\,{}^2E$	$\bar{6}'$	22.3.81	$32'1$	18.3.67	3	16.1.60	4
190.229	$P\bar{6}'2'c$	b,${}^1E\,{}^2E$	$\bar{6}'$	22.3.81	$32'1$	18.3.67	3	16.1.60	4
192.245	$P6/m'cc$	b,${}^1E_1\,{}^2E_1$	$6/m'$	23.4.85	$62'2'$	24.4.90	6	21.1.76	4
192.245	$P6/m'cc$	b,${}^1E_2\,{}^2E_2$	$6/m'$	23.4.85	$62'2'$	24.4.90	6	21.1.76	4
192.245	$P6/m'cc$	d,${}^1E\,{}^2E$	$\bar{6}'$	22.3.81	$32'1$	18.3.67	3	16.1.60	8
192.247	$P6'/mcc'$	c,E	32	18.1.65	$\bar{6}$	22.1.79	3	16.1.60	8
192.249	$P6'/m'cc'$	d,${}^1E\,{}^2E$	$\bar{6}'$	22.3.81	$32'1$	18.3.67	3	16.1.60	8
193.255	$P6_3/m'cm$	c,${}^1E\,{}^2E$	$\bar{6}'$	22.3.81	$32'1$	18.3.67	3	16.1.60	8
193.257	$P6_3'/mcm'$	b,E	$\bar{3}'m'1$	20.4.74	$\bar{6}m'2'$	26.5.99	3m'1	19.3.70	4
193.257	$P6_3'/mcm'$	d,E	32	18.1.65	$\bar{6}$	22.1.79	3	16.1.60	8
193.259	$P6_3'/m'cm'$	a,E	$\bar{6}'m'2$	26.3.97	$\bar{3}m'1$	20.5.75	3m'1	19.3.70	4
193.259	$P6_3'/m'cm'$	c,${}^1E\,{}^2E$	$\bar{6}'$	22.3.81	$32'1$	18.3.67	3	16.1.60	8
194.266	$P6_3'/mm'c$	a,E	$\bar{3}'m'1$	20.4.74	$\bar{6}m'2'$	26.5.99	3m'1	19.3.70	4
194.268	$P6_3'/m'm'c$	b,E	$\bar{6}'m'2$	26.3.97	$\bar{3}m'1$	20.5.75	3m'1	19.3.70	4
201.20	$Pn'\bar{3}'$	b,${}^1E\,{}^2E$	$\bar{3}'$	17.3.64	23	28.1.107	3	16.1.60	8
201.20	$Pn'\bar{3}'$	c,${}^1E\,{}^2E$	$\bar{3}'$	17.3.64	23	28.1.107	3	16.1.60	8
203.28	$Fd'\bar{3}'$	c,${}^1E\,{}^2E$	$\bar{3}'$	17.3.64	23	28.1.107	3	16.1.60	8
203.28	$Fd'\bar{3}'$	d,${}^1E\,{}^2E$	$\bar{3}'$	17.3.64	23	28.1.107	3	16.1.60	8
204.32	$Im'\bar{3}'$	c,${}^1E\,{}^2E$	$\bar{3}'$	17.3.64	$m'\bar{3}'$	29.3.111	3	16.1.60	8
215.72	$P\bar{4}'3m'$	c,B_2B_3	$\bar{4}'2m'$	14.4.51	$\bar{4}'3m'$	31.3.117	m'm'2	7.4.23	6
215.72	$P\bar{4}'3m'$	d,B_2B_3	$\bar{4}'2m'$	14.4.51	$\bar{4}'3m'$	31.3.117	m'm'2	7.4.23	6
217.80	$I\bar{4}'3m'$	b,B_2B_3	$\bar{4}'2m'$	14.4.51	$\bar{4}'3m'$	31.3.117	m'm'2	7.4.23	6
217.80	$I\bar{4}'3m'$	d,BB	$\bar{4}'$	10.3.34	$\bar{4}'2m'$	14.4.51	2	3.1.6	12
218.83	$P\bar{4}'3n'$	c,BB	$\bar{4}'$	10.3.34	222	6.1.17	2	3.1.6	12
218.83	$P\bar{4}'3n'$	d,BB	$\bar{4}'$	10.3.34	222	6.1.17	2	3.1.6	12
219.87	$F\bar{4}'3c'$	c,BB	$\bar{4}'$	10.3.34	23	28.1.107	2	3.1.6	12
219.87	$F\bar{4}'3c'$	d,BB	$\bar{4}'$	10.3.34	23	28.1.107	2	3.1.6	12
221.96	$Pm'\bar{3}'m'$	c,E	$4/m'm'm'$	15.7.59	$m'\bar{3}'m'$	32.5.122	4m'm'	13.4.47	6
221.96	$Pm'\bar{3}'m'$	d,E	$4/m'm'm'$	15.7.59	$m'\bar{3}'m'$	32.5.122	4m'm'	13.4.47	6
222.100	$Pn'\bar{3}'n$	c,${}^1E\,{}^2E$	$\bar{3}'$	17.3.64	$4'32'$	30.3.114	3	16.1.60	16
222.101	$Pn\bar{3}n'$	d,BB	$\bar{4}'$	10.3.34	$4'22'$	12.3.42	2	3.1.6	24
222.102	$Pn'\bar{3}'n'$	b,E	422	12.1.40	432	30.1.112	4	9.1.29	12
222.102	$Pn'\bar{3}'n'$	c,${}^1E\,{}^2E$	$\bar{3}'$	17.3.64	432	30.1.112	3	16.1.60	16

222.102	$Pn'\bar{3}'n'$	d,BB	$\bar{4}'$	10.3.34	422	12.1.40	2	3.1.6	24
223.107	$Pm\bar{3}n'$	c,B_1B_2	$\bar{4}'2'm$	14.3.50	mmm	8.1.24	mm2	7.1.20	12
223.107	$Pm\bar{3}n'$	d,B_1B_2	$\bar{4}'2'm$	14.3.50	mmm	8.1.24	mm2	7.1.20	12
223.108	$Pm'\bar{3}'n'$	c,B_2B_3	$\bar{4}'2m'$	14.4.51	$m'm'm'$	8.5.28	m'm'2	7.4.23	12
223.108	$Pm'\bar{3}'n'$	d,B_2B_3	$\bar{4}'2m'$	14.4.51	$m'm'm'$	8.5.28	m'm'2	7.4.23	12
223.108	$Pm'\bar{3}'n'$	e,E	32	18.1.65	$m'\bar{3}'$	29.3.111	3	16.1.60	16
224.112	$Pn'\bar{3}'m$	d,E	$\bar{4}2m$	14.1.48	$\bar{4}3m$	31.1.115	mm2	7.1.20	12
224.113	$Pn\bar{3}m'$	d,B_2B_3	$\bar{4}'2m'$	14.4.51	$\bar{4}'3m'$	31.3.117	m'm'2	7.4.23	12
224.114	$Pn'\bar{3}'m'$	d,B_2B_3	$\bar{4}'2m'$	14.4.51	$\bar{4}'3m'$	31.3.117	m'm'2	7.4.23	12
224.114	$Pn'\bar{3}'m'$	b,E	$\bar{3}'m'1$	20.4.74	$\bar{4}'3m'$	31.3.117	3m'1	19.3.70	8
224.114	$Pn'\bar{3}'m'$	c,E	$\bar{3}'m'1$	20.4.74	$\bar{4}'3m'$	31.3.117	3m'1	19.3.70	8
226.125	$Fm\bar{3}c'$	c,B_1B_2	$\bar{4}'2'm$	14.3.50	$m\bar{3}$	29.1.109	mm2	7.1.20	12
226.126	$Fm'\bar{3}'c'$	c,B_2B_3	$\bar{4}'2m'$	14.4.51	$m'\bar{3}'$	29.3.111	m'm'2	7.4.23	12
226.126	$Fm'\bar{3}'c'$	d,${}^1E{}^2E$	$4/m'$	11.4.38	432	30.1.112	4	9.1.29	12
227.132	$Fd'\bar{3}'m'$	c,E	$\bar{3}'m'1$	20.4.74	$\bar{4}'3m'$	31.3.117	3m'1	19.3.70	8
227.132	$Fd'\bar{3}'m'$	d,E	$\bar{3}'m'1$	20.4.74	$\bar{4}'3m'$	31.3.117	3m'1	19.3.70	8
228.136	$Fd'\bar{3}'c$	c,${}^1E{}^2E$	$\bar{3}'$	17.3.64	23	28.1.107	3	16.1.60	16
			$\bar{3}'$	17.3.64	$32'1$	18.3.67	3	16.1.60	16
228.137	$Fd\bar{3}c'$	d,BB	$\bar{4}'$	10.3.34	23	28.1.107	2	3.1.6	24
228.138	$Fd'\bar{3}'c'$	d,BB	$\bar{4}'$	10.3.34	23	28.1.107	2	3.1.6	24
228.138	$Fd'\bar{3}'c'$	b,E	32	18.1.65	23	28.1.107	3	16.1.60	16
			32	18.1.65	$\bar{3}'$	17.3.64	3	16.1.60	16
228.138	$Fd'\bar{3}'c'$	c,${}^1E{}^2E$	$\bar{3}'$	17.3.64	23	28.1.107	3	16.1.60	16
			$\bar{3}'$	17.3.64	32	18.1.65	3	16.1.60	16
229.143	$Im\bar{3}m'$	d,B_1B_2	$\bar{4}'2'm$	14.3.50	$4'/mm'm$	15.4.56	mm2	7.1.20	12
229.144	$Im'\bar{3}'m'$	b,E	$4/m'm'm'$	15.7.59	$m'\bar{3}'m'$	32.5.122	4m'm'	13.4.47	6
229.144	$Im'\bar{3}'m'$	c,E	$\bar{3}'m'1$	20.4.74	$m'\bar{3}'m'$	32.5.122	3m'1	19.3.70	8
229.144	$Im'\bar{3}'m'$	d,B_2B_3	$\bar{4}'2m'$	14.4.51	$4/m'm'm'$	15.7.59	m'm'2	7.4.23	12
230.147	$Ia'\bar{3}'d$	a,${}^1E{}^2E$	$\bar{3}'$	17.3.64	$32'1$	18.3.67	3	16.1.60	16
230.148	$Ia\bar{3}d'$	d,BB	$\bar{4}'$	10.3.34	$2'2'2$	6.3.19	2	3.1.6	24
230.149	$Ia'\bar{3}'d'$	d,BB	$\bar{4}'$	10.3.34	222	6.1.17	2	3.1.6	24

Supplementary Table 19: Exceptional composite band coreps induced from site-symmetry coreps in the Type-III double MSGs (SN 5). In order, the columns in this table list the number of the MSG in the BNS setting and the symbol of the MSG, the letter of the maximal Wyckoff position containing $\mathbf{q}$ and the double-valued corep of the site-symmetry group $G_{\mathbf{q}}$, the symbol of the MSG isomorphic to the site-symmetry group $G_{\mathbf{q}}$ in the Hermann-Mauguin notation of the MPOINT tool on the BCS[15–18] and the number of the MSG isomorphic to $G_{\mathbf{q}}$ in the convention established by Litvin in SRef. 10, the symbol and number of the MPG isomorphic to the reducing group $G_{\mathbf{q}'}$, the symbol and number of the MPG isomorphic to the intersection group $G_{\mathbf{q}_0} = G_{\mathbf{q}} \cap G_{\mathbf{q}'}$, and the dimension d of the exceptional composite band corep. See SN 24 for further information regarding exceptional composite band coreps.

MSG		Corep	$G_{\mathbf{q}}$		$G_{\mathbf{q}'}$		$G_{\mathbf{q}_0}$		d
11.52	$P2_1'/m$	a,$\overline{A}\overline{A}$	$\bar{1}'$	2.3.5	m	4.1.9	1	1.1.1	4
11.52	$P2_1'/m$	b,$\overline{A}\overline{A}$	$\bar{1}'$	2.3.5	m	4.1.9	1	1.1.1	4
11.52	$P2_1'/m$	c,$\overline{A}\overline{A}$	$\bar{1}'$	2.3.5	m	4.1.9	1	1.1.1	4
11.52	$P2_1'/m$	d,$\overline{A}\overline{A}$	$\bar{1}'$	2.3.5	m	4.1.9	1	1.1.1	4
11.53	$P2_1/m'$	a,$\overline{A}\overline{A}$	$\bar{1}'$	2.3.5	m'	4.3.11	1	1.1.1	4
11.53	$P2_1/m'$	b,$\overline{A}\overline{A}$	$\bar{1}'$	2.3.5	m'	4.3.11	1	1.1.1	4
11.53	$P2_1/m'$	c,$\overline{A}\overline{A}$	$\bar{1}'$	2.3.5	m'	4.3.11	1	1.1.1	4
11.53	$P2_1/m'$	d,$\overline{A}\overline{A}$	$\bar{1}'$	2.3.5	m'	4.3.11	1	1.1.1	4
12.60	$C2'/m$	e,$\overline{A}\overline{A}$	$\bar{1}'$	2.3.5	$2'/m$	5.3.14	1	1.1.1	4
12.60	$C2'/m$	f,$\overline{A}\overline{A}$	$\bar{1}'$	2.3.5	$2'/m$	5.3.14	1	1.1.1	4
12.61	$C2/m'$	e,$\overline{A}\overline{A}$	$\bar{1}'$	2.3.5	$2/m'$	5.4.15	1	1.1.1	4
12.61	$C2/m'$	f,$\overline{A}\overline{A}$	$\bar{1}'$	2.3.5	$2/m'$	5.4.15	1	1.1.1	4
13.67	$P2'/c$	a,$\overline{A}\overline{A}$	$\bar{1}'$	2.3.5	$2'$	3.3.8	1	1.1.1	4
13.67	$P2'/c$	b,$\overline{A}\overline{A}$	$\bar{1}'$	2.3.5	$2'$	3.3.8	1	1.1.1	4
13.67	$P2'/c$	c,$\overline{A}\overline{A}$	$\bar{1}'$	2.3.5	$2'$	3.3.8	1	1.1.1	4
13.67	$P2'/c$	d,$\overline{A}\overline{A}$	$\bar{1}'$	2.3.5	$2'$	3.3.8	1	1.1.1	4
13.68	$P2/c'$	a,$\overline{A}\overline{A}$	$\bar{1}'$	2.3.5	2	3.1.6	1	1.1.1	4
13.68	$P2/c'$	b,$\overline{A}\overline{A}$	$\bar{1}'$	2.3.5	2	3.1.6	1	1.1.1	4
13.68	$P2/c'$	c,$\overline{A}\overline{A}$	$\bar{1}'$	2.3.5	2	3.1.6	1	1.1.1	4

13.68	$P2/c'$	d,$\overline{AA}$	$\bar{1}'$	2.3.5	2	3.1.6	1	1.1.1	4
15.87	$C2'/c$	a,$\overline{AA}$	$\bar{1}'$	2.3.5	$2'$	3.3.8	1	1.1.1	4
15.87	$C2'/c$	b,$\overline{AA}$	$\bar{1}'$	2.3.5	$2'$	3.3.8	1	1.1.1	4
15.87	$C2'/c$	c,$\overline{AA}$	$\bar{1}'$	2.3.5	$2'$	3.3.8	1	1.1.1	4
15.87	$C2'/c$	d,$\overline{AA}$	$\bar{1}'$	2.3.5	$2'$	3.3.8	1	1.1.1	4
15.88	$C2/c'$	a,$\overline{AA}$	$\bar{1}'$	2.3.5	2	3.1.6	1	1.1.1	4
15.88	$C2/c'$	b,$\overline{AA}$	$\bar{1}'$	2.3.5	2	3.1.6	1	1.1.1	4
15.88	$C2/c'$	c,$\overline{AA}$	$\bar{1}'$	2.3.5	2	3.1.6	1	1.1.1	4
15.88	$C2/c'$	d,$\overline{AA}$	$\bar{1}'$	2.3.5	2	3.1.6	1	1.1.1	4
48.259	$Pn'nn$	e,$\overline{AA}$	$\bar{1}'$	2.3.5	$2'2'2$	6.3.19	1	1.1.1	8
48.259	$Pn'nn$	f,$\overline{AA}$	$\bar{1}'$	2.3.5	$2'2'2$	6.3.19	1	1.1.1	8
48.261	$Pn'n'n'$	e,$\overline{AA}$	$\bar{1}'$	2.3.5	222	6.1.17	1	1.1.1	8
48.261	$Pn'n'n'$	f,$\overline{AA}$	$\bar{1}'$	2.3.5	222	6.1.17	1	1.1.1	8
49.267	$Pc'cm$	a,$^1\overline{E}\,^2\overline{E}$	$2'/m$	5.3.14	$2'2'2$	6.3.19	2'	3.3.8	4
49.267	$Pc'cm$	b,$^1\overline{E}\,^2\overline{E}$	$2'/m$	5.3.14	$2'2'2$	6.3.19	2'	3.3.8	4
49.267	$Pc'cm$	c,$^1\overline{E}\,^2\overline{E}$	$2'/m$	5.3.14	$2'2'2$	6.3.19	2'	3.3.8	4
49.267	$Pc'cm$	d,$^1\overline{E}\,^2\overline{E}$	$2'/m$	5.3.14	$2'2'2$	6.3.19	2'	3.3.8	4
49.268	$Pccm'$	a,$^1\overline{E}\,^2\overline{E}$	$2/m'$	5.4.15	$2'2'2$	6.3.19	2	3.1.6	4
49.268	$Pccm'$	b,$^1\overline{E}\,^2\overline{E}$	$2/m'$	5.4.15	$2'2'2$	6.3.19	2	3.1.6	4
49.268	$Pccm'$	c,$^1\overline{E}\,^2\overline{E}$	$2/m'$	5.4.15	$2'2'2$	6.3.19	2	3.1.6	4
49.268	$Pccm'$	d,$^1\overline{E}\,^2\overline{E}$	$2/m'$	5.4.15	$2'2'2$	6.3.19	2	3.1.6	4
50.279	$Pb'an$	e,$\overline{AA}$	$\bar{1}'$	2.3.5	$2'2'2$	6.3.19	1	1.1.1	8
50.279	$Pb'an$	f,$\overline{AA}$	$\bar{1}'$	2.3.5	$2'2'2$	6.3.19	1	1.1.1	8
50.280	$Pban'$	e,$\overline{AA}$	$\bar{1}'$	2.3.5	$2'2'2$	6.3.19	1	1.1.1	8
50.280	$Pban'$	f,$\overline{AA}$	$\bar{1}'$	2.3.5	$2'2'2$	6.3.19	1	1.1.1	8
50.283	$Pb'a'n'$	e,$\overline{AA}$	$\bar{1}'$	2.3.5	222	6.1.17	1	1.1.1	8
50.283	$Pb'a'n'$	f,$\overline{AA}$	$\bar{1}'$	2.3.5	222	6.1.17	1	1.1.1	8
51.291	$Pm'ma$	a,$^1\overline{E}\,^2\overline{E}$	$2'/m$	5.3.14	$m'm2'$	7.3.22	m	4.1.9	4
51.291	$Pm'ma$	b,$^1\overline{E}\,^2\overline{E}$	$2'/m$	5.3.14	$m'm2'$	7.3.22	m	4.1.9	4
51.291	$Pm'ma$	c,$^1\overline{E}\,^2\overline{E}$	$2'/m$	5.3.14	$m'm2'$	7.3.22	m	4.1.9	4
51.291	$Pm'ma$	d,$^1\overline{E}\,^2\overline{E}$	$2'/m$	5.3.14	$m'm2'$	7.3.22	m	4.1.9	4
51.292	$Pmm'a$	a,$^1\overline{E}\,^2\overline{E}$	$2/m'$	5.4.15	$m'm2'$	7.3.22	m'	4.3.11	4
51.292	$Pmm'a$	b,$^1\overline{E}\,^2\overline{E}$	$2/m'$	5.4.15	$m'm2'$	7.3.22	m'	4.3.11	4
51.292	$Pmm'a$	c,$^1\overline{E}\,^2\overline{E}$	$2/m'$	5.4.15	$m'm2'$	7.3.22	m'	4.3.11	4
51.292	$Pmm'a$	d,$^1\overline{E}\,^2\overline{E}$	$2/m'$	5.4.15	$m'm2'$	7.3.22	m'	4.3.11	4
51.297	$Pm'm'a'$	a,$^1\overline{E}\,^2\overline{E}$	$2/m'$	5.4.15	$m'm'2$	7.4.23	m'	4.3.11	4
51.297	$Pm'm'a'$	b,$^1\overline{E}\,^2\overline{E}$	$2/m'$	5.4.15	$m'm'2$	7.4.23	m'	4.3.11	4
51.297	$Pm'm'a'$	c,$^1\overline{E}\,^2\overline{E}$	$2/m'$	5.4.15	$m'm'2$	7.4.23	m'	4.3.11	4
51.297	$Pm'm'a'$	d,$^1\overline{E}\,^2\overline{E}$	$2/m'$	5.4.15	$m'm'2$	7.4.23	m'	4.3.11	4
52.307	$Pn'na$	a,$\overline{AA}$	$\bar{1}'$	2.3.5	$2'$	3.3.8	1	1.1.1	8
			$\bar{1}'$	2.3.5	2	3.1.6	1	1.1.1	8
52.307	$Pn'na$	b,$\overline{AA}$	$\bar{1}'$	2.3.5	$2'$	3.3.8	1	1.1.1	8
			$\bar{1}'$	2.3.5	2	3.1.6	1	1.1.1	8
52.308	$Pnn'a$	a,$\overline{AA}$	$\bar{1}'$	2.3.5	$2'$	3.3.8	1	1.1.1	8
52.308	$Pnn'a$	b,$\overline{AA}$	$\bar{1}'$	2.3.5	$2'$	3.3.8	1	1.1.1	8
52.309	$Pnna'$	a,$\overline{AA}$	$\bar{1}'$	2.3.5	2	3.1.6	1	1.1.1	8
			$\bar{1}'$	2.3.5	$2'$	3.3.8	1	1.1.1	8
52.309	$Pnna'$	b,$\overline{AA}$	$\bar{1}'$	2.3.5	2	3.1.6	1	1.1.1	8
			$\bar{1}'$	2.3.5	$2'$	3.3.8	1	1.1.1	8
52.313	$Pn'n'a'$	a,$\overline{AA}$	$\bar{1}'$	2.3.5	2	3.1.6	1	1.1.1	8
52.313	$Pn'n'a'$	b,$\overline{AA}$	$\bar{1}'$	2.3.5	2	3.1.6	1	1.1.1	8
54.339	$Pc'ca$	a,$\overline{AA}$	$\bar{1}'$	2.3.5	$2'$	3.3.8	1	1.1.1	8
54.339	$Pc'ca$	b,$\overline{AA}$	$\bar{1}'$	2.3.5	$2'$	3.3.8	1	1.1.1	8
54.340	$Pcc'a$	a,$\overline{AA}$	$\bar{1}'$	2.3.5	2	3.1.6	1	1.1.1	8
			$\bar{1}'$	2.3.5	$2'$	3.3.8	1	1.1.1	8
54.340	$Pcc'a$	b,$\overline{AA}$	$\bar{1}'$	2.3.5	2	3.1.6	1	1.1.1	8
			$\bar{1}'$	2.3.5	$2'$	3.3.8	1	1.1.1	8
54.341	$Pcca'$	a,$\overline{AA}$	$\bar{1}'$	2.3.5	$2'$	3.3.8	1	1.1.1	8
			$\bar{1}'$	2.3.5	2	3.1.6	1	1.1.1	8
54.341	$Pcca'$	b,$\overline{AA}$	$\bar{1}'$	2.3.5	$2'$	3.3.8	1	1.1.1	8
			$\bar{1}'$	2.3.5	2	3.1.6	1	1.1.1	8
54.345	$Pc'c'a'$	a,$\overline{AA}$	$\bar{1}'$	2.3.5	2	3.1.6	1	1.1.1	8
54.345	$Pc'c'a'$	b,$\overline{AA}$	$\bar{1}'$	2.3.5	2	3.1.6	1	1.1.1	8
56.367	$Pc'cn$	a,$\overline{AA}$	$\bar{1}'$	2.3.5	$2'$	3.3.8	1	1.1.1	8
56.367	$Pc'cn$	b,$\overline{AA}$	$\bar{1}'$	2.3.5	$2'$	3.3.8	1	1.1.1	8
56.368	$Pccn'$	a,$\overline{AA}$	$\bar{1}'$	2.3.5	2	3.1.6	1	1.1.1	8
56.368	$Pccn'$	b,$\overline{AA}$	$\bar{1}'$	2.3.5	2	3.1.6	1	1.1.1	8
56.371	$Pc'c'n'$	a,$\overline{AA}$	$\bar{1}'$	2.3.5	2	3.1.6	1	1.1.1	8
56.371	$Pc'c'n'$	b,$\overline{AA}$	$\bar{1}'$	2.3.5	2	3.1.6	1	1.1.1	8

57.379	$Pb'cm$	a,$\overline{AA}$	$\bar{1}'$	2.3.5	2	3.1.6	1	1.1.1	8
			$\bar{1}'$	2.3.5	m	4.1.9	1	1.1.1	8
57.379	$Pb'cm$	b,$\overline{AA}$	$\bar{1}'$	2.3.5	2	3.1.6	1	1.1.1	8
			$\bar{1}'$	2.3.5	m	4.1.9	1	1.1.1	8
57.380	$Pbc'm$	a,$\overline{AA}$	$\bar{1}'$	2.3.5	$2'$	3.3.8	1	1.1.1	8
			$\bar{1}'$	2.3.5	m	4.1.9	1	1.1.1	8
57.380	$Pbc'm$	b,$\overline{AA}$	$\bar{1}'$	2.3.5	$2'$	3.3.8	1	1.1.1	8
			$\bar{1}'$	2.3.5	m	4.1.9	1	1.1.1	8
57.381	$Pbcm'$	a,$\overline{AA}$	$\bar{1}'$	2.3.5	$2'$	3.3.8	1	1.1.1	8
			$\bar{1}'$	2.3.5	m'	4.3.11	1	1.1.1	8
57.381	$Pbcm'$	b,$\overline{AA}$	$\bar{1}'$	2.3.5	$2'$	3.3.8	1	1.1.1	8
			$\bar{1}'$	2.3.5	m'	4.3.11	1	1.1.1	8
57.385	$Pb'c'm'$	a,$\overline{AA}$	$\bar{1}'$	2.3.5	2	3.1.6	1	1.1.1	8
			$\bar{1}'$	2.3.5	m'	4.3.11	1	1.1.1	8
57.385	$Pb'c'm'$	b,$\overline{AA}$	$\bar{1}'$	2.3.5	2	3.1.6	1	1.1.1	8
			$\bar{1}'$	2.3.5	m'	4.3.11	1	1.1.1	8
59.407	$Pm'mn$	c,$\overline{AA}$	$\bar{1}'$	2.3.5	$m'm2'$	7.3.22	1	1.1.1	8
59.407	$Pm'mn$	d,$\overline{AA}$	$\bar{1}'$	2.3.5	$m'm2'$	7.3.22	1	1.1.1	8
59.408	$Pmmn'$	c,$\overline{AA}$	$\bar{1}'$	2.3.5	$mm2$	7.1.20	1	1.1.1	8
59.408	$Pmmn'$	d,$\overline{AA}$	$\bar{1}'$	2.3.5	$mm2$	7.1.20	1	1.1.1	8
59.411	$Pm'm'n'$	c,$\overline{AA}$	$\bar{1}'$	2.3.5	$m'm'2$	7.4.23	1	1.1.1	8
59.411	$Pm'm'n'$	d,$\overline{AA}$	$\bar{1}'$	2.3.5	$m'm'2$	7.4.23	1	1.1.1	8
60.419	$Pb'cn$	a,$\overline{AA}$	$\bar{1}'$	2.3.5	$2'$	3.3.8	1	1.1.1	8
60.419	$Pb'cn$	b,$\overline{AA}$	$\bar{1}'$	2.3.5	$2'$	3.3.8	1	1.1.1	8
60.420	$Pbc'n$	a,$\overline{AA}$	$\bar{1}'$	2.3.5	2	3.1.6	1	1.1.1	8
60.420	$Pbc'n$	b,$\overline{AA}$	$\bar{1}'$	2.3.5	2	3.1.6	1	1.1.1	8
60.421	$Pbcn'$	a,$\overline{AA}$	$\bar{1}'$	2.3.5	$2'$	3.3.8	1	1.1.1	8
60.421	$Pbcn'$	b,$\overline{AA}$	$\bar{1}'$	2.3.5	$2'$	3.3.8	1	1.1.1	8
60.425	$Pb'c'n'$	a,$\overline{AA}$	$\bar{1}'$	2.3.5	2	3.1.6	1	1.1.1	8
60.425	$Pb'c'n'$	b,$\overline{AA}$	$\bar{1}'$	2.3.5	2	3.1.6	1	1.1.1	8
62.443	$Pn'ma$	a,$\overline{AA}$	$\bar{1}'$	2.3.5	m	4.1.9	1	1.1.1	8
62.443	$Pn'ma$	b,$\overline{AA}$	$\bar{1}'$	2.3.5	m	4.1.9	1	1.1.1	8
62.444	$Pnm'a$	a,$\overline{AA}$	$\bar{1}'$	2.3.5	m'	4.3.11	1	1.1.1	8
62.444	$Pnm'a$	b,$\overline{AA}$	$\bar{1}'$	2.3.5	m'	4.3.11	1	1.1.1	8
62.445	$Pnma'$	a,$\overline{AA}$	$\bar{1}'$	2.3.5	m	4.1.9	1	1.1.1	8
62.445	$Pnma'$	b,$\overline{AA}$	$\bar{1}'$	2.3.5	m	4.1.9	1	1.1.1	8
62.449	$Pn'm'a'$	a,$\overline{AA}$	$\bar{1}'$	2.3.5	m'	4.3.11	1	1.1.1	8
62.449	$Pn'm'a'$	b,$\overline{AA}$	$\bar{1}'$	2.3.5	m'	4.3.11	1	1.1.1	8
63.459	$Cm'cm$	a, $^1\overline{E}\,^2\overline{E}$	$2/m'$	5.4.15	$m'm2'$	7.3.22	m'	4.3.11	4
63.459	$Cm'cm$	b, $^1\overline{E}\,^2\overline{E}$	$2/m'$	5.4.15	$m'm2'$	7.3.22	m'	4.3.11	4
63.459	$Cm'cm$	d,$\overline{AA}$	$\bar{1}'$	2.3.5	$2/m'$	5.4.15	1	1.1.1	8
			$\bar{1}'$	2.3.5	$m'm2'$	7.3.22	1	1.1.1	8
63.460	$Cmc'm$	d,$\overline{AA}$	$\bar{1}'$	2.3.5	$2'/m$	5.3.14	1	1.1.1	8
			$\bar{1}'$	2.3.5	$mm2$	7.1.20	1	1.1.1	8
63.461	$Cmcm'$	a, $^1\overline{E}\,^2\overline{E}$	$2'/m$	5.3.14	$m'm2'$	7.3.22	m	4.1.9	4
63.461	$Cmcm'$	b, $^1\overline{E}\,^2\overline{E}$	$2'/m$	5.3.14	$m'm2'$	7.3.22	m	4.1.9	4
63.461	$Cmcm'$	d,$\overline{AA}$	$\bar{1}'$	2.3.5	$2'/m$	5.3.14	1	1.1.1	8
			$\bar{1}'$	2.3.5	$m'm2'$	7.3.22	1	1.1.1	8
63.465	$Cm'c'm'$	a, $^1\overline{E}\,^2\overline{E}$	$2/m'$	5.4.15	$m'm'2$	7.4.23	m'	4.3.11	4
63.465	$Cm'c'm'$	b, $^1\overline{E}\,^2\overline{E}$	$2/m'$	5.4.15	$m'm'2$	7.4.23	m'	4.3.11	4
63.465	$Cm'c'm'$	d,$\overline{AA}$	$\bar{1}'$	2.3.5	$2/m'$	5.4.15	1	1.1.1	8
			$\bar{1}'$	2.3.5	$m'm'2$	7.4.23	1	1.1.1	8
64.471	$Cm'ca$	c,$\overline{AA}$	$\bar{1}'$	2.3.5	$2/m'$	5.4.15	1	1.1.1	8
			$\bar{1}'$	2.3.5	$2'$	3.3.8	1	1.1.1	8
64.472	$Cmc'a$	c,$\overline{AA}$	$\bar{1}'$	2.3.5	$2'/m$	5.3.14	1	1.1.1	8
			$\bar{1}'$	2.3.5	2	3.1.6	1	1.1.1	8
64.473	$Cmca'$	c,$\overline{AA}$	$\bar{1}'$	2.3.5	$2'/m$	5.3.14	1	1.1.1	8
			$\bar{1}'$	2.3.5	$2'$	3.3.8	1	1.1.1	8
64.477	$Cm'c'a'$	c,$\overline{AA}$	$\bar{1}'$	2.3.5	$2/m'$	5.4.15	1	1.1.1	8
			$\bar{1}'$	2.3.5	2	3.1.6	1	1.1.1	8
65.483	$Cm'mm$	e, $^1\overline{E}\,^2\overline{E}$	$2'/m$	5.3.14	$m'mm$	8.3.26	m	4.1.9	4
65.483	$Cm'mm$	f, $^1\overline{E}\,^2\overline{E}$	$2'/m$	5.3.14	$m'mm$	8.3.26	m	4.1.9	4
65.484	$Cmmm'$	e, $^1\overline{E}\,^2\overline{E}$	$2/m'$	5.4.15	$m'mm$	8.3.26	m'	4.3.11	4
65.484	$Cmmm'$	f, $^1\overline{E}\,^2\overline{E}$	$2/m'$	5.4.15	$m'mm$	8.3.26	m'	4.3.11	4
65.487	$Cm'm'm'$	e, $^1\overline{E}\,^2\overline{E}$	$2/m'$	5.4.15	$m'm'm'$	8.5.28	m'	4.3.11	4
65.487	$Cm'm'm'$	f, $^1\overline{E}\,^2\overline{E}$	$2/m'$	5.4.15	$m'm'm'$	8.5.28	m'	4.3.11	4
66.493	$Cc'cm$	c, $^1\overline{E}\,^2\overline{E}$	$2'/m$	5.3.14	$2'2'2$	6.3.19	2'	3.3.8	4
66.493	$Cc'cm$	d, $^1\overline{E}\,^2\overline{E}$	$2'/m$	5.3.14	$2'2'2$	6.3.19	2'	3.3.8	4
66.494	$Cccm'$	c, $^1\overline{E}\,^2\overline{E}$	$2/m'$	5.4.15	$2'2'2$	6.3.19	2	3.1.6	4
66.494	$Cccm'$	d, $^1\overline{E}\,^2\overline{E}$	$2/m'$	5.4.15	$2'2'2$	6.3.19	2	3.1.6	4
67.503	$Cm'ma$	c, $^1\overline{E}\,^2\overline{E}$	$2/m'$	5.4.15	$2'2'2$	6.3.19	2	3.1.6	4

			$2/m'$	5.4.15	$m'm2'$	7.3.22	m'	4.3.11	4
67.503	$Cm'ma$	d, $^1\overline{E}\,^2\overline{E}$	$2/m'$	5.4.15	$2'2'2$	6.3.19	2	3.1.6	4
			$2/m'$	5.4.15	$m'm2'$	7.3.22	m'	4.3.11	4
67.503	$Cm'ma$	e, $^1\overline{E}\,^2\overline{E}$	$2'/m$	5.3.14	$2'2'2$	6.3.19	2'	3.3.8	4
			$2'/m$	5.3.14	$m'm2'$	7.3.22	m	4.1.9	4
67.503	$Cm'ma$	f, $^1\overline{E}\,^2\overline{E}$	$2'/m$	5.3.14	$2'2'2$	6.3.19	2'	3.3.8	4
			$2'/m$	5.3.14	$m'm2'$	7.3.22	m	4.1.9	4
67.504	$Cmma'$	c, $^1\overline{E}\,^2\overline{E}$	$2'/m$	5.3.14	$2'2'2$	6.3.19	2'	3.3.8	4
			$2'/m$	5.3.14	$mm2$	7.1.20	m	4.1.9	4
67.504	$Cmma'$	d, $^1\overline{E}\,^2\overline{E}$	$2'/m$	5.3.14	$2'2'2$	6.3.19	2'	3.3.8	4
			$2'/m$	5.3.14	$mm2$	7.1.20	m	4.1.9	4
67.504	$Cmma'$	e, $^1\overline{E}\,^2\overline{E}$	$2'/m$	5.3.14	$2'2'2$	6.3.19	2'	3.3.8	4
			$2'/m$	5.3.14	$mm2$	7.1.20	m	4.1.9	4
67.504	$Cmma'$	f, $^1\overline{E}\,^2\overline{E}$	$2'/m$	5.3.14	$2'2'2$	6.3.19	2'	3.3.8	4
			$2'/m$	5.3.14	$mm2$	7.1.20	m	4.1.9	4
67.504	$Cmma'$	g,$\overline{E}$	$mm2$	7.1.20	$2'/m$	5.3.14	m	4.1.9	4
67.507	$Cm'm'a'$	c, $^1\overline{E}\,^2\overline{E}$	$2/m'$	5.4.15	222	6.1.17	2	3.1.6	4
			$2/m'$	5.4.15	$m'm'2$	7.4.23	m'	4.3.11	4
67.507	$Cm'm'a'$	d, $^1\overline{E}\,^2\overline{E}$	$2/m'$	5.4.15	222	6.1.17	2	3.1.6	4
			$2/m'$	5.4.15	$m'm'2$	7.4.23	m'	4.3.11	4
67.507	$Cm'm'a'$	e, $^1\overline{E}\,^2\overline{E}$	$2/m'$	5.4.15	222	6.1.17	2	3.1.6	4
			$2/m'$	5.4.15	$m'm'2$	7.4.23	m'	4.3.11	4
67.507	$Cm'm'a'$	f, $^1\overline{E}\,^2\overline{E}$	$2/m'$	5.4.15	222	6.1.17	2	3.1.6	4
			$2/m'$	5.4.15	$m'm'2$	7.4.23	m'	4.3.11	4
67.507	$Cm'm'a'$	a,$\overline{E}$	222	6.1.17	$2/m'$	5.4.15	2	3.1.6	4
67.507	$Cm'm'a'$	b,$\overline{E}$	222	6.1.17	$2/m'$	5.4.15	2	3.1.6	4
68.513	$Cc'ca$	c,$\overline{AA}$	$\bar{1}'$	2.3.5	$2'2'2$	6.3.19	1	1.1.1	8
			$\bar{1}'$	2.3.5	$2'$	3.3.8	1	1.1.1	8
68.513	$Cc'ca$	d,$\overline{AA}$	$\bar{1}'$	2.3.5	$2'2'2$	6.3.19	1	1.1.1	8
			$\bar{1}'$	2.3.5	$2'$	3.3.8	1	1.1.1	8
68.514	$Ccca'$	c,$\overline{AA}$	$\bar{1}'$	2.3.5	$2'2'2$	6.3.19	1	1.1.1	8
			$\bar{1}'$	2.3.5	2	3.1.6	1	1.1.1	8
68.514	$Ccca'$	d,$\overline{AA}$	$\bar{1}'$	2.3.5	$2'2'2$	6.3.19	1	1.1.1	8
			$\bar{1}'$	2.3.5	2	3.1.6	1	1.1.1	8
68.517	$Cc'c'a'$	c,$\overline{AA}$	$\bar{1}'$	2.3.5	222	6.1.17	1	1.1.1	8
			$\bar{1}'$	2.3.5	2	3.1.6	1	1.1.1	8
68.517	$Cc'c'a'$	d,$\overline{AA}$	$\bar{1}'$	2.3.5	222	6.1.17	1	1.1.1	8
			$\bar{1}'$	2.3.5	2	3.1.6	1	1.1.1	8
69.523	$Fm'mm$	c, $^1\overline{E}\,^2\overline{E}$	$2/m'$	5.4.15	$m'mm$	8.3.26	m'	4.3.11	4
			$2/m'$	5.4.15	$2'2'2$	6.3.19	2	3.1.6	4
69.523	$Fm'mm$	d, $^1\overline{E}\,^2\overline{E}$	$2'/m$	5.3.14	$m'mm$	8.3.26	m	4.1.9	4
			$2'/m$	5.3.14	$2'2'2$	6.3.19	2'	3.3.8	4
69.523	$Fm'mm$	e, $^1\overline{E}\,^2\overline{E}$	$2'/m$	5.3.14	$m'mm$	8.3.26	m	4.1.9	4
			$2'/m$	5.3.14	$2'2'2$	6.3.19	2'	3.3.8	4
69.525	$Fm'm'm'$	c, $^1\overline{E}\,^2\overline{E}$	$2/m'$	5.4.15	$m'm'm'$	8.5.28	m'	4.3.11	4
			$2/m'$	5.4.15	222	6.1.17	2	3.1.6	4
69.525	$Fm'm'm'$	d, $^1\overline{E}\,^2\overline{E}$	$2/m'$	5.4.15	$m'm'm'$	8.5.28	m'	4.3.11	4
			$2/m'$	5.4.15	222	6.1.17	2	3.1.6	4
69.525	$Fm'm'm'$	e, $^1\overline{E}\,^2\overline{E}$	$2/m'$	5.4.15	$m'm'm'$	8.5.28	m'	4.3.11	4
			$2/m'$	5.4.15	222	6.1.17	2	3.1.6	4
69.525	$Fm'm'm'$	f,$\overline{E}$	222	6.1.17	$2/m'$	5.4.15	2	3.1.6	4
70.529	$Fd'dd$	c,$\overline{AA}$	$\bar{1}'$	2.3.5	$2'2'2$	6.3.19	1	1.1.1	8
70.529	$Fd'dd$	d,$\overline{AA}$	$\bar{1}'$	2.3.5	$2'2'2$	6.3.19	1	1.1.1	8
70.531	$Fd'd'd'$	c,$\overline{AA}$	$\bar{1}'$	2.3.5	222	6.1.17	1	1.1.1	8
70.531	$Fd'd'd'$	d,$\overline{AA}$	$\bar{1}'$	2.3.5	222	6.1.17	1	1.1.1	8
71.535	$Im'mm$	k,$\overline{AA}$	$\bar{1}'$	2.3.5	$m'mm$	8.3.26	1	1.1.1	8
71.537	$Im'm'm'$	k,$\overline{AA}$	$\bar{1}'$	2.3.5	$m'm'm'$	8.5.28	1	1.1.1	8
72.541	$Ib'am$	c, $^1\overline{E}\,^2\overline{E}$	$2'/m$	5.3.14	$2'2'2$	6.3.19	2'	3.3.8	4
72.541	$Ib'am$	d, $^1\overline{E}\,^2\overline{E}$	$2'/m$	5.3.14	$2'2'2$	6.3.19	2'	3.3.8	4
72.541	$Ib'am$	e,$\overline{AA}$	$\bar{1}'$	2.3.5	$2'2'2$	6.3.19	1	1.1.1	8
			$\bar{1}'$	2.3.5	$2'/m$	5.3.14	1	1.1.1	8
72.542	$Ibam'$	c, $^1\overline{E}\,^2\overline{E}$	$2/m'$	5.4.15	$2'2'2$	6.3.19	2	3.1.6	4
72.542	$Ibam'$	d, $^1\overline{E}\,^2\overline{E}$	$2/m'$	5.4.15	$2'2'2$	6.3.19	2	3.1.6	4
72.542	$Ibam'$	e,$\overline{AA}$	$\bar{1}'$	2.3.5	$2'2'2$	6.3.19	1	1.1.1	8
			$\bar{1}'$	2.3.5	$2/m'$	5.4.15	1	1.1.1	8
72.545	$Ib'a'm'$	e,$\overline{AA}$	$\bar{1}'$	2.3.5	222	6.1.17	1	1.1.1	8
			$\bar{1}'$	2.3.5	$2/m'$	5.4.15	1	1.1.1	8
73.550	$Ib'ca$	a,$\overline{AA}$	$\bar{1}'$	2.3.5	2	3.1.6	1	1.1.1	8
			$\bar{1}'$	2.3.5	$2'$	3.3.8	1	1.1.1	8

73.550	$Ib'ca$	b,$\overline{A}\overline{A}$	$\bar{1}'$	2.3.5	2	3.1.6	1	1.1.1	8
			$\bar{1}'$	2.3.5	$2'$	3.3.8	1	1.1.1	8
73.552	$Ib'c'a'$	a,$\overline{A}\overline{A}$	$\bar{1}'$	2.3.5	2	3.1.6	1	1.1.1	8
73.552	$Ib'c'a'$	b,$\overline{A}\overline{A}$	$\bar{1}'$	2.3.5	2	3.1.6	1	1.1.1	8
74.556	$Im'ma$	a, ${}^1\overline{E}\,{}^2\overline{E}$	$2/m'$	5.4.15	$m'm2'$	7.3.22	m'	4.3.11	4
74.556	$Im'ma$	b, ${}^1\overline{E}\,{}^2\overline{E}$	$2/m'$	5.4.15	$m'm2'$	7.3.22	m'	4.3.11	4
74.556	$Im'ma$	c, ${}^1\overline{E}\,{}^2\overline{E}$	$2'/m$	5.3.14	$m'm2'$	7.3.22	m	4.1.9	4
74.556	$Im'ma$	d, ${}^1\overline{E}\,{}^2\overline{E}$	$2'/m$	5.3.14	$m'm2'$	7.3.22	m	4.1.9	4
74.560	$Im'm'a'$	a, ${}^1\overline{E}\,{}^2\overline{E}$	$2/m'$	5.4.15	$m'm'2$	7.4.23	m'	4.3.11	4
74.560	$Im'm'a'$	b, ${}^1\overline{E}\,{}^2\overline{E}$	$2/m'$	5.4.15	$m'm'2$	7.4.23	m'	4.3.11	4
74.560	$Im'm'a'$	c, ${}^1\overline{E}\,{}^2\overline{E}$	$2/m'$	5.4.15	$m'm'2$	7.4.23	m'	4.3.11	4
74.560	$Im'm'a'$	d, ${}^1\overline{E}\,{}^2\overline{E}$	$2/m'$	5.4.15	$m'm'2$	7.4.23	m'	4.3.11	4
83.46	$P4/m'$	e, ${}^1\overline{E}\,{}^2\overline{E}$	$2/m'$	5.4.15	$4/m'$	11.4.38	m'	4.3.11	4
83.46	$P4/m'$	f, ${}^1\overline{E}\,{}^2\overline{E}$	$2/m'$	5.4.15	$4/m'$	11.4.38	m'	4.3.11	4
83.47	$P4'/m'$	e, ${}^1\overline{E}\,{}^2\overline{E}$	$2/m'$	5.4.15	$4'/m'$	11.5.39	m'	4.3.11	4
83.47	$P4'/m'$	f, ${}^1\overline{E}\,{}^2\overline{E}$	$2/m'$	5.4.15	$4'/m'$	11.5.39	m'	4.3.11	4
84.53	$P4_2'/m$	e, ${}^1\overline{E}\,{}^2\overline{E}$	$\bar{4}'$	10.3.34	$2/m$	5.1.12	2	3.1.6	4
84.53	$P4_2'/m$	f, ${}^1\overline{E}\,{}^2\overline{E}$	$\bar{4}'$	10.3.34	$2/m$	5.1.12	2	3.1.6	4
84.55	$P4_2'/m'$	a, ${}^1\overline{E}\,{}^2\overline{E}$	$2/m'$	5.4.15	$\bar{4}$	10.1.32	2	3.1.6	4
84.55	$P4_2'/m'$	b, ${}^1\overline{E}\,{}^2\overline{E}$	$2/m'$	5.4.15	$\bar{4}$	10.1.32	2	3.1.6	4
85.62	$P4/n'$	d,$\overline{A}\overline{A}$	$\bar{1}'$	2.3.5	$\bar{4}'$	10.3.34	1	1.1.1	8
			$\bar{1}'$	2.3.5	4	9.1.29	1	1.1.1	8
85.62	$P4/n'$	e,$\overline{A}\overline{A}$	$\bar{1}'$	2.3.5	$\bar{4}'$	10.3.34	1	1.1.1	8
			$\bar{1}'$	2.3.5	4	9.1.29	1	1.1.1	8
85.63	$P4'/n'$	d,$\overline{A}\overline{A}$	$\bar{1}'$	2.3.5	$\bar{4}$	10.1.32	1	1.1.1	8
			$\bar{1}'$	2.3.5	$4'$	9.3.31	1	1.1.1	8
85.63	$P4'/n'$	e,$\overline{A}\overline{A}$	$\bar{1}'$	2.3.5	$\bar{4}$	10.1.32	1	1.1.1	8
			$\bar{1}'$	2.3.5	$4'$	9.3.31	1	1.1.1	8
86.70	$P4_2/n'$	c,$\overline{A}\overline{A}$	$\bar{1}'$	2.3.5	$\bar{4}'$	10.3.34	1	1.1.1	8
			$\bar{1}'$	2.3.5	2	3.1.6	1	1.1.1	8
86.70	$P4_2/n'$	d,$\overline{A}\overline{A}$	$\bar{1}'$	2.3.5	$\bar{4}'$	10.3.34	1	1.1.1	8
			$\bar{1}'$	2.3.5	2	3.1.6	1	1.1.1	8
86.71	$P4_2'/n'$	c,$\overline{A}\overline{A}$	$\bar{1}'$	2.3.5	$\bar{4}$	10.1.32	1	1.1.1	8
			$\bar{1}'$	2.3.5	2	3.1.6	1	1.1.1	8
86.71	$P4_2'/n'$	d,$\overline{A}\overline{A}$	$\bar{1}'$	2.3.5	$\bar{4}$	10.1.32	1	1.1.1	8
			$\bar{1}'$	2.3.5	2	3.1.6	1	1.1.1	8
87.77	$I4'/m$	d, ${}^1\overline{E}\,{}^2\overline{E}$	$\bar{4}'$	10.3.34	$2/m$	5.1.12	2	3.1.6	4
87.78	$I4/m'$	c, ${}^1\overline{E}\,{}^2\overline{E}$	$2/m'$	5.4.15	$4/m'$	11.4.38	m'	4.3.11	4
			$2/m'$	5.4.15	$\bar{4}'$	10.3.34	2	3.1.6	4
87.78	$I4/m'$	f,$\overline{A}\overline{A}$	$\bar{1}'$	2.3.5	$4/m'$	11.4.38	1	1.1.1	8
			$\bar{1}'$	2.3.5	$2/m'$	5.4.15	1	1.1.1	8
			$\bar{1}'$	2.3.5	$\bar{4}'$	10.3.34	1	1.1.1	8
87.78	$I4/m'$	d, ${}^1\overline{E}\,{}^2\overline{E}$	$\bar{4}'$	10.3.34	$2/m'$	5.4.15	2	3.1.6	4
87.79	$I4'/m'$	c, ${}^1\overline{E}\,{}^2\overline{E}$	$2/m'$	5.4.15	$4'/m'$	11.5.39	m'	4.3.11	4
			$2/m'$	5.4.15	$\bar{4}$	10.1.32	2	3.1.6	4
87.79	$I4'/m'$	f,$\overline{A}\overline{A}$	$\bar{1}'$	2.3.5	$4'/m'$	11.5.39	1	1.1.1	8
			$\bar{1}'$	2.3.5	$2/m'$	5.4.15	1	1.1.1	8
			$\bar{1}'$	2.3.5	$\bar{4}$	10.1.32	1	1.1.1	8
88.84	$I4_1/a'$	c,$\overline{A}\overline{A}$	$\bar{1}'$	2.3.5	$\bar{4}'$	10.3.34	1	1.1.1	8
88.84	$I4_1/a'$	d,$\overline{A}\overline{A}$	$\bar{1}'$	2.3.5	$\bar{4}'$	10.3.34	1	1.1.1	8
88.85	$I4_1'/a'$	c,$\overline{A}\overline{A}$	$\bar{1}'$	2.3.5	$\bar{4}$	10.1.32	1	1.1.1	8
88.85	$I4_1'/a'$	d,$\overline{A}\overline{A}$	$\bar{1}'$	2.3.5	$\bar{4}$	10.1.32	1	1.1.1	8
89.89	$P4'22'$	e,$\overline{E}$	222	6.1.17	$4'22'$	12.3.42	2	3.1.6	4
89.89	$P4'22'$	f,$\overline{E}$	222	6.1.17	$4'22'$	12.3.42	2	3.1.6	4
93.121	$P4_2'22'$	a,$\overline{E}$	222	6.1.17	$2'2'2$	6.3.19	2	3.1.6	4
93.121	$P4_2'22'$	b,$\overline{E}$	222	6.1.17	$2'2'2$	6.3.19	2	3.1.6	4
93.123	$P4_2'2'2$	e,$\overline{E}$	222	6.1.17	$2'2'2$	6.3.19	2	3.1.6	4
93.123	$P4_2'2'2$	f,$\overline{E}$	222	6.1.17	$2'2'2$	6.3.19	2	3.1.6	4
97.153	$I4'22'$	c,$\overline{E}$	222	6.1.17	$4'22'$	12.3.42	2	3.1.6	4
			222	6.1.17	$2'2'2$	6.3.19	2	3.1.6	4
97.155	$I4'2'2$	d,$\overline{E}$	222	6.1.17	$2'2'2$	6.3.19	2	3.1.6	4
99.166	$P4'mm'$	c,$\overline{E}$	$mm2$	7.1.20	$4'm'm$	13.3.46	m	4.1.9	4
107.230	$I4'mm'$	b,$\overline{E}$	$mm2$	7.1.20	$4'm'm$	13.3.46	m	4.1.9	4
111.254	$P\bar{4}'2m'$	e,$\overline{E}$	222	6.1.17	$\bar{4}'2m'$	14.4.51	2	3.1.6	4
111.254	$P\bar{4}'2m'$	f,$\overline{E}$	222	6.1.17	$\bar{4}'2m'$	14.4.51	2	3.1.6	4
112.261	$P\bar{4}'2'c$	e, ${}^1\overline{E}\,{}^2\overline{E}$	$\bar{4}'$	10.3.34	$2'2'2$	6.3.19	2	3.1.6	4
112.261	$P\bar{4}'2'c$	f, ${}^1\overline{E}\,{}^2\overline{E}$	$\bar{4}'$	10.3.34	$2'2'2$	6.3.19	2	3.1.6	4
115.286	$P\bar{4}'m2'$	g,$\overline{E}$	$mm2$	7.1.20	$\bar{4}'2'm$	14.3.50	m	4.1.9	4
116.294	$P\bar{4}'c2'$	c, ${}^1\overline{E}\,{}^2\overline{E}$	$\bar{4}'$	10.3.34	$2'2'2$	6.3.19	2	3.1.6	4
116.294	$P\bar{4}'c2'$	d, ${}^1\overline{E}\,{}^2\overline{E}$	$\bar{4}'$	10.3.34	$2'2'2$	6.3.19	2	3.1.6	4

120.324	$I\bar{4}'c2'$	b, ${}^1\overline{E}\,{}^2\overline{E}$	$\bar{4}'$	10.3.34	$2'2'2$	6.3.19	2	3.1.6	4
120.324	$I\bar{4}'c2'$	c, ${}^1\overline{E}\,{}^2\overline{E}$	$\bar{4}'$	10.3.34	$2'2'2$	6.3.19	2	3.1.6	4
121.329	$I\bar{4}'2'm$	d, ${}^1\overline{E}\,{}^2\overline{E}$	$\bar{4}'$	10.3.34	$2'2'2$	6.3.19	2	3.1.6	4
121.330	$I\bar{4}'2m'$	c,$\overline{E}$	222	6.1.17	$\bar{4}'2m'$	14.4.51	2	3.1.6	4
			222	6.1.17	$\bar{4}'$	10.3.34	2	3.1.6	4
121.330	$I\bar{4}'2m'$	d, ${}^1\overline{E}\,{}^2\overline{E}$	$\bar{4}'$	10.3.34	222	6.1.17	2	3.1.6	4
123.341	$P4/m'mm$	e,$\overline{E}$	$m'mm$	8.3.26	$4/m'mm$	15.3.55	m'm2'	7.3.22	4
123.341	$P4/m'mm$	f,$\overline{E}$	$m'mm$	8.3.26	$4/m'mm$	15.3.55	m'm2'	7.3.22	4
123.344	$P4'/m'm'm$	e,$\overline{E}$	$m'm'm'$	8.5.28	$4'/m'm'm$	15.5.57	m'm'2	7.4.23	4
123.344	$P4'/m'm'm$	f,$\overline{E}$	$m'm'm'$	8.5.28	$4'/m'm'm$	15.5.57	m'm'2	7.4.23	4
123.346	$P4'/m'mm'$	e,$\overline{E}$	$m'mm$	8.3.26	$4'/m'm'm$	15.5.57	m'm2'	7.3.22	4
123.346	$P4'/m'mm'$	f,$\overline{E}$	$m'mm$	8.3.26	$4'/m'm'm$	15.5.57	m'm2'	7.3.22	4
123.347	$P4/m'm'm'$	e,$\overline{E}$	$m'm'm'$	8.5.28	$4/m'm'm'$	15.7.59	m'm'2	7.4.23	4
123.347	$P4/m'm'm'$	f,$\overline{E}$	$m'm'm'$	8.5.28	$4/m'm'm'$	15.7.59	m'm'2	7.4.23	4
124.353	$P4/m'cc$	b, ${}^1\overline{E}_2\,{}^2\overline{E}_2$	$4/m'$	11.4.38	$42'2'$	12.4.43	4	9.1.29	4
124.353	$P4/m'cc$	b, ${}^1\overline{E}_1\,{}^2\overline{E}_1$	$4/m'$	11.4.38	$42'2'$	12.4.43	4	9.1.29	4
124.353	$P4/m'cc$	d, ${}^1\overline{E}_2\,{}^2\overline{E}_2$	$4/m'$	11.4.38	$42'2'$	12.4.43	4	9.1.29	4
124.353	$P4/m'cc$	d, ${}^1\overline{E}_1\,{}^2\overline{E}_1$	$4/m'$	11.4.38	$42'2'$	12.4.43	4	9.1.29	4
124.353	$P4/m'cc$	e, ${}^1\overline{E}\,{}^2\overline{E}$	$2/m'$	5.4.15	$4/m'$	11.4.38	m'	4.3.11	8
			$2/m'$	5.4.15	$2'2'2$	6.3.19	2	3.1.6	8
124.355	$P4'/mcc'$	f,$\overline{E}$	222	6.1.17	$4'22'$	12.3.42	2	3.1.6	8
			222	6.1.17	$2/m$	5.1.12	2	3.1.6	8
124.356	$P4'/m'c'c$	e, ${}^1\overline{E}\,{}^2\overline{E}$	$2/m'$	5.4.15	$4'/m'$	11.5.39	m'	4.3.11	8
			$2/m'$	5.4.15	222	6.1.17	2	3.1.6	8
124.356	$P4'/m'c'c$	f,$\overline{E}$	222	6.1.17	$4'22'$	12.3.42	2	3.1.6	8
			222	6.1.17	$2/m'$	5.4.15	2	3.1.6	8
124.358	$P4'/m'cc'$	e, ${}^1\overline{E}\,{}^2\overline{E}$	$2/m'$	5.4.15	$4'/m'$	11.5.39	m'	4.3.11	8
			$2/m'$	5.4.15	$2'2'2$	6.3.19	2	3.1.6	8
124.359	$P4/m'c'c'$	e, ${}^1\overline{E}\,{}^2\overline{E}$	$2/m'$	5.4.15	$4/m'$	11.4.38	m'	4.3.11	8
			$2/m'$	5.4.15	222	6.1.17	2	3.1.6	8
124.359	$P4/m'c'c'$	f,$\overline{E}$	222	6.1.17	422	12.1.40	2	3.1.6	8
			222	6.1.17	$2/m'$	5.4.15	2	3.1.6	8
125.365	$P4/n'bm$	e, ${}^1\overline{E}\,{}^2\overline{E}$	$2'/m$	5.3.14	$42'2'$	12.4.43	2'	3.3.8	8
			$2'/m$	5.3.14	$\bar{4}'2'm$	14.3.50	m	4.1.9	8
125.365	$P4/n'bm$	f, ${}^1\overline{E}\,{}^2\overline{E}$	$2'/m$	5.3.14	$42'2'$	12.4.43	2'	3.3.8	8
			$2'/m$	5.3.14	$\bar{4}'2'm$	14.3.50	m	4.1.9	8
125.368	$P4'/n'b'm$	e, ${}^1\overline{E}\,{}^2\overline{E}$	$2'/m$	5.3.14	$4'22'$	12.3.42	2'	3.3.8	8
			$2'/m$	5.3.14	$\bar{4}2m$	14.1.48	m	4.1.9	8
125.368	$P4'/n'b'm$	f, ${}^1\overline{E}\,{}^2\overline{E}$	$2'/m$	5.3.14	$4'22'$	12.3.42	2'	3.3.8	8
			$2'/m$	5.3.14	$\bar{4}2m$	14.1.48	m	4.1.9	8
125.370	$P4'/n'bm'$	e, ${}^1\overline{E}\,{}^2\overline{E}$	$2/m'$	5.4.15	$4'22'$	12.3.42	2	3.1.6	8
			$2/m'$	5.4.15	$\bar{4}2'm'$	14.5.52	m'	4.3.11	8
125.370	$P4'/n'bm'$	f, ${}^1\overline{E}\,{}^2\overline{E}$	$2/m'$	5.4.15	$4'22'$	12.3.42	2	3.1.6	8
			$2/m'$	5.4.15	$\bar{4}2'm'$	14.5.52	m'	4.3.11	8
125.371	$P4/n'b'm'$	e, ${}^1\overline{E}\,{}^2\overline{E}$	$2/m'$	5.4.15	422	12.1.40	2	3.1.6	8
			$2/m'$	5.4.15	$\bar{4}'2m'$	14.4.51	m'	4.3.11	8
125.371	$P4/n'b'm'$	f, ${}^1\overline{E}\,{}^2\overline{E}$	$2/m'$	5.4.15	422	12.1.40	2	3.1.6	8
			$2/m'$	5.4.15	$\bar{4}'2m'$	14.4.51	m'	4.3.11	8
126.377	$P4/n'nc$	d, ${}^1\overline{E}\,{}^2\overline{E}$	$\bar{4}'$	10.3.34	$2'2'2$	6.3.19	2	3.1.6	8
126.377	$P4/n'nc$	f,$\overline{A}\overline{A}$	$\bar{1}'$	2.3.5	$42'2'$	12.4.43	1	1.1.1	16
			$\bar{1}'$	2.3.5	$2'2'2$	6.3.19	1	1.1.1	16
			$\bar{1}'$	2.3.5	$\bar{4}'$	10.3.34	1	1.1.1	16
126.378	$P4'/nn'c$	d, ${}^1\overline{E}\,{}^2\overline{E}$	$\bar{4}'$	10.3.34	$2'2'2$	6.3.19	2	3.1.6	8
126.379	$P4'/nnc'$	c,$\overline{E}$	222	6.1.17	$4'22'$	12.3.42	2	3.1.6	8
			222	6.1.17	$\bar{4}'$	10.3.34	2	3.1.6	8
126.379	$P4'/nnc'$	d, ${}^1\overline{E}\,{}^2\overline{E}$	$\bar{4}'$	10.3.34	222	6.1.17	2	3.1.6	8
126.380	$P4'/n'n'c$	c,$\overline{E}$	222	6.1.17	$4'22'$	12.3.42	2	3.1.6	8
			222	6.1.17	$\bar{4}$	10.1.32	2	3.1.6	8
126.380	$P4'/n'n'c$	f,$\overline{A}\overline{A}$	$\bar{1}'$	2.3.5	$4'22'$	12.3.42	1	1.1.1	16
			$\bar{1}'$	2.3.5	222	6.1.17	1	1.1.1	16
			$\bar{1}'$	2.3.5	$\bar{4}$	10.1.32	1	1.1.1	16
126.382	$P4'/n'nc'$	f,$\overline{A}\overline{A}$	$\bar{1}'$	2.3.5	$4'22'$	12.3.42	1	1.1.1	16
			$\bar{1}'$	2.3.5	$2'2'2$	6.3.19	1	1.1.1	16
			$\bar{1}'$	2.3.5	$\bar{4}$	10.1.32	1	1.1.1	16
126.383	$P4/n'n'c'$	c,$\overline{E}$	222	6.1.17	422	12.1.40	2	3.1.6	8
			222	6.1.17	$\bar{4}'$	10.3.34	2	3.1.6	8
126.383	$P4/n'n'c'$	f,$\overline{A}\overline{A}$	$\bar{1}'$	2.3.5	422	12.1.40	1	1.1.1	16
			$\bar{1}'$	2.3.5	222	6.1.17	1	1.1.1	16
			$\bar{1}'$	2.3.5	$\bar{4}'$	10.3.34	1	1.1.1	16
126.383	$P4/n'n'c'$	d, ${}^1\overline{E}\,{}^2\overline{E}$	$\bar{4}'$	10.3.34	222	6.1.17	2	3.1.6	8

128.401	$P4/m'nc$	c,${}^1\overline{E}\,{}^2\overline{E}$	$2/m'$	5.4.15	$4/m'$	11.4.38	m'	4.3.11	8
			$2/m'$	5.4.15	$2'2'2$	6.3.19	2	3.1.6	8
128.402	$P4'/mn'c$	d,$\overline{E}$	222	6.1.17	$2/m$	5.1.12	2	3.1.6	8
128.404	$P4'/m'n'c$	c,${}^1\overline{E}\,{}^2\overline{E}$	$2/m'$	5.4.15	$4'/m'$	11.5.39	m'	4.3.11	8
			$2/m'$	5.4.15	$2'2'2$	6.3.19	2	3.1.6	8
128.406	$P4'/m'nc'$	c,${}^1\overline{E}\,{}^2\overline{E}$	$2/m'$	5.4.15	$4'/m'$	11.5.39	m'	4.3.11	8
			$2/m'$	5.4.15	222	6.1.17	2	3.1.6	8
128.406	$P4'/m'nc'$	d,$\overline{E}$	222	6.1.17	$2/m'$	5.4.15	2	3.1.6	8
128.407	$P4/m'n'c'$	c,${}^1\overline{E}\,{}^2\overline{E}$	$2/m'$	5.4.15	$4/m'$	11.4.38	m'	4.3.11	8
			$2/m'$	5.4.15	222	6.1.17	2	3.1.6	8
128.407	$P4/m'n'c'$	d,$\overline{E}$	222	6.1.17	$2/m'$	5.4.15	2	3.1.6	8
129.413	$P4/n'mm$	d,${}^1\overline{E}\,{}^2\overline{E}$	$2'/m$	5.3.14	$\bar{4}'2'm$	14.3.50	2'	3.3.8	8
			$2'/m$	5.3.14	$4mm$	13.1.44	m	4.1.9	8
129.413	$P4/n'mm$	e,${}^1\overline{E}\,{}^2\overline{E}$	$2'/m$	5.3.14	$\bar{4}'2'm$	14.3.50	2'	3.3.8	8
			$2'/m$	5.3.14	$4mm$	13.1.44	m	4.1.9	8
129.416	$P4'/n'm'm$	d,${}^1\overline{E}\,{}^2\overline{E}$	$2'/m$	5.3.14	$\bar{4}2'm'$	14.5.52	2'	3.3.8	8
			$2'/m$	5.3.14	$4'm'm$	13.3.46	m	4.1.9	8
129.416	$P4'/n'm'm$	e,${}^1\overline{E}\,{}^2\overline{E}$	$2'/m$	5.3.14	$\bar{4}2'm'$	14.5.52	2'	3.3.8	8
			$2'/m$	5.3.14	$4'm'm$	13.3.46	m	4.1.9	8
129.418	$P4'/n'mm'$	d,${}^1\overline{E}\,{}^2\overline{E}$	$2/m'$	5.4.15	$\bar{4}2m$	14.1.48	2	3.1.6	8
			$2/m'$	5.4.15	$4'm'm$	13.3.46	m'	4.3.11	8
129.418	$P4'/n'mm'$	e,${}^1\overline{E}\,{}^2\overline{E}$	$2/m'$	5.4.15	$\bar{4}2m$	14.1.48	2	3.1.6	8
			$2/m'$	5.4.15	$4'm'm$	13.3.46	m'	4.3.11	8
129.419	$P4/n'm'm'$	d,${}^1\overline{E}\,{}^2\overline{E}$	$2/m'$	5.4.15	$\bar{4}'2m'$	14.4.51	2	3.1.6	8
			$2/m'$	5.4.15	$4m'm'$	13.4.47	m'	4.3.11	8
129.419	$P4/n'm'm'$	e,${}^1\overline{E}\,{}^2\overline{E}$	$2/m'$	5.4.15	$\bar{4}'2m'$	14.4.51	2	3.1.6	8
			$2/m'$	5.4.15	$4m'm'$	13.4.47	m'	4.3.11	8
130.425	$P4/n'cc$	b,${}^1\overline{E}\,{}^2\overline{E}$	$\bar{4}'$	10.3.34	$2'2'2$	6.3.19	2	3.1.6	8
130.425	$P4/n'cc$	d,$\overline{A}\overline{A}$	$\bar{1}'$	2.3.5	$2'2'2$	6.3.19	1	1.1.1	16
			$\bar{1}'$	2.3.5	$\bar{4}'$	10.3.34	1	1.1.1	16
			$\bar{1}'$	2.3.5	4	9.1.29	1	1.1.1	16
130.427	$P4'/ncc'$	b,${}^1\overline{E}\,{}^2\overline{E}$	$\bar{4}'$	10.3.34	$2'2'2$	6.3.19	2	3.1.6	8
130.428	$P4'/n'c'c$	d,$\overline{A}\overline{A}$	$\bar{1}'$	2.3.5	$2'2'2$	6.3.19	1	1.1.1	16
			$\bar{1}'$	2.3.5	$\bar{4}$	10.1.32	1	1.1.1	16
			$\bar{1}'$	2.3.5	$4'$	9.3.31	1	1.1.1	16
130.430	$P4'/n'cc'$	a,$\overline{E}$	222	6.1.17	$\bar{4}$	10.1.32	2	3.1.6	8
130.430	$P4'/n'cc'$	d,$\overline{A}\overline{A}$	$\bar{1}'$	2.3.5	222	6.1.17	1	1.1.1	16
			$\bar{1}'$	2.3.5	$\bar{4}$	10.1.32	1	1.1.1	16
			$\bar{1}'$	2.3.5	$4'$	9.3.31	1	1.1.1	16
130.431	$P4/n'c'c'$	d,$\overline{A}\overline{A}$	$\bar{1}'$	2.3.5	222	6.1.17	1	1.1.1	16
			$\bar{1}'$	2.3.5	$\bar{4}'$	10.3.34	1	1.1.1	16
			$\bar{1}'$	2.3.5	4	9.1.29	1	1.1.1	16
131.438	$P4_2'/mm'c$	e,$\overline{E}$	$\bar{4}'2m'$	14.4.51	$m'm'm$	8.4.27	m'm'2	7.4.23	4
131.438	$P4_2'/mm'c$	f,$\overline{E}$	$\bar{4}'2m'$	14.4.51	$m'm'm$	8.4.27	m'm'2	7.4.23	4
131.440	$P4_2'/m'm'c$	a,$\overline{E}$	$m'm'm'$	8.5.28	$\bar{4}2'm'$	14.5.52	m'm'2	7.4.23	4
131.440	$P4_2'/m'm'c$	b,$\overline{E}$	$m'm'm'$	8.5.28	$\bar{4}2'm'$	14.5.52	m'm'2	7.4.23	4
132.449	$P4_2/m'cm$	f,${}^1\overline{E}\,{}^2\overline{E}$	$2/m'$	5.4.15	$m'mm$	8.3.26	m'	4.3.11	8
			$2/m'$	5.4.15	$2'2'2$	6.3.19	2	3.1.6	8
132.451	$P4_2'/mcm'$	b,$\overline{E}$	$\bar{4}'2m'$	14.4.51	$m'm'm$	8.4.27	m'm'2	7.4.23	4
132.451	$P4_2'/mcm'$	d,$\overline{E}$	$\bar{4}'2m'$	14.4.51	$m'm'm$	8.4.27	m'm'2	7.4.23	4
132.451	$P4_2'/mcm'$	e,$\overline{E}$	222	6.1.17	$\bar{4}'2m'$	14.4.51	2	3.1.6	8
			222	6.1.17	$2/m$	5.1.12	2	3.1.6	8
132.452	$P4_2'/m'c'm$	e,$\overline{E}$	222	6.1.17	$\bar{4}2m$	14.1.48	2	3.1.6	8
			222	6.1.17	$2/m'$	5.4.15	2	3.1.6	8
132.452	$P4_2'/m'c'm$	f,${}^1\overline{E}\,{}^2\overline{E}$	$2/m'$	5.4.15	$m'mm$	8.3.26	m'	4.3.11	8
			$2/m'$	5.4.15	222	6.1.17	2	3.1.6	8
132.454	$P4_2'/m'cm'$	a,$\overline{E}$	$m'm'm'$	8.5.28	$\bar{4}2'm'$	14.5.52	m'm'2	7.4.23	4
132.454	$P4_2'/m'cm'$	c,$\overline{E}$	$m'm'm'$	8.5.28	$\bar{4}2'm'$	14.5.52	m'm'2	7.4.23	4
132.454	$P4_2'/m'cm'$	f,${}^1\overline{E}\,{}^2\overline{E}$	$2/m'$	5.4.15	$m'm'm'$	8.5.28	m'	4.3.11	8
			$2/m'$	5.4.15	$2'2'2$	6.3.19	2	3.1.6	8
132.455	$P4_2/m'c'm'$	e,$\overline{E}$	222	6.1.17	$\bar{4}'2m'$	14.4.51	2	3.1.6	8
			222	6.1.17	$2/m'$	5.4.15	2	3.1.6	8
132.455	$P4_2/m'c'm'$	f,${}^1\overline{E}\,{}^2\overline{E}$	$2/m'$	5.4.15	$m'm'm'$	8.5.28	m'	4.3.11	8
			$2/m'$	5.4.15	222	6.1.17	2	3.1.6	8
133.461	$P4_2/n'bc$	d,${}^1\overline{E}\,{}^2\overline{E}$	$\bar{4}'$	10.3.34	$2'2'2$	6.3.19	2	3.1.6	8
133.461	$P4_2/n'bc$	e,$\overline{A}\overline{A}$	$\bar{1}'$	2.3.5	$2'2'2$	6.3.19	1	1.1.1	16
			$\bar{1}'$	2.3.5	$\bar{4}'$	10.3.34	1	1.1.1	16
133.462	$P4_2'/nb'c$	c,$\overline{E}$	222	6.1.17	$2'2'2$	6.3.19	2	3.1.6	8
133.462	$P4_2'/nb'c$	d,${}^1\overline{E}\,{}^2\overline{E}$	$\bar{4}'$	10.3.34	$2'2'2$	6.3.19	2	3.1.6	8

133.463	$P4_2'/nbc'$	a,$\overline{E}$	222	6.1.17	$2'2'2$	6.3.19	2	3.1.6	8
133.463	$P4_2'/nbc'$	b,$\overline{E}$	222	6.1.17	$\bar{4}'$	10.3.34	2	3.1.6	8
133.463	$P4_2'/nbc'$	d, $^1\overline{E}\,^2\overline{E}$	$\bar{4}'$	10.3.34	222	6.1.17	2	3.1.6	8
133.464	$P4_2'/n'b'c$	a,$\overline{E}$	222	6.1.17	$2'2'2$	6.3.19	2	3.1.6	8
133.464	$P4_2'/n'b'c$	b,$\overline{E}$	222	6.1.17	$\bar{4}$	10.1.32	2	3.1.6	8
133.464	$P4_2'/n'b'c$	e,$\overline{AA}$	$\bar{1}'$	2.3.5	222	6.1.17	1	1.1.1	16
			$\bar{1}'$	2.3.5	$2'2'2$	6.3.19	1	1.1.1	16
			$1'$	2.3.5	4	10.1.32	1	1.1.1	16
133.466	$P4_2'/n'bc'$	c,$\overline{E}$	222	6.1.17	$2'2'2$	6.3.19	2	3.1.6	8
133.466	$P4_2'/n'bc'$	e,$\overline{AA}$	$\bar{1}'$	2.3.5	$2'2'2$	6.3.19	1	1.1.1	16
			$\bar{1}'$	2.3.5	222	6.1.17	1	1.1.1	16
			$\bar{1}'$	2.3.5	4	10.1.32	1	1.1.1	16
133.467	$P4_2/n'b'c'$	e,$\overline{AA}$	$\bar{1}'$	2.3.5	222	6.1.17	1	1.1.1	16
			$\bar{1}'$	2.3.5	$\bar{4}'$	10.3.34	1	1.1.1	16
134.473	$P4_2/n'nm$	e, $^1\overline{E}\,^2\overline{E}$	$2'/m$	5.3.14	$\bar{4}'2'm$	14.3.50	m	4.1.9	8
			$2'/m$	5.3.14	$2'2'2$	6.3.19	2'	3.3.8	8
134.473	$P4_2/n'nm$	f, $^1\overline{E}\,^2\overline{E}$	$2'/m$	5.3.14	$\bar{4}'2'm$	14.3.50	m	4.1.9	8
			$2'/m$	5.3.14	$2'2'2$	6.3.19	2'	3.3.8	8
134.474	$P4_2'/nn'm$	d,$\overline{E}$	222	6.1.17	$2'2'2$	6.3.19	2	3.1.6	8
			222	6.1.17	$2/m$	5.1.12	2	3.1.6	8
134.475	$P4_2'/nnm'$	c,$\overline{E}$	222	6.1.17	$\bar{4}'2m'$	14.4.51	2	3.1.6	8
			222	6.1.17	$2'2'2$	6.3.19	2	3.1.6	8
134.476	$P4_2'/n'n'm$	c,$\overline{E}$	222	6.1.17	$\bar{4}2m$	14.1.48	2	3.1.6	8
			222	6.1.17	$2'2'2$	6.3.19	2	3.1.6	8
134.476	$P4_2'/n'n'm$	e, $^1\overline{E}\,^2\overline{E}$	$2'/m$	5.3.14	$\bar{4}2m$	14.1.48	m	4.1.9	8
			$2'/m$	5.3.14	$2'2'2$	6.3.19	2'	3.3.8	8
134.476	$P4_2'/n'n'm$	f, $^1\overline{E}\,^2\overline{E}$	$2'/m$	5.3.14	$\bar{4}2m$	14.1.48	m	4.1.9	8
			$2'/m$	5.3.14	$2'2'2$	6.3.19	2'	3.3.8	8
134.478	$P4_2'/n'nm'$	d,$\overline{E}$	222	6.1.17	$2'2'2$	6.3.19	2	3.1.6	8
			222	6.1.17	$2/m'$	5.4.15	2	3.1.6	8
134.478	$P4_2'/n'nm'$	e, $^1\overline{E}\,^2\overline{E}$	$2/m'$	5.4.15	$\bar{4}2'm'$	14.5.52	m'	4.3.11	8
			$2/m'$	5.4.15	222	6.1.17	2	3.1.6	8
134.478	$P4_2'/n'nm'$	f, $^1\overline{E}\,^2\overline{E}$	$2/m'$	5.4.15	$\bar{4}2'm'$	14.5.52	m'	4.3.11	8
			$2/m'$	5.4.15	222	6.1.17	2	3.1.6	8
134.479	$P4_2/n'n'm'$	c,$\overline{E}$	222	6.1.17	$\bar{4}'2m'$	14.4.51	2	3.1.6	8
134.479	$P4_2/n'n'm'$	e, $^1\overline{E}\,^2\overline{E}$	$2/m'$	5.4.15	$\bar{4}'2m'$	14.4.51	m'	4.3.11	8
			$2/m'$	5.4.15	222	6.1.17	2	3.1.6	8
134.479	$P4_2/n'n'm'$	f, $^1\overline{E}\,^2\overline{E}$	$2/m'$	5.4.15	$\bar{4}'2m'$	14.4.51	m'	4.3.11	8
			$2/m'$	5.4.15	222	6.1.17	2	3.1.6	8
134.479	$P4_2/n'n'm'$	d,$\overline{E}$	222	6.1.17	$2/m'$	5.4.15	2	3.1.6	8
135.485	$P4_2/m'bc$	c, $^1\overline{E}\,^2\overline{E}$	$2/m'$	5.4.15	$2'2'2$	6.3.19	2	3.1.6	8
135.485	$P4_2/m'bc$	a, $^1\overline{E}\,^2\overline{E}$	$2/m'$	5.4.15	$\bar{4}'$	10.3.34	2	3.1.6	8
135.485	$P4_2/m'bc$	b, $^1\overline{E}\,^2\overline{E}$	$\bar{4}'$	10.3.34	$2/m'$	5.4.15	2	3.1.6	8
135.486	$P4_2'/mb'c$	b, $^1\overline{E}\,^2\overline{E}$	$\bar{4}'$	10.3.34	$2/m$	5.1.12	2	3.1.6	8
135.486	$P4_2'/mb'c$	d,$\overline{E}$	222	6.1.17	$2/m$	5.1.12	2	3.1.6	8
135.487	$P4_2'/mbc'$	b, $^1\overline{E}\,^2\overline{E}$	$\bar{4}'$	10.3.34	$2/m$	5.1.12	2	3.1.6	8
135.488	$P4_2'/m'b'c$	a, $^1\overline{E}\,^2\overline{E}$	$2/m'$	5.4.15	$\bar{4}$	10.1.32	2	3.1.6	8
135.488	$P4_2'/m'b'c$	c, $^1\overline{E}\,^2\overline{E}$	$2/m'$	5.4.15	$2'2'2$	6.3.19	2	3.1.6	8
135.490	$P4_2'/m'bc'$	a, $^1\overline{E}\,^2\overline{E}$	$2/m'$	5.4.15	$\bar{4}$	10.1.32	2	3.1.6	8
135.490	$P4_2'/m'bc'$	c, $^1\overline{E}\,^2\overline{E}$	$2/m'$	5.4.15	222	6.1.17	2	3.1.6	8
135.490	$P4_2'/m'bc'$	d,$\overline{E}$	222	6.1.17	$2/m'$	5.4.15	2	3.1.6	8
136.497	$P4_2/m'nm$	c, $^1\overline{E}\,^2\overline{E}$	$2/m'$	5.4.15	$m'mm$	8.3.26	m'	4.3.11	8
			$2/m'$	5.4.15	$\bar{4}'$	10.3.34	2	3.1.6	8
136.497	$P4_2/m'nm$	d, $^1\overline{E}\,^2\overline{E}$	$\bar{4}'$	10.3.34	$2/m'$	5.4.15	2	3.1.6	8
136.498	$P4_2'/mn'm$	d, $^1\overline{E}\,^2\overline{E}$	$\bar{4}'$	10.3.34	$2/m$	5.1.12	2	3.1.6	8
136.499	$P4_2'/mnm'$	d, $^1\overline{E}\,^2\overline{E}$	$\bar{4}'$	10.3.34	$2/m$	5.1.12	2	3.1.6	8
136.500	$P4_2'/m'n'm$	c, $^1\overline{E}\,^2\overline{E}$	$2/m'$	5.4.15	$m'mm$	8.3.26	m'	4.3.11	8
			$2/m'$	5.4.15	$\bar{4}$	10.1.32	2	3.1.6	8
136.502	$P4_2'/m'nm'$	c, $^1\overline{E}\,^2\overline{E}$	$2/m'$	5.4.15	$m'm'm'$	8.5.28	m'	4.3.11	8
			$2/m'$	5.4.15	$\bar{4}$	10.1.32	2	3.1.6	8
136.503	$P4_2/m'n'm'$	c, $^1\overline{E}\,^2\overline{E}$	$2/m'$	5.4.15	$m'm'm'$	8.5.28	m'	4.3.11	8
			$2/m'$	5.4.15	$\bar{4}'$	10.3.34	2	3.1.6	8
136.503	$P4_2/m'n'm'$	d, $^1\overline{E}\,^2\overline{E}$	$\bar{4}'$	10.3.34	$2/m'$	5.4.15	2	3.1.6	8
137.509	$P4_2/n'mc$	d,$\overline{E}$	$mm2$	7.1.20	$\bar{4}'2'm$	14.3.50	m	4.1.9	8
137.509	$P4_2/n'mc$	e,$\overline{AA}$	$\bar{1}'$	2.3.5	$\bar{4}'2'm$	14.3.50	1	1.1.1	16
			$\bar{1}'$	2.3.5	$mm2$	7.1.20	1	1.1.1	16
137.511	$P4_2'/nmc'$	d,$\overline{E}$	$mm2$	7.1.20	$\bar{4}'2'm$	14.3.50	m	4.1.9	8
137.512	$P4_2'/n'm'c$	e,$\overline{AA}$	$\bar{1}'$	2.3.5	$\bar{4}2'm'$	14.5.52	1	1.1.1	16
			$\bar{1}'$	2.3.5	$m'm'2$	7.4.23	1	1.1.1	16

137.514	$P4_2'/n'mc'$	d,$\overline{E}$	$mm2$	7.1.20	$\bar{4}2m$	14.1.48	m	4.1.9	8
137.514	$P4_2'/n'mc'$	e,$\overline{AA}$	$\bar{1}'$	2.3.5	$\bar{4}2m$	14.1.48	1	1.1.1	16
			$\bar{1}'$	2.3.5	$mm2$	7.1.20	1	1.1.1	16
137.515	$P4_2/n'm'c'$	e,$\overline{AA}$	$\bar{1}'$	2.3.5	$\bar{4}'2m'$	14.4.51	1	1.1.1	16
			$\bar{1}'$	2.3.5	$m'm'2$	7.4.23	1	1.1.1	16
138.521	$P4_2/n'cm$	b,$^1\overline{E}\,^2\overline{E}$	$\bar{4}'$	10.3.34	$2'2'2$	6.3.19	2	3.1.6	8
138.521	$P4_2/n'cm$	c,$^1\overline{E}\,^2\overline{E}$	$2'/m$	5.3.14	$2'2'2$	6.3.19	2'	3.3.8	8
			$2'/m$	5.3.14	$mm2$	7.1.20	m	4.1.9	8
138.521	$P4_2/n'cm$	d,$^1\overline{E}\,^2\overline{E}$	$2'/m$	5.3.14	$2'2'2$	6.3.19	2'	3.3.8	8
			$2'/m$	5.3.14	$mm2$	7.1.20	m	4.1.9	8
138.521	$P4_2/n'cm$	e,$\overline{E}$	$mm2$	7.1.20	$2'/m$	5.3.14	m	4.1.9	8
138.522	$P4_2'/nc'm$	a,$\overline{E}$	222	6.1.17	$\bar{4}'$	10.3.34	2	3.1.6	8
			222	6.1.17	$2/m$	5.1.12	2	3.1.6	8
138.522	$P4_2'/nc'm$	e,$\overline{E}$	$mm2$	7.1.20	$2/m$	5.1.12	m	4.1.9	8
138.522	$P4_2'/nc'm$	b,$^1\overline{E}\,^2\overline{E}$	$\bar{4}'$	10.3.34	222	6.1.17	2	3.1.6	8
138.523	$P4_2'/ncm'$	b,$^1\overline{E}\,^2\overline{E}$	$\bar{4}'$	10.3.34	$2'2'2$	6.3.19	2	3.1.6	8
138.524	$P4_2'/n'c'm$	c,$^1\overline{E}\,^2\overline{E}$	$2'/m$	5.3.14	$2'2'2$	6.3.19	2'	3.3.8	8
			$2'/m$	5.3.14	$mm2$	7.1.20	m	4.1.9	8
138.524	$P4_2'/n'c'm$	d,$^1\overline{E}\,^2\overline{E}$	$2'/m$	5.3.14	$2'2'2$	6.3.19	2'	3.3.8	8
			$2'/m$	5.3.14	$mm2$	7.1.20	m	4.1.9	8
138.524	$P4_2'/n'c'm$	e,$\overline{E}$	$mm2$	7.1.20	$2'/m$	5.3.14	m	4.1.9	8
138.526	$P4_2'/n'cm'$	a,$\overline{E}$	222	6.1.17	$\bar{4}$	10.1.32	2	3.1.6	8
			222	6.1.17	$2/m'$	5.4.15	2	3.1.6	8
138.526	$P4_2'/n'cm'$	c,$^1\overline{E}\,^2\overline{E}$	$2/m'$	5.4.15	222	6.1.17	2	3.1.6	8
			$2/m'$	5.4.15	$m'm'2$	7.4.23	m'	4.3.11	8
138.526	$P4_2'/n'cm'$	d,$^1\overline{E}\,^2\overline{E}$	$2/m'$	5.4.15	222	6.1.17	2	3.1.6	8
			$2/m'$	5.4.15	$m'm'2$	7.4.23	m'	4.3.11	8
138.527	$P4_2/n'c'm'$	c,$^1\overline{E}\,^2\overline{E}$	$2/m'$	5.4.15	222	6.1.17	2	3.1.6	8
			$2/m'$	5.4.15	$m'm'2$	7.4.23	m'	4.3.11	8
138.527	$P4_2/n'c'm'$	d,$^1\overline{E}\,^2\overline{E}$	$2/m'$	5.4.15	222	6.1.17	2	3.1.6	8
			$2/m'$	5.4.15	$m'm'2$	7.4.23	m'	4.3.11	8
138.527	$P4_2/n'c'm'$	a,$\overline{E}$	222	6.1.17	$\bar{4}'$	10.3.34	2	3.1.6	8
			222	6.1.17	$2/m'$	5.4.15	2	3.1.6	8
138.527	$P4_2/n'c'm'$	b,$^1\overline{E}\,^2\overline{E}$	$\bar{4}'$	10.3.34	222	6.1.17	2	3.1.6	8
139.533	$I4/m'mm$	c,$\overline{E}$	$m'mm$	8.3.26	$4/m'mm$	15.3.55	m'm2'	7.3.22	4
139.533	$I4/m'mm$	f,$^1\overline{E}\,^2\overline{E}$	$2'/m$	5.3.14	$4/m'mm$	15.3.55	m	4.1.9	8
			$2'/m$	5.3.14	$\bar{4}'2'm$	14.3.50	2'	3.3.8	8
139.534	$I4'/mm'm$	d,$\overline{E}$	$\bar{4}'2m'$	14.4.51	$m'm'm$	8.4.27	m'm'2	7.4.23	4
139.536	$I4'/m'm'm$	c,$\overline{E}$	$m'm'm'$	8.5.28	$4'/m'm'm$	15.5.57	m'm'2	7.4.23	4
			$m'm'm'$	8.5.28	$\bar{4}2'm'$	14.5.52	m'm'2	7.4.23	4
139.536	$I4'/m'm'm$	f,$^1\overline{E}\,^2\overline{E}$	$2'/m$	5.3.14	$4'/m'm'm$	15.5.57	m	4.1.9	8
			$2'/m$	5.3.14	$\bar{4}2'm'$	14.5.52	2'	3.3.8	8
139.538	$I4'/m'mm'$	c,$\overline{E}$	$m'mm$	8.3.26	$4'/m'm'm$	15.5.57	m'm2'	7.3.22	4
139.538	$I4'/m'mm'$	f,$^1\overline{E}\,^2\overline{E}$	$2/m'$	5.4.15	$4'/m'm'm$	15.5.57	m'	4.3.11	8
			$2/m'$	5.4.15	$\bar{4}2m$	14.1.48	2	3.1.6	8
139.539	$I4/m'm'm'$	c,$\overline{E}$	$m'm'm'$	8.5.28	$4/m'm'm'$	15.7.59	m'm'2	7.4.23	4
			$m'm'm'$	8.5.28	$\bar{4}'2m'$	14.4.51	m'm'2	7.4.23	4
139.539	$I4/m'm'm'$	f,$^1\overline{E}\,^2\overline{E}$	$2/m'$	5.4.15	$4/m'm'm'$	15.7.59	m'	4.3.11	8
			$2/m'$	5.4.15	$\bar{4}'2m'$	14.4.51	2	3.1.6	8
139.539	$I4/m'm'm'$	d,$\overline{E}$	$\bar{4}'2m'$	14.4.51	$m'm'm'$	8.5.28	m'm'2	7.4.23	4
140.543	$I4/m'cm$	c,$^1\overline{E}_2\,^2\overline{E}_2$	$4/m'$	11.4.38	$42'2'$	12.4.43	4	9.1.29	4
140.543	$I4/m'cm$	c,$^1\overline{E}_1\,^2\overline{E}_1$	$4/m'$	11.4.38	$42'2'$	12.4.43	4	9.1.29	4
140.543	$I4/m'cm$	e,$^1\overline{E}\,^2\overline{E}$	$2'/m$	5.3.14	$42'2'$	12.4.43	2'	3.3.8	8
			$2'/m$	5.3.14	$\bar{4}'2'm$	14.3.50	m	4.1.9	8
			$2'/m$	5.3.14	$m'mm$	8.3.26	m	4.1.9	8
140.545	$I4'/mcm'$	b,$\overline{E}$	$\bar{4}'2m'$	14.4.51	$m'm'm$	8.4.27	m'm'2	7.4.23	4
140.546	$I4'/m'c'm$	e,$^1\overline{E}\,^2\overline{E}$	$2'/m$	5.3.14	$4'22'$	12.3.42	2'	3.3.8	8
			$2'/m$	5.3.14	$\bar{4}2m$	14.1.48	m	4.1.9	8
			$2'/m$	5.3.14	$m'mm$	8.3.26	m	4.1.9	8
140.548	$I4'/m'cm'$	d,$\overline{E}$	$m'm'm'$	8.5.28	$\bar{4}2'm'$	14.5.52	m'm'2	7.4.23	4
140.548	$I4'/m'cm'$	e,$^1\overline{E}\,^2\overline{E}$	$2/m'$	5.4.15	$4'22'$	12.3.42	2	3.1.6	8
			$2/m'$	5.4.15	$\bar{4}2'm'$	14.5.52	m'	4.3.11	8
			$2/m'$	5.4.15	$m'm'm'$	8.5.28	m'	4.3.11	8
140.549	$I4/m'c'm'$	e,$^1\overline{E}\,^2\overline{E}$	$2/m'$	5.4.15	422	12.1.40	2	3.1.6	8
			$2/m'$	5.4.15	$\bar{4}'2m'$	14.4.51	m'	4.3.11	8
			$2/m'$	5.4.15	$m'm'm'$	8.5.28	m'	4.3.11	8
141.553	$I4_1/a'md$	c,$^1\overline{E}\,^2\overline{E}$	$2'/m$	5.3.14	$\bar{4}'2'm$	14.3.50	m	4.1.9	8
141.553	$I4_1/a'md$	d,$^1\overline{E}\,^2\overline{E}$	$2'/m$	5.3.14	$\bar{4}'2'm$	14.3.50	m	4.1.9	8

141.556	$I4_1'/a'm'd$	c, $^1\overline{E}\,^2\overline{E}$	$2/m'$	5.4.15	$\bar{4}2'm'$	14.5.52	m'	4.3.11	8
141.556	$I4_1'/a'm'd$	d, $^1\overline{E}\,^2\overline{E}$	$2/m'$	5.4.15	$\bar{4}2'm'$	14.5.52	m'	4.3.11	8
141.558	$I4_1'/a'md'$	c, $^1\overline{E}\,^2\overline{E}$	$2'/m$	5.3.14	$\bar{4}2m$	14.1.48	m	4.1.9	8
141.558	$I4_1'/a'md'$	d, $^1\overline{E}\,^2\overline{E}$	$2'/m$	5.3.14	$\bar{4}2m$	14.1.48	m	4.1.9	8
141.559	$I4_1/a'm'd'$	c, $^1\overline{E}\,^2\overline{E}$	$2/m'$	5.4.15	$\bar{4}'2m'$	14.4.51	m'	4.3.11	8
141.559	$I4_1/a'm'd'$	d, $^1\overline{E}\,^2\overline{E}$	$2/m'$	5.4.15	$\bar{4}'2m'$	14.4.51	m'	4.3.11	8
142.563	$I4_1/a'cd$	a, $^1\overline{E}\,^2\overline{E}$	$\bar{4}'$	10.3.34	$2'2'2$	6.3.19	2	3.1.6	8
142.563	$I4_1/a'cd$	c,$\overline{A}\overline{A}$	$\bar{1}'$	2.3.5	$\bar{4}'$	10.3.34	1	1.1.1	16
			$\bar{1}'$	2.3.5	$2'2'2$	6.3.19	1	1.1.1	16
			$\bar{1}'$	2.3.5	$2'$	3.3.8	1	1.1.1	16
142.565	$I4_1'/acd'$	a, $^1\overline{E}\,^2\overline{E}$	$\bar{4}'$	10.3.34	$2'2'2$	6.3.19	2	3.1.6	8
142.566	$I4_1'/a'c'd$	c,$\overline{A}\overline{A}$	$\bar{1}'$	2.3.5	$\bar{4}$	10.1.32	1	1.1.1	16
			$\bar{1}'$	2.3.5	$2'2'2$	6.3.19	1	1.1.1	16
			$\bar{1}'$	2.3.5	2	3.1.6	1	1.1.1	16
142.568	$I4_1'/a'cd'$	b,$\overline{E}$	222	6.1.17	$\bar{4}$	10.1.32	2	3.1.6	8
142.568	$I4_1'/a'cd'$	c,$\overline{A}\overline{A}$	$\bar{1}'$	2.3.5	$\bar{4}$	10.1.32	1	1.1.1	16
			$\bar{1}'$	2.3.5	222	6.1.17	1	1.1.1	16
			$\bar{1}'$	2.3.5	$2'$	3.3.8	1	1.1.1	16
142.569	$I4_1/a'c'd'$	c,$\overline{A}\overline{A}$	$\bar{1}'$	2.3.5	$\bar{4}'$	10.3.34	1	1.1.1	16
			$\bar{1}'$	2.3.5	222	6.1.17	1	1.1.1	16
			$\bar{1}'$	2.3.5	2	3.1.6	1	1.1.1	16
147.15	$P\bar{3}'$	e,$\overline{A}\overline{A}$	$\bar{1}'$	2.3.5	$\bar{3}'$	17.3.64	1	1.1.1	6
			$\bar{1}'$	2.3.5	3	16.1.60	1	1.1.1	6
147.15	$P\bar{3}'$	f,$\overline{A}\overline{A}$	$\bar{1}'$	2.3.5	$\bar{3}'$	17.3.64	1	1.1.1	6
			$\bar{1}'$	2.3.5	3	16.1.60	1	1.1.1	6
148.19	$R\bar{3}'$	d,$\overline{A}\overline{A}$	$\bar{1}'$	2.3.5	$\bar{3}'$	17.3.64	1	1.1.1	6
148.19	$R\bar{3}'$	e,$\overline{A}\overline{A}$	$\bar{1}'$	2.3.5	$\bar{3}'$	17.3.64	1	1.1.1	6
162.75	$P\bar{3}'1m$	f, $^1\overline{E}\,^2\overline{E}$	$2'/m$	5.3.14	$\bar{3}'1m$	20.3.73	2'	3.3.8	6
			$2'/m$	5.3.14	$\bar{3}'1m$	20.3.73	m	4.1.9	6
			$2'/m$	5.3.14	$32'$	18.3.67	2'	3.3.8	6
162.75	$P\bar{3}'1m$	g, $^1\overline{E}\,^2\overline{E}$	$2'/m$	5.3.14	$\bar{3}'1m$	20.3.73	m	4.1.9	6
			$2'/m$	5.3.14	$\bar{3}'1m$	20.3.73	2'	3.3.8	6
			$2'/m$	5.3.14	$32'$	18.3.67	2'	3.3.8	6
162.76	$P\bar{3}'1m'$	f, $^1\overline{E}\,^2\overline{E}$	$2/m'$	5.4.15	$\bar{3}'1m'$	20.4.74	2	3.1.6	6
			$2/m'$	5.4.15	$\bar{3}'1m'$	20.4.74	m'	4.3.11	6
			$2/m'$	5.4.15	32	18.1.65	2	3.1.6	6
162.76	$P\bar{3}'1m'$	g, $^1\overline{E}\,^2\overline{E}$	$2/m'$	5.4.15	$\bar{3}'1m'$	20.4.74	m'	4.3.11	6
			$2/m'$	5.4.15	$\bar{3}'1m'$	20.4.74	2	3.1.6	6
			$2/m'$	5.4.15	32	18.1.65	2	3.1.6	6
163.81	$P\bar{3}'1c$	b,$\overline{E}\overline{E}$	$\bar{3}'$	17.3.64	$32'$	18.3.67	3	16.1.60	4
163.81	$P\bar{3}'1c$	b, $^1\overline{E}\,^2\overline{E}$	$\bar{3}'$	17.3.64	$32'$	18.3.67	3	16.1.60	4
163.81	$P\bar{3}'1c$	g,$\overline{A}\overline{A}$	$\bar{1}'$	2.3.5	$32'$	18.3.67	1	1.1.1	12
			$\bar{1}'$	2.3.5	$\bar{3}'$	17.3.64	1	1.1.1	12
163.82	$P\bar{3}'1c'$	b,$\overline{E}\overline{E}$	$\bar{3}'$	17.3.64	32	18.1.65	3	16.1.60	4
163.82	$P\bar{3}'1c'$	g,$\overline{A}\overline{A}$	$\bar{1}'$	2.3.5	32	18.1.65	1	1.1.1	12
			$\bar{1}'$	2.3.5	$\bar{3}'$	17.3.64	1	1.1.1	12
164.87	$P\bar{3}'m1$	e, $^1\overline{E}\,^2\overline{E}$	$2'/m$	5.3.14	$\bar{3}'1m$	20.3.73	2'	3.3.8	6
			$2'/m$	5.3.14	$\bar{3}'1m$	20.3.73	m	4.1.9	6
			$2'/m$	5.3.14	$3m$	19.1.68	m	4.1.9	6
164.87	$P\bar{3}'m1$	f, $^1\overline{E}\,^2\overline{E}$	$2'/m$	5.3.14	$\bar{3}'1m$	20.3.73	m	4.1.9	6
			$2'/m$	5.3.14	$\bar{3}'1m$	20.3.73	2'	3.3.8	6
			$2'/m$	5.3.14	$3m$	19.1.68	m	4.1.9	6
164.88	$P\bar{3}'m'1$	e, $^1\overline{E}\,^2\overline{E}$	$2/m'$	5.4.15	$\bar{3}'1m'$	20.4.74	2	3.1.6	6
			$2/m'$	5.4.15	$\bar{3}'1m'$	20.4.74	m'	4.3.11	6
			$2/m'$	5.4.15	$3m'$	19.3.70	m'	4.3.11	6
164.88	$P\bar{3}'m'1$	f, $^1\overline{E}\,^2\overline{E}$	$2/m'$	5.4.15	$\bar{3}'1m'$	20.4.74	m'	4.3.11	6
			$2/m'$	5.4.15	$\bar{3}'1m'$	20.4.74	2	3.1.6	6
			$2/m'$	5.4.15	$3m'$	19.3.70	m'	4.3.11	6
165.93	$P\bar{3}'c1$	b,$\overline{E}\overline{E}$	$\bar{3}'$	17.3.64	$32'$	18.3.67	3	16.1.60	4
165.93	$P\bar{3}'c1$	b, $^1\overline{E}\,^2\overline{E}$	$\bar{3}'$	17.3.64	$32'$	18.3.67	3	16.1.60	4
165.93	$P\bar{3}'c1$	e,$\overline{A}\overline{A}$	$\bar{1}'$	2.3.5	$32'$	18.3.67	1	1.1.1	12
			$\bar{1}'$	2.3.5	$\bar{3}'$	17.3.64	1	1.1.1	12
			$\bar{1}'$	2.3.5	3	16.1.60	1	1.1.1	12
165.94	$P\bar{3}'c'1$	b,$\overline{E}\overline{E}$	$\bar{3}'$	17.3.64	32	18.1.65	3	16.1.60	4
165.94	$P\bar{3}'c'1$	e,$\overline{A}\overline{A}$	$\bar{1}'$	2.3.5	32	18.1.65	1	1.1.1	12
			$\bar{1}'$	2.3.5	$\bar{3}'$	17.3.64	1	1.1.1	12
			$\bar{1}'$	2.3.5	3	16.1.60	1	1.1.1	12
166.99	$R\bar{3}'m$	d, $^1\overline{E}\,^2\overline{E}$	$2'/m$	5.3.14	$\bar{3}'1m$	20.3.73	m	4.1.9	6
			$2'/m$	5.3.14	$\bar{3}'1m$	20.3.73	2'	3.3.8	6
166.99	$R\bar{3}'m$	e, $^1\overline{E}\,^2\overline{E}$	$2'/m$	5.3.14	$\bar{3}'1m$	20.3.73	2'	3.3.8	6
			$2'/m$	5.3.14	$\bar{3}'1m$	20.3.73	m	4.1.9	6
166.100	$R\bar{3}'m'$	d, $^1\overline{E}\,^2\overline{E}$	$2/m'$	5.4.15	$\bar{3}'1m'$	20.4.74	m'	4.3.11	6

			$2/m'$	5.4.15	$\bar{3}'1m'$	20.4.74	2	3.1.6	6
166.100	$R\bar{3}'m'$	e, $^1\overline{E}\,^2\overline{E}$	$2/m'$	5.4.15	$\bar{3}'1m'$	20.4.74	2	3.1.6	6
			$2/m'$	5.4.15	$\bar{3}'1m'$	20.4.74	m'	4.3.11	6
167.105	$R\bar{3}'c$	b, $\overline{E}\overline{E}$	$\bar{3}'$	17.3.64	$32'$	18.3.67	3	16.1.60	4
167.105	$R\bar{3}'c$	b, $^1\overline{E}\,^2\overline{E}$	$\bar{3}'$	17.3.64	$32'$	18.3.67	3	16.1.60	4
167.105	$R\bar{3}'c$	d, $\overline{A}\overline{A}$	$\bar{1}'$	2.3.5	$32'$	18.3.67	1	1.1.1	12
			$\bar{1}'$	2.3.5	$\bar{3}'$	17.3.64	1	1.1.1	12
167.106	$R\bar{3}'c'$	b, $\overline{E}\overline{E}$	$\bar{3}'$	17.3.64	32	18.1.65	3	16.1.60	4
167.106	$R\bar{3}'c'$	d, $\overline{A}\overline{A}$	$\bar{1}'$	2.3.5	32	18.1.65	1	1.1.1	12
			$\bar{1}'$	2.3.5	$\bar{3}'$	17.3.64	1	1.1.1	12
175.139	$P6'/m$	f, $^1\overline{E}\,^2\overline{E}$	$2'/m$	5.3.14	$6'/m$	23.3.84	m	4.1.9	6
			$2'/m$	5.3.14	$\bar{6}$	22.1.79	m	4.1.9	6
175.139	$P6'/m$	g, $^1\overline{E}\,^2\overline{E}$	$2'/m$	5.3.14	$6'/m$	23.3.84	m	4.1.9	6
			$2'/m$	5.3.14	$\bar{6}$	22.1.79	m	4.1.9	6
175.140	$P6/m'$	f, $^1\overline{E}\,^2\overline{E}$	$2/m'$	5.4.15	$6/m'$	23.4.85	m'	4.3.11	6
			$2/m'$	5.4.15	$\bar{6}'$	22.3.81	m'	4.3.11	6
175.140	$P6/m'$	g, $^1\overline{E}\,^2\overline{E}$	$2/m'$	5.4.15	$6/m'$	23.4.85	m'	4.3.11	6
			$2/m'$	5.4.15	$\bar{6}'$	22.3.81	m'	4.3.11	6
176.145	$P6_3'/m$	b, $\overline{E}\overline{E}$	$\bar{3}'$	17.3.64	$\bar{6}$	22.1.79	3	16.1.60	4
176.145	$P6_3'/m$	b, $^1\overline{E}\,^2\overline{E}$	$\bar{3}'$	17.3.64	$\bar{6}$	22.1.79	3	16.1.60	4
176.145	$P6_3'/m$	g, $\overline{A}\overline{A}$	$\bar{1}'$	2.3.5	$\bar{6}$	22.1.79	1	1.1.1	12
			$\bar{1}'$	2.3.5	$\bar{3}'$	17.3.64	1	1.1.1	12
176.146	$P6_3/m'$	b, $\overline{E}\overline{E}$	$\bar{3}'$	17.3.64	$\bar{6}'$	22.3.81	3	16.1.60	4
176.146	$P6_3/m'$	g, $\overline{A}\overline{A}$	$\bar{1}'$	2.3.5	$\bar{6}'$	22.3.81	1	1.1.1	12
			$\bar{1}'$	2.3.5	$\bar{3}'$	17.3.64	1	1.1.1	12
176.147	$P6_3'/m'$	a, $^1\overline{E}\,^2\overline{E}$	$\bar{6}'$	22.3.81	$\bar{3}$	17.1.62	3	16.1.60	4
182.181	$P6_3'2'2$	b, $\overline{E}_1$	32	18.1.65	$32'$	18.3.67	3	16.1.60	4
182.182	$P6_3'22'$	a, $\overline{E}_1$	32	18.1.65	$32'$	18.3.67	3	16.1.60	4
188.218	$P\bar{6}'c2'$	b, $^1\overline{E}\,^2\overline{E}$	$\bar{6}'$	22.3.81	$32'$	18.3.67	3	16.1.60	4
188.218	$P\bar{6}'c2'$	d, $^1\overline{E}\,^2\overline{E}$	$\bar{6}'$	22.3.81	$32'$	18.3.67	3	16.1.60	4
188.218	$P\bar{6}'c2'$	f, $^1\overline{E}\,^2\overline{E}$	$\bar{6}'$	22.3.81	$32'$	18.3.67	3	16.1.60	4
190.229	$P\bar{6}'2'c$	b, $^1\overline{E}\,^2\overline{E}$	$\bar{6}'$	22.3.81	$32'$	18.3.67	3	16.1.60	4
191.235	$P6/m'mm$	f, $\overline{E}$	$m'mm$	8.3.26	$6/m'mm$	27.3.102	m'm2'	7.3.22	6
			$m'mm$	8.3.26	$\bar{6}'m2'$	26.4.98	m'm2'	7.3.22	6
191.235	$P6/m'mm$	g, $\overline{E}$	$m'mm$	8.3.26	$6/m'mm$	27.3.102	m'm2'	7.3.22	6
			$m'mm$	8.3.26	$\bar{6}'m2'$	26.4.98	m'm2'	7.3.22	6
191.236	$P6'/mm'm$	f, $\overline{E}$	$m'mm$	8.3.26	$\bar{6}m'2'$	26.5.99	m'm2'	7.3.22	6
191.236	$P6'/mm'm$	g, $\overline{E}$	$m'mm$	8.3.26	$\bar{6}m'2'$	26.5.99	m'm2'	7.3.22	6
191.237	$P6'/mmm'$	f, $\overline{E}$	$m'mm$	8.3.26	$6'/mmm'$	27.4.103	m'm2'	7.3.22	6
191.237	$P6'/mmm'$	g, $\overline{E}$	$m'mm$	8.3.26	$6'/mmm'$	27.4.103	m'm2'	7.3.22	6
191.241	$P6/m'm'm'$	f, $\overline{E}$	$m'm'm'$	8.5.28	$6/m'm'm'$	27.7.106	m'm'2	7.4.23	6
			$m'm'm'$	8.5.28	$\bar{6}'m'2$	26.3.97	m'm'2	7.4.23	6
191.241	$P6/m'm'm'$	g, $\overline{E}$	$m'm'm'$	8.5.28	$6/m'm'm'$	27.7.106	m'm'2	7.4.23	6
			$m'm'm'$	8.5.28	$\bar{6}'m'2$	26.3.97	m'm'2	7.4.23	6
192.245	$P6/m'cc$	b, $^1\overline{E}_1\,^2\overline{E}_1$	$6/m'$	23.4.85	$62'2'$	24.4.90	6	21.1.76	4
192.245	$P6/m'cc$	b, $^1\overline{E}3\,^2\overline{E}3$	$6/m'$	23.4.85	$62'2'$	24.4.90	6	21.1.76	4
192.245	$P6/m'cc$	b, $^1\overline{E}_2\,^2\overline{E}_2$	$6/m'$	23.4.85	$62'2'$	24.4.90	6	21.1.76	4
192.245	$P6/m'cc$	d, $^1\overline{E}\,^2\overline{E}$	$\bar{6}'$	22.3.81	$32'$	18.3.67	3	16.1.60	8
192.245	$P6/m'cc$	g, $^1\overline{E}\,^2\overline{E}$	$2/m'$	5.4.15	$6/m'$	23.4.85	m'	4.3.11	12
			$2/m'$	5.4.15	$\bar{6}'$	22.3.81	m'	4.3.11	12
			$2/m'$	5.4.15	$2'2'2$	6.3.19	2	3.1.6	12
192.246	$P6'/mc'c$	b, $^1\overline{E}_1\,^2\overline{E}_1$	$6'/m$	23.3.84	$6'22'$	24.3.89	6'	21.3.78	4
192.246	$P6'/mc'c$	g, $^1\overline{E}\,^2\overline{E}$	$2'/m$	5.3.14	$6'/m$	23.3.84	m	4.1.9	12
			$2'/m$	5.3.14	$\bar{6}$	22.1.79	m	4.1.9	12
			$2'/m$	5.3.14	$2'2'2$	6.3.19	2'	3.3.8	12
192.247	$P6'/mcc'$	b, $^1\overline{E}_1\,^2\overline{E}_1$	$6'/m$	23.3.84	$6'22'$	24.3.89	6'	21.3.78	4
192.247	$P6'/mcc'$	c, $\overline{E}_1$	32	18.1.65	$\bar{6}$	22.1.79	3	16.1.60	8
192.247	$P6'/mcc'$	g, $^1\overline{E}\,^2\overline{E}$	$2'/m$	5.3.14	$6'/m$	23.3.84	m	4.1.9	12
			$2'/m$	5.3.14	$\bar{6}$	22.1.79	m	4.1.9	12
			$2'/m$	5.3.14	$2'2'2$	6.3.19	2'	3.3.8	12
192.249	$P6'/m'cc'$	d, $^1\overline{E}\,^2\overline{E}$	$\bar{6}'$	22.3.81	$32'$	18.3.67	3	16.1.60	8
192.251	$P6/m'c'c'$	f, $\overline{E}$	222	6.1.17	622	24.1.87	2	3.1.6	12
			222	6.1.17	32	18.1.65	2	3.1.6	12
			222	6.1.17	$2/m'$	5.4.15	2	3.1.6	12
192.251	$P6/m'c'c'$	g, $^1\overline{E}\,^2\overline{E}$	$2/m'$	5.4.15	$6/m'$	23.4.85	m'	4.3.11	12
			$2/m'$	5.4.15	$\bar{6}'$	22.3.81	m'	4.3.11	12
			$2/m'$	5.4.15	222	6.1.17	2	3.1.6	12
193.255	$P6_3/m'cm$	b, $^1\overline{E}\,^2\overline{E}$	$\bar{3}'1m$	20.3.73	$\bar{6}'m2'$	26.4.98	31m	19.1.68	4
193.255	$P6_3/m'cm$	c, $^1\overline{E}\,^2\overline{E}$	$\bar{6}'$	22.3.81	$32'$	18.3.67	3	16.1.60	8

193.255	$P6_3/m'cm$	f, ${}^1\overline{E}\,{}^2\overline{E}$	$2'/m$	5.3.14	$\bar{6}'m2'$	26.4.98	m	4.1.9	12
			$2'/m$	5.3.14	$\bar{3}'1m$	20.3.73	2'	3.3.8	12
			$2'/m$	5.3.14	$32'$	18.3.67	2'	3.3.8	12
193.256	$P6_3'/mc'm$	f, ${}^1\overline{E}\,{}^2\overline{E}$	$2'/m$	5.3.14	$\bar{6}m2$	26.1.95	m	4.1.9	12
			$2'/m$	5.3.14	$\bar{3}'1m$	20.3.73	2'	3.3.8	12
			$2'/m$	5.3.14	$32'$	18.3.67	2'	3.3.8	12
193.257	$P6_3'/mcm'$	b, ${}^1\overline{E}\,{}^2\overline{E}$	$\bar{3}'1m'$	20.4.74	$\bar{6}m'2'$	26.5.99	31m'	19.3.70	4
193.257	$P6_3'/mcm'$	b,$\overline{E}_1$	$\bar{3}'1m'$	20.4.74	$\bar{6}m'2'$	26.5.99	31m'	19.3.70	4
193.257	$P6_3'/mcm'$	d,$\overline{E}_1$	32	18.1.65	$\bar{6}$	22.1.79	3	16.1.60	8
193.257	$P6_3'/mcm'$	f, ${}^1\overline{E}\,{}^2\overline{E}$	$2/m'$	5.4.15	$\bar{6}m'2'$	26.5.99	m'	4.3.11	12
			$2/m'$	5.4.15	$\bar{3}'1m'$	20.4.74	2	3.1.6	12
			$2/m'$	5.4.15	32	18.1.65	2	3.1.6	12
193.259	$P6_3'/m'cm'$	a,$\overline{E}_1$	$\bar{6}'m'2$	26.3.97	$\bar{3}1m'$	20.5.75	31m'	19.3.70	4
193.259	$P6_3'/m'cm'$	c, ${}^1\overline{E}\,{}^2\overline{E}$	$\bar{6}'$	22.3.81	$32'$	18.3.67	3	16.1.60	8
193.261	$P6_3/m'c'm'$	b, ${}^1\overline{E}\,{}^2\overline{E}$	$\bar{3}'1m'$	20.4.74	$\bar{6}'m'2$	26.3.97	31m'	19.3.70	4
193.261	$P6_3/m'c'm'$	f, ${}^1\overline{E}\,{}^2\overline{E}$	$2/m'$	5.4.15	$\bar{6}'m'2$	26.3.97	m'	4.3.11	12
			$2/m'$	5.4.15	$\bar{3}'1m'$	20.4.74	2	3.1.6	12
			$2/m'$	5.4.15	32	18.1.65	2	3.1.6	12
194.265	$P6_3/m'mc$	a, ${}^1\overline{E}\,{}^2\overline{E}$	$\bar{3}'1m$	20.3.73	$\bar{6}'m2'$	26.4.98	31m	19.1.68	4
194.265	$P6_3/m'mc$	g, ${}^1\overline{E}\,{}^2\overline{E}$	$2'/m$	5.3.14	$\bar{3}'1m$	20.3.73	2'	3.3.8	12
			$2'/m$	5.3.14	$\bar{6}'m2'$	26.4.98	m	4.1.9	12
194.266	$P6_3'/mm'c$	a, ${}^1\overline{E}\,{}^2\overline{E}$	$\bar{3}'1m'$	20.4.74	$\bar{6}m'2'$	26.5.99	31m'	19.3.70	4
194.266	$P6_3'/mm'c$	a,$\overline{E}_1$	$\bar{3}'1m'$	20.4.74	$\bar{6}m'2'$	26.5.99	31m'	19.3.70	4
194.266	$P6_3'/mm'c$	g, ${}^1\overline{E}\,{}^2\overline{E}$	$2/m'$	5.4.15	$\bar{3}'1m'$	20.4.74	2	3.1.6	12
			$2/m'$	5.4.15	$\bar{6}m'2'$	26.5.99	m'	4.3.11	12
194.267	$P6_3'/mmc'$	g, ${}^1\overline{E}\,{}^2\overline{E}$	$2'/m$	5.3.14	$\bar{3}'1m$	20.3.73	2'	3.3.8	12
			$2'/m$	5.3.14	$\bar{6}m2$	26.1.95	m	4.1.9	12
194.268	$P6_3'/m'm'c$	b,$\overline{E}_1$	$\bar{6}'m'2$	26.3.97	$\bar{3}1m'$	20.5.75	31m'	19.3.70	4
194.271	$P6_3/m'm'c'$	a, ${}^1\overline{E}\,{}^2\overline{E}$	$\bar{3}'1m'$	20.4.74	$\bar{6}'m'2$	26.3.97	31m'	19.3.70	4
194.271	$P6_3/m'm'c'$	g, ${}^1\overline{E}\,{}^2\overline{E}$	$2/m'$	5.4.15	$\bar{3}'1m'$	20.4.74	2	3.1.6	12
			$2/m'$	5.4.15	$\bar{6}'m'2$	26.3.97	m'	4.3.11	12
200.16	$Pm'\bar{3}'$	c,$\overline{E}$	$m'm'm'$	8.5.28	$m'\bar{3}'$	29.3.111	m'm'2	7.4.23	6
200.16	$Pm'\bar{3}'$	d,$\overline{E}$	$m'm'm'$	8.5.28	$m'\bar{3}'$	29.3.111	m'm'2	7.4.23	6
201.20	$Pn'\bar{3}'$	b,$\overline{E}\overline{E}$	$\bar{3}'$	17.3.64	23	28.1.107	3	16.1.60	8
201.20	$Pn'\bar{3}'$	b, ${}^1\overline{E}\,{}^2\overline{E}$	$\bar{3}'$	17.3.64	23	28.1.107	3	16.1.60	8
201.20	$Pn'\bar{3}'$	c,$\overline{E}\overline{E}$	$\bar{3}'$	17.3.64	23	28.1.107	3	16.1.60	8
201.20	$Pn'\bar{3}'$	c, ${}^1\overline{E}\,{}^2\overline{E}$	$\bar{3}'$	17.3.64	23	28.1.107	3	16.1.60	8
201.20	$Pn'\bar{3}'$	d,$\overline{E}$	222	6.1.17	23	28.1.107	2	3.1.6	12
202.24	$Fm'\bar{3}'$	d, ${}^1\overline{E}\,{}^2\overline{E}$	$2/m'$	5.4.15	$m'\bar{3}'$	29.3.111	m'	4.3.11	12
			$2/m'$	5.4.15	23	28.1.107	2	3.1.6	12
203.28	$Fd'\bar{3}'$	c,$\overline{E}\overline{E}$	$\bar{3}'$	17.3.64	23	28.1.107	3	16.1.60	8
203.28	$Fd'\bar{3}'$	c, ${}^1\overline{E}\,{}^2\overline{E}$	$\bar{3}'$	17.3.64	23	28.1.107	3	16.1.60	8
203.28	$Fd'\bar{3}'$	d,$\overline{E}\overline{E}$	$\bar{3}'$	17.3.64	23	28.1.107	3	16.1.60	8
203.28	$Fd'\bar{3}'$	d, ${}^1\overline{E}\,{}^2\overline{E}$	$\bar{3}'$	17.3.64	23	28.1.107	3	16.1.60	8
204.32	$Im'\bar{3}'$	b,$\overline{E}$	$m'm'm'$	8.5.28	$m'\bar{3}'$	29.3.111	m'm'2	7.4.23	6
204.32	$Im'\bar{3}'$	c,$\overline{E}\overline{E}$	$\bar{3}'$	17.3.64	$m'\bar{3}'$	29.3.111	3	16.1.60	8
204.32	$Im'\bar{3}'$	c, ${}^1\overline{E}\,{}^2\overline{E}$	$\bar{3}'$	17.3.64	$m'\bar{3}'$	29.3.111	3	16.1.60	8
208.46	$P4_2'32'$	d,$\overline{E}$	222	6.1.17	23	28.1.107	2	3.1.6	12
			222	6.1.17	$2'2'2$	6.3.19	2	3.1.6	12
215.72	$P\bar{4}'3m'$	c,$\overline{E}$	$\bar{4}'2m'$	14.4.51	$\bar{4}'3m'$	31.3.117	m'm'2	7.4.23	6
215.72	$P\bar{4}'3m'$	d,$\overline{E}$	$\bar{4}'2m'$	14.4.51	$\bar{4}'3m'$	31.3.117	m'm'2	7.4.23	6
217.80	$I\bar{4}'3m'$	b,$\overline{E}$	$\bar{4}'2m'$	14.4.51	$\bar{4}'3m'$	31.3.117	m'm'2	7.4.23	6
217.80	$I\bar{4}'3m'$	d, ${}^1\overline{E}\,{}^2\overline{E}$	$\bar{4}'$	10.3.34	$\bar{4}'2m'$	14.4.51	2	3.1.6	12
218.83	$P\bar{4}'3n'$	b,$\overline{E}$	222	6.1.17	23	28.1.107	2	3.1.6	12
			222	6.1.17	$\bar{4}'$	10.3.34	2	3.1.6	12
218.83	$P\bar{4}'3n'$	c, ${}^1\overline{E}\,{}^2\overline{E}$	$\bar{4}'$	10.3.34	222	6.1.17	2	3.1.6	12
218.83	$P\bar{4}'3n'$	d, ${}^1\overline{E}\,{}^2\overline{E}$	$\bar{4}'$	10.3.34	222	6.1.17	2	3.1.6	12
219.87	$F\bar{4}'3c'$	c, ${}^1\overline{E}\,{}^2\overline{E}$	$\bar{4}'$	10.3.34	23	28.1.107	2	3.1.6	12
219.87	$F\bar{4}'3c'$	d, ${}^1\overline{E}\,{}^2\overline{E}$	$\bar{4}'$	10.3.34	23	28.1.107	2	3.1.6	12
221.96	$Pm'\bar{3}'m'$	c,$\overline{E}_2$	$4/m'm'm'$	15.7.59	$m'\bar{3}'m'$	32.5.122	4m'm'	13.4.47	6
221.96	$Pm'\bar{3}'m'$	c,$\overline{E}_1$	$4/m'm'm'$	15.7.59	$m'\bar{3}'m'$	32.5.122	4m'm'	13.4.47	6
221.96	$Pm'\bar{3}'m'$	d,$\overline{E}_2$	$4/m'm'm'$	15.7.59	$m'\bar{3}'m'$	32.5.122	4m'm'	13.4.47	6
221.96	$Pm'\bar{3}'m'$	d,$\overline{E}_1$	$4/m'm'm'$	15.7.59	$m'\bar{3}'m'$	32.5.122	4m'm'	13.4.47	6
222.100	$Pn'\bar{3}'n$	c,$\overline{E}\overline{E}$	$\bar{3}'$	17.3.64	$4'32'$	30.3.114	3	16.1.60	16
222.100	$Pn'\bar{3}'n$	c, ${}^1\overline{E}\,{}^2\overline{E}$	$\bar{3}'$	17.3.64	$4'32'$	30.3.114	3	16.1.60	16
222.101	$Pn\bar{3}n'$	d, ${}^1\overline{E}\,{}^2\overline{E}$	$\bar{4}'$	10.3.34	$4'22'$	12.3.42	2	3.1.6	24
222.102	$Pn'\bar{3}'n'$	b,$\overline{E}_2$	422	12.1.40	432	30.1.112	4	9.1.29	12
222.102	$Pn'\bar{3}'n'$	b,$\overline{E}_1$	422	12.1.40	432	30.1.112	4	9.1.29	12

222.102	$Pn'\bar{3}'n'$	c,$\overline{E}\overline{E}$	$\bar{3}'$	17.3.64	432	30.1.112	3	16.1.60	16
222.102	$Pn'\bar{3}'n'$	c, ${}^1\overline{E}\,{}^2\overline{E}$	$\bar{3}'$	17.3.64	432	30.1.112	3	16.1.60	16
222.102	$Pn'\bar{3}'n'$	d, ${}^1\overline{E}\,{}^2\overline{E}$	$\bar{4}'$	10.3.34	422	12.1.40	2	3.1.6	24
223.106	$Pm'\bar{3}n$	b,$\overline{E}$	$m'm'm'$	8.5.28	$m'\bar{3}'$	29.3.111	m'm'2	7.4.23	12
			$m'm'm'$	8.5.28	$\bar{4}2'm'$	14.5.52	m'm'2	7.4.23	12
223.108	$Pm'\bar{3}'n'$	b,$\overline{E}$	$m'm'm'$	8.5.28	$m'\bar{3}'$	29.3.111	m'm'2	7.4.23	12
			$m'm'm'$	8.5.28	$\bar{4}'2m'$	14.4.51	m'm'2	7.4.23	12
223.108	$Pm'\bar{3}'n'$	e,$\overline{E}_1$	32	18.1.65	$m'\bar{3}'$	29.3.111	3	16.1.60	16
223.108	$Pm'\bar{3}'n'$	c,$\overline{E}$	$\bar{4}'2m'$	14.4.51	$m'm'm'$	8.5.28	m'm'2	7.4.23	12
223.108	$Pm'\bar{3}'n'$	d,$\overline{E}$	$\bar{4}'2m'$	14.4.51	$m'm'm'$	8.5.28	m'm'2	7.4.23	12
224.113	$Pn\bar{3}m'$	d,$\overline{E}$	$\bar{4}'2m'$	14.4.51	$\bar{4}'3m'$	31.3.117	m'm'2	7.4.23	12
224.114	$Pn'\bar{3}'m'$	b, ${}^1\overline{E}\,{}^2\overline{E}$	$\bar{3}'1m'$	20.4.74	$\bar{4}'3m'$	31.3.117	31m'	19.3.70	8
224.114	$Pn'\bar{3}'m'$	b,$\overline{E}_1$	$\bar{3}'1m'$	20.4.74	$\bar{4}'3m'$	31.3.117	31m'	19.3.70	8
224.114	$Pn'\bar{3}'m'$	c, ${}^1\overline{E}\,{}^2\overline{E}$	$\bar{3}'1m'$	20.4.74	$\bar{4}'3m'$	31.3.117	31m'	19.3.70	8
224.114	$Pn'\bar{3}'m'$	c,$\overline{E}_1$	$\bar{3}'1m'$	20.4.74	$\bar{4}'3m'$	31.3.117	31m'	19.3.70	8
224.114	$Pn'\bar{3}'m'$	d,$\overline{E}$	$\bar{4}'2m'$	14.4.51	$\bar{4}'3m'$	31.3.117	m'm'2	7.4.23	12
224.114	$Pn'\bar{3}'m'$	f,$\overline{E}$	222	6.1.17	$\bar{3}'1m'$	20.4.74	2	3.1.6	24
			222	6.1.17	$\bar{4}'2m'$	14.4.51	2	3.1.6	24
225.118	$Fm'\bar{3}'m$	c,$\overline{F}$	$\bar{4}3m$	31.1.115	$m'\bar{3}'m$	32.3.120	31m	19.1.68	8
225.118	$Fm'\bar{3}'m$	d,$\overline{E}$	$m'mm$	8.3.26	$m'\bar{3}'m$	32.3.120	m'm2'	7.3.22	12
225.120	$Fm'\bar{3}'m'$	d,$\overline{E}$	$m'm'm'$	8.5.28	$m'\bar{3}'m'$	32.5.122	m'm'2	7.4.23	12
			$m'm'm'$	8.5.28	$\bar{4}'3m'$	31.3.117	m'm'2	7.4.23	12
226.126	$Fm'\bar{3}'c'$	c,$\overline{E}$	$\bar{4}'2m'$	14.4.51	$m'\bar{3}'$	29.3.111	m'm'2	7.4.23	12
226.126	$Fm'\bar{3}'c'$	d, ${}^1\overline{E}_2\,{}^2\overline{E}_2$	$4/m'$	11.4.38	432	30.1.112	4	9.1.29	12
226.126	$Fm'\bar{3}'c'$	d, ${}^1\overline{E}_1\,{}^2\overline{E}_1$	$4/m'$	11.4.38	432	30.1.112	4	9.1.29	12
227.132	$Fd'\bar{3}'m'$	c, ${}^1\overline{E}\,{}^2\overline{E}$	$\bar{3}'1m'$	20.4.74	$\bar{4}'3m'$	31.3.117	31m'	19.3.70	8
227.132	$Fd'\bar{3}'m'$	c,$\overline{E}_1$	$\bar{3}'1m'$	20.4.74	$\bar{4}'3m'$	31.3.117	31m'	19.3.70	8
227.132	$Fd'\bar{3}'m'$	d, ${}^1\overline{E}\,{}^2\overline{E}$	$\bar{3}'1m'$	20.4.74	$\bar{4}'3m'$	31.3.117	31m'	19.3.70	8
227.132	$Fd'\bar{3}'m'$	d,$\overline{E}_1$	$\bar{3}'1m'$	20.4.74	$\bar{4}'3m'$	31.3.117	31m'	19.3.70	8
228.136	$Fd'\bar{3}'c$	c,$\overline{E}\overline{E}$	$\bar{3}'$	17.3.64	23	28.1.107	3	16.1.60	16
			$\bar{3}'$	17.3.64	$32'$	18.3.67	3	16.1.60	16
228.136	$Fd'\bar{3}'c$	c, ${}^1\overline{E}\,{}^2\overline{E}$	$\bar{3}'$	17.3.64	23	28.1.107	3	16.1.60	16
			$\bar{3}'$	17.3.64	$32'$	18.3.67	3	16.1.60	16
228.137	$Fd\bar{3}c'$	d, ${}^1\overline{E}\,{}^2\overline{E}$	$\bar{4}'$	10.3.34	23	28.1.107	2	3.1.6	24
228.138	$Fd'\bar{3}'c'$	b,$\overline{E}_1$	32	18.1.65	23	28.1.107	3	16.1.60	16
			32	18.1.65	$\bar{3}'$	17.3.64	3	16.1.60	16
228.138	$Fd'\bar{3}'c'$	c,$\overline{E}\overline{E}$	$\bar{3}'$	17.3.64	23	28.1.107	3	16.1.60	16
			$\bar{3}'$	17.3.64	32	18.1.65	3	16.1.60	16
228.138	$Fd'\bar{3}'c'$	c, ${}^1\overline{E}\,{}^2\overline{E}$	$\bar{3}'$	17.3.64	23	28.1.107	3	16.1.60	16
			$\bar{3}'$	17.3.64	32	18.1.65	3	16.1.60	16
228.138	$Fd'\bar{3}'c'$	d, ${}^1\overline{E}\,{}^2\overline{E}$	$\bar{4}'$	10.3.34	23	28.1.107	2	3.1.6	24
229.142	$Im'\bar{3}'m$	c, ${}^1\overline{E}\,{}^2\overline{E}$	$\bar{3}'1m$	20.3.73	$m'\bar{3}'m$	32.3.120	31m	19.1.68	8
229.144	$Im'\bar{3}'m'$	b,$\overline{E}_2$	$4/m'm'm'$	15.7.59	$m'\bar{3}'m'$	32.5.122	4m'm'	13.4.47	6
229.144	$Im'\bar{3}'m'$	b,$\overline{E}_1$	$4/m'm'm'$	15.7.59	$m'\bar{3}'m'$	32.5.122	4m'm'	13.4.47	6
229.144	$Im'\bar{3}'m'$	c, ${}^1\overline{E}\,{}^2\overline{E}$	$\bar{3}'1m'$	20.4.74	$m'\bar{3}'m'$	32.5.122	31m'	19.3.70	8
229.144	$Im'\bar{3}'m'$	c,$\overline{E}_1$	$\bar{3}'1m'$	20.4.74	$m'\bar{3}'m'$	32.5.122	31m'	19.3.70	8
229.144	$Im'\bar{3}'m'$	d,$\overline{E}$	$\bar{4}'2m'$	14.4.51	$4/m'm'm'$	15.7.59	m'm'2	7.4.23	12
230.147	$Ia'\bar{3}'d$	a,$\overline{E}\overline{E}$	$\bar{3}'$	17.3.64	$32'$	18.3.67	3	16.1.60	16
230.147	$Ia'\bar{3}'d$	a, ${}^1\overline{E}\,{}^2\overline{E}$	$\bar{3}'$	17.3.64	$32'$	18.3.67	3	16.1.60	16
230.148	$Ia\bar{3}d'$	d, ${}^1\overline{E}\,{}^2\overline{E}$	$\bar{4}'$	10.3.34	$2'2'2$	6.3.19	2	3.1.6	24
230.149	$Ia'\bar{3}'d'$	a,$\overline{E}\overline{E}$	$\bar{3}'$	17.3.64	32	18.1.65	3	16.1.60	16
230.149	$Ia'\bar{3}'d'$	c,$\overline{E}$	222	6.1.17	32	18.1.65	2	3.1.6	24
			222	6.1.17	$\bar{4}'$	10.3.34	2	3.1.6	24
230.149	$Ia'\bar{3}'d'$	d, ${}^1\overline{E}\,{}^2\overline{E}$	$\bar{4}'$	10.3.34	222	6.1.17	2	3.1.6	24

Supplementary Table 20: Exceptional composite band coreps induced from site-symmetry coreps in the Type-IV single MSGs (SN 6). In order, the columns in this table list the number of the MSG in the BNS setting and the symbol of the MSG, the letter of the maximal Wyckoff position containing $\mathbf{q}$ and the single-valued corep of the site-symmetry group $G_{\mathbf{q}}$, the symbol of the MSG isomorphic to the site-symmetry group $G_{\mathbf{q}}$ in the Hermann-Mauguin notation of the MPOINT tool on the BCS[15–18] and the number of the MSG isomorphic to $G_{\mathbf{q}}$ in the convention established by Litvin in SRef. 10, the symbol and number of the MPG isomorphic to the reducing group $G_{\mathbf{q}'}$, the symbol and number of the MPG isomorphic to the intersection group $G_{\mathbf{q}_0} = G_{\mathbf{q}} \cap G_{\mathbf{q}'}$, and the dimension d of the exceptional composite band corep. See SN 24 for further information regarding exceptional composite band coreps.

MSG		Corep	$G_{\mathbf{q}}$		$G_{\mathbf{q}'}$		$G_{\mathbf{q}_0}$		d
81.36	$P_c\bar{4}$	b,BB	$\bar{4}'$	10.3.34	$\bar{4}$	10.1.32	2	3.1.6	4
81.36	$P_c\bar{4}$	d,BB	$\bar{4}'$	10.3.34	$\bar{4}$	10.1.32	2	3.1.6	4
82.42	$I_c\bar{4}$	b,BB	$\bar{4}'$	10.3.34	$\bar{4}$	10.1.32	2	3.1.6	4
82.42	$I_c\bar{4}$	c,BB	$\bar{4}'$	10.3.34	$\bar{4}$	10.1.32	2	3.1.6	4
83.48	P_c4/m	b,${}^1E\,{}^2E$	$4/m'$	11.4.38	$4/m$	11.1.35	4	9.1.29	4
83.48	P_c4/m	d,${}^1E\,{}^2E$	$4/m'$	11.4.38	$4/m$	11.1.35	4	9.1.29	4
83.50	P_I4/m	d,BB	$\bar{4}'$	10.3.34	$2/m$	5.1.12	2	3.1.6	8
84.57	P_C4_2/m	f,BB	$\bar{4}'$	10.3.34	$2/m$	5.1.12	2	3.1.6	8
85.64	P_c4/n	b,BB	$\bar{4}'$	10.3.34	$\bar{4}$	10.1.32	2	3.1.6	8
86.72	P_c4_2/n	a,BB	$\bar{4}'$	10.3.34	$\bar{4}$	10.1.32	2	3.1.6	8
86.73	P_C4_2/n	e,BB	$\bar{4}'$	10.3.34	$2/m'$	5.4.15	2	3.1.6	8
86.74	P_I4_2/n	d,BB	$\bar{4}'$	10.3.34	$2/m'$	5.4.15	2	3.1.6	8
87.80	I_c4/m	b,${}^1E\,{}^2E$	$4/m'$	11.4.38	$4/m$	11.1.35	4	9.1.29	4
88.86	I_c4_1/a	b,BB	$\bar{4}'$	10.3.34	$\bar{4}$	10.1.32	2	3.1.6	8
89.92	P_c422	a,E	422	12.1.40	$42'2'$	12.4.43	4	9.1.29	4
89.92	P_c422	c,E	422	12.1.40	$42'2'$	12.4.43	4	9.1.29	4
97.156	I_c422	a,E	422	12.1.40	$42'2'$	12.4.43	4	9.1.29	4
111.258	$P_I\bar{4}2m$	d,BB	$\bar{4}'$	10.3.34	222	6.1.17	2	3.1.6	8
112.264	$P_c\bar{4}2c$	b,B_2B_3	$\bar{4}'2m'$	14.4.51	$\bar{4}2'm'$	14.5.52	m'm'2	7.4.23	4
112.264	$P_c\bar{4}2c$	c,B_2B_3	$\bar{4}'2m'$	14.4.51	$\bar{4}2'm'$	14.5.52	m'm'2	7.4.23	4
112.265	$P_C\bar{4}2c$	d,BB	$\bar{4}'$	10.3.34	222	6.1.17	2	3.1.6	8
113.272	$P_c\bar{4}2_1m$	b,BB	$\bar{4}'$	10.3.34	$\bar{4}$	10.1.32	2	3.1.6	8
114.280	$P_c\bar{4}2_1c$	a,BB	$\bar{4}'$	10.3.34	$\bar{4}$	10.1.32	2	3.1.6	8
114.281	$P_C\bar{4}2_1c$	c,BB	$\bar{4}'$	10.3.34	$2'2'2$	6.3.19	2	3.1.6	8
114.282	$P_I\bar{4}2_1c$	d,BB	$\bar{4}'$	10.3.34	$2'2'2$	6.3.19	2	3.1.6	8
116.296	$P_c\bar{4}c2$	a,B_2B_3	$\bar{4}'2m'$	14.4.51	$\bar{4}2'm'$	14.5.52	m'm'2	7.4.23	4
116.296	$P_c\bar{4}c2$	b,B_2B_3	$\bar{4}'2m'$	14.4.51	$\bar{4}2'm'$	14.5.52	m'm'2	7.4.23	4
116.297	$P_C\bar{4}c2$	f,BB	$\bar{4}'$	10.3.34	$2'2'2$	6.3.19	2	3.1.6	8
116.298	$P_I\bar{4}c2$	c,BB	$\bar{4}'$	10.3.34	$2'2'2$	6.3.19	2	3.1.6	8
117.304	$P_c\bar{4}b2$	b,BB	$\bar{4}'$	10.3.34	$\bar{4}$	10.1.32	2	3.1.6	8
117.306	$P_I\bar{4}b2$	c,BB	$\bar{4}'$	10.3.34	222	6.1.17	2	3.1.6	8
118.312	$P_c\bar{4}n2$	a,BB	$\bar{4}'$	10.3.34	$\bar{4}$	10.1.32	2	3.1.6	8
118.313	$P_C\bar{4}n2$	e,BB	$\bar{4}'$	10.3.34	222	6.1.17	2	3.1.6	8
120.326	$I_c\bar{4}c2$	a,B_2B_3	$\bar{4}'2m'$	14.4.51	$\bar{4}2'm'$	14.5.52	m'm'2	7.4.23	4
120.326	$I_c\bar{4}c2$	b,B_2B_3	$\bar{4}'2m'$	14.4.51	$\bar{4}2'm'$	14.5.52	m'm'2	7.4.23	4
121.332	$I_c\bar{4}2m$	b,B_2B_3	$\bar{4}'2m'$	14.4.51	$\bar{4}2'm'$	14.5.52	m'm'2	7.4.23	4
122.338	$I_c\bar{4}2d$	b,BB	$\bar{4}'$	10.3.34	$\bar{4}$	10.1.32	2	3.1.6	8
123.350	P_I4/mmm	d,B_1B_2	$\bar{4}'2'm$	14.3.50	mmm	8.1.24	mm2	7.1.20	8
124.360	P_c4/mcc	b,E	$4/m'm'm'$	15.7.59	$4/mm'm'$	15.6.58	4m'm'	13.4.47	4
124.360	P_c4/mcc	d,E	$4/m'm'm'$	15.7.59	$4/mm'm'$	15.6.58	4m'm'	13.4.47	4
124.361	P_C4/mcc	a,E	422	12.1.40	$4/m$	11.1.35	4	9.1.29	8
124.362	P_I4/mcc	a,E	422	12.1.40	$4/m$	11.1.35	4	9.1.29	8
124.362	P_I4/mcc	b,B_2B_3	$\bar{4}'2m'$	14.4.51	$m'm'm$	8.4.27	m'm'2	7.4.23	8
125.372	P_c4/nbm	a,E	422	12.1.40	$42'2'$	12.4.43	4	9.1.29	8
125.374	P_I4/nbm	b,E	$\bar{4}2m$	14.1.48	$m'mm$	8.3.26	mm2	7.1.20	8
126.384	P_c4/nnc	b,E	422	12.1.40	$42'2'$	12.4.43	4	9.1.29	8
126.384	P_c4/nnc	d,B_2B_3	$\bar{4}'2m'$	14.4.51	$\bar{4}2'm'$	14.5.52	m'm'2	7.4.23	8
127.396	P_c4/mbm	b,${}^1E\,{}^2E$	$4/m'$	11.4.38	$4/m$	11.1.35	4	9.1.29	8
127.398	P_I4/mbm	b,B_1B_2	$\bar{4}'2'm$	14.3.50	mmm	8.1.24	mm2	7.1.20	8
128.408	P_c4/mnc	b,${}^1E\,{}^2E$	$4/m'$	11.4.38	$4/m$	11.1.35	4	9.1.29	8
128.410	P_I4/mnc	d,B_2B_3	$\bar{4}'2m'$	14.4.51	$m'm'm$	8.4.27	m'm'2	7.4.23	8
129.422	P_I4/nmm	d,E	$\bar{4}2m$	14.1.48	$m'mm$	8.3.26	mm2	7.1.20	8
130.432	P_c4/ncc	b,B_2B_3	$\bar{4}'2m'$	14.4.51	$\bar{4}2'm'$	14.5.52	m'm'2	7.4.23	8
130.433	P_C4/ncc	b,${}^1E\,{}^2E$	$4/m'$	11.4.38	$42'2'$	12.4.43	4	9.1.29	8
130.434	P_I4/ncc	c,${}^1E\,{}^2E$	$4/m'$	11.4.38	$42'2'$	12.4.43	4	9.1.29	8
131.445	P_C4_2/mmc	b,E	$\bar{4}2m$	14.1.48	mmm	8.1.24	mm2	7.1.20	8

131.445	P_C4_2/mmc	d,B_1B_2	$\bar{4}'2'm$	14.3.50	mmm	8.1.24	mm2	7.1.20	8
131.446	P_I4_2/mmc	d,E	$\bar{4}2m$	14.1.48	mmm	8.1.24	mm2	7.1.20	8
132.457	P_C4_2/mcm	e,E	$\bar{4}2m$	14.1.48	mmm	8.1.24	mm2	7.1.20	8
132.457	P_C4_2/mcm	f,B_2B_3	$\bar{4}'2m'$	14.4.51	$m'm'm$	8.4.27	m'm'2	7.4.23	8
132.458	P_I4_2/mcm	b,E	$\bar{4}2m$	14.1.48	mmm	8.1.24	mm2	7.1.20	8
133.468	P_c4_2/nbc	c,B_2B_3	$\bar{4}'2m'$	14.4.51	$\bar{4}2'm'$	14.5.52	m'm'2	7.4.23	8
133.469	P_C4_2/nbc	d,B_2B_3	$\bar{4}'2m'$	14.4.51	$m'm'm'$	8.5.28	m'm'2	7.4.23	8
133.470	P_I4_2/nbc	b,B_2B_3	$\bar{4}'2m'$	14.4.51	$m'm'm'$	8.5.28	m'm'2	7.4.23	8
134.481	P_C4_2/nnm	e,E	$\bar{4}2m$	14.1.48	$m'mm$	8.3.26	mm2	7.1.20	8
134.481	P_C4_2/nnm	f,B_2B_3	$\bar{4}'2m'$	14.4.51	$m'm'm'$	8.5.28	m'm'2	7.4.23	8
134.482	P_I4_2/nnm	d,B_2B_3	$\bar{4}'2m'$	14.4.51	$m'm'm'$	8.5.28	m'm'2	7.4.23	8
135.493	P_C4_2/mbc	b,B_2B_3	$\bar{4}'2m'$	14.4.51	$m'm'm$	8.4.27	m'm'2	7.4.23	8
136.505	P_C4_2/mnm	f,B_1B_2	$\bar{4}'2'm$	14.3.50	mmm	8.1.24	mm2	7.1.20	8
137.517	P_C4_2/nmc	b,B_1B_2	$\bar{4}'2'm$	14.3.50	$m'mm$	8.3.26	mm2	7.1.20	8
137.517	P_C4_2/nmc	d,E	$\bar{4}2m$	14.1.48	$m'mm$	8.3.26	mm2	7.1.20	8
137.518	P_I4_2/nmc	d,B_1B_2	$\bar{4}'2'm$	14.3.50	$m'mm$	8.3.26	mm2	7.1.20	8
138.528	P_c4_2/ncm	a,B_2B_3	$\bar{4}'2m'$	14.4.51	$\bar{4}2'm'$	14.5.52	m'm'2	7.4.23	8
138.529	P_C4_2/ncm	e,B_1B_2	$\bar{4}'2'm$	14.3.50	$m'mm$	8.3.26	mm2	7.1.20	8
138.530	P_I4_2/ncm	b,B_1B_2	$\bar{4}'2'm$	14.3.50	$m'mm$	8.3.26	mm2	7.1.20	8
140.550	I_c4/mcm	b,E	$4/m'm'm'$	15.7.59	$4/mm'm'$	15.6.58	4m'm'	13.4.47	4
142.570	I_c4_1/acd	b,B_2B_3	$\bar{4}'2m'$	14.4.51	$\bar{4}2'm'$	14.5.52	m'm'2	7.4.23	8
147.16	$P_c\bar{3}$	b,$^1E\,^2E$	$\bar{3}'$	17.3.64	$\bar{3}$	17.1.62	3	16.1.60	4
148.20	$R_I\bar{3}$	b,$^1E\,^2E$	$\bar{3}'$	17.3.64	$\bar{3}$	17.1.62	3	16.1.60	4
149.24	P_c312	a,E	32	18.1.65	$32'1$	18.3.67	3	16.1.60	4
149.24	P_c312	c,E	32	18.1.65	$32'1$	18.3.67	3	16.1.60	4
149.24	P_c312	e,E	32	18.1.65	$32'1$	18.3.67	3	16.1.60	4
150.28	P_c321	a,E	32	18.1.65	$32'1$	18.3.67	3	16.1.60	4
155.48	R_I32	a,E	32	18.1.65	$32'1$	18.3.67	3	16.1.60	4
162.78	$P_c\bar{3}1m$	c,E	32	18.1.65	$32'1$	18.3.67	3	16.1.60	8
163.84	$P_c\bar{3}1c$	b,E	$\bar{3}'m'1$	20.4.74	$\bar{3}m'1$	20.5.75	3m'1	19.3.70	4
163.84	$P_c\bar{3}1c$	d,E	32	18.1.65	$32'1$	18.3.67	3	16.1.60	8
165.96	$P_c\bar{3}c1$	b,E	$\bar{3}'m'1$	20.4.74	$\bar{3}m'1$	20.5.75	3m'1	19.3.70	4
167.108	$R_I\bar{3}c$	b,E	$\bar{3}'m'1$	20.4.74	$\bar{3}m'1$	20.5.75	3m'1	19.3.70	4
174.136	$P_c\bar{6}$	b,$^1E\,^2E$	$\bar{6}'$	22.3.81	$\bar{6}$	22.1.79	3	16.1.60	4
174.136	$P_c\bar{6}$	d,$^1E\,^2E$	$\bar{6}'$	22.3.81	$\bar{6}$	22.1.79	3	16.1.60	4
174.136	$P_c\bar{6}$	f,$^1E\,^2E$	$\bar{6}'$	22.3.81	$\bar{6}$	22.1.79	3	16.1.60	4
175.142	P_c6/m	b,$^1E_1\,^2E_1$	$6/m'$	23.4.85	$6/m$	23.1.82	6	21.1.76	4
175.142	P_c6/m	b,$^1E_2\,^2E_2$	$6/m'$	23.4.85	$6/m$	23.1.82	6	21.1.76	4
175.142	P_c6/m	d,$^1E\,^2E$	$\bar{6}'$	22.3.81	$\bar{6}$	22.1.79	3	16.1.60	8
176.148	P_c6_3/m	d,$^1E\,^2E$	$\bar{6}'$	22.3.81	$\bar{6}$	22.1.79	3	16.1.60	8
177.154	P_c622	a,E_2	622	24.1.87	$62'2'$	24.4.90	6	21.1.76	4
177.154	P_c622	a,E_1	622	24.1.87	$62'2'$	24.4.90	6	21.1.76	4
177.154	P_c622	c,E	32	18.1.65	$32'1$	18.3.67	3	16.1.60	8
182.184	P_c6_322	d,E	32	18.1.65	$32'1$	18.3.67	3	16.1.60	8
188.220	$P_c\bar{6}c2$	a,E	$\bar{6}'m'2$	26.3.97	$\bar{6}m'2'$	26.5.99	3m'1	19.3.70	4
188.220	$P_c\bar{6}c2$	c,E	$\bar{6}'m'2$	26.3.97	$\bar{6}m'2'$	26.5.99	3m'1	19.3.70	4
188.220	$P_c\bar{6}c2$	e,E	$\bar{6}'m'2$	26.3.97	$\bar{6}m'2'$	26.5.99	3m'1	19.3.70	4
189.226	$P_c\bar{6}2m$	d,$^1E\,^2E$	$\bar{6}'$	22.3.81	$\bar{6}$	22.1.79	3	16.1.60	8
190.232	$P_c\bar{6}2c$	a,E	$\bar{6}'m'2$	26.3.97	$\bar{6}m'2'$	26.5.99	3m'1	19.3.70	4
190.232	$P_c\bar{6}2c$	c,$^1E\,^2E$	$\bar{6}'$	22.3.81	$\bar{6}$	22.1.79	3	16.1.60	8
192.252	P_c6/mcc	b,E_2	$6/m'm'm'$	27.7.106	$6/mm'm'$	27.6.105	6m'm'	25.4.94	4
192.252	P_c6/mcc	b,E_1	$6/m'm'm'$	27.7.106	$6/mm'm'$	27.6.105	6m'm'	25.4.94	4
192.252	P_c6/mcc	d,E	$\bar{6}'m'2$	26.3.97	$\bar{6}m'2'$	26.5.99	3m'1	19.3.70	8
193.262	P_c6_3/mcm	d,E	$\bar{6}'m'2$	26.3.97	$\bar{6}m'2'$	26.5.99	3m'1	19.3.70	8
200.17	$P_Im\bar{3}$	c,$^1E\,^2E$	$\bar{3}'$	17.3.64	$m\bar{3}$	29.1.109	3	16.1.60	16
203.29	$F_Sd\bar{3}$	c,$^1E\,^2E$	$\bar{3}'$	17.3.64	23	28.1.107	3	16.1.60	16
			$\bar{3}'$	17.3.64	$\bar{3}$	17.1.62	3	16.1.60	16
205.36	$P_Ia\bar{3}$	a,$^1E\,^2E$	$\bar{3}'$	17.3.64	$\bar{3}$	17.1.62	3	16.1.60	16
207.43	P_I432	b,E	422	12.1.40	432	30.1.112	4	9.1.29	12
208.47	P_I4_232	c,E	32	18.1.65	$4'32'$	30.3.114	3	16.1.60	16
210.55	F_S4_132	b,E	32	18.1.65	23	28.1.107	3	16.1.60	16
			32	18.1.65	$32'1$	18.3.67	3	16.1.60	16
212.62	P_I4_332	a,E	32	18.1.65	$32'1$	18.3.67	3	16.1.60	16
213.66	P_I4_132	b,E	32	18.1.65	$32'1$	18.3.67	3	16.1.60	16
215.73	$P_I\bar{4}3m$	b,E	$\bar{4}2m$	14.1.48	$\bar{4}3m$	31.1.115	mm2	7.1.20	12
215.73	$P_I\bar{4}3m$	d,BB	$\bar{4}'$	10.3.34	$\bar{4}2m$	14.1.48	2	3.1.6	24
216.77	$F_S\bar{4}3m$	c,B_1B_2	$\bar{4}'2'm$	14.3.50	$\bar{4}3m$	31.1.115	mm2	7.1.20	12
216.77	$F_S\bar{4}3m$	d,B_1B_2	$\bar{4}'2'm$	14.3.50	$\bar{4}3m$	31.1.115	mm2	7.1.20	12
218.84	$P_I\bar{4}3n$	b,B_2B_3	$\bar{4}'2m'$	14.4.51	$\bar{4}'3m'$	31.3.117	m'm'2	7.4.23	12
221.97	$P_Im\bar{3}m$	d,B_1B_2	$\bar{4}'2'm$	14.3.50	$4/mmm$	15.1.53	mm2	7.1.20	24
222.103	$P_In\bar{3}n$	b,E	$4/m'm'm'$	15.7.59	$m'\bar{3}'m'$	32.5.122	4m'm'	13.4.47	12
223.109	$P_Im\bar{3}n$	c,E	$\bar{3}'m'1$	20.4.74	$m\bar{3}m'$	32.4.121	3m'1	19.3.70	16
223.109	$P_Im\bar{3}n$	d,E	$\bar{4}2m$	14.1.48	$4'/mm'm$	15.4.56	mm2	7.1.20	24

224.115	$P_In\bar{3}m$	d,B_2B_3	$\bar{4}'2m'$	14.4.51	$4'/m'm'm$	15.5.57	m'm'2	7.4.23	24
227.133	$F_Sd\bar{3}m$	d,B_1B_2	$\bar{4}'2'm$	14.3.50	$\bar{4}3m$	31.1.115	mm2	7.1.20	24
228.139	$F_Sd\bar{3}c$	c,E	$\bar{3}'m'1$	20.4.74	$\bar{4}'3m'$	31.3.117	3m'1	19.3.70	16
			$\bar{3}'m'1$	20.4.74	$\bar{3}m'1$	20.5.75	3m'1	19.3.70	16

Supplementary Table 21: Exceptional composite band coreps induced from site-symmetry coreps in the Type-IV double MSGs (SN 6). In order, the columns in this table list the number of the MSG in the BNS setting and the symbol of the MSG, the letter of the maximal Wyckoff position containing $\mathbf{q}$ and the double-valued corep of the site-symmetry group $G_{\mathbf{q}}$, the symbol of the MSG isomorphic to the site-symmetry group $G_{\mathbf{q}}$ in the Hermann-Mauguin notation of the MPOINT tool on the BCS[15–18] and the number of the MSG isomorphic to $G_{\mathbf{q}}$ in the convention established by Litvin in SRef. 10, the symbol and number of the MPG isomorphic to the reducing group $G_{\mathbf{q}'}$, the symbol and number of the MPG isomorphic to the intersection group $G_{\mathbf{q}_0} = G_{\mathbf{q}} \cap G_{\mathbf{q}'}$, and the dimension d of the exceptional composite band corep. See SN 24 for further information regarding exceptional composite band coreps.

MSG		Corep	$G_{\mathbf{q}}$		$G_{\mathbf{q}'}$		$G_{\mathbf{q}_0}$		d
2.7	$P_S\bar{1}$	b,$\overline{AA}$	$\bar{1}'$	2.3.5	$\bar{1}$	2.1.3	1	1.1.1	4
2.7	$P_S\bar{1}$	f,$\overline{AA}$	$\bar{1}'$	2.3.5	$\bar{1}$	2.1.3	1	1.1.1	4
2.7	$P_S\bar{1}$	g,$\overline{AA}$	$\bar{1}'$	2.3.5	$\bar{1}$	2.1.3	1	1.1.1	4
2.7	$P_S\bar{1}$	h,$\overline{AA}$	$\bar{1}'$	2.3.5	$\bar{1}$	2.1.3	1	1.1.1	4
10.47	P_a2/m	d,${}^1\overline{E}{}^2\overline{E}$	$2'/m$	5.3.14	$2/m$	5.1.12	m	4.1.9	4
10.47	P_a2/m	e,${}^1\overline{E}{}^2\overline{E}$	$2'/m$	5.3.14	$2/m$	5.1.12	m	4.1.9	4
10.47	P_a2/m	g,${}^1\overline{E}{}^2\overline{E}$	$2'/m$	5.3.14	$2/m$	5.1.12	m	4.1.9	4
10.47	P_a2/m	h,${}^1\overline{E}{}^2\overline{E}$	$2'/m$	5.3.14	$2/m$	5.1.12	m	4.1.9	4
10.48	P_b2/m	b,${}^1\overline{E}{}^2\overline{E}$	$2/m'$	5.4.15	$2/m$	5.1.12	2	3.1.6	4
10.48	P_b2/m	e,${}^1\overline{E}{}^2\overline{E}$	$2/m'$	5.4.15	$2/m$	5.1.12	2	3.1.6	4
10.48	P_b2/m	f,${}^1\overline{E}{}^2\overline{E}$	$2/m'$	5.4.15	$2/m$	5.1.12	2	3.1.6	4
10.48	P_b2/m	h,${}^1\overline{E}{}^2\overline{E}$	$2/m'$	5.4.15	$2/m$	5.1.12	2	3.1.6	4
10.49	P_C2/m	e,$\overline{AA}$	$\bar{1}'$	2.3.5	$2/m$	5.1.12	1	1.1.1	8
10.49	P_C2/m	f,$\overline{AA}$	$\bar{1}'$	2.3.5	$2/m$	5.1.12	1	1.1.1	8
11.55	P_a2_1/m	b,$\overline{AA}$	$\bar{1}'$	2.3.5	$\bar{1}$	2.1.3	1	1.1.1	8
			$\bar{1}'$	2.3.5	m	4.1.9	1	1.1.1	8
11.55	P_a2_1/m	d,$\overline{AA}$	$\bar{1}'$	2.3.5	$\bar{1}$	2.1.3	1	1.1.1	8
			$\bar{1}'$	2.3.5	m	4.1.9	1	1.1.1	8
11.56	P_b2_1/m	a,${}^1\overline{E}{}^2\overline{E}$	$2'/m$	5.3.14	$2'/m'$	5.5.16	2'	3.3.8	4
11.56	P_b2_1/m	c,${}^1\overline{E}{}^2\overline{E}$	$2'/m$	5.3.14	$2'/m'$	5.5.16	2'	3.3.8	4
11.56	P_b2_1/m	d,${}^1\overline{E}{}^2\overline{E}$	$2'/m$	5.3.14	$2'/m'$	5.5.16	2'	3.3.8	4
11.56	P_b2_1/m	g,${}^1\overline{E}{}^2\overline{E}$	$2'/m$	5.3.14	$2'/m'$	5.5.16	2'	3.3.8	4
12.63	C_c2/m	c,${}^1\overline{E}{}^2\overline{E}$	$2'/m$	5.3.14	$2/m$	5.1.12	m	4.1.9	4
12.63	C_c2/m	d,${}^1\overline{E}{}^2\overline{E}$	$2'/m$	5.3.14	$2/m$	5.1.12	m	4.1.9	4
12.63	C_c2/m	f,$\overline{AA}$	$\bar{1}'$	2.3.5	$2/m$	5.1.12	1	1.1.1	8
			$\bar{1}'$	2.3.5	$2'/m$	5.3.14	1	1.1.1	8
			$\bar{1}'$	2.3.5	$\bar{1}$	2.1.3	1	1.1.1	8
12.64	C_a2/m	b,${}^1\overline{E}{}^2\overline{E}$	$2/m'$	5.4.15	$2/m$	5.1.12	2	3.1.6	4
			$2/m'$	5.4.15	$2'/m'$	5.5.16	m'	4.3.11	4
12.64	C_a2/m	d,${}^1\overline{E}{}^2\overline{E}$	$2'/m$	5.3.14	$2/m$	5.1.12	m	4.1.9	4
			$2'/m$	5.3.14	$2'/m'$	5.5.16	2'	3.3.8	4
12.64	C_a2/m	f,${}^1\overline{E}{}^2\overline{E}$	$2/m'$	5.4.15	$2/m$	5.1.12	2	3.1.6	4
			$2/m'$	5.4.15	$2'/m'$	5.5.16	m'	4.3.11	4
12.64	C_a2/m	g,${}^1\overline{E}{}^2\overline{E}$	$2'/m$	5.3.14	$2/m$	5.1.12	m	4.1.9	4
			$2'/m$	5.3.14	$2'/m'$	5.5.16	2'	3.3.8	4
13.70	P_a2/c	b,$\overline{AA}$	$\bar{1}'$	2.3.5	$\bar{1}$	2.1.3	1	1.1.1	8
			$\bar{1}'$	2.3.5	2	3.1.6	1	1.1.1	8
			$\bar{1}'$	2.3.5	2'	3.3.8	1	1.1.1	8
13.70	P_a2/c	d,$\overline{AA}$	$\bar{1}'$	2.3.5	$\bar{1}$	2.1.3	1	1.1.1	8
			$\bar{1}'$	2.3.5	2	3.1.6	1	1.1.1	8
			$\bar{1}'$	2.3.5	2'	3.3.8	1	1.1.1	8
13.71	P_b2/c	b,$\overline{AA}$	$\bar{1}'$	2.3.5	$\bar{1}$	2.1.3	1	1.1.1	8
			$\bar{1}'$	2.3.5	2	3.1.6	1	1.1.1	8
13.71	P_b2/c	c,$\overline{AA}$	$\bar{1}'$	2.3.5	$\bar{1}$	2.1.3	1	1.1.1	8
			$\bar{1}'$	2.3.5	2	3.1.6	1	1.1.1	8
13.72	P_c2/c	a,${}^1\overline{E}{}^2\overline{E}$	$2/m'$	5.4.15	$2'/m'$	5.5.16	m'	4.3.11	4

13.72	P_c2/c	b, $^1\overline{E}\,^2\overline{E}$	$2/m'$	5.4.15	$2'/m'$	5.5.16	m’	4.3.11	4
13.72	P_c2/c	d, $^1\overline{E}\,^2\overline{E}$	$2/m'$	5.4.15	$2'/m'$	5.5.16	m’	4.3.11	4
13.72	P_c2/c	e, $^1\overline{E}\,^2\overline{E}$	$2/m'$	5.4.15	$2'/m'$	5.5.16	m’	4.3.11	4
13.74	P_C2/c	c,$\overline{A}\overline{A}$	$\bar{1}'$	2.3.5	$\bar{1}$	2.1.3	1	1.1.1	8
			$\bar{1}'$	2.3.5	2	3.1.6	1	1.1.1	8
13.74	P_C2/c	d,$\overline{A}\overline{A}$	$\bar{1}'$	2.3.5	$\bar{1}$	2.1.3	1	1.1.1	8
			$\bar{1}'$	2.3.5	2	3.1.6	1	1.1.1	8
14.80	P_a2_1/c	b,$\overline{A}\overline{A}$	$\bar{1}'$	2.3.5	$\bar{1}$	2.1.3	1	1.1.1	8
14.80	P_a2_1/c	d,$\overline{A}\overline{A}$	$\bar{1}'$	2.3.5	$\bar{1}$	2.1.3	1	1.1.1	8
14.81	P_b2_1/c	a,$\overline{A}\overline{A}$	$\bar{1}'$	2.3.5	$\bar{1}$	2.1.3	1	1.1.1	8
			$\bar{1}'$	2.3.5	$2'$	3.3.8	1	1.1.1	8
14.81	P_b2_1/c	d,$\overline{A}\overline{A}$	$\bar{1}'$	2.3.5	$\bar{1}$	2.1.3	1	1.1.1	8
			$\bar{1}'$	2.3.5	$2'$	3.3.8	1	1.1.1	8
14.82	P_c2_1/c	a,$\overline{A}\overline{A}$	$\bar{1}'$	2.3.5	$\bar{1}$	2.1.3	1	1.1.1	8
			$\bar{1}'$	2.3.5	m'	4.3.11	1	1.1.1	8
14.82	P_c2_1/c	b,$\overline{A}\overline{A}$	$\bar{1}'$	2.3.5	$\bar{1}$	2.1.3	1	1.1.1	8
			$\bar{1}'$	2.3.5	m'	4.3.11	1	1.1.1	8
14.83	P_A2_1/c	e,$\overline{A}\overline{A}$	$\bar{1}'$	2.3.5	$2'/m'$	5.5.16	1	1.1.1	8
14.83	P_A2_1/c	f,$\overline{A}\overline{A}$	$\bar{1}'$	2.3.5	$2'/m'$	5.5.16	1	1.1.1	8
14.84	P_C2_1/c	a,$\overline{A}\overline{A}$	$\bar{1}'$	2.3.5	$\bar{1}$	2.1.3	1	1.1.1	8
			$\bar{1}'$	2.3.5	$2'$	3.3.8	1	1.1.1	8
14.84	P_C2_1/c	b,$\overline{A}\overline{A}$	$\bar{1}'$	2.3.5	$\bar{1}$	2.1.3	1	1.1.1	8
			$\bar{1}'$	2.3.5	$2'$	3.3.8	1	1.1.1	8
15.90	C_c2/c	a, $^1\overline{E}\,^2\overline{E}$	$2/m'$	5.4.15	$2'/m'$	5.5.16	m’	4.3.11	4
15.90	C_c2/c	b, $^1\overline{E}\,^2\overline{E}$	$2/m'$	5.4.15	$2'/m'$	5.5.16	m’	4.3.11	4
15.90	C_c2/c	e,$\overline{A}\overline{A}$	$\bar{1}'$	2.3.5	$2/m'$	5.4.15	1	1.1.1	8
			$\bar{1}'$	2.3.5	$2'/m'$	5.5.16	1	1.1.1	8
			$\bar{1}'$	2.3.5	$\bar{1}$	2.1.3	1	1.1.1	8
15.91	C_a2/c	c,$\overline{A}\overline{A}$	$\bar{1}'$	2.3.5	$\bar{1}$	2.1.3	1	1.1.1	8
			$\bar{1}'$	2.3.5	2	3.1.6	1	1.1.1	8
			$\bar{1}'$	2.3.5	$2'$	3.3.8	1	1.1.1	8
15.91	C_a2/c	d,$\overline{A}\overline{A}$	$\bar{1}'$	2.3.5	$\bar{1}$	2.1.3	1	1.1.1	8
			$\bar{1}'$	2.3.5	2	3.1.6	1	1.1.1	8
			$\bar{1}'$	2.3.5	$2'$	3.3.8	1	1.1.1	8
16.4	P_a222	a,$\overline{E}$	222	6.1.17	$2'2'2$	6.3.19	2	3.1.6	4
16.4	P_a222	c,$\overline{E}$	222	6.1.17	$2'2'2$	6.3.19	2	3.1.6	4
16.4	P_a222	d,$\overline{E}$	222	6.1.17	$2'2'2$	6.3.19	2	3.1.6	4
16.4	P_a222	g,$\overline{E}$	222	6.1.17	$2'2'2$	6.3.19	2	3.1.6	4
21.42	C_c222	a,$\overline{E}$	222	6.1.17	$2'2'2$	6.3.19	2	3.1.6	4
21.42	C_c222	b,$\overline{E}$	222	6.1.17	$2'2'2$	6.3.19	2	3.1.6	4
21.43	C_a222	a,$\overline{E}$	222	6.1.17	$2'2'2$	6.3.19	2	3.1.6	4
21.43	C_a222	d,$\overline{E}$	222	6.1.17	$2'2'2$	6.3.19	2	3.1.6	4
22.48	F_S222	a,$\overline{E}$	222	6.1.17	$2'2'2$	6.3.19	2	3.1.6	4
22.48	F_S222	h,$\overline{E}$	222	6.1.17	$2'2'2$	6.3.19	2	3.1.6	4
23.52	I_c222	a,$\overline{E}$	222	6.1.17	$2'2'2$	6.3.19	2	3.1.6	4
23.52	I_c222	b,$\overline{E}$	222	6.1.17	$2'2'2$	6.3.19	2	3.1.6	4
25.62	P_amm2	a,$\overline{E}$	$mm2$	7.1.20	$m'm2'$	7.3.22	m	4.1.9	4
25.62	P_amm2	b,$\overline{E}$	$mm2$	7.1.20	$m'm2'$	7.3.22	m	4.1.9	4
35.170	C_amm2	a,$\overline{E}$	$mm2$	7.1.20	$m'm2'$	7.3.22	m	4.1.9	4
38.192	A_amm2	a,$\overline{E}$	$mm2$	7.1.20	$m'm2'$	7.3.22	m	4.1.9	4
38.193	A_bmm2	a,$\overline{E}$	$mm2$	7.1.20	$m'm2'$	7.3.22	m	4.1.9	4
38.193	A_bmm2	c,$\overline{E}$	$mm2$	7.1.20	$m'm2'$	7.3.22	m	4.1.9	4
42.223	F_Smm2	a,$\overline{E}$	$mm2$	7.1.20	$m'm2'$	7.3.22	m	4.1.9	4
44.234	I_amm2	a,$\overline{E}$	$mm2$	7.1.20	$m'm2'$	7.3.22	m	4.1.9	4
47.255	P_Cmmm	e, $^1\overline{E}\,^2\overline{E}$	$2'/m$	5.3.14	mmm	8.1.24	m	4.1.9	8
47.255	P_Cmmm	f, $^1\overline{E}\,^2\overline{E}$	$2'/m$	5.3.14	mmm	8.1.24	m	4.1.9	8
47.256	P_Immm	k,$\overline{A}\overline{A}$	$\bar{1}'$	2.3.5	mmm	8.1.24	1	1.1.1	16
48.262	P_cnnn	c,$\overline{E}$	222	6.1.17	$2'2'2$	6.3.19	2	3.1.6	8
48.262	P_cnnn	d,$\overline{E}$	222	6.1.17	$2'2'2$	6.3.19	2	3.1.6	8
48.262	P_cnnn	f,$\overline{A}\overline{A}$	$\bar{1}'$	2.3.5	$2'2'2$	6.3.19	1	1.1.1	16
			$\bar{1}'$	2.3.5	222	6.1.17	1	1.1.1	16
			$\bar{1}'$	2.3.5	$\bar{1}$	2.1.3	1	1.1.1	16
48.263	P_Cnnn	c, $^1\overline{E}\,^2\overline{E}$	$2/m'$	5.4.15	222	6.1.17	2	3.1.6	8
			$2/m'$	5.4.15	$2'/m'$	5.5.16	m’	4.3.11	8
48.263	P_Cnnn	d, $^1\overline{E}\,^2\overline{E}$	$2/m'$	5.4.15	222	6.1.17	2	3.1.6	8
			$2/m'$	5.4.15	$2'/m'$	5.5.16	m’	4.3.11	8
48.263	P_Cnnn	a,$\overline{E}$	222	6.1.17	$2/m'$	5.4.15	2	3.1.6	8
48.263	P_Cnnn	b,$\overline{E}$	222	6.1.17	$2/m'$	5.4.15	2	3.1.6	8
49.272	P_accm	b, $^1\overline{E}\,^2\overline{E}$	$2'/m$	5.3.14	$2/m$	5.1.12	m	4.1.9	8
			$2'/m$	5.3.14	$2'2'2$	6.3.19	2’	3.3.8	8
49.272	P_accm	d, $^1\overline{E}\,^2\overline{E}$	$2'/m$	5.3.14	$2/m$	5.1.12	m	4.1.9	8

			$2'/m$	5.3.14	$2'2'2$	6.3.19	2'	3.3.8	8
49.272	P_accm	e,$\overline{E}$	222	6.1.17	$2/m$	5.1.12	2	3.1.6	8
			222	6.1.17	$2'2'2$	6.3.19	2	3.1.6	8
49.272	P_accm	g,$\overline{E}$	222	6.1.17	$2/m$	5.1.12	2	3.1.6	8
			222	6.1.17	$2'2'2$	6.3.19	2	3.1.6	8
49.273	P_cccm	c,$\overline{E}$	$m'm'm'$	8.5.28	$m'm'm$	8.4.27	m'm'2	7.4.23	4
49.273	P_cccm	d,$\overline{E}$	$m'm'm'$	8.5.28	$m'm'm$	8.4.27	m'm'2	7.4.23	4
49.273	P_cccm	g,$\overline{E}$	$m'm'm'$	8.5.28	$m'm'm$	8.4.27	m'm'2	7.4.23	4
49.273	P_cccm	h,$\overline{E}$	$m'm'm'$	8.5.28	$m'm'm$	8.4.27	m'm'2	7.4.23	4
49.274	P_Bccm	a,$\overline{E}$	222	6.1.17	$2/m$	5.1.12	2	3.1.6	8
			222	6.1.17	$2/m'$	5.4.15	2	3.1.6	8
49.274	P_Bccm	b,$\overline{E}$	222	6.1.17	$2/m$	5.1.12	2	3.1.6	8
			222	6.1.17	$2/m'$	5.4.15	2	3.1.6	8
49.274	P_Bccm	e, $^1\overline{E}\,^2\overline{E}$	$2/m'$	5.4.15	222	6.1.17	2	3.1.6	8
			$2/m'$	5.4.15	$m'm2'$	7.3.22	m'	4.3.11	8
49.274	P_Bccm	f, $^1\overline{E}\,^2\overline{E}$	$2/m'$	5.4.15	222	6.1.17	2	3.1.6	8
			$2/m'$	5.4.15	$m'm2'$	7.3.22	m'	4.3.11	8
49.275	P_Cccm	a,$\overline{E}$	222	6.1.17	$2/m$	5.1.12	2	3.1.6	8
49.275	P_Cccm	b,$\overline{E}$	222	6.1.17	$2/m$	5.1.12	2	3.1.6	8
49.275	P_Cccm	e, $^1\overline{E}\,^2\overline{E}$	$2'/m$	5.3.14	$2/m$	5.1.12	m	4.1.9	8
49.275	P_Cccm	f, $^1\overline{E}\,^2\overline{E}$	$2'/m$	5.3.14	$2/m$	5.1.12	m	4.1.9	8
49.276	P_Iccm	a,$\overline{E}$	222	6.1.17	$2/m$	5.1.12	2	3.1.6	8
49.276	P_Iccm	b,$\overline{E}$	222	6.1.17	$2/m$	5.1.12	2	3.1.6	8
49.276	P_Iccm	e,$\overline{AA}$	$\bar{1}'$	2.3.5	222	6.1.17	1	1.1.1	16
			$\bar{1}'$	2.3.5	$2/m$	5.1.12	1	1.1.1	16
50.284	P_aban	a, $^1\overline{E}\,^2\overline{E}$	$2/m'$	5.4.15	$2'/m'$	5.5.16	m'	4.3.11	8
			$2/m'$	5.4.15	222	6.1.17	2	3.1.6	8
50.284	P_aban	c, $^1\overline{E}\,^2\overline{E}$	$2/m'$	5.4.15	$2'/m'$	5.5.16	m'	4.3.11	8
			$2/m'$	5.4.15	222	6.1.17	2	3.1.6	8
50.284	P_aban	e,$\overline{E}$	222	6.1.17	$2/m'$	5.4.15	2	3.1.6	8
			222	6.1.17	$2'2'2$	6.3.19	2	3.1.6	8
50.284	P_aban	g,$\overline{E}$	222	6.1.17	$2/m'$	5.4.15	2	3.1.6	8
			222	6.1.17	$2'2'2$	6.3.19	2	3.1.6	8
50.285	P_cban	a,$\overline{E}$	222	6.1.17	$2'2'2$	6.3.19	2	3.1.6	8
50.285	P_cban	b,$\overline{E}$	222	6.1.17	$2'2'2$	6.3.19	2	3.1.6	8
50.285	P_cban	f,$\overline{AA}$	$\bar{1}'$	2.3.5	222	6.1.17	1	1.1.1	16
			$\bar{1}'$	2.3.5	$2'2'2$	6.3.19	1	1.1.1	16
			$\bar{1}'$	2.3.5	$\bar{1}$	2.1.3	1	1.1.1	16
50.286	P_Aban	c,$\overline{AA}$	$\bar{1}'$	2.3.5	222	6.1.17	1	1.1.1	16
			$\bar{1}'$	2.3.5	$\bar{1}$	2.1.3	1	1.1.1	16
			$\bar{1}'$	2.3.5	$2'$	3.3.8	1	1.1.1	16
51.298	P_amma	a,$\overline{E}$	$m'mm$	8.3.26	$m'm'm$	8.4.27	m'm2'	7.3.22	4
51.298	P_amma	c,$\overline{E}$	$m'mm$	8.3.26	$m'm'm$	8.4.27	m'm2'	7.3.22	4
51.298	P_amma	e,$\overline{E}$	$m'mm$	8.3.26	$m'm'm$	8.4.27	m'm2'	7.3.22	4
51.298	P_amma	g,$\overline{E}$	$m'mm$	8.3.26	$m'm'm$	8.4.27	m'm2'	7.3.22	4
51.299	P_bmma	b, $^1\overline{E}\,^2\overline{E}$	$2/m'$	5.4.15	$2/m$	5.1.12	2	3.1.6	8
			$2/m'$	5.4.15	$m'm2'$	7.3.22	m'	4.3.11	8
51.299	P_bmma	d, $^1\overline{E}\,^2\overline{E}$	$2/m'$	5.4.15	$2/m$	5.1.12	2	3.1.6	8
			$2/m'$	5.4.15	$m'm2'$	7.3.22	m'	4.3.11	8
51.299	P_bmma	e,$\overline{E}$	$mm2$	7.1.20	$2/m$	5.1.12	m	4.1.9	8
			$mm2$	7.1.20	$m'm2'$	7.3.22	m	4.1.9	8
51.300	P_cmma	c, $^1\overline{E}\,^2\overline{E}$	$2'/m$	5.3.14	$2/m$	5.1.12	m	4.1.9	8
			$2'/m$	5.3.14	$mm2$	7.1.20	m	4.1.9	8
51.300	P_cmma	d, $^1\overline{E}\,^2\overline{E}$	$2'/m$	5.3.14	$2/m$	5.1.12	m	4.1.9	8
			$2'/m$	5.3.14	$mm2$	7.1.20	m	4.1.9	8
51.300	P_cmma	e,$\overline{E}$	$mm2$	7.1.20	$2/m$	5.1.12	m	4.1.9	8
			$mm2$	7.1.20	$2'/m$	5.3.14	m	4.1.9	8
51.300	P_cmma	f,$\overline{E}$	$mm2$	7.1.20	$2/m$	5.1.12	m	4.1.9	8
			$mm2$	7.1.20	$2'/m$	5.3.14	m	4.1.9	8
51.301	P_Amma	c,$\overline{E}$	$mm2$	7.1.20	$2/m$	5.1.12	m	4.1.9	8
51.301	P_Amma	d,$\overline{AA}$	$\bar{1}'$	2.3.5	$2/m$	5.1.12	1	1.1.1	16
			$\bar{1}'$	2.3.5	$mm2$	7.1.20	1	1.1.1	16
51.303	P_Cmma	c, $^1\overline{E}\,^2\overline{E}$	$2'/m$	5.3.14	$2'2'2$	6.3.19	2'	3.3.8	8
			$2'/m$	5.3.14	$mm2$	7.1.20	m	4.1.9	8
51.303	P_Cmma	d, $^1\overline{E}\,^2\overline{E}$	$2'/m$	5.3.14	$2'2'2$	6.3.19	2'	3.3.8	8
			$2'/m$	5.3.14	$mm2$	7.1.20	m	4.1.9	8
51.303	P_Cmma	g,$\overline{E}$	$mm2$	7.1.20	$2'/m$	5.3.14	m	4.1.9	8
			$mm2$	7.1.20	$2/m$	5.1.12	m	4.1.9	8
51.304	P_Imma	e,$\overline{E}$	$mm2$	7.1.20	$2/m$	5.1.12	m	4.1.9	8
			$mm2$	7.1.20	$2'/m$	5.3.14	m	4.1.9	8

51.304	$P_{I}mma$	c, ${}^1\overline{E}\,{}^2\overline{E}$	$2'/m$	5.3.14	$mm2$	7.1.20	m	4.1.9	8
51.304	$P_{I}mma$	d, ${}^1\overline{E}\,{}^2\overline{E}$	$2'/m$	5.3.14	$mm2$	7.1.20	m	4.1.9	8
52.314	$P_{a}nna$	c, ${}^1\overline{E}\,{}^2\overline{E}$	$2/m'$	5.4.15	$2'/m'$	5.5.16	m’	4.3.11	8
52.314	$P_{a}nna$	d, ${}^1\overline{E}\,{}^2\overline{E}$	$2/m'$	5.4.15	$2'/m'$	5.5.16	m’	4.3.11	8
52.315	$P_{b}nna$	e,$\overline{AA}$	$\bar{1}'$	2.3.5	$2'2'2$	6.3.19	1	1.1.1	16
			$\bar{1}'$	2.3.5	$\bar{1}$	2.1.3	1	1.1.1	16
52.316	$P_{c}nna$	b,$\overline{AA}$	$\bar{1}'$	2.3.5	$\bar{1}$	2.1.3	1	1.1.1	16
			$\bar{1}'$	2.3.5	2	3.1.6	1	1.1.1	16
			$\bar{1}'$	2.3.5	$2'$	3.3.8	1	1.1.1	16
52.317	$P_{A}nna$	e, ${}^1\overline{E}\,{}^2\overline{E}$	$2/m'$	5.4.15	$2'/m'$	5.5.16	m’	4.3.11	8
52.317	$P_{A}nna$	f, ${}^1\overline{E}\,{}^2\overline{E}$	$2/m'$	5.4.15	$2'/m'$	5.5.16	m’	4.3.11	8
52.318	$P_{B}nna$	a, ${}^1\overline{E}\,{}^2\overline{E}$	$2/m'$	5.4.15	$m'm'2$	7.4.23	m’	4.3.11	8
52.318	$P_{B}nna$	b, ${}^1\overline{E}\,{}^2\overline{E}$	$2/m'$	5.4.15	$m'm'2$	7.4.23	m’	4.3.11	8
52.319	$P_{C}nna$	c,$\overline{AA}$	$\bar{1}'$	2.3.5	$2'2'2$	6.3.19	1	1.1.1	16
			$\bar{1}'$	2.3.5	$\bar{1}$	2.1.3	1	1.1.1	16
			$\bar{1}'$	2.3.5	2	3.1.6	1	1.1.1	16
52.320	$P_{I}nna$	c, ${}^1\overline{E}\,{}^2\overline{E}$	$2/m'$	5.4.15	$m'm'2$	7.4.23	m’	4.3.11	8
52.320	$P_{I}nna$	d, ${}^1\overline{E}\,{}^2\overline{E}$	$2/m'$	5.4.15	$m'm'2$	7.4.23	m’	4.3.11	8
53.330	$P_{a}mna$	b, ${}^1\overline{E}\,{}^2\overline{E}$	$2/m'$	5.4.15	$2/m$	5.1.12	2	3.1.6	8
			$2/m'$	5.4.15	$m'm'2$	7.4.23	m’	4.3.11	8
53.330	$P_{a}mna$	d, ${}^1\overline{E}\,{}^2\overline{E}$	$2/m'$	5.4.15	$2/m$	5.1.12	2	3.1.6	8
			$2/m'$	5.4.15	$m'm'2$	7.4.23	m’	4.3.11	8
53.331	$P_{b}mna$	c, ${}^1\overline{E}\,{}^2\overline{E}$	$2'/m$	5.3.14	$2/m$	5.1.12	m	4.1.9	8
53.331	$P_{b}mna$	d, ${}^1\overline{E}\,{}^2\overline{E}$	$2'/m$	5.3.14	$2/m$	5.1.12	m	4.1.9	8
53.332	$P_{c}mna$	b, ${}^1\overline{E}\,{}^2\overline{E}$	$2'/m$	5.3.14	$2/m$	5.1.12	m	4.1.9	8
			$2'/m$	5.3.14	$2'2'2$	6.3.19	2’	3.3.8	8
53.332	$P_{c}mna$	d, ${}^1\overline{E}\,{}^2\overline{E}$	$2'/m$	5.3.14	$2/m$	5.1.12	m	4.1.9	8
			$2'/m$	5.3.14	$2'2'2$	6.3.19	2’	3.3.8	8
53.333	$P_{A}mna$	c, ${}^1\overline{E}\,{}^2\overline{E}$	$2'/m$	5.3.14	$2'2'2$	6.3.19	2’	3.3.8	8
			$2'/m$	5.3.14	$2/m$	5.1.12	m	4.1.9	8
53.333	$P_{A}mna$	d, ${}^1\overline{E}\,{}^2\overline{E}$	$2'/m$	5.3.14	$2'2'2$	6.3.19	2’	3.3.8	8
			$2'/m$	5.3.14	$2/m$	5.1.12	m	4.1.9	8
53.334	$P_{B}mna$	e, ${}^1\overline{E}\,{}^2\overline{E}$	$2/m'$	5.4.15	$m'm'm$	8.4.27	m’	4.3.11	8
53.334	$P_{B}mna$	f, ${}^1\overline{E}\,{}^2\overline{E}$	$2/m'$	5.4.15	$m'm'm$	8.4.27	m’	4.3.11	8
53.335	$P_{C}mna$	c,$\overline{AA}$	$\bar{1}'$	2.3.5	$2/m$	5.1.12	1	1.1.1	16
			$\bar{1}'$	2.3.5	2	3.1.6	1	1.1.1	16
53.336	$P_{I}mna$	c, ${}^1\overline{E}\,{}^2\overline{E}$	$2/m'$	5.4.15	$m'm2'$	7.3.22	m’	4.3.11	8
53.336	$P_{I}mna$	d, ${}^1\overline{E}\,{}^2\overline{E}$	$2/m'$	5.4.15	$m'm2'$	7.3.22	m’	4.3.11	8
54.346	$P_{a}cca$	a, ${}^1\overline{E}\,{}^2\overline{E}$	$2/m'$	5.4.15	$2'/m'$	5.5.16	m’	4.3.11	8
			$2/m'$	5.4.15	$2'2'2$	6.3.19	2	3.1.6	8
54.346	$P_{a}cca$	c, ${}^1\overline{E}\,{}^2\overline{E}$	$2/m'$	5.4.15	$2'/m'$	5.5.16	m’	4.3.11	8
			$2/m'$	5.4.15	$2'2'2$	6.3.19	2	3.1.6	8
54.347	$P_{b}cca$	b,$\overline{AA}$	$\bar{1}'$	2.3.5	$\bar{1}$	2.1.3	1	1.1.1	16
			$\bar{1}'$	2.3.5	2	3.1.6	1	1.1.1	16
			$\bar{1}'$	2.3.5	$2'$	3.3.8	1	1.1.1	16
54.348	$P_{c}cca$	c, ${}^1\overline{E}\,{}^2\overline{E}$	$2/m'$	5.4.15	$2'/m'$	5.5.16	m’	4.3.11	8
			$2/m'$	5.4.15	$m'm'2$	7.4.23	m’	4.3.11	8
54.348	$P_{c}cca$	d, ${}^1\overline{E}\,{}^2\overline{E}$	$2/m'$	5.4.15	$2'/m'$	5.5.16	m’	4.3.11	8
			$2/m'$	5.4.15	$m'm'2$	7.4.23	m’	4.3.11	8
54.350	$P_{B}cca$	c, ${}^1\overline{E}\,{}^2\overline{E}$	$2/m'$	5.4.15	$2'2'2$	6.3.19	2	3.1.6	8
			$2/m'$	5.4.15	$m'm'2$	7.4.23	m’	4.3.11	8
54.350	$P_{B}cca$	d, ${}^1\overline{E}\,{}^2\overline{E}$	$2/m'$	5.4.15	$2'2'2$	6.3.19	2	3.1.6	8
			$2/m'$	5.4.15	$m'm'2$	7.4.23	m’	4.3.11	8
54.351	$P_{C}cca$	d,$\overline{AA}$	$\bar{1}'$	2.3.5	$2'2'2$	6.3.19	1	1.1.1	16
			$\bar{1}'$	2.3.5	$\bar{1}$	2.1.3	1	1.1.1	16
			$\bar{1}'$	2.3.5	2	3.1.6	1	1.1.1	16
54.352	$P_{I}cca$	b,$\overline{AA}$	$\bar{1}'$	2.3.5	$\bar{1}$	2.1.3	1	1.1.1	16
			$\bar{1}'$	2.3.5	$2'$	3.3.8	1	1.1.1	16
			$\bar{1}'$	2.3.5	2	3.1.6	1	1.1.1	16
55.360	$P_{a}bam$	a, ${}^1\overline{E}\,{}^2\overline{E}$	$2'/m$	5.3.14	$2/m$	5.1.12	m	4.1.9	8
			$2'/m$	5.3.14	$m'm2'$	7.3.22	m	4.1.9	8
55.360	$P_{a}bam$	b, ${}^1\overline{E}\,{}^2\overline{E}$	$2'/m$	5.3.14	$2/m$	5.1.12	m	4.1.9	8
			$2'/m$	5.3.14	$m'm2'$	7.3.22	m	4.1.9	8
55.361	$P_{c}bam$	b, ${}^1\overline{E}\,{}^2\overline{E}$	$2/m'$	5.4.15	$2/m$	5.1.12	2	3.1.6	8
55.361	$P_{c}bam$	d, ${}^1\overline{E}\,{}^2\overline{E}$	$2/m'$	5.4.15	$2/m$	5.1.12	2	3.1.6	8
55.362	$P_{A}bam$	c,$\overline{AA}$	$\bar{1}'$	2.3.5	$2/m$	5.1.12	1	1.1.1	16
			$\bar{1}'$	2.3.5	$2'$	3.3.8	1	1.1.1	16
55.363	$P_{C}bam$	e, ${}^1\overline{E}\,{}^2\overline{E}$	$2'/m$	5.3.14	$m'm'm$	8.4.27	m	4.1.9	8
55.363	$P_{C}bam$	f, ${}^1\overline{E}\,{}^2\overline{E}$	$2'/m$	5.3.14	$m'm'm$	8.4.27	m	4.1.9	8
55.364	$P_{I}bam$	e,$\overline{AA}$	$\bar{1}'$	2.3.5	$2'2'2$	6.3.19	1	1.1.1	16

			$\bar{1}'$	2.3.5	$2/m$	5.1.12	1	1.1.1	16
56.372	P_bccn	a,$\overline{AA}$	$\bar{1}'$	2.3.5	$\bar{1}$	2.1.3	1	1.1.1	16
			$\bar{1}'$	2.3.5	$2'$	3.3.8	1	1.1.1	16
			$\bar{1}'$	2.3.5	2	3.1.6	1	1.1.1	16
56.373	P_cccn	d,$\overline{AA}$	$\bar{1}'$	2.3.5	$m'm'2$	7.4.23	1	1.1.1	16
			$\bar{1}'$	2.3.5	$\bar{1}$	2.1.3	1	1.1.1	16
56.374	P_Accn	c,$\overline{AA}$	$\bar{1}'$	2.3.5	$2'/m'$	5.5.16	1	1.1.1	16
			$\bar{1}'$	2.3.5	2	3.1.6	1	1.1.1	16
56.375	P_Cccn	c, ${}^1\overline{E}{}^2\overline{E}$	$2/m'$	5.4.15	$2'2'2$	6.3.19	2	3.1.6	8
			$2/m'$	5.4.15	$2'/m'$	5.5.16	m'	4.3.11	8
56.375	P_Cccn	d, ${}^1\overline{E}{}^2\overline{E}$	$2/m'$	5.4.15	$2'2'2$	6.3.19	2	3.1.6	8
			$2/m'$	5.4.15	$2'/m'$	5.5.16	m'	4.3.11	8
56.376	P_Iccn	c, ${}^1\overline{E}{}^2\overline{E}$	$2/m'$	5.4.15	$2'2'2$	6.3.19	2	3.1.6	8
56.376	P_Iccn	d, ${}^1\overline{E}{}^2\overline{E}$	$2/m'$	5.4.15	$2'2'2$	6.3.19	2	3.1.6	8
57.386	P_abcm	b,$\overline{AA}$	$\bar{1}'$	2.3.5	$\bar{1}$	2.1.3	1	1.1.1	16
			$\bar{1}'$	2.3.5	2	3.1.6	1	1.1.1	16
			$\bar{1}'$	2.3.5	m	4.1.9	1	1.1.1	16
57.387	P_bbcm	a, ${}^1\overline{E}{}^2\overline{E}$	$2/m'$	5.4.15	$2'/m'$	5.5.16	m'	4.3.11	8
			$2/m'$	5.4.15	$m'm2'$	7.3.22	m'	4.3.11	8
57.387	P_bbcm	b, ${}^1\overline{E}{}^2\overline{E}$	$2/m'$	5.4.15	$2'/m'$	5.5.16	m'	4.3.11	8
			$2/m'$	5.4.15	$m'm2'$	7.3.22	m'	4.3.11	8
57.388	P_cbcm	a, ${}^1\overline{E}{}^2\overline{E}$	$2'/m$	5.3.14	$2'/m'$	5.5.16	2'	3.3.8	8
			$2'/m$	5.3.14	$m'm2'$	7.3.22	m	4.1.9	8
57.388	P_cbcm	c, ${}^1\overline{E}{}^2\overline{E}$	$2'/m$	5.3.14	$2'/m'$	5.5.16	2'	3.3.8	8
			$2'/m$	5.3.14	$m'm2'$	7.3.22	m	4.1.9	8
57.389	P_Abcm	c, ${}^1\overline{E}{}^2\overline{E}$	$2'/m$	5.3.14	$2'2'2$	6.3.19	2'	3.3.8	8
			$2'/m$	5.3.14	$m'm2'$	7.3.22	m	4.1.9	8
57.389	P_Abcm	d, ${}^1\overline{E}{}^2\overline{E}$	$2'/m$	5.3.14	$2'2'2$	6.3.19	2'	3.3.8	8
			$2'/m$	5.3.14	$m'm2'$	7.3.22	m	4.1.9	8
57.391	P_Cbcm	a, ${}^1\overline{E}{}^2\overline{E}$	$2/m'$	5.4.15	$m'm2'$	7.3.22	m'	4.3.11	8
57.391	P_Cbcm	b, ${}^1\overline{E}{}^2\overline{E}$	$2/m'$	5.4.15	$m'm2'$	7.3.22	m'	4.3.11	8
57.392	P_Ibcm	c, ${}^1\overline{E}{}^2\overline{E}$	$2'/m$	5.3.14	$2'2'2$	6.3.19	2'	3.3.8	8
57.392	P_Ibcm	d, ${}^1\overline{E}{}^2\overline{E}$	$2'/m$	5.3.14	$2'2'2$	6.3.19	2'	3.3.8	8
58.400	P_annm	a, ${}^1\overline{E}{}^2\overline{E}$	$2'/m$	5.3.14	$2/m$	5.1.12	m	4.1.9	8
58.400	P_annm	b, ${}^1\overline{E}{}^2\overline{E}$	$2'/m$	5.3.14	$2/m$	5.1.12	m	4.1.9	8
58.401	P_cnnm	b, ${}^1\overline{E}{}^2\overline{E}$	$2/m'$	5.4.15	$2/m$	5.1.12	2	3.1.6	8
58.401	P_cnnm	d, ${}^1\overline{E}{}^2\overline{E}$	$2/m'$	5.4.15	$2/m$	5.1.12	2	3.1.6	8
58.402	P_Bnnm	d,$\overline{AA}$	$\bar{1}'$	2.3.5	$2/m$	5.1.12	1	1.1.1	16
			$\bar{1}'$	2.3.5	$m'm2'$	7.3.22	1	1.1.1	16
58.403	P_Cnnm	e, ${}^1\overline{E}{}^2\overline{E}$	$2'/m$	5.3.14	$2/m$	5.1.12	m	4.1.9	8
58.403	P_Cnnm	f, ${}^1\overline{E}{}^2\overline{E}$	$2'/m$	5.3.14	$2/m$	5.1.12	m	4.1.9	8
58.404	P_Innm	k,$\overline{AA}$	$\bar{1}'$	2.3.5	$m'm'm$	8.4.27	1	1.1.1	16
59.412	P_bmmn	a, ${}^1\overline{E}{}^2\overline{E}$	$2'/m$	5.3.14	$2'/m'$	5.5.16	2'	3.3.8	8
			$2'/m$	5.3.14	$mm2$	7.1.20	m	4.1.9	8
59.412	P_bmmn	c, ${}^1\overline{E}{}^2\overline{E}$	$2'/m$	5.3.14	$2'/m'$	5.5.16	2'	3.3.8	8
			$2'/m$	5.3.14	$mm2$	7.1.20	m	4.1.9	8
59.412	P_bmmn	e,$\overline{E}$	$mm2$	7.1.20	$2'/m$	5.3.14	m	4.1.9	8
			$mm2$	7.1.20	$m'm2'$	7.3.22	m	4.1.9	8
59.413	P_cmmn	d,$\overline{AA}$	$\bar{1}'$	2.3.5	$mm2$	7.1.20	1	1.1.1	16
			$\bar{1}'$	2.3.5	$\bar{1}$	2.1.3	1	1.1.1	16
60.426	P_abcn	a,$\overline{AA}$	$\bar{1}'$	2.3.5	$\bar{1}$	2.1.3	1	1.1.1	16
			$\bar{1}'$	2.3.5	$2'$	3.3.8	1	1.1.1	16
			$\bar{1}'$	2.3.5	2	3.1.6	1	1.1.1	16
60.427	P_bbcn	b,$\overline{AA}$	$\bar{1}'$	2.3.5	$\bar{1}$	2.1.3	1	1.1.1	16
			$\bar{1}'$	2.3.5	2	3.1.6	1	1.1.1	16
			$\bar{1}'$	2.3.5	m'	4.3.11	1	1.1.1	16
60.428	P_cbcn	a, ${}^1\overline{E}{}^2\overline{E}$	$2/m'$	5.4.15	$2'/m'$	5.5.16	m'	4.3.11	8
60.428	P_cbcn	b, ${}^1\overline{E}{}^2\overline{E}$	$2/m'$	5.4.15	$2'/m'$	5.5.16	m'	4.3.11	8
60.430	P_Bbcn	d,$\overline{AA}$	$\bar{1}'$	2.3.5	$2'2'2$	6.3.19	1	1.1.1	16
			$\bar{1}'$	2.3.5	$\bar{1}$	2.1.3	1	1.1.1	16
			$\bar{1}'$	2.3.5	$2'$	3.3.8	1	1.1.1	16
60.431	P_Cbcn	d,$\overline{AA}$	$\bar{1}'$	2.3.5	$2'/m'$	5.5.16	1	1.1.1	16
			$\bar{1}'$	2.3.5	$m'm'2$	7.4.23	1	1.1.1	16
60.432	P_Ibcn	e,$\overline{AA}$	$\bar{1}'$	2.3.5	$2'2'2$	6.3.19	1	1.1.1	16
			$\bar{1}'$	2.3.5	$2'/m'$	5.5.16	1	1.1.1	16
61.438	P_abca	a,$\overline{AA}$	$\bar{1}'$	2.3.5	$\bar{1}$	2.1.3	1	1.1.1	16
			$\bar{1}'$	2.3.5	$2'$	3.3.8	1	1.1.1	16
			$\bar{1}'$	2.3.5	m'	4.3.11	1	1.1.1	16
61.439	P_Cbca	c,$\overline{AA}$	$\bar{1}'$	2.3.5	$2'/m'$	5.5.16	1	1.1.1	16
			$\bar{1}'$	2.3.5	$2'$	3.3.8	1	1.1.1	16
61.440	P_Ibca	a,$\overline{AA}$	$\bar{1}'$	2.3.5	$\bar{1}$	2.1.3	1	1.1.1	16
			$\bar{1}'$	2.3.5	$2'$	3.3.8	1	1.1.1	16

62.450	P_anma	c,$\overline{A}\overline{A}$	$\bar{1}'$	2.3.5	$m'm2'$	7.3.22	1	1.1.1	16
			$\bar{1}'$	2.3.5	$\bar{1}$	2.1.3	1	1.1.1	16
62.451	P_bnma	a, ${}^1\overline{E}{}^2\overline{E}$	$2'/m$	5.3.14	$2'/m'$	5.5.16	2'	3.3.8	8
62.451	P_bnma	c, ${}^1\overline{E}{}^2\overline{E}$	$2'/m$	5.3.14	$2'/m'$	5.5.16	2'	3.3.8	8
62.452	P_cnma	a,$\overline{A}\overline{A}$	$\bar{1}'$	2.3.5	$\bar{1}$	2.1.3	1	1.1.1	16
			$\bar{1}'$	2.3.5	$2'$	3.3.8	1	1.1.1	16
			$\bar{1}'$	2.3.5	m	4.1.9	1	1.1.1	16
62.453	P_Anma	a, ${}^1\overline{E}{}^2\overline{E}$	$2'/m$	5.3.14	$m'm2'$	7.3.22	m	4.1.9	8
62.453	P_Anma	b, ${}^1\overline{E}{}^2\overline{E}$	$2'/m$	5.3.14	$m'm2'$	7.3.22	m	4.1.9	8
62.454	P_Bnma	d,$\overline{A}\overline{A}$	$\bar{1}'$	2.3.5	$2'/m'$	5.5.16	1	1.1.1	16
			$\bar{1}'$	2.3.5	$m'm2'$	7.3.22	1	1.1.1	16
62.456	P_Inma	c, ${}^1\overline{E}{}^2\overline{E}$	$2'/m$	5.3.14	$m'm2'$	7.3.22	m	4.1.9	8
62.456	P_Inma	d, ${}^1\overline{E}{}^2\overline{E}$	$2'/m$	5.3.14	$m'm2'$	7.3.22	m	4.1.9	8
63.466	C_cmcm	c,$\overline{E}$	$m'mm$	8.3.26	$m'm'm$	8.4.27	m'm2'	7.3.22	4
63.466	C_cmcm	d,$\overline{E}$	$m'mm$	8.3.26	$m'm'm$	8.4.27	m'm2'	7.3.22	4
63.466	C_cmcm	f, ${}^1\overline{E}{}^2\overline{E}$	$2'/m$	5.3.14	$m'mm$	8.3.26	m	4.1.9	8
			$2'/m$	5.3.14	$2'/m'$	5.5.16	2'	3.3.8	8
63.467	C_amcm	b, ${}^1\overline{E}{}^2\overline{E}$	$2/m'$	5.4.15	$2/m$	5.1.12	2	3.1.6	8
			$2/m'$	5.4.15	$2'/m'$	5.5.16	m'	4.3.11	8
			$2/m'$	5.4.15	$m'm2'$	7.3.22	m'	4.3.11	8
63.467	C_amcm	c, ${}^1\overline{E}{}^2\overline{E}$	$2'/m$	5.3.14	$2/m$	5.1.12	m	4.1.9	8
			$2'/m$	5.3.14	$2'/m'$	5.5.16	2'	3.3.8	8
			$2'/m$	5.3.14	$mm2$	7.1.20	m	4.1.9	8
63.467	C_amcm	e,$\overline{E}$	$mm2$	7.1.20	$2/m$	5.1.12	m	4.1.9	8
			$mm2$	7.1.20	$2'/m$	5.3.14	m	4.1.9	8
			$mm2$	7.1.20	$m'm2'$	7.3.22	m	4.1.9	8
63.468	C_Amcm	e, ${}^1\overline{E}{}^2\overline{E}$	$2'/m$	5.3.14	$m'mm$	8.3.26	m	4.1.9	8
			$2'/m$	5.3.14	$2'2'2$	6.3.19	2'	3.3.8	8
64.478	C_cmca	c, ${}^1\overline{E}{}^2\overline{E}$	$2/m'$	5.4.15	$2'2'2$	6.3.19	2	3.1.6	8
			$2/m'$	5.4.15	$2'/m'$	5.5.16	m'	4.3.11	8
			$2/m'$	5.4.15	$m'm2'$	7.3.22	m'	4.3.11	8
64.478	C_cmca	e, ${}^1\overline{E}{}^2\overline{E}$	$2'/m$	5.3.14	$2'2'2$	6.3.19	2'	3.3.8	8
			$2'/m$	5.3.14	$2/m$	5.1.12	m	4.1.9	8
			$2'/m$	5.3.14	$m'm2'$	7.3.22	m	4.1.9	8
64.479	C_amca	a, ${}^1\overline{E}{}^2\overline{E}$	$2'/m$	5.3.14	$2'/m'$	5.5.16	2'	3.3.8	8
			$2'/m$	5.3.14	$2/m$	5.1.12	m	4.1.9	8
			$2'/m$	5.3.14	$m'm2'$	7.3.22	m	4.1.9	8
64.479	C_amca	d, ${}^1\overline{E}{}^2\overline{E}$	$2/m'$	5.4.15	$2'/m'$	5.5.16	m'	4.3.11	8
			$2/m'$	5.4.15	$2/m$	5.1.12	2	3.1.6	8
			$2/m'$	5.4.15	$m'm'2$	7.4.23	m'	4.3.11	8
64.480	C_Amca	c, ${}^1\overline{E}{}^2\overline{E}$	$2'/m$	5.3.14	$m'm'm$	8.4.27	m	4.1.9	8
			$2'/m$	5.3.14	$2'2'2$	6.3.19	2'	3.3.8	8
64.480	C_Amca	d, ${}^1\overline{E}{}^2\overline{E}$	$2/m'$	5.4.15	$m'm'm$	8.4.27	m'	4.3.11	8
			$2/m'$	5.4.15	$2'2'2$	6.3.19	2	3.1.6	8
65.488	C_cmmm	f, ${}^1\overline{E}{}^2\overline{E}$	$2/m'$	5.4.15	$m'mm$	8.3.26	m'	4.3.11	8
			$2/m'$	5.4.15	$2/m$	5.1.12	2	3.1.6	8
65.489	C_ammm	b,$\overline{E}$	$m'mm$	8.3.26	$m'm'm$	8.4.27	m'm2'	7.3.22	4
65.489	C_ammm	d,$\overline{E}$	$m'mm$	8.3.26	$m'm'm$	8.4.27	m'm2'	7.3.22	4
65.489	C_ammm	e,$\overline{E}$	$m'mm$	8.3.26	$m'm'm$	8.4.27	m'm2'	7.3.22	4
65.489	C_ammm	g,$\overline{E}$	$m'mm$	8.3.26	$m'm'm$	8.4.27	m'm2'	7.3.22	4
65.490	C_Ammm	c, ${}^1\overline{E}{}^2\overline{E}$	$2'/m$	5.3.14	mmm	8.1.24	m	4.1.9	8
			$2'/m$	5.3.14	$2'2'2$	6.3.19	2'	3.3.8	8
65.490	C_Ammm	d, ${}^1\overline{E}{}^2\overline{E}$	$2'/m$	5.3.14	mmm	8.1.24	m	4.1.9	8
			$2'/m$	5.3.14	$2'2'2$	6.3.19	2'	3.3.8	8
66.498	C_cccm	c,$\overline{E}$	$m'm'm'$	8.5.28	$m'm'm$	8.4.27	m'm'2	7.4.23	4
66.498	C_cccm	d,$\overline{E}$	$m'm'm'$	8.5.28	$m'm'm$	8.4.27	m'm'2	7.4.23	4
66.498	C_cccm	f, ${}^1\overline{E}{}^2\overline{E}$	$2/m'$	5.4.15	$m'm'm'$	8.5.28	m'	4.3.11	8
			$2/m'$	5.4.15	$2/m$	5.1.12	2	3.1.6	8
66.499	C_accm	c, ${}^1\overline{E}{}^2\overline{E}$	$2'/m$	5.3.14	$2/m$	5.1.12	m	4.1.9	8
			$2'/m$	5.3.14	$2'2'2$	6.3.19	2'	3.3.8	8
66.499	C_accm	d, ${}^1\overline{E}{}^2\overline{E}$	$2'/m$	5.3.14	$2/m$	5.1.12	m	4.1.9	8
			$2'/m$	5.3.14	$2'2'2$	6.3.19	2'	3.3.8	8
66.499	C_accm	e,$\overline{E}$	222	6.1.17	$2/m$	5.1.12	2	3.1.6	8
			222	6.1.17	$2'2'2$	6.3.19	2	3.1.6	8
66.500	C_Accm	c, ${}^1\overline{E}{}^2\overline{E}$	$2/m'$	5.4.15	$m'm'm$	8.4.27	m'	4.3.11	8
			$2/m'$	5.4.15	222	6.1.17	2	3.1.6	8
66.500	C_Accm	d, ${}^1\overline{E}{}^2\overline{E}$	$2/m'$	5.4.15	$m'm'm$	8.4.27	m'	4.3.11	8
			$2/m'$	5.4.15	222	6.1.17	2	3.1.6	8
66.500	C_Accm	f,$\overline{E}$	222	6.1.17	$2/m'$	5.4.15	2	3.1.6	8

			222	6.1.17	$2/m$	5.1.12	2	3.1.6	8
67.508	C_cmma	a,$\overline{E}$	222	6.1.17	$2'2'2$	6.3.19	2	3.1.6	8
			222	6.1.17	$2/m$	5.1.12	2	3.1.6	8
67.508	C_cmma	d,$^1\overline{E}\,^2\overline{E}$	$2'/m$	5.3.14	$2'2'2$	6.3.19	2'	3.3.8	8
			$2'/m$	5.3.14	$2/m$	5.1.12	m	4.1.9	8
			$2'/m$	5.3.14	$mm2$	7.1.20	m	4.1.9	8
67.508	C_cmma	f,$^1\overline{E}\,^2\overline{E}$	$2'/m$	5.3.14	$2'2'2$	6.3.19	2'	3.3.8	8
			$2'/m$	5.3.14	$2/m$	5.1.12	m	4.1.9	8
			$2'/m$	5.3.14	$mm2$	7.1.20	m	4.1.9	8
67.508	C_cmma	g,$\overline{E}$	$mm2$	7.1.20	$2/m$	5.1.12	m	4.1.9	8
			$mm2$	7.1.20	$2'/m$	5.3.14	m	4.1.9	8
67.509	C_amma	a,$\overline{E}$	$m'mm$	8.3.26	$m'm'm$	8.4.27	m'm2'	7.3.22	4
67.509	C_amma	c,$\overline{E}$	$m'mm$	8.3.26	$m'm'm$	8.4.27	m'm2'	7.3.22	4
67.509	C_amma	f,$\overline{E}$	$m'm'm'$	8.5.28	$m'm'm$	8.4.27	m'm'2	7.4.23	4
67.509	C_amma	h,$\overline{E}$	$m'm'm'$	8.5.28	$m'm'm$	8.4.27	m'm'2	7.4.23	4
67.510	C_Amma	e,$^1\overline{E}\,^2\overline{E}$	$2/m'$	5.4.15	$m'mm$	8.3.26	m'	4.3.11	8
			$2/m'$	5.4.15	222	6.1.17	2	3.1.6	8
67.510	C_Amma	f,$\overline{E}$	222	6.1.17	$2/m$	5.1.12	2	3.1.6	8
			222	6.1.17	$2/m'$	5.4.15	2	3.1.6	8
68.518	C_ccca	b,$\overline{E}$	222	6.1.17	$2'2'2$	6.3.19	2	3.1.6	8
			222	6.1.17	$2/m'$	5.4.15	2	3.1.6	8
68.518	C_ccca	d,$^1\overline{E}\,^2\overline{E}$	$2/m'$	5.4.15	222	6.1.17	2	3.1.6	8
			$2/m'$	5.4.15	$2'/m'$	5.5.16	m'	4.3.11	8
			$2/m'$	5.4.15	$m'm'2$	7.4.23	m'	4.3.11	8
68.518	C_ccca	f,$^1\overline{E}\,^2\overline{E}$	$2/m'$	5.4.15	222	6.1.17	2	3.1.6	8
			$2/m'$	5.4.15	$2'/m'$	5.5.16	m'	4.3.11	8
			$2/m'$	5.4.15	$m'm'2$	7.4.23	m'	4.3.11	8
68.519	C_acca	a,$^1\overline{E}\,^2\overline{E}$	$2/m'$	5.4.15	$2'/m'$	5.5.16	m'	4.3.11	8
			$2/m'$	5.4.15	$2'2'2$	6.3.19	2	3.1.6	8
68.519	C_acca	b,$^1\overline{E}\,^2\overline{E}$	$2/m'$	5.4.15	$2'/m'$	5.5.16	m'	4.3.11	8
			$2/m'$	5.4.15	222	6.1.17	2	3.1.6	8
68.519	C_acca	h,$\overline{E}$	222	6.1.17	$2/m'$	5.4.15	2	3.1.6	8
			222	6.1.17	$2'2'2$	6.3.19	2	3.1.6	8
68.520	C_Acca	e,$^1\overline{E}\,^2\overline{E}$	$2/m'$	5.4.15	$m'm'm'$	8.5.28	m'	4.3.11	8
			$2/m'$	5.4.15	$2'2'2$	6.3.19	2	3.1.6	8
69.526	F_Smmm	b,$\overline{E}$	$m'mm$	8.3.26	$m'm'm$	8.4.27	m'm2'	7.3.22	4
69.526	F_Smmm	c,$\overline{E}$	$m'mm$	8.3.26	$m'm'm$	8.4.27	m'm2'	7.3.22	4
69.526	F_Smmm	e,$\overline{E}$	$m'mm$	8.3.26	$m'm'm$	8.4.27	m'm2'	7.3.22	4
69.526	F_Smmm	h,$\overline{E}$	$m'm'm'$	8.5.28	$m'm'm$	8.4.27	m'm'2	7.4.23	4
70.532	F_Sddd	a,$\overline{E}$	222	6.1.17	$2'2'2$	6.3.19	2	3.1.6	8
70.532	F_Sddd	f,$\overline{A}\overline{A}$	$\bar{1}'$	2.3.5	222	6.1.17	1	1.1.1	16
			$\bar{1}'$	2.3.5	$2'2'2$	6.3.19	1	1.1.1	16
			$\bar{1}'$	2.3.5	$\bar{1}$	2.1.3	1	1.1.1	16
71.538	I_cmmm	e,$^1\overline{E}\,^2\overline{E}$	$2'/m$	5.3.14	mmm	8.1.24	m	4.1.9	8
			$2'/m$	5.3.14	$2'/m'$	5.5.16	2'	3.3.8	8
72.546	I_cbam	c,$\overline{E}$	$m'm'm'$	8.5.28	$m'm'm$	8.4.27	m'm'2	7.4.23	4
72.546	I_cbam	d,$\overline{E}$	$m'm'm'$	8.5.28	$m'm'm$	8.4.27	m'm'2	7.4.23	4
72.546	I_cbam	e,$^1\overline{E}\,^2\overline{E}$	$2'/m$	5.3.14	$m'm'm$	8.4.27	m	4.1.9	8
			$2'/m$	5.3.14	$2'/m'$	5.5.16	2'	3.3.8	8
72.547	I_bbam	a,$\overline{E}$	222	6.1.17	$2'2'2$	6.3.19	2	3.1.6	8
			222	6.1.17	$2/m$	5.1.12	2	3.1.6	8
			222	6.1.17	$2/m'$	5.4.15	2	3.1.6	8
72.547	I_bbam	d,$^1\overline{E}\,^2\overline{E}$	$2'/m$	5.3.14	$2'2'2$	6.3.19	2'	3.3.8	8
			$2'/m$	5.3.14	$2/m$	5.1.12	m	4.1.9	8
			$2'/m$	5.3.14	$m'm2'$	7.3.22	m	4.1.9	8
72.547	I_bbam	e,$^1\overline{E}\,^2\overline{E}$	$2/m'$	5.4.15	222	6.1.17	2	3.1.6	8
			$2/m'$	5.4.15	$2'/m'$	5.5.16	m'	4.3.11	8
			$2/m'$	5.4.15	$m'm2'$	7.3.22	m'	4.3.11	8
73.553	I_cbca	d,$^1\overline{E}\,^2\overline{E}$	$2/m'$	5.4.15	$2'2'2$	6.3.19	2	3.1.6	8
			$2/m'$	5.4.15	$2'/m'$	5.5.16	m'	4.3.11	8
			$2/m'$	5.4.15	$m'm'2$	7.4.23	m'	4.3.11	8
73.553	I_cbca	e,$^1\overline{E}\,^2\overline{E}$	$2/m'$	5.4.15	$2'2'2$	6.3.19	2	3.1.6	8
			$2/m'$	5.4.15	$2'/m'$	5.5.16	m'	4.3.11	8
			$2/m'$	5.4.15	$m'm'2$	7.4.23	m'	4.3.11	8
74.561	I_cmma	c,$^1\overline{E}\,^2\overline{E}$	$2'/m$	5.3.14	$2'2'2$	6.3.19	2'	3.3.8	8
			$2'/m$	5.3.14	$2/m$	5.1.12	m	4.1.9	8
			$2'/m$	5.3.14	$mm2$	7.1.20	m	4.1.9	8
74.561	I_cmma	f,$^1\overline{E}\,^2\overline{E}$	$2'/m$	5.3.14	$2'2'2$	6.3.19	2'	3.3.8	8
			$2'/m$	5.3.14	$2/m$	5.1.12	m	4.1.9	8

			$2'/m$	5.3.14	$mm2$	7.1.20	m	4.1.9	8
74.561	I_cmma	g,$\overline{E}$	$mm2$	7.1.20	$2'/m$	5.3.14	m	4.1.9	8
			$mm2$	7.1.20	$2/m$	5.1.12	m	4.1.9	8
74.562	I_bmma	a,$\overline{E}$	$m'mm$	8.3.26	$m'm'm$	8.4.27	m'm2'	7.3.22	4
74.562	I_bmma	b,$\overline{E}$	$m'mm$	8.3.26	$m'm'm$	8.4.27	m'm2'	7.3.22	4
74.562	I_bmma	f, ${}^1\overline{E}\,{}^2\overline{E}$	$2/m'$	5.4.15	$m'm'm$	8.4.27	m'	4.3.11	8
			$2/m'$	5.4.15	$2/m$	5.1.12	2	3.1.6	8
81.36	$P_c\bar{4}$	b, ${}^1\overline{E}\,{}^2\overline{E}$	$\bar{4}'$	10.3.34	$\bar{4}$	10.1.32	2	3.1.6	4
81.36	$P_c\bar{4}$	d, ${}^1\overline{E}\,{}^2\overline{E}$	$\bar{4}'$	10.3.34	$\bar{4}$	10.1.32	2	3.1.6	4
82.42	$I_c\bar{4}$	b, ${}^1\overline{E}\,{}^2\overline{E}$	$\bar{4}'$	10.3.34	$\bar{4}$	10.1.32	2	3.1.6	4
82.42	$I_c\bar{4}$	c, ${}^1\overline{E}\,{}^2\overline{E}$	$\bar{4}'$	10.3.34	$\bar{4}$	10.1.32	2	3.1.6	4
83.48	P_c4/m	b, ${}^1\overline{E}_2\,{}^2\overline{E}_2$	$4/m'$	11.4.38	$4/m$	11.1.35	4	9.1.29	4
83.48	P_c4/m	b, ${}^1\overline{E}_1\,{}^2\overline{E}_1$	$4/m'$	11.4.38	$4/m$	11.1.35	4	9.1.29	4
83.48	P_c4/m	d, ${}^1\overline{E}_2\,{}^2\overline{E}_2$	$4/m'$	11.4.38	$4/m$	11.1.35	4	9.1.29	4
83.48	P_c4/m	d, ${}^1\overline{E}_1\,{}^2\overline{E}_1$	$4/m'$	11.4.38	$4/m$	11.1.35	4	9.1.29	4
83.48	P_c4/m	f, ${}^1\overline{E}\,{}^2\overline{E}$	$2/m'$	5.4.15	$4/m'$	11.4.38	m'	4.3.11	8
			$2/m'$	5.4.15	$2/m$	5.1.12	2	3.1.6	8
83.49	P_C4/m	e, ${}^1\overline{E}\,{}^2\overline{E}$	$2'/m$	5.3.14	$4/m$	11.1.35	m	4.1.9	8
			$2'/m$	5.3.14	$4'/m$	11.3.37	m	4.1.9	8
83.49	P_C4/m	f, ${}^1\overline{E}\,{}^2\overline{E}$	$2'/m$	5.3.14	$4/m$	11.1.35	m	4.1.9	8
			$2'/m$	5.3.14	$4'/m$	11.3.37	m	4.1.9	8
83.50	P_I4/m	d, ${}^1\overline{E}\,{}^2\overline{E}$	$\bar{4}'$	10.3.34	$2/m$	5.1.12	2	3.1.6	8
83.50	P_I4/m	f,$\overline{AA}$	$\bar{1}'$	2.3.5	$4/m$	11.1.35	1	1.1.1	16
			$\bar{1}'$	2.3.5	$2/m$	5.1.12	1	1.1.1	16
			$\bar{1}'$	2.3.5	$\bar{4}'$	10.3.34	1	1.1.1	16
84.56	P_c4_2/m	f, ${}^1\overline{E}\,{}^2\overline{E}$	$2/m'$	5.4.15	$4'/m'$	11.5.39	m'	4.3.11	8
			$2/m'$	5.4.15	$2/m$	5.1.12	2	3.1.6	8
84.57	P_C4_2/m	c, ${}^1\overline{E}\,{}^2\overline{E}$	$2'/m$	5.3.14	$2/m$	5.1.12	m	4.1.9	8
84.57	P_C4_2/m	d, ${}^1\overline{E}\,{}^2\overline{E}$	$2'/m$	5.3.14	$2/m$	5.1.12	m	4.1.9	8
84.57	P_C4_2/m	f, ${}^1\overline{E}\,{}^2\overline{E}$	$\bar{4}'$	10.3.34	$2/m$	5.1.12	2	3.1.6	8
84.58	P_I4_2/m	f,$\overline{AA}$	$\bar{1}'$	2.3.5	$4'/m$	11.3.37	1	1.1.1	16
			$\bar{1}'$	2.3.5	$2/m$	5.1.12	1	1.1.1	16
			$\bar{1}'$	2.3.5	$\bar{4}$	10.1.32	1	1.1.1	16
85.64	P_c4/n	b, ${}^1\overline{E}\,{}^2\overline{E}$	$\bar{4}'$	10.3.34	$\bar{4}$	10.1.32	2	3.1.6	8
85.64	P_c4/n	e,$\overline{AA}$	$\bar{1}'$	2.3.5	$\bar{4}$	10.1.32	1	1.1.1	16
			$\bar{1}'$	2.3.5	$\bar{4}'$	10.3.34	1	1.1.1	16
			$\bar{1}'$	2.3.5	4	9.1.29	1	1.1.1	16
			$\bar{1}'$	2.3.5	1	2.1.3	1	1.1.1	16
85.66	P_I4/n	c, ${}^1\overline{E}\,{}^2\overline{E}$	$2/m'$	5.4.15	$4/m'$	11.4.38	m'	4.3.11	8
			$2/m'$	5.4.15	$\bar{4}$	10.1.32	2	3.1.6	8
86.72	P_c4_2/n	a, ${}^1\overline{E}\,{}^2\overline{E}$	$\bar{4}'$	10.3.34	$\bar{4}$	10.1.32	2	3.1.6	8
86.72	P_c4_2/n	e,$\overline{AA}$	$\bar{1}'$	2.3.5	$\bar{4}'$	10.3.34	1	1.1.1	16
			$\bar{1}'$	2.3.5	$\bar{4}$	10.1.32	1	1.1.1	16
			$\bar{1}'$	2.3.5	$4'$	9.3.31	1	1.1.1	16
			$\bar{1}'$	2.3.5	1	2.1.3	1	1.1.1	16
86.73	P_C4_2/n	a, ${}^1\overline{E}\,{}^2\overline{E}$	$2/m'$	5.4.15	$2'/m'$	5.5.16	m'	4.3.11	8
			$2/m'$	5.4.15	$\bar{4}'$	10.3.34	2	3.1.6	8
86.73	P_C4_2/n	b, ${}^1\overline{E}\,{}^2\overline{E}$	$2/m'$	5.4.15	$2'/m'$	5.5.16	m'	4.3.11	8
			$2/m'$	5.4.15	$\bar{4}$	10.1.32	2	3.1.6	8
86.73	P_C4_2/n	e, ${}^1\overline{E}\,{}^2\overline{E}$	$\bar{4}'$	10.3.34	$2/m'$	5.4.15	2	3.1.6	8
86.74	P_I4_2/n	c, ${}^1\overline{E}\,{}^2\overline{E}$	$2/m'$	5.4.15	$4'/m'$	11.5.39	m'	4.3.11	8
			$2/m'$	5.4.15	$\bar{4}'$	10.3.34	2	3.1.6	8
86.74	P_I4_2/n	d, ${}^1\overline{E}\,{}^2\overline{E}$	$\bar{4}'$	10.3.34	$2/m'$	5.4.15	2	3.1.6	8
87.80	I_c4/m	b, ${}^1\overline{E}_2\,{}^2\overline{E}_2$	$4/m'$	11.4.38	$4/m$	11.1.35	4	9.1.29	4
87.80	I_c4/m	b, ${}^1\overline{E}_1\,{}^2\overline{E}_1$	$4/m'$	11.4.38	$4/m$	11.1.35	4	9.1.29	4
87.80	I_c4/m	e, ${}^1\overline{E}\,{}^2\overline{E}$	$2'/m$	5.3.14	$4/m$	11.1.35	m	4.1.9	8
			$2'/m$	5.3.14	$4'/m$	11.3.37	m	4.1.9	8
			$2'/m$	5.3.14	$2'/m'$	5.5.16	2'	3.3.8	8
88.86	I_c4_1/a	b, ${}^1\overline{E}\,{}^2\overline{E}$	$\bar{4}'$	10.3.34	$\bar{4}$	10.1.32	2	3.1.6	8
88.86	I_c4_1/a	d,$\overline{AA}$	$\bar{1}'$	2.3.5	$\bar{4}$	10.1.32	1	1.1.1	16
			$\bar{1}'$	2.3.5	$\bar{4}'$	10.3.34	1	1.1.1	16
			$\bar{1}'$	2.3.5	1	2.1.3	1	1.1.1	16
			$\bar{1}'$	2.3.5	$2'$	3.3.8	1	1.1.1	16
89.92	P_c422	a,$\overline{E}_2$	422	12.1.40	$42'2'$	12.4.43	4	9.1.29	4
89.92	P_c422	a,$\overline{E}_1$	422	12.1.40	$42'2'$	12.4.43	4	9.1.29	4
89.92	P_c422	c,$\overline{E}_2$	422	12.1.40	$42'2'$	12.4.43	4	9.1.29	4
89.92	P_c422	c,$\overline{E}_1$	422	12.1.40	$42'2'$	12.4.43	4	9.1.29	4
89.92	P_c422	e,$\overline{E}$	222	6.1.17	422	12.1.40	2	3.1.6	8
			222	6.1.17	$2'2'2$	6.3.19	2	3.1.6	8
89.94	P_I422	c,$\overline{E}$	222	6.1.17	422	12.1.40	2	3.1.6	8

			222	6.1.17	$2'2'2$	6.3.19	2	3.1.6	8
90.100	P_c42_12	a,$\overline{E}$	222	6.1.17	$2'2'2$	6.3.19	2	3.1.6	8
90.102	P_I42_12	d,$\overline{E}$	222	6.1.17	$2'2'2$	6.3.19	2	3.1.6	8
93.124	P_c4_222	e,$\overline{E}$	222	6.1.17	$4'22'$	12.3.42	2	3.1.6	8
			222	6.1.17	$2'2'2$	6.3.19	2	3.1.6	8
93.125	P_C4_222	a,$\overline{E}$	222	6.1.17	$2'2'2$	6.3.19	2	3.1.6	8
93.125	P_C4_222	f,$\overline{E}$	222	6.1.17	$2'2'2$	6.3.19	2	3.1.6	8
93.126	P_I4_222	c,$\overline{E}$	222	6.1.17	$4'22'$	12.3.42	2	3.1.6	8
94.132	$P_c4_22_12$	a,$\overline{E}$	222	6.1.17	$2'2'2$	6.3.19	2	3.1.6	8
94.133	$P_C4_22_12$	a,$\overline{E}$	222	6.1.17	$2'2'2$	6.3.19	2	3.1.6	8
97.156	I_c422	a,$\overline{E}_2$	422	12.1.40	$42'2'$	12.4.43	4	9.1.29	4
97.156	I_c422	a,$\overline{E}_1$	422	12.1.40	$42'2'$	12.4.43	4	9.1.29	4
98.162	I_c4_122	d,$\overline{E}$	222	6.1.17	$2'2'2$	6.3.19	2	3.1.6	8
99.168	P_c4mm	c,$\overline{E}$	$mm2$	7.1.20	$4mm$	13.1.44	m	4.1.9	8
99.170	P_I4mm	b,$\overline{E}$	$mm2$	7.1.20	$4mm$	13.1.44	m	4.1.9	8
105.216	P_c4_2mc	c,$\overline{E}$	$mm2$	7.1.20	$4'm'm$	13.3.46	m	4.1.9	8
105.218	P_I4_2mc	b,$\overline{E}$	$mm2$	7.1.20	$4'm'm$	13.3.46	m	4.1.9	8
111.256	$P_c\bar{4}2m$	e,$\overline{E}$	222	6.1.17	$\bar{4}2m$	14.1.48	2	3.1.6	8
			222	6.1.17	$2'2'2$	6.3.19	2	3.1.6	8
111.258	$P_I\bar{4}2m$	c,$\overline{E}$	222	6.1.17	$\bar{4}2m$	14.1.48	2	3.1.6	8
			222	6.1.17	$\bar{4}'$	10.3.34	2	3.1.6	8
111.258	$P_I\bar{4}2m$	d, ${}^1\overline{E}\,{}^2\overline{E}$	$\bar{4}'$	10.3.34	222	6.1.17	2	3.1.6	8
112.264	$P_c\bar{4}2c$	b,$\overline{E}$	$\bar{4}'2m'$	14.4.51	$\bar{4}2'm'$	14.5.52	m'm'2	7.4.23	4
112.264	$P_c\bar{4}2c$	c,$\overline{E}$	$\bar{4}'2m'$	14.4.51	$\bar{4}2'm'$	14.5.52	m'm'2	7.4.23	4
112.264	$P_c\bar{4}2c$	f,$\overline{E}$	222	6.1.17	$\bar{4}'2m'$	14.4.51	2	3.1.6	8
			222	6.1.17	$2'2'2$	6.3.19	2	3.1.6	8
112.265	$P_C\bar{4}2c$	a,$\overline{E}$	222	6.1.17	$\bar{4}$	10.1.32	2	3.1.6	8
112.265	$P_C\bar{4}2c$	b,$\overline{E}$	222	6.1.17	$\bar{4}'$	10.3.34	2	3.1.6	8
112.265	$P_C\bar{4}2c$	d, ${}^1\overline{E}\,{}^2\overline{E}$	$\bar{4}'$	10.3.34	222	6.1.17	2	3.1.6	8
112.266	$P_I\bar{4}2c$	c,$\overline{E}$	222	6.1.17	$\bar{4}'2m'$	14.4.51	2	3.1.6	8
			222	6.1.17	$\bar{4}$	10.1.32	2	3.1.6	8
113.272	$P_c\bar{4}2_1m$	b, ${}^1\overline{E}\,{}^2\overline{E}$	$\bar{4}'$	10.3.34	$\bar{4}$	10.1.32	2	3.1.6	8
114.280	$P_c\bar{4}2_1c$	a, ${}^1\overline{E}\,{}^2\overline{E}$	$\bar{4}'$	10.3.34	$\bar{4}$	10.1.32	2	3.1.6	8
114.281	$P_C\bar{4}2_1c$	c, ${}^1\overline{E}\,{}^2\overline{E}$	$\bar{4}'$	10.3.34	$2'2'2$	6.3.19	2	3.1.6	8
114.282	$P_I\bar{4}2_1c$	d, ${}^1\overline{E}\,{}^2\overline{E}$	$\bar{4}'$	10.3.34	$2'2'2$	6.3.19	2	3.1.6	8
115.288	$P_c\bar{4}m2$	g,$\overline{E}$	$mm2$	7.1.20	$\bar{4}2m$	14.1.48	m	4.1.9	8
			$mm2$	7.1.20	$\bar{4}'2'm$	14.3.50	m	4.1.9	8
116.296	$P_c\bar{4}c2$	a,$\overline{E}$	$\bar{4}'2m'$	14.4.51	$\bar{4}2'm'$	14.5.52	m'm'2	7.4.23	4
116.296	$P_c\bar{4}c2$	b,$\overline{E}$	$\bar{4}'2m'$	14.4.51	$\bar{4}2'm'$	14.5.52	m'm'2	7.4.23	4
116.297	$P_C\bar{4}c2$	a,$\overline{E}$	222	6.1.17	$2'2'2$	6.3.19	2	3.1.6	8
			222	6.1.17	$\bar{4}$	10.1.32	2	3.1.6	8
116.297	$P_C\bar{4}c2$	f, ${}^1\overline{E}\,{}^2\overline{E}$	$\bar{4}'$	10.3.34	$2'2'2$	6.3.19	2	3.1.6	8
116.298	$P_I\bar{4}c2$	a,$\overline{E}$	222	6.1.17	$\bar{4}$	10.1.32	2	3.1.6	8
116.298	$P_I\bar{4}c2$	c, ${}^1\overline{E}\,{}^2\overline{E}$	$\bar{4}'$	10.3.34	$2'2'2$	6.3.19	2	3.1.6	8
117.304	$P_c\bar{4}b2$	b, ${}^1\overline{E}\,{}^2\overline{E}$	$\bar{4}'$	10.3.34	$\bar{4}$	10.1.32	2	3.1.6	8
117.304	$P_c\bar{4}b2$	c,$\overline{E}$	222	6.1.17	$2'2'2$	6.3.19	2	3.1.6	8
118.312	$P_c\bar{4}n2$	a, ${}^1\overline{E}\,{}^2\overline{E}$	$\bar{4}'$	10.3.34	$\bar{4}$	10.1.32	2	3.1.6	8
118.312	$P_c\bar{4}n2$	c,$\overline{E}$	222	6.1.17	$2'2'2$	6.3.19	2	3.1.6	8
118.313	$P_C\bar{4}n2$	a,$\overline{E}$	222	6.1.17	$2'2'2$	6.3.19	2	3.1.6	8
			222	6.1.17	$\bar{4}'$	10.3.34	2	3.1.6	8
118.313	$P_C\bar{4}n2$	e, ${}^1\overline{E}\,{}^2\overline{E}$	$\bar{4}'$	10.3.34	222	6.1.17	2	3.1.6	8
120.326	$I_c\bar{4}c2$	a,$\overline{E}$	$\bar{4}'2m'$	14.4.51	$\bar{4}2'm'$	14.5.52	m'm'2	7.4.23	4
120.326	$I_c\bar{4}c2$	b,$\overline{E}$	$\bar{4}'2m'$	14.4.51	$\bar{4}2'm'$	14.5.52	m'm'2	7.4.23	4
121.332	$I_c\bar{4}2m$	b,$\overline{E}$	$\bar{4}'2m'$	14.4.51	$\bar{4}2'm'$	14.5.52	m'm'2	7.4.23	4
122.338	$I_c\bar{4}2d$	b, ${}^1\overline{E}\,{}^2\overline{E}$	$\bar{4}'$	10.3.34	$\bar{4}$	10.1.32	2	3.1.6	8
123.348	P_c4/mmm	e,$\overline{E}$	$m'mm$	8.3.26	$4/m'mm$	15.3.55	m'm2'	7.3.22	8
123.349	P_C4/mmm	e,$\overline{E}$	$m'mm$	8.3.26	$4'/mm'm$	15.4.56	m'm2'	7.3.22	8
123.349	P_C4/mmm	f,$\overline{E}$	$m'mm$	8.3.26	$4'/mm'm$	15.4.56	m'm2'	7.3.22	8
123.350	P_I4/mmm	f, ${}^1\overline{E}\,{}^2\overline{E}$	$2'/m$	5.3.14	$4/mmm$	15.1.53	m	4.1.9	16
			$2'/m$	5.3.14	$\bar{4}'2'm$	14.3.50	2'	3.3.8	16
124.360	P_c4/mcc	b,$\overline{E}_2$	$4/m'm'm'$	15.7.59	$4/mm'm'$	15.6.58	4m'm'	13.4.47	4
124.360	P_c4/mcc	b,$\overline{E}_1$	$4/m'm'm'$	15.7.59	$4/mm'm'$	15.6.58	4m'm'	13.4.47	4
124.360	P_c4/mcc	d,$\overline{E}_2$	$4/m'm'm'$	15.7.59	$4/mm'm'$	15.6.58	4m'm'	13.4.47	4
124.360	P_c4/mcc	d,$\overline{E}_1$	$4/m'm'm'$	15.7.59	$4/mm'm'$	15.6.58	4m'm'	13.4.47	4
124.360	P_c4/mcc	e,$\overline{E}$	$m'm'm'$	8.5.28	$4/m'm'm'$	15.7.59	m'm'2	7.4.23	8
			$m'm'm'$	8.5.28	$m'm'm$	8.4.27	m'm'2	7.4.23	8
124.361	P_C4/mcc	a,$\overline{E}_2$	422	12.1.40	$4/m$	11.1.35	4	9.1.29	8
124.361	P_C4/mcc	a,$\overline{E}_1$	422	12.1.40	$4/m$	11.1.35	4	9.1.29	8
124.361	P_C4/mcc	e, ${}^1\overline{E}\,{}^2\overline{E}$	$2'/m$	5.3.14	$4/m$	11.1.35	m	4.1.9	16
			$2'/m$	5.3.14	$4'/m$	11.3.37	m	4.1.9	16

			$2'/m$	5.3.14	$2'2'2$	6.3.19	2'	3.3.8	16
124.362	P_I4/mcc	a,$\overline{E}_2$	422	12.1.40	$4/m$	11.1.35	4	9.1.29	8
124.362	P_I4/mcc	a,$\overline{E}_1$	422	12.1.40	$4/m$	11.1.35	4	9.1.29	8
124.362	P_I4/mcc	b,$\overline{E}$	$\bar{4}'2m'$	14.4.51	$m'm'm$	8.4.27	m'm'2	7.4.23	8
124.362	P_I4/mcc	e,${}^1\overline{E}\,{}^2\overline{E}$	$2/m'$	5.4.15	422	12.1.40	2	3.1.6	16
			$2/m'$	5.4.15	$\bar{4}'2m'$	14.4.51	m'	4.3.11	16
			$2/m'$	5.4.15	$m'm'm$	8.4.27	m'	4.3.11	16
125.372	P_c4/nbm	a,$\overline{E}_2$	422	12.1.40	$42'2'$	12.4.43	4	9.1.29	8
125.372	P_c4/nbm	a,$\overline{E}_1$	422	12.1.40	$42'2'$	12.4.43	4	9.1.29	8
125.372	P_c4/nbm	f,${}^1\overline{E}\,{}^2\overline{E}$	$2'/m$	5.3.14	$42'2'$	12.4.43	2'	3.3.8	16
			$2'/m$	5.3.14	$\bar{4}2m$	14.1.48	m	4.1.9	16
			$2'/m$	5.3.14	$\bar{4}'2'm$	14.3.50	m	4.1.9	16
			$2'/m$	5.3.14	$2/m$	5.1.12	m	4.1.9	16
126.384	P_c4/nnc	b,$\overline{E}_2$	422	12.1.40	$42'2'$	12.4.43	4	9.1.29	8
126.384	P_c4/nnc	b,$\overline{E}_1$	422	12.1.40	$42'2'$	12.4.43	4	9.1.29	8
126.384	P_c4/nnc	d,$\overline{E}$	$\bar{4}'2m'$	14.4.51	$\bar{4}2'm'$	14.5.52	m'm'2	7.4.23	8
126.384	P_c4/nnc	f,${}^1\overline{E}\,{}^2\overline{E}$	$2/m'$	5.4.15	422	12.1.40	2	3.1.6	16
			$2/m'$	5.4.15	$\bar{4}2'm'$	14.5.52	m'	4.3.11	16
			$2/m'$	5.4.15	$\bar{4}'2m'$	14.4.51	m'	4.3.11	16
			$2/m'$	5.4.15	$2'/m'$	5.5.16	m'	4.3.11	16
126.386	P_I4/nnc	c,$\overline{E}$	$m'm'm'$	8.5.28	$4/m'm'm'$	15.7.59	m'm'2	7.4.23	8
			$m'm'm'$	8.5.28	$\bar{4}2'm'$	14.5.52	m'm'2	7.4.23	8
127.396	P_c4/mbm	b,${}^1\overline{E}_2\,{}^2\overline{E}_2$	$4/m'$	11.4.38	$4/m$	11.1.35	4	9.1.29	8
127.396	P_c4/mbm	b,${}^1\overline{E}_1\,{}^2\overline{E}_1$	$4/m'$	11.4.38	$4/m$	11.1.35	4	9.1.29	8
127.397	P_C4/mbm	e,$\overline{E}$	$m'mm$	8.3.26	$4/mm'm'$	15.6.58	m'm2'	7.3.22	8
127.397	P_C4/mbm	f,$\overline{E}$	$m'mm$	8.3.26	$4/mm'm'$	15.6.58	m'm2'	7.3.22	8
127.398	P_I4/mbm	e,${}^1\overline{E}\,{}^2\overline{E}$	$2'/m$	5.3.14	$42'2'$	12.4.43	2'	3.3.8	16
			$2'/m$	5.3.14	$\bar{4}'2'm$	14.3.50	m	4.1.9	16
			$2'/m$	5.3.14	mmm	8.1.24	m	4.1.9	16
128.408	P_c4/mnc	b,${}^1\overline{E}_2\,{}^2\overline{E}_2$	$4/m'$	11.4.38	$4/m$	11.1.35	4	9.1.29	8
128.408	P_c4/mnc	b,${}^1\overline{E}_1\,{}^2\overline{E}_1$	$4/m'$	11.4.38	$4/m$	11.1.35	4	9.1.29	8
128.408	P_c4/mnc	c,$\overline{E}$	$m'm'm'$	8.5.28	$m'm'm$	8.4.27	m'm'2	7.4.23	8
128.409	P_C4/mnc	e,${}^1\overline{E}\,{}^2\overline{E}$	$2'/m$	5.3.14	$4'/m$	11.3.37	m	4.1.9	16
			$2'/m$	5.3.14	$4/m$	11.1.35	m	4.1.9	16
			$2'/m$	5.3.14	$2'2'2$	6.3.19	2'	3.3.8	16
128.410	P_I4/mnc	d,$\overline{E}$	$\bar{4}'2m'$	14.4.51	$m'm'm$	8.4.27	m'm'2	7.4.23	8
128.410	P_I4/mnc	f,${}^1\overline{E}\,{}^2\overline{E}$	$2/m'$	5.4.15	$4/mm'm'$	15.6.58	m'	4.3.11	16
			$2/m'$	5.4.15	$\bar{4}'2m'$	14.4.51	2	3.1.6	16
129.420	P_c4/nmm	e,${}^1\overline{E}\,{}^2\overline{E}$	$2'/m$	5.3.14	$\bar{4}'2'm$	14.3.50	2'	3.3.8	16
			$2'/m$	5.3.14	$4mm$	13.1.44	m	4.1.9	16
			$2'/m$	5.3.14	$2/m$	5.1.12	m	4.1.9	16
129.422	P_I4/nmm	c,$\overline{E}$	$m'mm$	8.3.26	$4/m'mm$	15.3.55	m'm2'	7.3.22	8
130.432	P_c4/ncc	b,$\overline{E}$	$\bar{4}'2m'$	14.4.51	$\bar{4}2'm'$	14.5.52	m'm'2	7.4.23	8
130.432	P_c4/ncc	e,${}^1\overline{E}\,{}^2\overline{E}$	$2/m'$	5.4.15	$\bar{4}'2m'$	14.4.51	2	3.1.6	16
			$2/m'$	5.4.15	$4m'm'$	13.4.47	m'	4.3.11	16
			$2/m'$	5.4.15	$2'/m'$	5.5.16	m'	4.3.11	16
130.433	P_C4/ncc	b,${}^1\overline{E}_2\,{}^2\overline{E}_2$	$4/m'$	11.4.38	$42'2'$	12.4.43	4	9.1.29	8
130.433	P_C4/ncc	b,${}^1\overline{E}_1\,{}^2\overline{E}_1$	$4/m'$	11.4.38	$42'2'$	12.4.43	4	9.1.29	8
130.434	P_I4/ncc	c,${}^1\overline{E}_2\,{}^2\overline{E}_2$	$4/m'$	11.4.38	$42'2'$	12.4.43	4	9.1.29	8
130.434	P_I4/ncc	c,${}^1\overline{E}_1\,{}^2\overline{E}_1$	$4/m'$	11.4.38	$42'2'$	12.4.43	4	9.1.29	8
130.434	P_I4/ncc	d,$\overline{E}$	$m'm'm'$	8.5.28	$\bar{4}2'm'$	14.5.52	m'm'2	7.4.23	8
131.444	P_c4_2/mmc	e,$\overline{E}$	$m'mm$	8.3.26	$4'/m'm'm$	15.5.57	m'm2'	7.3.22	8
131.445	P_C4_2/mmc	f,${}^1\overline{E}\,{}^2\overline{E}$	$2'/m$	5.3.14	mmm	8.1.24	m	4.1.9	16
			$2'/m$	5.3.14	$2'2'2$	6.3.19	2'	3.3.8	16
131.446	P_I4_2/mmc	f,${}^1\overline{E}\,{}^2\overline{E}$	$2/m'$	5.4.15	$4'/mm'm$	15.4.56	m'	4.3.11	16
			$2/m'$	5.4.15	$\bar{4}2m$	14.1.48	2	3.1.6	16
132.456	P_c4_2/mcm	e,$\overline{E}$	$m'm'm'$	8.5.28	$4'/m'm'm$	15.5.57	m'm'2	7.4.23	8
			$m'm'm'$	8.5.28	$m'm'm$	8.4.27	m'm'2	7.4.23	8
132.457	P_C4_2/mcm	c,$\overline{E}$	$m'mm$	8.3.26	$m'm'm$	8.4.27	m'm2'	7.3.22	8
132.457	P_C4_2/mcm	d,$\overline{E}$	$m'mm$	8.3.26	$m'm'm$	8.4.27	m'm2'	7.3.22	8
132.457	P_C4_2/mcm	f,$\overline{E}$	$\bar{4}'2m'$	14.4.51	$m'm'm$	8.4.27	m'm'2	7.4.23	8
132.458	P_I4_2/mcm	e,${}^1\overline{E}\,{}^2\overline{E}$	$2'/m$	5.3.14	$4'22'$	12.3.42	2'	3.3.8	16
			$2'/m$	5.3.14	$\bar{4}2m$	14.1.48	m	4.1.9	16
			$2'/m$	5.3.14	mmm	8.1.24	m	4.1.9	16
133.468	P_c4_2/nbc	c,$\overline{E}$	$\bar{4}'2m'$	14.4.51	$\bar{4}2'm'$	14.5.52	m'm'2	7.4.23	8
133.468	P_c4_2/nbc	f,${}^1\overline{E}\,{}^2\overline{E}$	$2/m'$	5.4.15	$4'22'$	12.3.42	2	3.1.6	16
			$2/m'$	5.4.15	$\bar{4}'2m'$	14.4.51	m'	4.3.11	16

			$2/m'$	5.4.15	$\bar{4}2'm'$	14.5.52	m'	4.3.11	16
			$2/m'$	5.4.15	$2'/m'$	5.5.16	m'	4.3.11	16
133.469	P_C4_2/nbc	a,$\overline{E}$	$m'm'm'$	8.5.28	$\bar{4}2'm'$	14.5.52	m'm'2	7.4.23	8
133.469	P_C4_2/nbc	c,$\overline{E}$	$m'm'm'$	8.5.28	$\bar{4}'2m'$	14.4.51	m'm'2	7.4.23	8
133.469	P_C4_2/nbc	d,$\overline{E}$	$\bar{4}'2m'$	14.4.51	$m'm'm'$	8.5.28	m'm'2	7.4.23	8
134.480	P_c4_2/nnm	f, $^1\overline{E}\,^2\overline{E}$	$2'/m$	5.3.14	$4'22'$	12.3.42	2'	3.3.8	16
			$2'/m$	5.3.14	$\bar{4}'2'm$	14.3.50	m	4.1.9	16
			$2'/m$	5.3.14	$\bar{4}2m$	14.1.48	m	4.1.9	16
			$2'/m$	5.3.14	$2/m$	5.1.12	m	4.1.9	16
134.481	P_C4_2/nnm	a,$\overline{E}$	$m'mm$	8.3.26	$m'm'm$	8.4.27	m'm2'	7.3.22	8
134.481	P_C4_2/nnm	b,$\overline{E}$	$m'm'm'$	8.5.28	$m'm'm$	8.4.27	m'm'2	7.4.23	8
			$m'm'm'$	8.5.28	$\bar{4}'2m'$	14.4.51	m'm'2	7.4.23	8
134.481	P_C4_2/nnm	f,$\overline{E}$	$\bar{4}'2m'$	14.4.51	$m'm'm'$	8.5.28	m'm'2	7.4.23	8
134.482	P_I4_2/nnm	c,$\overline{E}$	$m'm'm'$	8.5.28	$4'/m'm'm$	15.5.57	m'm'2	7.4.23	8
			$m'm'm'$	8.5.28	$\bar{4}'2m'$	14.4.51	m'm'2	7.4.23	8
134.482	P_I4_2/nnm	d,$\overline{E}$	$\bar{4}'2m'$	14.4.51	$m'm'm'$	8.5.28	m'm'2	7.4.23	8
135.492	P_c4_2/mbc	c,$\overline{E}$	$m'm'm'$	8.5.28	$m'm'm$	8.4.27	m'm'2	7.4.23	8
135.493	P_C4_2/mbc	b,$\overline{E}$	$\bar{4}'2m'$	14.4.51	$m'm'm$	8.4.27	m'm'2	7.4.23	8
135.493	P_C4_2/mbc	f, $^1\overline{E}\,^2\overline{E}$	$2'/m$	5.3.14	$m'm'm$	8.4.27	m	4.1.9	16
			$2'/m$	5.3.14	$2'2'2$	6.3.19	2'	3.3.8	16
135.494	P_I4_2/mbc	e, $^1\overline{E}\,^2\overline{E}$	$2/m'$	5.4.15	$4'22'$	12.3.42	2	3.1.6	16
			$2/m'$	5.4.15	$\bar{4}2'm'$	14.5.52	m'	4.3.11	16
			$2/m'$	5.4.15	$m'm'm$	8.4.27	m'	4.3.11	16
136.505	P_C4_2/mnm	c,$\overline{E}$	$m'mm$	8.3.26	$m'm'm$	8.4.27	m'm2'	7.3.22	8
136.505	P_C4_2/mnm	d,$\overline{E}$	$m'mm$	8.3.26	$m'm'm$	8.4.27	m'm2'	7.3.22	8
136.506	P_I4_2/mnm	f, $^1\overline{E}\,^2\overline{E}$	$2'/m$	5.3.14	$4'/mm'm$	15.4.56	m	4.1.9	16
			$2'/m$	5.3.14	$\bar{4}2'm'$	14.5.52	2'	3.3.8	16
137.516	P_c4_2/nmc	e, $^1\overline{E}\,^2\overline{E}$	$2/m'$	5.4.15	$\bar{4}2m$	14.1.48	2	3.1.6	16
			$2/m'$	5.4.15	$4'm'm$	13.3.46	m'	4.3.11	16
			$2/m'$	5.4.15	$2'/m'$	5.5.16	m'	4.3.11	16
137.518	P_I4_2/nmc	c,$\overline{E}$	$m'mm$	8.3.26	$4'/m'm'm$	15.5.57	m'm2'	7.3.22	8
138.528	P_c4_2/ncm	a,$\overline{E}$	$\bar{4}'2m'$	14.4.51	$\bar{4}2'm'$	14.5.52	m'm'2	7.4.23	8
138.528	P_c4_2/ncm	e, $^1\overline{E}\,^2\overline{E}$	$2'/m$	5.3.14	$\bar{4}2'm'$	14.5.52	2'	3.3.8	16
			$2'/m$	5.3.14	$4'm'm$	13.3.46	m	4.1.9	16
			$2'/m$	5.3.14	$2/m$	5.1.12	m	4.1.9	16
138.529	P_C4_2/ncm	a,$\overline{E}$	$m'mm$	8.3.26	$m'm'm$	8.4.27	m'm2'	7.3.22	8
138.529	P_C4_2/ncm	b,$\overline{E}$	$m'm'm'$	8.5.28	$m'm'm$	8.4.27	m'm'2	7.4.23	8
			$m'm'm'$	8.5.28	$\bar{4}2'm'$	14.5.52	m'm'2	7.4.23	8
139.540	I_c4/mmm	f,$\overline{E}$	$m'mm$	8.3.26	$4'/mm'm$	15.4.56	m'm2'	7.3.22	8
			$m'mm$	8.3.26	$m'm'm$	8.4.27	m'm2'	7.3.22	8
140.550	I_c4/mcm	b,$\overline{E}_2$	$4/m'm'm'$	15.7.59	$4/mm'm'$	15.6.58	4m'm'	13.4.47	4
140.550	I_c4/mcm	b,$\overline{E}_1$	$4/m'm'm'$	15.7.59	$4/mm'm'$	15.6.58	4m'm'	13.4.47	4
140.550	I_c4/mcm	f,$\overline{E}$	$m'mm$	8.3.26	$4/mm'm'$	15.6.58	m'm2'	7.3.22	8
			$m'mm$	8.3.26	$m'm'm$	8.4.27	m'm2'	7.3.22	8
141.560	I_c4_1/amd	f, $^1\overline{E}\,^2\overline{E}$	$2'/m$	5.3.14	$\bar{4}2m$	14.1.48	m	4.1.9	16
			$2'/m$	5.3.14	$\bar{4}'2'm$	14.3.50	m	4.1.9	16
			$2'/m$	5.3.14	$2'2'2$	6.3.19	2'	3.3.8	16
			$2'/m$	5.3.14	$2/m$	5.1.12	m	4.1.9	16
142.570	I_c4_1/acd	b,$\overline{E}$	$\bar{4}'2m'$	14.4.51	$\bar{4}2'm'$	14.5.52	m'm'2	7.4.23	8
142.570	I_c4_1/acd	f, $^1\overline{E}\,^2\overline{E}$	$2/m'$	5.4.15	$\bar{4}2'm'$	14.5.52	m'	4.3.11	16
			$2/m'$	5.4.15	$\bar{4}'2m'$	14.4.51	m'	4.3.11	16
			$2/m'$	5.4.15	$2'2'2$	6.3.19	2	3.1.6	16
			$2/m'$	5.4.15	$2'/m'$	5.5.16	m'	4.3.11	16
147.16	$P_c\bar{3}$	b,$\overline{E}\overline{E}$	$\bar{3}'$	17.3.64	$\bar{3}$	17.1.62	3	16.1.60	4
147.16	$P_c\bar{3}$	b, $^1\overline{E}\,^2\overline{E}$	$\bar{3}'$	17.3.64	$\bar{3}$	17.1.62	3	16.1.60	4
147.16	$P_c\bar{3}$	f,$\overline{A}\overline{A}$	$\bar{1}'$	2.3.5	$\bar{3}$	17.1.62	1	1.1.1	12
			$\bar{1}'$	2.3.5	$\bar{3}'$	17.3.64	1	1.1.1	12
			$\bar{1}'$	2.3.5	3	16.1.60	1	1.1.1	12
			$\bar{1}'$	2.3.5	$\bar{1}$	2.1.3	1	1.1.1	12
148.20	$R_I\bar{3}$	b,$\overline{E}\overline{E}$	$\bar{3}'$	17.3.64	$\bar{3}$	17.1.62	3	16.1.60	4
148.20	$R_I\bar{3}$	b, $^1\overline{E}\,^2\overline{E}$	$\bar{3}'$	17.3.64	$\bar{3}$	17.1.62	3	16.1.60	4
148.20	$R_I\bar{3}$	d,$\overline{A}\overline{A}$	$\bar{1}'$	2.3.5	$\bar{3}$	17.1.62	1	1.1.1	12
			$\bar{1}'$	2.3.5	$\bar{3}'$	17.3.64	1	1.1.1	12
			$\bar{1}'$	2.3.5	$\bar{1}$	2.1.3	1	1.1.1	12
149.24	P_c312	a,$\overline{E}_1$	32	18.1.65	$32'$	18.3.67	3	16.1.60	4
149.24	P_c312	c,$\overline{E}_1$	32	18.1.65	$32'$	18.3.67	3	16.1.60	4
149.24	P_c312	e,$\overline{E}_1$	32	18.1.65	$32'$	18.3.67	3	16.1.60	4
150.28	P_c321	a,$\overline{E}_1$	32	18.1.65	$32'$	18.3.67	3	16.1.60	4
155.48	R_I32	a,$\overline{E}_1$	32	18.1.65	$32'$	18.3.67	3	16.1.60	4
162.78	$P_c\bar{3}1m$	b, $^1\overline{E}\,^2\overline{E}$	$\bar{3}'1m$	20.3.73	$\bar{3}1m$	20.1.71	31m	19.1.68	4

162.78	$P_c\bar{3}1m$	c,$\overline{E}_1$	32	18.1.65	$32'$	18.3.67	3	16.1.60	8
162.78	$P_c\bar{3}1m$	g,$^1\overline{E}\,^2\overline{E}$	$2'/m$	5.3.14	$\bar{3}1m$	20.1.71	m	4.1.9	12
			$2'/m$	5.3.14	$\bar{3}'1m$	20.3.73	2'	3.3.8	12
			$2'/m$	5.3.14	$32'$	18.3.67	2'	3.3.8	12
			$2'/m$	5.3.14	$2/m$	5.1.12	m	4.1.9	12
163.84	$P_c\bar{3}1c$	b,$^1\overline{E}\,^2\overline{E}$	$\bar{3}'1m'$	20.4.74	$\bar{3}1m'$	20.5.75	31m'	19.3.70	4
163.84	$P_c\bar{3}1c$	b,$\overline{E}_1$	$\bar{3}'1m'$	20.4.74	$\bar{3}1m'$	20.5.75	31m'	19.3.70	4
163.84	$P_c\bar{3}1c$	d,$\overline{E}_1$	32	18.1.65	$32'$	18.3.67	3	16.1.60	8
163.84	$P_c\bar{3}1c$	g,$^1\overline{E}\,^2\overline{E}$	$2/m'$	5.4.15	$\bar{3}1m'$	20.5.75	m'	4.3.11	12
			$2/m'$	5.4.15	$\bar{3}'1m'$	20.4.74	2	3.1.6	12
			$2/m'$	5.4.15	32	18.1.65	2	3.1.6	12
			$2/m'$	5.4.15	$2'/m'$	5.5.16	m'	4.3.11	12
164.90	$P_c\bar{3}m1$	b,$^1\overline{E}\,^2\overline{E}$	$\bar{3}'1m$	20.3.73	$\bar{3}1m$	20.1.71	31m	19.1.68	4
164.90	$P_c\bar{3}m1$	f,$^1\overline{E}\,^2\overline{E}$	$2'/m$	5.3.14	$\bar{3}1m$	20.1.71	m	4.1.9	12
			$2'/m$	5.3.14	$\bar{3}'1m$	20.3.73	2'	3.3.8	12
			$2'/m$	5.3.14	$3m$	19.1.68	m	4.1.9	12
			$2'/m$	5.3.14	$2/m$	5.1.12	m	4.1.9	12
165.96	$P_c\bar{3}c1$	b,$^1\overline{E}\,^2\overline{E}$	$\bar{3}'1m'$	20.4.74	$\bar{3}1m'$	20.5.75	31m'	19.3.70	4
165.96	$P_c\bar{3}c1$	b,$\overline{E}_1$	$\bar{3}'1m'$	20.4.74	$\bar{3}1m'$	20.5.75	31m'	19.3.70	4
165.96	$P_c\bar{3}c1$	f,$^1\overline{E}\,^2\overline{E}$	$2/m'$	5.4.15	$\bar{3}1m'$	20.5.75	m'	4.3.11	12
			$2/m'$	5.4.15	$\bar{3}'1m'$	20.4.74	2	3.1.6	12
			$2/m'$	5.4.15	$3m'$	19.3.70	m'	4.3.11	12
			$2/m'$	5.4.15	$2'/m'$	5.5.16	m'	4.3.11	12
166.102	$R_I\bar{3}m$	b,$^1\overline{E}\,^2\overline{E}$	$\bar{3}'1m$	20.3.73	$\bar{3}1m$	20.1.71	31m	19.1.68	4
166.102	$R_I\bar{3}m$	d,$^1\overline{E}\,^2\overline{E}$	$2'/m$	5.3.14	$\bar{3}1m$	20.1.71	m	4.1.9	12
			$2'/m$	5.3.14	$\bar{3}'1m$	20.3.73	2'	3.3.8	12
			$2'/m$	5.3.14	$2/m$	5.1.12	m	4.1.9	12
167.108	$R_I\bar{3}c$	b,$^1\overline{E}\,^2\overline{E}$	$\bar{3}'1m'$	20.4.74	$\bar{3}1m'$	20.5.75	31m'	19.3.70	4
167.108	$R_I\bar{3}c$	b,$\overline{E}_1$	$\bar{3}'1m'$	20.4.74	$\bar{3}1m'$	20.5.75	31m'	19.3.70	4
167.108	$R_I\bar{3}c$	d,$^1\overline{E}\,^2\overline{E}$	$2/m'$	5.4.15	$\bar{3}1m'$	20.5.75	m'	4.3.11	12
			$2/m'$	5.4.15	$\bar{3}'1m'$	20.4.74	2	3.1.6	12
			$2/m'$	5.4.15	$2'/m'$	5.5.16	m'	4.3.11	12
174.136	$P_c\bar{6}$	b,$^1\overline{E}\,^2\overline{E}$	$\bar{6}'$	22.3.81	$\bar{6}$	22.1.79	3	16.1.60	4
174.136	$P_c\bar{6}$	d,$^1\overline{E}\,^2\overline{E}$	$\bar{6}'$	22.3.81	$\bar{6}$	22.1.79	3	16.1.60	4
174.136	$P_c\bar{6}$	f,$^1\overline{E}\,^2\overline{E}$	$\bar{6}'$	22.3.81	$\bar{6}$	22.1.79	3	16.1.60	4
175.142	P_c6/m	b,$^1\overline{E}_1\,^2\overline{E}_1$	$6/m'$	23.4.85	$6/m$	23.1.82	6	21.1.76	4
175.142	P_c6/m	b,$^1\overline{E}3\,^2\overline{E}3$	$6/m'$	23.4.85	$6/m$	23.1.82	6	21.1.76	4
175.142	P_c6/m	b,$^1\overline{E}_2\,^2\overline{E}_2$	$6/m'$	23.4.85	$6/m$	23.1.82	6	21.1.76	4
175.142	P_c6/m	d,$^1\overline{E}\,^2\overline{E}$	$\bar{6}'$	22.3.81	$\bar{6}$	22.1.79	3	16.1.60	8
175.142	P_c6/m	g,$^1\overline{E}\,^2\overline{E}$	$2/m'$	5.4.15	$6/m'$	23.4.85	m'	4.3.11	12
			$2/m'$	5.4.15	$\bar{6}'$	22.3.81	m'	4.3.11	12
			$2/m'$	5.4.15	$2/m$	5.1.12	2	3.1.6	12
176.148	P_c6_3/m	a,$^1\overline{E}_1\,^2\overline{E}_1$	$6'/m$	23.3.84	$6'/m'$	23.5.86	6'	21.3.78	4
176.148	P_c6_3/m	d,$^1\overline{E}\,^2\overline{E}$	$\bar{6}'$	22.3.81	$\bar{6}$	22.1.79	3	16.1.60	8
176.148	P_c6_3/m	f,$^1\overline{E}\,^2\overline{E}$	$2'/m$	5.3.14	$6'/m$	23.3.84	m	4.1.9	12
			$2'/m$	5.3.14	$\bar{6}$	22.1.79	m	4.1.9	12
			$2'/m$	5.3.14	$2'/m'$	5.5.16	2'	3.3.8	12
177.154	P_c622	a,$\overline{E}_3$	622	24.1.87	$62'2'$	24.4.90	6	21.1.76	4
177.154	P_c622	a,$\overline{E}_2$	622	24.1.87	$62'2'$	24.4.90	6	21.1.76	4
177.154	P_c622	a,$\overline{E}_1$	622	24.1.87	$62'2'$	24.4.90	6	21.1.76	4
177.154	P_c622	c,$\overline{E}_1$	32	18.1.65	$32'$	18.3.67	3	16.1.60	8
177.154	P_c622	f,$\overline{E}$	222	6.1.17	622	24.1.87	2	3.1.6	12
			222	6.1.17	32	18.1.65	2	3.1.6	12
			222	6.1.17	$2'2'2$	6.3.19	2	3.1.6	12
180.172	P_c6_222	a,$\overline{E}$	222	6.1.17	$2'2'2$	6.3.19	2	3.1.6	12
180.172	P_c6_222	c,$\overline{E}$	222	6.1.17	$2'2'2$	6.3.19	2	3.1.6	12
181.178	P_c6_422	a,$\overline{E}$	222	6.1.17	$2'2'2$	6.3.19	2	3.1.6	12
181.178	P_c6_422	c,$\overline{E}$	222	6.1.17	$2'2'2$	6.3.19	2	3.1.6	12
182.184	P_c6_322	d,$\overline{E}_1$	32	18.1.65	$32'$	18.3.67	3	16.1.60	8
183.190	P_c6mm	c,$\overline{E}$	$mm2$	7.1.20	$6mm$	25.1.91	m	4.1.9	12
			$mm2$	7.1.20	$3m$	19.1.68	m	4.1.9	12
187.214	$P_c\bar{6}m2$	a,$\overline{E}_3$	$\bar{6}m2$	26.1.95	$\bar{6}'m2'$	26.4.98	31m	19.1.68	4
187.214	$P_c\bar{6}m2$	c,$\overline{E}_3$	$\bar{6}m2$	26.1.95	$\bar{6}'m2'$	26.4.98	31m	19.1.68	4
187.214	$P_c\bar{6}m2$	e,$\overline{E}_3$	$\bar{6}m2$	26.1.95	$\bar{6}'m2'$	26.4.98	31m	19.1.68	4
188.220	$P_c\bar{6}c2$	a,$\overline{E}_1$	$\bar{6}'m'2$	26.3.97	$\bar{6}m'2'$	26.5.99	31m'	19.3.70	4
188.220	$P_c\bar{6}c2$	c,$\overline{E}_1$	$\bar{6}'m'2$	26.3.97	$\bar{6}m'2'$	26.5.99	31m'	19.3.70	4
188.220	$P_c\bar{6}c2$	e,$\overline{E}_1$	$\bar{6}'m'2$	26.3.97	$\bar{6}m'2'$	26.5.99	31m'	19.3.70	4
189.226	$P_c\bar{6}2m$	a,$\overline{E}_3$	$\bar{6}m2$	26.1.95	$\bar{6}'m2'$	26.4.98	31m	19.1.68	4
189.226	$P_c\bar{6}2m$	d,$^1\overline{E}\,^2\overline{E}$	$\bar{6}'$	22.3.81	$\bar{6}$	22.1.79	3	16.1.60	8

190.232	$P_c\bar{6}2c$	a,$\overline{E}_1$	$\bar{6}'m'2$	26.3.97	$\bar{6}m'2'$	26.5.99	31m'	19.3.70	4
190.232	$P_c\bar{6}2c$	c, $^1\overline{E}\,^2\overline{E}$	$\bar{6}'$	22.3.81	$\bar{6}$	22.1.79	3	16.1.60	8
191.242	P_c6/mmm	c,$\overline{E}_3$	$\bar{6}m2$	26.1.95	$\bar{6}'m2'$	26.4.98	31m	19.1.68	8
191.242	P_c6/mmm	g,$\overline{E}$	$m'mm$	8.3.26	$6/m'mm$	27.3.102	m'm2'	7.3.22	12
			$m'mm$	8.3.26	$\bar{6}'m2'$	26.4.98	m'm2'	7.3.22	12
192.252	P_c6/mcc	b,$\overline{E}_3$	$6/m'm'm'$	27.7.106	$6/mm'm'$	27.6.105	6m'm'	25.4.94	4
192.252	P_c6/mcc	b,$\overline{E}_2$	$6/m'm'm'$	27.7.106	$6/mm'm'$	27.6.105	6m'm'	25.4.94	4
192.252	P_c6/mcc	b,$\overline{E}_1$	$6/m'm'm'$	27.7.106	$6/mm'm'$	27.6.105	6m'm'	25.4.94	4
192.252	P_c6/mcc	d,$\overline{E}_1$	$\bar{6}'m'2$	26.3.97	$\bar{6}m'2'$	26.5.99	31m'	19.3.70	8
192.252	P_c6/mcc	g,$\overline{E}$	$m'm'm'$	8.5.28	$6/m'm'm'$	27.7.106	m'm'2	7.4.23	12
			$m'm'm'$	8.5.28	$\bar{6}'m'2$	26.3.97	m'm'2	7.4.23	12
			$m'm'm'$	8.5.28	$m'm'm$	8.4.27	m'm'2	7.4.23	12
193.262	P_c6_3/mcm	a,$\overline{E}_3$	$6'/mmm'$	27.4.103	$6'/m'mm'$	27.5.104	6'mm'	25.3.93	4
193.262	P_c6_3/mcm	d,$\overline{E}_1$	$\bar{6}'m'2$	26.3.97	$\bar{6}m'2'$	26.5.99	31m'	19.3.70	8
193.262	P_c6_3/mcm	f,$\overline{E}$	$m'mm$	8.3.26	$\bar{6}m'2'$	26.5.99	m'm2'	7.3.22	12
			$m'mm$	8.3.26	$m'm'm$	8.4.27	m'm2'	7.3.22	12
194.272	P_c6_3/mmc	a,$\overline{E}_3$	$6'/mmm'$	27.4.103	$6'/m'mm'$	27.5.104	6'mm'	25.3.93	4
194.272	P_c6_3/mmc	c,$\overline{E}_3$	$\bar{6}m2$	26.1.95	$\bar{6}'m2'$	26.4.98	31m	19.1.68	8
194.272	P_c6_3/mmc	f,$\overline{E}$	$m'mm$	8.3.26	$6'/mmm'$	27.4.103	m'm2'	7.3.22	12
			$m'mm$	8.3.26	$m'm'm$	8.4.27	m'm2'	7.3.22	12
195.3	P_I23	b,$\overline{E}$	222	6.1.17	23	28.1.107	2	3.1.6	12
200.17	$P_Im\bar{3}$	c,$\overline{E}\overline{E}$	$\bar{3}'$	17.3.64	$m\bar{3}$	29.1.109	3	16.1.60	16
200.17	$P_Im\bar{3}$	c, $^1\overline{E}\,^2\overline{E}$	$\bar{3}'$	17.3.64	$m\bar{3}$	29.1.109	3	16.1.60	16
201.21	$P_In\bar{3}$	b,$\overline{E}$	$m'm'm'$	8.5.28	$m'\bar{3}'$	29.3.111	m'm'2	7.4.23	12
202.25	$F_Sm\bar{3}$	d,$\overline{E}$	$m'mm$	8.3.26	$m'm'm$	8.4.27	m'm2'	7.3.22	12
203.29	$F_Sd\bar{3}$	c,$\overline{E}\overline{E}$	$\bar{3}'$	17.3.64	23	28.1.107	3	16.1.60	16
			$\bar{3}'$	17.3.64	$\bar{3}$	17.1.62	3	16.1.60	16
203.29	$F_Sd\bar{3}$	c, $^1\overline{E}\,^2\overline{E}$	$\bar{3}'$	17.3.64	23	28.1.107	3	16.1.60	16
			$\bar{3}'$	17.3.64	$\bar{3}$	17.1.62	3	16.1.60	16
205.36	$P_Ia\bar{3}$	a,$\overline{E}\overline{E}$	$\bar{3}'$	17.3.64	$\bar{3}$	17.1.62	3	16.1.60	16
205.36	$P_Ia\bar{3}$	a, $^1\overline{E}\,^2\overline{E}$	$\bar{3}'$	17.3.64	$\bar{3}$	17.1.62	3	16.1.60	16
207.43	P_I432	b,$\overline{E}_2$	422	12.1.40	432	30.1.112	4	9.1.29	12
207.43	P_I432	b,$\overline{E}_1$	422	12.1.40	432	30.1.112	4	9.1.29	12
208.47	P_I4_232	c,$\overline{E}_1$	32	18.1.65	$4'32'$	30.3.114	3	16.1.60	16
208.47	P_I4_232	d,$\overline{E}$	222	6.1.17	$4'22'$	12.3.42	2	3.1.6	24
			222	6.1.17	32	18.1.65	2	3.1.6	24
210.55	F_S4_132	b,$\overline{E}_1$	32	18.1.65	23	28.1.107	3	16.1.60	16
			32	18.1.65	$32'$	18.3.67	3	16.1.60	16
212.62	P_I4_332	a,$\overline{E}_1$	32	18.1.65	$32'$	18.3.67	3	16.1.60	16
213.66	P_I4_132	b,$\overline{E}_1$	32	18.1.65	$32'$	18.3.67	3	16.1.60	16
215.73	$P_I\bar{4}3m$	d, $^1\overline{E}\,^2\overline{E}$	$\bar{4}'$	10.3.34	$\bar{4}2m$	14.1.48	2	3.1.6	24
218.84	$P_I\bar{4}3n$	b,$\overline{E}$	$\bar{4}'2m'$	14.4.51	$\bar{4}'3m'$	31.3.117	m'm'2	7.4.23	12
221.97	$P_Im\bar{3}m$	c, $^1\overline{E}\,^2\overline{E}$	$\bar{3}'1m$	20.3.73	$m\bar{3}m$	32.1.118	31m	19.1.68	16
222.103	$P_In\bar{3}n$	b,$\overline{E}_2$	$4/m'm'm'$	15.7.59	$m'\bar{3}'m'$	32.5.122	4m'm'	13.4.47	12
222.103	$P_In\bar{3}n$	b,$\overline{E}_1$	$4/m'm'm'$	15.7.59	$m'\bar{3}'m'$	32.5.122	4m'm'	13.4.47	12
223.109	$P_Im\bar{3}n$	c, $^1\overline{E}\,^2\overline{E}$	$\bar{3}'1m'$	20.4.74	$m\bar{3}m'$	32.4.121	31m'	19.3.70	16
223.109	$P_Im\bar{3}n$	c,$\overline{E}_1$	$\bar{3}'1m'$	20.4.74	$m\bar{3}m'$	32.4.121	31m'	19.3.70	16
224.115	$P_In\bar{3}m$	d,$\overline{E}$	$\bar{4}'2m'$	14.4.51	$4'/m'm'm$	15.5.57	m'm'2	7.4.23	24
227.133	$F_Sd\bar{3}m$	a,$\overline{F}$	$\bar{4}3m$	31.1.115	$\bar{3}1m$	20.1.71	31m	19.1.68	16
			$\bar{4}3m$	31.1.115	$\bar{3}'1m$	20.3.73	31m	19.1.68	16
227.133	$F_Sd\bar{3}m$	c, $^1\overline{E}\,^2\overline{E}$	$\bar{3}'1m$	20.3.73	$\bar{4}3m$	31.1.115	31m	19.1.68	16
			$\bar{3}'1m$	20.3.73	$\bar{3}1m$	20.1.71	31m	19.1.68	16
228.139	$F_Sd\bar{3}c$	c, $^1\overline{E}\,^2\overline{E}$	$\bar{3}'1m'$	20.4.74	$\bar{4}'3m'$	31.3.117	31m'	19.3.70	16
			$\bar{3}'1m'$	20.4.74	$\bar{3}1m'$	20.5.75	31m'	19.3.70	16
228.139	$F_Sd\bar{3}c$	c,$\overline{E}_1$	$\bar{3}'1m'$	20.4.74	$\bar{4}'3m'$	31.3.117	31m'	19.3.70	16
			$\bar{3}'1m'$	20.4.74	$\bar{3}1m'$	20.5.75	31m'	19.3.70	16

38. Maximum and Minimum Dimensions of the Single- and Double-Valued MEBRs

In this section we provide a complete tabulation of the maximum (M) and minimum (m) EBR dimension in each single and double SSG. As discussed in SN 23, the EBRs are composed of the MEBRs of the Type-I MSGs and the PEBRs of the Type-II SGs previously computed for TQC[1–6], as well as the MEBRs of the Type-III and Type-IV MSGs that were calculated for the present work. For each EBR $\tilde{\rho}_{\mathbf{q}}^{G}$, the dimension of $\tilde{\rho}_{\mathbf{q}}^{G}$ is defined as $\chi_{\tilde{\rho}_{\mathbf{q}}}(E)\times n$, where $\tilde{\rho}_{\mathbf{q}}$ is the (co)rep of the site-symmetry group $G_{\mathbf{q}}$ from which $\tilde{\rho}_{\mathbf{q}}^{G}$ is induced [see SEq. (162) and the surrounding text], and where n is the multiplicity of the Wyckoff position indexed by $\mathbf{q}$ [see the text surrounding SEq. (163)]. For each single and double SSG, we have confirmed that the minimum EBR dimension m is consistent with the minimum atomic insulator dimension previously calculated in SRef. 65. We emphasize that the maximum EBR dimension M does not always coincide with the maximum band connectivity in an SSG, due to the possibility of decomposable [*i.e.* disconnected or split] EBRs (see SRefs. 1,3,5,6,87,109,136 and the text surrounding Supplementary Tables 8 and 9). To calculate the maximum band connectivity in the 1,651 SSGs, one must perform the intermediate tabulation of the *basic bands*[87], which for each SSG are composed of the disconnected branches of the decomposable EBRs, the connected [indecomposable] EBRs, and the connected topological bands in the SSG (SN 26). The basic bands of the Type-II double SGs were previously tabulated in SRef. 87; we leave the complete enumeration of the basic bands of the single SSGs and the double MSGs for future works.

Supplementary Table 22: Maximum and minimum dimensions of the single-valued EBRs of the 1,651 single SSGs. In order, the columns in this table list the symbol of the SSG, the number of the SSG in the BNS setting[67], the maximum EBR dimension in the SSG (M), and the minimum EBR dimension in the SSG (m).

Symbol	BNS Number	M	m	Symbol	BNS Number	M	m	Symbol	BNS Number	M	m	Symbol	BNS Number	M	m
$P1$	1.1	1	1	$P11'$	1.2	1	1	P_S1	1.3	2	2	$P\bar{1}$	2.4	1	1
$P\bar{1}1'$	2.5	1	1	$P\bar{1}'$	2.6	1	1	$P_S\bar{1}$	2.7	2	2	$P2$	3.1	1	1
$P21'$	3.2	1	1	$P2'$	3.3	1	1	P_a2	3.4	2	2	P_b2	3.5	2	2
P_C2	3.6	2	2	$P2_1$	4.7	2	2	$P2_11'$	4.8	2	2	$P2_1'$	4.9	2	2
P_a2_1	4.10	4	4	P_b2_1	4.11	2	2	P_C2_1	4.12	2	2	$C2$	5.13	1	1
$C21'$	5.14	1	1	$C2'$	5.15	1	1	C_c2	5.16	2	2	C_a2	5.17	2	2
Pm	6.18	1	1	$Pm1'$	6.19	1	1	Pm'	6.20	1	1	P_am	6.21	2	2
P_bm	6.22	2	2	P_Cm	6.23	2	2	Pc	7.24	2	2	$Pc1'$	7.25	2	2
Pc'	7.26	2	2	P_ac	7.27	4	4	P_cc	7.28	2	2	P_bc	7.29	4	4
P_Cc	7.30	4	4	P_Ac	7.31	2	2	Cm	8.32	1	1	$Cm1'$	8.33	1	1
Cm'	8.34	1	1	C_cm	8.35	2	2	C_am	8.36	2	2	Cc	9.37	2	2
$Cc1'$	9.38	2	2	Cc'	9.39	2	2	C_cc	9.40	2	2	C_ac	9.41	4	4
$P2/m$	10.42	1	1	$P2/m1'$	10.43	1	1	$P2'/m$	10.44	1	1	$P2/m'$	10.45	1	1
$P2'/m'$	10.46	1	1	P_a2/m	10.47	2	2	P_b2/m	10.48	2	2	P_C2/m	10.49	4	2
$P2_1/m$	11.50	2	2	$P2_1/m1'$	11.51	2	2	$P2_1'/m$	11.52	2	2	$P2_1/m'$	11.53	2	2
$P2_1'/m'$	11.54	2	2	P_a2_1/m	11.55	4	4	P_b2_1/m	11.56	2	2	P_C2_1/m	11.57	4	2
$C2/m$	12.58	2	1	$C2/m1'$	12.59	2	1	$C2'/m$	12.60	2	1	$C2/m'$	12.61	2	1
$C2'/m'$	12.62	2	1	C_c2/m	12.63	4	2	C_a2/m	12.64	2	2	$P2/c$	13.65	2	2
$P2/c1'$	13.66	2	2	$P2'/c$	13.67	2	2	$P2/c'$	13.68	2	2	$P2'/c'$	13.69	2	2
P_a2/c	13.70	4	4	P_b2/c	13.71	4	4	P_c2/c	13.72	2	2	P_A2/c	13.73	4	2
P_C2/c	13.74	4	4	$P2_1/c$	14.75	2	2	$P2_1/c1'$	14.76	2	2	$P2_1'/c$	14.77	2	2
$P2_1/c'$	14.78	2	2	$P2_1'/c'$	14.79	2	2	P_a2_1/c	14.80	4	4	P_b2_1/c	14.81	4	4
P_c2_1/c	14.82	4	4	P_A2_1/c	14.83	4	2	P_C2_1/c	14.84	4	4	$C2/c$	15.85	2	2
$C2/c1'$	15.86	2	2	$C2'/c$	15.87	2	2	$C2/c'$	15.88	2	2	$C2'/c'$	15.89	2	2
C_c2/c	15.90	4	2	C_a2/c	15.91	4	4	$P222$	16.1	1	1	$P2221'$	16.2	1	1
$P2'2'2$	16.3	1	1	P_a222	16.4	2	2	P_C222	16.5	4	2	P_I222	16.6	2	2
$P222_1$	17.7	2	2	$P222_11'$	17.8	2	2	$P2'2'2_1$	17.9	2	2	$P22'2_1'$	17.10	2	2
P_a222_1	17.11	4	4	P_c222_1	17.12	2	2	P_B222_1	17.13	4	2	P_C222_1	17.14	4	4
P_I222_1	17.15	4	4	$P2_12_12$	18.16	2	2	$P2_12_121'$	18.17	2	2	$P2_1'2_1'2$	18.18	2	2
$P2_12_1'2'$	18.19	2	2	$P_b2_12_12$	18.20	4	4	$P_c2_12_12$	18.21	4	4	$P_B2_12_12$	18.22	4	4
$P_C2_12_12$	18.23	4	2	$P_I2_12_12$	18.24	2	2	$P2_12_12_1$	19.25	4	4	$P2_12_12_11'$	19.26	4	4
$P2_1'2_1'2_1$	19.27	4	4	$P_c2_12_12_1$	19.28	4	4	$P_C2_12_12_1$	19.29	4	4	$P_I2_12_12_1$	19.30	4	4
$C222_1$	20.31	2	2	$C222_11'$	20.32	2	2	$C2'2'2_1$	20.33	2	2	$C22'2_1'$	20.34	2	2
C_c222_1	20.35	4	2	C_a222_1	20.36	4	4	C_A222_1	20.37	2	2	$C222$	21.38	2	1
$C2221'$	21.39	2	1	$C2'2'2$	21.40	2	1	$C22'2'$	21.41	2	1	C_c222	21.42	4	2
C_a222	21.43	2	2	C_A222	21.44	2	2	$F222$	22.45	1	1	$F2221'$	22.46	1	1
$F2'2'2$	22.47	1	1	F_S222	22.48	2	2	$I222$	23.49	1	1	$I2221'$	23.50	1	1
$I2'2'2$	23.51	1	1	I_c222	23.52	4	2	$I2_12_12_1$	24.53	2	2	$I2_12_12_11'$	24.54	2	2
$I2_1'2_1'2_1$	24.55	2	2	$I_c2_12_12_1$	24.56	4	2	$Pmm2$	25.57	1	1	$Pmm21'$	25.58	1	1
$Pm'm2'$	25.59	1	1	$Pm'm'2$	25.60	1	1	P_cmm2	25.61	2	2	P_amm2	25.62	2	2
P_Cmm2	25.63	4	2	P_Amm2	25.64	2	2	P_Imm2	25.65	2	2	$Pmc2_1$	26.66	2	2
$Pmc2_11'$	26.67	2	2	$Pm'c2_1'$	26.68	2	2	$Pmc'2_1'$	26.69	2	2	$Pm'c'2_1$	26.70	2	2
P_amc2_1	26.71	4	4	P_bmc2_1	26.72	4	4	P_cmc2_1	26.73	2	2	P_Amc2_1	26.74	2	2
P_Bmc2_1	26.75	4	4	P_Cmc2_1	26.76	4	4	P_Imc2_1	26.77	4	4	$Pcc2$	27.78	2	2

$Pcc21'$	27.79	2	2	$Pc'c2'$	27.80	2	2	$Pc'c'2$	27.81	2	2	P_ccc2	27.82	2	2
P_acc2	27.83	4	4	P_Ccc2	27.84	4	4	P_Acc2	27.85	4	4	P_Icc2	27.86	4	4
$Pma2$	28.87	2	2	$Pma21'$	28.88	2	2	$Pm'a2'$	28.89	2	2	$Pma'2'$	28.90	2	2
$Pm'a'2$	28.91	2	2	P_ama2	28.92	2	2	P_bma2	28.93	4	4	P_cma2	28.94	4	4
P_Ama2	28.95	4	4	P_Bma2	28.96	4	4	P_Cma2	28.97	4	2	P_Ima2	28.98	4	4
$Pca2_1$	29.99	4	4	$Pca2_11'$	29.100	4	4	$Pc'a2_1'$	29.101	4	4	$Pca'2_1'$	29.102	4	4
$Pc'a'2_1$	29.103	4	4	P_aca2_1	29.104	4	4	P_bca2_1	29.105	8	8	P_cca2_1	29.106	4	4
P_Aca2_1	29.107	4	4	P_Bca2_1	29.108	4	4	P_Cca2_1	29.109	4	4	P_Ica2_1	29.110	4	4
$Pnc2$	30.111	2	2	$Pnc21'$	30.112	2	2	$Pn'c2'$	30.113	2	2	$Pnc'2'$	30.114	2	2
$Pn'c'2$	30.115	2	2	P_anc2	30.116	4	4	P_bnc2	30.117	4	4	P_cnc2	30.118	4	4
P_Anc2	30.119	2	2	P_Bnc2	30.120	4	4	P_Cnc2	30.121	4	4	P_Inc2	30.122	4	4
$Pmn2_1$	31.123	2	2	$Pmn2_11'$	31.124	2	2	$Pm'n2_1'$	31.125	2	2	$Pmn'2_1'$	31.126	2	2
$Pm'n'2_1$	31.127	2	2	P_amn2_1	31.128	4	4	P_bmn2_1	31.129	4	4	P_cmn2_1	31.130	4	4
P_Amn2_1	31.131	4	4	P_Bmn2_1	31.132	2	2	P_Cmn2_1	31.133	4	4	P_Imn2_1	31.134	2	2
$Pba2$	32.135	2	2	$Pba21'$	32.136	2	2	$Pb'a2'$	32.137	2	2	$Pb'a'2$	32.138	2	2
P_cba2	32.139	4	4	P_bba2	32.140	4	4	P_Cba2	32.141	4	2	P_Aba2	32.142	4	4
P_Iba2	32.143	4	4	$Pna2_1$	33.144	4	4	$Pna2_11'$	33.145	4	4	$Pn'a2_1'$	33.146	4	4
$Pna'2_1'$	33.147	4	4	$Pn'a'2_1$	33.148	4	4	P_ana2_1	33.149	4	4	P_bna2_1	33.150	8	8
P_cna2_1	33.151	4	4	P_Ana2_1	33.152	4	4	P_Bna2_1	33.153	4	4	P_Cna2_1	33.154	4	4
P_Ina2_1	33.155	4	4	$Pnn2$	34.156	2	2	$Pnn21'$	34.157	2	2	$Pn'n2'$	34.158	2	2
$Pn'n'2$	34.159	2	2	P_ann2	34.160	4	4	P_cnn2	34.161	4	4	P_Ann2	34.162	4	4
P_Cnn2	34.163	4	4	P_Inn2	34.164	2	2	$Cmm2$	35.165	2	1	$Cmm21'$	35.166	2	1
$Cm'm2'$	35.167	2	1	$Cm'm'2$	35.168	2	1	C_cmm2	35.169	4	2	C_amm2	35.170	2	2
C_Amm2	35.171	4	2	$Cmc2_1$	36.172	2	2	$Cmc2_11'$	36.173	2	2	$Cm'c2_1'$	36.174	2	2
$Cmc'2_1'$	36.175	2	2	$Cm'c'2_1$	36.176	2	2	C_cmc2_1	36.177	4	2	C_amc2_1	36.178	4	4
C_Amc2_1	36.179	4	2	$Ccc2$	37.180	2	2	$Ccc21'$	37.181	2	2	$Cc'c2'$	37.182	2	2
$Cc'c'2$	37.183	2	2	C_ccc2	37.184	4	2	C_acc2	37.185	4	4	C_Acc2	37.186	4	2
$Amm2$	38.187	1	1	$Amm21'$	38.188	1	1	$Am'm2'$	38.189	1	1	$Amm'2'$	38.190	1	1
$Am'm'2$	38.191	1	1	A_amm2	38.192	2	2	A_bmm2	38.193	2	2	A_Bmm2	38.194	4	2
$Abm2$	39.195	2	2	$Abm21'$	39.196	2	2	$Ab'm2'$	39.197	2	2	$Abm'2'$	39.198	2	2
$Ab'm'2$	39.199	2	2	A_abm2	39.200	4	4	A_bbm2	39.201	2	2	A_Bbm2	39.202	4	2
$Ama2$	40.203	2	2	$Ama21'$	40.204	2	2	$Am'a2'$	40.205	2	2	$Ama'2'$	40.206	2	2
$Am'a'2$	40.207	2	2	A_ama2	40.208	2	2	A_bma2	40.209	4	4	A_Bma2	40.210	4	2
$Aba2$	41.211	2	2	$Aba21'$	41.212	2	2	$Ab'a2'$	41.213	2	2	$Aba'2'$	41.214	2	2
$Ab'a'2$	41.215	2	2	A_aba2	41.216	4	4	A_bba2	41.217	4	4	A_Bba2	41.218	4	2
$Fmm2$	42.219	2	1	$Fmm21'$	42.220	2	1	$Fm'm2'$	42.221	2	1	$Fm'm'2$	42.222	2	1
F_Smm2	42.223	2	2	$Fdd2$	43.224	2	2	$Fdd21'$	43.225	2	2	$Fd'd2'$	43.226	2	2
$Fd'd'2$	43.227	2	2	F_Sdd2	43.228	4	4	$Imm2$	44.229	1	1	$Imm21'$	44.230	1	1
$Im'm2'$	44.231	1	1	$Im'm'2$	44.232	1	1	I_cmm2	44.233	4	2	I_amm2	44.234	2	2
$Iba2$	45.235	2	2	$Iba21'$	45.236	2	2	$Ib'a2'$	45.237	2	2	$Ib'a'2$	45.238	2	2
I_cba2	45.239	4	2	I_aba2	45.240	4	4	$Ima2$	46.241	2	2	$Ima21'$	46.242	2	2
$Im'a2'$	46.243	2	2	$Ima'2'$	46.244	2	2	$Im'a'2$	46.245	2	2	I_cma2	46.246	4	2
I_ama2	46.247	2	2	I_bma2	46.248	4	4	$Pmmm$	47.249	1	1	$Pmmm1'$	47.250	1	1
$Pm'mm$	47.251	1	1	$Pm'm'm$	47.252	1	1	$Pm'm'm'$	47.253	1	1	P_ammm	47.254	2	2
P_Cmmm	47.255	4	2	P_Immm	47.256	8	2	$Pnnn$	48.257	4	2	$Pnnn1'$	48.258	4	2
$Pn'nn$	48.259	4	2	$Pn'n'n$	48.260	4	2	$Pn'n'n'$	48.261	4	2	P_cnnn	48.262	8	4
P_Cnnn	48.263	4	4	P_Innn	48.264	8	2	$Pccm$	49.265	2	2	$Pccm1'$	49.266	2	2
$Pc'cm$	49.267	2	2	$Pccm'$	49.268	2	2	$Pc'c'm$	49.269	2	2	$Pc'cm'$	49.270	2	2
$Pc'c'm'$	49.271	2	2	P_accm	49.272	4	4	P_cccm	49.273	2	2	P_Bccm	49.274	4	4
P_Cccm	49.275	4	4	P_Iccm	49.276	8	4	$Pban$	50.277	4	2	$Pban1'$	50.278	4	2
$Pb'an$	50.279	4	2	$Pban'$	50.280	4	2	$Pb'a'n$	50.281	4	2	$Pb'an'$	50.282	4	2
$Pb'a'n'$	50.283	4	2	P_aban	50.284	4	4	P_cban	50.285	8	4	P_Aban	50.286	8	4
P_Cban	50.287	4	2	P_Iban	50.288	8	4	$Pmma$	51.289	2	2	$Pmma1'$	51.290	2	2
$Pm'ma$	51.291	2	2	$Pmm'a$	51.292	2	2	$Pmma'$	51.293	2	2	$Pm'm'a$	51.294	2	2
$Pmm'a'$	51.295	2	2	$Pm'ma'$	51.296	2	2	$Pm'm'a'$	51.297	2	2	P_amma	51.298	2	2
P_bmma	51.299	4	4	P_cmma	51.300	4	4	P_Amma	51.301	8	4	P_Bmma	51.302	4	2
P_Cmma	51.303	4	4	P_Imma	51.304	4	4	$Pnna$	52.305	4	4	$Pnna1'$	52.306	4	4
$Pn'na$	52.307	4	4	$Pnn'a$	52.308	4	4	$Pnna'$	52.309	4	4	$Pn'n'a$	52.310	4	4
$Pnn'a'$	52.311	4	4	$Pn'na'$	52.312	4	4	$Pn'n'a'$	52.313	4	4	P_anna	52.314	8	4
P_bnna	52.315	8	4	P_cnna	52.316	8	8	P_Anna	52.317	4	4	P_Bnna	52.318	8	4
P_Cnna	52.319	8	4	P_Inna	52.320	4	4	$Pmna$	53.321	4	2	$Pmna1'$	53.322	4	2
$Pm'na$	53.323	4	2	$Pmn'a$	53.324	4	2	$Pmna'$	53.325	4	2	$Pm'n'a$	53.326	4	2
$Pmn'a'$	53.327	4	2	$Pm'na'$	53.328	4	2	$Pm'n'a'$	53.329	4	2	P_amna	53.330	4	4
P_bmna	53.331	8	4	P_cmna	53.332	4	4	P_Amna	53.333	4	4	P_Bmna	53.334	4	2
P_Cmna	53.335	8	4	P_Imna	53.336	4	4	$Pcca$	54.337	4	4	$Pcca1'$	54.338	4	4
$Pc'ca$	54.339	4	4	$Pcc'a$	54.340	4	4	$Pcca'$	54.341	4	4	$Pc'c'a$	54.342	4	4
$Pcc'a'$	54.343	4	4	$Pc'ca'$	54.344	4	4	$Pc'c'a'$	54.345	4	4	P_acca	54.346	4	4
P_bcca	54.347	8	8	P_ccca	54.348	4	4	P_Acca	54.349	8	4	P_Bcca	54.350	4	4
P_Ccca	54.351	8	4	P_Icca	54.352	8	8	$Pbam$	55.353	2	2	$Pbam1'$	55.354	2	2
$Pb'am$	55.355	2	2	$Pbam'$	55.356	2	2	$Pb'a'm$	55.357	2	2	$Pb'am'$	55.358	2	2
$Pb'a'm'$	55.359	2	2	P_abam	55.360	4	4	P_cbam	55.361	4	4	P_Abam	55.362	8	4
P_Cbam	55.363	4	2	P_Ibam	55.364	8	4	$Pccn$	56.365	4	4	$Pccn1'$	56.366	4	4
$Pc'cn$	56.367	4	4	$Pccn'$	56.368	4	4	$Pc'c'n$	56.369	4	4	$Pc'cn'$	56.370	4	4
$Pc'c'n'$	56.371	4	4	P_bccn	56.372	8	8	P_cccn	56.373	8	4	P_Accn	56.374	8	4
P_Cccn	56.375	4	4	P_Iccn	56.376	8	4	$Pbcm$	57.377	4	4	$Pbcm1'$	57.378	4	4
$Pb'cm$	57.379	4	4	$Pbc'm$	57.380	4	4	$Pbcm'$	57.381	4	4	$Pb'c'm$	57.382	4	4
$Pbc'm'$	57.383	4	4	$Pb'cm'$	57.384	4	4	$Pb'c'm'$	57.385	4	4	P_abcm	57.386	8	8
P_bbcm	57.387	4	4	P_cbcm	57.388	4	4	P_Abcm	57.389	4	4	P_Bbcm	57.390	8	4
P_Cbcm	57.391	8	4	P_Ibcm	57.392	8	4	$Pnnm$	58.393	2	2	$Pnnm1'$	58.394	2	2
$Pn'nm$	58.395	2	2	$Pnnm'$	58.396	2	2	$Pn'n'm$	58.397	2	2	$Pnn'm'$	58.398	2	2

$Pn'n'm'$	58.399	2	2	P_annm	58.400	8	4	P_cnnm	58.401	4	4	P_Bnnm	58.402	8	4
P_Cnnm	58.403	4	4	P_Innm	58.404	8	2	$Pmmn$	59.405	4	2	$Pmmn1'$	59.406	4	2
$Pm'mn$	59.407	4	2	$Pmmn'$	59.408	4	2	$Pm'm'n$	59.409	4	2	$Pmm'n'$	59.410	4	2
$Pm'm'n'$	59.411	4	2	P_bmmn	59.412	4	4	P_cmmn	59.413	8	4	P_Bmmn	59.414	8	4
P_Cmmn	59.415	4	2	P_Immn	59.416	8	2	$Pbcn$	60.417	4	4	$Pbcn1'$	60.418	4	4
$Pb'cn$	60.419	4	4	$Pbc'n$	60.420	4	4	$Pbcn'$	60.421	4	4	$Pb'c'n$	60.422	4	4
$Pbc'n'$	60.423	4	4	$Pb'cn'$	60.424	4	4	$Pb'c'n'$	60.425	4	4	P_abcn	60.426	8	8
P_bbcn	60.427	8	8	P_cbcn	60.428	8	4	P_Abcn	60.429	8	4	P_Bbcn	60.430	8	4
P_Cbcn	60.431	8	4	P_Ibcn	60.432	8	4	$Pbca$	61.433	4	4	$Pbca1'$	61.434	4	4
$Pb'ca$	61.435	4	4	$Pb'c'a$	61.436	4	4	$Pb'c'a'$	61.437	4	4	P_abca	61.438	8	8
P_Cbca	61.439	8	4	P_Ibca	61.440	8	8	$Pnma$	62.441	4	4	$Pnma1'$	62.442	4	4
$Pn'ma$	62.443	4	4	$Pnm'a$	62.444	4	4	$Pnma'$	62.445	4	4	$Pn'm'a$	62.446	4	4
$Pnm'a'$	62.447	4	4	$Pn'ma'$	62.448	4	4	$Pn'm'a'$	62.449	4	4	P_anma	62.450	8	4
P_bnma	62.451	4	4	P_cnma	62.452	8	8	P_Anma	62.453	8	4	P_Bnma	62.454	8	4
P_Cnma	62.455	8	4	P_Inma	62.456	4	4	$Cmcm$	63.457	4	2	$Cmcm1'$	63.458	4	2
$Cm'cm$	63.459	4	2	$Cmc'm$	63.460	4	2	$Cmcm'$	63.461	4	2	$Cm'c'm$	63.462	4	2
$Cmc'm'$	63.463	4	2	$Cm'cm'$	63.464	4	2	$Cm'c'm'$	63.465	4	2	C_cmcm	63.466	4	2
C_amcm	63.467	4	4	C_Amcm	63.468	4	2	$Cmca$	64.469	4	2	$Cmca1'$	64.470	4	2
$Cm'ca$	64.471	4	2	$Cmc'a$	64.472	4	2	$Cmca'$	64.473	4	2	$Cm'c'a$	64.474	4	2
$Cmc'a'$	64.475	4	2	$Cm'ca'$	64.476	4	2	$Cm'c'a'$	64.477	4	2	C_cmca	64.478	4	4
C_amca	64.479	4	4	C_Amca	64.480	4	2	$Cmmm$	65.481	2	1	$Cmmm1'$	65.482	2	1
$Cm'mm$	65.483	2	1	$Cmmm'$	65.484	2	1	$Cm'm'm$	65.485	2	1	$Cmm'm'$	65.486	2	1
$Cm'm'm'$	65.487	2	1	C_cmmm	65.488	4	2	C_ammm	65.489	2	2	C_Ammm	65.490	4	2
$Cccm$	66.491	2	2	$Cccm1'$	66.492	2	2	$Cc'cm$	66.493	2	2	$Cccm'$	66.494	2	2
$Cc'c'm$	66.495	2	2	$Ccc'm'$	66.496	2	2	$Cc'c'm'$	66.497	2	2	C_cccm	66.498	4	2
C_accm	66.499	4	4	C_Accm	66.500	4	2	$Cmma$	67.501	2	2	$Cmma1'$	67.502	2	2
$Cm'ma$	67.503	2	2	$Cmma'$	67.504	2	2	$Cm'm'a$	67.505	2	2	$Cmm'a'$	67.506	2	2
$Cm'm'a'$	67.507	2	2	C_cmma	67.508	4	4	C_amma	67.509	2	2	C_Amma	67.510	4	2
$Ccca$	68.511	4	2	$Ccca1'$	68.512	4	2	$Cc'ca$	68.513	4	2	$Ccca'$	68.514	4	2
$Cc'c'a$	68.515	4	2	$Ccc'a'$	68.516	4	2	$Cc'c'a'$	68.517	4	2	C_ccca	68.518	4	4
C_acca	68.519	4	4	C_Acca	68.520	4	2	$Fmmm$	69.521	2	1	$Fmmm1'$	69.522	2	1
$Fm'mm$	69.523	2	1	$Fm'm'm$	69.524	2	1	$Fm'm'm'$	69.525	2	1	F_Smmm	69.526	2	2
$Fddd$	70.527	4	2	$Fddd1'$	70.528	4	2	$Fd'dd$	70.529	4	2	$Fd'd'd$	70.530	4	2
$Fd'd'd'$	70.531	4	2	F_Sddd	70.532	8	4	$Immm$	71.533	4	1	$Immm1'$	71.534	4	1
$Im'mm$	71.535	4	1	$Im'm'm$	71.536	4	1	$Im'm'm'$	71.537	4	1	I_cmmm	71.538	4	2
$Ibam$	72.539	4	2	$Ibam1'$	72.540	4	2	$Ib'am$	72.541	4	2	$Ibam'$	72.542	4	2
$Ib'a'm$	72.543	4	2	$Iba'm'$	72.544	4	2	$Ib'a'm'$	72.545	4	2	I_cbam	72.546	4	2
I_bbam	72.547	4	4	$Ibca$	73.548	4	4	$Ibca1'$	73.549	4	4	$Ib'ca$	73.550	4	4
$Ib'c'a$	73.551	4	4	$Ib'c'a'$	73.552	4	4	I_cbca	73.553	4	4	$Imma$	74.554	2	2
$Imma1'$	74.555	2	2	$Im'ma$	74.556	2	2	$Imma'$	74.557	2	2	$Im'm'a$	74.558	2	2
$Imm'a'$	74.559	2	2	$Im'm'a'$	74.560	2	2	I_cmma	74.561	4	4	I_bmma	74.562	4	2
$P4$	75.1	2	1	$P41'$	75.2	2	1	$P4'$	75.3	2	1	P_c4	75.4	4	2
P_C4	75.5	4	2	P_I4	75.6	4	2	$P4_1$	76.7	4	4	$P4_11'$	76.8	4	4
$P4_1'$	76.9	4	4	P_c4_1	76.10	4	4	P_C4_1	76.11	8	8	P_I4_1	76.12	4	4
$P4_2$	77.13	2	2	$P4_21'$	77.14	2	2	$P4_2'$	77.15	2	2	P_c4_2	77.16	4	2
P_C4_2	77.17	4	4	P_I4_2	77.18	4	2	$P4_3$	78.19	4	4	$P4_31'$	78.20	4	4
$P4_3'$	78.21	4	4	P_c4_3	78.22	4	4	P_C4_3	78.23	8	8	P_I4_3	78.24	4	4
$I4$	79.25	2	1	$I41'$	79.26	2	1	$I4'$	79.27	2	1	I_c4	79.28	4	2
$I4_1$	80.29	2	2	$I4_11'$	80.30	2	2	$I4_1'$	80.31	2	2	I_c4_1	80.32	4	4
$P4$	81.33	2	1	$P41'$	81.34	2	1	$P4'$	81.35	2	1	P_c4	81.36	4	2
P_C4	81.37	4	2	P_I4	81.38	4	2	$I4$	82.39	1	1	$I41'$	82.40	2	1
$I4'$	82.41	2	1	I_c4	82.42	4	2	$P4/m$	83.43	2	1	$P4/m1'$	83.44	2	1
$P4'/m$	83.45	2	1	$P4/m'$	83.46	2	1	$P4'/m'$	83.47	2	1	P_c4/m	83.48	4	2
P_C4/m	83.49	4	2	P_I4/m	83.50	8	2	$P4_2/m$	84.51	2	2	$P4_2/m1'$	84.52	2	2
$P4_2'/m$	84.53	2	2	$P4_2/m'$	84.54	2	2	$P4_2'/m'$	84.55	2	2	P_c4_2/m	84.56	4	2
P_C4_2/m	84.57	4	4	P_I4_2/m	84.58	8	2	$P4/n$	85.59	4	2	$P4/n1'$	85.60	4	2
$P4'/n$	85.61	4	2	$P4/n'$	85.62	4	2	$P4'/n'$	85.63	4	2	P_c4/n	85.64	8	4
P_C4/n	85.65	4	2	P_I4/n	85.66	8	2	$P4_2/n$	86.67	4	2	$P4_2/n1'$	86.68	4	2
$P4_2'/n$	86.69	4	2	$P4_2/n'$	86.70	4	2	$P4_2'/n'$	86.71	4	2	P_c4_2/n	86.72	8	4
P_C4_2/n	86.73	4	4	P_I4_2/n	86.74	8	2	$I4/m$	87.75	4	1	$I4/m1'$	87.76	4	1
$I4'/m$	87.77	4	1	$I4/m'$	87.78	4	1	$I4'/m'$	87.79	4	1	I_c4/m	87.80	4	2
$I4_1/a$	88.81	4	2	$I4_1/a1'$	88.82	4	2	$I4_1'/a$	88.83	4	2	$I4_1/a'$	88.84	4	2
$I4_1'/a'$	88.85	4	2	I_c4_1/a	88.86	8	4	$P422$	89.87	2	1	$P4221'$	89.88	2	1
$P4'22'$	89.89	2	1	$P42'2'$	89.90	2	1	$P4'2'2$	89.91	2	1	P_c422	89.92	4	2
P_C422	89.93	4	2	P_I422	89.94	4	2	$P42_12$	90.95	2	2	$P42_121'$	90.96	4	2
$P4'2_12'$	90.97	4	2	$P42_1'2'$	90.98	2	2	$P4'2_1'2$	90.99	4	2	P_c42_12	90.100	4	4
P_C42_12	90.101	4	2	P_I42_12	90.102	4	2	$P4_122$	91.103	4	4	$P4_1221'$	91.104	4	4
$P4_1'22'$	91.105	4	4	$P4_12'2'$	91.106	4	4	$P4_1'2'2$	91.107	4	4	P_c4_122	91.108	4	4
P_C4_122	91.109	8	8	P_I4_122	91.110	8	4	$P4_12_12$	92.111	4	4	$P4_12_121'$	92.112	4	4
$P4_1'2_12'$	92.113	4	4	$P4_12_1'2'$	92.114	4	4	$P4_1'2_1'2$	92.115	4	4	$P_c4_12_12$	92.116	8	4
$P_C4_12_12$	92.117	8	8	$P_I4_12_12$	92.118	8	4	$P4_222$	93.119	2	2	$P4_2221'$	93.120	2	2
$P4_2'22'$	93.121	2	2	$P4_22'2'$	93.122	2	2	$P4_2'2'2$	93.123	2	2	P_c4_222	93.124	4	2
P_C4_222	93.125	4	4	P_I4_222	93.126	4	2	$P4_22_12$	94.127	4	2	$P4_22_121'$	94.128	4	2
$P4_2'2_12'$	94.129	4	2	$P4_22_1'2'$	94.130	4	2	$P4_2'2_1'2$	94.131	4	2	$P_c4_22_12$	94.132	8	4
$P_C4_22_12$	94.133	4	4	$P_I4_22_12$	94.134	4	2	$P4_322$	95.135	4	4	$P4_3221'$	95.136	4	4
$P4_3'22'$	95.137	4	4	$P4_32'2'$	95.138	4	4	$P4_3'2'2$	95.139	4	4	P_c4_322	95.140	4	4

P_C4_322	95.141	8	8	P_I4_322	95.142	8	4	$P4_32_12$	96.143	4	4	$P4_32_121'$	96.144	4	4
$P4'_32_12'$	96.145	4	4	$P4_32'_12'$	96.146	4	4	$P4'_32'_12$	96.147	4	4	$P_c4_32_12$	96.148	8	4
$P_C4_32_12$	96.149	8	8	$P_I4_32_12$	96.150	8	4	$I422$	97.151	2	1	$I4221'$	97.152	2	1
$I4'22'$	97.153	2	1	$I42'2'$	97.154	2	1	$I4'2'2$	97.155	2	1	I_c422	97.156	4	2
$I4_122$	98.157	4	2	$I4_1221'$	98.158	4	2	$I4'_122'$	98.159	4	2	$I4_12'2'$	98.160	4	2
$I4'_12'2$	98.161	4	2	I_c4_122	98.162	4	4	$P4mm$	99.163	2	1	$P4mm1'$	99.164	2	1
$P4'm'm$	99.165	2	1	$P4'mm'$	99.166	2	1	$P4m'm'$	99.167	2	1	P_c4mm	99.168	4	2
P_C4mm	99.169	4	2	P_I4mm	99.170	4	2	$P4bm$	100.171	2	2	$P4bm1'$	100.172	4	2
$P4'b'm$	100.173	4	2	$P4'bm'$	100.174	4	2	$P4b'm'$	100.175	2	2	P_c4bm	100.176	4	4
P_C4bm	100.177	4	2	P_I4bm	100.178	4	4	$P4_2cm$	101.179	4	2	$P4_2cm1'$	101.180	4	2
$P4'_2c'm$	101.181	4	2	$P4'_2cm'$	101.182	4	2	$P4_2c'm'$	101.183	4	2	P_c4_2cm	101.184	4	2
P_C4_2cm	101.185	4	4	P_I4_2cm	101.186	8	4	$P4_2nm$	102.187	4	2	$P4_2nm1'$	102.188	4	2
$P4'_2n'm$	102.189	4	2	$P4'_2nm'$	102.190	4	2	$P4_2n'm'$	102.191	4	2	P_c4_2nm	102.192	8	4
P_C4_2nm	102.193	4	4	P_I4_2nm	102.194	4	2	$P4cc$	103.195	4	2	$P4cc1'$	103.196	4	2
$P4'c'c$	103.197	4	2	$P4'cc'$	103.198	4	2	$P4c'c'$	103.199	4	2	P_c4cc	103.200	4	2
P_C4cc	103.201	8	4	P_I4cc	103.202	4	4	$P4nc$	104.203	4	2	$P4nc1'$	104.204	4	2
$P4'n'c$	104.205	4	2	$P4'nc'$	104.206	4	2	$P4n'c'$	104.207	4	2	P_c4nc	104.208	4	4
P_C4nc	104.209	8	4	P_I4nc	104.210	4	2	$P4_2mc$	105.211	2	2	$P4_2mc1'$	105.212	2	2
$P4'_2m'c$	105.213	2	2	$P4'_2mc'$	105.214	2	2	$P4_2m'c'$	105.215	2	2	P_c4_2mc	105.216	4	2
P_C4_2mc	105.217	8	4	P_I4_2mc	105.218	4	2	$P4_2bc$	106.219	4	4	$P4_2bc1'$	106.220	4	4
$P4'_2b'c$	106.221	4	4	$P4'_2bc'$	106.222	4	4	$P4_2b'c'$	106.223	4	4	P_c4_2bc	106.224	8	4
P_C4_2bc	106.225	8	4	P_I4_2bc	106.226	8	4	$I4mm$	107.227	2	1	$I4mm1'$	107.228	2	1
$I4'm'm$	107.229	2	1	$I4'mm'$	107.230	2	1	$I4m'm'$	107.231	2	1	I_c4mm	107.232	4	2
$I4cm$	108.233	2	2	$I4cm1'$	108.234	4	2	$I4'c'm$	108.235	4	2	$I4'cm'$	108.236	4	2
$I4c'm'$	108.237	2	2	I_c4cm	108.238	4	2	$I4_1md$	109.239	2	2	$I4_1md1'$	109.240	2	2
$I4'_1m'd$	109.241	2	2	$I4'_1md'$	109.242	2	2	$I4_1m'd'$	109.243	2	2	I_c4_1md	109.244	8	4
$I4_1cd$	110.245	4	4	$I4_1cd1'$	110.246	4	4	$I4'_1c'd$	110.247	4	4	$I4'_1cd'$	110.248	4	4
$I4_1c'd'$	110.249	4	4	I_c4_1cd	110.250	8	4	$P\bar{4}2m$	111.251	2	1	$P\bar{4}2m1'$	111.252	2	1
$P\bar{4}'2'm$	111.253	2	1	$P\bar{4}'2m'$	111.254	2	1	$P\bar{4}2'm'$	111.255	2	1	$P_c\bar{4}2m$	111.256	4	2
$P_C\bar{4}2m$	111.257	4	2	$P_I\bar{4}2m$	111.258	4	2	$P\bar{4}2c$	112.259	2	2	$P\bar{4}2c1'$	112.260	2	2
$P\bar{4}'2'c$	112.261	2	2	$P\bar{4}'2c'$	112.262	2	2	$P\bar{4}2'c'$	112.263	2	2	$P_c\bar{4}2c$	112.264	4	2
$P_C\bar{4}2c$	112.265	8	4	$P_I\bar{4}2c$	112.266	4	2	$P\bar{4}2_1m$	113.267	2	2	$P\bar{4}2_1m1'$	113.268	4	2
$P\bar{4}'2'_1m$	113.269	4	2	$P\bar{4}'2_1m'$	113.270	4	2	$P\bar{4}2'_1m'$	113.271	2	2	$P_c\bar{4}2_1m$	113.272	4	4
$P_C\bar{4}2_1m$	113.273	4	2	$P_I\bar{4}2_1m$	113.274	4	2	$P\bar{4}2_1c$	114.275	4	2	$P\bar{4}2_1c1'$	114.276	4	2
$P\bar{4}'2'_1c$	114.277	4	2	$P\bar{4}'2_1c'$	114.278	4	2	$P\bar{4}2'_1c'$	114.279	4	2	$P_c\bar{4}2_1c$	114.280	4	4
$P_C\bar{4}2_1c$	114.281	8	4	$P_I\bar{4}2_1c$	114.282	4	2	$P\bar{4}m2$	115.283	2	1	$P\bar{4}m21'$	115.284	2	1
$P\bar{4}'m'2$	115.285	2	1	$P\bar{4}'m2'$	115.286	2	1	$P\bar{4}m'2'$	115.287	2	1	$P_c\bar{4}m2$	115.288	4	2
$P_C\bar{4}m2$	115.289	4	2	$P_I\bar{4}m2$	115.290	4	2	$P\bar{4}c2$	116.291	4	2	$P\bar{4}c21'$	116.292	4	2
$P\bar{4}'c'2$	116.293	4	2	$P\bar{4}'c2'$	116.294	4	2	$P\bar{4}c'2'$	116.295	4	2	$P_c\bar{4}c2$	116.296	4	2
$P_C\bar{4}c2$	116.297	4	4	$P_I\bar{4}c2$	116.298	4	4	$P\bar{4}b2$	117.299	2	2	$P\bar{4}b21'$	117.300	4	2
$P\bar{4}'b'2$	117.301	4	2	$P\bar{4}'b2'$	117.302	4	2	$P\bar{4}b'2'$	117.303	2	2	$P_c\bar{4}b2$	117.304	4	4
$P_C\bar{4}b2$	117.305	4	2	$P_I\bar{4}b2$	117.306	4	4	$P\bar{4}n2$	118.307	2	2	$P\bar{4}n21'$	118.308	4	2
$P\bar{4}'n'2$	118.309	4	2	$P\bar{4}'n2'$	118.310	4	2	$P\bar{4}n'2'$	118.311	2	2	$P_c\bar{4}n2$	118.312	4	4
$P_C\bar{4}n2$	118.313	4	4	$P_I\bar{4}n2$	118.314	4	2	$I\bar{4}m2$	119.315	2	1	$I\bar{4}m21'$	119.316	2	1
$I\bar{4}'m'2$	119.317	2	1	$I\bar{4}'m2'$	119.318	2	1	$I\bar{4}m'2'$	119.319	1	1	$I_c\bar{4}m2$	119.320	4	2
$I\bar{4}c2$	120.321	2	2	$I\bar{4}c21'$	120.322	2	2	$I\bar{4}'c'2$	120.323	2	2	$I\bar{4}'c2'$	120.324	2	2
$I\bar{4}c'2'$	120.325	2	2	$I_c\bar{4}c2$	120.326	4	2	$I\bar{4}2m$	121.327	2	1	$I\bar{4}2m1'$	121.328	2	1
$I\bar{4}'2'm$	121.329	2	1	$I\bar{4}'2m'$	121.330	2	1	$I\bar{4}2'm'$	121.331	2	1	$I_c\bar{4}2m$	121.332	4	2
$I\bar{4}2d$	122.333	4	2	$I\bar{4}2d1'$	122.334	4	2	$I\bar{4}'2'd$	122.335	4	2	$I\bar{4}'2d'$	122.336	4	2
$I\bar{4}2'd'$	122.337	4	2	$I_c\bar{4}2d$	122.338	4	4	$P4/mmm$	123.339	2	1	$P4/mmm1'$	123.340	2	1
$P4/m'mm$	123.341	2	1	$P4'/mm'm$	123.342	2	1	$P4'/mmm'$	123.343	2	1	$P4'/m'm'm$	123.344	2	1
$P4/mm'm'$	123.345	2	1	$P4'/m'mm'$	123.346	2	1	$P4/m'm'm'$	123.347	2	1	P_c4/mmm	123.348	4	2
P_C4/mmm	123.349	4	2	P_I4/mmm	123.350	8	2	$P4/mcc$	124.351	4	2	$P4/mcc1'$	124.352	4	2
$P4/m'cc$	124.353	4	2	$P4'/mc'c$	124.354	4	2	$P4'/mcc'$	124.355	4	2	$P4'/m'c'c$	124.356	4	2
$P4/mc'c'$	124.357	4	2	$P4'/m'cc'$	124.358	4	2	$P4/m'c'c'$	124.359	4	2	P_c4/mcc	124.360	4	2
P_C4/mcc	124.361	8	4	P_I4/mcc	124.362	8	4	$P4/nbm$	125.363	4	2	$P4/nbm1'$	125.364	4	2
$P4/n'bm$	125.365	4	2	$P4'/nb'm$	125.366	4	2	$P4'/nbm'$	125.367	4	2	$P4'/n'b'm$	125.368	4	2
$P4/nb'm'$	125.369	4	2	$P4'/n'bm'$	125.370	4	2	$P4/n'b'm'$	125.371	4	2	P_c4/nbm	125.372	8	4
P_C4/nbm	125.373	4	2	P_I4/nbm	125.374	8	4	$P4/nnc$	126.375	8	2	$P4/nnc1'$	126.376	8	2
$P4/n'nc$	126.377	8	2	$P4'/nn'c$	126.378	8	2	$P4'/nnc'$	126.379	8	2	$P4'/n'n'c$	126.380	8	2
$P4/nn'c'$	126.381	8	2	$P4'/n'nc'$	126.382	8	2	$P4/n'n'c'$	126.383	8	2	P_c4/nnc	126.384	8	4
P_C4/nnc	126.385	8	4	P_I4/nnc	126.386	8	2	$P4/mbm$	127.387	2	2	$P4/mbm1'$	127.388	4	2
$P4/m'bm$	127.389	4	2	$P4'/mb'm$	127.390	4	2	$P4'/mbm'$	127.391	4	2	$P4'/m'b'm$	127.392	4	2
$P4/mb'm'$	127.393	2	2	$P4'/m'bm'$	127.394	4	2	$P4/m'b'm'$	127.395	4	2	P_c4/mbm	127.396	4	4
P_C4/mbm	127.397	4	2	P_I4/mbm	127.398	8	4	$P4/mnc$	128.399	4	2	$P4/mnc1'$	128.400	4	2
$P4/m'nc$	128.401	4	2	$P4'/mn'c$	128.402	4	2	$P4'/mnc'$	128.403	4	2	$P4'/m'n'c$	128.404	4	2
$P4/mn'c'$	128.405	4	2	$P4'/m'nc'$	128.406	4	2	$P4/m'n'c'$	128.407	4	2	P_c4/mnc	128.408	4	4
P_C4/mnc	128.409	8	4	P_I4/mnc	128.410	8	2	$P4/nmm$	129.411	4	2	$P4/nmm1'$	129.412	4	2
$P4/n'mm$	129.413	4	2	$P4'/nm'm$	129.414	4	2	$P4'/nmm'$	129.415	4	2	$P4'/n'm'm$	129.416	4	2
$P4/nm'm'$	129.417	4	2	$P4'/n'mm'$	129.418	4	2	$P4/n'm'm'$	129.419	4	2	P_c4/nmm	129.420	8	4
P_C4/nmm	129.421	4	2	P_I4/nmm	129.422	8	2	$P4/ncc$	130.423	8	4	$P4/ncc1'$	130.424	8	4
$P4/n'cc$	130.425	8	4	$P4'/nc'c$	130.426	8	4	$P4'/ncc'$	130.427	8	4	$P4'/n'c'c$	130.428	8	4
$P4/nc'c'$	130.429	8	4	$P4'/n'cc'$	130.430	8	4	$P4/n'c'c'$	130.431	8	4	P_c4/ncc	130.432	8	4

P_C4/ncc	130.433	8	4	P_I4/ncc	130.434	8	4	$P4_2/mmc$	131.435	2	2	$P4_2/mmc1'$	131.436	2	2
$P4_2/m'mc$	131.437	2	2	$P4_2'/mm'c$	131.438	2	2	$P4_2'/mmc'$	131.439	2	2	$P4_2'/m'm'c$	131.440	2	2
$P4_2/mm'c'$	131.441	2	2	$P4_2'/m'mc'$	131.442	2	2	$P4_2/m'm'c'$	131.443	2	2	P_c4_2/mmc	131.444	4	2
P_C4_2/mmc	131.445	8	4	P_I4_2/mmc	131.446	8	2	$P4_2/mcm$	132.447	4	2	$P4_2/mcm1'$	132.448	4	2
$P4_2/m'cm$	132.449	4	2	$P4_2'/mc'm$	132.450	4	2	$P4_2'/mcm'$	132.451	4	2	$P4_2'/m'c'm$	132.452	4	2
$P4_2/mc'm'$	132.453	4	2	$P4_2'/m'cm'$	132.454	4	2	$P4_2/m'c'm'$	132.455	4	2	P_c4_2/mcm	132.456	4	2
P_C4_2/mcm	132.457	4	4	P_I4_2/mcm	132.458	8	4	$P4_2/nbc$	133.459	8	4	$P4_2/nbc1'$	133.460	8	4
$P4_2/n'bc$	133.461	8	4	$P4_2'/nb'c$	133.462	8	4	$P4_2'/nbc'$	133.463	8	4	$P4_2'/n'b'c$	133.464	8	4
$P4_2/nb'c'$	133.465	8	4	$P4_2'/n'bc'$	133.466	8	4	$P4_2/n'b'c'$	133.467	8	4	P_c4_2/nbc	133.468	8	4
P_C4_2/nbc	133.469	8	4	P_I4_2/nbc	133.470	8	4	$P4_2/nnm$	134.471	4	2	$P4_2/nnm1'$	134.472	4	2
$P4_2/n'nm$	134.473	4	2	$P4_2'/nn'm$	134.474	4	2	$P4_2'/nnm'$	134.475	4	2	$P4_2'/n'n'm$	134.476	4	2
$P4_2/nn'm'$	134.477	4	2	$P4_2'/n'nm'$	134.478	4	2	$P4_2/n'n'm'$	134.479	4	2	P_c4_2/nnm	134.480	8	4
P_C4_2/nnm	134.481	4	4	P_I4_2/nnm	134.482	8	2	$P4_2/mbc$	135.483	4	4	$P4_2/mbc1'$	135.484	4	4
$P4_2/m'bc$	135.485	4	4	$P4_2'/mb'c$	135.486	4	4	$P4_2'/mbc'$	135.487	4	4	$P4_2'/m'b'c$	135.488	4	4
$P4_2/mb'c'$	135.489	4	4	$P4_2'/m'bc'$	135.490	4	4	$P4_2/m'b'c'$	135.491	4	4	P_c4_2/mbc	135.492	8	4
P_C4_2/mbc	135.493	8	4	P_I4_2/mbc	135.494	8	4	$P4_2/mnm$	136.495	4	2	$P4_2/mnm1'$	136.496	4	2
$P4_2/m'nm$	136.497	4	2	$P4_2'/mn'm$	136.498	4	2	$P4_2'/mnm'$	136.499	4	2	$P4_2'/m'n'm$	136.500	4	2
$P4_2/mn'm'$	136.501	4	2	$P4_2'/m'nm'$	136.502	4	2	$P4_2/m'n'm'$	136.503	4	2	P_c4_2/mnm	136.504	8	4
P_C4_2/mnm	136.505	4	4	P_I4_2/mnm	136.506	8	2	$P4_2/nmc$	137.507	8	2	$P4_2/nmc1'$	137.508	8	2
$P4_2/n'mc$	137.509	8	2	$P4_2'/nm'c$	137.510	8	2	$P4_2'/nmc'$	137.511	8	2	$P4_2'/n'm'c$	137.512	8	2
$P4_2/nm'c'$	137.513	8	2	$P4_2'/n'mc'$	137.514	8	2	$P4_2/n'm'c'$	137.515	8	2	P_c4_2/nmc	137.516	8	4
P_C4_2/nmc	137.517	8	4	P_I4_2/nmc	137.518	8	2	$P4_2/ncm$	138.519	4	4	$P4_2/ncm1'$	138.520	4	4
$P4_2/n'cm$	138.521	4	4	$P4_2'/nc'm$	138.522	4	4	$P4_2'/ncm'$	138.523	4	4	$P4_2'/n'c'm$	138.524	4	4
$P4_2/nc'm'$	138.525	4	4	$P4_2'/n'cm'$	138.526	4	4	$P4_2/n'c'm'$	138.527	4	4	P_c4_2/ncm	138.528	8	4
P_C4_2/ncm	138.529	4	4	P_I4_2/ncm	138.530	8	4	$I4/mmm$	139.531	4	1	$I4/mmm1'$	139.532	4	1
$I4/m'mm$	139.533	4	1	$I4'/mm'm$	139.534	4	1	$I4'/mmm'$	139.535	4	1	$I4'/m'm'm$	139.536	4	1
$I4/mm'm'$	139.537	4	1	$I4'/m'mm'$	139.538	4	1	$I4/m'm'm'$	139.539	4	1	I_c4/mmm	139.540	4	2
$I4/mcm$	140.541	4	2	$I4/mcm1'$	140.542	4	2	$I4/m'cm$	140.543	4	2	$I4'/mc'm$	140.544	4	2
$I4'/mcm'$	140.545	4	2	$I4'/m'c'm$	140.546	4	2	$I4/mc'm'$	140.547	4	2	$I4'/m'cm'$	140.548	4	2
$I4/m'c'm'$	140.549	4	2	I_c4/mcm	140.550	4	2	$I4_1/amd$	141.551	4	2	$I4_1/amd1'$	141.552	4	2
$I4_1/a'md$	141.553	4	2	$I4_1'/am'd$	141.554	4	2	$I4_1'/amd'$	141.555	4	2	$I4_1'/a'm'd$	141.556	4	2
$I4_1/am'd'$	141.557	4	2	$I4_1'/a'md'$	141.558	4	2	$I4_1/a'm'd'$	141.559	4	2	I_c4_1/amd	141.560	8	4
$I4_1/acd$	142.561	8	4	$I4_1/acd1'$	142.562	8	4	$I4_1/a'cd$	142.563	8	4	$I4_1'/ac'd$	142.564	8	4
$I4_1'/acd'$	142.565	8	4	$I4_1'/a'c'd$	142.566	8	4	$I4_1/ac'd'$	142.567	8	4	$I4_1'/a'cd'$	142.568	8	4
$I4_1/a'c'd'$	142.569	8	4	I_c4_1/acd	142.570	8	4	$P3$	143.1	1	1	$P31'$	143.2	2	1
P_c3	143.3	2	2	$P3_1$	144.4	3	3	$P3_11'$	144.5	3	3	P_c3_1	144.6	6	6
$P3_2$	145.7	3	3	$P3_21'$	145.8	3	3	P_c3_2	145.9	6	6	$R3$	146.10	1	1
$R31'$	146.11	2	1	R_I3	146.12	2	2	$P3$	147.13	3	1	$P31'$	147.14	4	1
$P3'$	147.15	3	1	P_c3	147.16	6	2	$R3$	148.17	3	1	$R31'$	148.18	3	1
$R3'$	148.19	3	1	R_I3	148.20	6	2	$P312$	149.21	2	1	$P3121'$	149.22	2	1
$P312'$	149.23	1	1	P_c312	149.24	2	2	$P321$	150.25	2	1	$P3211'$	150.26	4	1
$P32'1$	150.27	2	1	P_c321	150.28	4	2	$P3_112$	151.29	3	3	$P3_1121'$	151.30	3	3
$P3_112'$	151.31	3	3	P_c3_112	151.32	6	6	$P3_121$	152.33	3	3	$P3_1211'$	152.34	3	3
$P3_12'1$	152.35	3	3	P_c3_121	152.36	6	6	$P3_212$	153.37	3	3	$P3_2121'$	153.38	3	3
$P3_212'$	153.39	3	3	P_c3_212	153.40	6	6	$P3_221$	154.41	3	3	$P3_2211'$	154.42	3	3
$P3_22'1$	154.43	3	3	P_c3_221	154.44	6	6	$R32$	155.45	2	1	$R321'$	155.46	2	1
$R32'$	155.47	1	1	R_I32	155.48	2	2	$P3m1$	156.49	2	1	$P3m11'$	156.50	2	1
$P3m'1$	156.51	1	1	P_c3m1	156.52	4	2	$P31m$	157.53	2	1	$P31m1'$	157.54	4	1
$P31m'$	157.55	2	1	P_c31m	157.56	4	2	$P3c1$	158.57	2	2	$P3c11'$	158.58	4	2
$P3c'1$	158.59	2	2	P_c3c1	158.60	2	2	$P31c$	159.61	2	2	$P31c1'$	159.62	4	2
$P31c'$	159.63	2	2	P_c31c	159.64	4	2	$R3m$	160.65	2	1	$R3m1'$	160.66	2	1
$R3m'$	160.67	1	1	R_I3m	160.68	4	2	$R3c$	161.69	2	2	$R3c1'$	161.70	4	2
$R3c'$	161.71	2	2	R_I3c	161.72	2	2	$P31m$	162.73	4	1	$P31m1'$	162.74	4	1
$P3'1m$	162.75	3	1	$P3'1m'$	162.76	4	1	$P31m'$	162.77	3	1	P_c31m	162.78	6	2
$P31c$	163.79	6	2	$P31c1'$	163.80	6	2	$P3'1c$	163.81	6	2	$P3'1c'$	163.82	6	2
$P31c'$	163.83	6	2	P_c31c	163.84	6	2	$P3m1$	164.85	4	1	$P3m11'$	164.86	4	1
$P3'm1$	164.87	4	1	$P3'm'1$	164.88	3	1	$P3m'1$	164.89	3	1	P_c3m1	164.90	8	2
$P3c1$	165.91	6	2	$P3c11'$	165.92	8	2	$P3'c1$	165.93	6	2	$P3'c'1$	165.94	6	2
$P3c'1$	165.95	6	2	P_c3c1	165.96	6	2	$R3m$	166.97	3	1	$R3m1'$	166.98	3	1
$R3'm$	166.99	3	1	$R3'm'$	166.100	3	1	$R3m'$	166.101	3	1	R_I3m	166.102	6	2
$R3c$	167.103	6	2	$R3c1'$	167.104	6	2	$R3'c$	167.105	6	2	$R3'c'$	167.106	6	2
$R3c'$	167.107	6	2	R_I3c	167.108	6	2	$P6$	168.109	3	1	$P61'$	168.110	4	1
$P6'$	168.111	3	1	P_c6	168.112	6	2	$P6_1$	169.113	6	6	$P6_11'$	169.114	6	6
$P6_1'$	169.115	6	6	P_c6_1	169.116	6	6	$P6_5$	170.117	6	6	$P6_51'$	170.118	6	6
$P6_5'$	170.119	6	6	P_c6_5	170.120	6	6	$P6_2$	171.121	3	3	$P6_21'$	171.122	3	3
$P6_2'$	171.123	3	3	P_c6_2	171.124	6	6	$P6_4$	172.125	3	3	$P6_41'$	172.126	3	3
$P6_4'$	172.127	3	3	P_c6_4	172.128	6	6	$P6_3$	173.129	2	2	$P6_31'$	173.130	4	2
$P6_3'$	173.131	2	2	P_c6_3	173.132	6	2	$P6$	174.133	1	1	$P61'$	174.134	2	1
$P6'$	174.135	2	1	P_c6	174.136	2	2	$P6/m$	175.137	3	1	$P6/m1'$	175.138	4	1
$P6'/m$	175.139	3	1	$P6/m'$	175.140	4	1	$P6'/m'$	175.141	4	1	P_c6/m	175.142	6	2
$P6_3/m$	176.143	6	2	$P6_3/m1'$	176.144	6	2	$P6_3'/m$	176.145	6	2	$P6_3/m'$	176.146	6	2

$P6_3'/m'$	176.147	6	2	P_c6_3/m	176.148	6	2	$P622$	177.149	4	1	$P6221'$	177.150	4	1
$P6'2'2$	177.151	4	1	$P6'22'$	177.152	3	1	$P62'2'$	177.153	3	1	P_c622	177.154	6	2
$P6_122$	178.155	6	6	$P6_1221'$	178.156	6	6	$P6_1'2'2$	178.157	6	6	$P6_1'22'$	178.158	6	6
$P6_12'2'$	178.159	6	6	P_c6_122	178.160	6	6	$P6_522$	179.161	6	6	$P6_5221'$	179.162	6	6
$P6_5'2'2$	179.163	6	6	$P6_5'22'$	179.164	6	6	$P6_52'2'$	179.165	6	6	P_c6_522	179.166	6	6
$P6_222$	180.167	3	3	$P6_2221'$	180.168	3	3	$P6_2'2'2$	180.169	3	3	$P6_2'22'$	180.170	3	3
$P6_22'2'$	180.171	3	3	P_c6_222	180.172	6	6	$P6_422$	181.173	3	3	$P6_4221'$	181.174	3	3
$P6_4'2'2$	181.175	3	3	$P6_4'22'$	181.176	3	3	$P6_42'2'$	181.177	3	3	P_c6_422	181.178	6	6
$P6_322$	182.179	4	2	$P6_3221'$	182.180	4	2	$P6_3'2'2$	182.181	4	2	$P6_3'22'$	182.182	2	2
$P6_32'2'$	182.183	2	2	P_c6_322	182.184	6	2	$P6mm$	183.185	4	1	$P6mm1'$	183.186	4	1
$P6'm'm$	183.187	3	1	$P6'mm'$	183.188	4	1	$P6m'm'$	183.189	3	1	P_c6mm	183.190	8	2
$P6cc$	184.191	6	2	$P6cc1'$	184.192	8	2	$P6'c'c$	184.193	6	2	$P6'cc'$	184.194	6	2
$P6c'c'$	184.195	6	2	P_c6cc	184.196	6	2	$P6_3cm$	185.197	4	2	$P6_3cm1'$	185.198	8	2
$P6_3'c'm$	185.199	4	2	$P6_3'cm'$	185.200	4	2	$P6_3c'm'$	185.201	4	2	P_c6_3cm	185.202	6	2
$P6_3mc$	186.203	4	2	$P6_3mc1'$	186.204	4	2	$P6_3'm'c$	186.205	2	2	$P6_3'mc'$	186.206	4	2
$P6_3m'c'$	186.207	2	2	P_c6_3mc	186.208	8	2	$P6m2$	187.209	2	1	$P6m21'$	187.210	2	1
$P6'm'2$	187.211	2	1	$P6'm2'$	187.212	2	1	$P6m'2'$	187.213	1	1	P_c6m2	187.214	4	2
$P6c2$	188.215	2	2	$P6c21'$	188.216	4	2	$P6'c'2$	188.217	4	2	$P6'c2'$	188.218	2	2
$P6c'2'$	188.219	2	2	P_c6c2	188.220	2	2	$P62m$	189.221	2	1	$P62m1'$	189.222	4	1
$P6'2'm$	189.223	4	1	$P6'2m'$	189.224	4	1	$P62'm'$	189.225	2	1	P_c62m	189.226	4	2
$P62c$	190.227	2	2	$P62c1'$	190.228	4	2	$P6'2'c$	190.229	4	2	$P6'2c'$	190.230	4	2
$P62'c'$	190.231	2	2	P_c62c	190.232	4	2	$P6/mmm$	191.233	4	1	$P6/mmm1'$	191.234	4	1
$P6/m'mm$	191.235	4	1	$P6'/mm'm$	191.236	3	1	$P6'/mmm'$	191.237	4	1	$P6'/m'm'm$	191.238	4	1
$P6'/m'mm'$	191.239	4	1	$P6/mm'm'$	191.240	3	1	$P6/m'm'm'$	191.241	4	1	P_c6/mmm	191.242	8	2
$P6/mcc$	192.243	6	2	$P6/mcc1'$	192.244	8	2	$P6/m'cc$	192.245	6	2	$P6'/mc'c$	192.246	6	2
$P6'/mcc'$	192.247	6	2	$P6'/m'c'c$	192.248	8	2	$P6'/m'cc'$	192.249	6	2	$P6/mc'c'$	192.250	6	2
$P6/m'c'c'$	192.251	8	2	P_c6/mcc	192.252	6	2	$P6_3/mcm$	193.253	6	2	$P6_3/mcm1'$	193.254	8	2
$P6_3/m'cm$	193.255	6	2	$P6_3'/mc'm$	193.256	6	2	$P6_3'/mcm'$	193.257	6	2	$P6_3'/m'c'm$	193.258	8	2
$P6_3'/m'cm'$	193.259	6	2	$P6_3/mc'm'$	193.260	6	2	$P6_3/m'c'm'$	193.261	8	2	P_c6_3/mcm	193.262	6	2
$P6_3/mmc$	194.263	6	2	$P6_3/mmc1'$	194.264	6	2	$P6_3/m'mc$	194.265	6	2	$P6_3'/mm'c$	194.266	6	2
$P6_3'/mmc'$	194.267	6	2	$P6_3'/m'm'c$	194.268	6	2	$P6_3'/m'mc'$	194.269	6	2	$P6_3/mm'c'$	194.270	6	2
$P6_3/m'm'c'$	194.271	6	2	P_c6_3/mmc	194.272	8	2	$P23$	195.1	3	1	$P231'$	195.2	3	1
P_I23	195.3	6	2	$F23$	196.4	3	1	$F231'$	196.5	3	1	F_S23	196.6	6	2
$I23$	197.7	3	1	$I231'$	197.8	3	1	$P2_13$	198.9	4	4	$P2_131'$	198.10	8	4
P_I2_13	198.11	12	8	$I2_13$	199.12	6	4	$I2_131'$	199.13	8	4	$Pm3$	200.14	3	1
$Pm31'$	200.15	3	1	$Pm'3'$	200.16	3	1	P_Im3	200.17	8	2	$Pn3$	201.18	6	2
$Pn31'$	201.19	8	2	$Pn'3'$	201.20	6	2	P_In3	201.21	8	2	$Fm3$	202.22	6	1
$Fm31'$	202.23	6	1	$Fm'3'$	202.24	6	1	F_Sm3	202.25	6	2	$Fd3$	203.26	6	2
$Fd31'$	203.27	8	2	$Fd'3'$	203.28	6	2	F_Sd3	203.29	12	4	$Im3$	204.30	4	1
$Im31'$	204.31	8	1	$Im'3'$	204.32	4	1	$Pa3$	205.33	4	4	$Pa31'$	205.34	8	4
$Pa'3'$	205.35	8	4	P_Ia3	205.36	24	8	$Ia3$	206.37	12	4	$Ia31'$	206.38	12	4
$Ia'3'$	206.39	12	4	$P432$	207.40	3	1	$P4321'$	207.41	6	1	$P4'32'$	207.42	6	1
P_I432	207.43	12	2	$P4_232$	208.44	6	2	$P4_2321'$	208.45	8	2	$P4_2'32'$	208.46	6	2
P_I4_232	208.47	12	2	$F432$	209.48	6	1	$F4321'$	209.49	6	1	$F4'32'$	209.50	6	1
F_S432	209.51	12	2	$F4_132$	210.52	6	2	$F4_1321'$	210.53	8	2	$F4_1'32'$	210.54	6	2
F_S4_132	210.55	12	4	$I432$	211.56	6	1	$I4321'$	211.57	8	1	$I4'32'$	211.58	6	1
$P4_332$	212.59	8	4	$P4_3321'$	212.60	8	4	$P4_3'32'$	212.61	4	4	P_I4_332	212.62	12	8
$P4_132$	213.63	8	4	$P4_1321'$	213.64	8	4	$P4_1'32'$	213.65	4	4	P_I4_132	213.66	12	8
$I4_132$	214.67	8	4	$I4_1321'$	214.68	8	4	$I4_1'32'$	214.69	6	4	$P43m$	215.70	3	1
$P43m1'$	215.71	3	1	$P4'3m'$	215.72	3	1	P_I43m	215.73	12	2	$F43m$	216.74	3	1
$F43m1'$	216.75	3	1	$F4'3m'$	216.76	3	1	F_S43m	216.77	6	2	$I43m$	217.78	6	1
$I43m1'$	217.79	6	1	$I4'3m'$	217.80	6	1	$P43n$	218.81	6	2	$P43n1'$	218.82	6	2
$P4'3n'$	218.83	6	2	P_I43n	218.84	12	2	$F43c$	219.85	6	2	$F43c1'$	219.86	6	2
$F4'3c'$	219.87	6	2	F_S43c	219.88	6	2	$I43d$	220.89	8	6	$I43d1'$	220.90	16	6
$I4'3d'$	220.91	12	6	$Pm3m$	221.92	6	1	$Pm3m1'$	221.93	6	1	$Pm'3'm$	221.94	6	1
$Pm3m'$	221.95	6	1	$Pm'3'm'$	221.96	3	1	P_Im3m	221.97	16	2	$Pn3n$	222.98	12	2
$Pn3n1'$	222.99	16	2	$Pn'3'n$	222.100	12	2	$Pn3n'$	222.101	12	2	$Pn'3'n'$	222.102	12	2
P_In3n	222.103	12	2	$Pm3n$	223.104	8	2	$Pm3n1'$	223.105	16	2	$Pm'3'n$	223.106	8	2
$Pm3n'$	223.107	8	2	$Pm'3'n'$	223.108	8	2	P_Im3n	223.109	12	2	$Pn3m$	224.110	12	2
$Pn3m1'$	224.111	12	2	$Pn'3'm$	224.112	12	2	$Pn3m'$	224.113	12	2	$Pn'3'm'$	224.114	12	2
P_In3m	224.115	16	2	$Fm3m$	225.116	6	1	$Fm3m1'$	225.117	6	1	$Fm'3'm$	225.118	6	1
$Fm3m'$	225.119	6	1	$Fm'3'm'$	225.120	6	1	F_Sm3m	225.121	12	2	$Fm3c$	226.122	6	2
$Fm3c1'$	226.123	12	2	$Fm'3'c$	226.124	12	2	$Fm3c'$	226.125	12	2	$Fm'3'c'$	226.126	6	2
F_Sm3c	226.127	12	2	$Fd3m$	227.128	8	2	$Fd3m1'$	227.129	8	2	$Fd'3'm$	227.130	8	2
$Fd3m'$	227.131	6	2	$Fd'3'm'$	227.132	6	2	F_Sd3m	227.133	24	4	$Fd3c$	228.134	12	4
$Fd3c1'$	228.135	16	4	$Fd'3'c$	228.136	12	4	$Fd3c'$	228.137	12	4	$Fd'3'c'$	228.138	12	4
F_Sd3c	228.139	24	4	$Im3m$	229.140	8	1	$Im3m1'$	229.141	8	1	$Im'3'm$	229.142	8	1
$Im3m'$	229.143	6	1	$Im'3'm'$	229.144	6	1	$Ia3d$	230.145	12	8	$Ia3d1'$	230.146	16	8
$Ia'3'd$	230.147	12	8	$Ia3d'$	230.148	12	8	$Ia'3'd'$	230.149	16	8				

Supplementary Table 23: Maximum and minimum dimensions of the double-valued EBRs of the 1,651 double SSGs. In order, the columns in this table list the symbol of the SSG, the number of the SSG in the BNS setting[67], the maximum EBR dimension in the SSG (M), and the minimum EBR dimension in the SSG (m).

Symbol	BNS Number	M	m	Symbol	BNS Number	M	m	Symbol	BNS Number	M	m	Symbol	BNS Number	M	m
$P1$	1.1	1	1	$P11'$	1.2	2	2	P_S1	1.3	2	2	$P\bar{1}$	2.4	1	1
$P\bar{1}1'$	2.5	2	2	$P\bar{1}'$	2.6	2	2	$P_S\bar{1}$	2.7	2	2	$P2$	3.1	1	1
$P21'$	3.2	2	2	$P2'$	3.3	1	1	P_a2	3.4	2	2	P_b2	3.5	2	2
P_C2	3.6	2	2	$P2_1$	4.7	2	2	$P2_11'$	4.8	4	4	$P2_1'$	4.9	2	2
P_a2_1	4.10	4	4	P_b2_1	4.11	2	2	P_C2_1	4.12	2	2	$C2$	5.13	1	1
$C21'$	5.14	2	2	$C2'$	5.15	1	1	C_c2	5.16	2	2	C_a2	5.17	2	2
Pm	6.18	1	1	$Pm1'$	6.19	2	2	Pm'	6.20	1	1	P_am	6.21	2	2
P_bm	6.22	2	2	P_Cm	6.23	2	2	Pc	7.24	2	2	$Pc1'$	7.25	4	4
Pc'	7.26	2	2	P_ac	7.27	4	4	P_cc	7.28	2	2	P_bc	7.29	4	4
P_Cc	7.30	4	4	P_Ac	7.31	2	2	Cm	8.32	1	1	$Cm1'$	8.33	2	2
Cm'	8.34	1	1	C_cm	8.35	2	2	C_am	8.36	2	2	Cc	9.37	2	2
$Cc1'$	9.38	4	4	Cc'	9.39	2	2	C_cc	9.40	2	2	C_ac	9.41	4	4
$P2/m$	10.42	1	1	$P2/m1'$	10.43	2	2	$P2'/m$	10.44	2	2	$P2/m'$	10.45	2	2
$P2'/m'$	10.46	1	1	P_a2/m	10.47	2	2	P_b2/m	10.48	2	2	P_C2/m	10.49	2	2
$P2_1/m$	11.50	2	2	$P2_1/m1'$	11.51	4	4	$P2_1'/m$	11.52	2	2	$P2_1/m'$	11.53	2	2
$P2_1'/m'$	11.54	2	2	P_a2_1/m	11.55	4	4	P_b2_1/m	11.56	2	2	P_C2_1/m	11.57	4	4
$C2/m$	12.58	2	1	$C2/m1'$	12.59	4	2	$C2'/m$	12.60	2	2	$C2/m'$	12.61	2	2
$C2'/m'$	12.62	2	1	C_c2/m	12.63	4	2	C_a2/m	12.64	2	2	$P2/c$	13.65	2	2
$P2/c1'$	13.66	4	4	$P2'/c$	13.67	2	2	$P2/c'$	13.68	2	2	$P2'/c'$	13.69	2	2
P_a2/c	13.70	4	4	P_b2/c	13.71	4	4	P_c2/c	13.72	2	2	P_A2/c	13.73	4	4
P_C2/c	13.74	4	4	$P2_1/c$	14.75	2	2	$P2_1/c1'$	14.76	4	4	$P2_1'/c$	14.77	4	4
$P2_1/c'$	14.78	4	4	$P2_1'/c'$	14.79	2	2	P_a2_1/c	14.80	4	4	P_b2_1/c	14.81	4	4
P_c2_1/c	14.82	4	4	P_A2_1/c	14.83	2	2	P_C2_1/c	14.84	4	4	$C2/c$	15.85	2	2
$C2/c1'$	15.86	4	4	$C2'/c$	15.87	2	2	$C2/c'$	15.88	2	2	$C2'/c'$	15.89	2	2
C_c2/c	15.90	4	2	C_a2/c	15.91	4	4	$P222$	16.1	2	2	$P2221'$	16.2	2	2
$P2'2'2$	16.3	1	1	P_a222	16.4	2	2	P_C222	16.5	4	4	P_I222	16.6	4	4
$P222_1$	17.7	2	2	$P222_11'$	17.8	4	4	$P2'2'2_1$	17.9	2	2	$P22'2_1'$	17.10	2	2
P_a222_1	17.11	4	4	P_c222_1	17.12	2	2	P_B222_1	17.13	4	2	P_C222_1	17.14	4	4
P_I222_1	17.15	4	4	$P2_12_12$	18.16	2	2	$P2_12_121'$	18.17	4	4	$P2_1'2_1'2$	18.18	2	2
$P2_12_1'2'$	18.19	2	2	$P_b2_12_12$	18.20	4	4	$P_c2_12_12$	18.21	4	4	$P_B2_12_12$	18.22	4	4
$P_C2_12_12$	18.23	4	2	$P_I2_12_12$	18.24	2	2	$P2_12_12_1$	19.25	4	4	$P2_12_12_11'$	19.26	8	8
$P2_1'2_1'2_1$	19.27	4	4	$P_c2_12_12_1$	19.28	4	4	$P_C2_12_12_1$	19.29	4	4	$P_I2_12_12_1$	19.30	4	4
$C222_1$	20.31	2	2	$C222_11'$	20.32	4	4	$C2'2'2_1$	20.33	2	2	$C22'2_1'$	20.34	2	2
C_c222_1	20.35	4	2	C_a222_1	20.36	4	4	C_A222_1	20.37	2	2	$C222$	21.38	2	2
$C2221'$	21.39	4	2	$C2'2'2$	21.40	2	1	$C22'2'$	21.41	2	1	C_c222	21.42	4	2
C_a222	21.43	2	2	C_A222	21.44	4	2	$F222$	22.45	2	2	$F2221'$	22.46	2	2
$F2'2'2$	22.47	1	1	F_S222	22.48	2	2	$I222$	23.49	2	2	$I2221'$	23.50	2	2
$I2'2'2$	23.51	1	1	I_c222	23.52	4	2	$I2_12_12_1$	24.53	2	2	$I2_12_12_11'$	24.54	4	4
$I2_1'2_1'2_1$	24.55	2	2	$I_c2_12_12_1$	24.56	4	2	$Pmm2$	25.57	2	2	$Pmm21'$	25.58	2	2
$Pm'm2'$	25.59	1	1	$Pm'm'2$	25.60	1	1	P_cmm2	25.61	4	4	P_amm2	25.62	2	2
P_Cmm2	25.63	4	4	P_Amm2	25.64	4	4	P_Imm2	25.65	4	4	$Pmc2_1$	26.66	2	2
$Pmc2_11'$	26.67	4	4	$Pm'c2_1'$	26.68	2	2	$Pmc'2_1'$	26.69	2	2	$Pm'c'2_1$	26.70	2	2
P_amc2_1	26.71	4	4	P_bmc2_1	26.72	4	4	P_cmc2_1	26.73	2	2	P_Amc2_1	26.74	2	2
P_Bmc2_1	26.75	4	4	P_Cmc2_1	26.76	4	4	P_Imc2_1	26.77	4	4	$Pcc2$	27.78	2	2
$Pcc21'$	27.79	4	4	$Pc'c2'$	27.80	2	2	$Pc'c'2$	27.81	2	2	P_ccc2	27.82	2	2
P_acc2	27.83	4	4	P_Ccc2	27.84	4	4	P_Acc2	27.85	4	4	P_Icc2	27.86	4	4
$Pma2$	28.87	2	2	$Pma21'$	28.88	4	4	$Pm'a2'$	28.89	2	2	$Pma'2'$	28.90	2	2
$Pm'a'2$	28.91	2	2	P_ama2	28.92	2	2	P_bma2	28.93	4	4	P_cma2	28.94	4	4
P_Ama2	28.95	4	4	P_Bma2	28.96	4	4	P_Cma2	28.97	4	2	P_Ima2	28.98	4	4
$Pca2_1$	29.99	4	4	$Pca2_11'$	29.100	8	8	$Pc'a2_1'$	29.101	4	4	$Pca'2_1'$	29.102	4	4
$Pc'a'2_1$	29.103	4	4	P_aca2_1	29.104	4	4	P_bca2_1	29.105	8	8	P_cca2_1	29.106	4	4
P_Aca2_1	29.107	4	4	P_Bca2_1	29.108	4	4	P_Cca2_1	29.109	4	4	P_Ica2_1	29.110	4	4
$Pnc2$	30.111	2	2	$Pnc21'$	30.112	4	4	$Pn'c2'$	30.113	2	2	$Pnc'2'$	30.114	2	2
$Pn'c'2$	30.115	2	2	P_anc2	30.116	4	4	P_bnc2	30.117	4	4	P_cnc2	30.118	4	4
P_Anc2	30.119	2	2	P_Bnc2	30.120	4	4	P_Cnc2	30.121	4	4	P_Inc2	30.122	4	4
$Pmn2_1$	31.123	2	2	$Pmn2_11'$	31.124	4	4	$Pm'n2_1'$	31.125	2	2	$Pmn'2_1'$	31.126	2	2
$Pm'n'2_1$	31.127	2	2	P_amn2_1	31.128	4	4	P_bmn2_1	31.129	4	4	P_cmn2_1	31.130	4	4
P_Amn2_1	31.131	4	4	P_Bmn2_1	31.132	2	2	P_Cmn2_1	31.133	4	4	P_Imn2_1	31.134	2	2
$Pba2$	32.135	2	2	$Pba21'$	32.136	4	4	$Pb'a2'$	32.137	2	2	$Pb'a'2$	32.138	2	2
P_cba2	32.139	4	4	P_bba2	32.140	4	4	P_Cba2	32.141	4	2	P_Aba2	32.142	4	4
P_Iba2	32.143	4	4	$Pna2_1$	33.144	4	4	$Pna2_11'$	33.145	8	8	$Pn'a2_1'$	33.146	4	4
$Pna'2_1'$	33.147	4	4	$Pn'a'2_1$	33.148	4	4	P_ana2_1	33.149	4	4	P_bna2_1	33.150	8	8
P_cna2_1	33.151	4	4	P_Ana2_1	33.152	4	4	P_Bna2_1	33.153	4	4	P_Cna2_1	33.154	4	4
P_Ina2_1	33.155	4	4	$Pnn2$	34.156	2	2	$Pnn21'$	34.157	4	4	$Pn'n2'$	34.158	2	2
$Pn'n'2$	34.159	2	2	P_ann2	34.160	4	4	P_cnn2	34.161	4	4	P_Ann2	34.162	4	4
P_Cnn2	34.163	4	4	P_Inn2	34.164	2	2	$Cmm2$	35.165	2	2	$Cmm21'$	35.166	4	2
$Cm'm2'$	35.167	2	1	$Cm'm'2$	35.168	2	1	C_cmm2	35.169	4	4	C_amm2	35.170	2	2
C_Amm2	35.171	4	4	$Cmc2_1$	36.172	2	2	$Cmc2_11'$	36.173	4	4	$Cm'c2_1'$	36.174	2	2
$Cmc'2_1'$	36.175	2	2	$Cm'c'2_1$	36.176	2	2	C_cmc2_1	36.177	4	2	C_amc2_1	36.178	4	4

C_Amc2_1	36.179	4	2	$Ccc2$	37.180	2	2	$Ccc21'$	37.181	4	4	$Cc'c2'$	37.182	2	2
$Cc'c'2$	37.183	2	2	C_ccc2	37.184	4	2	C_acc2	37.185	4	4	C_Acc2	37.186	4	2
$Amm2$	38.187	2	2	$Amm21'$	38.188	2	2	$Am'm2'$	38.189	1	1	$Amm'2'$	38.190	1	1
$Am'm'2$	38.191	1	1	A_amm2	38.192	2	2	A_bmm2	38.193	2	2	A_Bmm2	38.194	4	4
$Abm2$	39.195	2	2	$Abm21'$	39.196	4	4	$Ab'm2'$	39.197	2	2	$Abm'2'$	39.198	2	2
$Ab'm'2$	39.199	2	2	A_abm2	39.200	4	4	A_bbm2	39.201	2	2	A_Bbm2	39.202	4	2
$Ama2$	40.203	2	2	$Ama21'$	40.204	4	4	$Am'a2'$	40.205	2	2	$Ama'2'$	40.206	2	2
$Am'a'2$	40.207	2	2	A_ama2	40.208	2	2	A_bma2	40.209	4	4	A_Bma2	40.210	4	2
$Aba2$	41.211	2	2	$Aba21'$	41.212	4	4	$Ab'a2'$	41.213	2	2	$Aba'2'$	41.214	2	2
$Ab'a'2$	41.215	2	2	A_aba2	41.216	4	4	A_bba2	41.217	4	4	A_Bba2	41.218	4	2
$Fmm2$	42.219	2	2	$Fmm21'$	42.220	4	2	$Fm'm2'$	42.221	2	1	$Fm'm'2$	42.222	2	1
F_Smm2	42.223	2	2	$Fdd2$	43.224	2	2	$Fdd21'$	43.225	4	4	$Fd'd2'$	43.226	2	2
$Fd'd'2$	43.227	2	2	F_Sdd2	43.228	4	4	$Imm2$	44.229	2	2	$Imm21'$	44.230	2	2
$Im'm2'$	44.231	1	1	$Im'm'2$	44.232	1	1	I_cmm2	44.233	4	4	I_amm2	44.234	2	2
$Iba2$	45.235	2	2	$Iba21'$	45.236	4	4	$Ib'a2'$	45.237	2	2	$Ib'a'2$	45.238	2	2
I_cba2	45.239	4	2	I_aba2	45.240	4	4	$Ima2$	46.241	2	2	$Ima21'$	46.242	4	4
$Im'a2'$	46.243	2	2	$Ima'2'$	46.244	2	2	$Im'a'2$	46.245	2	2	I_cma2	46.246	4	2
I_ama2	46.247	2	2	I_bma2	46.248	4	4	$Pmmm$	47.249	2	2	$Pmmm1'$	47.250	2	2
$Pm'mm$	47.251	2	2	$Pm'm'm$	47.252	1	1	$Pm'm'm'$	47.253	2	2	P_ammm	47.254	4	4
P_Cmmm	47.255	4	4	P_Immm	47.256	4	4	$Pnnn$	48.257	4	4	$Pnnn1'$	48.258	8	4
$Pn'nn$	48.259	2	2	$Pn'n'n$	48.260	4	2	$Pn'n'n'$	48.261	4	4	P_cnnn	48.262	8	4
P_Cnnn	48.263	4	4	P_Innn	48.264	8	4	$Pccm$	49.265	2	2	$Pccm1'$	49.266	4	4
$Pc'cm$	49.267	2	2	$Pccm'$	49.268	2	2	$Pc'c'm$	49.269	2	2	$Pc'cm'$	49.270	2	2
$Pc'c'm'$	49.271	4	4	P_accm	49.272	4	4	P_cccm	49.273	2	2	P_Bccm	49.274	4	4
P_Cccm	49.275	4	4	P_Iccm	49.276	4	4	$Pban$	50.277	4	4	$Pban1'$	50.278	8	4
$Pb'an$	50.279	2	2	$Pban'$	50.280	2	2	$Pb'a'n$	50.281	4	2	$Pb'an'$	50.282	4	2
$Pb'a'n'$	50.283	4	4	P_aban	50.284	4	4	P_cban	50.285	8	4	P_Aban	50.286	8	8
P_Cban	50.287	4	4	P_Iban	50.288	8	8	$Pmma$	51.289	2	2	$Pmma1'$	51.290	4	4
$Pm'ma$	51.291	2	2	$Pmm'a$	51.292	2	2	$Pmma'$	51.293	4	4	$Pm'm'a$	51.294	2	2
$Pmm'a'$	51.295	2	2	$Pm'ma'$	51.296	2	2	$Pm'm'a'$	51.297	2	2	P_amma	51.298	2	2
P_bmma	51.299	4	4	P_cmma	51.300	4	4	P_Amma	51.301	4	4	P_Bmma	51.302	4	4
P_Cmma	51.303	4	4	P_Imma	51.304	4	4	$Pnna$	52.305	4	4	$Pnna1'$	52.306	8	8
$Pn'na$	52.307	4	4	$Pnn'a$	52.308	4	4	$Pnna'$	52.309	4	4	$Pn'n'a$	52.310	4	4
$Pnn'a'$	52.311	4	4	$Pn'na'$	52.312	4	4	$Pn'n'a'$	52.313	4	4	P_anna	52.314	8	4
P_bnna	52.315	8	4	P_cnna	52.316	8	8	P_Anna	52.317	4	4	P_Bnna	52.318	8	4
P_Cnna	52.319	8	4	P_Inna	52.320	4	4	$Pmna$	53.321	4	2	$Pmna1'$	53.322	8	4
$Pm'na$	53.323	4	4	$Pmn'a$	53.324	4	4	$Pmna'$	53.325	4	4	$Pm'n'a$	53.326	4	2
$Pmn'a'$	53.327	4	2	$Pm'na'$	53.328	4	2	$Pm'n'a'$	53.329	4	4	P_amna	53.330	4	4
P_bmna	53.331	8	4	P_cmna	53.332	4	4	P_Amna	53.333	4	4	P_Bmna	53.334	2	2
P_Cmna	53.335	8	4	P_Imna	53.336	4	4	$Pcca$	54.337	4	4	$Pcca1'$	54.338	8	8
$Pc'ca$	54.339	4	4	$Pcc'a$	54.340	4	4	$Pcca'$	54.341	4	4	$Pc'c'a$	54.342	4	4
$Pcc'a'$	54.343	4	4	$Pc'ca'$	54.344	4	4	$Pc'c'a'$	54.345	4	4	P_acca	54.346	4	4
P_bcca	54.347	8	8	P_ccca	54.348	4	4	P_Acca	54.349	8	8	P_Bcca	54.350	4	4
P_Ccca	54.351	8	4	P_Icca	54.352	8	8	$Pbam$	55.353	2	2	$Pbam1'$	55.354	4	4
$Pb'am$	55.355	4	4	$Pbam'$	55.356	4	4	$Pb'a'm$	55.357	2	2	$Pb'am'$	55.358	2	2
$Pb'a'm'$	55.359	4	4	P_abam	55.360	4	4	P_cbam	55.361	4	4	P_Abam	55.362	8	4
P_Cbam	55.363	2	2	P_Ibam	55.364	4	4	$Pccn$	56.365	4	4	$Pccn1'$	56.366	8	8
$Pc'cn$	56.367	4	4	$Pccn'$	56.368	4	4	$Pc'c'n$	56.369	4	4	$Pc'cn'$	56.370	4	4
$Pc'c'n'$	56.371	4	4	P_bccn	56.372	8	8	P_cccn	56.373	8	4	P_Accn	56.374	8	4
P_Cccn	56.375	4	4	P_Iccn	56.376	8	4	$Pbcm$	57.377	4	4	$Pbcm1'$	57.378	8	8
$Pb'cm$	57.379	4	4	$Pbc'm$	57.380	4	4	$Pbcm'$	57.381	4	4	$Pb'c'm$	57.382	4	4
$Pbc'm'$	57.383	4	4	$Pb'cm'$	57.384	4	4	$Pb'c'm'$	57.385	4	4	P_abcm	57.386	8	8
P_bbcm	57.387	4	4	P_cbcm	57.388	4	4	P_Abcm	57.389	4	4	P_Bbcm	57.390	8	8
P_Cbcm	57.391	8	4	P_Ibcm	57.392	8	4	$Pnnm$	58.393	2	2	$Pnnm1'$	58.394	4	4
$Pn'nm$	58.395	4	4	$Pnnm'$	58.396	4	4	$Pn'n'm$	58.397	2	2	$Pnn'm'$	58.398	2	2
$Pn'n'm'$	58.399	4	4	P_annm	58.400	8	4	P_cnnm	58.401	4	4	P_Bnnm	58.402	4	4
P_Cnnm	58.403	4	4	P_Innm	58.404	2	2	$Pmmn$	59.405	4	4	$Pmmn1'$	59.406	8	4
$Pm'mn$	59.407	2	2	$Pmmn'$	59.408	4	4	$Pm'm'n$	59.409	4	2	$Pmm'n'$	59.410	4	2
$Pm'm'n'$	59.411	2	2	P_bmmn	59.412	4	4	P_cmmn	59.413	8	8	P_Bmmn	59.414	8	8
P_Cmmn	59.415	4	4	P_Immn	59.416	8	4	$Pbcn$	60.417	4	4	$Pbcn1'$	60.418	8	8
$Pb'cn$	60.419	4	4	$Pbc'n$	60.420	4	4	$Pbcn'$	60.421	4	4	$Pb'c'n$	60.422	4	4
$Pbc'n'$	60.423	4	4	$Pb'cn'$	60.424	4	4	$Pb'c'n'$	60.425	4	4	P_abcn	60.426	8	8
P_bbcn	60.427	8	8	P_cbcn	60.428	8	4	P_Abcn	60.429	8	8	P_Bbcn	60.430	8	4
P_Cbcn	60.431	4	4	P_Ibcn	60.432	4	4	$Pbca$	61.433	4	4	$Pbca1'$	61.434	8	8
$Pb'ca$	61.435	8	8	$Pb'c'a$	61.436	4	4	$Pb'c'a'$	61.437	8	8	P_abca	61.438	8	8
P_Cbca	61.439	8	4	P_Ibca	61.440	8	8	$Pnma$	62.441	4	4	$Pnma1'$	62.442	8	8
$Pn'ma$	62.443	4	4	$Pnm'a$	62.444	4	4	$Pnma'$	62.445	4	4	$Pn'm'a$	62.446	4	4
$Pnm'a'$	62.447	4	4	$Pn'ma'$	62.448	4	4	$Pn'm'a'$	62.449	4	4	P_anma	62.450	8	4
P_bnma	62.451	4	4	P_cnma	62.452	8	8	P_Anma	62.453	8	4	P_Bnma	62.454	4	4
P_Cnma	62.455	8	8	P_Inma	62.456	4	4	$Cmcm$	63.457	4	2	$Cmcm1'$	63.458	8	4
$Cm'cm$	63.459	2	2	$Cmc'm$	63.460	4	4	$Cmcm'$	63.461	2	2	$Cm'c'm$	63.462	4	2
$Cmc'm'$	63.463	4	2	$Cm'cm'$	63.464	4	2	$Cm'c'm'$	63.465	2	2	C_cmcm	63.466	4	2
C_amcm	63.467	4	4	C_Amcm	63.468	4	4	$Cmca$	64.469	4	2	$Cmca1'$	64.470	8	4
$Cm'ca$	64.471	4	4	$Cmc'a$	64.472	4	4	$Cmca'$	64.473	4	4	$Cm'c'a$	64.474	4	2
$Cmc'a'$	64.475	4	2	$Cm'ca'$	64.476	4	2	$Cm'c'a'$	64.477	4	4	C_cmca	64.478	4	4
C_amca	64.479	4	4	C_Amca	64.480	4	2	$Cmmm$	65.481	2	2	$Cmmm1'$	65.482	4	2
$Cm'mm$	65.483	2	2	$Cmmm'$	65.484	2	2	$Cm'm'm$	65.485	2	1	$Cmm'm'$	65.486	2	1
$Cm'm'm'$	65.487	2	2	C_cmmm	65.488	4	4	C_ammm	65.489	4	2	C_Ammm	65.490	4	4
$Cccm$	66.491	2	2	$Cccm1'$	66.492	4	4	$Cc'cm$	66.493	2	2	$Cccm'$	66.494	2	2
$Cc'c'm$	66.495	2	2	$Ccc'm'$	66.496	2	2	$Cc'c'm'$	66.497	4	4	C_cccm	66.498	4	2

C_accm	66.499	4	4	C_Accm	66.500	4	2	$Cmma$	67.501	2	2	$Cmma1'$	67.502	4	4
$Cm'ma$	67.503	2	2	$Cmma'$	67.504	2	2	$Cm'm'a$	67.505	2	2	$Cmm'a'$	67.506	2	2
$Cm'm'a'$	67.507	2	2	C_cmma	67.508	4	4	C_amma	67.509	2	2	C_Amma	67.510	4	4
$Ccca$	68.511	4	4	$Ccca1'$	68.512	8	4	$Cc'ca$	68.513	4	2	$Ccca'$	68.514	4	2
$Cc'c'a$	68.515	4	2	$Ccc'a'$	68.516	4	2	$Cc'c'a'$	68.517	4	4	C_ccca	68.518	4	4
C_acca	68.519	4	4	C_Acca	68.520	4	4	$Fmmm$	69.521	2	2	$Fmmm1'$	69.522	4	2
$Fm'mm$	69.523	2	2	$Fm'm'm$	69.524	2	1	$Fm'm'm'$	69.525	2	2	F_Smmm	69.526	4	2
$Fddd$	70.527	4	4	$Fddd1'$	70.528	8	4	$Fd'dd$	70.529	2	2	$Fd'd'd$	70.530	4	2
$Fd'd'd'$	70.531	4	4	F_Sddd	70.532	8	4	$Immm$	71.533	4	2	$Immm1'$	71.534	8	2
$Im'mm$	71.535	2	2	$Im'm'm$	71.536	4	1	$Im'm'm'$	71.537	2	2	I_cmmm	71.538	4	4
$Ibam$	72.539	4	2	$Ibam1'$	72.540	8	4	$Ib'am$	72.541	2	2	$Ibam'$	72.542	2	2
$Ib'a'm$	72.543	4	2	$Iba'm'$	72.544	4	2	$Ib'a'm'$	72.545	4	4	I_cbam	72.546	4	2
I_bbam	72.547	4	4	$Ibca$	73.548	4	4	$Ibca1'$	73.549	8	8	$Ib'ca$	73.550	4	4
$Ib'c'a$	73.551	4	4	$Ib'c'a'$	73.552	4	4	I_cbca	73.553	4	4	$Imma$	74.554	2	2
$Imma1'$	74.555	4	4	$Im'ma$	74.556	2	2	$Imma'$	74.557	4	4	$Im'm'a$	74.558	2	2
$Imm'a'$	74.559	2	2	$Im'm'a'$	74.560	2	2	I_cmma	74.561	4	4	I_bmma	74.562	4	2
$P4$	75.1	2	1	$P41'$	75.2	4	2	$P4'$	75.3	2	2	P_c4	75.4	4	2
P_C4	75.5	4	2	P_I4	75.6	4	2	$P4_1$	76.7	4	4	$P4_11'$	76.8	8	8
$P4'_1$	76.9	4	4	P_c4_1	76.10	4	4	P_C4_1	76.11	8	8	P_I4_1	76.12	4	4
$P4_2$	77.13	2	2	$P4_21'$	77.14	4	4	$P4'_2$	77.15	2	2	P_c4_2	77.16	4	4
P_C4_2	77.17	4	4	P_I4_2	77.18	4	4	$P4_3$	78.19	4	4	$P4_31'$	78.20	8	8
$P4'_3$	78.21	4	4	P_c4_3	78.22	4	4	P_C4_3	78.23	8	8	P_I4_3	78.24	4	4
$I4$	79.25	2	1	$I41'$	79.26	4	2	$I4'$	79.27	2	2	I_c4	79.28	4	2
$I4_1$	80.29	2	2	$I4_11'$	80.30	4	4	$I4'_1$	80.31	2	2	I_c4_1	80.32	4	4
$P\bar{4}$	81.33	2	1	$P\bar{4}1'$	81.34	4	2	$P\bar{4}'$	81.35	2	2	$P_c\bar{4}$	81.36	4	2
$P_C\bar{4}$	81.37	4	2	$P_I\bar{4}$	81.38	4	2	$I\bar{4}$	82.39	1	1	$I\bar{4}1'$	82.40	2	2
$I\bar{4}'$	82.41	2	2	$I_c\bar{4}$	82.42	4	2	$P4/m$	83.43	2	1	$P4/m1'$	83.44	4	2
$P4'/m$	83.45	2	2	$P4/m'$	83.46	2	2	$P4'/m'$	83.47	2	2	P_c4/m	83.48	4	2
P_C4/m	83.49	4	2	P_I4/m	83.50	4	2	$P4_2/m$	84.51	2	2	$P4_2/m1'$	84.52	4	4
$P4'_2/m$	84.53	2	2	$P4_2/m'$	84.54	4	4	$P4'_2/m'$	84.55	2	2	P_c4_2/m	84.56	4	4
P_C4_2/m	84.57	4	4	P_I4_2/m	84.58	4	4	$P4/n$	85.59	4	2	$P4/n1'$	85.60	8	4
$P4'/n$	85.61	4	4	$P4/n'$	85.62	4	2	$P4'/n'$	85.63	4	2	P_c4/n	85.64	8	4
P_C4/n	85.65	4	4	P_I4/n	85.66	8	4	$P4_2/n$	86.67	4	2	$P4_2/n1'$	86.68	8	4
$P4'_2/n$	86.69	4	4	$P4_2/n'$	86.70	4	4	$P4'_2/n'$	86.71	4	2	P_c4_2/n	86.72	8	4
P_C4_2/n	86.73	4	4	P_I4_2/n	86.74	8	4	$I4/m$	87.75	4	1	$I4/m1'$	87.76	8	2
$I4'/m$	87.77	4	2	$I4/m'$	87.78	2	2	$I4'/m'$	87.79	2	2	I_c4/m	87.80	4	2
$I4_1/a$	88.81	4	2	$I4_1/a1'$	88.82	8	4	$I4'_1/a$	88.83	4	4	$I4_1/a'$	88.84	4	4
$I4'_1/a'$	88.85	2	2	I_c4_1/a	88.86	8	4	$P422$	89.87	2	2	$P4221'$	89.88	4	2
$P4'22'$	89.89	2	2	$P42'2'$	89.90	2	1	$P4'2'2$	89.91	2	2	P_c422	89.92	4	2
P_C422	89.93	4	4	P_I422	89.94	4	4	$P42_12$	90.95	4	2	$P42_121'$	90.96	4	4
$P4'2_12'$	90.97	4	2	$P42'_12'$	90.98	2	2	$P4'2'_12$	90.99	4	4	P_c42_12	90.100	4	4
P_C42_12	90.101	4	2	P_I42_12	90.102	4	2	$P4_122$	91.103	4	4	$P4_1221'$	91.104	8	8
$P4'_122'$	91.105	4	4	$P4_12'2'$	91.106	4	4	$P4'_12'2$	91.107	4	4	P_c4_122	91.108	4	4
P_C4_122	91.109	8	8	P_I4_122	91.110	8	4	$P4_12_12$	92.111	4	4	$P4_12_121'$	92.112	8	8
$P4'_12_12'$	92.113	4	4	$P4_12'_12'$	92.114	4	4	$P4'_12'_12$	92.115	4	4	$P_c4_12_12$	92.116	8	4
$P_C4_12_12$	92.117	8	8	$P_I4_12_12$	92.118	8	4	$P4_222$	93.119	4	4	$P4_2221'$	93.120	4	4
$P4'_222'$	93.121	2	2	$P4_22'2'$	93.122	2	2	$P4'_22'2$	93.123	2	2	P_c4_222	93.124	4	4
P_C4_222	93.125	4	4	P_I4_222	93.126	4	4	$P4_22_12$	94.127	4	4	$P4_22_121'$	94.128	8	4
$P4'_22_12'$	94.129	4	2	$P4_22'_12'$	94.130	4	2	$P4'_22'_12$	94.131	4	4	$P_c4_22_12$	94.132	8	4
$P_C4_22_12$	94.133	4	4	$P_I4_22_12$	94.134	4	4	$P4_322$	95.135	4	4	$P4_3221'$	95.136	8	8
$P4'_322'$	95.137	4	4	$P4_32'2'$	95.138	4	4	$P4'_32'2$	95.139	4	4	P_c4_322	95.140	4	4
P_C4_322	95.141	8	8	P_I4_322	95.142	8	4	$P4_32_12$	96.143	4	4	$P4_32_121'$	96.144	8	8
$P4'_32_12'$	96.145	4	4	$P4_32'_12'$	96.146	4	4	$P4'_32'_12$	96.147	4	4	$P_c4_32_12$	96.148	8	4
$P_C4_32_12$	96.149	8	8	$P_I4_32_12$	96.150	8	4	$I422$	97.151	2	2	$I4221'$	97.152	4	2
$I4'22'$	97.153	2	2	$I42'2'$	97.154	2	1	$I4'2'2$	97.155	2	2	I_c422	97.156	4	2
$I4_122$	98.157	4	4	$I4_1221'$	98.158	8	4	$I4'_122'$	98.159	4	2	$I4_12'2'$	98.160	4	2
$I4'_12'2$	98.161	4	4	I_c4_122	98.162	4	4	$P4mm$	99.163	2	2	$P4mm1'$	99.164	4	2
$P4'm'm$	99.165	2	2	$P4'mm'$	99.166	2	2	$P4m'm'$	99.167	2	1	P_c4mm	99.168	4	4
P_C4mm	99.169	4	4	P_I4mm	99.170	4	4	$P4bm$	100.171	4	2	$P4bm1'$	100.172	4	4
$P4'b'm$	100.173	4	4	$P4'bm'$	100.174	4	2	$P4b'm'$	100.175	2	2	P_c4bm	100.176	8	4
P_C4bm	100.177	4	2	P_I4bm	100.178	8	4	$P4_2cm$	101.179	4	4	$P4_2cm1'$	101.180	8	4
$P4'_2c'm$	101.181	4	4	$P4'_2cm'$	101.182	4	2	$P4_2c'm'$	101.183	4	2	P_c4_2cm	101.184	4	4
P_C4_2cm	101.185	8	4	P_I4_2cm	101.186	8	8	$P4_2nm$	102.187	4	4	$P4_2nm1'$	102.188	8	4
$P4'_2n'm$	102.189	4	4	$P4'_2nm'$	102.190	4	2	$P4_2n'm'$	102.191	4	2	P_c4_2nm	102.192	8	8
P_C4_2nm	102.193	8	4	P_I4_2nm	102.194	4	4	$P4cc$	103.195	4	2	$P4cc1'$	103.196	8	4
$P4'c'c$	103.197	4	4	$P4'cc'$	103.198	4	4	$P4c'c'$	103.199	4	2	P_c4cc	103.200	4	2
P_C4cc	103.201	8	4	P_I4cc	103.202	4	4	$P4nc$	104.203	4	2	$P4nc1'$	104.204	8	4
$P4'n'c$	104.205	4	4	$P4'nc'$	104.206	4	4	$P4n'c'$	104.207	4	2	P_c4nc	104.208	4	4
P_C4nc	104.209	8	4	P_I4nc	104.210	4	2	$P4_2mc$	105.211	4	4	$P4_2mc1'$	105.212	4	4
$P4'_2m'c$	105.213	2	2	$P4'_2mc'$	105.214	4	4	$P4_2m'c'$	105.215	2	2	P_c4_2mc	105.216	4	4
P_C4_2mc	105.217	8	8	P_I4_2mc	105.218	4	4	$P4_2bc$	106.219	4	4	$P4_2bc1'$	106.220	8	8
$P4'_2b'c$	106.221	4	4	$P4'_2bc'$	106.222	4	4	$P4_2b'c'$	106.223	4	4	P_c4_2bc	106.224	8	4
P_C4_2bc	106.225	8	4	P_I4_2bc	106.226	8	4	$I4mm$	107.227	2	2	$I4mm1'$	107.228	4	2
$I4'm'm$	107.229	2	2	$I4'mm'$	107.230	2	2	$I4m'm'$	107.231	2	1	I_c4mm	107.232	4	4
$I4cm$	108.233	4	2	$I4cm1'$	108.234	4	4	$I4'c'm$	108.235	4	4	$I4'cm'$	108.236	4	2

$I4c'm'$	108.237	2	2	I_c4cm	108.238	4	2	$I4_1md$	109.239	4	4	$I4_1md1'$	109.240	4	4
$I4_1'm'd$	109.241	2	2	$I4_1'md'$	109.242	4	4	$I4_1m'd'$	109.243	2	2	I_c4_1md	109.244	8	8
$I4_1cd$	110.245	4	4	$I4_1cd1'$	110.246	8	8	$I4_1'c'd$	110.247	4	4	$I4_1'cd'$	110.248	4	4
$I4_1c'd'$	110.249	4	4	I_c4_1cd	110.250	8	4	$P42m$	111.251	2	2	$P42m1'$	111.252	4	2
$P4'2'm$	111.253	2	2	$P4'2m'$	111.254	2	2	$P42'm'$	111.255	2	1	P_c42m	111.256	4	4
P_C42m	111.257	4	4	P_I42m	111.258	4	4	$P42c$	112.259	2	2	$P42c1'$	112.260	4	4
$P4'2'c$	112.261	2	2	$P4'2c'$	112.262	4	4	$P42'c'$	112.263	2	2	P_c42c	112.264	4	2
P_C42c	112.265	8	4	P_I42c	112.266	4	4	$P42_1m$	113.267	4	2	$P42_1m1'$	113.268	4	4
$P4'2_1'm$	113.269	4	4	$P4'2_1m'$	113.270	4	2	$P42_1'm'$	113.271	2	2	P_c42_1m	113.272	8	4
P_C42_1m	113.273	4	2	P_I42_1m	113.274	4	4	$P42_1c$	114.275	4	2	$P42_1c1'$	114.276	8	4
$P4'2_1'c$	114.277	4	4	$P4'2_1c'$	114.278	4	4	$P42_1'c'$	114.279	4	2	P_c42_1c	114.280	4	4
P_C42_1c	114.281	8	4	P_I42_1c	114.282	4	2	$P4m2$	115.283	2	2	$P4m21'$	115.284	4	2
$P4'm'2$	115.285	2	2	$P4'm2'$	115.286	2	2	$P4m'2'$	115.287	2	1	P_c4m2	115.288	4	4
P_C4m2	115.289	4	4	P_I4m2	115.290	4	4	$P4c2$	116.291	4	2	$P4c21'$	116.292	8	4
$P4'c'2$	116.293	4	4	$P4'c2'$	116.294	4	2	$P4c'2'$	116.295	4	2	P_c4c2	116.296	4	2
P_C4c2	116.297	4	4	P_I4c2	116.298	4	4	$P4b2$	117.299	4	2	$P4b21'$	117.300	4	4
$P4'b'2$	117.301	4	4	$P4'b2'$	117.302	4	2	$P4b'2'$	117.303	2	2	P_c4b2	117.304	4	4
P_C4b2	117.305	4	2	P_I4b2	117.306	8	4	$P4n2$	118.307	4	2	$P4n21'$	118.308	4	4
$P4'n'2$	118.309	4	4	$P4'n2'$	118.310	4	2	$P4n'2'$	118.311	2	2	P_c4n2	118.312	4	4
P_C4n2	118.313	4	4	P_I4n2	118.314	4	2	$I4m2$	119.315	2	2	$I4m21'$	119.316	2	2
$I4'm'2$	119.317	2	2	$I4'm2'$	119.318	2	2	$I4m'2'$	119.319	1	1	I_c4m2	119.320	4	4
$I4c2$	120.321	2	2	$I4c21'$	120.322	4	4	$I4'c'2$	120.323	4	4	$I4'c2'$	120.324	2	2
$I4c'2'$	120.325	2	2	I_c4c2	120.326	4	2	$I42m$	121.327	2	2	$I42m1'$	121.328	4	2
$I4'2'm$	121.329	2	2	$I4'2m'$	121.330	2	2	$I42'm'$	121.331	2	1	I_c42m	121.332	4	2
$I42d$	122.333	4	2	$I42d1'$	122.334	8	4	$I4'2'd$	122.335	4	4	$I4'2d'$	122.336	4	4
$I42'd'$	122.337	4	2	I_c42d	122.338	4	4	$P4/mmm$	123.339	4	2	$P4/mmm1'$	123.340	4	2
$P4/m'mm$	123.341	2	2	$P4'/mm'm$	123.342	2	2	$P4'/mmm'$	123.343	4	2	$P4'/m'm'm$	123.344	2	2
$P4/mm'm'$	123.345	2	1	$P4'/m'mm'$	123.346	2	2	$P4/m'm'm'$	123.347	2	2	P_c4/mmm	123.348	8	4
P_C4/mmm	123.349	4	4	P_I4/mmm	123.350	8	4	$P4/mcc$	124.351	4	2	$P4/mcc1'$	124.352	8	4
$P4/m'cc$	124.353	4	2	$P4'/mc'c$	124.354	4	4	$P4'/mcc'$	124.355	4	4	$P4'/m'c'c$	124.356	4	4
$P4/mc'c'$	124.357	4	2	$P4'/m'cc'$	124.358	4	4	$P4/m'c'c'$	124.359	4	4	P_c4/mcc	124.360	4	2
P_C4/mcc	124.361	8	4	P_I4/mcc	124.362	4	4	$P4/nbm$	125.363	4	4	$P4/nbm1'$	125.364	8	4
$P4/n'bm$	125.365	4	2	$P4'/nb'm$	125.366	4	4	$P4'/nbm'$	125.367	4	4	$P4'/n'b'm$	125.368	4	4
$P4/nb'm'$	125.369	4	2	$P4'/n'bm'$	125.370	4	2	$P4/n'b'm'$	125.371	4	4	P_c4/nbm	125.372	8	4
P_C4/nbm	125.373	4	4	P_I4/nbm	125.374	8	8	$P4/nnc$	126.375	8	4	$P4/nnc1'$	126.376	16	4
$P4/n'nc$	126.377	4	2	$P4'/nn'c$	126.378	8	4	$P4'/nnc'$	126.379	8	4	$P4'/n'n'c$	126.380	4	4
$P4/nn'c'$	126.381	8	2	$P4'/n'nc'$	126.382	4	4	$P4/n'n'c'$	126.383	4	4	P_c4/nnc	126.384	8	4
P_C4/nnc	126.385	8	8	P_I4/nnc	126.386	8	4	$P4/mbm$	127.387	4	2	$P4/mbm1'$	127.388	4	4
$P4/m'bm$	127.389	4	4	$P4'/mb'm$	127.390	4	4	$P4'/mbm'$	127.391	4	2	$P4'/m'b'm$	127.392	4	4
$P4/mb'm'$	127.393	2	2	$P4'/m'bm'$	127.394	4	4	$P4/m'b'm'$	127.395	4	4	P_c4/mbm	127.396	8	4
P_C4/mbm	127.397	4	2	P_I4/mbm	127.398	8	4	$P4/mnc$	128.399	4	2	$P4/mnc1'$	128.400	8	4
$P4/m'nc$	128.401	4	4	$P4'/mn'c$	128.402	4	4	$P4'/mnc'$	128.403	4	4	$P4'/m'n'c$	128.404	4	4
$P4/mn'c'$	128.405	4	2	$P4'/m'nc'$	128.406	4	4	$P4/m'n'c'$	128.407	4	4	P_c4/mnc	128.408	4	4
P_C4/mnc	128.409	8	4	P_I4/mnc	128.410	4	2	$P4/nmm$	129.411	4	4	$P4/nmm1'$	129.412	8	4
$P4/n'mm$	129.413	4	4	$P4'/nm'm$	129.414	4	4	$P4'/nmm'$	129.415	4	4	$P4'/n'm'm$	129.416	4	2
$P4/nm'm'$	129.417	4	2	$P4'/n'mm'$	129.418	4	4	$P4/n'm'm'$	129.419	4	2	P_c4/nmm	129.420	8	8
P_C4/nmm	129.421	4	4	P_I4/nmm	129.422	8	4	$P4/ncc$	130.423	8	4	$P4/ncc1'$	130.424	16	8
$P4/n'cc$	130.425	4	4	$P4'/nc'c$	130.426	8	8	$P4'/ncc'$	130.427	8	4	$P4'/n'c'c$	130.428	8	4
$P4/nc'c'$	130.429	8	4	$P4'/n'cc'$	130.430	8	4	$P4/n'c'c'$	130.431	8	4	P_c4/ncc	130.432	8	4
P_C4/ncc	130.433	8	4	P_I4/ncc	130.434	8	4	$P4_2/mmc$	131.435	4	4	$P4_2/mmc1'$	131.436	4	4
$P4_2/m'mc$	131.437	4	4	$P4_2'/mm'c$	131.438	2	2	$P4_2'/mmc'$	131.439	4	4	$P4_2'/m'm'c$	131.440	2	2
$P4_2/mm'c'$	131.441	2	2	$P4_2'/m'mc'$	131.442	4	4	$P4_2/m'm'c'$	131.443	4	4	P_c4_2/mmc	131.444	8	4
P_C4_2/mmc	131.445	8	8	P_I4_2/mmc	131.446	8	4	$P4_2/mcm$	132.447	4	4	$P4_2/mcm1'$	132.448	8	4
$P4_2/m'cm$	132.449	4	4	$P4_2'/mc'm$	132.450	4	4	$P4_2'/mcm'$	132.451	4	2	$P4_2'/m'c'm$	132.452	4	4
$P4_2/mc'm'$	132.453	4	2	$P4_2'/m'cm'$	132.454	4	2	$P4_2/m'c'm'$	132.455	4	4	P_c4_2/mcm	132.456	4	4
P_C4_2/mcm	132.457	8	4	P_I4_2/mcm	132.458	8	8	$P4_2/nbc$	133.459	8	4	$P4_2/nbc1'$	133.460	16	8
$P4_2/n'bc$	133.461	4	4	$P4_2'/nb'c$	133.462	8	4	$P4_2'/nbc'$	133.463	8	4	$P4_2'/n'b'c$	133.464	4	4
$P4_2/nb'c'$	133.465	8	4	$P4_2'/n'bc'$	133.466	4	4	$P4_2/n'b'c'$	133.467	8	8	P_c4_2/nbc	133.468	8	4
P_C4_2/nbc	133.469	8	4	P_I4_2/nbc	133.470	8	8	$P4_2/nnm$	134.471	4	4	$P4_2/nnm1'$	134.472	8	4
$P4_2/n'nm$	134.473	4	4	$P4_2'/nn'm$	134.474	4	4	$P4_2'/nnm'$	134.475	4	4	$P4_2'/n'n'm$	134.476	4	4
$P4_2/nn'm'$	134.477	4	2	$P4_2'/n'nm'$	134.478	4	2	$P4_2/n'n'm'$	134.479	4	4	P_c4_2/nnm	134.480	8	8
P_C4_2/nnm	134.481	8	4	P_I4_2/nnm	134.482	8	4	$P4_2/mbc$	135.483	4	4	$P4_2/mbc1'$	135.484	8	8
$P4_2/m'bc$	135.485	4	4	$P4_2'/mb'c$	135.486	4	4	$P4_2'/mbc'$	135.487	4	4	$P4_2'/m'b'c$	135.488	4	4
$P4_2/mb'c'$	135.489	4	4	$P4_2'/m'bc'$	135.490	4	4	$P4_2/m'b'c'$	135.491	8	8	P_c4_2/mbc	135.492	8	4
P_C4_2/mbc	135.493	8	4	P_I4_2/mbc	135.494	8	4	$P4_2/mnm$	136.495	4	4	$P4_2/mnm1'$	136.496	8	4
$P4_2/m'nm$	136.497	4	4	$P4_2'/mn'm$	136.498	4	4	$P4_2'/mnm'$	136.499	4	2	$P4_2'/m'n'm$	136.500	4	4
$P4_2/mn'm'$	136.501	4	2	$P4_2'/m'nm'$	136.502	4	4	$P4_2/m'n'm'$	136.503	4	4	P_c4_2/mnm	136.504	8	8
P_C4_2/mnm	136.505	8	4	P_I4_2/mnm	136.506	4	4	$P4_2/nmc$	137.507	8	4	$P4_2/nmc1'$	137.508	16	4
$P4_2/n'mc$	137.509	4	4	$P4_2'/nm'c$	137.510	8	4	$P4_2'/nmc'$	137.511	8	4	$P4_2'/n'm'c$	137.512	4	2
$P4_2/nm'c'$	137.513	8	2	$P4_2'/n'mc'$	137.514	4	4	$P4_2/n'm'c'$	137.515	4	4	P_c4_2/nmc	137.516	8	8

P_C4_2/nmc	137.517	8	8	P_I4_2/nmc	137.518	8	4	$P4_2/ncm$	138.519	4	4	$P4_2/ncm1'$	138.520	8	8
$P4_2/n'cm$	138.521	8	4	$P4_2'/nc'm$	138.522	8	4	$P4_2'/ncm'$	138.523	4	4	$P4_2'/n'c'm$	138.524	8	4
$P4_2/nc'm'$	138.525	4	4	$P4_2'/n'cm'$	138.526	4	4	$P4_2/n'c'm'$	138.527	8	4	P_c4_2/ncm	138.528	8	4
P_C4_2/ncm	138.529	8	4	P_I4_2/ncm	138.530	8	8	$I4/mmm$	139.531	4	2	$I4/mmm1'$	139.532	8	2
$I4/m'mm$	139.533	4	2	$I4'/mm'm$	139.534	4	2	$I4'/mmm'$	139.535	4	2	$I4'/m'm'm$	139.536	2	2
$I4/mm'm'$	139.537	4	1	$I4'/m'mm'$	139.538	4	2	$I4/m'm'm'$	139.539	2	2	I_c4/mmm	139.540	4	4
$I4/mcm$	140.541	4	2	$I4/mcm1'$	140.542	8	4	$I4/m'cm$	140.543	4	2	$I4'/mc'm$	140.544	4	4
$I4'/mcm'$	140.545	4	2	$I4'/m'c'm$	140.546	4	4	$I4/mc'm'$	140.547	4	2	$I4'/m'cm'$	140.548	4	2
$I4/m'c'm'$	140.549	4	4	I_c4/mcm	140.550	4	2	$I4_1/amd$	141.551	4	4	$I4_1/amd1'$	141.552	8	4
$I4_1/a'md$	141.553	4	4	$I4_1'/am'd$	141.554	4	4	$I4_1'/amd'$	141.555	4	4	$I4_1'/a'm'd$	141.556	2	2
$I4_1/am'd'$	141.557	4	2	$I4_1'/a'md'$	141.558	4	4	$I4_1/a'm'd'$	141.559	4	4	I_c4_1/amd	141.560	8	8
$I4_1/acd$	142.561	8	4	$I4_1/acd1'$	142.562	16	8	$I4_1/a'cd$	142.563	8	4	$I4_1'/ac'd$	142.564	8	8
$I4_1'/acd'$	142.565	8	4	$I4_1'/a'c'd$	142.566	8	4	$I4_1/ac'd'$	142.567	8	4	$I4_1'/a'cd'$	142.568	8	4
$I4_1/a'c'd'$	142.569	8	8	I_c4_1/acd	142.570	8	4	$P3$	143.1	1	1	$P31'$	143.2	2	2
P_c3	143.3	2	2	$P3_1$	144.4	3	3	$P3_11'$	144.5	6	6	P_c3_1	144.6	6	6
$P3_2$	145.7	3	3	$P3_21'$	145.8	6	6	P_c3_2	145.9	6	6	$R3$	146.10	1	1
$R31'$	146.11	2	2	R_I3	146.12	2	2	$P3$	147.13	3	1	$P31'$	147.14	6	2
$P3'$	147.15	2	2	P_c3	147.16	6	2	$R3$	148.17	3	1	$R31'$	148.18	6	2
$R3'$	148.19	2	2	R_I3	148.20	6	2	$P312$	149.21	2	1	$P3121'$	149.22	2	2
$P312'$	149.23	1	1	P_c312	149.24	2	2	$P321$	150.25	2	1	$P3211'$	150.26	4	2
$P32'1$	150.27	2	1	P_c321	150.28	4	2	$P3_112$	151.29	3	3	$P3_1121'$	151.30	6	6
$P3_112'$	151.31	3	3	P_c3_112	151.32	6	6	$P3_121$	152.33	3	3	$P3_1211'$	152.34	6	6
$P3_12'1$	152.35	3	3	P_c3_121	152.36	6	6	$P3_212$	153.37	3	3	$P3_2121'$	153.38	6	6
$P3_212'$	153.39	3	3	P_c3_212	153.40	6	6	$P3_221$	154.41	3	3	$P3_2211'$	154.42	6	6
$P3_22'1$	154.43	3	3	P_c3_221	154.44	6	6	$R32$	155.45	2	1	$R321'$	155.46	2	2
$R32'$	155.47	1	1	R_I32	155.48	2	2	$P3m1$	156.49	2	1	$P3m11'$	156.50	2	2
$P3m'1$	156.51	1	1	P_c3m1	156.52	4	2	$P31m$	157.53	2	1	$P31m1'$	157.54	4	2
$P31m'$	157.55	2	1	P_c31m	157.56	4	2	$P3c1$	158.57	2	2	$P3c11'$	158.58	4	4
$P3c'1$	158.59	2	2	P_c3c1	158.60	2	2	$P31c$	159.61	2	2	$P31c1'$	159.62	4	4
$P31c'$	159.63	2	2	P_c31c	159.64	4	2	$R3m$	160.65	2	1	$R3m1'$	160.66	2	2
$R3m'$	160.67	1	1	R_I3m	160.68	4	2	$R3c$	161.69	2	2	$R3c1'$	161.70	4	4
$R3c'$	161.71	2	2	R_I3c	161.72	2	2	$P31m$	162.73	4	1	$P31m1'$	162.74	6	2
$P3'1m$	162.75	2	2	$P3'1m'$	162.76	4	2	$P31m'$	162.77	3	1	P_c31m	162.78	6	2
$P31c$	163.79	6	2	$P31c1'$	163.80	12	4	$P3'1c$	163.81	2	2	$P3'1c'$	163.82	4	2
$P31c'$	163.83	6	2	P_c31c	163.84	6	2	$P3m1$	164.85	4	1	$P3m11'$	164.86	6	2
$P3'm1$	164.87	4	2	$P3'm'1$	164.88	2	2	$P3m'1$	164.89	3	1	P_c3m1	164.90	8	2
$P3c1$	165.91	6	2	$P3c11'$	165.92	12	4	$P3'c1$	165.93	4	2	$P3'c'1$	165.94	4	2
$P3c'1$	165.95	6	2	P_c3c1	165.96	6	2	$R3m$	166.97	3	1	$R3m1'$	166.98	6	2
$R3'm$	166.99	2	2	$R3'm'$	166.100	2	2	$R3m'$	166.101	3	1	R_I3m	166.102	6	2
$R3c$	167.103	6	2	$R3c1'$	167.104	12	4	$R3'c$	167.105	2	2	$R3'c'$	167.106	4	2
$R3c'$	167.107	6	2	R_I3c	167.108	6	2	$P6$	168.109	3	1	$P61'$	168.110	6	2
$P6'$	168.111	3	1	P_c6	168.112	6	2	$P6_1$	169.113	6	6	$P6_11'$	169.114	12	12
$P6_1'$	169.115	6	6	P_c6_1	169.116	6	6	$P6_5$	170.117	6	6	$P6_51'$	170.118	12	12
$P6_5'$	170.119	6	6	P_c6_5	170.120	6	6	$P6_2$	171.121	3	3	$P6_21'$	171.122	6	6
$P6_2'$	171.123	3	3	P_c6_2	171.124	6	6	$P6_4$	172.125	3	3	$P6_41'$	172.126	6	6
$P6_4'$	172.127	3	3	P_c6_4	172.128	6	6	$P6_3$	173.129	2	2	$P6_31'$	173.130	4	4
$P6_3'$	173.131	2	2	P_c6_3	173.132	6	2	$P6$	174.133	1	1	$P61'$	174.134	2	2
$P6'$	174.135	2	1	P_c6	174.136	2	2	$P6/m$	175.137	3	1	$P6/m1'$	175.138	6	2
$P6'/m$	175.139	2	2	$P6/m'$	175.140	4	2	$P6'/m'$	175.141	4	1	P_c6/m	175.142	6	2
$P6_3/m$	176.143	6	2	$P6_3/m1'$	176.144	12	4	$P6_3'/m$	176.145	2	2	$P6_3/m'$	176.146	4	2
$P6_3'/m'$	176.147	6	2	P_c6_3/m	176.148	6	2	$P622$	177.149	4	2	$P6221'$	177.150	6	2
$P6'2'2$	177.151	4	1	$P6'22'$	177.152	3	1	$P62'2'$	177.153	3	1	P_c622	177.154	6	2
$P6_122$	178.155	6	6	$P6_1221'$	178.156	12	12	$P6_1'2'2$	178.157	6	6	$P6_1'22'$	178.158	6	6
$P6_12'2'$	178.159	6	6	P_c6_122	178.160	6	6	$P6_522$	179.161	6	6	$P6_5221'$	179.162	12	12
$P6_5'2'2$	179.163	6	6	$P6_5'22'$	179.164	6	6	$P6_52'2'$	179.165	6	6	P_c6_522	179.166	6	6
$P6_222$	180.167	6	6	$P6_2221'$	180.168	6	6	$P6_2'2'2$	180.169	3	3	$P6_2'22'$	180.170	3	3
$P6_22'2'$	180.171	3	3	P_c6_222	180.172	6	6	$P6_422$	181.173	6	6	$P6_4221'$	181.174	6	6
$P6_4'2'2$	181.175	3	3	$P6_4'22'$	181.176	3	3	$P6_42'2'$	181.177	3	3	P_c6_422	181.178	6	6
$P6_322$	182.179	4	2	$P6_3221'$	182.180	4	4	$P6_3'2'2$	182.181	4	2	$P6_3'22'$	182.182	2	2
$P6_32'2'$	182.183	2	2	P_c6_322	182.184	6	2	$P6mm$	183.185	4	2	$P6mm1'$	183.186	6	2
$P6'm'm$	183.187	3	1	$P6'mm'$	183.188	4	1	$P6m'm'$	183.189	3	1	P_c6mm	183.190	8	4
$P6cc$	184.191	6	2	$P6cc1'$	184.192	12	4	$P6'c'c$	184.193	6	2	$P6'cc'$	184.194	6	2
$P6c'c'$	184.195	6	2	P_c6cc	184.196	6	2	$P6_3cm$	185.197	4	2	$P6_3cm1'$	185.198	8	4
$P6_3'c'm$	185.199	4	2	$P6_3'cm'$	185.200	4	2	$P6_3c'm'$	185.201	4	2	P_c6_3cm	185.202	6	2
$P6_3mc$	186.203	4	2	$P6_3mc1'$	186.204	4	4	$P6_3'm'c$	186.205	2	2	$P6_3'mc'$	186.206	4	2
$P6_3m'c'$	186.207	2	2	P_c6_3mc	186.208	8	2	$P6m2$	187.209	2	2	$P6m21'$	187.210	2	2
$P6'm'2$	187.211	2	1	$P6'm2'$	187.212	2	1	$P6m'2'$	187.213	1	1	P_c6m2	187.214	4	2
$P6c2$	188.215	2	2	$P6c21'$	188.216	4	4	$P6'c'2$	188.217	4	2	$P6'c2'$	188.218	2	2
$P6c'2'$	188.219	2	2	P_c6c2	188.220	2	2	$P62m$	189.221	2	2	$P62m1'$	189.222	4	2
$P6'2'm$	189.223	4	1	$P6'2m'$	189.224	4	1	$P62'm'$	189.225	2	1	P_c62m	189.226	4	2
$P62c$	190.227	2	2	$P62c1'$	190.228	4	4	$P6'2'c$	190.229	4	2	$P6'2c'$	190.230	4	2
$P62'c'$	190.231	2	2	P_c62c	190.232	4	2	$P6/mmm$	191.233	6	2	$P6/mmm1'$	191.234	6	2
$P6/m'mm$	191.235	4	2	$P6'/mm'm$	191.236	2	2	$P6'/mmm'$	191.237	4	2	$P6'/m'm'm$	191.238	4	1
$P6'/m'mm'$	191.239	4	1	$P6/mm'm'$	191.240	3	1	$P6/m'm'm'$	191.241	4	2	P_c6/mmm	191.242	12	4

$P6/mcc$	192.243	6	2	$P6/mcc1'$	192.244	12	4	$P6/m'cc$	192.245	6	2	$P6'/mc'c$	192.246	6	2
$P6'/mcc'$	192.247	6	2	$P6'/m'c'c$	192.248	8	2	$P6'/m'cc'$	192.249	6	2	$P6/mc'c'$	192.250	6	2
$P6/m'c'c'$	192.251	8	4	P_c6/mcc	192.252	6	2	$P6_3/mcm$	193.253	6	2	$P6_3/mcm1'$	193.254	12	4
$P6_3/m'cm$	193.255	4	2	$P6_3'/mc'm$	193.256	4	4	$P6_3'/mcm'$	193.257	4	2	$P6_3'/m'c'm$	193.258	8	2
$P6_3'/m'cm'$	193.259	6	2	$P6_3/mc'm'$	193.260	6	2	$P6_3/m'c'm'$	193.261	8	2	P_c6_3/mcm	193.262	6	2
$P6_3/mmc$	194.263	6	2	$P6_3/mmc1'$	194.264	12	4	$P6_3/m'mc$	194.265	4	2	$P6_3'/mm'c$	194.266	2	2
$P6_3'/mmc'$	194.267	4	4	$P6_3'/m'm'c$	194.268	6	2	$P6_3'/m'mc'$	194.269	6	2	$P6_3/mm'c'$	194.270	6	2
$P6_3/m'm'c'$	194.271	4	2	P_c6_3/mmc	194.272	8	2	$P23$	195.1	2	2	$P231'$	195.2	6	2
P_I23	195.3	4	4	$F23$	196.4	2	2	$F231'$	196.5	4	2	F_S23	196.6	6	4
$I23$	197.7	2	2	$I231'$	197.8	6	2	$P2_13$	198.9	4	4	$P2_131'$	198.10	8	8
P_I2_13	198.11	12	8	$I2_13$	199.12	6	4	$I2_131'$	199.13	12	8	$Pm3$	200.14	6	2
$Pm31'$	200.15	6	2	$Pm'3'$	200.16	4	2	P_Im3	200.17	12	4	$Pn3$	201.18	4	4
$Pn31'$	201.19	12	4	$Pn'3'$	201.20	4	4	P_In3	201.21	8	4	$Fm3$	202.22	6	2
$Fm31'$	202.23	12	2	$Fm'3'$	202.24	4	2	F_Sm3	202.25	8	4	$Fd3$	203.26	4	4
$Fd31'$	203.27	8	4	$Fd'3'$	203.28	4	4	F_Sd3	203.29	12	8	$Im3$	204.30	6	2
$Im31'$	204.31	8	2	$Im'3'$	204.32	4	2	$Pa3$	205.33	4	4	$Pa31'$	205.34	8	8
$Pa'3'$	205.35	8	8	P_Ia3	205.36	24	8	$Ia3$	206.37	12	4	$Ia31'$	206.38	24	8
$Ia'3'$	206.39	12	8	$P432$	207.40	4	2	$P4321'$	207.41	6	2	$P4'32'$	207.42	6	2
P_I432	207.43	12	4	$P4_232$	208.44	4	4	$P4_2321'$	208.45	12	4	$P4_2'32'$	208.46	6	4
P_I4_232	208.47	12	4	$F432$	209.48	4	2	$F4321'$	209.49	12	2	$F4'32'$	209.50	6	2
F_S432	209.51	12	4	$F4_132$	210.52	4	4	$F4_1321'$	210.53	8	4	$F4_1'32'$	210.54	4	4
F_S4_132	210.55	12	8	$I432$	211.56	4	2	$I4321'$	211.57	12	2	$I4'32'$	211.58	6	2
$P4_332$	212.59	8	4	$P4_3321'$	212.60	8	8	$P4_3'32'$	212.61	4	4	P_I4_332	212.62	12	8
$P4_132$	213.63	8	4	$P4_1321'$	213.64	8	8	$P4_1'32'$	213.65	4	4	P_I4_132	213.66	12	8
$I4_132$	214.67	8	4	$I4_1321'$	214.68	12	8	$I4_1'32'$	214.69	6	4	$P43m$	215.70	6	2
$P43m1'$	215.71	6	2	$P4'3m'$	215.72	2	2	P_I43m	215.73	12	4	$F43m$	216.74	4	2
$F43m1'$	216.75	4	2	$F4'3m'$	216.76	2	2	F_S43m	216.77	12	4	$I43m$	217.78	6	2
$I43m1'$	217.79	12	2	$I4'3m'$	217.80	2	2	$P43n$	218.81	6	4	$P43n1'$	218.82	12	4
$P4'3n'$	218.83	4	4	P_I43n	218.84	12	4	$F43c$	219.85	6	4	$F43c1'$	219.86	12	4
$F4'3c'$	219.87	4	4	F_S43c	219.88	6	4	$I43d$	220.89	8	6	$I43d1'$	220.90	16	12
$I4'3d'$	220.91	12	8	$Pm3m$	221.92	6	2	$Pm3m1'$	221.93	6	2	$Pm'3'm$	221.94	6	2
$Pm3m'$	221.95	6	2	$Pm'3'm'$	221.96	4	2	P_Im3m	221.97	24	4	$Pn3n$	222.98	12	4
$Pn3n1'$	222.99	24	4	$Pn'3'n$	222.100	12	4	$Pn3n'$	222.101	12	4	$Pn'3'n'$	222.102	8	4
P_In3n	222.103	12	4	$Pm3n$	223.104	12	4	$Pm3n1'$	223.105	16	4	$Pm'3'n$	223.106	8	4
$Pm3n'$	223.107	12	4	$Pm'3'n'$	223.108	8	4	P_Im3n	223.109	24	4	$Pn3m$	224.110	12	4
$Pn3m1'$	224.111	24	4	$Pn'3'm$	224.112	12	4	$Pn3m'$	224.113	12	4	$Pn'3'm'$	224.114	4	4
P_In3m	224.115	16	4	$Fm3m$	225.116	12	2	$Fm3m1'$	225.117	12	2	$Fm'3'm$	225.118	4	2
$Fm3m'$	225.119	6	2	$Fm'3'm'$	225.120	4	2	F_Sm3m	225.121	12	4	$Fm3c$	226.122	12	4
$Fm3c1'$	226.123	12	4	$Fm'3'c$	226.124	12	4	$Fm3c'$	226.125	12	4	$Fm'3'c'$	226.126	8	4
F_Sm3c	226.127	12	4	$Fd3m$	227.128	8	4	$Fd3m1'$	227.129	8	4	$Fd'3'm$	227.130	8	4
$Fd3m'$	227.131	4	4	$Fd'3'm'$	227.132	4	4	F_Sd3m	227.133	24	8	$Fd3c$	228.134	12	8
$Fd3c1'$	228.135	24	8	$Fd'3'c$	228.136	12	8	$Fd3c'$	228.137	8	8	$Fd'3'c'$	228.138	8	8
F_Sd3c	228.139	24	8	$Im3m$	229.140	12	2	$Im3m1'$	229.141	12	2	$Im'3'm$	229.142	8	2
$Im3m'$	229.143	12	2	$Im'3'm'$	229.144	4	2	$Ia3d$	230.145	12	8	$Ia3d1'$	230.146	24	16
$Ia'3'd$	230.147	12	8	$Ia3d'$	230.148	12	8	$Ia'3'd'$	230.149	16	8				

39. Minimal SSG Dependencies for the Double SIs in the 1,651 Double SSGs

In this section, we list the minimal double SSG M with the minimal double SIs on which the double SIs in each double SSG G are dependent (see SN 30 for calculation details).

Supplementary Table 24: Minimal SSG dependencies for the double SIs in the 1,651 double SSGs. In order, the columns in this table list the symbol of the SSG G, the number of G in the BNS setting[67], the type of G (see SN 2), the number of the minimal double SSG M with the minimal double SIs on which the double SIs in G are dependent (see SN 30), the symbol of M, and the type of M. Entries for which $G = M$ are highlighted in bold text. We have omitted double SSGs G for which $|Z^G| = 1$ [see SEq. (217) and the surrounding text].

Double SSG		Type	Minimal SSG		Type	Double SSG		Type	Minimal SSG		Type
**2.4**	$\mathbf{P\bar{1}}$	**I**	**2.4**	$\mathbf{P\bar{1}}$	**I**	**2.5**	$\mathbf{P\bar{1}1'}$	**II**	**2.5**	$\mathbf{P\bar{1}1'}$	**II**
2.7	$P_S\bar{1}$	IV	2.4	$P\bar{1}$	I	**3.1**	$\mathbf{P2}$	**I**	**3.1**	$\mathbf{P2}$	**I**
3.4	P_a2	IV	3.1	$P2$	I	**10.42**	$\mathbf{P2/m}$	**I**	**10.42**	$\mathbf{P2/m}$	**I**
10.43	$P2/m1'$	II	2.5	$P\bar{1}1'$	II	10.46	$P2'/m'$	III	2.4	$P\bar{1}$	I
10.47	P_a2/m	IV	10.42	$P2/m$	I	10.48	P_b2/m	IV	2.4	$P\bar{1}$	I
10.49	P_C2/m	IV	2.4	$P\bar{1}$	I	11.50	$P2_1/m$	I	2.4	$P\bar{1}$	I
11.51	$P2_1/m1'$	II	2.5	$P\bar{1}1'$	II	11.54	$P2_1'/m'$	III	2.4	$P\bar{1}$	I
11.55	P_a2_1/m	IV	2.4	$P\bar{1}$	I	11.56	P_b2_1/m	IV	2.4	$P\bar{1}$	I
11.57	P_C2_1/m	IV	2.4	$P\bar{1}$	I	12.58	$C2/m$	I	2.4	$P\bar{1}$	I
12.59	$C2/m1'$	II	2.5	$P\bar{1}1'$	II	12.62	$C2'/m'$	III	2.4	$P\bar{1}$	I
12.63	C_c2/m	IV	2.4	$P\bar{1}$	I	12.64	C_a2/m	IV	2.4	$P\bar{1}$	I
13.65	$P2/c$	I	2.4	$P\bar{1}$	I	13.66	$P2/c1'$	II	2.5	$P\bar{1}1'$	II
13.69	$P2'/c'$	III	2.4	$P\bar{1}$	I	13.70	P_a2/c	IV	2.4	$P\bar{1}$	I
13.71	P_b2/c	IV	2.4	$P\bar{1}$	I	13.72	P_c2/c	IV	2.4	$P\bar{1}$	I
13.73	P_A2/c	IV	2.4	$P\bar{1}$	I	13.74	P_C2/c	IV	2.4	$P\bar{1}$	I
14.75	$P2_1/c$	I	2.4	$P\bar{1}$	I	14.76	$P2_1/c1'$	II	2.5	$P\bar{1}1'$	II
14.79	$P2_1'/c'$	III	2.4	$P\bar{1}$	I	14.80	P_a2_1/c	IV	2.4	$P\bar{1}$	I
14.81	P_b2_1/c	IV	2.4	$P\bar{1}$	I	14.82	P_c2_1/c	IV	2.4	$P\bar{1}$	I
14.83	P_A2_1/c	IV	2.4	$P\bar{1}$	I	14.84	P_C2_1/c	IV	2.4	$P\bar{1}$	I
15.85	$C2/c$	I	2.4	$P\bar{1}$	I	15.86	$C2/c1'$	II	2.5	$P\bar{1}1'$	II
15.89	$C2'/c'$	III	2.4	$P\bar{1}$	I	15.90	C_c2/c	IV	2.4	$P\bar{1}$	I
15.91	C_a2/c	IV	2.4	$P\bar{1}$	I	16.3	$P2'2'2$	III	3.1	$P2$	I
17.10	$P22'2_1'$	III	3.1	$P2$	I	18.18	$P2_1'2_1'2$	III	3.1	$P2$	I
21.40	$C2'2'2$	III	3.1	$P2$	I	25.60	$Pm'm'2$	III	3.1	$P2$	I
**27.81**	$\mathbf{Pc'c'2}$	**III**	**27.81**	$\mathbf{Pc'c'2}$	**III**	28.91	$Pm'a'2$	III	3.1	$P2$	I
30.117	P_bnc2	IV	27.81	$Pc'c'2$	III	32.138	$Pb'a'2$	III	3.1	$P2$	I
34.163	P_Cnn2	IV	27.81	$Pc'c'2$	III	35.168	$Cm'm'2$	III	3.1	$P2$	I
37.183	$Cc'c'2$	III	27.81	$Pc'c'2$	III	37.185	C_acc2	IV	27.81	$Pc'c'2$	III
39.199	$Ab'm'2$	III	27.81	$Pc'c'2$	III	**41.215**	$\mathbf{Ab'a'2}$	**III**	**41.215**	$\mathbf{Ab'a'2}$	**III**
42.222	$Fm'm'2$	III	27.81	$Pc'c'2$	III	45.238	$Ib'a'2$	III	27.81	$Pc'c'2$	III
**47.249**	$\mathbf{Pmmm}$	**I**	**47.249**	$\mathbf{Pmmm}$	**I**	47.250	$Pmmm1'$	II	2.5	$P\bar{1}1'$	II
47.252	$Pm'm'm$	III	10.42	$P2/m$	I	47.254	P_ammm	IV	47.249	$Pmmm$	I
47.255	P_Cmmm	IV	47.249	$Pmmm$	I	47.256	P_Immm	IV	47.249	$Pmmm$	I
48.257	$Pnnn$	I	2.4	$P\bar{1}$	I	48.258	$Pnnn1'$	II	2.5	$P\bar{1}1'$	II
48.260	$Pn'n'n$	III	2.4	$P\bar{1}$	I	48.262	P_cnnn	IV	2.4	$P\bar{1}$	I
48.263	P_Cnnn	IV	2.4	$P\bar{1}$	I	48.264	P_Innn	IV	2.4	$P\bar{1}$	I
49.265	$Pccm$	I	2.4	$P\bar{1}$	I	49.266	$Pccm1'$	II	2.5	$P\bar{1}1'$	II
49.269	$Pc'c'm$	III	10.42	$P2/m$	I	49.270	$Pc'cm'$	III	2.4	$P\bar{1}$	I
49.272	P_accm	IV	2.4	$P\bar{1}$	I	49.273	P_cccm	IV	2.4	$P\bar{1}$	I
49.274	P_Bccm	IV	2.4	$P\bar{1}$	I	49.275	P_Cccm	IV	2.4	$P\bar{1}$	I
49.276	P_Iccm	IV	2.4	$P\bar{1}$	I	50.277	$Pban$	I	2.4	$P\bar{1}$	I
50.278	$Pban1'$	II	2.5	$P\bar{1}1'$	II	50.281	$Pb'a'n$	III	2.4	$P\bar{1}$	I
50.282	$Pb'an'$	III	2.4	$P\bar{1}$	I	50.284	P_aban	IV	2.4	$P\bar{1}$	I
50.285	P_cban	IV	2.4	$P\bar{1}$	I	50.286	P_Aban	IV	2.4	$P\bar{1}$	I
50.287	P_Cban	IV	2.4	$P\bar{1}$	I	50.288	P_Iban	IV	2.4	$P\bar{1}$	I
51.289	$Pmma$	I	10.42	$P2/m$	I	51.290	$Pmma1'$	II	2.5	$P\bar{1}1'$	II
51.294	$Pm'm'a$	III	2.4	$P\bar{1}$	I	51.295	$Pmm'a'$	III	2.4	$P\bar{1}$	I
51.296	$Pm'ma'$	III	10.42	$P2/m$	I	51.298	P_amma	IV	10.42	$P2/m$	I
51.299	P_bmma	IV	2.4	$P\bar{1}$	I	51.300	P_cmma	IV	10.42	$P2/m$	I
51.301	P_Amma	IV	2.4	$P\bar{1}$	I	51.302	P_Bmma	IV	10.42	$P2/m$	I
51.303	P_Cmma	IV	2.4	$P\bar{1}$	I	51.304	P_Imma	IV	2.4	$P\bar{1}$	I
52.305	$Pnna$	I	2.4	$P\bar{1}$	I	52.306	$Pnna1'$	II	2.5	$P\bar{1}1'$	II
52.310	$Pn'n'a$	III	2.4	$P\bar{1}$	I	52.311	$Pnn'a'$	III	2.4	$P\bar{1}$	I
52.312	$Pn'na'$	III	2.4	$P\bar{1}$	I	52.314	P_anna	IV	2.4	$P\bar{1}$	I
52.315	P_bnna	IV	2.4	$P\bar{1}$	I	52.316	P_cnna	IV	2.4	$P\bar{1}$	I

52.317	P_Anna	IV	2.4	$P1$	I	52.318	P_Bnna	IV	2.4	$P1$	I
52.319	P_Cnna	IV	2.4	$P1$	I	52.320	P_Inna	IV	2.4	$P1$	I
53.321	$Pmna$	I	2.4	$P1$	I	53.322	$Pmna1'$	II	2.5	$P11'$	II
53.326	$Pm'n'a$	III	2.4	$P1$	I	53.327	$Pmn'a'$	III	10.42	$P2/m$	I
53.328	$Pm'na'$	III	2.4	$P1$	I	53.330	P_amna	IV	2.4	$P1$	I
53.331	P_bmna	IV	2.4	$P1$	I	53.332	P_cmna	IV	2.4	$P1$	I
53.333	P_Amna	IV	2.4	$P1$	I	53.334	P_Bmna	IV	2.4	$P1$	I
53.335	P_Cmna	IV	2.4	$P1$	I	53.336	P_Imna	IV	2.4	$P1$	I
54.337	$Pcca$	I	2.4	$P1$	I	54.338	$Pcca1'$	II	2.5	$P11'$	II
**54.342**	**$Pc'c'a$**	**III**	**54.342**	**$Pc'c'a$**	**III**	54.343	$Pcc'a'$	III	2.4	$P1$	I
54.344	$Pc'ca'$	III	2.4	$P1$	I	54.346	P_acca	IV	2.4	$P1$	I
54.347	P_bcca	IV	2.4	$P1$	I	54.348	P_ccca	IV	2.4	$P1$	I
54.349	P_Acca	IV	2.4	$P1$	I	54.350	P_Bcca	IV	2.4	$P1$	I
54.351	P_Ccca	IV	2.4	$P1$	I	54.352	P_Icca	IV	2.4	$P1$	I
55.353	$Pbam$	I	10.42	$P2/m$	I	55.354	$Pbam1'$	II	2.5	$P11'$	II
55.357	$Pb'a'm$	III	10.42	$P2/m$	I	55.358	$Pb'am'$	III	2.4	$P1$	I
55.360	P_abam	IV	10.42	$P2/m$	I	55.361	P_cbam	IV	2.4	$P1$	I
55.362	P_Abam	IV	2.4	$P1$	I	55.363	P_Cbam	IV	10.42	$P2/m$	I
55.364	P_Ibam	IV	2.4	$P1$	I	56.365	$Pccn$	I	2.4	$P1$	I
56.366	$Pccn1'$	II	2.5	$P11'$	II	**56.369**	**$Pc'c'n$**	**III**	**56.369**	**$Pc'c'n$**	**III**
56.370	$Pc'cn'$	III	2.4	$P1$	I	56.372	P_bccn	IV	2.4	$P1$	I
56.373	P_cccn	IV	2.4	$P1$	I	56.374	P_Accn	IV	2.4	$P1$	I
56.375	P_Cccn	IV	2.4	$P1$	I	56.376	P_Iccn	IV	2.4	$P1$	I
57.377	$Pbcm$	I	2.4	$P1$	I	57.378	$Pbcm1'$	II	2.5	$P11'$	II
57.382	$Pb'c'm$	III	2.4	$P1$	I	57.383	$Pbc'm'$	III	2.4	$P1$	I
57.384	$Pb'cm'$	III	2.4	$P1$	I	57.386	P_abcm	IV	2.4	$P1$	I
57.387	P_bbcm	IV	2.4	$P1$	I	57.388	P_cbcm	IV	2.4	$P1$	I
57.389	P_Abcm	IV	2.4	$P1$	I	57.390	P_Bbcm	IV	2.4	$P1$	I
57.391	P_Cbcm	IV	2.4	$P1$	I	57.392	P_Ibcm	IV	2.4	$P1$	I
58.393	$Pnnm$	I	2.4	$P1$	I	58.394	$Pnnm1'$	II	2.5	$P11'$	II
58.397	$Pn'n'm$	III	10.42	$P2/m$	I	58.398	$Pnn'm'$	III	2.4	$P1$	I
58.400	P_annm	IV	2.4	$P1$	I	58.401	P_cnnm	IV	2.4	$P1$	I
58.402	P_Bnnm	IV	2.4	$P1$	I	58.403	P_Cnnm	IV	2.4	$P1$	I
58.404	P_Innm	IV	2.4	$P1$	I	59.405	$Pmmn$	I	2.4	$P1$	I
59.406	$Pmmn1'$	II	2.5	$P11'$	II	59.409	$Pm'm'n$	III	2.4	$P1$	I
59.410	$Pmm'n'$	III	2.4	$P1$	I	59.412	P_bmmn	IV	2.4	$P1$	I
59.413	P_cmmn	IV	2.4	$P1$	I	59.414	P_Bmmn	IV	2.4	$P1$	I
59.415	P_Cmmn	IV	2.4	$P1$	I	59.416	P_Immn	IV	2.4	$P1$	I
60.417	$Pbcn$	I	2.4	$P1$	I	60.418	$Pbcn1'$	II	2.5	$P11'$	II
60.422	$Pb'c'n$	III	2.4	$P1$	I	60.423	$Pbc'n'$	III	2.4	$P1$	I
**60.424**	**$Pb'cn'$**	**III**	**60.424**	**$Pb'cn'$**	**III**	60.426	P_abcn	IV	2.4	$P1$	I
60.427	P_bbcn	IV	2.4	$P1$	I	60.428	P_cbcn	IV	2.4	$P1$	I
60.429	P_Abcn	IV	2.4	$P1$	I	60.430	P_Bbcn	IV	2.4	$P1$	I
60.431	P_Cbcn	IV	2.4	$P1$	I	60.432	P_Ibcn	IV	2.4	$P1$	I
61.433	$Pbca$	I	2.4	$P1$	I	61.434	$Pbca1'$	II	2.5	$P11'$	II
61.436	$Pb'c'a$	III	2.4	$P1$	I	61.438	P_abca	IV	2.4	$P1$	I
61.439	P_Cbca	IV	2.4	$P1$	I	61.440	P_Ibca	IV	2.4	$P1$	I
62.441	$Pnma$	I	2.4	$P1$	I	62.442	$Pnma1'$	II	2.5	$P11'$	II
62.446	$Pn'm'a$	III	2.4	$P1$	I	62.447	$Pnm'a'$	III	2.4	$P1$	I
62.448	$Pn'ma'$	III	2.4	$P1$	I	62.450	P_anma	IV	2.4	$P1$	I
62.451	P_bnma	IV	2.4	$P1$	I	62.452	P_cnma	IV	2.4	$P1$	I
62.453	P_Anma	IV	2.4	$P1$	I	62.454	P_Bnma	IV	2.4	$P1$	I
62.455	P_Cnma	IV	2.4	$P1$	I	62.456	P_Inma	IV	2.4	$P1$	I
63.457	$Cmcm$	I	2.4	$P1$	I	63.458	$Cmcm1'$	II	2.5	$P11'$	II
63.462	$Cm'c'm$	III	2.4	$P1$	I	63.463	$Cmc'm'$	III	2.4	$P1$	I
63.464	$Cm'cm'$	III	2.4	$P1$	I	63.466	C_cmcm	IV	2.4	$P1$	I
63.467	C_amcm	IV	2.4	$P1$	I	63.468	C_Amcm	IV	2.4	$P1$	I
64.469	$Cmca$	I	2.4	$P1$	I	64.470	$Cmca1'$	II	2.5	$P11'$	II
64.474	$Cm'c'a$	III	2.4	$P1$	I	64.475	$Cmc'a'$	III	2.4	$P1$	I
64.476	$Cm'ca'$	III	2.4	$P1$	I	64.478	C_cmca	IV	2.4	$P1$	I
64.479	C_amca	IV	2.4	$P1$	I	64.480	C_Amca	IV	2.4	$P1$	I
65.481	$Cmmm$	I	47.249	$Pmmm$	I	65.482	$Cmmm1'$	II	2.5	$P11'$	II
65.485	$Cm'm'm$	III	10.42	$P2/m$	I	65.486	$Cmm'm'$	III	2.4	$P1$	I
65.488	C_cmmm	IV	47.249	$Pmmm$	I	65.489	C_ammm	IV	47.249	$Pmmm$	I
65.490	C_Ammm	IV	47.249	$Pmmm$	I	66.491	$Cccm$	I	2.4	$P1$	I
66.492	$Cccm1'$	II	2.5	$P11'$	II	66.495	$Cc'c'm$	III	10.42	$P2/m$	I
66.496	$Ccc'm'$	III	2.4	$P1$	I	66.498	C_cccm	IV	2.4	$P1$	I
66.499	C_accm	IV	2.4	$P1$	I	66.500	C_Accm	IV	2.4	$P1$	I
67.501	$Cmma$	I	2.4	$P1$	I	67.502	$Cmma1'$	II	2.5	$P11'$	II
67.505	$Cm'm'a$	III	2.4	$P1$	I	67.506	$Cmm'a'$	III	2.4	$P1$	I
67.508	C_cmma	IV	2.4	$P1$	I	67.509	C_amma	IV	2.4	$P1$	I
67.510	C_Amma	IV	2.4	$P1$	I	68.511	$Ccca$	I	2.4	$P1$	I
68.512	$Ccca1'$	II	2.5	$P11'$	II	68.515	$Cc'c'a$	III	54.342	$Pc'c'a$	III
68.516	$Ccc'a'$	III	2.4	$P1$	I	68.518	C_ccca	IV	2.4	$P1$	I
68.519	C_acca	IV	2.4	$P1$	I	68.520	C_Acca	IV	2.4	$P1$	I
69.521	$Fmmm$	I	47.249	$Pmmm$	I	69.522	$Fmmm1'$	II	2.5	$P11'$	II
69.524	$Fm'm'm$	III	2.4	$P1$	I	69.526	F_Smmm	IV	47.249	$Pmmm$	I
70.527	$Fddd$	I	2.4	$P1$	I	70.528	$Fddd1'$	II	2.5	$P11'$	II
70.530	$Fd'd'd$	III	2.4	$P1$	I	70.532	F_Sddd	IV	2.4	$P1$	I

71.533	$Immm$	I	47.249	$Pmmm$	I	71.534	$Immm1'$	II	2.5	$P\bar{1}1'$	II
71.536	$Im'm'm$	III	2.4	$P\bar{1}$	I	71.538	I_cmmm	IV	47.249	$Pmmm$	I
72.539	$Ibam$	I	2.4	$P\bar{1}$	I	72.540	$Ibam1'$	II	2.5	$P\bar{1}1'$	II
72.543	$Ib'a'm$	III	2.4	$P\bar{1}$	I	72.544	$Iba'm'$	III	2.4	$P\bar{1}$	I
72.546	I_cbam	IV	2.4	$P\bar{1}$	I	72.547	I_bbam	IV	2.4	$P\bar{1}$	I
73.548	$Ibca$	I	2.4	$P\bar{1}$	I	73.549	$Ibca1'$	II	2.5	$P\bar{1}1'$	II
73.551	$Ib'c'a$	III	2.4	$P\bar{1}$	I	73.553	I_cbca	IV	2.4	$P\bar{1}$	I
74.554	$Imma$	I	2.4	$P\bar{1}$	I	74.555	$Imma1'$	II	2.5	$P\bar{1}1'$	II
74.558	$Im'm'a$	III	2.4	$P\bar{1}$	I	74.559	$Imm'a'$	III	2.4	$P\bar{1}$	I
74.561	I_cmma	IV	2.4	$P\bar{1}$	I	74.562	I_bmma	IV	2.4	$P\bar{1}$	I
**75.1**	$\mathbf{P4}$	**I**	**75.1**	$\mathbf{P4}$	**I**	75.5	P_C4	IV	75.1	$P4$	I
**77.13**	$\mathbf{P4_2}$	**I**	**77.13**	$\mathbf{P4_2}$	**I**	77.17	P_C4_2	IV	77.13	$P4_2$	I
79.25	$I4$	I	75.1	$P4$	I	**81.33**	$\mathbf{P\bar{4}}$	**I**	**81.33**	$\mathbf{P\bar{4}}$	**I**
81.34	$P\bar{4}1'$	II	81.33	$P\bar{4}$	I	81.36	$P_c\bar{4}$	IV	81.33	$P\bar{4}$	I
81.37	$P_C\bar{4}$	IV	81.33	$P\bar{4}$	I	81.38	$P_I\bar{4}$	IV	81.33	$P\bar{4}$	I
82.39	$I\bar{4}$	I	81.33	$P\bar{4}$	I	82.40	$I\bar{4}1'$	II	81.33	$P\bar{4}$	I
82.42	$I_c\bar{4}$	IV	81.33	$P\bar{4}$	I	**83.43**	$\mathbf{P4/m}$	**I**	**83.43**	$\mathbf{P4/m}$	**I**
**83.44**	$\mathbf{P4/m1'}$	**II**	**83.44**	$\mathbf{P4/m1'}$	**II**	**83.45**	$\mathbf{P4'/m}$	**III**	**83.45**	$\mathbf{P4'/m}$	**III**
83.47	$P4'/m'$	III	81.33	$P\bar{4}$	I	83.48	P_c4/m	IV	83.43	$P4/m$	I
83.49	P_C4/m	IV	83.43	$P4/m$	I	83.50	P_I4/m	IV	83.43	$P4/m$	I
**84.51**	$\mathbf{P4_2/m}$	**I**	**84.51**	$\mathbf{P4_2/m}$	**I**	84.52	$P4_2/m1'$	II	2.5	$P\bar{1}1'$	II
84.53	$P4_2'/m$	III	10.42	$P2/m$	I	84.55	$P4_2'/m'$	III	81.33	$P\bar{4}$	I
84.56	P_c4_2/m	IV	83.45	$P4'/m$	III	84.57	P_C4_2/m	IV	84.51	$P4_2/m$	I
84.58	P_I4_2/m	IV	83.45	$P4'/m$	III	85.59	$P4/n$	I	81.33	$P\bar{4}$	I
85.60	$P4/n1'$	II	2.5	$P\bar{1}1'$	II	85.61	$P4'/n$	III	2.4	$P\bar{1}$	I
85.63	$P4'/n'$	III	81.33	$P\bar{4}$	I	85.64	P_c4/n	IV	2.4	$P\bar{1}$	I
85.65	P_C4/n	IV	2.4	$P\bar{1}$	I	85.66	P_I4/n	IV	2.4	$P\bar{1}$	I
86.67	$P4_2/n$	I	81.33	$P\bar{4}$	I	86.68	$P4_2/n1'$	II	2.5	$P\bar{1}1'$	II
86.69	$P4_2'/n$	III	2.4	$P\bar{1}$	I	86.71	$P4_2'/n'$	III	81.33	$P\bar{4}$	I
86.72	P_c4_2/n	IV	2.4	$P\bar{1}$	I	86.73	P_C4_2/n	IV	2.4	$P\bar{1}$	I
86.74	P_I4_2/n	IV	2.4	$P\bar{1}$	I	87.75	$I4/m$	I	83.43	$P4/m$	I
**87.76**	$\mathbf{I4/m1'}$	**II**	**87.76**	$\mathbf{I4/m1'}$	**II**	87.77	$I4'/m$	III	83.45	$P4'/m$	III
87.79	$I4'/m'$	III	81.33	$P\bar{4}$	I	87.80	I_c4/m	IV	83.43	$P4/m$	I
**88.81**	$\mathbf{I4_1/a}$	**I**	**88.81**	$\mathbf{I4_1/a}$	**I**	88.82	$I4_1/a1'$	II	2.5	$P\bar{1}1'$	II
88.83	$I4_1'/a$	III	2.4	$P\bar{1}$	I	88.85	$I4_1'/a'$	III	81.33	$P\bar{4}$	I
88.86	I_c4_1/a	IV	2.4	$P\bar{1}$	I	89.90	$P42'2'$	III	75.1	$P4$	I
90.98	$P42_1'2'$	III	75.1	$P4$	I	93.122	$P4_22'2'$	III	77.13	$P4_2$	I
94.130	$P4_22_1'2'$	III	77.13	$P4_2$	I	97.154	$I42'2'$	III	75.1	$P4$	I
99.167	$P4m'm'$	III	75.1	$P4$	I	100.175	$P4b'm'$	III	75.1	$P4$	I
101.183	$P4_2c'm'$	III	27.81	$Pc'c'2$	III	102.191	$P4_2n'm'$	III	77.13	$P4_2$	I
**103.199**	$\mathbf{P4c'c'}$	**III**	**103.199**	$\mathbf{P4c'c'}$	**III**	104.207	$P4n'c'$	III	27.81	$Pc'c'2$	III
104.209	P_C4nc	IV	103.199	$P4c'c'$	III	105.215	$P4_2m'c'$	III	27.81	$Pc'c'2$	III
106.223	$P4_2b'c'$	III	27.81	$Pc'c'2$	III	107.231	$I4m'm'$	III	27.81	$Pc'c'2$	III
108.237	$I4c'm'$	III	103.199	$P4c'c'$	III	**110.249**	$\mathbf{I4_1c'd'}$	**III**	**110.249**	$\mathbf{I4_1c'd'}$	**III**
111.251	$P\bar{4}2m$	I	81.33	$P\bar{4}$	I	111.252	$P\bar{4}2m1'$	II	81.33	$P\bar{4}$	I
111.255	$P\bar{4}2'm'$	III	81.33	$P\bar{4}$	I	111.256	$P_c\bar{4}2m$	IV	81.33	$P\bar{4}$	I
111.257	$P_C\bar{4}2m$	IV	81.33	$P\bar{4}$	I	111.258	$P_I\bar{4}2m$	IV	81.33	$P\bar{4}$	I
112.259	$P\bar{4}2c$	I	81.33	$P\bar{4}$	I	112.260	$P\bar{4}2c1'$	II	81.33	$P\bar{4}$	I
112.263	$P\bar{4}2'c'$	III	81.33	$P\bar{4}$	I	112.264	$P_c\bar{4}2c$	IV	81.33	$P\bar{4}$	I
112.265	$P_C\bar{4}2c$	IV	81.33	$P\bar{4}$	I	112.266	$P_I\bar{4}2c$	IV	81.33	$P\bar{4}$	I
113.267	$P\bar{4}2_1m$	I	81.33	$P\bar{4}$	I	113.268	$P\bar{4}2_1m1'$	II	81.33	$P\bar{4}$	I
113.271	$P\bar{4}2_1'm'$	III	81.33	$P\bar{4}$	I	113.272	$P_c\bar{4}2_1m$	IV	81.33	$P\bar{4}$	I
113.273	$P_C\bar{4}2_1m$	IV	81.33	$P\bar{4}$	I	113.274	$P_I\bar{4}2_1m$	IV	81.33	$P\bar{4}$	I
114.275	$P\bar{4}2_1c$	I	81.33	$P\bar{4}$	I	114.276	$P\bar{4}2_1c1'$	II	81.33	$P\bar{4}$	I
114.279	$P\bar{4}2_1'c'$	III	81.33	$P\bar{4}$	I	114.280	$P_c\bar{4}2_1c$	IV	81.33	$P\bar{4}$	I
114.281	$P_C\bar{4}2_1c$	IV	81.33	$P\bar{4}$	I	114.282	$P_I\bar{4}2_1c$	IV	81.33	$P\bar{4}$	I
115.283	$P\bar{4}m2$	I	81.33	$P\bar{4}$	I	115.284	$P\bar{4}m21'$	II	81.33	$P\bar{4}$	I
115.287	$P\bar{4}m'2'$	III	81.33	$P\bar{4}$	I	115.288	$P_c\bar{4}m2$	IV	81.33	$P\bar{4}$	I
115.289	$P_C\bar{4}m2$	IV	81.33	$P\bar{4}$	I	115.290	$P_I\bar{4}m2$	IV	81.33	$P\bar{4}$	I
116.291	$P\bar{4}c2$	I	81.33	$P\bar{4}$	I	116.292	$P\bar{4}c21'$	II	81.33	$P\bar{4}$	I
116.295	$P\bar{4}c'2'$	III	81.33	$P\bar{4}$	I	116.296	$P_c\bar{4}c2$	IV	81.33	$P\bar{4}$	I
116.297	$P_C\bar{4}c2$	IV	81.33	$P\bar{4}$	I	116.298	$P_I\bar{4}c2$	IV	81.33	$P\bar{4}$	I
117.299	$P\bar{4}b2$	I	81.33	$P\bar{4}$	I	117.300	$P\bar{4}b21'$	II	81.33	$P\bar{4}$	I
117.303	$P\bar{4}b'2'$	III	81.33	$P\bar{4}$	I	117.304	$P_c\bar{4}b2$	IV	81.33	$P\bar{4}$	I
117.305	$P_C\bar{4}b2$	IV	81.33	$P\bar{4}$	I	117.306	$P_I\bar{4}b2$	IV	81.33	$P\bar{4}$	I
118.307	$P\bar{4}n2$	I	81.33	$P\bar{4}$	I	118.308	$P\bar{4}n21'$	II	81.33	$P\bar{4}$	I
118.311	$P\bar{4}n'2'$	III	81.33	$P\bar{4}$	I	118.312	$P_c\bar{4}n2$	IV	81.33	$P\bar{4}$	I
118.313	$P_C\bar{4}n2$	IV	81.33	$P\bar{4}$	I	118.314	$P_I\bar{4}n2$	IV	81.33	$P\bar{4}$	I
119.315	$I\bar{4}m2$	I	81.33	$P\bar{4}$	I	119.316	$I\bar{4}m21'$	II	81.33	$P\bar{4}$	I
119.319	$I\bar{4}m'2'$	III	81.33	$P\bar{4}$	I	119.320	$I_c\bar{4}m2$	IV	81.33	$P\bar{4}$	I
120.321	$I\bar{4}c2$	I	81.33	$P\bar{4}$	I	120.322	$I\bar{4}c21'$	II	81.33	$P\bar{4}$	I
120.325	$I\bar{4}c'2'$	III	81.33	$P\bar{4}$	I	120.326	$I_c\bar{4}c2$	IV	81.33	$P\bar{4}$	I
121.327	$I\bar{4}2m$	I	81.33	$P\bar{4}$	I	121.328	$I\bar{4}2m1'$	II	81.33	$P\bar{4}$	I
121.331	$I\bar{4}2'm'$	III	81.33	$P\bar{4}$	I	121.332	$I_c\bar{4}2m$	IV	81.33	$P\bar{4}$	I
122.333	$I\bar{4}2d$	I	81.33	$P\bar{4}$	I	122.334	$I\bar{4}2d1'$	II	81.33	$P\bar{4}$	I
122.337	$I\bar{4}2'd'$	III	81.33	$P\bar{4}$	I	122.338	$I_c\bar{4}2d$	IV	81.33	$P\bar{4}$	I
**123.339**	$\mathbf{P4/mmm}$	**I**	**123.339**	$\mathbf{P4/mmm}$	**I**	123.340	$P4/mmm1'$	II	83.44	$P4/m1'$	II

123.342	$P4'/mm'm$	III	47.249	$Pmmm$	I	123.343	$P4'/mmm'$	III	47.249	$Pmmm$	I
123.344	$P4'/m'm'm$	III	81.33	$P\bar{4}$	I	123.345	$P4/mm'm'$	III	83.43	$P4/m$	I
123.346	$P4'/m'mm'$	III	81.33	$P\bar{4}$	I	123.348	P_c4/mmm	IV	123.339	$P4/mmm$	I
123.349	P_C4/mmm	IV	123.339	$P4/mmm$	I	123.350	P_I4/mmm	IV	123.339	$P4/mmm$	I
124.351	$P4/mcc$	I	83.43	$P4/m$	I	124.352	$P4/mcc1'$	II	83.44	$P4/m1'$	II
124.354	$P4'/mc'c$	III	83.45	$P4'/m$	III	124.355	$P4'/mcc'$	III	83.45	$P4'/m$	III
124.356	$P4'/m'c'c$	III	81.33	$P\bar{4}$	I	124.357	$P4/mc'c'$	III	83.43	$P4/m$	I
124.358	$P4'/m'cc'$	III	81.33	$P\bar{4}$	I	124.360	P_c4/mcc	IV	83.43	$P4/m$	I
124.361	P_C4/mcc	IV	83.43	$P4/m$	I	124.362	P_I4/mcc	IV	83.43	$P4/m$	I
125.363	$P4/nbm$	I	2.4	$P\bar{1}$	I	125.364	$P4/nbm1'$	II	2.5	$P\bar{1}1'$	II
125.366	$P4'/nb'm$	III	2.4	$P\bar{1}$	I	125.367	$P4'/nbm'$	III	2.4	$P\bar{1}$	I
125.368	$P4'/n'b'm$	III	81.33	$P\bar{4}$	I	125.369	$P4/nb'm'$	III	81.33	$P\bar{4}$	I
125.370	$P4'/n'bm'$	III	81.33	$P\bar{4}$	I	125.372	P_c4/nbm	IV	2.4	$P\bar{1}$	I
125.373	P_C4/nbm	IV	2.4	$P\bar{1}$	I	125.374	P_I4/nbm	IV	2.4	$P\bar{1}$	I
126.375	$P4/nnc$	I	2.4	$P\bar{1}$	I	126.376	$P4/nnc1'$	II	2.5	$P\bar{1}1'$	II
126.378	$P4'/nn'c$	III	2.4	$P\bar{1}$	I	126.379	$P4'/nnc'$	III	2.4	$P\bar{1}$	I
126.380	$P4'/n'n'c$	III	81.33	$P\bar{4}$	I	126.381	$P4/nn'c'$	III	54.342	$Pc'c'a$	III
126.382	$P4'/n'nc'$	III	81.33	$P\bar{4}$	I	126.384	P_c4/nnc	IV	2.4	$P\bar{1}$	I
126.385	P_C4/nnc	IV	2.4	$P\bar{1}$	I	126.386	P_I4/nnc	IV	2.4	$P\bar{1}$	I
127.387	$P4/mbm$	I	83.43	$P4/m$	I	127.388	$P4/mbm1'$	II	83.44	$P4/m1'$	II
127.390	$P4'/mb'm$	III	47.249	$Pmmm$	I	127.391	$P4'/mbm'$	III	83.45	$P4'/m$	III
127.392	$P4'/m'b'm$	III	81.33	$P\bar{4}$	I	127.393	$P4/mb'm'$	III	83.43	$P4/m$	I
127.394	$P4'/m'bm'$	III	81.33	$P\bar{4}$	I	127.396	P_c4/mbm	IV	47.249	$Pmmm$	I
127.397	P_C4/mbm	IV	83.43	$P4/m$	I	127.398	P_I4/mbm	IV	47.249	$Pmmm$	I
128.399	$P4/mnc$	I	83.43	$P4/m$	I	128.400	$P4/mnc1'$	II	83.44	$P4/m1'$	II
128.402	$P4'/mn'c$	III	83.45	$P4'/m$	III	128.403	$P4'/mnc'$	III	83.45	$P4'/m$	III
128.404	$P4'/m'n'c$	III	81.33	$P\bar{4}$	I	128.405	$P4/mn'c'$	III	83.43	$P4/m$	I
128.406	$P4'/m'nc'$	III	81.33	$P\bar{4}$	I	128.408	P_c4/mnc	IV	83.43	$P4/m$	I
128.409	P_C4/mnc	IV	83.43	$P4/m$	I	128.410	P_I4/mnc	IV	83.43	$P4/m$	I
129.411	$P4/nmm$	I	2.4	$P\bar{1}$	I	129.412	$P4/nmm1'$	II	2.5	$P\bar{1}1'$	II
129.414	$P4'/nm'm$	III	2.4	$P\bar{1}$	I	129.415	$P4'/nmm'$	III	2.4	$P\bar{1}$	I
129.416	$P4'/n'm'm$	III	81.33	$P\bar{4}$	I	129.417	$P4/nm'm'$	III	81.33	$P\bar{4}$	I
129.418	$P4'/n'mm'$	III	81.33	$P\bar{4}$	I	129.420	P_c4/nmm	IV	2.4	$P\bar{1}$	I
129.421	P_C4/nmm	IV	2.4	$P\bar{1}$	I	129.422	P_I4/nmm	IV	2.4	$P\bar{1}$	I
130.423	$P4/ncc$	I	2.4	$P\bar{1}$	I	130.424	$P4/ncc1'$	II	2.5	$P\bar{1}1'$	II
130.426	$P4'/nc'c$	III	2.4	$P\bar{1}$	I	130.427	$P4'/ncc'$	III	2.4	$P\bar{1}$	I
130.428	$P4'/n'c'c$	III	81.33	$P\bar{4}$	I	**130.429**	$\mathbf{P4/nc'c'}$	**III**	**130.429**	$\mathbf{P4/nc'c'}$	**III**
130.430	$P4'/n'cc'$	III	81.33	$P\bar{4}$	I	130.432	P_c4/ncc	IV	2.4	$P\bar{1}$	I
130.433	P_C4/ncc	IV	2.4	$P\bar{1}$	I	130.434	P_I4/ncc	IV	2.4	$P\bar{1}$	I
131.435	$P4_2/mmc$	I	47.249	$Pmmm$	I	131.436	$P4_2/mmc1'$	II	2.5	$P\bar{1}1'$	II
131.438	$P4_2'/mm'c$	III	2.4	$P\bar{1}$	I	131.439	$P4_2'/mmc'$	III	47.249	$Pmmm$	I
131.440	$P4_2'/m'm'c$	III	81.33	$P\bar{4}$	I	131.441	$P4_2/mm'c'$	III	84.51	$P4_2/m$	I
131.442	$P4_2'/m'mc'$	III	81.33	$P\bar{4}$	I	131.444	P_c4_2/mmc	IV	47.249	$Pmmm$	I
131.445	P_C4_2/mmc	IV	47.249	$Pmmm$	I	131.446	P_I4_2/mmc	IV	47.249	$Pmmm$	I
132.447	$P4_2/mcm$	I	47.249	$Pmmm$	I	132.448	$P4_2/mcm1'$	II	2.5	$P\bar{1}1'$	II
132.450	$P4_2'/mc'm$	III	47.249	$Pmmm$	I	132.451	$P4_2'/mcm'$	III	2.4	$P\bar{1}$	I
132.452	$P4_2'/m'c'm$	III	81.33	$P\bar{4}$	I	132.453	$P4_2/mc'm'$	III	84.51	$P4_2/m$	I
132.454	$P4_2'/m'cm'$	III	81.33	$P\bar{4}$	I	132.456	P_c4_2/mcm	IV	47.249	$Pmmm$	I
132.457	P_C4_2/mcm	IV	47.249	$Pmmm$	I	132.458	P_I4_2/mcm	IV	47.249	$Pmmm$	I
133.459	$P4_2/nbc$	I	2.4	$P\bar{1}$	I	133.460	$P4_2/nbc1'$	II	2.5	$P\bar{1}1'$	II
133.462	$P4_2'/nb'c$	III	2.4	$P\bar{1}$	I	133.463	$P4_2'/nbc'$	III	2.4	$P\bar{1}$	I
133.464	$P4_2'/n'b'c$	III	81.33	$P\bar{4}$	I	133.465	$P4_2/nb'c'$	III	54.342	$Pc'c'a$	III
133.466	$P4_2'/n'bc'$	III	81.33	$P\bar{4}$	I	133.468	P_c4_2/nbc	IV	2.4	$P\bar{1}$	I
133.469	P_C4_2/nbc	IV	2.4	$P\bar{1}$	I	133.470	P_I4_2/nbc	IV	2.4	$P\bar{1}$	I
134.471	$P4_2/nnm$	I	2.4	$P\bar{1}$	I	134.472	$P4_2/nnm1'$	II	2.5	$P\bar{1}1'$	II
134.474	$P4_2'/nn'm$	III	2.4	$P\bar{1}$	I	134.475	$P4_2'/nnm'$	III	2.4	$P\bar{1}$	I
134.476	$P4_2'/n'n'm$	III	81.33	$P\bar{4}$	I	134.477	$P4_2/nn'm'$	III	81.33	$P\bar{4}$	I
134.478	$P4_2'/n'nm'$	III	81.33	$P\bar{4}$	I	134.480	P_c4_2/nnm	IV	2.4	$P\bar{1}$	I
134.481	P_C4_2/nnm	IV	2.4	$P\bar{1}$	I	134.482	P_I4_2/nnm	IV	2.4	$P\bar{1}$	I
135.483	$P4_2/mbc$	I	84.51	$P4_2/m$	I	135.484	$P4_2/mbc1'$	II	2.5	$P\bar{1}1'$	II
135.486	$P4_2'/mb'c$	III	2.4	$P\bar{1}$	I	**135.487**	$\mathbf{P4_2'/mbc'}$	**III**	**135.487**	$\mathbf{P4_2'/mbc'}$	**III**
135.488	$P4_2'/m'b'c$	III	81.33	$P\bar{4}$	I	135.489	$P4_2/mb'c'$	III	84.51	$P4_2/m$	I
135.490	$P4_2'/m'bc'$	III	81.33	$P\bar{4}$	I	135.492	P_c4_2/mbc	IV	83.45	$P4'/m$	III
135.493	P_C4_2/mbc	IV	84.51	$P4_2/m$	I	135.494	P_I4_2/mbc	IV	83.45	$P4'/m$	III
136.495	$P4_2/mnm$	I	47.249	$Pmmm$	I	136.496	$P4_2/mnm1'$	II	2.5	$P\bar{1}1'$	II
136.498	$P4_2'/mn'm$	III	47.249	$Pmmm$	I	136.499	$P4_2'/mnm'$	III	2.4	$P\bar{1}$	I
136.500	$P4_2'/m'n'm$	III	81.33	$P\bar{4}$	I	136.501	$P4_2/mn'm'$	III	84.51	$P4_2/m$	I
136.502	$P4_2'/m'nm'$	III	81.33	$P\bar{4}$	I	136.504	P_c4_2/mnm	IV	47.249	$Pmmm$	I

136.505	P_C4_2/mnm	IV	47.249	$Pmmm$	I	136.506	P_I4_2/mnm	IV	47.249	$Pmmm$	I
137.507	$P4_2/nmc$	I	2.4	$P\bar{1}$	I	137.508	$P4_2/nmc1'$	II	2.5	$P\bar{1}1'$	II
137.510	$P4'_2/nm'c$	III	2.4	$P\bar{1}$	I	137.511	$P4'_2/nmc'$	III	2.4	$P\bar{1}$	I
137.512	$P4'_2/n'm'c$	III	81.33	$P\bar{4}$	I	137.513	$P4_2/nm'c'$	III	54.342	$Pc'c'a$	III
137.514	$P4'_2/n'mc'$	III	81.33	$P\bar{4}$	I	137.516	P_c4_2/nmc	IV	2.4	$P\bar{1}$	I
137.517	P_C4_2/nmc	IV	2.4	$P\bar{1}$	I	137.518	P_I4_2/nmc	IV	2.4	$P\bar{1}$	I
138.519	$P4_2/ncm$	I	2.4	$P\bar{1}$	I	138.520	$P4_2/ncm1'$	II	2.5	$P\bar{1}1'$	II
138.522	$P4'_2/nc'm$	III	2.4	$P\bar{1}$	I	138.523	$P4'_2/ncm'$	III	2.4	$P\bar{1}$	I
138.524	$P4'_2/n'c'm$	III	81.33	$P\bar{4}$	I	138.525	$P4_2/nc'm'$	III	56.369	$Pc'c'n$	III
138.526	$P4'_2/n'cm'$	III	81.33	$P\bar{4}$	I	138.528	P_c4_2/ncm	IV	2.4	$P\bar{1}$	I
138.529	P_C4_2/ncm	IV	2.4	$P\bar{1}$	I	138.530	P_I4_2/ncm	IV	2.4	$P\bar{1}$	I
139.531	$I4/mmm$	I	123.339	$P4/mmm$	I	139.532	$I4/mmm1'$	II	87.76	$I4/m1'$	II
139.534	$I4'/mm'm$	III	47.249	$Pmmm$	I	139.535	$I4'/mmm'$	III	47.249	$Pmmm$	I
139.536	$I4'/m'm'm$	III	81.33	$P\bar{4}$	I	139.537	$I4/mm'm'$	III	83.43	$P4/m$	I
139.538	$I4'/m'mm'$	III	81.33	$P\bar{4}$	I	139.540	I_c4/mmm	IV	123.339	$P4/mmm$	I
140.541	$I4/mcm$	I	47.249	$Pmmm$	I	140.542	$I4/mcm1'$	II	87.76	$I4/m1'$	II
140.544	$I4'/mc'm$	III	47.249	$Pmmm$	I	140.545	$I4'/mcm'$	III	83.45	$P4'/m$	III
140.546	$I4'/m'c'm$	III	81.33	$P\bar{4}$	I	140.547	$I4/mc'm'$	III	83.43	$P4/m$	I
140.548	$I4'/m'cm'$	III	81.33	$P\bar{4}$	I	140.550	I_c4/mcm	IV	47.249	$Pmmm$	I
141.551	$I4_1/amd$	I	2.4	$P\bar{1}$	I	141.552	$I4_1/amd1'$	II	2.5	$P\bar{1}1'$	II
141.554	$I4'_1/am'd$	III	2.4	$P\bar{1}$	I	141.555	$I4'_1/amd'$	III	2.4	$P\bar{1}$	I
141.556	$I4'_1/a'm'd$	III	81.33	$P\bar{4}$	I	141.557	$I4_1/am'd'$	III	88.81	$I4_1/a$	I
141.558	$I4'_1/a'md'$	III	81.33	$P\bar{4}$	I	141.560	I_c4_1/amd	IV	2.4	$P\bar{1}$	I
142.561	$I4_1/acd$	I	2.4	$P\bar{1}$	I	142.562	$I4_1/acd1'$	II	2.5	$P\bar{1}1'$	II
142.564	$I4'_1/ac'd$	III	2.4	$P\bar{1}$	I	142.565	$I4'_1/acd'$	III	2.4	$P\bar{1}$	I
142.566	$I4'_1/a'c'd$	III	81.33	$P\bar{4}$	I	142.567	$I4_1/ac'd'$	III	88.81	$I4_1/a$	I
142.568	$I4'_1/a'cd'$	III	81.33	$P\bar{4}$	I	142.570	I_c4_1/acd	IV	2.4	$P\bar{1}$	I
**143.1**	$\mathbf{P3}$	**I**	**143.1**	$\mathbf{P3}$	**I**	**147.13**	$\mathbf{P\bar{3}}$	**I**	**147.13**	$\mathbf{P\bar{3}}$	**I**
147.14	$P\bar{3}1'$	II	2.5	$P\bar{1}1'$	II	147.16	$P_c\bar{3}$	IV	2.4	$P\bar{1}$	I
148.17	$R\bar{3}$	I	2.4	$P\bar{1}$	I	148.18	$R\bar{3}1'$	II	2.5	$P\bar{1}1'$	II
148.20	$R_I\bar{3}$	IV	2.4	$P\bar{1}$	I	149.23	$P312'$	III	143.1	$P3$	I
150.27	$P32'1$	III	143.1	$P3$	I	156.51	$P3m'1$	III	143.1	$P3$	I
157.55	$P31m'$	III	143.1	$P3$	I	158.59	$P3c'1$	III	143.1	$P3$	I
159.63	$P31c'$	III	143.1	$P3$	I	162.73	$P\bar{3}1m$	I	2.4	$P\bar{1}$	I
162.74	$P\bar{3}1m1'$	II	2.5	$P\bar{1}1'$	II	162.77	$P\bar{3}1m'$	III	147.13	$P\bar{3}$	I
162.78	$P_c\bar{3}1m$	IV	2.4	$P\bar{1}$	I	163.79	$P\bar{3}1c$	I	2.4	$P\bar{1}$	I
163.80	$P\bar{3}1c1'$	II	2.5	$P\bar{1}1'$	II	163.83	$P\bar{3}1c'$	III	147.13	$P\bar{3}$	I
163.84	$P_c\bar{3}1c$	IV	2.4	$P\bar{1}$	I	164.85	$P\bar{3}m1$	I	2.4	$P\bar{1}$	I
164.86	$P\bar{3}m11'$	II	2.5	$P\bar{1}1'$	II	164.89	$P\bar{3}m'1$	III	147.13	$P\bar{3}$	I
164.90	$P_c\bar{3}m1$	IV	2.4	$P\bar{1}$	I	165.91	$P\bar{3}c1$	I	2.4	$P\bar{1}$	I
165.92	$P\bar{3}c11'$	II	2.5	$P\bar{1}1'$	II	165.95	$P\bar{3}c'1$	III	147.13	$P\bar{3}$	I
165.96	$P_c\bar{3}c1$	IV	2.4	$P\bar{1}$	I	166.97	$R\bar{3}m$	I	2.4	$P\bar{1}$	I
166.98	$R\bar{3}m1'$	II	2.5	$P\bar{1}1'$	II	166.101	$R\bar{3}m'$	III	2.4	$P\bar{1}$	I
166.102	$R_I\bar{3}m$	IV	2.4	$P\bar{1}$	I	167.103	$R\bar{3}c$	I	2.4	$P\bar{1}$	I
167.104	$R\bar{3}c1'$	II	2.5	$P\bar{1}1'$	II	167.107	$R\bar{3}c'$	III	2.4	$P\bar{1}$	I
167.108	$R_I\bar{3}c$	IV	2.4	$P\bar{1}$	I	**168.109**	$\mathbf{P6}$	**I**	**168.109**	$\mathbf{P6}$	**I**
171.121	$P6_2$	I	3.1	$P2$	I	172.125	$P6_4$	I	3.1	$P2$	I
173.129	$P6_3$	I	143.1	$P3$	I	**174.133**	$\mathbf{P\bar{6}}$	**I**	**174.133**	$\mathbf{P\bar{6}}$	**I**
174.134	$P\bar{6}1'$	II	174.133	$P\bar{6}$	I	174.136	$P_c\bar{6}$	IV	174.133	$P\bar{6}$	I
**175.137**	$\mathbf{P6/m}$	**I**	**175.137**	$\mathbf{P6/m}$	**I**	**175.138**	$\mathbf{P6/m1'}$	**II**	**175.138**	$\mathbf{P6/m1'}$	**II**
175.139	$P6'/m$	III	174.133	$P\bar{6}$	I	175.141	$P6'/m'$	III	2.4	$P\bar{1}$	I
175.142	P_c6/m	IV	175.137	$P6/m$	I	**176.143**	$\mathbf{P6_3/m}$	**I**	**176.143**	$\mathbf{P6_3/m}$	**I**
**176.144**	$\mathbf{P6_3/m1'}$	**II**	**176.144**	$\mathbf{P6_3/m1'}$	**II**	176.145	$P6'_3/m$	III	174.133	$P\bar{6}$	I
176.147	$P6'_3/m'$	III	2.4	$P\bar{1}$	I	176.148	P_c6_3/m	IV	176.143	$P6_3/m$	I
177.153	$P62'2'$	III	168.109	$P6$	I	180.171	$P6_22'2'$	III	3.1	$P2$	I
181.177	$P6_42'2'$	III	3.1	$P2$	I	182.183	$P6_32'2'$	III	143.1	$P3$	I
183.189	$P6m'm'$	III	168.109	$P6$	I	**184.195**	$\mathbf{P6c'c'}$	**III**	**184.195**	$\mathbf{P6c'c'}$	**III**
185.201	$P6_3c'm'$	III	143.1	$P3$	I	186.207	$P6_3m'c'$	III	143.1	$P3$	I
187.209	$P\bar{6}m2$	I	174.133	$P\bar{6}$	I	187.210	$P\bar{6}m21'$	II	174.133	$P\bar{6}$	I
187.213	$P\bar{6}m'2'$	III	174.133	$P\bar{6}$	I	187.214	$P_c\bar{6}m2$	IV	174.133	$P\bar{6}$	I
188.215	$P\bar{6}c2$	I	174.133	$P\bar{6}$	I	188.216	$P\bar{6}c21'$	II	174.133	$P\bar{6}$	I
188.219	$P\bar{6}c'2'$	III	174.133	$P\bar{6}$	I	188.220	$P_c\bar{6}c2$	IV	174.133	$P\bar{6}$	I
189.221	$P\bar{6}2m$	I	174.133	$P\bar{6}$	I	189.222	$P\bar{6}2m1'$	II	174.133	$P\bar{6}$	I
189.225	$P\bar{6}2'm'$	III	174.133	$P\bar{6}$	I	189.226	$P_c\bar{6}2m$	IV	174.133	$P\bar{6}$	I
190.227	$P\bar{6}2c$	I	174.133	$P\bar{6}$	I	190.228	$P\bar{6}2c1'$	II	174.133	$P\bar{6}$	I
190.231	$P\bar{6}2'c'$	III	174.133	$P\bar{6}$	I	190.232	$P_c\bar{6}2c$	IV	174.133	$P\bar{6}$	I
**191.233**	$\mathbf{P6/mmm}$	**I**	**191.233**	$\mathbf{P6/mmm}$	**I**	191.234	$P6/mmm1'$	II	175.138	$P6/m1'$	II
191.236	$P6'/mm'm$	III	174.133	$P\bar{6}$	I	191.237	$P6'/mmm'$	III	174.133	$P\bar{6}$	I
191.238	$P6'/m'm'm$	III	2.4	$P\bar{1}$	I	191.239	$P6'/m'mm'$	III	2.4	$P\bar{1}$	I
191.240	$P6/mm'm'$	III	175.137	$P6/m$	I	191.242	P_c6/mmm	IV	191.233	$P6/mmm$	I
192.243	$P6/mcc$	I	175.137	$P6/m$	I	192.244	$P6/mcc1'$	II	175.138	$P6/m1'$	II
192.246	$P6'/mc'c$	III	174.133	$P\bar{6}$	I	192.247	$P6'/mcc'$	III	174.133	$P\bar{6}$	I
192.248	$P6'/m'c'c$	III	2.4	$P\bar{1}$	I	192.249	$P6'/m'cc'$	III	2.4	$P\bar{1}$	I

192.250	$P6/mc'c'$	III	175.137	$P6/m$	I	192.252	P_c6/mcc	IV	175.137	$P6/m$	I
193.253	$P6_3/mcm$	I	176.143	$P6_3/m$	I	193.254	$P6_3/mcm1'$	II	176.144	$P6_3/m1'$	II
193.256	$P6_3'/mc'm$	III	174.133	$P6$	I	193.257	$P6_3'/mcm'$	III	174.133	$P6$	I
193.258	$P6_3'/m'c'm$	III	2.4	$P1$	I	193.259	$P6_3'/m'cm'$	III	2.4	$P1$	I
193.260	$P6_3/mc'm'$	III	176.143	$P6_3/m$	I	193.262	P_c6_3/mcm	IV	176.143	$P6_3/m$	I
194.263	$P6_3/mmc$	I	176.143	$P6_3/m$	I	194.264	$P6_3/mmc1'$	II	176.144	$P6_3/m1'$	II
194.266	$P6_3'/mm'c$	III	174.133	$P6$	I	194.267	$P6_3'/mmc'$	III	174.133	$P6$	I
194.268	$P6_3'/m'm'c$	III	2.4	$P1$	I	194.269	$P6_3'/m'mc'$	III	2.4	$P1$	I
194.270	$P6_3/mm'c'$	III	176.143	$P6_3/m$	I	194.272	P_c6_3/mmc	IV	176.143	$P6_3/m$	I
200.14	$Pm3$	I	47.249	$Pmmm$	I	200.15	$Pm31'$	II	2.5	$P11'$	II
200.17	P_Im3	IV	47.249	$Pmmm$	I	201.18	$Pn3$	I	2.4	$P1$	I
201.19	$Pn31'$	II	2.5	$P11'$	II	201.21	P_In3	IV	2.4	$P1$	I
202.22	$Fm3$	I	47.249	$Pmmm$	I	202.23	$Fm31'$	II	2.5	$P11'$	II
202.25	F_Sm3	IV	47.249	$Pmmm$	I	203.26	$Fd3$	I	2.4	$P1$	I
203.27	$Fd31'$	II	2.5	$P11'$	II	203.29	F_Sd3	IV	2.4	$P1$	I
204.30	$Im3$	I	47.249	$Pmmm$	I	204.31	$Im31'$	II	2.5	$P11'$	II
205.33	$Pa3$	I	2.4	$P1$	I	205.34	$Pa31'$	II	2.5	$P11'$	II
205.36	P_Ia3	IV	2.4	$P1$	I	206.37	$Ia3$	I	2.4	$P1$	I
206.38	$Ia31'$	II	2.5	$P11'$	II	215.70	$P43m$	I	81.33	$P4$	I
215.71	$P43m1'$	II	81.33	$P4$	I	215.73	P_I43m	IV	81.33	$P4$	I
216.74	$F43m$	I	81.33	$P4$	I	216.75	$F43m1'$	II	81.33	$P4$	I
216.77	F_S43m	IV	81.33	$P4$	I	217.78	$I43m$	I	81.33	$P4$	I
217.79	$I43m1'$	II	81.33	$P4$	I	218.81	$P43n$	I	81.33	$P4$	I
218.82	$P43n1'$	II	81.33	$P4$	I	218.84	P_I43n	IV	81.33	$P4$	I
219.85	$F43c$	I	81.33	$P4$	I	219.86	$F43c1'$	II	81.33	$P4$	I
219.88	F_S43c	IV	81.33	$P4$	I	220.89	$I43d$	I	81.33	$P4$	I
220.90	$I43d1'$	II	81.33	$P4$	I	221.92	$Pm3m$	I	123.339	$P4/mmm$	I
221.93	$Pm3m1'$	II	83.44	$P4/m1'$	II	221.94	$Pm'3'm$	III	81.33	$P4$	I
221.95	$Pm3m'$	III	47.249	$Pmmm$	I	221.97	P_Im3m	IV	123.339	$P4/mmm$	I
222.98	$Pn3n$	I	2.4	$P1$	I	222.99	$Pn3n1'$	II	2.5	$P11'$	II
222.100	$Pn'3'n$	III	81.33	$P4$	I	222.101	$Pn3n'$	III	2.4	$P1$	I
222.103	P_In3n	IV	2.4	$P1$	I	223.104	$Pm3n$	I	47.249	$Pmmm$	I
223.105	$Pm3n1'$	II	2.5	$P11'$	II	223.106	$Pm'3'n$	III	81.33	$P4$	I
223.107	$Pm3n'$	III	47.249	$Pmmm$	I	223.109	P_Im3n	IV	47.249	$Pmmm$	I
224.110	$Pn3m$	I	2.4	$P1$	I	224.111	$Pn3m1'$	II	2.5	$P11'$	II
224.112	$Pn'3'm$	III	81.33	$P4$	I	224.113	$Pn3m'$	III	2.4	$P1$	I
224.115	P_In3m	IV	2.4	$P1$	I	225.116	$Fm3m$	I	123.339	$P4/mmm$	I
225.117	$Fm3m1'$	II	83.44	$P4/m1'$	II	225.118	$Fm'3'm$	III	81.33	$P4$	I
225.119	$Fm3m'$	III	47.249	$Pmmm$	I	225.121	F_Sm3m	IV	123.339	$P4/mmm$	I
226.122	$Fm3c$	I	47.249	$Pmmm$	I	226.123	$Fm3c1'$	II	87.76	$I4/m1'$	II
226.124	$Fm'3'c$	III	81.33	$P4$	I	226.125	$Fm3c'$	III	47.249	$Pmmm$	I
226.127	F_Sm3c	IV	47.249	$Pmmm$	I	227.128	$Fd3m$	I	2.4	$P1$	I
227.129	$Fd3m1'$	II	2.5	$P11'$	II	227.130	$Fd'3'm$	III	81.33	$P4$	I
227.131	$Fd3m'$	III	2.4	$P1$	I	227.133	F_Sd3m	IV	2.4	$P1$	I
228.134	$Fd3c$	I	2.4	$P1$	I	228.135	$Fd3c1'$	II	2.5	$P11'$	II
228.136	$Fd'3'c$	III	81.33	$P4$	I	228.137	$Fd3c'$	III	2.4	$P1$	I
228.139	F_Sd3c	IV	2.4	$P1$	I	229.140	$Im3m$	I	123.339	$P4/mmm$	I
229.141	$Im3m1'$	II	87.76	$I4/m1'$	II	229.142	$Im'3'm$	III	81.33	$P4$	I
229.143	$Im3m'$	III	47.249	$Pmmm$	I	230.145	$Ia3d$	I	2.4	$P1$	I
230.146	$Ia3d1'$	II	2.5	$P11'$	II	230.147	$Ia'3'd$	III	81.33	$P4$	I
230.148	$Ia3d'$	III	2.4	$P1$	I						

SUPPLEMENTARY REFERENCES

[1] Bradlyn, B. *et al.* Topological quantum chemistry. *Nature* **547**, 298–305 (2017). URL https://doi.org/10.1038/nature23268.

[2] Elcoro, L. *et al.* Double crystallographic groups and their representations on the Bilbao Crystallographic Server. *Journal of Applied Crystallography* **50**, 1457–1477 (2017). URL https://doi.org/10.1107/S1600576717011712.

[3] Vergniory, M. G. *et al.* Graph theory data for topological quantum chemistry. *Phys. Rev. E* **96**, 023310 (2017). URL https://link.aps.org/doi/10.1103/PhysRevE.96.023310.

[4] Cano, J. *et al.* Building blocks of topological quantum chemistry: Elementary band representations. *Phys. Rev. B* **97**, 035139 (2018). URL https://link.aps.org/doi/10.1103/PhysRevB.97.035139.

[5] Cano, J. *et al.* Topology of disconnected elementary band representations. *Phys. Rev. Lett.* **120**, 266401 (2018). URL https://link.aps.org/doi/10.1103/PhysRevLett.120.266401.

[6] Bradlyn, B., Wang, Z., Cano, J. & Bernevig, B. A. Disconnected elementary band representations, fragile topology, and Wilson loops as topological indices: An example on the triangular lattice. *Phys. Rev. B* **99**, 045140 (2019). URL https://link.aps.org/doi/10.1103/PhysRevB.99.045140.

[7] Shubnikov, A. V. *Symmetry and Antisymmetry of Finite Figures* (USSR Academy of Sciences, Moscow, USSR, 1951).

[8] Aroyo, M. I., Kirov, A., Capillas, C., Perez-Mato, J. M. & Wondratschek, H. Bilbao Crystallographic Server. II. Representations of crystallographic point groups and space groups. *Acta Crystallographica Section A* **62**, 115–128 (2006). URL https://doi.org/10.1107/S0108767305040286.

[9] Altmann, S. & Herzig, P. *Point-Group Theory Tables* (University of Vienna, 2011), 2 edn. URL http://phaidra.univie.ac.at/o:104731.

[10] Litvin, D. B. *Magnetic Group Tables* (International Union of Crystallography, 2013).

[11] Evarestov, R. & Smirnov, V. *Site Symmetry in Crystals: Theory and Applications*. Springer Series in Solid-State Sciences (Springer Berlin Heidelberg, 2012). URL https://books.google.com/books?id=h-79CAAAQBAJ.

[12] Evarestov, R. A., Smirnov, V. P. & Egorov, S. A. Band corepresentations of magnetic space groups. *physica status solidi (b)* **151**, 275–282 (1989). URL https://onlinelibrary.wiley.com/doi/abs/10.1002/pssb.2221510132.

[13] Aroyo, M. I. *et al.* Bilbao Crystallographic Server: I. Databases and crystallographic computing programs. *Zeitschrift für Kristallographie - Crystalline Materials* **221**, 15 – 27 (2006). URL https://www.degruyter.com/view/journals/zkri/221/1/article-p15.xml.

[14] Aroyo, M. I., Kirov, A., Capillas, C., Perez-Mato, J. M. & Wondratschek, H. Bilbao Crystallographic Server. II. Representations of crystallographic point groups and space groups. *Acta Crystallographica Section A* **62**, 115–128 (2006). URL https://doi.org/10.1107/S0108767305040286.

[15] Gallego, S. V. *et al.* MAGNDATA: towards a database of magnetic structures. I. The commensurate case. *Journal of Applied Crystallography* **49**, 1750–1776 (2016). URL http://scripts.iucr.org/cgi-bin/paper?ks5532.

[16] Gallego, S. V. *et al.* MAGNDATA: towards a database of magnetic structures. II. The incommensurate case. *Journal of Applied Crystallography* **49**, 1941–1956 (2016). URL http://scripts.iucr.org/cgi-bin/paper?ks5530.

[17] Gallego, S. V., Tasci, E. S., de la Flor, G., Perez-Mato, J. M. & Aroyo, M. I. Magnetic symmetry in the Bilbao Crystallographic Server: A computer program to provide systematic absences of magnetic neutron diffraction. *Journal of Applied Crystallography* **45**, 1236–1247 (2012). URL https://doi.org/10.1107/S0021889812042185.

[18] Perez-Mato, J. *et al.* Symmetry-based computational tools for magnetic crystallography. *Annual Review of Materials Research* **45**, 217–248 (2015). URL https://doi.org/10.1146/annurev-matsci-070214-021008. https://doi.org/10.1146/annurev-matsci-070214-021008.

[19] Wang, C., Duan, W., Glazman, L. & Alexandradinata, A. Landau quantization of nearly degenerate bands and full symmetry classification of Landau level crossings. *Phys. Rev. B* **100**, 014442 (2019). URL https://link.aps.org/doi/10.1103/PhysRevB.100.014442.

[20] Zak, J. Band representations and symmetry types of bands in solids. *Phys. Rev. B* **23**, 2824–2835 (1981). URL https://link.aps.org/doi/10.1103/PhysRevB.23.2824.

[21] Zak, J. Band representations of space groups. *Phys. Rev. B* **26**, 3010–3023 (1982). URL https://link.aps.org/doi/10.1103/PhysRevB.26.3010.

[22] Cano, J. & Bradlyn, B. Band representations and topological quantum chemistry. *Annual Review of Condensed Matter Physics* **12**, 225–246 (2021). URL https://doi.org/10.1146/annurev-conmatphys-041720-124134.

[23] Kruthoff, J., de Boer, J., van Wezel, J., Kane, C. L. & Slager, R.-J. Topological classification of crystalline insulators through band structure combinatorics. *Phys. Rev. X* **7**, 041069 (2017). URL https://link.aps.org/doi/10.1103/PhysRevX.7.

041069.

[24] Po, H. C., Vishwanath, A. & Watanabe, H. Symmetry-based indicators of band topology in the 230 space groups. *Nature Communications* **8**, 50 (2017). URL https://doi.org/10.1038/s41467-017-00133-2.

[25] Song, Z., Fang, Z. & Fang, C. $(d-2)$-dimensional edge states of rotation symmetry protected topological states. *Phys. Rev. Lett.* **119**, 246402 (2017). URL https://link.aps.org/doi/10.1103/PhysRevLett.119.246402.

[26] Song, Z., Zhang, T., Fang, Z. & Fang, C. Quantitative mappings between symmetry and topology in solids. *Nature Communications* **9**, 3530 (2018). URL https://doi.org/10.1038/s41467-018-06010-w.

[27] Khalaf, E., Po, H. C., Vishwanath, A. & Watanabe, H. Symmetry indicators and anomalous surface states of topological crystalline insulators. *Phys. Rev. X* **8**, 031070 (2018). URL https://link.aps.org/doi/10.1103/PhysRevX.8.031070.

[28] Wang, Z., Wieder, B. J., Li, J., Yan, B. & Bernevig, B. A. Higher-Order Topology, Monopole Nodal Lines, and the Origin of Large Fermi Arcs in Transition Metal Dichalcogenides $X\mathrm{Te}_2$ $(X = \mathrm{Mo},\mathrm{W})$. *Phys. Rev. Lett.* **123**, 186401 (2019). URL https://link.aps.org/doi/10.1103/PhysRevLett.123.186401.

[29] Song, Z., Zhang, T. & Fang, C. Diagnosis for nonmagnetic topological semimetals in the absence of spin-orbital coupling. *Phys. Rev. X* **8**, 031069 (2018). URL https://link.aps.org/doi/10.1103/PhysRevX.8.031069.

[30] Po, H. C. Symmetry indicators of band topology. *Journal of Physics: Condensed Matter* **32**, 263001 (2020). URL https://doi.org/10.1088%2F1361-648x%2Fab7adb.

[31] Ono, S., Po, H. C. & Shiozaki, K. $\mathbb{Z}_2$-enriched symmetry indicators for topological superconductors in the 1651 magnetic space groups. *Phys. Rev. Research* **3**, 023086 (2021). URL https://link.aps.org/doi/10.1103/PhysRevResearch.3.023086.

[32] Fu, L. & Kane, C. L. Topological insulators with inversion symmetry. *Phys. Rev. B* **76**, 045302 (2007). URL https://link.aps.org/doi/10.1103/PhysRevB.76.045302.

[33] Bradley, C. & Cracknell, A. *The Mathematical Theory of Symmetry in Solids: Representation Theory for Point Groups and Space Groups* (Clarendon Press, 1972). URL https://books.google.com/books?id=OKXvAAAAMAAJ.

[34] Schindler, F. *et al.* Higher-order topological insulators. *Science Advances* **4** (2018). URL http://advances.sciencemag.org/content/4/6/eaat0346.

[35] Schindler, F. *et al.* Higher-order topology in bismuth. *Nature Physics* **14**, 918–924 (2018). URL https://doi.org/10.1038/s41567-018-0224-7.

[36] Fang, C. & Fu, L. New classes of topological crystalline insulators having surface rotation anomaly. *Science Advances* **5** (2019). URL https://advances.sciencemag.org/content/5/12/eaat2374.

[37] Langbehn, J., Peng, Y., Trifunovic, L., von Oppen, F. & Brouwer, P. W. Reflection-symmetric second-order topological insulators and superconductors. *Phys. Rev. Lett.* **119**, 246401 (2017). URL https://link.aps.org/doi/10.1103/PhysRevLett.119.246401.

[38] Wieder, B. J. & Bernevig, B. A. The Axion Insulator as a Pump of Fragile Topology. *arXiv e-prints* arXiv:1810.02373 (2018). 1810.02373.

[39] Wieder, B. J. *et al.* Wallpaper fermions and the nonsymmorphic Dirac insulator. *Science* **361**, 246–251 (2018). URL http://science.sciencemag.org/content/361/6399/246.

[40] Wilczek, F. Two applications of axion electrodynamics. *Phys. Rev. Lett.* **58**, 1799–1802 (1987). URL https://link.aps.org/doi/10.1103/PhysRevLett.58.1799.

[41] Qi, X.-L., Hughes, T. L. & Zhang, S.-C. Topological field theory of time-reversal invariant insulators. *Phys. Rev. B* **78**, 195424 (2008). URL https://link.aps.org/doi/10.1103/PhysRevB.78.195424.

[42] Essin, A. M., Moore, J. E. & Vanderbilt, D. Magnetoelectric polarizability and axion electrodynamics in crystalline insulators. *Phys. Rev. Lett.* **102**, 146805 (2009). URL https://link.aps.org/doi/10.1103/PhysRevLett.102.146805.

[43] Hughes, T. L., Prodan, E. & Bernevig, B. A. Inversion-symmetric topological insulators. *Phys. Rev. B* **83**, 245132 (2011). URL https://link.aps.org/doi/10.1103/PhysRevB.83.245132.

[44] Turner, A. M., Zhang, Y., Mong, R. S. K. & Vishwanath, A. Quantized response and topology of magnetic insulators with inversion symmetry. *Phys. Rev. B* **85**, 165120 (2012). URL https://link.aps.org/doi/10.1103/PhysRevB.85.165120.

[45] Xu, Y., Song, Z., Wang, Z., Weng, H. & Dai, X. Higher-Order Topology of the Axion Insulator $\mathrm{EuIn_2As_2}$. *Phys. Rev. Lett.* **122**, 256402 (2019). URL https://link.aps.org/doi/10.1103/PhysRevLett.122.256402.

[46] Varnava, N. & Vanderbilt, D. Surfaces of axion insulators. *Phys. Rev. B* **98**, 245117 (2018). URL https://link.aps.org/doi/10.1103/PhysRevB.98.245117.

[47] Varnava, N., Souza, I. & Vanderbilt, D. Axion coupling in the hybrid Wannier representation. *Phys. Rev. B* **101**, 155130 (2020). URL https://link.aps.org/doi/10.1103/PhysRevB.101.155130.

[48] Ahn, J. & Yang, B.-J. Symmetry representation approach to topological invariants in $C_{2z}T$-symmetric systems. *Phys. Rev. B* **99**, 235125 (2019). URL https://link.aps.org/doi/10.1103/PhysRevB.99.235125.

[49] Coh, S. & Vanderbilt, D. Canonical magnetic insulators with isotropic magnetoelectric coupling. *Phys. Rev. B* **88**, 121106

(2013). URL https://link.aps.org/doi/10.1103/PhysRevB.88.121106.

[50] Alexandradinata, A., Nelson, A. & Soluyanov, A. A. Teleportation of Berry curvature on the surface of a Hopf insulator. *Phys. Rev. B* **103**, 045107 (2021). URL https://link.aps.org/doi/10.1103/PhysRevB.103.045107.

[51] Klett, M. *et al.* Topology and magnetism in the Kondo insulator phase diagram. *Phys. Rev. B* **101**, 161112 (2020). URL https://link.aps.org/doi/10.1103/PhysRevB.101.161112.

[52] Ahn, J., Kim, D., Kim, Y. & Yang, B.-J. Band topology and linking structure of nodal line semimetals with Z_2 monopole charges. *Phys. Rev. Lett.* **121**, 106403 (2018). URL https://link.aps.org/doi/10.1103/PhysRevLett.121.106403.

[53] Kim, H., Shiozaki, K. & Murakami, S. Glide-symmetric magnetic topological crystalline insulators with inversion symmetry. *Phys. Rev. B* **100**, 165202 (2019). URL https://link.aps.org/doi/10.1103/PhysRevB.100.165202.

[54] Takahashi, R., Tanaka, Y. & Murakami, S. Bulk-edge and bulk-hinge correspondence in inversion-symmetric insulators. *Phys. Rev. Research* **2**, 013300 (2020). URL https://link.aps.org/doi/10.1103/PhysRevResearch.2.013300.

[55] Okuma, N., Sato, M. & Shiozaki, K. Topological classification under nonmagnetic and magnetic point group symmetry: Application of real-space Atiyah-Hirzebruch spectral sequence to higher-order topology. *Phys. Rev. B* **99**, 085127 (2019). URL https://link.aps.org/doi/10.1103/PhysRevB.99.085127.

[56] Lee, E., Furusaki, A. & Yang, B.-J. Fractional charge bound to a vortex in two-dimensional topological crystalline insulators. *Phys. Rev. B* **101**, 241109 (2020). URL https://link.aps.org/doi/10.1103/PhysRevB.101.241109.

[57] Wieder, B. J., Lin, K.-S. & Bradlyn, B. Axionic band topology in inversion-symmetric Weyl-charge-density waves. *Phys. Rev. Research* **2**, 042010 (2020). URL https://link.aps.org/doi/10.1103/PhysRevResearch.2.042010.

[58] Olsen, T., Taherinejad, M., Vanderbilt, D. & Souza, I. Surface theorem for the Chern-Simons axion coupling. *Phys. Rev. B* **95**, 075137 (2017). URL https://link.aps.org/doi/10.1103/PhysRevB.95.075137.

[59] Olsen, T., Rauch, T., Vanderbilt, D. & Souza, I. Gapless hinge states from adiabatic pumping of axion coupling. *Phys. Rev. B* **102**, 035166 (2020). URL https://link.aps.org/doi/10.1103/PhysRevB.102.035166.

[60] van Miert, G. & Ortix, C. Higher-order topological insulators protected by inversion and rotoinversion symmetries. *Phys. Rev. B* **98**, 081110 (2018). URL https://link.aps.org/doi/10.1103/PhysRevB.98.081110.

[61] Zamorzaev, A. M. *Generalization of Fedorov Groups*. Dissertation, Leningrad University, Leningrad (1953).

[62] Belov, N., Neronova, N. & Smirnova, T. The 1651 Shubnikov groups. *Trudy Inst. Kristall.* **11**, 33–67 (1955).

[63] Shubnikov, A., Belov, N. & Shubnikova, A. *Colored Symmetry* (Macmillan, 1964). URL https://books.google.ca/books?id=QKk9AAAAIAAJ.

[64] Yang, J., Liu, Z.-X. & Fang, C. Unlocking of time reversal, space-time inversion and rotation invariants in magnetic materials. *arXiv e-prints* arXiv:2009.07864 (2020). 2009.07864.

[65] Watanabe, H., Po, H. C. & Vishwanath, A. Structure and topology of band structures in the 1651 magnetic space groups. *Science Advances* **4** (2018). URL https://advances.sciencemag.org/content/4/8/eaat8685.

[66] Watanabe, H. Lieb-Schultz-Mattis-type filling constraints in the 1651 magnetic space groups. *Phys. Rev. B* **97**, 165117 (2018). URL https://link.aps.org/doi/10.1103/PhysRevB.97.165117.

[67] Belov, N. & Neronova, T., N.N .and Smirnova. Shubnikov groups. *Sov. Phys. Crystallogr.* **2**, 311 (1957).

[68] Opechowski, W. & Guccione, R. *Magnetism, Ch. 3*, vol. 2A (New York: Academic Press, 1965).

[69] Aroyo, M. I. (ed.) *International Tables for Crystallography, Volume A: Space-Group Symmetry*, vol. A (International Union of Crystallography, 2016). URL http://it.iucr.org/A/.

[70] Kopsky, V. & Litvin, D. *International Tables for Crystallography, Volume E: Subperiodic Groups*. International Tables for Crystallography (Springer Netherlands, 2002). URL https://books.google.com/books?id=if8nMGopkNgC.

[71] Wieder, B. J. *et al.* Strong and fragile topological Dirac semimetals with higher-order Fermi arcs. *Nature Communications* **11**, 627 (2020). URL https://doi.org/10.1038/s41467-020-14443-5.

[72] Cano, J., Bradlyn, B. & Vergniory, M. G. Multifold nodal points in magnetic materials. *APL Materials* **7**, 101125 (2019). URL https://doi.org/10.1063/1.5124314.

[73] Wieder, B. J. & Kane, C. L. Spin-orbit semimetals in the layer groups. *Phys. Rev. B* **94**, 155108 (2016). URL https://link.aps.org/doi/10.1103/PhysRevB.94.155108.

[74] Young, S. M. & Wieder, B. J. Filling-Enforced Magnetic Dirac Semimetals in Two Dimensions. *Phys. Rev. Lett.* **118**, 186401 (2017). URL https://link.aps.org/doi/10.1103/PhysRevLett.118.186401.

[75] Conway, J., Burgiel, H. & Goodman-Strauss, C. *The Symmetries of Things*. Ak Peters Series (Taylor & Francis, 2008). URL https://books.google.com/books?id=EtQCk0TNafsC.

[76] Ascher, E. & Janner, A. Subgroups of black–white point groups. *Acta Crystallographica* **18**, 325–330 (1965). URL https://onlinelibrary.wiley.com/doi/abs/10.1107/S0365110X65000762.

[77] Perez-Mato, J. M., Gallego, S. V., Elcoro, L., Tasci, E. & Aroyo, M. I. Symmetry conditions for type II multiferroicity in commensurate magnetic structures. *Journal of Physics: Condensed Matter* **28**, 286001 (2016). URL http://stacks.iop.

org/0953-8984/28/i=28/a=286001.

78 Miller, S. & Love, W. *Tables of Irreducible Representations of Space Groups and Corepresentations of Magnetic Space Groups* (1967). URL https://books.google.com/books?id=jYQLtAEACAAJ.

79 Aroyo, M. I. *et al.* Brillouin-zone database on the *Bilbao Crystallographic Server*. *Acta Crystallographica Section A* **70**, 126–137 (2014). URL https://doi.org/10.1107/S205327331303091X.

80 Wigner, E. P. Über die operation der zeitumkehr in der quantenmechanik. *Nachr. Akad. Wiss. Göttingen Math.-Phys. Kl.* 546–559 (1932). URL https://doi.org/10.1007/978-3-662-02781-3_15.

81 Wigner, E. & Griffin, J. *Group Theory and Its Application to the Quantum Mechanics of Atomic Spectra* (Academic Press, 1959). URL https://books.google.com/books?id=BZsEAQAAIAAJ.

82 Stokes, H. T., Campbell, B. J. & Cordes, R. Tabulation of irreducible representations of the crystallographic space groups and their superspace extensions. *Acta Crystallographica Section A* **69**, 388–395 (2013). URL https://doi.org/10.1107/S0108767313007538.

83 Michel, L. & Zak, J. Elementary energy bands in crystals are connected. *Physics Reports* **341**, 377 – 395 (2001). URL http://www.sciencedirect.com/science/article/pii/S0370157300000934.

84 Bacry, H., Michel, L. & Zak, J. Symmetry and classification of energy bands in crystals. In Doebner, H.-D., Hennig, J.-D. & Palev, T. D. (eds.) *Group Theoretical Methods in Physics*, 289–308 (Springer Berlin Heidelberg, Berlin, Heidelberg, 1988).

85 Höller, J. & Alexandradinata, A. Topological Bloch oscillations. *Phys. Rev. B* **98**, 024310 (2018). URL https://link.aps.org/doi/10.1103/PhysRevB.98.024310.

86 Michel, L. & Zak, J. Connectivity of energy bands in crystals. *Phys. Rev. B* **59**, 5998–6001 (1999). URL https://link.aps.org/doi/10.1103/PhysRevB.59.5998.

87 Elcoro, L., Song, Z. & Bernevig, B. A. Application of induction procedure and Smith decomposition in calculation and topological classification of electronic band structures in the 230 space groups. *Phys. Rev. B* **102**, 035110 (2020). URL https://link.aps.org/doi/10.1103/PhysRevB.102.035110.

88 Shi, W. *et al.* A charge-density-wave topological semimetal. *Nature Physics* **17**, 381–387 (2021). URL https://doi.org/10.1038/s41567-020-01104-z.

89 Iraola, M. *et al.* IrRep: symmetry eigenvalues and irreducible representations of ab initio band structures. *arXiv e-prints* arXiv:2009.01764 (2020). 2009.01764.

90 Cracknell, A. P. The application of Landau's theory of continuous phase transitions to magnetic phase transitions. *Journal of Physics C: Solid State Physics* **4**, 2488 (1971). URL http://stacks.iop.org/0022-3719/4/i=16/a=014.

91 Samokhin, K. V. & Walker, M. B. Order parameter symmetry in ferromagnetic superconductors. *Phys. Rev. B* **66**, 174501 (2002). URL https://link.aps.org/doi/10.1103/PhysRevB.66.174501.

92 Micklitz, T. & Norman, M. R. Odd parity and line nodes in nonsymmorphic superconductors. *Phys. Rev. B* **80**, 100506 (2009). URL https://link.aps.org/doi/10.1103/PhysRevB.80.100506.

93 Sumita, S., Nomoto, T. & Yanase, Y. Multipole Superconductivity in Nonsymmorphic Sr_2IrO_4. *Phys. Rev. Lett.* **119**, 027001 (2017). URL https://link.aps.org/doi/10.1103/PhysRevLett.119.027001.

94 Cao, K., Giustino, F. & Radaelli, P. G. Theory of Electromagnons in CuO. *Phys. Rev. Lett.* **114**, 197201 (2015). URL https://link.aps.org/doi/10.1103/PhysRevLett.114.197201.

95 Benfatto, L. *et al.* Field dependence of the magnetic spectrum in anisotropic and Dzyaloshinskii-Moriya antiferromagnets. II. Raman spectroscopy. *Phys. Rev. B* **74**, 024416 (2006). URL https://link.aps.org/doi/10.1103/PhysRevB.74.024416.

96 Herring, C. Character tables for two space groups. *Journal of the Franklin Institute* **233**, 525 – 543 (1942). URL http://www.sciencedirect.com/science/article/pii/S0016003242904629.

97 Frobenius, G. & Schur, I. *Über die reellen Darstellungen der endlichen Gruppen: Über die Äquivalenz der Gruppen linearer Substitutionen. Von G. Frobenius und I. Schur*. Preussische Akademie der Wissenschaften Berlin: Sitzungsberichte der Preußischen Akademie der Wissenschaften zu Berlin (Reichsdr., 1906). URL https://books.google.com/books?id=lyU-cgAACAAJ.

98 Fulton, W., Harris, W. & Harris, J. *Representation Theory: A First Course*. Graduate Texts in Mathematics (Springer New York, 1991). URL https://books.google.com/books?id=6GUH8ARxhp8C.

99 Dimmock, J. & Wheeler, R. Irreducible representations of magnetic groups. *Journal of Physics and Chemistry of Solids* **23**, 729 – 741 (1962). URL http://www.sciencedirect.com/science/article/pii/0022369762905310.

100 Young, S. M. *et al.* Dirac Semimetal in Three Dimensions. *Phys. Rev. Lett.* **108**, 140405 (2012). URL http://link.aps.org/doi/10.1103/PhysRevLett.108.140405.

101 Watanabe, H., Po, H. C., Vishwanath, A. & Zaletel, M. Filling constraints for spin-orbit coupled insulators in symmorphic and nonsymmorphic crystals. *Proceedings of the National Academy of Sciences* **112**, 14551–14556 (2015). URL http://www.pnas.org/content/112/47/14551.abstract.

102 Mañes, J. L. Existence of bulk chiral fermions and crystal symmetry. *Phys. Rev. B* **85**, 155118 (2012). URL https:

//link.aps.org/doi/10.1103/PhysRevB.85.155118.

103 Wieder, B. J., Kim, Y., Rappe, A. M. & Kane, C. L. Double Dirac Semimetals in Three Dimensions. *Phys. Rev. Lett.* **116**, 186402 (2016). URL http://link.aps.org/doi/10.1103/PhysRevLett.116.186402.

104 Bradlyn, B. *et al.* Beyond Dirac and Weyl fermions: Unconventional quasiparticles in conventional crystals. *Science* **353** (2016). URL http://science.sciencemag.org/content/353/6299/aaf5037.

105 Chang, G. *et al.* Unconventional Chiral Fermions and Large Topological Fermi Arcs in RhSi. *Phys. Rev. Lett.* **119**, 206401 (2017). URL https://link.aps.org/doi/10.1103/PhysRevLett.119.206401.

106 Tang, P., Zhou, Q. & Zhang, S.-C. Multiple Types of Topological Fermions in Transition Metal Silicides. *Phys. Rev. Lett.* **119**, 206402 (2017). URL https://link.aps.org/doi/10.1103/PhysRevLett.119.206402.

107 Chang, G. *et al.* Topological quantum properties of chiral crystals. *Nature Materials* **17**, 978–985 (2018). URL https://doi.org/10.1038/s41563-018-0169-3.

108 Scott, L. & Serre, J. *Linear Representations of Finite Groups*. Graduate Texts in Mathematics (Springer New York, 1996). URL https://books.google.com/books?id=NCfZgr54TJ4C.

109 Vergniory, M. G. *et al.* A complete catalogue of high-quality topological materials. *Nature* **566**, 480–485 (2019). URL https://doi.org/10.1038/s41586-019-0954-4.

110 Xu, Y. *et al.* High-throughput calculations of magnetic topological materials. *Nature* **586**, 702–707 (2020). URL https://doi.org/10.1038/s41586-020-2837-0.

111 Young, S. M. & Kane, C. L. Dirac Semimetals in Two Dimensions. *Phys. Rev. Lett.* **115**, 126803 (2015). URL http://link.aps.org/doi/10.1103/PhysRevLett.115.126803.

112 Wang, Z., Alexandradinata, A., Cava, R. J. & Bernevig, B. A. Hourglass fermions. *Nature* **532**, 189–194 (2016). URL https://doi.org/10.1038/nature17410.

113 Alexandradinata, A., Wang, Z. & Bernevig, B. A. Topological insulators from group cohomology. *Phys. Rev. X* **6**, 021008 (2016). URL https://link.aps.org/doi/10.1103/PhysRevX.6.021008.

114 Marzari, N. & Vanderbilt, D. Maximally localized generalized Wannier functions for composite energy bands. *Phys. Rev. B* **56**, 12847–12865 (1997). URL https://link.aps.org/doi/10.1103/PhysRevB.56.12847.

115 Marzari, N., Mostofi, A. A., Yates, J. R., Souza, I. & Vanderbilt, D. Maximally localized Wannier functions: Theory and applications. *Rev. Mod. Phys.* **84**, 1419–1475 (2012). URL https://link.aps.org/doi/10.1103/RevModPhys.84.1419.

116 Zak, J. Berry's phase for energy bands in solids. *Phys. Rev. Lett.* **62**, 2747–2750 (1989). URL https://link.aps.org/doi/10.1103/PhysRevLett.62.2747.

117 King-Smith, R. D. & Vanderbilt, D. Theory of polarization of crystalline solids. *Phys. Rev. B* **47**, 1651–1654 (1993). URL https://link.aps.org/doi/10.1103/PhysRevB.47.1651.

118 Soluyanov, A. A. & Vanderbilt, D. Smooth gauge for topological insulators. *Phys. Rev. B* **85**, 115415 (2012). URL https://link.aps.org/doi/10.1103/PhysRevB.85.115415.

119 Yu, R., Qi, X. L., Bernevig, A., Fang, Z. & Dai, X. Equivalent expression of $\mathbb{Z}_2$ topological invariant for band insulators using the non-Abelian Berry connection. *Phys. Rev. B* **84**, 075119 (2011). URL https://link.aps.org/doi/10.1103/PhysRevB.84.075119.

120 Fidkowski, L., Jackson, T. S. & Klich, I. Model Characterization of Gapless Edge Modes of Topological Insulators Using Intermediate Brillouin-Zone Functions. *Phys. Rev. Lett.* **107**, 036601 (2011). URL https://link.aps.org/doi/10.1103/PhysRevLett.107.036601.

121 Alexandradinata, A., Dai, X. & Bernevig, B. A. Wilson-loop characterization of inversion-symmetric topological insulators. *Phys. Rev. B* **89**, 155114 (2014). URL https://link.aps.org/doi/10.1103/PhysRevB.89.155114.

122 Benalcazar, W. A., Bernevig, B. A. & Hughes, T. L. Quantized electric multipole insulators. *Science* **357**, 61–66 (2017). URL http://science.sciencemag.org/content/357/6346/61.

123 Benalcazar, W. A., Bernevig, B. A. & Hughes, T. L. Electric multipole moments, topological multipole moment pumping, and chiral hinge states in crystalline insulators. *Phys. Rev. B* **96**, 245115 (2017). URL https://link.aps.org/doi/10.1103/PhysRevB.96.245115.

124 Schindler, F., Tsirkin, S. S., Neupert, T., Bernevig, B. A. & Wieder, B. J. *In Preparation*.

125 Kooi, S. H., van Miert, G. & Ortix, C. Classification of crystalline insulators without symmetry indicators: Atomic and fragile topological phases in twofold rotation symmetric systems. *Phys. Rev. B* **100**, 115160 (2019). URL https://link.aps.org/doi/10.1103/PhysRevB.100.115160.

126 Alexandradinata, A., Höller, J., Wang, C., Cheng, H. & Lu, L. Crystallographic splitting theorem for band representations and fragile topological photonic crystals. *Phys. Rev. B* **102**, 115117 (2020). URL https://link.aps.org/doi/10.1103/PhysRevB.102.115117.

127 Berg, M., De Witt-Morette, C., Gwo, S. & Kramer, E. The Pin Groups in Physics: C, P and T. *Reviews in Mathematical Physics* **13**, 953–1034 (2001). URL https://doi.org/10.1142/S0129055X01000922.

[128] McQuarrie, D. A. & Simon, J. D. *Physical Chemistry: A Molecular Approach* (University Science Books, 1997). URL https://books.google.com/books?id=f-bje0-DEYUC.

[129] Blanco, M. A., Flórez, M. & Bermejo, M. Evaluation of the rotation matrices in the basis of real spherical harmonics. *Journal of Molecular Structure: THEOCHEM* **419**, 19 – 27 (1997). URL http://www.sciencedirect.com/science/article/pii/S0166128097001851.

[130] Muggli, J. Cubic harmonics as linear combinations of spherical harmonics. *Zeitschrift für angewandte Mathematik und Physik ZAMP* **23**, 311–317 (1972). URL https://doi.org/10.1007/BF01593094.

[131] Bethe, H. Termaufspaltung in kristallen. *Annalen der Physik* **395**, 133–208 (1929). URL https://onlinelibrary.wiley.com/doi/abs/10.1002/andp.19293950202.

[132] Van Vleck, J. H. Theory of the Variations in Paramagnetic Anisotropy Among Different Salts of the Iron Group. *Phys. Rev.* **41**, 208–215 (1932). URL https://link.aps.org/doi/10.1103/PhysRev.41.208.

[133] Mulliken, R. S. Electronic Structures of Polyatomic Molecules and Valence. IV. Electronic States, Quantum Theory of the Double Bond. *Phys. Rev.* **43**, 279–302 (1933). URL https://link.aps.org/doi/10.1103/PhysRev.43.279.

[134] Setyawan, W. & Curtarolo, S. High-throughput electronic band structure calculations: Challenges and tools. *Computational Materials Science* **49**, 299 – 312 (2010). URL http://www.sciencedirect.com/science/article/pii/S0927025610002697.

[135] Yu, P. & Cardona, M. *Fundamentals of Semiconductors: Physics and Materials Properties*. No. v. 3 in Advanced texts in physics (Springer Berlin Heidelberg, 2005). URL https://books.google.com/books?id=W9pdJZoAeyEC.

[136] Po, H. C., Watanabe, H. & Vishwanath, A. Fragile Topology and Wannier Obstructions. *Phys. Rev. Lett.* **121**, 126402 (2018). URL https://link.aps.org/doi/10.1103/PhysRevLett.121.126402.

[137] Liu, S., Vishwanath, A. & Khalaf, E. Shift insulators: Rotation-protected two-dimensional topological crystalline insulators. *Phys. Rev. X* **9**, 031003 (2019). URL https://link.aps.org/doi/10.1103/PhysRevX.9.031003.

[138] Bouhon, A., Black-Schaffer, A. M. & Slager, R.-J. Wilson loop approach to fragile topology of split elementary band representations and topological crystalline insulators with time-reversal symmetry. *Phys. Rev. B* **100**, 195135 (2019). URL https://link.aps.org/doi/10.1103/PhysRevB.100.195135.

[139] Ahn, J., Park, S. & Yang, B.-J. Failure of Nielsen-Ninomiya Theorem and Fragile Topology in Two-Dimensional Systems with Space-Time Inversion Symmetry: Application to Twisted Bilayer Graphene at Magic Angle. *Phys. Rev. X* **9**, 021013 (2019). URL https://link.aps.org/doi/10.1103/PhysRevX.9.021013.

[140] Song, Z.-D., Elcoro, L., Xu, Y.-F., Regnault, N. & Bernevig, B. A. Fragile phases as affine monoids: Classification and material examples. *Phys. Rev. X* **10**, 031001 (2020). URL https://link.aps.org/doi/10.1103/PhysRevX.10.031001.

[141] Peri, V. *et al.* Experimental characterization of fragile topology in an acoustic metamaterial. *Science* **367**, 797–800 (2020). URL https://science.sciencemag.org/content/367/6479/797.

[142] Song, Z. *et al.* All magic angles in twisted bilayer graphene are topological. *Phys. Rev. Lett.* **123**, 036401 (2019). URL https://link.aps.org/doi/10.1103/PhysRevLett.123.036401.

[143] Song, Z.-D., Elcoro, L. & Bernevig, B. A. Twisted bulk-boundary correspondence of fragile topology. *Science* **367**, 794–797 (2020). URL https://science.sciencemag.org/content/367/6479/794.

[144] Hwang, Y., Ahn, J. & Yang, B.-J. Fragile topology protected by inversion symmetry: Diagnosis, bulk-boundary correspondence, and Wilson loop. *Phys. Rev. B* **100**, 205126 (2019). URL https://link.aps.org/doi/10.1103/PhysRevB.100.205126.

[145] Alexandradinata, A. & Höller, J. No-go theorem for topological insulators and high-throughput identification of Chern insulators. *Phys. Rev. B* **98**, 184305 (2018). URL https://link.aps.org/doi/10.1103/PhysRevB.98.184305.

[146] Nelson, A., Neupert, T., Bzdušek, T. c. v. & Alexandradinata, A. Multicellularity of delicate topological insulators. *Phys. Rev. Lett.* **126**, 216404 (2021). URL https://link.aps.org/doi/10.1103/PhysRevLett.126.216404.

[147] Bouhon, A., Lange, G. F. & Slager, R.-J. Topological correspondence between magnetic space group representations and subdimensions. *Phys. Rev. B* **103**, 245127 (2021). URL https://link.aps.org/doi/10.1103/PhysRevB.103.245127.

[148] Lange, G. F., Bouhon, A. & Slager, R.-J. Subdimensional topologies, indicators, and higher order boundary effects. *Phys. Rev. B* **103**, 195145 (2021). URL https://link.aps.org/doi/10.1103/PhysRevB.103.195145.

[149] Cano, J., Elcoro, L., Aroyo, M. I., Bernevig, B. A. & Bradlyn, B. Topology invisible to eigenvalues in obstructed atomic insulators. *arXiv e-prints* arXiv:2107.00647 (2021). 2107.00647.

[150] Watanabe, H., Po, H. C., Zaletel, M. P. & Vishwanath, A. Filling-enforced gaplessness in band structures of the 230 space groups. *Phys. Rev. Lett.* **117**, 096404 (2016). URL https://link.aps.org/doi/10.1103/PhysRevLett.117.096404.

[151] Po, H. C., Watanabe, H., Zaletel, M. P. & Vishwanath, A. Filling-enforced quantum band insulators in spin-orbit coupled crystals. *Science Advances* **2** (2016). URL https://advances.sciencemag.org/content/2/4/e1501782.

[152] Lieb, E., Schultz, T. & Mattis, D. Two soluble models of an antiferromagnetic chain. *Annals of Physics* **16**, 407 – 466 (1961). URL http://www.sciencedirect.com/science/article/pii/0003491661901154.

[153] Else, D. V. & Thorngren, R. Topological theory of Lieb-Schultz-Mattis theorems in quantum spin systems. *Phys. Rev. B*

101, 224437 (2020). URL https://link.aps.org/doi/10.1103/PhysRevB.101.224437.

154 Kim, Y., Wieder, B. J., Kane, C. L. & Rappe, A. M. Dirac Line Nodes in Inversion-Symmetric Crystals. *Phys. Rev. Lett.* **115**, 036806 (2015). URL https://link.aps.org/doi/10.1103/PhysRevLett.115.036806.

155 Thouless, D. J. Wannier functions for magnetic sub-bands. *Journal of Physics C: Solid State Physics* **17**, L325 (1984). URL http://stacks.iop.org/0022-3719/17/i=12/a=003.

156 Qi, X.-L., Wu, Y.-S. & Zhang, S.-C. Topological quantization of the spin Hall effect in two-dimensional paramagnetic semiconductors. *Phys. Rev. B* **74**, 085308 (2006). URL https://link.aps.org/doi/10.1103/PhysRevB.74.085308.

157 Soluyanov, A. A. & Vanderbilt, D. Wannier representation of $\mathbb{Z}_2$ topological insulators. *Phys. Rev. B* **83**, 035108 (2011). URL https://link.aps.org/doi/10.1103/PhysRevB.83.035108.

158 Fu, L. & Kane, C. L. Time reversal polarization and a Z_2 adiabatic spin pump. *Phys. Rev. B* **74**, 195312 (2006). URL https://link.aps.org/doi/10.1103/PhysRevB.74.195312.

159 Moore, J. E. & Balents, L. Topological invariants of time-reversal-invariant band structures. *Phys. Rev. B* **75**, 121306 (2007). URL http://link.aps.org/doi/10.1103/PhysRevB.75.121306.

160 Fu, L., Kane, C. L. & Mele, E. J. Topological insulators in three dimensions. *Phys. Rev. Lett.* **98**, 106803 (2007). URL https://link.aps.org/doi/10.1103/PhysRevLett.98.106803.

161 Hsieh, T. H. *et al.* Topological crystalline insulators in the SnTe material class. *Nature Communications* **3**, 982 (2012). URL https://doi.org/10.1038/ncomms1969.

162 Teo, J. C. Y., Fu, L. & Kane, C. L. Surface states and topological invariants in three-dimensional topological insulators: Application to $Bi_{1-x}Sb_x$. *Phys. Rev. B* **78**, 045426 (2008). URL https://link.aps.org/doi/10.1103/PhysRevB.78.045426.

163 Fu, L. Topological crystalline insulators. *Phys. Rev. Lett.* **106**, 106802 (2011). URL https://link.aps.org/doi/10.1103/PhysRevLett.106.106802.

164 Fang, C., Gilbert, M. J. & Bernevig, B. A. Bulk topological invariants in noninteracting point group symmetric insulators. *Phys. Rev. B* **86**, 115112 (2012). URL https://link.aps.org/doi/10.1103/PhysRevB.86.115112.

165 Damnjanović, M., Milošević, I. & Vujičić, M. Magnetic line groups. II. Corepresentations of the magnetic line groups isogonal to the point groups C_n, S_{2n}, and C_{nh}. *Phys. Rev. B* **39**, 4610–4619 (1989). URL https://link.aps.org/doi/10.1103/PhysRevB.39.4610.

166 Damnjanović, M. & Milošević, I. Magnetic line groups. III. Corepresentations of the magnetic line groups isogonal to the point groups D_n, C_{nv}, D_{nd}, and D_{nh}. *Phys. Rev. B* **43**, 13482–13500 (1991). URL https://link.aps.org/doi/10.1103/PhysRevB.43.13482.

167 Song, H., Huang, S.-J., Fu, L. & Hermele, M. Topological phases protected by point group symmetry. *Phys. Rev. X* **7**, 011020 (2017). URL https://link.aps.org/doi/10.1103/PhysRevX.7.011020.

168 Song, Z., Huang, S.-J., Qi, Y., Fang, C. & Hermele, M. Topological states from topological crystals. *Science Advances* **5**, eaax2007 (2019). URL https://advances.sciencemag.org/content/5/12/eaax2007.

169 Smith, H. J. S. & Sylvester, J. J. On systems of linear indeterminate equations and congruences. *Philosophical Transactions of the Royal Society of London* **151**, 293–326 (1861). URL https://royalsocietypublishing.org/doi/abs/10.1098/rstl.1861.0016.

170 Tang, F., Po, H. C., Vishwanath, A. & Wan, X. Comprehensive search for topological materials using symmetry indicators. *Nature* **566**, 486–489 (2019). URL https://doi.org/10.1038/s41586-019-0937-5.

171 Zhang, T. *et al.* Catalogue of topological electronic materials. *Nature* **566**, 475–479 (2019). URL https://doi.org/10.1038/s41586-019-0944-6.

172 Vergniory, M. G. *et al.* All Topological Bands of All Stoichiometric Materials. *arXiv e-prints* arXiv:2105.09954 (2021). 2105.09954.

173 Fang, C. & Fu, L. New classes of three-dimensional topological crystalline insulators: Nonsymmorphic and magnetic. *Phys. Rev. B* **91**, 161105 (2015). URL https://link.aps.org/doi/10.1103/PhysRevB.91.161105.

174 Hsieh, D. *et al.* A topological Dirac insulator in a quantum spin Hall phase. *Nature* **452**, 970–974 (2008). URL https://doi.org/10.1038/nature06843.

175 Gao, J. *et al.* High-throughput screening for Weyl semimetals with $S4$ symmetry. *Science Bulletin* **66**, 667–675 (2021). URL https://www.sciencedirect.com/science/article/pii/S2095927320307738.

176 Peng, B., Jiang, Y., Fang, Z., Weng, H. & Fang, C. Topological classification and diagnosis in magnetically ordered electronic materials. *arXiv e-prints* arXiv:2102.12645 (2021). 2102.12645.

177 Mong, R. S. K., Essin, A. M. & Moore, J. E. Antiferromagnetic topological insulators. *Phys. Rev. B* **81**, 245209 (2010). URL https://link.aps.org/doi/10.1103/PhysRevB.81.245209.

178 Wan, X., Turner, A. M., Vishwanath, A. & Savrasov, S. Y. Topological semimetal and Fermi-arc surface states in the electronic structure of pyrochlore iridates. *Phys. Rev. B* **83**, 205101 (2011). URL https://link.aps.org/doi/10.1103/

PhysRevB.83.205101.

[179] Weng, H., Fang, C., Fang, Z., Bernevig, B. A. & Dai, X. Weyl Semimetal Phase in Noncentrosymmetric Transition-Metal Monophosphides. *Phys. Rev. X* **5**, 011029 (2015). URL https://link.aps.org/doi/10.1103/PhysRevX.5.011029.

[180] Xu, S.-Y. *et al.* Discovery of a Weyl fermion semimetal and topological Fermi arcs **349**, 613–617 (2015). URL https://science.sciencemag.org/content/349/6248/613.

[181] Armitage, N. P., Mele, E. J. & Vishwanath, A. Weyl and Dirac semimetals in three-dimensional solids. *Rev. Mod. Phys.* **90**, 015001 (2018). URL https://link.aps.org/doi/10.1103/RevModPhys.90.015001.

[182] Burkov, A. A. & Balents, L. Weyl semimetal in a topological insulator multilayer. *Phys. Rev. Lett.* **107**, 127205 (2011). URL https://link.aps.org/doi/10.1103/PhysRevLett.107.127205.

[183] Khalaf, E. Higher-order topological insulators and superconductors protected by inversion symmetry. *Phys. Rev. B* **97**, 205136 (2018). URL https://link.aps.org/doi/10.1103/PhysRevB.97.205136.

[184] Fang, C., Chen, Y., Kee, H.-Y. & Fu, L. Topological nodal line semimetals with and without spin-orbital coupling. *Phys. Rev. B* **92**, 081201 (2015). URL https://link.aps.org/doi/10.1103/PhysRevB.92.081201.

[185] Li, K., Li, C., Hu, J., Li, Y. & Fang, C. Dirac and Nodal Line Magnons in Three-Dimensional Antiferromagnets. *Phys. Rev. Lett.* **119**, 247202 (2017). URL https://link.aps.org/doi/10.1103/PhysRevLett.119.247202.

[186] Zilberberg, O. *et al.* Photonic topological boundary pumping as a probe of 4D quantum Hall physics. *Nature* **553**, 59–62 (2018). URL https://doi.org/10.1038/nature25011.

[187] Petrides, I. & Zilberberg, O. Higher-order topological insulators, topological pumps and the quantum Hall effect in high dimensions. *Phys. Rev. Research* **2**, 022049 (2020). URL https://link.aps.org/doi/10.1103/PhysRevResearch.2.022049.

[188] Haldane, F. D. M. Model for a Quantum Hall Effect without Landau Levels: Condensed-Matter Realization of the "Parity Anomaly". *Phys. Rev. Lett.* **61**, 2015–2018 (1988). URL https://link.aps.org/doi/10.1103/PhysRevLett.61.2015.

[189] Neupert, T., Santos, L., Chamon, C. & Mudry, C. Fractional Quantum Hall States at Zero Magnetic Field. *Phys. Rev. Lett.* **106**, 236804 (2011). URL https://link.aps.org/doi/10.1103/PhysRevLett.106.236804.

[190] Alvarez-Gaumé, L. & Witten, E. Gravitational anomalies. *Nuclear Physics B* **234**, 269 – 330 (1984). URL http://www.sciencedirect.com/science/article/pii/055032138490066X.

[191] Redlich, A. N. Gauge noninvariance and parity nonconservation of three-dimensional fermions. *Phys. Rev. Lett.* **52**, 18–21 (1984). URL https://link.aps.org/doi/10.1103/PhysRevLett.52.18.

[192] Jackiw, R. Fractional charge and zero modes for planar systems in a magnetic field. *Phys. Rev. D* **29**, 2375–2377 (1984). URL https://link.aps.org/doi/10.1103/PhysRevD.29.2375.

[193] Mulligan, M. & Burnell, F. J. Topological insulators avoid the parity anomaly. *Phys. Rev. B* **88**, 085104 (2013). URL https://link.aps.org/doi/10.1103/PhysRevB.88.085104.

[194] Lapa, M. F. Parity anomaly from the Hamiltonian point of view. *Phys. Rev. B* **99**, 235144 (2019). URL https://link.aps.org/doi/10.1103/PhysRevB.99.235144.

[195] Bernevig, B. A., Hughes, T. L. & Zhang, S.-C. Quantum Spin Hall Effect and Topological Phase Transition in HgTe Quantum Wells. *Science* **314**, 1757–1761 (2006). URL http://science.sciencemag.org/content/314/5806/1757.
